# Genotypic and Phenotypic Diversity of the Replication-Competent HIV Reservoir in Treated Patients

**DOI:** 10.1128/spectrum.00784-22

**Published:** 2022-06-30

**Authors:** Alexandre Nicolas, Julie Migraine, Jacques Dutrieux, Maud Salmona, Alexandra Tauzin, Atsuko Hachiya, Jérôme Estaquier, Jean-Michel Molina, François Clavel, Allan J. Hance, Fabrizio Mammano

**Affiliations:** a INSERM U941, Université Paris-Diderot, Paris, France; b INSERM UMR-1124, Université de Paris, Paris, France; c Université de Paris, Institut Cochin, INSERM, U1016, CNRS, UMR8104, Paris, France; d APHP Hôpital Saint-Louis, Paris, France; e National Hospital Organization Nagoya Medical Center, Nagoya, Japan; f Centre de Recherche du CHU de Québec, Université Laval, Québec, Quebec, Canada; g INSERM U1259 MAVIVH, Université de Tours, Tours, France; Fundacio irsiCaixa

**Keywords:** HIV-1, reservoir, genotypic diversity, treatment, genotypic identification, human immunodeficiency virus

## Abstract

In HIV infection, viral rebound after treatment discontinuation is considered to originate predominantly from viral genomes integrated in resting CD4^+^ T lymphocytes. Replication-competent proviral genomes represent a minority of the total HIV DNA. While the quantification of the HIV reservoir has been extensively studied, the diversity of genomes that compose the reservoir was less explored. Here, we measured the genotypic and phenotypic diversity in eight patients with different treatment histories. Between 4 and 14 (mean, 8) individual viral isolates per patient were obtained using a virus outgrowth assay, and their near-full-length genomes were sequenced. The mean pairwise distance (MPD) observed in different patients correlated with the time before undetectable viremia was achieved (*r* = 0.864, *P* = 0.0194), suggesting that the complexity of the replication-competent reservoir mirrors that present at treatment initiation. No correlation was instead observed between MPD and the duration of successful treatment (mean, 8 years; range, 2 to 21 years). For 5 of the 8 patients, genotypically identical viral isolates were observed in independent wells, suggesting clonal expansion of infected cells. Identical viruses represented between 25 and 60% of the isolates (mean, 48%). The proportion of identical viral isolates correlated with the duration of treatment (*r* = 0.822, *P* = 0.0190), suggesting progressive clonal expansion of infected cells during ART. A broader range of infectivity was also observed among isolates from patients with delayed viremia control (*r* = 0.79, *P* = 0.025). This work unveiled differences in the genotypic and phenotypic features of the replication-competent reservoir from treated patients and suggests that delaying treatment results in increased diversity of the reservoir.

**IMPORTANCE** In HIV-infected and effectively treated individuals, integrated proviral genomes may persist for decades. The vast majority of the genomes, however, are defective, and only the replication-competent fraction represents a threat of viral reemergence. The quantification of the reservoir has been thoroughly explored, while the diversity of the genomes has been insufficiently studied. Its characterization, however, is relevant for the design of strategies aiming the reduction of the reservoir. Here, we explored the replication-competent near-full-length HIV genomes of eight patients who experienced differences in the delay before viremia control and in treatment duration. We found that delayed effective treatment was associated with increased genetic diversity of the reservoir. The duration of treatment did not impact the diversity but was associated with higher frequency of clonally expanded sequences. Thus, early treatment initiation has the double advantage of reducing both the size and the diversity of the reservoir.

## INTRODUCTION

The HIV-1 reservoir consists of long-lived cells harboring integrated proviral genomes, able to lead to viral replication upon cellular activation. These cells are thought to be the origin of the viral rebound when suppressive treatment is interrupted (1–6). The reservoir is seeded from the early phase of infection and initiation of antiretroviral therapy (ART) during this phase reduces total cell-associated HIV DNA, thus delaying viral rebound upon treatment interruption (7–12). Despite the ability of ART to maintain viremia under the detection level and to reduce mortality among HIV-1-infected individuals, lifelong therapy is required, as it does not affect the reservoir. The reservoir is thus considered to be the main obstacle to achieving a cure for HIV-1 (13, 14).

A major effort has been made to characterize the HIV reservoir. It is now clear that the reservoir is relatively stable over time in terms of quantity, with a low rate of decrease in most individuals (15–20). Homeostatic proliferation of latently infected CD4^+^ T cells by clonal expansion plays an important role in maintaining the reservoir (21–33), while it is still unclear if residual replication in sanctuary sites or within tissues with low drug penetration also occurs (34–38). Interestingly, despite the fact that the reservoir is thought to be the origin of the viral rebound during analytical treatment interruption (ATI), there are only a few reports in which the rebound virus could be assigned to sequences present in the reservoir during ART (39, 40). This can be due to limitation in the sampling of the reservoir. In several instances, however, recombination appears to play an important role in viral rebound (39–43).

One of the complexities in the study of the reservoir resides in its definition, which for some authors includes all viral genomes integrated into latent cells that persist during therapy. Numerous studies based on total or integrated HIV DNA, however, have shown that most of the proviruses are defective and thus incompetent for viral emergence (44–47). Next-generation sequencing (NGS) applied to near-full-length viral genome analyses made it possible to distinguish the few intact sequences from the vast majority of defective ones (26, 31, 44, 46, 48). Recently developed surrogate markers of intact genomes (49–51) allowed to estimate their frequency and persistence in large patient cohorts (47). However, even intact sequences may or may not be expressed depending on the integration site, epigenetic restriction, and quality of the stimulation of the cell (44, 52). A series of recent studies have collectively shown that a significant proportion of genomes is expressed under ART, while integration into chromosomal regions that are intrinsically silent favors the persistence of intact sequences both in elite controllers and in patients undergoing long-term ART (53–55). Finally, adding to the complexity, we recently showed that among intact and expressed *env* sequences present in the reservoir of patients, some resulted in the expression of functionally impaired glycoproteins (56).

Measuring the “true reservoir” has always been challenging and a focus of attention. Overall, only a fraction of the few intact genomes that can potentially lead to viral production upon cell activation will produce transcripts, proteins, or infectious viral particles (57). The quantitative virus outgrowth assay (qVOA) is considered the gold standard for the measure and the study of the inducible and replication-competent reservoir, as it allows the outgrowth of viruses through stimulations and culture. Its major limitation is due to the inability to induce all replication-competent genomes, leading to an underestimation of the size of the reservoir (44). Other quantification methods also have specific drawbacks (58), and qVOA has the advantage of leading to production of independent virus isolates from each patient that can be fully characterized.

One feature of the replication-competent reservoir that has not been thoroughly explored concerns the diversity of the viral populations persisting during ART. It is well established that in the absence of treatment, the HIV quasispecies diversify quickly over time (59, 60). By preventing virus replication, effective treatment halts the genetic evolution of the virus (61–65). As the reservoir starts to be seeded early after infection, it will archive the virus genomes over the course of the untreated infection, including transmitted/founder variants (66), although the genomes present at the time of treatment initiation are often overrepresented (66, 67). Analyses of the total proviral DNA have shown that early treatment reduces the reservoir size but also its diversity, compared to those in untreated patients. Only a few studies, however, have begun to explore the diversity of the replication-competent reservoir, and they focused on subgenomic regions (56, 57, 68–70). It is conceivable that the colonization and virus persistence in this specific fraction of the reservoir may display some specificities. The seeding process targets cells in a specific activation state that will favor the establishment of a latent infection. Also, depending on the cellular events that allow maintaining the reservoir, and on the selective pressures that may act on it, the diversity of the replication-competent reservoir could change after years of treatment. Indeed, recent reports have shown that the decay of intact proviruses is faster than the one measured for the largely defective proviral population (49, 51), a phenomenon that is in part compensated for by the accumulation of clonal expansion (31, 51). These quantitative variations may be accompanied by qualitative shifts. Measuring the diversity of the viral populations harbored in the reservoir is important as it informs on the mechanisms that maintain the reservoir over time. It can also provide new insights for therapeutic strategies that aim to reduce the reservoir size, since targeting a heterogeneous reservoir may be expected to be more difficult (71).

In this study, we aimed to define the diversity of the replication-competent reservoir persisting in patients and how it is related to their therapeutic histories. We used qVOA followed by NGS to sequence the near-full-length genomes from outgrown viral particles. We then assessed the diversity by phylogenetic analyses and characterized differences in the infectivity of individual viral isolates. The diversity in each patient was then considered in the context of the time before controlled viremia and effective treatment durations. Our results show that the diversity of the replication-competent reservoir correlates with the delay before viremia control, whereas the frequency of clonal expansion events correlates with the duration of effective treatment.

## RESULTS

### Isolation of replication-competent viruses from the T-cell reservoirs of eight patients.

To assess the diversity of the replication-competent reservoir, we studied eight HIV-infected, ART-treated patients characterized by different treatment histories (Table 1). We did not include patients who were treated during the acute phase of infection, to allow the establishment of a sufficiently diverse reservoir. Also, we only considered patients who were treated for at least 2 years, to allow stabilization of the virus populations in the reservoir and avoid fluctuations induced by the recent introduction of treatment (72). Two of the eight patients received a relatively early treatment (1 and 1.2 years after the estimated time of infection), whereas two other individuals were not diagnosed or efficiently treated for 20 years (Table 1). The duration of effective treatment, defined as persistently undetectable viremia, ranged from 2 to 21 years, and all samples used here were collected within a month after a negative viremia result.

**TABLE 1:** Viral, immunological, and clinical data of individuals participating in the study

Patient	CD4^+^ T cell count (per mm^3^ of blood)	IUPM	No. of isolates	Clones (%)	MPD	Tropism	Time (yrs) before controlled viremia	Effective treatment duration (yrs)
M	1,022	2.05	5	0	NA	CCR5	1	7
X	612	21.5	9	25	0.008	CCR5	1.2	9.3
AE	982	8.4	8	50	0.014	CCR5	9.5	18
V	757	1.57	5	60	0.016	CCR5 & CXCR4	5	21
S	354	0.31	4	0	0.024	CCR5	7.5	2
K	576	1.58	10	0	0.031	CXCR4	14.2	6.2
T	913	9.04	6	50	0.031	CCR5 & CXCR4	20	12.7
AB	681	1.54	14	57	0.052	CCR5 & CXCR4	20	9

To study the reservoir of each patient, we performed a qVOA based on previously described protocols (73, 74), with some modifications. Briefly, we sorted resting CD4^+^ cells by negative selection with antibody-coupled magnetic beads, starting from 40 mL of blood. Cells were plated in individual wells and then activated with anti-CD3 and anti-CD28 bead-coupled antibodies. The next day, they were cultured with phytohemagglutinin (PHA)-activated donor CD4^+^ T cells, allowing viral outgrowth that was detected by p24 enzyme-linked immunosorbent assay (ELISA). We used extreme limiting dilution analysis (ELDA) (75) to estimate the infectious units per million of CD4^+^ cells (IUPM). The IUPM ranged from 0.31 to 21.5, with a median of 1.9. These values are consistent with the viral outgrowth assays performed by other teams (19, 44, 68, 76, 77), validating our approach. The number of viral variants that could be isolated from the replication-competent reservoir ranged from 4 (patient S [patient samples were assigned sequential designations upon receipt in the laboratory]) to 14 (patient AB).

### Near-full-length sequencing and phylogenetic analyses.

To determine the genotypic diversity of the replication-competent reservoir, we used next-generation sequencing to obtain the near-full-length genomes of the isolated viruses. Viral RNA was extracted from the viral particles released in the culture supernatant and then reverse transcribed to cDNA. Viral cDNA was then amplified using 4 sets of primers to obtain 4 overlapping fragments, covering the entire genome as previously described (78). By assembling sequences using HXB2 genome as a reference, we obtained the near-full-length genomes (supplemental file 1) of all the viral isolates for 7 of the 8 patients. For one patient (M), we could sequence only *gag*, *env* and *nef* due to technical issues, limiting the analyses that could be conducted for this patient.

**FIG 1:**
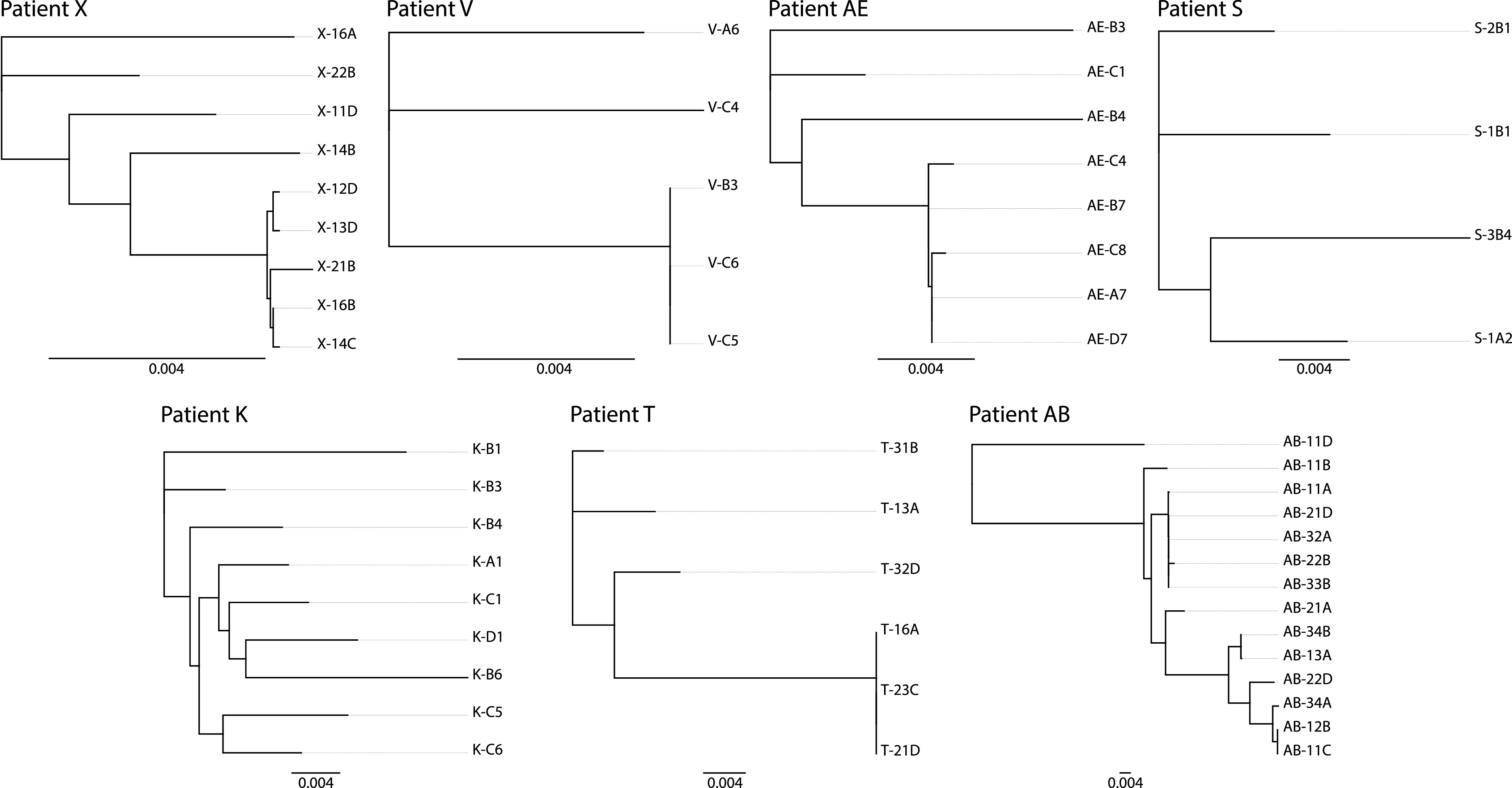
Phylogenetic trees of the near-full-length HIV-1 variants isolated from the replication-competent reservoir of each patient. Maximum-likelihood phylogeny relating within-host near-full-length HIV sequences. Trees were built according to a maximum-likelihood approach using PhyML software. All the trees are represented at the same size in order to facilitate reading of the figure. Bar, 0.004 (all trees). Clusters of identical viral isolates (sequences diverging for less than 4 nucleotides) are indicated by geometric symbols.

To represent diversity and evolution across sequences we used maximum-likelihood methods (PhyML v3.2.2) to build trees for each patient, based on near-full-length genome alignments (Fig. 1). Each tree was bootstrapped 1,000 times. We observed different compositions of the reservoir across the patients, represented by different tree topologies and branch length (Fig. 1). In 5 of the 8 patients, clusters of identical sequences were observed. These sequences are most likely derived from clonal expansion of infected cells. Sequences that differed for fewer than 4 nucleotides on the full genome were considered identical, allowing us to account for rare mutations that may arise in culture or errors introduced during the sequencing workflow (52). Identical sequences represented an average of 48.8% of the total number of sequences (range, 25% to 60%), showing that clonal expansion plays a key role in maintaining the replication-competent viral reservoir (Table 1). For the three other patients, including patient M, for whom only partial sequences are available, all sequences differed.

**FIG 2:**
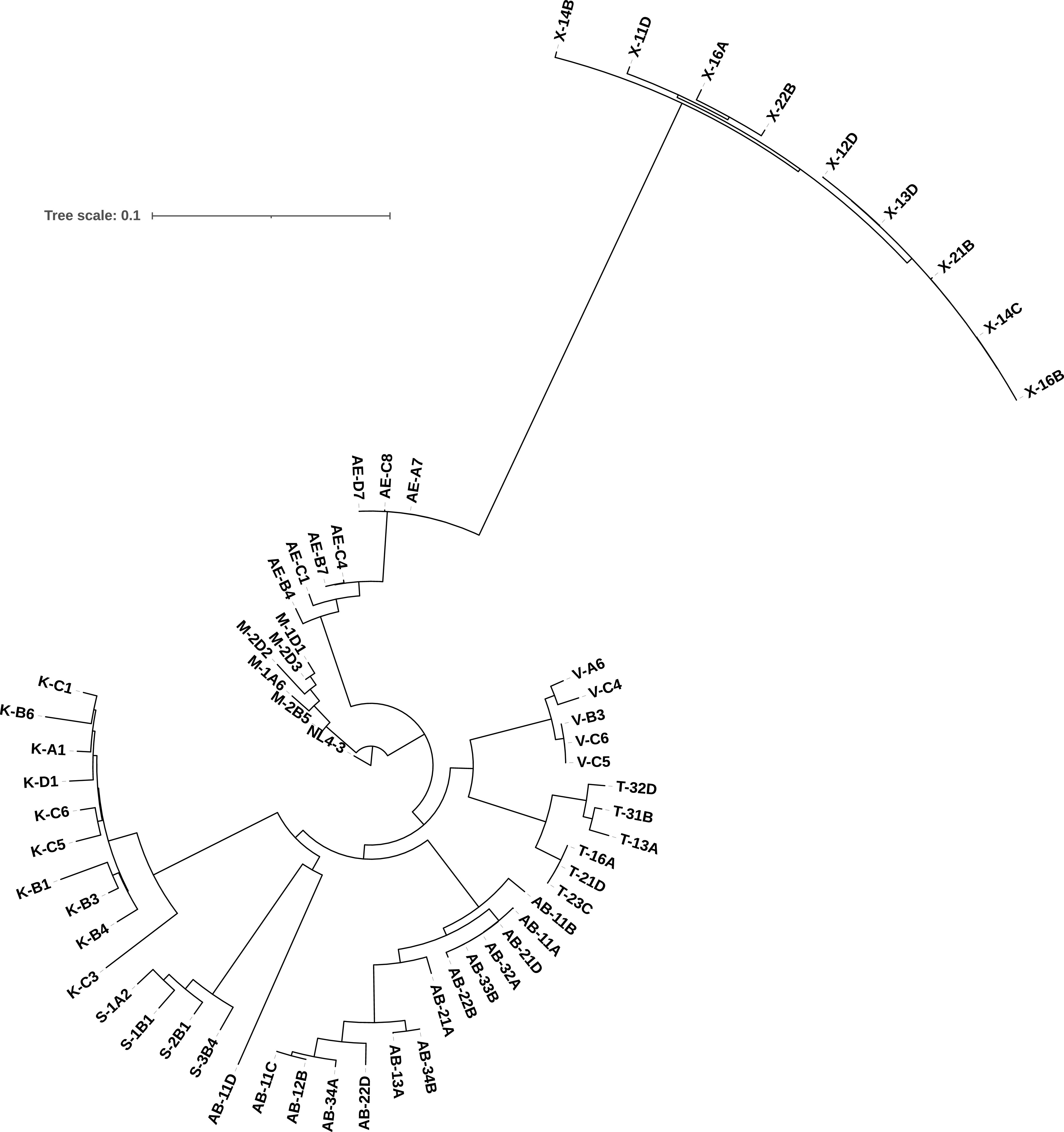
Interpatient phylogenetic tree of replication-competent HIV-1 isolates. Maximum-likelihood phylogeny relating among-host near-full-length HIV sequences. The sequence of the reference strain NL4.3 was used to root the tree. All the sequences of each patient clustered together, with the exception of sequence 11D from patient AB. All patients carried subtype B sequences, with the exception of patient X, who harbored a subtype AG virus population.

Another relevant feature that emerged from the phylogenetic analysis is that patient AB appears to have been infected by two different viruses. Indeed, sequence 11D branches separately from all the other sequences from this patient. In addition, on a tree constructed with all the sequences from the eight patients, 11D is on an independent branch, while all other sequences of each patient cluster together (Fig. 2).

### Genotypic diversity of the replication-competent reservoir.

To determine the diversity of the reservoir, we first calculated the mean pairwise genetic distance (MPD) between all the sequences intrapatient. This measure, which is based on full-length-sequence differences, is commonly used, as it allows a simple estimation of the diversity of a population (79–82).

The MPD was determined based on the percentage of identity between the sequences rather than by computing the patristic distances, because of the relatively small number of sequences. Indeed, for trees with a low number of sequences, the introduction of one additional sequence could change the topology of the tree and thus modify the patristic distances. Using the number of identical nucleotides is a better raw indicator of diversity in this situation. Clonal sequences were counted only once in the analysis of diversity. Counting the same sequence multiple times would reduce the MPD score and underestimate the diversity of the reservoir of a patient. Since clonal populations wax and wane over time (28), their relative frequency at one time point is irrelevant for our purpose. Nevertheless, clonal sequences should not be ignored, as they contribute to the replication-competent reservoir and may play an important role in the viral rebound (39, 41).

We first measured the diversity of the overall genomes. The score, based on the percentage of homology between unique sequences, varied from the lowest value of 0.008 (patient X) to 0.052 (patient AB), a >6-fold difference (Table 1). The high diversity within the reservoir of patient AB is in part due to the putative double infection. Indeed, sequence 11D shows more than a 12% difference with the other sequences from this patient. If we exclude this sequence, the MPD of patient AB decreases to 0.035, which is still the highest diversity score among these patients. We verified that the low number of sequences did not introduce a bias in our study, by analyzing whether there was a correlation between the MPD score and the number of sequences. No correlation was found (*r* = 0.216, *P* = 0.637).

### Analyses of the diversity of individual genes.

**FIG 3:**
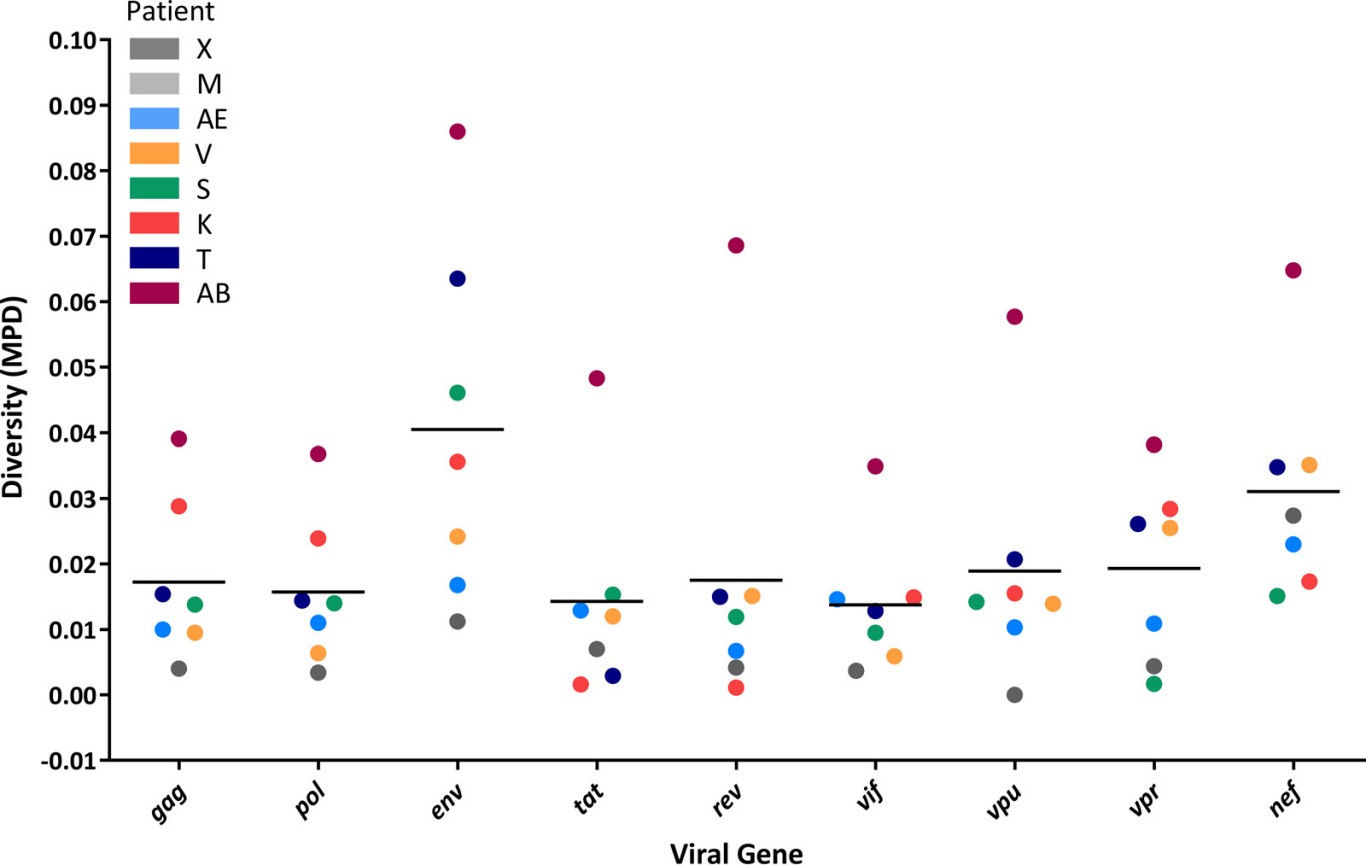
Genetic diversity for individual viral genes. The genetic diversity of each viral gene, expressed by the MPD, is represented by patient-specific colored dots. The mean interpatient values are represented by black bars. The broader range of diversity was observed for the *env* gene, followed by *nef*, whereas *tat*, *rev*, and accessory genes are relatively conserved.

Near-full-length sequencing allowed us to compare the diversity among viral genes for reservoir viruses (Table 2 and Fig. 3). In 4 patients (AB, K, S, and T), the highest genetic diversity was observed in *env*, whereas for patients X, AE, and V, *nef* was the most variable gene (Table 2). This observation is in agreement with a previous description of these two genes as carrying the highest diversity for actively replicating viruses (60) and with the continuous seeding of the reservoir (66). Interestingly, some viral genomes with identical *env* and *nef* genes could still display mutations elsewhere on the genome. On the other hand, the lowest diversity was found in different genes (*rev*, *tat*, *vif*, *vpr*, and *vpu*) for different patients (Table 2). In addition to the MPD, for each gene we also calculated the maximal pairwise distance (Table 2) between the two most diverse isolates observed in a patient, which was in general 2- to 3-fold higher than the MPD.

**TABLE 2:** MPD and the maximal pairwise distance for each viral gene

Patient	MPD (maximal pairwise distance) for^*a*^:								
	*gag*	*pol*	*env*	*tat*	*rev*	*vif*	*vpu*	*vpr*	*nef*
M	0.0000 (0.0000)	NA (NA)	0.0366 (0.0573)	NA (NA)	NA (NA)	NA (NA)	NA (NA)	NA (NA)	0.0442 (0.1004)
X	0.0040 (0.0072)	0.0034 (0.0063)	0.0112 (0.0202)	0.0070 (0.0115)	0.0042 (0.0108)	0.0037 (0.0086	0.0000 (0.0000)	0.0044 (0.0069)	**0.0274** (0.0613)
AE	0.0100 (0.0147)	0.0110 (0.0193)	0.0168 (0.0372)	0.0129 (0.0230)	0.0067 (0.0114)	0.0146 (0.0225)	0.0103 (0.0325)	0.0109 (0.0243)	**0.0230** (0.0476)
V	0.0095 (0.0100)	0.0064 (0.0077)	0.0242 (0.0289)	0.0120 (0.0153)	0.0151 (0.0199)	0.0059 (0.0069)	0.0139 (0.0163)	0.0255 (0.0275)	**0.0351** (0.0496)
S	0.0138 (0.0177)	0.0140 (0.0162)	**0.0461** (0.0594)	0.0153 (0.0230)	0.0119 (0.0171)	0.0095 (0.0121)	0.0142 (0.0244)	0.0017 (0.0035)	0.0151 (0.0250)
K	0.0288 (0.0484)	0.0239 (0.0453)	**0.0356** (0.0876)	0.0016 (0.0055)	0.0011 (0.0054)	0.0149 (0.0277)	0.0155 (0.0443)	0.0284 (0.1075)	0.0173 (0.0276)
T	0.0154 (0.0172)	0.0144 (0.0165)	**0.0635** (0.0747)	0.0029 (0.0038)	0.0150 (0.0199)	0.0128 (0.0225)	0.0207 (0.0285)	0.0261 (0.0378)	0.0348 (0.0460)
AB	0.0391 (0.1054)	0.0368 (0.0791)	**0.0860** (0.1752)	0.0483 (0.1341)	0.0686 (0.1624)	0.0349 (0.1092)	0.0577 (0.1687)	0.0382 (0.1054)	0.0648 (0.1706)

a MPD values for the most and least diverse gene are in boldface and underlined, respectively. NA, not available.

### Tropism of the viruses that compose the replication-competent reservoir.

A specific aspect of viral diversity concerns the cellular tropism. In the vast majority of patient, the virus population present at the beginning of the infection uses the chemokine receptor CCR5 in addition to CD4 to enter target cells (R5 viruses). During an untreated infection, virus evolution leads in approximately half of the patients to an expansion of virus tropism. The emergence of viruses able to use CXCR4 either exclusively (X4 viruses) or in addition to CCR5 (R5X4 dual-tropic viruses) correlates with disease progression (83). This evolution process is blocked when successful treatment is initiated. The presence of viruses with CXCR4 tropism within the reservoir (84) is an additional indicator of diversity of the virus population and may be a parameter to consider in strategies aiming to reduce the reservoir. We thus used the Geno2Pheno algorithm to infer the phenotype of the viral isolates based on their *env* gene sequences. This tool provides a score of the likelihood for a virus to be able to use CXCR4, but it cannot distinguish X4 viruses from R5X4. All patients were infected with subtype B HIV except patient X, who carried a subtype AG virus (REGA v3.4). We observed only R5 viruses in patients M, X, AE, and S, both R5- and CXCR4-tropic viruses in patients V, T, and AB, and only CXCR4-tropic viruses (which could be X4 or dual-tropic) in patient K (Table 1). For patient AB, the isolate 11D, from a putative double infection, had an R5 exclusive tropism, while 6 isolates were CXCR4-tropic, including the two pairs of identical sequences in the lower part of the phylogenetic tree shown in Fig. 1. In agreement with expectations, our data revealed a trend of higher frequency of CRXC4-tropic viruses in patients that received a delayed treatment (*P* = 0.08).

Altogether, these observations allow us to define the diversity of the replication-competent reservoir of the eight patients. The diversity varies from one individual to another, with a continuum of the MPD between 0.008 and 0.052. This diversity is distributed throughout the genome but is mainly concentrated in the *env* and *nef* genes. There is also a trend to find CXCR4-using viruses among the reservoirs of greater diversity.

### The diversity of the replication-competent reservoir correlates with the delay before reaching undetectable viremia.

We then asked whether the differences in MPD observed among the replication-competent reservoirs of patients were associated with their treatment histories. The two main variables considered are the time before controlled viremia and the duration of effective treatment. Delay in effective treatment initiation is associated with a greater diversity of the quasispecies of the circulating virus and could then impact the diversity of the replication-competent reservoir. Once viremia becomes indetectable by standard assays, the reservoir is known to remain stable in terms of quantity, but it is unknown if the diversity of the archived genomes changes during effective treatment, particularly in the replication-competent fraction of the reservoir.

**FIG 4:**
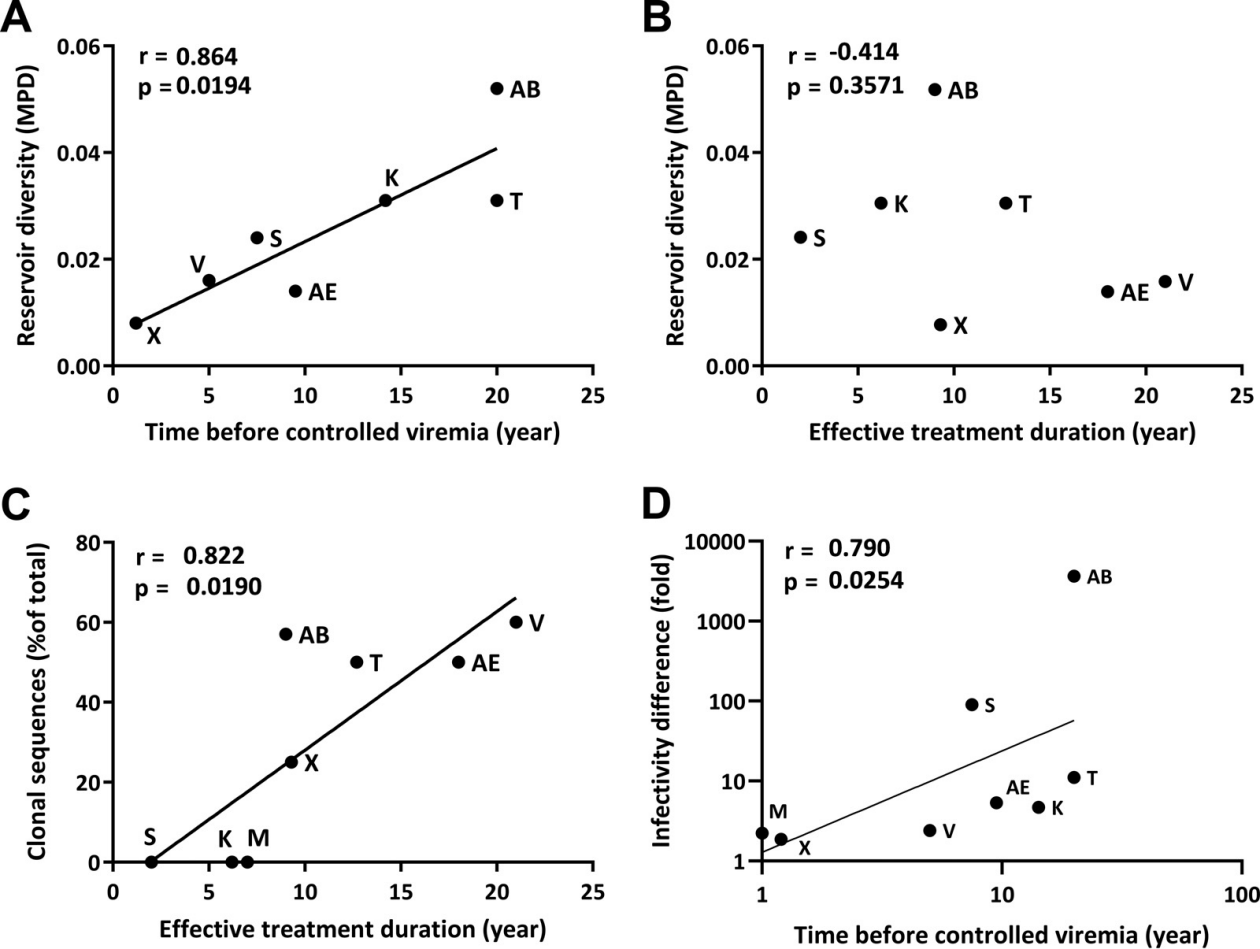
Correlation between the genetic diversity of the replication-competent reservoir and specific features of treatment histories. (A) Correlation between the diversity of the replication-competent reservoir (expressed by the MPD) and the delay before controlled viremia (*P* = 0.0194, *r* = 0.864). (B) No correlation was observed between the diversity of the replication-competent reservoir and the duration of a suppressive treatment (*P* = 0.3571, *r* = −0.414). (C) Correlation between the frequency of clonal sequences within the replication-competent reservoir and the duration of a suppressive treatment (*P* = 0.0190, *r* = 0.822). (D) Correlation between the difference of infectivity for isolates from each patient and the time before reaching controlled viremia (*P* = 0.0254, *r* = 0.790). Spearman test.

A significant correlation was observed between the MPD and the time before viremia control (*r* = 0.864, *P* = 0.0194) (Fig. 4A). A greater diversity was observed in the replication-competent reservoir of patients who received a late treatment leading to delayed viremia control than in the others, in agreement with the diversification of the virus population. In contrast, no correlation was observed between MPD and effective treatment duration (*r* = −0.414, *P* = 0.3571) (Fig. 4B). This result indicates that the diversity of the replication-competent reservoir remains stable during prolonged ART. Overall, the diversity of the replication-competent reservoir reflects the quasi-species present at the time of effective treatment initiation.

As previously mentioned, some patients harbor identical sequences in their replication-competent reservoirs, which very likely result from clonal expansion of infected cells. We observed a significant correlation between the percentage of identical viral sequences and the duration of effective treatment (*r* = 0.8225, *P* = 0.019), indicating that clonal expansion events are more frequent in patient receiving long term treatment (Fig. 4C). This is consistent with previous observations, showing higher frequency of clones in treated patients than in untreated controllers (45) and its increase with the duration of treatment (31). As mentioned above, identical sequences were excluded from the measure of MPD, so we tested if their inclusion would modify the observed correlation. Including all available sequences, we confirmed the absence of correlation between diversity and effective treatment duration (*r* = −0.464, *P* = 0.3024), while the correlation with the time before controlled viremia held true (*r* = 0.83, *P* = 0.030) (data not shown).

### Phenotypic analyses of the replication-competent reservoir.

We measured the diversity in the reservoir of patients based on genome-wide analysis and genotypic characterization. We next evaluated the phenotypic repercussions of the observed diversity for virus infectivity. It is important to characterize the phenotype of viruses present in the reservoir, as they are considered responsible for the viral rebound upon treatment cessation. To do so, we measured the infectivity in a single-cycle assay using reporter TZM-bl cells, which are susceptible to both R5 and X4 viruses. Cells were infected with a p24-normalized virus input, and the efficiency of infection was measured based on the long terminal repeat (LTR)-driven expression of β-galactosidase, using a chemiluminescent assay. Using p24-normalized virus inputs facilitates the comparison of infectivity among biological isolates that were obtained at different times during our study.

**FIG 5:**
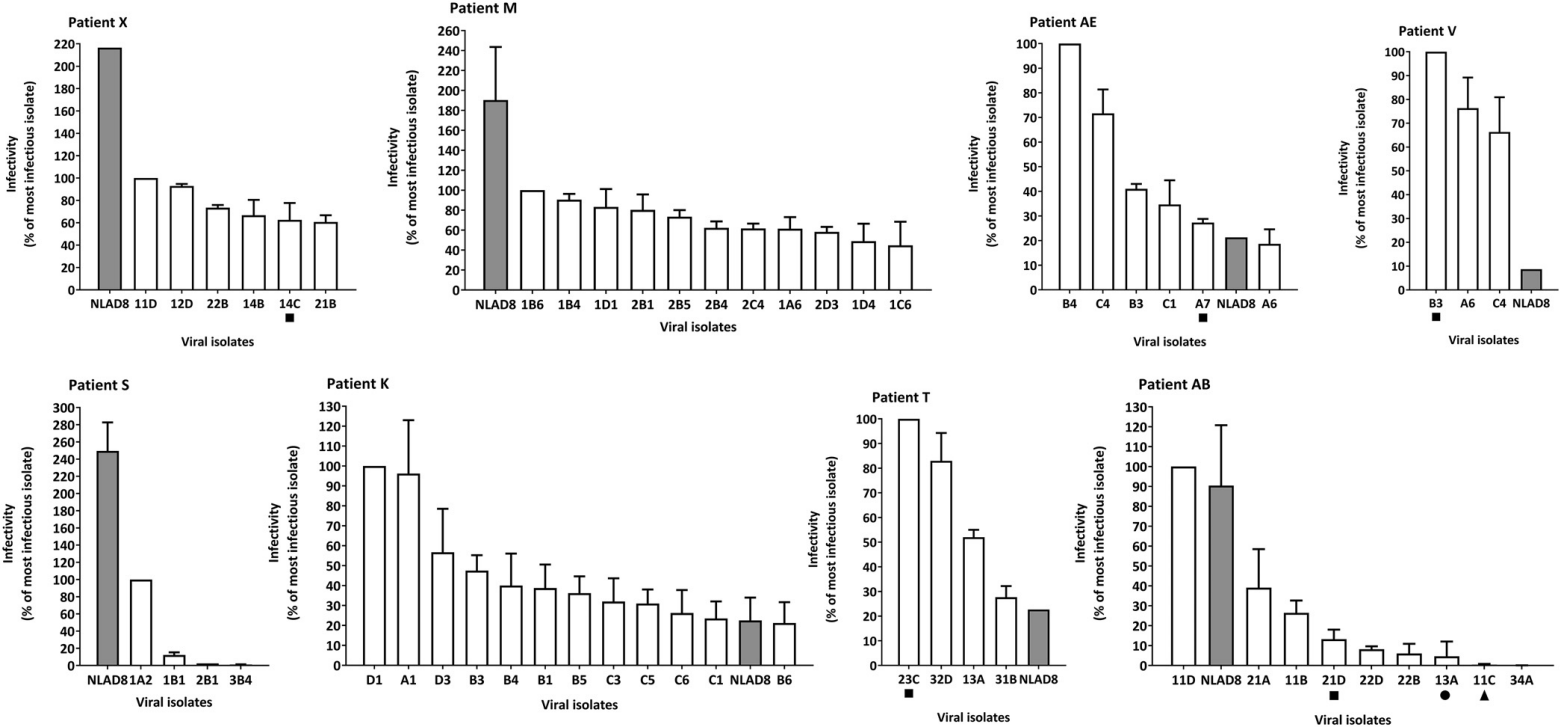
Single-cycle infectivity of the replication-competent isolates from the reservoirs of patients. The infectivity of each viral isolate was assessed by a single-cycle infection assay in TZM-bl cells. These cells express CD4 and both viral coreceptors, as well as the β-galactosidase reporter gene under the control of HIV LTR. The cells were exposed to serial dilutions of viral isolates for 24 h and then lysed, and the production of β-galactosidase was estimated by a chemiluminescent assay (Roche). Infectivity was measured as a function of p24 input and expressed as the percentage of the value for the most infectious isolate for each patient. The means and standard deviations from at least three independent experiments are shown. Geometric symbols indicate isolates for which identical sequences were identified in the reservoir. The reference strain NL-AD8 (gray bars) was used as a control.

We observed different levels of infectivity for isolates from all patients (Fig. 5). To compare the range of infectivity for the isolates of each patient, we calculated the difference between the most and least infectious isolate. We then measured the correlation between infectivity and time before controlled viremia (Fig. 4D). The positive correlation we found (*r* = 0.7904, *P* = 0.0254) shows that a broader range of infectivity was associated with delayed treatment, as was observed for the genotypic diversity assessed by the MPD.

The phenotypic diversity generally reflected that of the genotype. Interestingly, however, some virus isolates separated by great genetic distance could still display similar infectivities, and viruses on the same branch or from close clusters could reveal distinct phenotypes. A good example of this is that viruses B4 and B1 from patient K are distinct from each other on the tree, each of them representing a branch, and yet they show the same infectivity. On the other hand, A1 and C1 are close, but A1 is 4 times more infectious. Even if some sequences are not genetically far from each other, a few mutations can be responsible for relevant phenotypic differences. Finally, CXCR4-tropic viruses, when coexisting with R5-tropic viruses, could be either the most infectious (patient AE) or the least infectious (patient AB) variants.

Although the aim of our analysis was to measure the diversity of infectivity within the virus population of each patient, the use of a reference strain as control allows the additional indirect comparison of the virus populations among patients. In some patients (AE, K, T, and V), most virus isolates showed higher infectivity than the reference strain NL-AD8, while for other patients (M, S, X, and AB), the reservoir consisted mostly of viruses with lower infectivity than the reference strain. The viruses isolated from the reservoir of the patient AB were less infectious than NL-AD8, with the exception of the virus 11D, strengthening the hypothesis of a dual infection for this patient. An additional feature emerging from this analysis is that viruses characterized by high infectivity can persist in the reservoir of patients despite 2 decades of effective therapy, as illustrated by patients AE and V, who were successfully treated for 18 and 21 years, respectively.

## DISCUSSION

The quantification of the viral reservoir persisting in treated patients has been extensively explored. Its measure assists the interpretation of the kinetics of virus rebound upon treatment interruption and makes it possible to gauge the efficacy of interventions aiming at the reduction of the reservoir (85). Much less attention has been devoted to the diversity of the genomes that compose the reservoir. This qualitative assessment, however, yields information on the process of seeding of the reservoir (66) and may help to design strategies to reduce the reservoir size. Indeed, targeting a homogeneous virus population with immunotherapeutic approaches (e.g., using antibodies) could be less complicated than facing a population in which sequence variability would further complicate the task (71).

To explore this qualitative dimension of the reservoir, we chose to focus on the replication-competent viral genomes, which can be selectively explored using a VOA. Independent viruses were isolated from a relatively small volume of blood (40 mL), and their full genomes were sequenced. The use of a small blood sample facilitated the ethical approval of the protocol and the recruitment of patients but impacted the number of isolates that could be obtained. However, sequencing the full genome partly compensated for that limitation, and the overall approach allowed us to distinguish different compositions of the reservoirs in the eight studied patients. Our study highlights the complexity and heterogeneity of the replication-competent reservoir that persists despite years of effective treatments.

The topology of phylogenetic trees, based on the full viral genome, provides a snapshot of viral diversity in each individual. A relevant feature that emerged from the analysis of the trees is the presence of identical sequences in the reservoir of 5 of 8 patients, indicating clonal expansion events of cells carrying intact and competent genomes. For the three patients displaying only unique sequences, we have not excluded the possibility that the use of larger blood samples may disclose the presence of clonal expansion. When present, clonal sequences represented approximately half of the virus isolates, confirming their importance for the maintenance of the reservoir (86). The relative frequencies of clones are consistent with previous reports (25, 27, 28, 39, 45, 52, 87–89). In one of the patients (patient AB), clones belonging to three different lineages were detected and accounted for 8 of the 14 isolates from this patient. We also observed a correlation between the frequency of clonal expansion events and the duration of treatment, indicating that clonal expansion events tend to accumulate over time in patients. A progressive increase is the proportion of clonal sequence was previously reported by studying integration sites of viral genomes at different time points from 3 patients (45). However, all the genomes identified in that study were defective. An increase of clonally expanded cells carrying intact genomes was recently described also in one of two longitudinally followed patients (31). Moreover, by monitoring 8 treated individuals, Halvas et al. (90) showed that long lasting expanded clonal populations may be the cause of persistent nonsuppressible viremia, with some of the variants being replication-competent. Also, by sequencing viral *env* genes from the supernatant of qVOA cultures, Lorenzi et al. (68) observed that clonal expanded viral sequences represented more than half of the replication-competent reservoir in patients. Our results support the hypothesis that the replication-competent reservoir is in large part maintained by clonal expansion (86).

The main aim of our study was to explore the diversity of the virus populations, which we accomplished by measuring the MPD, by computing the distance between all pairs of sequences. Since the frequencies of clones were shown to fluctuate over time (28), and because the presence of clonal sequences underestimates the MPD, we calculated the MPD counting each series of clones as a single sequence. By comparing the MPD to the treatment history of the patients, we found a significant correlation between the delay before reaching undetectable viremia and the extent of genetic diversity of the virus population. Importantly, the correlation also held true when all clonal sequences were computed. In the absence of treatment, the increase in diversity as a function of the time was largely documented (60, 91, 92) and was also observed at the level of integrated DNA (93). As a consequence, early effective treatment initiation is associated with lower DNA reservoir size and diversity, as reviewed by Simonetti and Kearney (94). Given that the vast majority of proviral DNA is defective; however, the diversity of the replication-competent component deserved to be directly addressed (71). Our conclusion, based on the analysis of the full-length genome of VOA isolates, is consistent with a recent report showing a higher genetic complexity in *gag* and *nef* genes in the replication-competent reservoir of patients who were perinatally infected and treated after a mean of 22 years, compared to patients infected as adolescent and treated after a mean of 6 years (70).

In addition to the delay in viremia control, we tested whether the duration of treatment had an impact on the diversity of the replication-competent reservoir but found no correlation. Thus, the diversity of the replication-competent reservoir appears to be stable, similar to what was previously shown for the global proviral DNA population (61, 63–65). This result appears to be at odds with recent reports demonstrating a more rapid decline of the replication-competent fraction of the reservoir under ART, compared to defective genomes (31, 49, 51). A faster elimination of cells carrying intact proviruses might result in a reduction of the diversity over time. However, as shown in the report by Pinzone et al., the decline of the infected cell was obscured by clonal expansion events (31). Elimination of clonally expanded cells carrying replication-competent viral genomes would affect the number of sequences but not necessarily the diversity. In the context of a continuous expansion and contraction of clonal populations over time (28), the higher but still limited decay of intact sequences might not exert a measurable impact on the diversity of the population. Of note, differences in the frequency of clonal sequences were recently demonstrated between proviral DNA sequences, qVOA isolates and viruses that reactivated *in vivo* after short treatment interruptions (69).

Together, these results are important to consider in order to develop strategies aiming at the reduction or the elimination of the reservoir. The initiation of an early treatment limits the size of the reservoir, which is associated with a viral rebound delay (71, 95) and appears to be a determining factor in the establishment of a posttreatment control (10, 96–98). Here, we show that it also limits the diversity of the replication-competent reservoir, a subpopulation that is relevant for the design of immunotherapeutic approaches. Indeed, recent studies have compared the genetic composition of the intact proviruses to the genome of the virus that emerged during analytical treatment interruptions in the presence of broadly neutralizing antibodies (40, 42). While a direct proviral counterpart of the rebounding virus could not be identified, the emerging virus appears to be the result of recombination events that allow to overcome the complex selective pressure of the environment, including the neutralizing antibodies. Recombination can only occur when one cell is infected by two viruses (99), highlighting the importance of the replication-competent and inducible fraction of the reservoir. In this context, our documentation of highly infectious variants even after decades of effective treatment is of particular concern.

## MATERIALS AND METHODS

### Study participants.

Blood samples were obtained from 8 HIV-1-infected individuals, for whom a regular longitudinal follow-up demonstrating the effectiveness of treatment by undetectable viral load was available. The study includes only participants who initiated treatment during chronic phase of infection. At the time of sampling, participants had been on effective ART for at least 2 years, and blood was collected within a month from a negative viremia test (20 copies/mL). Informed consent was obtained from all the patients, according to the French national regulation.

### Resting CD4^+^ T cell isolation.

Peripheral blood samples of 40 mL were collected and diluted 1 to 2 in phosphate-buffered saline (PBS) containing 2% fetal bovine serum (FBS). Peripheral blood mononuclear cells (PBMCs) were isolated by centrifugation using Sepmate tubes (StemCell Technologies) prefilled with 12 mL of Ficoll. Isolated PBMCs were then centrifuged for 10 min at 1,200 × *g* at room temperature, and the cells were resuspended at 5 × 10^7^ cells/mL in PBS with 2% FBS and 1 mM EDTA. Resting CD4^+^ T cells were then sorted by negative purification using a modified EasySep human CD4^+^ T cell kit (StemCell Technologies) with the addition of antibodies against CD25, CD69, and HLADR, in order to remove activated cells. Isolation was performed according to the manufacturer’s protocol.

### Quantitative viral outgrowth assay.

Resting CD4^+^ T cells (CD69^−^ CD25^−^ HLADR^−^) were immediately plated at two different concentrations (200,000 cells or 40,000 cells per well) in 200 μL of Roswell Park Memorial Institute medium (RPMI) in round bottom 96-well plates and activated using anti-CD3/CD28 Dynabeads (Thermo Fisher Scientific) at a ratio of 1 bead to 2 cells. The next day, the cells were transferred in individual wells of 24-well plates and cocultivated with 10^5^ PHA-activated (1 μg/mL) CD4^+^ T cells from a healthy donor in RPMI with 100 U/mL of interleukin 2 (IL-2) to allow viral outgrowth. Viral emergence was monitored each week for 3 weeks by quantification of p24 protein using an ELISA (Ingen) in the culture supernatants. Each positive well was isolated, and the supernatant was eliminated and replaced by fresh RPMI with IL-2. Virus-containing supernatant was collected 48 h later and immediately frozen at −80°C. For p24-negative wells after 1 week of culture, 10^5^ new donor cells were added and the wells were tested again 1 week later.

### Next-generation sequencing.

Viral isolates were incubated at 37°C during 30 min with a mix of Turbo DNase (Thermo Fisher Scientific), Benzonase (Merck), Baseline-Zero DNase (Frilabo), and RNase A (Roche) to eliminate all human genomic contamination while the viral genome was protected in the particle. Enzymes were inactivated and viral particle lysed. Viral RNA was extracted using a QIAamp viral RNA kit (Qiagen), reverse transcribed and amplified using the SuperScriptIV one-step RT-PCR (Thermo Fisher Scientific) and set of overlapping primers as described by Ode et al. (78). Viral cDNA was purified using the NucleoSpin PCR/gel cleanup kit (Macherey-Nagel) and quantified with a Qubit fluorometer (Invitrogen). Libraries were then produced using the Nextera XT kit (Illumina). Following the Illumina protocol, libraries were produced by individual tags for each patient. Genomes were amplified on the MiSeq platform, and contigs were generated by *de novo* assembly with a previously described protocol (78).

### Data analysis.

Phylogenetic analyses were conducted using Geneious (R8) software. Reconstructed sequences from each patient were annotated manually and aligned with NL4.3 using MAFFT (100) (INS-i algorithm, scoring matrix = 200 PAM [point accepted mutation]/*k* = 2, gap open penalty = 1.53, offset value = 0.123) to make sure that the sequences were complete. Alignments were manually verified, and sequences were corrected; in particular, gaps due to missing information were filled with “?” to avoid mismatch counts and ignore these positions in the further analysis. A second verification of the sequences was performed, and each gene was extracted and verified by translation to amino acids to verify open reading frames. After these verifications, full-length genomes were aligned as well as individual gene sequences. Phylogenetic trees were constructed using a maximum-likelihood approach. We used PHYML (101) parameterized with a Tamura-Nei substitution model and 1,000 bootstraps. Trees were produced for full-length genomes as well as for each individual gene. The overall diversity for each tree is determined by the mean pairwise genetic distance (MPD) between all the sequences from a same tree. MPD is one of the most common sequence-based statistic to estimate the diversity of HIV populations within individuals (59, 61, 62, 81, 93, 102, 103). The MPD is calculated based on the percentage of identity matrix:

$$mn\left(X\right)=\frac{\sum \left\{1-\frac{Xp}{100}\right\}}{nXp}$$

where *mn*(*X*) is the mean of the genetic distance between all the possible pairs of sequences, *Xp* is the percentage of identity between sequences in a pair, and *nXp* is the number of pairs. This results in a score representative of the average genetic diversity of an HIV population within an individual. Correlations were analyzed using the nonparametric Spearman test, on Prism 7 (GraphPad).

### Single cycle infection.

TZM-bl cells were cultured in 96-well flat-bottom plates at 15,000 cells per well in 100 μL of Dulbecco's modified Eagle medium (DMEM). The cells were infected with limiting dilutions of viral samples from 1 ng to 0.06 ng, in the presence of DEAE dextran (final concentration, 30 μg/mL). At 24 h postinfection, a chemiluminescent assay was performed using a β-Gal reporter gene assay (Roche). The signal was measured on a VariosKan (Thermo Fisher Scientific). Infectivity was measured as β-Gal activity per ng of p24 and expressed as a percentage of the most infectious virus for each patient.

### Data availability.

The nucleotide sequences obtained and described here are available as supplemental file 1.
