# Supplementary material for: Genotypic and Phenotypic Diversity of the Replication-Competent HIV Reservoir in Treated Patients
Source: Microbiol Spectr. 2022 Jun 30;10(4):e00784-22. doi: 10.1128/spectrum.00784-22 (PMC9431663; doi:10.1128/spectrum.00784-22)
Supplement: Supplemental file 1 — Near full-length sequences of HIV-1 variants isolated from the replication competent reservoir of patients. Download spectrum.00784-22-s0001.pdf, PDF file, 0.4 MB [file spectrum.00784-22-s0001.pdf]

>AB-11A HIV-1 genome, derived from RNA genomic sequence
ACGCAGGACTCGGCTTGCTGAAGCGCGCACGGCAAGAGGCGAGGGGCGGCGACTGGTGAGTACGCCAAATTTT
GACTAGCGGAGGCTAGAAGGAGAGAGATGGGTGCGAGAGCGTCAGTATTAAGTGCGGGGGAATTAGATACGTG
GGAAAAAATTCGGTTAAGGCCAGGGGGAAAGAAAAAATATAGATTAAAACATATAGTATGGGCAAGCAGGGAG
CTAGAACGATTTGCAGTTAATCCTGGCCTGTTAGAAACATCAGCAGGCTGTAGACAAATACTGGGACAGCTAC
ATCCATCCCTTCAGACAGGATCAGAAGAACTTAGATCATTATATAATACAGTAGCAACCCTCTATTGTGTGCA
TCAAAAGATAGAGGTAAAAGACACCAAGGAAGCTTTAGAGAAGATAGAGGAAGAACAAAACAAAAGTAAGAAA
AAGGCACAACAAGCAGCAGCTGACACAGGAAACAGCAGCCAGGTCAGCCAAAATTACCCCATAGTACAGAACC
TCCAGGGGCAAATGGTACATCAGACCATGTCACCTAGAACTTTAAATGCATGGGTAAAAGTAATAGAAGAGAA
GGCTTTCAGCCCAGAAGTAATACCCATGTTTTCAGCATTATCAGAAGGAGCCACCCCACAAGATTTAAACACC
ATGCTAAACACAGTGGGGGGACATCAAGCAGCCATGCAAATGTTAAAAGATACCATCAATGAGGAAGCTGCGG
AATGGGATAGATTGCATCCAGTGCATGCAGGGCCTATTGCACCAGGCCAGATGAGAGAACCAAGGGGAAGTGA
CATAGCAGGAACTACTAGTACCCTTCAGGAACAAATAGGATGGATGACACATAATCCACCTATCCCAGTAGGA
GAAATTTATAAGAGATGGATAATCCTGGGATTAAATAAAATAGTAAGAATGTATAGCCCTACCAGCATTCTGG
ACATAAAACAAGGACCAAAAGAACCCTTTAGAGATTATGTAGACCGGTTCTATAAAACTCTAAGAGCCGAGCA
AGCTTCACAGGATGTAAAAAATTGGATGACAGAAACCTTGTTGGTCCAAAATGCGAATCCAGATTGTAAGACT
ATTTTAAAAGCATTGGGACCAGCAGCTACATTAGAAGAAATGATGACAGCATGTCAGGGAGTGGGGGGACCCA
GCCATAAAGCAAGAGTCTTGGCTGAAGCAATGAGCCAAGCAACAGGTTCAGCTACCATAATGATGCAGAGAGG
CAATTTTAGGAACCAAAGAAAGACTGTTAAGTGTTTCAATTGTGGCAAAGAGGGGCACATAGCCAGAAATTGC
AGGGCCCCTAGGAAAAAGGGCTGTTGGAAATGTGGAAAGGAAGGACACCAAATGAAGGATTGCACTGAGAGAC
AGGCTAATTTTTTAGGGAAGATCTGGCCTTCCCACAAGGGAAGGCCAGGGAATTTCCTTCAGAGCAGACCAGA
GCCAACAGCCCCACCAGAAGAGAGCTTCAGGTTTGGGGAAGCAACAACTCCCTCTCAGAAGCAGGAGACGATA
GACAAGGAACTGTATCCTTTAACCTCCCTCAAATCACTCTTTGGCAACGACCCCTTGTCACAGTAAGGATAGG
GGGGCAACTAAAAGAAGCTCTATTAGATACAGGAGCAGATGATACAGTATTAGAAGAAATGAATTTGCCAGGA
AGATGGAAACCAAAAATGATAGGGGGAATTGGAGGTTTTATCAAAGTAAGACAGTATGATCAGATACTCATAG
AAATCTGTGGACATAAAGCTATAGGTACAGTATTAGTAGGACCTACACCTGTCAACATAATTGGAAGGAATCT
GTTGACTCAGATTGGTTGCACTTTAAATTTTCCCATTAGTCCTATTGAAACTGTACCAGTAAAACTAAAGCCA
GGAATGGATGGCCCAAAAGTTAAACAATGGCCATTGACAGAAGAAAAAATAAAAGCATTAGTAGAAATTTGTA
CAGAAATGGAAAAGGAAGGAAAATTTCAAAAATTGGGCCTGAAAATCCATACAATACTCCAGTATTTGCCAT
AAAGAAAAAAGACAGTACTAAATGGAGAAAATTGGTAGATTTCAGAGAACTTAATAAGAGAACTCAAGACTTC
TGGGAAGTTCAATTAGGAATACCACATCCTGCAGGGTTAAAAAAGAAAAAATCAGTAACAGTCCTGGATGTGG
GTGATGCATATTTTTCAGTCCCCTTAGATGAAAACTTTAGAAAGTATACTGCATTTACCATACCTAGTACAAA
CAATGAGACACCAGGGACTAGATATCAGTACAATGTGCTGCCACAGGGATGGAAAGGATCACCAGCAATATTC
CAAAGTAGCATGACAAAAATCTTAGAGCCTTTTAGAAAACAAAATCCAGACATAGTTATCTATCAATACATGG
ATGATTTGTATGTAGGATCCGACTTAGAAATAGGGCAGCATAGAATAAAAATAGAGGAACTGAGAGAACATCT
GTTGAGGTGGGGATTTACCACACCAGACAAAAAACATCAAAAAGAACCTCCATTCCTTTGGATGGGTTATGAA
CTCCATCCTGATAAATGGACAGTACAGCCTATAGTGCTGCCAGAGAAAGACAGCTGGACTGTCAATGACATAC
AGAAGTTAGTGGGAAAATTGAATTGGGCAAGTCAGATTTATCCAGGGATTAAAGTAAAGCAATTATGTAAGCT
CCTTAGGGGAACCAAAGCACTAACAGAAGTAATACCACTAACAGAAGAAGCAGAGCCAGAACTAGCAGAAAAC
AGGGAGATTCTAAAAGAACCAGTACATGGAGTGTATTATGACCCATCAAAAGACTTAGTAGCAGAAATACAGA
AGCAGGGGCAAGGCCAATGGACATATCAAATTTATCAAGAGCCATGTAAAAATCTGAAAACAGGAAAGTATGC
AAGAATGAGGGGTGCCCACACTAATGATATAAAACAGTTAACAGAGGCAGTGCAAAATATAGCCACAGAAGGC
ATAATAATATGGGGAAAGACTCCTAAATTTAGACTACCCATACAAAAGGAAACATGGGAAGCATGGTGGATGG
AGTATTGGCAAGCCACCT?GATTCCTGAATGGGAATTTGTCAATACCCCTCCCTTAGTAAAATTATGGTATCA
GTTAGAGAAAGAACCCATAGAGGGAGCAGAAACCTTCTATGTAGATGGGGCAGCTAATAGGGAGACTAAATTA
GGAAAAGCAGGATATGTTACTGACAGAGGAAGACAAAAAGTTGTCTCCCTAACTGACACAACAAATCAGAAGA
CTGAGTTACAAGCAATTCATCTAGCGTTGCAGGATTCGGGACTAGAAGTAAACATAGTGACAGACTCACAATA
TGCATTAGGAATCATTCAAGCACAACCAGATAAAAGTGAATCAGAGTTAGTCAGTCAAATAATAGAGCAGTTA
ATAAAAAAGGAAAAAGTCTACCTGGCATGGGTACCAGCACACAAAGGAATTGGAGGAAATGAACAAGTAGATA
AATTAGTCAGTACTGGAATCAGGAGAGTACTATTTTTAGATGGAATAGATAAGGCCCAAGAAGAACATGAGAA
ATATCACAGTAATTGGAGAGCAATGGCTAGTGATTTTAACCTGCCACCTGTAATAGCAAAAGAGATAGTAGCC
TGCTGTGATAAATGTCAGCTAAAAGGAGAAGCCATGCATGGACAAGTAGACTGTAGTCCAGGAATATGGCAAC
TAGACTGTACACATTTAGAAGGAAAAATTGTCCTGGTAGCAGTTCATGTAGCCAGTGGATATATAGAAGCAGA
AGTCATTCCAGCAGAGACAGGGCAGGAAACAGCATACTTTCTCTTAAAATTAGCAGGAAGATGGCCAGTAAAA

ACAATACATACAGACAATGGCAGCAATTTCACCAGTAATGTGGTTAAGGCTGCCTGTTGGTGGGCAGGGATCA
AGCAGGAATTTGGCATTCCCTACAATCCCCAAAGTCAAGGAGTAGTAGAATCTATGAATAAAGAATTAAAGAA
AATTATAGGACAGGTAAGAGATCAGGCTGAACATCTTAGGACAGCAGTACAAATGGCAGTATTCATCCACAAT
TTTAAGAGAAAAGGGGGGATTGGGGGGTACAGTGCAGGGGAAAGAATAGTAGACATGATAGCAACAGACATAC
AAACTAAAGAATTACAAAAACAAATTACAAAAATTCAAAATTTTCGGGTTTATTACAGGGACAGCAGAGATCC
ACTTTGGAAAGGACCAGCAAAGCTTCTCTGGAAAGGTGAAGGGGCAGTAGTAATACAAGATAATAGTGATATA
AAAGTAGTGCCAAGAAGAAAAGCAAAGATCATTAGGGATTATGGAAAACAGATGGCAGGTGATGATTGTGTGG
CAAGTAGACAGGATGAGGATTAGAACATGGAAAAGTTTAGTAAAACACCATATGTATGTTTCACGGAAAGCTG
GGAAATGGTTTTATAGACATCACTATGAAAGCACTCATCCAAAAATAAGTTCAGAAGTACACATCCCACTAGG
GGATGCTAGATTGGTAATAACAACATATTGGGGTCTGCATACAGGAGAAAGAGACTGGCAGTTGGGCCATGGA
GTCTCCATAGAATGGAGGAAAAAGAGATATAGCACACAAGTAGACCCTGATCTAGCAGACCAACTAATCCATC
TGTATTATTTTGATTGTTTTTCAGAATCTGCTATAAGACATGCCATATTAGGACATATAGTTAGACCTAGTTG
TGAATATCAAGCAGGACATAACAAGGTAGGATCCCTACAATACTTGGCACTAACAGCACTAATAAAACCAAAG
AAGATAAAGCCACCTTTGCCTAGTGTTAAGAAACTGACAGAGGATAGATGGAACAAGCCCCAGAAGACCAAGG
GCCACAGAGGGAGCCATACAATGAATGGACGCTAGAGCTTTTAGAGGAGCTTAAGAGTGAAGCTGTTAGACAT
TTTCCTAGGGCATGGCTACATAGCTTAGGACAATATATCTATGAAACTTATGGGGATACTTGGGCAGGAGTGG
AGGCCATAATAAGAATGCTGCAACAACTGCTGTTTATTCATTTCAGAATTGGGTGTCGCCATAGCAGAATAGG
CATTATTCAACAGAGGAGAGCAAGACATGGAGCCAGTAGATCCTAGACTAGAACCCTGGAAGCATCCAGGAAG
TCAGCCTAGGACTCCTTGTACCAATTGCTATTGTAAAAAGTGTTGCCTTCATTGCCAAGTTTGTTTTATGAAA
AAAGGCTTAGGCATCTCCTATGGCAGGAAGAAGCGGAGACAGCGACGAAGACCTCCTCAAGACAGTGAGAATC
ATCAAGTTCCTCTATCAAAGCAGTAAGTAGTACATGTAATGCAACCTTTACATATAGTAGCAATAGTAGCATT
AGTAGTAGCAGCAATAATAGCAATAGTTGTGTGGACCATAGTAGGCATAGAATATAGGAAAATATTAAGACAA
AGAAAAATAGACAGGTTAATTGATAGAATAAGAGAAAGAGCAGAAGACAGTGGCAATGAAAGTGAAGGAGACC
AGGAAGAATTATCAGCACTTGTGGAGATGGGGCACCATGCTCCTTGGGATGTTGATGATCTGTAGGGCTGCAG
AACAGTTGTGGGTCACAGTCTATTATGGGGTACCTGTGTGGAAAGAAGCAAATACCACTCTATTTTGTGCATC
AGATGCTAAAGCATATGATACAGAGGTACATAATGTTTGGGCCACACATGCCTGTGTACCCACAGACCCCAAC
CCACAAGAAGTAGTATTGGAAAATGTGACAGAAGAATTTAACATGTGGAAAAATAACATGGTAGAACAGATGC
ATGAAGATATAATCAGTTTATGGGATCAAAGCCTAAAGCCATGTGTAAAATTAACCCCACTCTGTGTTACTTT
AGATTGCACTGATTTGAGGAATGCTACTAATGCCACTAATAGTAATGGGACAATAAAGGAAGAAATGAAAAAC
TGCTCTTTCAATATCACCACAAGCATAAGAGATAAGGTGCAGAAAGAATATGCACTTTTTTATAGACTTGATA
TAGTACAAATAGAGAATGATAATACTAACAATACTAACAATACTAGCTATAGGATGATAAATTGTAATACCTC
AGTCATTACACAGGCCTGTCCAAAGATATCCTTTGAGCCAATTCCCATACATTATTGTGCCCCGGCTGGTTTT
GCGATTCTAAAGTGTAACAATAAGACGTTCAATGGAAAAGGACCATGTAAAAATGTCAGCACAGTACAATGTA
CACATGGAATTAAGCCAGTAGTGTCAACTCAACTGCTGTTAAATGGCAGTCTAGCAGAAAAAGAGGTGGTAAT
TAGATCTGTCAATTTCACAGACAATGCTAAAACCATAATAGTACAGCTGAACAAATCTGTAGAAATTAATTGT
ACAAGACCCAACAACAATACAAGAAAAAGTATACATATAGGACCAGGGAGAGCATTTTATGCAACAGGAGACA
TAATAGGAGATATAAGACAAGCACATTGTAACATTAGTAGAGCAGAATGGAATAACACTTTAAGACAGATAGC
TAAAAAATTAAGAGAACAATTTGTGAATAAAACAATAGTCTTTAATCAATCCTCAGGAGGGGACCCAGAAATT
GTAATGCACAGTTTTAATTGTGGAGGGGAATTTTTCTACTGTAATTCAACACAATTGTTTAATAGTACTTGGA
ATGAAACCAACATTGACGGAAATGACACCACTAAAGGAGATAATATCACAGATGTCATCACACTCCCATGCAG
AATAAAACAAATTATAAACATGTGGCAGGAAGTAGGAAAAGCAATGTATGCCCCTCCCATCAGAGGACAAATT
AATTGTTTATCAAATATTACAGGGCTGCTATTAACAAGAGATGGTGGTAATCAGAGTGGGAACACCGAGATCT
TCAGACCTGTAGGGGGAAATATGAAGGACAATTGGAGAAGTGAATTATATAAATATAAAGTAGTACAAATTGA
ACCATTAGGAGTAGCACCCACCAAGGCAAAGAGAAGAGTGGTGCAGAGAGAAAAAAGAGCAGTGGGAACGATA
GGAGCCATGTTCCTTGGGTTCTTGGGAGCAGCAGGAAGCACTATGGGCGCAGCGTCAATGACGCTGACGGTAC
AGGCCAGACAATTATTGTCTGGTATAGTGCAACAGCAGAGCAATTTGCTGAGGGCTATTGAGGCGCAACAGCA
TATGTTGCAACTCACAGTCTGGGGCATCAAGCAGCTCCAGGCAAGAGTACTGGCTGTGGAAAGATACCTACAG
GATCAACGGCTCCTAGGGATTTGGGGTTGCTCTGGAAAACTCATCTGCACCACTGCTGTGCCTTGGAATACTA
GTTGGAGTAATAAAAATCTGACTCAGATTTGGGATAACATGACCTGGATGCAGTGGGAAAAAGAAATTAACAA
TTACACAGGAGTAATATACAACTTACTTGAAAAATCGCAGAACCAACAAGAAAAGAATGAACAAGAATTATTG
GAATTAGATGAGTGGGCAAGTTTGTGGAATTGGTTTGACATAACAAAATGGCTGTGGTATATAAAAATATTCA
TAATGATAGTAGGAGGCTTGGTAGGTTTGAGAATAGTTTTTGCTGTACTTTCTATAGTGAATAGAGTTAGGCA
GGGATACTCACCATTATCATTTCAGACCCTCCTCCCAGCCCCGAGGGGACCCGACAGGCCCGAAGGAATA?AA
GAAGAAGGTGGAGAGAGAGACAGAGGCAGATCAAATCGATTAGCAACTGGATTCTTGATACTTTTCTGGGACG

ACCTGCGGAGCCTGTGCCTCTTCAGCTACCACCGATTGAGAGACTTACTCTTGATTGTAGGGAGGATTGTGGG
AATTCTGGGACACAGGGGGTGGGAGATCCTCAAATATTGGTGGAATCTCCTGCAATATTGGAGTCAGGAACTA
AAGAATAGTGCTGTTAGCTTGCTCAATGCCACAGCTATCGCAGTAGCTGAGGGAACAGATAGGGTTATAGAAG
TAGTACGAAGAGTTTTTAGAGCTATTCTCCACATACCTACAAGAGTGAGACAGGGCTTGGAAAGGGCTTTGCT
ATAAGATGGGTGGCAAGTGGTCAAAACGTAGTCTGGGTGGATGGCCTAATGTAAGGGAAAGAATGAGAAGAAC
TGAGCCAGCAGCAGATGGGGTGGGAGCAGTATCTCGAGACCTGGAAAAACATGGGGCAATCACAAGTAGCAAT
ACAGCAACTAGTAATGCTGCCTGTGCCTGGCTAGAAGCACAAGAGGAAGAGGAGGTGGGTTTTCCAGTCAGAC
CTCAGGTACCTTTAAGACCAATGACTTACAAGGGAGCTTTAGATCTTAGCCACTTTTTAAGAGAAAAGGGGGG
ACTGGAAGGGCTAATTCACTCCCAGAAAAGACAAGAGATCCTTGATCTGTGGGTCTACCACACACAAGGCTAC
TTCCCTGATTGGCAGAACTACACACCAGGGCCAGGGATCAGATATCCCCTGACCTTTGGATGGTGCTTCAAGT
TAGTACCAGTTGAGCCAGACGAAGAAGAAAACAGCAGCTTGCTACACCCTATGAGCCAGCATGGGATGGAGGA
CACGGAGAGAGAAGTGTTAAAGTGGAAGTTTGACAGCCGCCTAGCATTTCATCACATGGCCCGAGAGCTGCAT
CCGGAGTATTACAAAGACTGCTGACACCGGGTTTTCTACAAGGGACTTTCCGCTGGGGACTTTCCAGGGAGGC
GTGGCCTGGGCGGGACTGGGGAGTGGCGAGCCCTCAGATGCTGCATATAAGCAGCTGCTTTTTGCCTGTACTG
GGTCTCTCTGGTTAGACCAGATCAGAGCCTGGGAGCTCTCTGGCTAACTAGGGAACCCACTGCTTAAGCCTCA
ATAAAGCTTGCCT

>AB-11B HIV-1 genome, derived from RNA genomic sequence
ACGCAGGACTCGGCTTGCTGAAGCGCGCACGGCAAGAGGCGAGGGGCGGCGACTGGTGAGTACGCCAAAATTT
TGACTAGCGGAGGCTAGAAGGAGAGAGATGGGTGCGAGAGCGTCAGTATTAAGTGCGGGGGAATTAGATACAT
GGGAAAAAATTCGGTTAAGGCCAGGGGGAAAGAAAAAATATAGATTAAAACATATAGTATGGGCAAGCAGGGA
GCTAGAACGATTCGCAGTTAATCCTGGCCTGTTAGAAACATCAGCAGGCTGTAGACAAATACTGGGACAGCTA
CAACCAGCCCTTCAGACAGGATCAGAGGAACTTAGATCATTATATAATACAGTAGCAACCCTCTATTGTGTGC
ATCAAAAGATAGAGGTAAAAGACACCAAGGAAGCTTTAGAGAAGATAGAGGAAGAGCAAAACAAAAGCAAGAA
AAAGGCACAGCAAGCAGCAGCTGACACAGGAAACAGCAGCCAGGTCAGCCAAAATTACCCTATAGTACAGAAC
CTCCAGGGGCAAATGGTACATCAGACCATGTCACCTAGAACTTTAAATGCATGGGTAAAAGTAATAGAAGAGA
AGGCTTTCAGCCCAGAAGTAATACCCATGTTTTCAGCATTATCAGAAGGAGCCACCCCACAAGACTTAAACAC
CATGCTAAACACAGTGGGGGGACATCAAGCAGCCATGCAAATGTTAAAAGATACCATCAATGAGGAAGCTGCA
GAATGGGATAGATTGCATCCAGTGCATGCAGGGCCTATTGCACCAGGCCAGATGAGAGAACCAAGGGGAAGTG
ACATAGCAGGAACTACTAGTACCCTTCAGGAACAAATAGGATGGATGACACATAATCCACCTATCCCAGTAGG
AGAAATTTATAAGAGATGGATAATCCTGGGATTAAATAAAATAGTAAGAATGTATAGCCCTACTAGCATTCTG
GACATAAAACAAGGACCAAAAGAACCCTTTAGAGATTATGTAGACCGGTTCTATAAAACTCTAAGAGCCGAGC
AAGCTTCACAGGATGTAAAAAATTGGATGACAGAAACCTTGTTGGTCCAAAATGCGAATCCAGATTGTAAGAC
TATTTTAAAAGCATTGGGACCAGCAGCTACATTAGAAGAAATGATGACAGCATGTCAGGGAGTGGGGGGACCC
AGCCATAAAGCAAGAGTCTTGGCCGAAGCAATGAGCCAAGCAACAGGCTCAGCTAACATAATGATGCAGAGAG
GCAATTTTAGGAACCAAAGAAAGACTGTTAAGTGTTTCAATTGTGGCAAAGAGGGGCACATAGCCAGAAATTG
CAGGGCCCCTAGGAAAAAGGGCTGTTGGAAATGTGGAAAGGAAGGACACCAAATGAAGGATTGCACTGAGAGA
CAGGCTAATTTTTTAGGGAAGATCTGGCCTTCCCACAAGGGAAGGCCAGGGAATTTTCTTCAGAGCAGACCAG
AGCCAACAGCCCCACCAGAAGAGAGCTTCAGGTTTGGGGAAGCAACAACTCCCTCTCAGAAGCAGGAGACGAT
AGACAAGGAACTGTATCCTTTAACCTCCCTCAAATCACTCTTTGGCAACGACCCCTCGTCACAATAAGGATAG
GGGGGCAACTAAAGGAAGCTCTATTAGATACAGGAGCAGATGATACAGTATTAGAAGAAATGAATTTACCAGG
AAGATGGAAACCAAAAATGATAGGGGGAATTGGAGGTTTTATCAAAGTAAGACAGTATGATCAGGTACTCATA
GAAATCTGTGGACATAAAGCTATAGGTACAGTATTAGTAGGACCTACGCCTGTCAACATAATTGGAAGAAATC
TGTTGACTCAGATTGGTTGCACTTTAAATTTCCCCATTAGTCCTATTGAAACTGTACCAGTAAAATTAAAGCC
AGGAATGGACGGCCCAAAAGTTAAACAATGGCCATTGACAGAAGAAAAAATAAAAGCATTAGTAGAAATTTGT
ACAGAAATGGAAAAGGAAGGGAAAATTTCAAAAATTGGGCCTGAAAATCCATATAATACTCCAGTATTTGCCA
TAAAGAAAAAAGACAGTACTAAATGGAGAAAATTGGTAGATTTCAGAGAACTTAATAAGAGAACTCAAGATTT
CTGGGAAGTCCAATTAGGAATACCACATCCCGCAGGGTTAAAAAAGAAAAAATCAGTAACAGTCCTGGATGTG
GGTGATGCATATTTTTCAGTTCCCCTAGATGAAAACTTTAGAAAGTATACTGCATTTACCATACCTAGTGTAA
ACAATGAGACACCGGGGACTAGATATCAGTACAATGTGCTTCCACAGGGATGGAAAGGATCACCAGCAATATT
CCAAAGTAGCATGACAAGAATCTTAGAGCCTTTTAGAAAACAAAATCCAGACATAGTTATCTATCAATACATG
GATGATTTGTATGTAGGATCCGACTTAGAAATAGGGCAGCATAGAACAAAAATAGAGGAACTGAGACAACATC
TGTTGAGGTGGGGATTTACCACACCAGACAAAAAACATCAAAAAGAACCTCCATTCCTGTGGATGGGTTATGA
ACTCCATCCTGATAAATGGACAGTACAGCCTATAGTGCTGCCAGAAAAAGACAGCTGGACTGTCAATGACATA
CAGAAGTTAGTGGGAAAATTGAATTGGGCAAGTCAGATTTATCCAGGGATTAAAGTAAGGCAATTATGTAAGC
TCCTTAGGGGAACCAAAGCACTAACAGAAGTAATACCACTAACAGAAGAAGCAGAGCTAGAACTAGCAGAAAA
CAGGGAGATTCTAAAAGAACCAGTACATGGAGTGTATTATGACCCATCAAAAGACTTAGTAGCAGAAATACAG
AAGCAGGGGCAAGGCCAATGGACATATCAAATTTATCAAGAGCCATGTAAAAATCTGAAAACAGGAAAGTATG
CAAGAATGAGGGGTGCCCACACTAATGATATAAAACAGTTAACAGAGGCAGTGCAAAAAATAACCATGGAAAG
CATAATAATATGGGGAAAGACTCCTAAATTTAAACTACCCATACAAAAGGAAACATGGGAAGCATGGTGGATG
GAGTATTGGCAAGCCACCTGGATTCCTGAGTGGGAGTTTGTCAATACCCCTCCCTTAGTAAAATTATGGTATC
AGTTAGAGAAAGAACCCATAGAGGGAGCAGAAACCTTCTATGTAGATGGGGCAGCTAATAGGGAGACTAAATT
AGGAAAAGCAGGATATGTTACTGACAGAGGAAGACAAAAGGTTGTCTCCCTATCTGACACAACAAATCAGAAG
ACTGAGTTACAAGCAATTCATCTAGCGCTGCAGGATTCGGGACTAGAAGTGAACATAGTAACAGACTCACAAT
ATGCATTAGGAATCATTCAAGCACAACCAGATAAAAGTGAATCAGAGTTAGTCAGTCAAATAATAGAGCAGTT
AATAAAAAAGGAAAAAGTCTACCTGGCATGGGTACCAGCACACAAAGGAATTGGAGGAAATGAACAGGTAGAT
AAATTAGTCAGTACTGGAATCAGGAGAGTACTATTTTTAGATGGAATAGATAAGGCCCAAGAAGAACATGAGA
AATATCACAGTAATTGGAGAGCAATGGCTAGTGATTTTAACCTGCCACCTGTAATAGCAAAAGAAATAGTAGC
CTGCTGTGATAAATGTCAGCTAAAAGGAGAAGCCATACATGGACAAGTAGACTGTAGTCCAGGAATATGGCAA
CTAGATTGTACACATTTAGAAGGAAAATTATCCTGGTGGCAGTTCATGTAGCCAGTGGATATATAGAAGCAG
AAGTCATTCCAGCAGAGACAGGGCAGGAAACAGCATACTTTCTCTTAAAATTAGCAGGAAGATGGCCAGTAAA

AACAATACATACAGACAATGGCAGCAATTTCACCAGTAATGTGGTTAAGGCTGCCTGTTGGTGGGCAGGGATC
AAGCAGGAATTTGGCATTCCCTACAATCCCCAAAGTCAAGGAGTAGTAGAATCTATGAATAAAGAATTAAAGA
AAATTATAGGACAGGTAAGAGATCAGGCTGAACATCTTAAGACAGCAGTACAAATGGCAGTATTCATCCACAA
TTTTAAGAGAAAAGGGGGGATTGGGGGGTACAGTGCAGGGGAAAGAATAGTAGACATAATAGCAACAGACATA
CAAACTAAAGAATTACAAAAACAAATTACAAAAATTCAAAATTTTCGGGTTTATTACAGGGACAGCAGAGATC
CACTTTGGAAAGGACCAGCAAAGCTTCTCTGGAAAGGTGAAGGGGCAGTAGTAATACAAGATAATAGTGATAT
AAAAGTAGTGCCAAGAAGAAAAGCAAAGATCATTAGGGATTATGGAAAACAGATGGCAGGTGATGATTGTGTG
GCAAGTAGACAGGATGAGGATTAGAACATGGAAAAGTTTAGTAAAACACCATATGTATGTTTCACGGAAAGCT
GGGAAATGGTTTTATAGACATCACTATGAAAGCACTCATCCAAAAATAAGTTCAGAAGTACACATCCCACTAG
GGGATGCTAGATTGGTAATAACAACATATTGGGGTCTGCATACAGGAGAAAGAGACTGGCAGTTGGGCCATGG
AGTCTCCATAGAATGGAGGAAAAAGAGATATAGCACACAAGTAGACCCTGATCTAGCAGACCAACTAATCCAT
CTGTATTATTTTGATTGTTTTTCAGAATCTGCTATAAGACATGCCATATTAGGATATATAGTTAGACCTAGTT
GTGAATATCAAGCAGGACATAACAAGGTAGGATCCCTACAATACTTGGCATTAGCAGCATTAATAAAACCTAA
AAAGATAAAGCCACCTTTGCCTAGTGTTAAGAAACTGACAGAGGATAGATGGAACAAGCCCCAGAAGACCAAG
GGCCACAGAGGGAGCCATACAATGAATGGACACTAGAGCTTTTAGAGGAGCTTAAGAGTGAAGCTGTTAGACA
TTTTCCTAGGGCATGGCTACATAGCTTAGGACAATATATCTATGAAAATTATGGGGATACTTGGGCAGGAGTG
GAAGCCATAATAAGAATGCTGCAACAACTGCTGTTTATTCATTTCAGAATTGGGTGTCGCCATAGCAGAATAG
GCATTATTCAACAGAGGAGAGCAAGAAATGGAGCCAGTAGATCCTAGACTAGAGCCCTGGAAGCATCCAGGAA
GTCAGCCTAGGACTCCCTGTACCAATTGCTATTGTAAAAAGTGTTGCCTTCATTGCCAAGTTTGTTTTATGAA
AAAAGGCTTAGGCATCTCCTATGGCAGGAAGAAGCGGAGACAGCGACGAAGACCTCCTCAAGACAGTAAGAAT
CATCAAGCTCCTCTATCAAAGCAGTAAGTAGTACATGTAATGCAATCTTTACAAATATTAGCAATAGTAGCAT
TAGTAGTAGCAGCAATAATAGCAATAGTTGTGTGGACCATAGTAGGCATAGAATATAGGAAAATATTAAGACA
AAGAAAAATAGACAGGTTAATTGATAGAATAAGAGAAAGAGCAGAAGACAGTGGCAATGAAAGCGAAGGAGAC
CAGGAAGAATTATCAGCACTTGTGGAGATGGGGCACCATGCTCCTTGGGATGTTGATGATCTGTAGTGCTGCA
GAACAATTGTGGGTCACAGTCTATTATGGGGTACCTGTGTGGAAAGAAGCAACTACCACTCTATTTTGTGCAT
CAGATGCTAAAGCATATGATACAGAGGTACATAATGTTTGGGCCACACATGCCTGTGTACCCACAGACCCCAA
CCCACAAGAAGTAGTATTGGAAAATGTGACAGAAGAATTTAACATGTGGAAAAACAACATGGTAGAACAGATG
CATGAAGATATAATCAGTCTATGGGATCAAAGCCTAAAGCCATGTGTAAAATTAACCCCACTCTGTGTTACTT
TAAACTGCACTGATTTGAGGAATGTTACTAATGCCACTGATTTGAAGAATGCTACTAATGCCACTAATAGTAA
TGGGACAATAGAGGAAGGAGAAATGAAAAACTGCTCTTTCAATATCACCACAAGCATAAGAGATAAGGTGCAG
AAAGAATATGCACTTTTTTATAGACTTGATGTAGTACAAATAGAGAATGATAATACTAGCAATACTAGCTATA
GGATGATAAATTGTAATACCTCAGTCATTACACAGGCCTGTCCAAAGATATCCTTTGAGCCAATTCCCATACA
TTATTGTGCCCCGGCTGGTTTTGCGATTCTAAAGTGTAACAATAAGACGTTCAATGGAAAAGGACCATGTAAA
AATGTCAGCACAGTACAATGTACACATGGAATTAAGCCAGTAGTGTCAACTCAACTGCTGTTAAATGGCAGTC
TAGCAGAAAAAGAGGTGGTAATTAGATCTGTCAATTTCACAGACAATGCTAAAACCATAATAGTACAGCTGAA
CAAATCTGTAGAAATTAACTGTACAAGACCCAACAACAATACCAGAAAAAGTATACATATAGGACCAGGGAGA
GCATTTTATGCAACAGGAGACATAATAGGAGATATAAGACAAGCACATTGTAACATCAGTAGAACAGAATGGA
ATAACACTTTAAGACAGATAGCTAAAAAATTAAGAGAACAATTTGTGAATAAAACAATAGTCTTTAATCACTC
CTCAGGAGGGGACCCAGAAATTGTAATGCACAGTTTTAATTGTGGAGGGGAATTTTTCTACTGTAATTCAACA
CAACTGTTTAATAGTACTTGGAATGATACTAACATTGACGGAAATAACACCACTAAAGGAAATAATACAGATG
TCATCACACTCCCATGCAGAATAAAACAAATTATAAACATGTGGCAGGAAGTAGGAAAAGCAATGTACGCTCC
TCCCATCAGAGGACAAATTAATTGCTTATCAAATATTACAGGGCTGCTATTAACAAGAGATGGTGGTAGTCAT
AACGGGAATAACACCAATAACACCGAGACCTTCAGACCTGGAGGGGGAGATATGAAAGACAATTGGAGAAGTG
AATTATATAAATATAAAGTAGTACAAATTGAACCATTAGGAGTAGCACCCACCAAGGCAAAGAGAAGAGTGGT
GCAGAGAGAAAAAAGAGCAGTGGGAACAATAGGAGCCATGTTCCTTGGGTTCTTGGGAGCAGCAGGAAGCACT
ATGGGCGCAGCGTCAATGACGCTGACGGTACAGGCCAGACAATTATTGTCTGGTATAGTGCAACAGCAGAGCA
ATTTGCTGAGGGCTATTGAGGCGCAACAGCATATGTTGCAACTCACAGTCTGGGGCATCAAGCAGCTCCAGGC
AAGAGTACTGGCTGTGGAAAGATACCTACAGGATCAACGGCTCCTAGGGATTTGGGGTTGCTCTGGAAAACTC
ATCTGCACCACTGCTGTGCCTTGGAATACTAGTTGGAGTAATAAAAATCTGACTCAGATTTGGGATAACATGA
CCTGGATGCAGTGGGAAAAAGAAATTAACAATTACACAAAAGTAATATACAGCTTAATTGAAGAATCGCAGAA
CCAACAAGAAAAGAATGAACAAGAATTATTGGAATTAGATGAGTGGGCAAGTTTGTGGAATTGGTTTGACATA
ACAAAATGGCTGTGGTATATAAAAATATTCATAATAATAGTAGGAGGCTTGGTAGGTTTGAGAATAGTTTTTG
CTGTACTTTCTATAGTGAATAAAGTTAGGCAGGGATACTCACCATTATCATTTCAGACCCTCCTCCCAGCCCC
GAGGGGACCCGACAGGCCCGAAGGAATA?AAGAAGAAGGTGGAGAGAGAGGCAGAGACAGATCAAATCGATTA

GCAACTGGATTCTTGATACTTTTCTGGGACGACCTGCGGAGCCTGTGCCTCTTCAGCTACCACCGATTGAGAG
ACTTACTCTTGATTGTAGTGAGGATTGTGGAAATTCTGGGACACAGGGGGTGGGAGATCCTCAAATATTGGTG
GAATCTCCTGCAATATTGGAGTCAGGAACTAAAGAATAGTGCTGTTAGCTTGCTCAATGCCACAGCTATCGCA
GTAGCTGAGGGAACAGATAGGGTTATAGAAGTAGTACGAAGAGCTTTTAGAGCTATTCTCCACATACCTACAA
GAGTGAGACAGGGCTTGGAAAGGGCTTTGCTATAAGATGGGTGGCAAGTGGTCAAAACGTAGTCTGGGTGGAT
GGCCTAATGTAAGGGAAAGAATGAGAAGAACTGAGCCAGCAGCAGAGGGGGTGGGAGCAGTATCTCGAGACCT
GGAAAAACATGGAGCAATCACAAGTAGCAATACAGCAACTAGTAATGCTGCCTGTGCCTGGCTAGAAGCACAA
GAGGAAGAGGAGGTGGGTTTTCCAGTCAGACCTCAGGTACCTTTAAGACCAATGACTTACAAGGGAGCTTTAG
ATCTTAGCCACTTTTTAAGAGAAAAGGGGGGACTGGAAGGGCTAATTCACTCCCAGAAAAGACAAGAGATCCT
TGATCTGTGGGTCTACCACACACAAGGCTACTTCCCTGATTGGCAGAACTACACACCAGGGCCAGGGACCAGA
TATCCACTGACCTTTGGATGGTGCTTTAAGTTAGTACCAGTAGAGCCAGACGAGGAAGAGAACAGCAGCTTGC
TACACCCTATGAGCCAGCATGGGATGGAGGACACGGAGAAAGAAGTGTTAAAGTGGAAGTTTGACAGCCGCCT
AGCATTTCGTCACATGGCCCGAGAGCTGCATCCGGAGTATTACAAAGACTGCTGACACCGAGTTTTCTACAGG
GGACTTTCCGCTGGGGACTTTCCAGGGAGGCGTGGCCTGGGCGGGACTGGGGAGTGGCGAGCCCTCAGATGCT
GCATATAAGCAGCTGCTTTTTGCCTGTACTGGGTCTCTCTGGTTAGACCAGATCAGAGCCTGGGAGCTCTCTG
GCTAACTAGGGAACCCACTGCTTAAGCCTCAATAAAGCTTGCCT

>AB-11C HIV-1 genome, derived from RNA genomic sequence
ACGCAGGACTCGGCTTGCTGAAGCGCGCACGGCAAGGGGCGAGGGGCGGCGACTGGTGAGTACGCCAATTTTT
GACTAGCGGAGGCTAGAAGGAGAGAGATGGGTGCGAGAGCGTCAGTATTAAGTGCGGGGGAATTAGATACATG
GGAAAAAATTCGGTTAAGGCCAGGAGGAAAGAAAAAATATAGATTAAAACATATAGTATGGGCAAGCAGGGAG
CTAGAACGATTTGCAGTTAATCCTGGCCTATTAGAAACATCAGCAGGATGTAGACAAATAATGGGACAGCTAC
ATCCATCCCTTCAGACAGGATCAGAAGAACTTAGGTCATTATATAATACAGTAGCAGTCCTCTATTGTGTACA
TCAAAAGATAGAGGTAAAAGACACCAAGGAAGCTTTAGAGAAGGTAGAGGAAGAGCAAAACAAAAGTAAGAAA
AAGGTACAGCAAGCGGCAGCTGACGCAGGAAACAGCAGCCCGGTCAGCCAAAATTACCCTATAGTACAGAACC
TCCAGGGGCAAATGGTACACCAGACCATGTCACCTAGAACTTTAAATGCATGGGTAAAAGTGATAGAAGAGAA
GGCTTTCAGCCCAGAAGTAATACCCATGTTTTCAGCATTATCAGAAGGAGCCACCCCACAAGACTTAAACACC
ATGCTAAACACAGTGGGGGGACATCAAGCAGCCATGCAAATGTTAAAAGATACCATCAATGAGGAGGCTGCAG
AATGGGATAGATTGCATCCAGTGCATGCAGGGCCTATTGCACCAGGCCAGATGAGAGAACCAAGGGGAAGTGA
CATAGCAGGAACTACTAGTACCCTTCAGGAACAAATAGGATGGATGACACATAATCCACCTATCCCGGTAGGA
GAAATCTATAAGAGATGGATAATCCTGGGATTAAATAAAATAGTAAGAATGTATAGCCCTACCAGCATTCTGG
ACATAAAGCAAGGACCAAAAGAACCCTTTAGAGATTATGTAGACCGGTTCTATAAAACTCTAAGAGCCGAGCA
AGCTTCACAGGATGTAAAAAATTGGATGACAGAAACCTTGTTGGTCCAAAATGCGAATCCAGATTGTAAGACC
ATTTTAAAAGCATTGGGACCAGCAGCCACATTAGAAGAAATGATGACAGCATGTCAAGGAGTGGGAGGACCCA
GCCATAAAGCAAGAGTCTTGGCTGAAGCAATGAGCCAAGCAACAGGTCCAGCTAACATAATGATGCAGAGGGG
TAATTTTAGGAACCAAAGAAAGACTGTTAAGTGTTTCAATTGTGGCAAAGAGGGGCACATAGCCAGAAATTGC
AGGGCCCCTAGGAAAAAGGGCTGTTGGAAATGTGGAAAGGAAGGACACCAAATGAAGGATTGCACTGAGAGAC
AGGCTAATTTTTTAGGGAGAATCTGGCCTTCCCACAAGGGGAGGCCAGGGAATTTTCTTCAGAGCAGACCAGA
GCCATCAGCCCCACCAGAAGAGAGCTTCAGGTTTGGGGAGGAAGCAACAACTCCCCCTCAGAAGCAGGAGACG
ATAGACAAGGAACTGTATCCTTTAACCTCCCTCAAATCCCTCTTTGGCAACGACCCATCCTCACAGTAAGGGT
AGGGGGGCACCTAATAGAAGCTCTATTAGATACAGGAGCAGATGATACAGTGTTAGAAGAAATAAATTTACCA
GGAAGATGGAAACCAAAAATGATAGGGGGAATTGGAGGTTTTGTCAAAGTAAGACAATATGAGCAGGTACCCA
TAGAAATCTGTGGGCATGAAGTTATAAGTACAGTATTAGTAGGACCTACACCTGCCAACGTAATTGGAAGAAA
TGTGATGTCTCAAATTGGTTGTACTTTAAATTTTCCCATTAGTCCTATTGAAACTGTACCAGTAAAATTAAAG
CCAGGAATGGATGGCCCAAAAGTTAAACAATGGCCATTGACAGAAGAAAAAATAAAAGCATTAGTAGAAATTT
GTACAGAATTGGAAAAGGAAGGAAAATTTCAAAAATTGGGCCTGAAAATCCGTACAATACTCCAGTATTTGC
CATAAAGAAAAAGAACAGTACTAAATGGAGAAAATTGGTAGATTTCAGAGAACTTAATAAGAGAACTCAAGAC
TTTTGGGAAGTTCAATTGGGAATACCACATCCCGCAGGGTTAAAAAAGAAAAAATCAGTAACAGTCTTGGATG
TGGGTGATGCATATTTTTCAGTTCCCTTGGATGAAGACTTTAGAAAGTATACTGCATTCACCATACCTAGTAC
AAACAATGAGACACCAGGGATTAGATATCAATACAATGTGCTGCCACAGGGATGGAAAGGATCACCAGCAATA
TTCCAAAGTAGCATGACAAAAATCTTAGAGCCTTTTAGAAAACAAAATCCAGACATAGTTATCTATCAATACG
TGGATGATTTGTATGTAAGCTCAGACTTAGAAATAGGGCAGCATAGAAAAAAAATAGAGGACCTGAGAGAACA
TCTGTGGAGGTGGGGATTTTACACACCAGACAAAAAACATCAGAAAGAACCTCCATTCCTCTGGATGGGTTAT
GAACTCCATCCTGATGAATGGACAGTACAGCCTATAGTGCTGCCAGAAAAAGACAGCTGGACTGTCAATGACA
TACAGAAGTTAGTGGGAAAATTGAATTGGGCAAGTCAAATTTATCCAGGGATTAAAGTAAAGCAATTATGTAA
ACTCCTTAAGGGAACCAAGGCACTAACAGAGGTAGTACCACTAACAGAGGAAGCAGAGCTAGAACTAGCAGAA
AACAGGGAAATTCTAAAAGAACCAGTATATGGAGTGTATTATGACCCATCAAAAGAATTAATAGCAGAAATAC
AGAAGCAGGGACAAGGCCAATGGACATATCAAATTTATCAAGAGCAAGGTAAAAATTTGAAAACAGGAAAATA
TGCAAGAATGAGGAGTGCCCACACTAATGATATAAAACAGTTAACAGAGGCAGTGCAAAAAATAAGCATGGAA
AGCATAGTAATATGGGGGAAGACTCCTAAATTTAGACTACCCATACAAAAGGAAACATGGGAAGCATGGTGGA
TGGAGTATTGGCAAGCCACCTGGATTCCTGAGTGGGAGTTTGTCAACACCCCTCCCTTAGTAAAATTATGGTA
TCAGTTAGAGAAAGAACCCATAGTAGGAGCAGAAACCTTCTATGTAGATGGGGCAGCTAATAGGGAAACTAAA
TTAGGAAAAGCAGGATATGTTACTGACAGAGGAAGACAAAAAGTTGTCTCCCTGACTGACACAACAAATCAGA
AGACTGAGTTACAAGCAATTCATCTAGCCTTGCAGGATTCGGGATTAGAAGTAAACATAGTAACAGACTCACA
ATATGCATTAGGAATCATTCAAGCACAACCAGATCAAAGTGAATCAGAGTTAGTCAATCAAATAATAGAGCAG
TTAATAAAAAAGGAAAAAATCTACTTGGCATGGGTACCAGCACATAAAGGGATTGGAGGAAATGAGCAGGTAG
ATAAATTAGTCAGTACTGGAGTCAGGAGAGTACTATTTTTAGATGGAATAGATAAGGCCCAAGAAGAACATGA
GAAATATCACAGTAATTGGAGAGCAATGGCTAGTGATTTTAACCTGCCACCTGTAATAGCAAAAGAAATAGTA
GCCTGCTGTGATAAATGTCAGCTAAAAGGAGAAGCCATGCATGGACAAGTAGACTGTAGTCCAGGAATATGGC
AACTAGATTGTACACATTTAGAAGGAAAAATTATCATAGTAGCAGTTCATGTAGCCAGTGGATATGTAGAAGC
AGAAGTCATTCCAGCAGAGACAGGACAGGAAACAGCATACTTTCTCTTAAAATTAGCAGGAAGATGGCCAGTA

AAAACAATACATACAGACAATGGCAGCAATTTCACCAGTAATGTGGTTAAGGCTGCCTGTTGGTGGGCAGGGA
TCAAGCAGGAATTTGGCATTCCCTACAATCCCCAAAGTCAAGGAGTAGTAGAATCCATGAATAAAGAATTAAA
GAAAATTATAGGACAGGTAAGAGATCAAGCTGAACATCTTAGGACAGCAGTACAAATGGCAGTATTCATTCAC
AATTTTAAGAGAAAAGGGGGGATTGGGGGGTACAGTGCAGGGGAAAGAATAGTAGACATAATAGCAACAGACA
TACAAACTAAAGAGTTACAAAAACAAATTACAAAAGTTCAAAATTTTCGGGTTTATTACAGGGACAGCAGAGA
TCCACTTTGGAAAGGACCAGCAAAACTTCTCTGGAAAGGTGAAGGGGCAGTAGTAATACAAGAAAATAGTGAT
ATAAAAGTAGTGCCAAGAAGAAAAGCAAAGATTATTAGGGATTATGGAAAACAGATGGCAGGTGATGATTGTG
TGGCAAGTAGACAGGATGAGGATTAAAACATGGAAAAGTTTAGTAAAACACCATATGTATGTCTCACGGAAGG
CTGGGAAATGGTTTTATAGACATCACTATGAAAGCACTCATCCAAAAATAAGTTCAGAAGTACACATCCCACT
AGGGGATGCTAGATTGGTAATAACAACATATTGGGGTCTGCAAACAGGAGAAAGAGACTGGCAGTTGGGCCAT
GGAGTCTCTATAGAATGGAGGAAAAGGAGATATAGCACACAAGTAGACCCTGATCTAGCAGACCAACTAATCC
ATCTGTATTATTTTGATTGTTTTTCAGAATCTGCTATAAGACATGCCATATTAGGACATATAGTTAGACCTAG
TTGTGAATATCAAGCAGGACATAACAAGGTAGGATCCCTACAATACTTGGCACTAACAGCACTAATAAAACCA
AAGAAGATAAAGCCACCTTTGCCTAGTGTTAAGAAACTGACAGAGGATAGATGGAACAAGCCCCGGAAGACCA
AGGGCCACAGAGGGAGCCATACAATGAATGGACACTAGAGCTTTTAGAGGAGCTTAAGAGTGAAGCTGTTAGA
CATTTTCCTAGGGCATGGCTACATAGCTTAGGACAATATATCTATGAAACTTATGGGGACACTTGGGCAGGAG
TGGAGGCCATAATAAGAATGCTGCAACAACTGCTATTTATTCATTTCAGAATTGGGTGTCGCCATAGCAGAAT
AGGCATTATTCGACAGAGGAGAGCAAGAAATGGAGCCAGTAGATCCTAGACTAGAGCCCTGGAAGCATCCAGG
AAGTCAGCCTAGGACTCCTTGTACCACTTGTTATTGTAAAAAGTGTTGCCTTCATTGCCAAGTTTGTTTTATG
AAAAAAGGCTTAGGCATCTCCTATGGCAGGAAGAAGCGGAGACAGCGACGAAGACCTCCTCCAGACAGTAAGA
ATCATCAAGTTCCTCCATCAAAACAGTAAGTAGTACATGTAATGCAACCTTTAAATATAGTGGCAATAGTAGC
ATTAGTAGTAGCAGCAATAATAGCAATAGTTGTGTGGACCATAGTAGGCATAGAATATAGGAAAATATTAAGA
CAAAGGAAAATAGATAGGTTAATTGATAGGATAAGAGAAAGAGCAGAAGACAGTGGCAATGAAAGCGAAGGAG
ACCAGGAAGAATTATCAGCACTTGTGGAAATGGGGCACGATGCTCCTTGGGATGTTGCTGATCTGTAGTGCTG
CAGAACAATTGTGGGTCACAGTCTATTATGGGGTACCTGTGTGGAAAGACGCAAATACCACTCTATTTTGTGC
ATCAGATGCTAAAGCATATGAGAGAGAGGCACATAATGTTTGGGCCACACATGCCTGTGTACCCACAGACCCC
GACCCACAAGAAGTAGAATTGGCAAATGTGACAGAGGAATTTAACATGTGGGAAAACGACATGGTAGAACAGA
TGCATGAAGATATAATTAGTTTATGGGATCAAAGCCTAAAGCCATGTGTAAAATTAACCCCGCTCTGTGTCAC
TTTAAATTGCACTGATCTGGGGGATGTTAATACTACTACCCCTAATATTACTACTACTAATGAGACAATAAAG
GGAGAAATGAAAAACTGCTCTTTCAATATTACCACAAGCATAAGAGATAAGGTGCAGAAAGAATATGCACTTT
TTTATAAACTTGATATAGTAGAAATAAAGAATGATAATACTAACAATACTGACAGGACTAACTATACTAGCTA
TATAATGAGACATTGTAATGCCTCAGTCATTACACAGGCCTGTCCAAAGACATCCTTTGAGCCAATTCCAATA
CATTATTGTGCCCCGGCTGGTTTTGCGATTCTAAAGTGTAACAATAAGACATTCGATGGAAAAGGGAACTGTA
CAAATGTCAGCATAGTACAATGTACACATGGAATTAAGCCAGTAGTGTCAACTCAGCTGCTGTTAAATGGCAG
TTTAGCAGAAGAAAAGGTGGTAATTAGATCTGTCAATTTCTCAAACAATGCTAAAACCATAATAGTACAGCTG
AACACATCTGTAGGAATTAACTGTACAAGACCAAACAACAACACAGGAAAAACTATACACCTGGGATGGAGGA
GATCATTTTTTACAACAGAGACCATAATAGGAGATATAAGACGAGCACATTGTAATATTAGTAGAACAGCATG
GAATAACACTTTAAGACAGATAGCTGGAGAGTTAAGAAAACAATTTGGGAATAAAACAATAGCCTTTAATCGC
TCCTCAGGAGGGGACCCAGAGATTGTAATGCACAGTTTTAATTGTGGAGGGGAATTTTTCTACTGTGATACAA
CACAACTGTTTAATAGTACTTGGAATGGGACCGACATTTACAGCCATCGGAATGACACTGACATTAACAGAAA
TACCACAAATAATAAGACAGAGGTCATCACACTCCCATGCAGAATAAAACAAATTGTAAACATGTGGCAGGGA
ATAGGAAAAGCAATGTATGCCCCTCCCATCAGAGGACGAATTTATTGTGTATCAAATATTACAGGGCTGCTAT
TAACAAGAGATGGTGGTAATCAGAGTGGGAGCAACACCACCGAGACCTTCAGACCTCAAGGGGGAGACATGAA
GGACAATTGGAGGAGTGAATTATATAAATATAAAGTAGTACAACTTGAACCATTAGGAGTAGCACCCACCAAG
GCAAAGAGAAGAGTGGTGCAGAGAGAAAAAAGAGCAGTGGGAATGCTAGGAGCCATGTTCCTTGGGTTCTTGG
GAGCAGCAGGAAGCACTATGGGCGCAGCGTCAATGACGCTGACGGTACAGGCCAGACAATTAATGTCTGGTAT
AGTGCAACAGCAGAGCAATTTGCTGAGGGCTATTGAGGCGCAACAGCGCATGTTGCAACTCACAGTCTGGGGC
ATCAAGCAGCTCCAGGCAAGAGTACTGGCTGTGGAAAGATACCTACAGGATCAACGGCTCCTAGGGATTTGGG
GTTGCTCTGGAAAGCTCATCTGCACCACTGCTGTGCCTTGGAATAGTAGTTGGAGTAATAAAAATCTGACTCA
GATTTGGGATAACATGACCTGGATGCAGTGGGAAAAAGAAATTAACAATTACACAGGAGTAATATACAACTTA
CTTGAAGAATCGCAACACCAACAAGAAAAGAATGAACAAGAATTGTTGGAATTAGATAAGTGGGACAGTTTAT
GGAATTGGTTTGACATAACACAGTGGCTGTGGTATATAAAAATATTCATAATGATAGTAGGAGGCTTGATAGG
TTTAAGAATAATTTTTGCTGTGCTTTCTATAGTGAATAAAGTTAGGCAGGGATATTCACCATTATCATTTCAG
ACCCTCCTCCCGGTCCCGAGGGGACCCGACGGCCCGAAGGAACAGAAGAAGAAGGTGGAGAGAGAGACAGAG

GCAGATCAAACAGATTAGCAACTGGATTCTTGACAATTTTCTGGGAAGACCTACGGAACCTGTGCCTCTTCCT
CTACCACCGCTTGAGAGACTTACTCTTGATTGTAGGGAGGATTGTGGAAATTCTGGGACGCAGGGGGTGGGAG
ATCCTCAAATATTGGTGGAACCTCCTGCAATATTGGAGTCAGGAACTAAAGAATAGTGCTGTTAGCTTGCTCA
ATGCCACAGCTATTGCAGTAGCTGAGGGAACAGATAGGGTTATAGAAGTAGTGCAAAGAGCTTTTAGAGCTAT
TCTCCATATACCTACAAGAATAAGACAGGGCTTGGAAAGGGCTTTGCTATAAGATGGGTGGTAAGTGGTCAAA
ACATCTGAGTGGGTGGCCTAAGGTAAGGGAAAGAATAGAAAGAACTGAGCCAGCAGCAGATGGGGTGGGAGCA
GTTTCTCGAGACCTGGAAAAACATGGAGCAATCACAAGTAGCAATACAGCAACCACTAATGCTGCCTGTGCCT
GGCTAGAAGCACAAGAAGAAGAGGAGGTGGGTTTTCCAGTCAGACCTCAGGTACCTTTAAGACCCATGACTTA
CAAGGGAGCTTTAGATCTTAGCCACTTTTTAAGAGAAAAGGGGGGACTGGAAGGGTTAATTTACTCCCAGAAA
AGACAAGAGATCCTTGATCTGTGGGTCTACCACACACAAGGCTACTTCCCTGATTGGCAGAACTACACACCAG
GGCCAGGGATCAGATATCCCCTGACCTTTGGATGGTGCTTCAAGTTAGTACCAGTTGAGCCAGACGAGGAAGA
GAACAGCAGCCTGCTACACCCTATGAGCCAGCATGGAATGGGGGACACGGAGAAAGAAGTGTTAAAGTGGAAG
TTTGACAGCCACCTAGCATTCCGTCACATGGCCCGAGAGCTGCATCCGGAGTATTACAAAGACTGCTGACACC
GAGCTTTCTGCAAGGGACTTTCCGCTGGGGACTTTCCAGGGAGGCGTGGCCTGGGCGGGACTGGGGAGTGGCG
AGCCCTCAGATGCTGCATATAAGCAGCTGCTTTTTGCCTGTACTGGGTCTCTCTGGTTAGCCCAGATCCGAGC
CTGGGAGCTCTCTGGCTAACTAGGGAACCCACTGCTTAAGCCTCAATAAAGCTTGCCT

```
>AB-11D HIV-1 genome, derived from RNA genomic sequence
ACGCAGGACTCGGCTTGCTGAGGTGCACACGGCAAGAGGCGAGGGGCGGCGACTGGTGAGTACGCCGAAAACT
TTTGACTAGCGGAGGCTAGAAGGAGAGAGATGGGTGCGAGAGCGTCAGTATTAAGCGGTAGCGAATTAGATCG
GTGGGAAAAAATTCGGTTAAGGCCAGGGGGAAGGAAAAAGTACCAGTTAAAACATTTAATATGGGCAAGCAGG
GAGCTAGAGCGATTTGCAGTCAATCCTGGCCTGTTAGAAACATCAGAAGGCTGCAGACAAATAATAGGACAGC
TACAACCATCCCTACAGACAGGATCAGAAGAACTTAAATCATTATTTAATACAGTAGCAGTCCTCTATTGTGT
ACATCAAAGGATAACAGTAAAAGACACCAAGGAAGCTTTAGAAAAGATAGAGGAAGAGCAAAACAAAAGTAAG
AAAAAAGCACAACAAGCAGCAGCTGACACAGGAAACGGCAACCAGGTCAGCCAAAATTACCCTATAGTGCAGA
ACATGCAGGGGCAAATGGTACATCAGGCTATCTCACCTAGAACTTTAAATGCATGGGTGAAAGTAGTAGAAGA
GAAGGCTTTCAGCCCAGAAGTAATACCCATGTTTTCAGCATTATCAGAAGGAGCCACCCCTCAAGATTTAAAC
ACCATGCTAAACACAGTGGGGGGGCACCAAGCAGCCATGCAAATGTTAAAAGAGACCATCAATGAGGAAGCTG
CAGAATGGGATAGAACACACCCAGCGCAGGCAGGGCCTGTTGCACCAGGCCAGATGAGAGAACCAAGGGGGAG
TGACATAGCAGGAACTACTAGCACCCTTCAGGAACAGGTGGGATGGATGACAAGTAATCCACCCATCCCAGTA
GGAGAAATTTATAAAAGATGGATAATCATGGGATTAAACAAAGTAGTAAGAATGTATAGCCCTACCAGCATTC
TAGACATAAGACAAGGACCAAAGGAACCTTTTAGAGATTATGTAGACCGGTTCTATAAAACTCTAAGAGCTGA
GCAAGCTTCACAGGAGGTAAAACACTGGATGACAGAAACCTTGTTGGTCCAAAATGCAAACCCAGATTGTAAG
ACTATCTTAAAGGCATTGGGACCAGCGGCTACACTAGAAGAAATGATGACGGCATGTCAGGGAGTGGGAGGAC
CCAGCCATAAAGCAAGAGTTTTGGCTGAAGCAATGAGCCAAGCAACAGCTGCTTCAGCTAGCATAATGATGCA
GAAGGGCAATTTTAGGCACCAAAAGAAAAGTGTTAAGTGTTTCAATTGTGGCAAAGAAGGGCACATAGCCAGA
AATTGCAGGGCCCCTAGGAGAAAAGGCTGTTGGAAATGTGGAAGGGAAGGACACCAAATGAAAGAATGCTCTG
AGAGACAGGCTAATTTTTTAGGGAAAATCTGGCCTTCCCACAAGGGGAGGCCAGGGAATTTCCTTCAGAACAG
ACCAGAGAGCAGACTAGAGCCAACAGCCCCACCAGAGGAGAGCCTCAGGGAAGTGACAGCAGCTCCTTCTCAG
AAACAGGAGACGATAGACAAGGATCTGTATCCTTTGACTTCCCTCAGATCACTCTTTGGCAACGACCCATCGT
CACAATAAAGATAGGGGGGCAGCTAAAGGAAGCTCTATTAGATACAGGAGCAGATGATACAGTATTAGAAGAA
ATGAATTTGCCAGGAAGATGGAAACCAAAAATAATAGGGGGAATTGGAGGTTTTATCAAAGTAAGACAGTATG
ATCAAGTACCCATAGAAATTTGTGGGCATAAAGCTGAAGGTACAGTATTAATAGGACCTACACCTGTCAACAT
AATTGGAAGAAACCTGCTGACTCAGCTTGGTTGCACTTTAAATTTTCCCATTAGTCCTATTGAAACTGTACCA
GTAAAATTAAAGCCAGGAATGGATGGCCCAAAGGTTAAACAATGGCCATTGACAGAAGAAAAAATAAAGGCAT
TAGTAGGAATTTGTGCAGAAATGGAAAAGGAAGGCAAAATTTCAAAAATTGGGCCTGAAAATCCATACAACAC
TCCAGTATTTGCTATAAAGAAAAAGAACAGTGATAAATGGAGAAAATTAGTAGATTTCAGAGAACTTAATAAG
AGAACTCAAGACTTCTGGGAAGTTCAATTAGGAATACCACATCCTGCAGGGTTAAAAAAGAAGAAATCAGTAA
CAGTGCTGGATGTGGGTGATGCATATTTTTCAATTCCCTTAGATAAAGACTTCAGGAAGTATACTGCATTTAC
CATACCTAGCATAAATAATGAGACACCAGGGATCAGATATCAGTACAATGTGCTTCCACAGGGATGGAAGGGA
TCACCAGCAATATTCCAAAGTAGCATGACAAAAATCTTAGAGCCTTTTAGAAAACACAATCCAGACATAGTTA
TCTATCAGTACATGGATGACTTATATGTAGGATCTGATTTAGAAATAGGGGAGCATAGAACAAAAATTGAGGA
ACTGAGACAACATCTGTTGAAGTGGGGACTTCTCACACCAGACCAAAAACATCAGAAAGAACCCCCATTCCTC
TGGATGGGTTATGAACTCCATCCTGATAAATGGACAGTACAGCCTATAGTGCTGCCAGACAAAGATAGCTGGA
CTGTCAATGACATACAAAAGTTAGTGGGAAAATTGAATTGGGCAAGTCAGATTTACCCAGGGATTAAAGTAAA
GCAATTATGTAAACTTCTTAGGGGAACCAAAGCACTAACAGAAGTAGTACCACTAACAGAAGAAGCAGAGCTA
GAACTAGCAGAAAACAGGGAGATTCTAAAAGAACCGGTACATGGGGCCTATTATGACCCATCAAAAGACTTAA
TAGCAGAAATACAGAAGCAGGAGCACGGTCAATGGACATATCAAATTTATCAAGAGCCATTTAAAAATCTGAA
AACAGGAAAGTATGCAAAAACTAGGGGTACCCATACCAATGATGTAAAACAGTTAACAGAGGCAGTGCAAAAA
ATAGCCACAGAAAGCATAGTAATATGGGGAAAGACTCCTAAATTTAGACTGCCCATACAGAAAGAAACATGGG
ACACATGGTGGACAGAGTATTGGCAGGCTACCTGGATTCCTGAGTGGGAGTTTGTCAACACCCCTCCTTTAGT
GAAATTATGGTACCAGCTAGAAAAGGAACCCATAGTAGGAGCAGAAACTTTCTATGTAGATGGGGCAGCCAAC
AGGGAGACTAAATTAGGAAAAGCAGGATATGTTACTGACAAAGGGAGACAAAAGGTTATCTCCCTAGATGACA
CAACAAATCAGAAGACTGAATTACAAGCAATTCATCTAGCTTTGCAAGATTCAGGATTAGAAGTAAACATAGT
AACAGACTCACAATATGCATTAGGAATTATTCAAGCACAACCAGATAAAAGTGAATCAGAGATAGTCAGTCAA
ATAATAGAGCAGCTAATAAAAAAGGAAAAGGTCTATCTGGCATGGGTACCAGCACACAAAGGAATTGGAGGAA
ATGAAAAAGTGGACAAATTAGTCAGTACTGGAATCAGGAAAGTACTATTTTTAGATGGAATAGATAGGGCCCA
AGAAGAACATGAGAAATATCACAGTAATTGGAGAGCAATGGCTAGTGATTTTAACCTGCCACCTATAGTAGCA
AAAGAAATAGTAGCCAGTTGTGATAAATGTCAACTAAAAGGAGAAGCCATGCATGGACAAGTAGATTGTAGCC
CAGGAATATGGCAATTAGATTGTACACATTTAGAAGGAAAAATTATCCTGGTAGCAGTCCATGTAGCCAGTGG
ATACATAGAAGCAGAAGTTATTCCAGCAGAAACAGGGCAGGAAACAGCATACTTTATCTTAAGATTAGCAGGG
```

AGATGGCCAGTAAAAACAATACATACAGACAATGGCAGCAATTTCACCAGTGCTACAGTCAAGGCCGCCTGTT
GGTGGACGGGGATCAAACAGGAATTTGGCATCCCCTACAATCCCCAAAGTCAAGGGGTAGTAGAATCTATGAA
TAATGAATTAAAGAAAATTATAGGACAAGTAAGAGATCAGGCTGAACATCTTAAGACAGCAGTACAAATGGCA
GTATTCATCCACAATTTTAAAAGAAGAGGGGGGATTGGGGGGTACAGTGCAGGGGAAAGAATAATAGACATAA
TAGCAACAGACATACAAACTAAAGAATTACAAAAACAAATTACAAATATTCAAAAATTTCGGGTTTATTACAG
GGACAGCAGAGATCCACTTTGGAAAGGACCAGCAAAGCTTCTCTGGAAAGGTGAAGGGGCGGTAGTAATACAA
GATAATAGTGACATAAAAGTAGTGCCAAGAAGAAAAGCAAAAATCATTAGGGATTATGGAAAACAGATGGCAG
GTGATGATTGTGTGGCAAGTAGACAGGATGAGGATTAGAACATGGATAAGTTTAGTAAAACATCATATACATA
TTTCAAAGAAAGCTAAGAAATGGGTTTATAAACATCACTATGAAAACAATCATCCAAGAATAAGTTCAGAAGT
ACACATCCCACTAGGGGATGCTAAATTGGTAATAACAACATATTGGGGTCTGCATACAGGAGAAAGAGACTGG
CATTTGGGTCAGGGAGTCTCCATAGAATGGAGGCAAAGGAGTTATGTCACACAAGTAGATCCTAACCTAGCAG
ACCAACTAATTCATCTGTATTACTTTGATTGTTTTTCAGAATCTGCTATAAGAAAAGCCATAATAGGACATAT
AGTTAATCCTAGTTGTGAATACCCAGCAGGACATAATAAGGTAGGGTCTTTACAGTACTTGGCACTAACAGCA
TTGACAACACCAAAAAGAAGTAAGCCACCTTTACCTAGTATTACAAAGCTGACAGAGGATAGATGGAACAAGC
CCCGGAAGACCAAGGGCCACAGAGAGAGCCATTCAATGAGTGGACAATAGAACTTTTAGAAGAGCTTAAGCAT
GAAGCTGTTAAACACTTTCCTAGGCCATGGCTTCAAGGCTTAGGACACTATATCTATGAAACTTATGGGGATA
CCTGGGCAGGAGTGGAAGCCATAATAAGAATCCTGCAACAACTGCTGTTTATCCATTTCAGAATTGGGTGCCG
ACATAGCAGAATAGGCATCAACACTCGACAGAGGAGAGCAAGAAATGGATCCAGTAGATCCTAGATTAGAGCC
CTGGAAGCATCCAGGAAGCCAGCCTAAAACTGCTTGTAATAATTGCTATTGTAAAAACTGTTGCTATCATTGC
CAAGTTTGTTTCTTGAAAAAAGGCTTAGGCATCTCCTATGGCAGGAAGAAGCGGAGACAGCGACGAAGAGCTC
CTGAAAGCAGTCAGACTCATCAAGAAGCTCTATCAAAGCAGTAAGTAGTAAATGTAATGTGGTCCTTAGTAAT
AGTAGCAATAGTAGCATATATAGTAGCAATAATAATAGCAATAGTTGTGTGGTCTATAGTGTATATAGAATAT
AAGAAAATAGTAAGGCAAAGAAAAATAGATAGGTTAATTGATAGAATTACAGAAAGAGCAGAAGACAGTGGCA
ATGAGAGTGATGGGGATCAGGAAGAATTGTCAGCACTGGTGGAAATGGGGCACGTTGCTCCTTGGAATGTTAA
TGATGATATGTAGTGCTGAAGAAGAATTGTGGGTCACAGTACATTATGGGGTACCTGTGTGGAAAGAAGCAAC
CACCACTCTATTTTGTGCATCAGATGCTAAAGCTTATGATACAGAGAAACATAATGTTTGGGCCACACATGCC
TGTGTACCCACAGACCCCAACCCACAAGAAGTACTATTGGTAAATGTGACAGAAAATTTTAATATGTGGAAAA
ATGGCATGGTAGAGCAGATGCATGAGGATATAATTAGTTTATGGGATCAAAGTCTAAAGCCATGTGTAAAATT
AACCCCACTCTGTGTTACCTTAAATTGCACAGATGCGAGAAATAATACTGTTGCCAATAGTACTAATGCCAAT
AATAGTAGCTTGCAATGGATGGAACAGGGAGAAATAAAAAACTGCTCTTTTAATATCACTTCAACCATAAAAG
ATAAGATGCAGGAAGCACGTGCACTTTTTTATAAATCTGATGTAGTACCAATAGATAAAAGTAATGATACTTA
TACGTTGATAAGTTGTAATACCTCAGTCCTTACACAGGCCTGTCCAAAAATATCCTTTGAGCCAATTCCCATA
CATTATTGTGCCCCGGCTGGTTTTGCGATTATAAAGTGTAACAATAAGACATTCAATGGAAAAGGACCGTGTA
CAAATGTCAGCACAGTACAATGTACACATGGAATTAAGCCAGTAGTGTCAACTCAATTGCTGTTGAATGGCAG
CCTAGCAGAAGATGAGGTAGTAATTAGCTCTGAAAATCTCACAAATAATGCTAAGGTTATAATAGTACATCTG
AATGAATCAGTAGAAATTAATTGTACAAGACCCAACAACAATACAAGAAAAAGTATACATATAGGACCAGGGC
GCGCATTTTATGCAACAGGAGATATAGTAGGAGATATAAGACAAGCACATTGTAACATTAGTAGAGAAAGATG
GAATAACACCTTACAAAAAATAGTTGGAAAACTAAGAGAACATTTTGTGAACAAAACAATAGTCTTTAATAGA
TCCTCAGGAGGGGACCCAGAAATTGTAATGCACAATTTTAATTGTGGAGGGGAATTTTTCTACTGTAATACAA
CACAACTGTTTAATAGTACTTGGTCTTCTAATGGCACTTACACAGGGCCTAAAAGCAATGACACAACAACAAT
CACACTCCCATGCAGAATAAAACAAATTATAAACTTGTGGCAGGAAGTAGGAAAAGCAATGTATGCCCCTCCT
ATCCAAGGATTAATTAAATGCTCATCAAATATTACAGGGCTAATATTAACAAGAGATGGTGGTAATAATTACA
CTAACAGCACCGAGGAGATCTTCAGACCTGCAGGAGGAGATATGAGGGACAATTGGAGGAGTGAATTATATAA
ATATAAGGTAGTACAAATTGAACCAATAGGACTGGCACCCTCCAAGGCAAAGAGAAGAGTGGTGCAGAGAGAG
AAAAGAGCAGTGGGATTAGGAGCTGTGTTCCTTGGGTTCTTGGGAGCAGCAGGAAGCACTATGGGCGCAGCGT
CAATAACGCTGACGGTACAAGCCAGACAATTGTTGTCTGGTATAGTGCAGCAGCAGAACAATCTGCTGAGAGC
TATTGAGGCGCAACAGCATATGTTGCAACTCACAGTCTGGGGCATCAAGCAGCTCCAGGCAAGAGTCCTGGCT
CTGGAAAGATACCTGAGGGATCAACAGCTCCTGGGAATTTGGGGTTGCTCTGGAAAACTCATCTGCACCACTG
CTGTGCCTTGGAATGCTAGTTGGAGCAATAATAGATCTCTGAATAGTATTTGGAATAACATGACCTGGATGGA
GTGGGAAAGAGAAATTGATAATTACACAGACTTAATATACAACTTACTTGAAGAATCGCAAAACCAACAAGAA
AGGAATGAACAAGAGTTATTGGAATTAGATAAATGGGCAAGTTTGTGGAATTGGTTTACCATAACAAATTGGC
TGTGGTATATAAAAATATTCATAATGATAGTAGCAGGCTTGGTAGGGTTAAGGATAGTTTTTCTTATACTTTC
TGTAGCGAATAGAGTTAGGCAGGGATACTCACCATTATCGTTTCAGACCCACCTCCCAACACAGAGGGGACCC
GACAGGCCCGGAGGAACAGAAGAAGAAGGTGGAGGGAGAGACAGAGGCAGATCCGAAGGCTTAGTGGATGGAT

TCTTAGCACTTTTGTGGAGCGATCTGAGGAGCCTGTTACTTTTCCTCTACCACCGCTTGAGAGACTTACTCTT
GATTGCAGCGAGGATTGTGGAACTTCTGGGACGCAGGGGGTGGGAACTCCTCAAGTATTGTTGGAATCTCCTA
CAGTATTGGAGCCTGGAACTGAAGAATAGTGCTATTAGCTTGCTTAATGCTATAACCATAGCAGTAGCTGAGG
GGACAGATAGAATTATAGAAGTAATACAAATAGTTGTTAGGGGTATTCTCCACATACCTAGAAGAATAAGACA
GGGCCTCGAAAGGATTTTGCTATAAGATGGGTGGTAAGTGGTCAAAACGTAGTGTGCCTGGATGGGCTACTAT
AAGGGAAAGAATGAGACGAGCTGAGCCAACAACAGAGCCAGCAGCAGATGGGGTGGGAGCGGTATCTCGAGAC
CTGGAAAGACATGGAGCAATCACAAGTAGCAACACAGCATCTACCAATGCTGATTGCGCCTGGCTAGAAACAC
AAGAGGATGAGGAAGTAGGTTTTCCAGTCAGACCTCAGGTGCCTTTAAGACCAATGACTTACAAGGCATCTTT
GGATCTTAGCCACTTTTTAAAAGAAAAGGGGGGACTGGACGGGCTAATTTGGTCCCAAAGGAGACAAGACATC
CTTGATCTGTGGGTCTACCACACACAGGGCTTCTTCCCTGATTGGCAGAACTACACACCAGGGCCAGGGACCA
GATACCCCCTGACCTTTGGGTGGTGCTTCAAGCTAGTACCAGCTGATCCAGAACAGGTAGAGAAAGCCAATGA
AGGAGAGAACATCAATCTGTTACACCCTATGAGCCAGCATGGAATGGATGACCCAGAGAAAGAAGTGCTGCTG
TGGAAGTTTGACAGCCGCCTAGCCTTCCATCACATGGCCCGCGAGCTACATCCGGAGTACTACAAGGACTGCT
GACATCGAGCTATCTACAAGGGACTTTCCGCTGGGGACTTTCCAGGGAAGGCGTGGCCTGGGCGGGACCGGGG
AGTGGCAAACCCTCAGATGCTGCATATAAGCAGCTGCTTTCGCCTGTACTGGGTCTCTCTGGTTAGACCAGAT
CAGAGCCTGGGAGCTCTCTGGCTAGCTAGGGAACCCACTGCTTAAGCCTCAATAAAGCTTGCCT

>AB-12B HIV-1 genome, derived from RNA genomic sequence
ACGCAGGACTCGGCTTGCTGAAGCGCGCACGGCAAGGGGCGAGGGGCGGCGACTGGTGAGTACGCCAATTTTT
GACTAGCGGAGGCTAGAAGGAGAGAGATGGGTGCGAGAGCGTCAGTATTAAGTGCGGGGGAATTAGATACATG
GGAAAAAATTCGGTTAAGGCCAGGAGGAAAGAAAAAATATAGATTAAAACATATAGTATGGGCAAGCAGGGAG
CTAGAACGATTTGCAGTTAATCCTGGCCTATTAGAAACATCAGCAGGATGTAGACAAATAATGGGACAGCTAC
ATCCATCCCTTCAGACAGGATCAGAAGAACTTAGGTCATTATATAATACAGTAGCAGTCCTCTATTGTGTACA
TCAAAAGATAGAGGTAAAAGACACCAAGGAAGCTTTAGAGAAGGTAGAGGAAGAGCAAAACAAAAGTAAGAAA
AAGGTACAGCAAGCGGCAGCTGACGCAGGAAACAGCAGCCCGGTCAGCCAAAATTACCCTATAGTACAGAACC
TCCAGGGGCAAATGGTACACCAGACCATGTCACCTAGAACTTTAAATGCATGGGTAAAAGTGATAGAAGAGAA
GGCTTTCAGCCCAGAAGTAATACCCATGTTTTCAGCATTATCAGAAGGAGCCACCCCACAAGACTTAAACACC
ATGCTAAACACAGTGGGGGGACATCAAGCAGCCATGCAAATGTTAAAAGATACCATCAATGAGGAGGCTGCAG
AATGGGATAGATTGCATCCAGTGCATGCAGGGCCTATTGCACCAGGCCAGATGAGAGAACCAAGGGGAAGTGA
CATAGCAGGAACTACTAGTACCCTTCAGGAACAAATAGGATGGATGACACATAATCCACCTATCCCGGTAGGA
GAAATCTATAAGAGATGGATAATCCTGGGATTAAATAAAATAGTAAGAATGTATAGCCCTACCAGCATTCTGG
ACATAAAGCAAGGACCAAAAGAACCCTTTAGAGATTATGTAGACCGGTTCTATAAAACTCTAAGAGCCGAGCA
AGCTTCACAGGATGTAAAAAATTGGATGACAGAAACCTTGTTGGTCCAAAATGCGAATCCAGATTGTAAGACC
ATTTTAAAAGCATTGGGACCAGCAGCCACATTAGAAGAAATGATGACAGCATGTCAAGGAGTGGGAGGACCCA
GCCATAAAGCAAGAGTCTTGGCTGAAGCAATGAGCCAAGCAACAGGTCCAGCTAACATAATGATGCAGAGGGG
TAATTTTAGGAACCAAAGAAAGACTGTTAAGTGTTTCAATTGTGGCAAAGAGGGGCACATAGCCAGAAATTGC
AGGGCCCCTAGGAAAAAGGGCTGTTGGAAATGTGGAAAGGAAGGACACCAAATGAAGGATTGCACTGAGAGAC
AGGCTAATTTTTTAGGGAGAATCTGGCCTTCCCACAAGGGGAGGCCAGGGAATTTTCTTCAGAGCAGACCAGA
GCCATCAGCCCCACCAGAAGAGAGCTTCAGGTTTGGGGAGGAAGCAACAACTCCCCCTCAGAAGCAGGAGACG
ATAGACAAGGAACTGTATCCTTTAACCTCCCTCAAATCCCTCTTTGGCAACGACCCATCCTCACAGTAAGGGT
AGGGGGGCACCTAATAGAAGCTCTATTAGATACAGGAGCAGATGATACAGTGTTAGAAGAAATAAATTTACCA
GGAAGATGGAAACCAAAAATGATAGGGGGAATTGGAGGTTTTGTCAAAGTAAGACAATATGAGCAGGTACCCA
TAGAAATCTGTGGGCATGAAGTTATAAGTACAGTATTAGTAGGACCTACACCTGCCAACGTAATTGGAAGAAA
TGTGATGTCTCAAATTGGTTGTACTTTAAATTTTCCCATTAGTCCTATTGAAACTGTACCAGTAAAATTAAAG
CCAGGAATGGATGGCCCAAAAGTTAAACAATGGCCATTGACAGAAGAAAAAATAAAAGCATTAGTAGAAATTT
GTACAGAATTGGAAAAGGAAGGGAAAATTTCAAAAATTGGGCCTGAAAATCCGTACAATACTCCAGTATTTGC
CATAAAGAAAAAGAACAGTACTAAATGGAGAAAATTGGTAGATTTCAGAGAACTTAATAAGAGAACTCAAGAC
TTTTGGGAAGTTCAATTGGGAATACCACATCCCGCAGGGTTAAAAAAGAAAAAATCAGTAACAGTCTTGGATG
TGGGTGATGCATATTTTTCAGTTCCCTTGGATGAAGACTTTAGAAAGTATACTGCATTCACCATACCTAGTAC
AAACAATGAGACACCAGGGATTAGATATCAATACAATGTGCTGCCACAGGGATGGAAAGGATCACCAGCAATA
TTCCAAAGTAGCATGACAAAAATCTTAGAGCCTTTTAGAAAACAAAATCCAGACATAGTTATCTATCAATACG
TGGATGATTTGTATGTAAGCTCAGACTTAGAAATAGGGCAGCATAGAAAAAAAATAGAGGACCTGAGAGAACA
TCTGTGGAGGTGGGGATTTTACACACCAGACAAAAAACATCAGAAAGAACCTCCATTCCTCTGGATGGGTTAT
GAACTCCATCCTGATGAATGGACAGTACAGCCTATAGTGCTGCCAGAAAAAGACAGCTGGACTGTCAATGACA
TACAGAAGTTAGTGGGAAAATTGAATTGGGCAAGTCAAATTTATCCAGGGATTAAAGTAAAGCAATTATGTAA
ACTCCTTAAGGGAACCAAGGCACTAACAGAGGTAGTACCACTAACAGAGGAAGCAGAGCTAGAACTAGCAGAA
AACAGGGAAATTCTAAAAGAACCAGTATATGGAGTGTATTATGACCCATCAAAAGAATTAATAGCAGAAATAC
AGAAGCAGGGACAAGGCCAATGGACATATCAAATTTATCAAGAGCAAGGTAAAAATTTGAAAACAGGAAAATA
TGCAAGAATGAGGAGTGCCCACACTAATGATATAAAACAGTTAACAGAGGCAGTGCAAAAAATAAGCATGGAA
AGCATAGTAATATGGGGGAAGACTCCTAAATTTAGACTACCCATACAAAAGGAAACATGGGAAGCATGGTGGA
TGGAGTATTGGCAAGCCACCTGGATTCCTGAGTGGGAGTTTGTCAACACCCCTCCCTTAGTAAAATTATGGTA
TCAGTTAGAGAAAGAACCCATAGTAGGAGCAGAAACCTTCTATGTAGATGGGGCAGCTAATAGGGAAACTAAA
TTAGGAAAAGCAGGATATGTTACTGACAGAGGAAGACAAAAAGTTGTCTCCCTGACTGACACAACAAATCAGA
AGACTGAGTTACAAGCAATTCATCTAGCCTTGCAGGATTCGGGATTAGAAGTAAACATAGTAACAGACTCACA
ATATGCATTAGGAATCATTCAAGCACAACCAGATCAAAGTGAATCAGAGTTAGTCAATCAAATAATAGAGCAG
TTAATAAAAAAGGAAAAAATCTACTTGGCATGGGTACCAGCACATAAAGGGATTGGAGGAAATGAGCAGGTAG
ATAAATTAGTCAGTACTGGAGTCAGGAGAGTACTATTTTTAGATGGAATAGATAAGGCCCAAGAAGAACATGA
GAAATATCACAGTAATTGGAGAGCAATGGCTAGTGATTTTAACCTGCCACCTGTAATAGCAAAAGAAATAGTA
GCCTGCTGTGATAAATGTCAGCTAAAAGGAGAAGCCATGCATGGACAAGTAGACTGTAGTCCAGGAATATGGC
AACTAGATTGTACACATTTAGAAGGAAAAATTATCATAGTAGCAGTTCATGTAGCCAGTGGATATGTAGAAGC
AGAAGTCATTCCAGCAGAGACAGGACAGGAAACAGCATACTTTCTCTTAAAATTAGCAGGAAGATGGCCAGTA

AAAACAATACATACAGACAATGGCAGCAATTTCACCAGTAATGTGGTTAAGGCTGCCTGTTGGTGGGCAGGGA
TCAAGCAGGAATTTGGCATTCCCTACAATCCCCAAAGTCAAGGAGTAGTAGAATCCATGAATAAAGAATTAAA
GAAAATTATAGGACAGGTAAGAGATCAAGCTGAACATCTTAGGACAGCAGTACAAATGGCAGTATTCATTCAC
AATTTTAAGAGAAAAGGGGGGATTGGGGGGTACAGTGCAGGGGAAAGAATAGTAGACATAATAGCAACAGACA
TACAAACTAAAGAGTTACAAAAACAAATTACAAAAGTTCAAAATTTTCGGGTTTATTACAGGGACAGCAGAGA
TCCACTTTGGAAAGGACCAGCAAAACTTCTCTGGAAAGGTGAAGGGGCAGTAGTAATACAAGAAAATAGTGAT
ATAAAAGTAGTGCCAAGAAGAAAAGCAAAGATTATTAGGGATTATGGAAAACAGATGGCAGGTGATGATTGTG
TGGCAAGTAGACAGGATGAGGATTAAAACATGGAAAAGTTTAGTAAAACACCATATGTATGTCTCACGGAAGG
CTGGGAAATGGTTTTATAGACATCACTATGAAAGCACTCATCCAAAAATAAGTTCAGAAGTACACATCCCACT
AGGGGATGCTAGATTGGTAATAACAACATATTGGGGTCTGCAAACAGGAGAAAGAGACTGGCAGTTGGGCCAT
GGAGTCTCTATAGAATGGAGGAAAAGGAGATATAGCACACAAGTAGACCCTGATCTAGCAGACCAACTAATCC
ATCTGTATTATTTTGATTGTTTTTCAGAATCTGCTATAAGACATGCCATATTAGGACATATAGTTAGACCTAG
TTGTGAATATCAAGCAGGACATAACAAGGTAGGATCCCTACAATACTTGGCACTAACAGCACTAATAAAACCA
AAGAAGATAAAGCCACCTTTGCCTAGTGTTAAGAAACTGACAGAGGATAGATGGAACAAGCCCCGGAAGACCA
AGGGCCACAGAGGGAGCCATACAATGAATGGACACTAGAGCTTTTAGAGGAGCTTAAGAGTGAAGCTGTTAGA
CATTTTCCTAGGGCATGGCTACATAGCTTAGGACAATATATCTATGAAACTTATGGGGACACTTGGGCAGGAG
TGGAGGCCATAATAAGAATGCTGCAACAACTGCTATTTATTCATTTCAGAATTGGGTGTCGCCATAGCAGAAT
AGGCATTATTCGACAGAGGAGAGCAAGAAATGGAGCCAGTAGATCCTAGACTAGAGCCCTGGAAGCATCCAGG
AAGTCAGCCTAGGACTCCTTGTACCACTTGTTATTGTAAAAAGTGTTGCCTTCATTGCCAAGTTTGTTTTATG
AAAAAAGGCTTAGGCATCTCCTATGGCAGGAAGAAGCGGAGACAGCGACGAAGACCTCCTCCAGACAGTAAGA
ATCATCAAGTTCCTCCATCAAAACAGTAAGTAGTACATGTAATGCAACCTTTAAATATAGTGGCAATAGTAGC
ATTAGTAGTAGCAGCAATAATAGCAATAGTTGTGTGGACCATAGTAGGCATAGAATATAGGAAAATATTAAGA
CAAAGGAAAATAGATAGGTTAATTGATAGGATAAGAGAAAGAGCAGAAGACAGTGGCAATGAAAGCGAAGGAG
ACCAGGAAGAATTATCAGCACTTGTGGAAATGGGGCACGATGCTCCTTGGGATGTTGCTGATCTGTAGTGCTG
CAGAACAATTGTGGGTCACAGTCTATTATGGGGTACCTGTGTGGAAAGACGCAAATACCACTCTATTTTGTGC
ATCAGATGCTAAAGCATATGAGAGAGAGGCACATAATGTTTGGGCCACACATGCCTGTGTACCCACAGACCCC
GACCCACAAGAAGTAGAATTGGCAAATGTGACAGAGGAATTTAACATGTGGGAAAACGACATGGTAGAACAGA
TGCATGAAGATATAATTAGTTTATGGGATCAAAGCCTAAAGCCATGTGTAAAATTAACCCCGCTCTGTGTCAC
TTTAAATTGCACTGATCTGGGGGATGTTAATACTACTACCCCTAATATTACTACTACTAATGAGACAATAAAG
GGAGAAATGAAAAACTGCTCTTTCAATATTACCACAAGCATAAGAGATAAGGTGCAGAAAGAATATGCACTTT
TTTATAAACTTGATATAGTAGAAATAAAGAATGATAATACTAACAATACTGACAGGACTAACTATACTAGCTA
TATAATGAGACATTGTAATGCCTCAGTCATTACACAGGCCTGTCCAAAGACATCCTTTGAGCCAATTCCAATA
CATTATTGTGCCCCGGCTGGTTTTGCGATTCTAAAGTGTAACAATAAGACATTCGATGGAAAAGGGAACTGTA
CAAATGTCAGCATAGTACAATGTACACATGGAATTAAGCCAGTAGTGTCAACTCAGCTGCTGTTAAATGGCAG
TTTAGCAGAAGAAAAGGTGGTAATTAGATCTGTCAATTTCTCAAACAATGCTAAAACCATAATAGTACAGCTG
AACACATCTGTAGGAATTAACTGTACAAGACCAAACAACAACACAGGAAAAACTATACACCTGGGATGGAGGA
GATCATTTTTTACAACAGAGACCATAATAGGAGATATAAGACGAGCACATTGTAATATTAGTAGAACAGCATG
GAATAACACTTTAAGACAGATAGCTGGAGAGTTAAGAAAACAATTTGGGAATAAAACAATAGCCTTTAATCGC
TCCTCAGGAGGGGACCCAGAGATTGTAATGCACAGTTTTAATTGTGGAGGGGAATTTTTCTACTGTGATACAA
CACAACTGTTTAATAGTACTTGGAATGGGACCGACATTTACAGCCATCGGAATGACACTGACATTAACAGAAA
TACCACAAATAATAAGACAGAGGTCATCACACTCCCATGCAGAATAAAACAAATTGTAAACATGTGGCAGGGA
ATAGGAAAAGCAATGTATGCCCCTCCCATCAGAGGACGAATTTATTGTGTATCAAATATTACAGGGCTGCTAT
TAACAAGAGATGGTGGTAATCAGAGTGGGAGCAACACCACCGAGACCTTCAGACCTCAAGGGGGAGACATGAA
GGACAATTGGAGGAGTGAATTATATAAATATAAAGTAGTACAACTTGAACCATTAGGAGTAGCACCCACCAAG
GCAAAGAGAAGAGTGGTGCAGAGAGAAAAAAGAGCAGTGGGAATGCTAGGAGCCATGTTCCTTGGGTTCTTGG
GAGCAGCAGGAAGCACTATGGGCGCAGCGTCAATGACGCTGACGGTACAGGCCAGACAATTAATGTCTGGTAT
AGTGCAACAGCAGAGCAATTTGCTGAGGGCTATTGAGGCGCAACAGCGCATGTTGCAACTCACAGTCTGGGGC
ATCAAGCAGCTCCAGGCAAGAGTACTGGCTGTGGAAAGATACCTACAGGATCAACGGCTCCTAGGGATTTGGG
GTTGCTCTGGAAAGCTCATCTGCACCACTGCTGTGCCTTGGAATAGTAGTTGGAGTAATAAAAATCTGACTCA
GATTTGGGATAACATGACCTGGATGCAGTGGGAAAAAGAAATTAACAATTACACAGGAGTAATATACAACTTA
CTTGAAGAATCGCAACACCAACAAGAAAAGAATGAACAAGAATTGTTGGAATTAGATAAGTGGGACAGTTTAT
GGAATTGGTTTGACATAACACAGTGGCTGTGGTATATAAAAATATTCATAATGATAGTAGGAGGCTTGATAGG
TTTAAGAATAATTTTTGCTGTGCTTTCTATAGTGAATAAAGTTAGGCAGGGATATTCACCATTATCATTTCAG
ACCCTCCTCCCGGTCCCGAGGGGACCCGACGGCCCGAAGGAACAGAAGAAGAAGGTGGAGAGAGAGACAGAG

GCAGATCAAACAGATTAGCAACTGGATTCTTGACAATTTTCTGGGAAGACCTACGGAACCTGTGCCTCTTCCT
CTACCACCGCTTGAGAGACTTACTCTTGATTGTAGGGAGGATTGTGGAAATTCTGGGACGCAGGGGGTGGGAG
ATCCTCAAATATTGGTGGAACCTCCTGCAATATTGGAGTCAGGAACTAAAGAATAGTGCTGTTAGCTTGCTCA
ATGCCACAGCTATTGCAGTAGCTGAGGGAACAGATAGGGTTATAGAAGTAGTGCAAAGAGCTTTTAGAGCTAT
TCTCCATATACCTACAAGAATAAGACAGGGCTTGGAAAGGGCTTTGCTATAAGATGGGTGGTAAGTGGTCAAA
ACATCTGAGTGGGTGGCCTAAGGTAAGGGAAAGAATAGAAAGAACTGAGCCAGCAGCAGATGGGGTGGGAGCA
GTTTCTCGAGACCTGGAAAAACATGGAGCAATCACAAGTAGCAATACAGCAACCACTAATGCTGCCTGTGCCT
GGCTAGAAGCACAAGAAGAAGAGGAGGTGGGTTTTCCAGTCAGACCTCAGGTACCTTTAAGACCCATGACTTA
CAAGGGAGCTTTAGATCTTAGCCACTTTTTAAGAGAAAAGGGGGGACTGGAAGGGTTAATTTACTCCCAGAAA
AGACAAGAGATCCTTGATCTGTGGGTCTACCACACACAAGGCTACTTCCCTGATTGGCAGAACTACACACCAG
GGCCAGGGATCAGATATCCCCTGACCTTTGGATGGTGCTTCAAGTTAGTACCAGTTGAGCCAGACGAGGAAGA
GAACAGCAGCCTGCTACACCCTATGAGCCAGCATGGAATGGGGGACACGGAGAAAGAAGTGTTAAAGTGGAAG
TTTGACAGCCACCTAGCATTCCGTCACATGGCCCGAGAGCTGCATCCGGAGTATTACAAAGACTGCTGACACC
GAGCTTTCTGCAAGGGACTTTCCGCTGGGGACTTTCCAGGGAGGCGTGGCCTGGGCGGGACTGGGGAGTGGCG
AGCCCTCAGATGCTGCATATAAGCAGCTGCTTTTTGCCTGTACTGGGTCTCTCTGGTTAGCCCAGATCCGAGC
CTGGGAGCTCTCTGGCTAACTAGGGAACCCACTGCTTAAGCCTCAATAAAGCTTGCCT
>AB-13A HIV-1 genome, derived from RNA genomic sequence
ACGCAGGACTCGGCTTGCTGAAGCGCGCACGGCAAGAGGCGAGGGGCGGCGACTGGTGAGTACGCCAATTTTT
GACTAGCGGAGGCTAGAAGGAGAGAGATGGGTGCGAGAGCGTCAGTATTAAGTGCGGGGGAATTAGATACATG
GGAAAAAATTCGGTTAAGGCCAGGAGGAAAGAAAAAATATAGATTAAAACATATAGTATGGGCAAGCAGGGAG
CTAGAACGATTTGCAGTTAATCCTGGCCTGTTAGAAACATCAGCAGGATGTAGACAAATACTGGGACAGCTAC
ATCCATCCCTTCAGACAGGATCAGAAGAACTTAGGTCATTATATAATACAGTAGCAACCCTCTATTGTGTACA
TCAAAAGATAGAGGTAAAAGACACCAAGGAAGCTTTAGAGAAGGTAGAGGAAGAGCAAAACAAAAGTAAGAAA
AAAGCACAGCAAGCGGCAGCTGACACAGGAAACAGCAGCCCGGTCAGCCAAAATTACCCTATAGTACAGAACC
TCCAGGGGCAAATGGTACACCAGACCATGTCACCTAGAACTTTAAATGCATGGGTAAAAGTGATAGAAGAGAA
GGCTTTCAGCCCAGAAGTAATACCCATGTTTTCAGCATTATCAGAAGGAGCCACCCCACAAGATTTAAACACC
ATGCTAAACACAGTGGGGGGACATCAAGCAGCCATGCAAATGTTAAAAGATACCATCAATGAGGAAGCTGCAG
AATGGGATAGATTGCATCCAGTGCATGCAGGGCCTATTGCACCAGGCCAGATGAGAGAACCAAGGGGAAGTGA
CATAGCAGGAACTACTAGTACCCTTCAGGAACAAATAGGATGGATGACACATAATCCACCTATCCCAGTAGGA
GAAATCTATAAGAGATGGATAATCCTGGGATTAAATAAAATAGTAAGAATGTATAGCCCTACCAGCATTCTGG
ACATAAAACAAGGACCAAAAGAACCCTTTAGAGATTATGTAGACCGGTTCTATAAAACTCTAAGAGCCGAGCA
AGCTTCACAGGATGTAAAAAATTGGATGACAGAAACCTTGTTGGTCCAAAATGCGAATCCAGATTGTAAGACC
ATTTTAAAAGCATTGGGACCAGCAGCTACATTAGAAGAAATGATGACAGCATGTCAGGGAGTGGGAGGACCCA
GCCATAAAGCAAGAGTCTTGGCTGAAGCAATGAGCCAAGCAACAGGTTCAGCTAACATAATGATGCAGAGAGG
CAATTTCAGGAACCAAAGAAAGACTGTTAAGTGTTTCAATTGTGGCAAAGAGGGGCACATAGCCAGAAATTGC
AGGGCCCCTAGGAAAAAGGGCTGTTGGAAATGTGGAAAGGAAGGACACCAAATGAAGGATTGCACTGAGAGAC
AGGCTAATTTTTTAGGGAAAATCTGGCCTTCCCACAAGGGGAGGCCAGGGAATTTTCTTCAGAGCAGACCAGA
ACCATCAGCCCCACCAGAAGAGAGCTTCAGGTTTGGGGAAGCAGCAACAACTCCCTCTCAGAAACAGGAGACG
ATAGACAAGGAACTGTATCCTTTAACCTCCCTCAAATCACTCTTTGGCAACGACCCATCGTCACAGTAAGGGT
AGGGGGGCAACTAATAGAAGCTCTATTAGATACAGGAGCAGATGATACAGTATTAGAAGAAATAAATTTACCA
GGAAGATGGAAACCAAAAATGATAGGGGGAATTGGAGGTTTTGTCAAAGTAAGACAGTATGATCAGGTACCCA
TAGAAATCTGTGGGCATAAAGTTATAGGTACAGTATTAGTAGGACCTACACCTACCAACGTAATTGGAAGAAA
TGTGATGACTCAAATTGGTTGCACTTTAAATTTTCCCATTAGTCCTATTGAAACTGTACCAGTAAAATTAAAG
CCAGGAATGGATGGCCCAAAAGTTAAACAATGGCCATTGACAGAAGAAAAAATAAAAGCATTAGTAGAAATTT
GTACAGAATTGGAAAAGGAAGGAAAAATTTCAAAAATTGGGCCTGAAAATCCATACAATACTCCAGTATTTGC
CATAAAGAAAAAAGACAGTACTAAATGGAGAAAATTGGTAGATTTCAGAGAACTTAATAAGAGAACTCAAGAC
TTCTGGGAAGTTCAATTGGGAATACCACATCCCGCAGGGTTAAAAAAGAAAAAATCAGTAACAGTCTTGGATG
TGGGTGATGCATATTTTTCAGTTCCCTTGGATGAAGACTTTAGAAAGTATACTGCATTCACCATACCTAGTAC
AAACAATGAGACACCAGGGACTAGATATCAGTACAATGTGCTGCCACAGGGATGGAAAGGATCACCAGCAATA
TTCCAAAGTAGCATGACAAAAATCTTAGAGCCTTTTAGAAAACAAAATCCAGACATAGTTATCTATCAATACG
TGGATGATTTGTATGTAGGATCAGACTTAGAAATAGGGCAGCATAGGATAAAAATAGAGGAACTGAGAGAACA
TCTGTTGAGGTGGGGATTTTACACACCAGACAAAAAACATCAGAAAGAACCTCCATTCCTCTGGATGGGTTAT
GAACTCCATCCTGATAAATGGACAGTACAGCCTATAGTGCTGCCAGAAAAAGACAGCTGGACTGTCAATGACA
TACAGAAGTTAGTGGGAAAATTGAATTGGGCAAGTCAGATTTATCCAGGGATTAAAGTAAAGCAATTATGTAA

GCTCCTTAGGGGAACCAAGGCACTAACAGAGGTAGTACCACTAACAGAGGAAGCAGAGCTAGAACTAGCAGAA
AACAGGGAAATTCTAAAAGAACCAGTACATGGAGTGTATTATGACCCATCAAAAGACTTAGTAGCAGAAATAC
AGAAGCAGGGACAAGGCCAATGGACATATCAAATTTATCAAGAGCAAGGTAAAAATTTGAAAACAGGAAAGTA
TGCAAGAATGAGGGGTGCCCACACTAATGATATAAAACAGTTAACAGAGGCAGTGCAAAAAATAAGCATGGAA
AGCATAGTAATATGGGGAAAGACTCCTAAATTTAAACTACCCATACAAAAGGAAACATGGGAAGCATGGTGGA
TGGAGTATTGGCAAGCCACCTGGATTCCTGAGTGGGAGTTTGTCAATACCCCTCCCTTAGTAAAATTATGGTA
TCAGTTAGAGAAAGAACCCATAGTAGGAGCAGAAACTTTCTATGTAGATGGGGCAGCTAATAGGGAAACTAAA
TTAGGAAAAGCAGGATATGTTACTGACAGAGGAAGACAAAAAGTTGTCTCCCTGACTGACACAACAAATCAGA
AGACTGAGTTACAAGCAATTCATCTAGCCTTGCAGGATTCGGGATTAGAAGTAAACATAGTAACAGACTCACA
ATATGCATTAGGAATCATTCAAGCACAACCAGATCAAAGTGAATCAGAGTTAGTCAATCAAATAATAGAGCAG
TTAATAAAAAAGGAAAAAATCTACTTGGCATGGGTACCAGCACATAAAGGGATTGGAGGAAATGAGCAGGTAG
ATAAATTAGTCAGTACTGGAGTCAGGAAAGTACTATTTTTAGATGGAATAGATAAGGCCCAAGAAGAACATGA
GAAATATCACAGTAATTGGAGAGCAATGGCTAGTGATTTTAACCTGCCACCTGTAATAGCAAAAGAAATAGTA
GCCTGCTGTGATAAATGTCAGCTCAAAGGAGAAGCCATGCATGGACAAGTAGACTGTAGTCCAGGAATATGGC
AACTAGATTGTACACATTTAGAAGGAAAAATTATCATAGTAGCAGTTCATGTAGCCAGTGGATATGTAGAAGC
AGAAGTCATTCCAGCAGAGACAGGACAGGAAACAGCATACTTTCTCTTAAAATTAGCAGGAAGATGGCCAGTA
AAAACAATACATACAGACAATGGCAGCAATTTCACCAGTAATGTGGTTAAGGCTGCCTGTTGGTGGGCAGGGA
TCAAGCAGGAATTTGGCATTCCCTACAATCCCCAAAGTCAAGGAGTAGTAGAATCCATGAATAAAGAATTAAA
GAAAATTATAGGACAGGTAAGAGATCAGGCTGAACATCTTAAGACAGCAGTACAAATGGCAGTATTCATCCAC
AATTTTAAGAGAAAAGGGGGGATTGGGGGGTACAGTGCAGGGGAAAGAATAGTAGACATAATAGCAACAGACA
TACAAACTAAAGAGTTACAAAAACAAATTACAAAAATTCAAAATTTTCGGGTTTATTACAGGGACAGCAGAGA
TCCACTTTGGAAAGGACCAGCAAAGCTTCTCTGGAAAGGTGAAGGGGCAGTAGTAATACAAGATAATAGTGAT
ATAAAAGTAGTGCCAAGAAGAAAAGCAAAGATCATTAGGGATTATGGAAAACAGATGGCAGGTGATGATTGTG
TGGCAAGTAGACAGGATGAGGATTAGAACATGGAAAAGTTTAGTAAAACACCATATGTATGTCTCACGGAAAG
CTGGGAAATGGTTTTATAGACATCACTATGAAAGCACTCATCCAAAAATAAGTTCAGAAGTACACATCCCAAT
AGGGGATGCTAGATTGGTAATAACAACATATTGGGGTCTGCAAACAGGAGAAAGAGACTGGCAATTGGGCCAT
GGAGTCTCCATAGAATGGAGGAAAGGGAGATATAGCACACAAGTAGACCCTGATCTAGCAGACCAACTAATCC
ATCTGTATTATTTTGATTGTTTTTCAGAATCTGCTATAAGACATGCCATATTAGGACATATAGTTAGACCTAG
TTGTGAATATCAAGCAGGACATAACAAGGTAGGATCCCTACAATACTTGGCACTAGCAGCATTAATAAAACCA
AAGAAGATAAAGCCACCTTTGCCTAGTGTTAAGAAACTGACAGAGGATAGATGGAACAAGCCCCGGAAGACCA
AGGGCCACAGAGGGAGCCATACAATGAATGGGCACTAGAGCTTTTAGAGGAGCTTAAGAGTGAAGCTGTTAGA
CATTTTCCTAGGGCATGGCTACATAGCTTAGGACAATATATCTATGAAACTTATGGGGACACTTGGGCAGGAG
TGGAGGCCATAATAAGAATACTGCAACAACTGCTGTTTATTCATTTCAGAATTGGGTGTCACCATAGCAGAAT
AGGCATTATTCGACAGAGGAGAGCAAGAAATGGAGCCAGTAGATCCTAGACTAGAGCCCTGGAAGCATCCAGG
AAGTCAGCCTAGGACTCCTTGTACCAATTGCTATTGTAAAAAGTGTTGCCTTCATTGCCAAGTTTGTTTTATG
AAAAAAGGCTTAGGCATCTCCTATGGCAGGAAGAAGCGGAGACAGCGACGAAGACCTCCTCCAGACAGTAAGA
ATCATCAAGTTCCTCTATCAAAGCAGTAAGTAGTACATGTAATGCAACCTTTAAATACAGTAGCAATAGTAGC
ATTAGTAGTAGCAGCAATAATAGCAATAGTTGTGTGGACCATAGTAGGCATAGAATATAGGAAAATATTAAGA
CAAAGAAAAATAGATAGGTTAATTGATAGAATAAGAGAAAGAGCAGAAGACAGTGGCAATGAAAGCGAAGGAG
ACCAGGAAGAATTATCAGCACTTGTGGAAATGGGGCACGATGCTCCTTGGGATGTTGATGATCTGTAGTGCTG
CAGAACAATTGTGGGTCACAGTCTATTATGGGGTACCTGTGTGGAAAGACGCAAATACCACTCTATTTTGTGC
ATCAGATGCTAAAGCATATGAGACAGAGGCACATAATGTTTGGGCCACACATGCCTGTGTACCCACAGACCCC
AACCCACAAGAAGTAGTATTGGAAAATGTGACAGAGGAATTTAATATGTGGGAAAACAACATGGTAGAACAGA
TGCATGAAGATATAATCAGTTTATGGGATCAAAGCCTAAAGCCATGTGTAAAATTAACCCCGCTCTGTGTCAC
TTTAAATTGCACTGATCTGGGGAATGTTACTAATACCACTAATAGTACTACTAATGAGACAATAAAGGAAGAA
ATGAAAAACTGCTCTTTCAATATTACCACAAGCATAAGAGATAAAGTGCAGAAAGAATATGCACTTTTTTATA
AACTTGATATAGTAGAAATAAAGGATGATAATACTAACAATACTGACAATACTAGCTATATAATGAGACATTG
TAATGCCTCAGTCATTACACAGGCCTGTCCAAAGACATCCTTTGAGCCAATTCCCATACATTATTGTGCCCCG
GCTGGTTTTGCGATTCTAAAGTGTAACAATAAGACATTCGATGGAAAAGGGAACTGTACAAATGTCAGCACAG
TACAATGTACACATGGAATTAAGCCAGTAGTGTCAACTCAGCTGCTGTTAAATGGCAGTTTAGCAGAAGAAGA
GGTGGTAATTAGATCTGTCAATTTCTCAAACAATGCTAAAACCATAATAGTACAGCTGAACAAATCTGTAGAA
ATTAACTGTACAAGACCCAACAACAATACAGGAAAAACTATACATATGGGATGGAGGAGATCATTTTATACAA
CAGAGACCATAATAGGAGATATAAGACGAGCACATTGTAACATTAGTAGAACAGCATGGAATAACACTTTAAG
ACAGATAGCTGGAGAATTAGAAAACAATTTGGGAATAAACCAATAGTCTTTAATCACTCCTCAGGAGGGGAC

CCAGAAATTGTAATGCATAGTTTTAATTGTGGAGGGGAATTTTTCTACTGTGATACAACACAACTGTTTAATA
GTACTTGGAATGAAACCGACATTAACAGAAATAACACAAGTGAAGGAAATAAGACAGATGTCATCACACTCCC
ATGCAGAATAAAACAAATTATAAACATGTGGCAGGGAATAGGAAAAGCAATGTATGCCCCTCCCATCAGAGGA
CGAATTAATTGTGTATCAAATATTACAGGGCTGCTATTAACAAGAGATGGTGGTAATCAGAGTGAACAGCGCA
GCGAAACCTTCAGACCTCAAGGGGGAGACATGAAGGATAATTGGAGAAGCGAATTATATAAATATAAAGTAGT
ACAACTTGAACCATTAGGAGTAGCACCCACCAAGGCAAAGAGAAGAGTGGTGCAGAGAGAAAAAAGAGCAGTG
GGACTGTTAGGAGCCATGTTCCTTGGGTTCTTGGGAGCAGCAGGAAGCACTATGGGCGCAGCGTCAATGACGC
TGACGGTACAGGCCAGACAATTAATGTCTGGTATAGTGCAACAGCAGAGCAATTTGCTGAGGGCTATTGAGGC
GCAACAGCGTATGTTGCAACTCACAGTCTGGGGCATCAAGCAGCTCCAGGCGAGAGTACTAGCTGTGGAAAGA
TACCTACAGGATCAACGGCTCCTAGGGATTTGGGGATGCTCTGGAAAGCTCATCTGCACCACTGCTGTGCCTT
GGAATAGTAGTTGGAGTAATAAAAATCTGACTCAGATTTGGGATAACATGACCTGGATGCAGTGGGAAAAAGA
AATTAACAATTACACAGGAGTAATATACAACTTACTTGAAAAATCGCAAAACCAACAAGAAAAGAATGAACAA
GAATTGTTGGAATTAGATAAGTGGGACAGTTTATGGAATTGGTTTGACATAACAAAATGGCTGTGGTATATAA
AAATATTCATAATGATAGTAGGAGGCTTGATAGGTTTAAGAATAATTTTTGCTGTGCTTTCTATAGTGAATAA
AGTTAGGCAGGGATACTCACCATTATCATTTCAGACCCTCCTCCCAGTCCCGAGGGGACCCGACGGCCCGAA
GGAACAGAAGAAGAAGGTGGAGAGAGAGACAGAGGCAGATCAAATCGATTAGCAACTGGATTCTTGACAATTT
TCTGGGAAGACCTACGAAGCCTGTGCCTCTTCCTCTACCACCGCTTGAGAGACTTACTCTTGATTGTAGGGAG
GATTGTGGAAATTCTGGGACGCAGGGGGTGGGAGATCCTCAAATATTGGTGGAATCTCCTGCAATATTGGAGT
CAGGAACTAAAGAATAGTGCTGTTAGCTTGCTCAATGCCACAGCTATTGCAGTAGCTGAGGGAACAGATAGGG
TTATAGAAGTAGTGCAAAGAGCTTTTAGAGCTATTCTCCATATACCTACAAGAATAAGACAGGGCTTGGAAAG
GGCTTTGCTATAAGATGGGTGGCAAGTGGTCAAAACATAATGGATGGCCTAAGGTAAGGGAAAGAATAGAAAG
AACTGAGCCAGCAGCAGATGGGGTGGGAGCAGTTTCTCGAGACCTGGAAAAACATGGAGCAATCACAAGTAGC
AATACAGCAACTAATAATGCTGCCTGTGCCTGGCTAGAAGCACAAGAGGAAGAGGAGGTGGGTTTTCCAGTCA
GACCTCAGGTACCTTTAAGACCAATGACTTACAAGGGAGCTTTAGATCTTAGCCACTTTTTAAGAGAAAAGGG
GGGACTGGAAGGGTTAATTTACTCCCAGAAAAGACAAGAGATCCTTGATCTGTGGGTCTACCACACACAAGGC
TACTTCCCTGATTGGCAGAACTACACACCAGGGCCAGGGATCAGATATCCCCTGACCTTTGGATGGTGCTTCA
AGTTAGTACCAGTTGAGCCAGACGAGGAAGAGAACAGCAGCCTGCTACACCCTATGAGCCAGCATGGAATGGA
GGACACGGAGAAAGAAGTGTTAAAGTGGAAGTTTGACAGCCACCTAGCATTTCGTCACATGGCCCGAGAGCTG
CATCCGGAGTATTACAAAGACTGCTGACACCGAGTTTTCTACAAGGGACTTTCCGCTGGGGACTTTCCGGGGA
GGCGTGGCCTGGGCGGGACTGGGGAGTGGCGAGCCCTCAGATGCTGCATATAAGCAGCTGCTTTTTGCCTGTA
CTGGGTCTCTCTGGTTAGACC

>AB-21A HIV-1 genome, derived from RNA genomic sequence
ACGCAGGACTCGGCTTGCTGAAGCGCGCACGGCAAGAGGCGAGGGGCGGCGACTGGTGAGTACGCCAAATTTT
GACTAGCGGAGGCTAGAAGGAGAGAGATGGGTGCGAGAGCGTCAGTATTAAGTGCGGGGGAATTAGATACATG
GGAAAAAATTCGGTTAAGGCCAGGGGGAAAGAAAAAATATAGATTAAAACATATAGTATGGGCAAGCAGGGAG
CTAGAACGATTCGCAGTTAATCCTGGCCTGTTAGAAACATCAGCAGGCTGTAGACAAATACTGGGACAGCTAC
ATCCATCCCTTCAGACAGGATCAGAAGAACTTAGATCATTATATAATACAGTAGCAACCCTCTATTGTGTGCA
TCAAAAGATAGAGGTAAAAGACACCAAGGAAGCTTTAGAGAAGATAGAGGAAGAGCAAAACAAAAGTAAGAAA
AAAGCACAGCAAGCAGCAGCTGACACAGGAAACAGCGGCCAGGTCAGCCAAAATTACCCTATAGTACAGAACC
TCCAGGGGCAAATGGTACATCAGACCATGTCACCTAGAACTTTAAATGCATGGGTAAAAGTAATAGAAGAGAA
GGCTTTCAGCCCAGAAGTAATACCCATGTTTTCAGCATTATCAGAAGGAGCCACCCCACAAGATTTAAACACC
ATGCTAAACACAGTGGGGGGACATCAAGCAGCCATGCAAATGTTAAAAGATACCATCAATGAGGAAGCTGCAG
AATGGGATAGATTGCATCCAGTGCATGCAGGGCCTATTGCACCAGGCCAGATGAGAGAACCAAGGGGAAGTGA
CATAGCAGGAACTACTAGTACCCTTCAGGAACAAATAGGATGGATGACACACAATCCACCTATCCCAGTAGGA
GAAATCTATAAGAGATGGATAATCCTGGGGTTAAATAAAATAGTAAGAATGTATAGCCCTACTAGCATTCTGG
ACATAAAACAAGGACCAAAAGAACCCTTTAGAGATTATGTAGACCGGTTCTATAAAACTCTAAGAGCCGAGCA
AGCTTCACAGGATGTAAAAAATTGGATGACAGAAACCTTGTTGGTCCAAAATGCAAATCCAGATTGTAAGACT
ATTTTAAAAGCATTAGGACCAGCAGCTACATTAGAAGAAATGATGACAGCATGTCAGGGAGTGGGAGGACCCA
GCCATAAAGCAAGAGTCTTGGCTGAAGCAATGAGCCAAGCAACAGGTTCAGCTAACATAATGATGCAGAGAGG
TAATTTTAGGAACCAAAGAAAGACTGTTAAGTGTTTCAATTGTGGCAAAGAGGGGCACATAGCCAGAAATTGC
AGAGCCCCTAGGAAAAAGGGCTGTTGGAAATGTGGAAAGGAAGGACACCAAATGAAGGATTGCACTGAGAGGC
AGGCTAATTTTTTAGGGAAAATCTGGCCTTCCCACAAGGGAAGGCCAGGGAATTTTCTTCAGAGCAGACCAGA
GCCAACAGCCCCACCAGAAGAGAGCTTCAGGTTTGGGGAAGCAACAACTCCCTCTCAGAAGCAGGAGACGATA
GACAAGGAAATGTATCCTTTAACCTCCCTCAAATCACTCTTTGGCAACGACCCCTCGTCACAGTAAGGATAGG
GGGGCAACTAAAGGAAGCTCTATTAGATACAGGAGCAGATGATACAGTATTAGAAGAAATGAATTTGCCAGGA
AGGTGGAAACCAAAAATGATAGGGGGAATTGGAGGTTTTATCAAAGTAAGACAGTATGATCAGATACTCGTAG
AAATCTGTGGACATAAAGCTATAGGTACAGTATTAGTAGGACCTACACCTGTCAACATAATTGGAAGAAATCT
GTTGACTCAGATTGGTTGCACTTTAAATTTTCCCATTAGTCCTATTGAAACTGTACCAGTAAAATTAAAGCCA
GGAATGGATGGCCCAAAAGTTAAACAATGGCCATTGACAGAAGAAAAAATAAAAGCATTAGTAGAAATTTGTA
CAGAAATGGAAAAGGAAGGGAAAATTTCAAAAATTGGGCCTGAAAATCCATACAATACTCCAGTGTTTGCCAT
AAAGAAAAAAGACAGTACTAAATGGAGAAAATTGGTAGATTTCAGAGAACTTAATAAGAGAACTCAAGACTTC
TGGGAAGTTCAATTAGGAATACCACATCCCGCAGGGTTAAAAAAGAAAAAATCAGTAACAGTCCTGGATGTGG
GTGATGCATATTTTTCAGTTCCCTTAGATGAAGACTTTAGAAAGTATACTGCATTCACCATACCTAGTACAAA
CAATGAAACACCAGGGACTAGATATCAGTACAATGTGCTGCCACAGGGATGGAAAGGATCACCAGCAATATTC
CAAAGTAGCATGACAAAAATCTTAGAGCCTTTTAGAAAACAAAATCCAGACATAGTTATCTATCAATACATGG
ATGATTTGTATGTAGGATCCGACTTAGAAATAGGGCAGCATAGAATAAAAATAGAGGAACTGAGAGAACATCT
GTTGAGGTGGGGATTTACCACACCAGACAAAAAACATCAGAAAGAACCTCCATTCCTCTGGATGGGTTATGAA
CTCCATCCTGATAAATGGACAGTGCAGCCTATAGTGCTGCCAGAAAAAGACAGCTGGACTGTCAATGACATAC
AGAAGTTAGTGGGAAAATTGAACTGGGCCAGTCAGATTTATCCAGGGGTTAAAGTAAAGCAATTATGTAAGCT
CCTTAGGGGAACCAAAGCACTAACAGAAGTAATACCACTAACAGAAGAAGCAGAGCTAGAACTAGCAGAAAAC
AGGGAGATTCTAAAAGAACCAGTACATGGAGTGTATTATGACCCATCAAAAGACTTAGTAGCAGAAATACAGA
AGCAGGGGCAAGGCCAATGGACATATCAAATTTATCAAGAGCCATGTAAAAATCTGAAAACAGGAAAGTATGC
AAGAATGAGGGGTGCCCACACTAATGATATAAAACAGTTAACAGAGGCAGTGCAAAAAATAACCATGGAAAGC
ATAATAATATGGGGAAAAACTCCTAAATTTAGACTACCCATACAAAAGGAAACATGGGAAGCATGGTGGATGG
AGTATTGGCAAGCCACCTGGATTCCTGAGTGGGAGTTTGTCAATACCCCTCCCTTAGTAAAATTATGGTACCA
GTTAGAGAAAGAACCCATAGAGGGAGCAGAAACTTTTTATGTAGATGGGGCAGCTAATAGGGAGACTAAATTA
GGAAAAGCAGGATATGTTACTGACAGAGGAAGACAAAAAGTTGTCTCCCTAACTGACACAACAAATCAGAAGA
CTGAGTTACAAGCAATTCATCTAGCGTTGCAGGATTCGGGACTAGAAGTGAACATTGTAACAGACTCACAATA
TGCATTAGGAATCATTCAAGCACAACCAGATCAAAGTGAATCAGAGTTAGTTAATCAAATAATAGAGCAGTTA
ATAAAAAAGGAAAAAATCTACCTGGCATGGGTACCAGCACATAAAGGAATTGGAGGAAATGAGCAGGTAGATA
AATTAGTCAGTACTGGAATCAGGAAAGTACTATTTTTAGATGGAATAGATAAGGCCCAAGAAGAACATGAGAG
ATATCACAGTAATTGGAGAGCAATGGCTAGTGATTTTAACCTGCCACCTGTAATAGCAAAAGAAATAGTAGCC
TGCTGTGATAAATGTCAGCTAAAAGGAGAAGCCATGCATGGACAAGTAGACTGTAGTCCAGGAATATGGCAAC
TAGATTGTACACATTTAGAAGGAAAAATTATCATAGTAGCAGTTCATGTAGCCAGTGGATATATAGAAGCAGA
AGTCATTCCAGCAGAGACAGGACAGGAAACAGCATACTTTCTCTTAAAATTAGCAGGAAGATGGCCAGTAAAA

GCAATACATACAGACAATGGCAGCAATTTCACCAGTAATGTGGTTAAGGCTGCCTGTTGGTGGGCAGGGATCA
AGCAGGAATTTGGCATTCCCTACAATCCCCAAAGTCAAGGAGTAGTAGAATCTATGAATAAAGAATTAAAGAA
AATTATAGGACAGGTAAGAGATCAGGCTGAACATCTTAAGACAGCAGTACAAATGGCAGTATTCATCCACAAT
TTTAAGAGAAAAGGGGGGATTGGGGGGTACAGTGCAGGGGAAAGAATAGTAGACATAATAGCAACAGACATAC
AAACTAAAGAATTACAAAAACAAATTACAAAAATTCAAAATTTTCGGGTTTATTACAGGGACAGCAGAGATCC
ACTTTGGAAAGGACCAGCAAAGCTTCTCTGGAAAGGTGAAGGGGCAGTAGTAATACAAGATAATAGTGATATA
AAAGTAGTGCCAAGAAGAAAAGCAAAGATCATTAGGGATTATGGAAAACAGATGGCAGGTGATGATTGTGTGG
CAAGTAGACAGGATGAGGATTAGAACATGGAAAAGTTTAGTAAAACACCATATGTATGTTTCACGGAAAGCTG
GGAAATGGTTTTATAGACATCACTATGAAAGCACTCATCCAAAAATAAGTTCAGAAGTACACATCCCACTAGG
GGATGCTAGATTGGTAGTAACAACATATTGGGGTCTGCAAACAGGAGAAAGAGACTGGCAGTTGGGCCATGGA
GTCTCCATAGAATGGAGGAAAAGGAAATATAGCACACAAGTAGACCCTGATCTAGCAGACCAACTAATCCATC
TGTATTATTTTGATTGTTTTTCAGAATCTGCTATAAGACATGCCATATTAGGACATATAGTTAGACCTAGTTG
TGAATATCAAGCAGGACATAACAAGGTAGGATCCCTACAATACTTGGCACTAACAGCACTAATAAAACCAAAG
AAGATAAAGCCACCTTTGCCTAGTGTTAAGAAACTGACAGAGGATAGATGGAACAAGCCCCAGAAGACCAAGG
GCCACAGAGGGAGCCATACAATGAATGGACACTAGAGCTTTTAGAGGAGCTTAAGAGTGAAGCTGTTAGACAT
TTTCCTAGGGCATGGCTACATAGCTTAGGACAATATATCTATGAAACTTATGGGGATACTTGGGCAGGAGTGG
AAGCCATAATAAGAACGCTGCAACAACTGCTGTTTATTCATTTCAGAATTGGGTGTCGCCATAGCAGAATAGG
CATTATTCGACAGAGGAGAGCAAGAAATGGAGCCAGTAGATCCTAGACTAGAGCCCTGGAAGCATCCAGGAAG
TCAGCCTAGGACTCCTTGTACCAATTGCTATTGTAAAAAGTGTTGCCTTCATTGCCAAGTTTGTTTTATGAAA
AAAGGCTTAGGCATCTCCTATGGCAGGAAGAAGCGGAGACAGCGACGAAGACCTCCTCAAGACAGTGAAAATC
ATCAAGTTCCTCTATCAGAGCAGTAAGTAGTACATGTAATGCAACCTTTACATATAGTAGCAATAGTAGCATT
AGTAGTAGCAGCAATAATAGCAATAGTTGTGTGGACCATAGTAGGCATAGAATATAGGAAAATATTAAGACAA
AGAAAAATAGACAGGTTAATTGATAGAATAAGAGAAAGAGCAGAAGACAGTGGCAATGAAAGCGAAGGAGACC
AGGAAGAATTATCAGCACTTGTGGAGATGGGGCACCATGCTCCTTGGGATGTTGATGATCTGTAGTGCTGCAG
AACAATTGTGGGTCACAGTCTATTATGGGGTACCTGTGTGGAAAGAAGCAAATACCACTCTATTTTGTGCATC
AGATGCTAAAGCATATGATACAGAGGCACATAATGTTTGGGCCACACATGCCTGTGTACCCACAGACCCCAAC
CCACAAGAAGTAGTATTGGAAAATGTGACAGAAGAATTTAACATGTGGAAAAATAACATGGTAGAACAGATGC
ATGAAGATATAATCAGTCTATGGGATCAAAGCCTAAAGCCATGTGTAAAATTAACCCCACTCTGTGTTACTTT
AGACTGCACTGATTTGAGGAATGCTACTAATGCCACTAATAGTAATGGGACAATAAAGGAAGAAATGAGAAAC
TGCTCTTTCAATATCACCACAAGCATAAGAGATAAGGTGCAGAAAGAATATGCACTTTTTTATAGACTAGATA
TAGTACAAATAAAGGATGATAATACTAACAATACTAACAATACTAGCTATAGGATGATAAATTGTAATACCTC
AGTCATTACACAGGCCTGTCCAAAGATATCCTTTGAGCCAATTCCCATACATTATTGTGCCCCGGCTGGTTTT
GCGATTCTAAAGTGTAACAATAAGACGTTCGATGGAAAAGGACTGTGTAAAAATGTCAGCACAGTACAATGTA
CACATGGAATTAAGCCAGTAGTGTCAACTCAACTGCTGTTAAATGGCAGTCTAGCAGAAAAAGAGGTGGTAAT
TAGATCTGTCAATTTCTCAGACAATGCTCAAACCATAATAGTACAGCTGAACAAATCTGTAGAAATTAACTGT
ACAAGACCCAGCAACAATACAAGGAAAAGTATACATATAGGACCAGGGAGAGCATTTTATGCAACAGGAGACA
TAATAGGAGATATAAGACAAGCACATTGTAACATTAGTAGAGCAGAATGGAATAACACTTTAAGACAGATAGC
TAAAAAATTAAGAGAACAATTTGTGAATAAAACAATAATCTTTAATCACTCCTCAGGAGGGGACCCAGAAATT
GTAATGCACAGTTTTAATTGTGGAGGGGAATTTTTCTACTGTAATACAACACAACTGTTTAACAGTACTTGGA
ATAAAACCAACATTGACGGAAATGACACCACTAAAGGAGATAATATCACAGATGTCATCACACTCCCATGCAG
AATAAAACAAATTATAAACATGTGGCAGGGAGTAGGAAAAGCAATGTATGCCCCTCCCATCAGAGGACAAATT
AATTGTTTATCAAATATTACAGGGCTGCTATTAACAAGAGATGGTGGTAATCAGAGTGGGAGCAACGAGACCT
TCAGACCTGAAGGGGGAAATATGAAGGACAATTGGAGAAGTGAACTATATAAATATAAAGTAGTACAACTTGA
ACCATTAGGAGTAGCACCCACCAAGGCAAAGAGAAGAGTGGTGCAGAGAGAAAAAAGAGCAGTGGGAATGATA
GGAGCCATGTTCCTTGGGTTCTTGGGAGCAGCAGGAAGCACTATGGGCGCAGCGTCAATGACGCTGACGGTAC
AGGCCAGACAATTATTGTCTGGTATAGTGCAACAGCAGAGCAATTTGCTGAAGGCTATTGAGGCGCAACAGCA
TATGTTGCAACTCACAGTCTGGGGCATCAAGCAGCTCCAGGCAAGAGTACTGGCTGTGGAAAGATACCTACAG
GATCAACGGCTCCTAGGGATTTGGGGTTGCTCTGGAAAACTCATCTGCACCACTGCTGTGCCTTGGAATACTA
GCTGGAGTAGTAAAAATCTGACTCAGATTTGGGATAACATGACCTGGATGCAGTGGGAAAAAGAAATTAACAA
TTACACAGGAGTAATATACAACTTACTTGAAAAATCGCAGAACCAACAAGAAAAGAATGAACAAGAATTGTTG
GAATTAGATAAGTGGGACAGTTTGTGGAATTGGTTTGACATAACAAAATGGCTGTGGTATATAAAAATATTCA
TAATAATAGTAGGAGGCTTGGTAGGTTTGAGAATAGTTTTTGCTGTACTCTCTATAGTGAATAAAGTTAGGCA
GGGATACTCACCATTATCATTTCAGACCCTCCTCCCAGCCCCGAGGGGACCCGACAGGCCCGAAGGAATA?AA
GAAGAAGGTGGAGAGAGAGACAGAGGCAGATCAAATCGATTAGCAACTGGATTCTTGATACTTTTCTGGGAAG

ACCTGCGGAGCCTGTGCCTCTTCCTCTACCACCGCTTGAGAGACTTACTCTTGATTCTAGCGAGGATTGTGGA
AATTCTGGGACACAGGGGGTGGGAGATCCTCAAATATTGGTGGAATCTCCTGCAATATTGGAGTCAGGAACTA
AAGAATAGTGCTGTTAGCTTGCTCAATGCCACAGCTATCGCAGTAGCTGAGGGAACAGATAGGGTTATAGAAG
TAGTGCAAAGAGCTTTTAGAGCTATTCTCCACATACCTACAAGAGTGAGACAGGGCTTGGAAAGGGCTTTGCT
ATAAGATGGGTGGCAAGTGGTCAAAACGTAGTCTGGGTGGATGGCCTAATGTAAGGGAAAGAATGAGAAGAAC
TGAGCCAGCAGCAGAGGGGGTGGGAGCAGTATCTCGAGACTTGGAAAAACATGGAGCAATCACAAGTAGCAAT
ACAGCAACTAGTAATGCTGCCTGTGCCTGGCTAGAAGCACAAGAGGAAGAGGAGGTGGGTTTTCCAGTCAGAC
CTCAGGTACCTTTAAGACCAATGACTTACAAGGGAGCTTTAGATCTTAGCCACTTTTTAAGAGAAAAGGGGGG
ACTGGAAGGGTTAATTCACTCCCAGAAAAGACAAGAGATCCTTGATCTGTGGGTCTACCACACACAAGGTTAC
TTCCCTGATTGGCAGAACTACACACCAGGGCCAGGGATCAGATATCCCCTGACCTTTGGATGGTGCTTCAAGT
TAGTACCAGTTGAGCCAGACGAAGAAGAGAACAGCAGCTTGCTACACCCTATGAGCCAGCATGGGATGGAGGA
TACGGAGAAAGAAGTGTTAAAGTGGAAGTTTGACAGCCACCTAGCATTTCGTCACATGGCCCGAGAGCTGCAT
CCGGAGTATTACAAAGACTGCTGACACCGAGTTTTCTGCAAGGGACTTTCCGCTGGGGACTTTCCAGGGAGGC
GTGGCCTGGGCGGGACTGGGGAGTGGCGAGCCCTCAGATGCTGCATATAAGCAGCTGCTTTTTGCCTGTACTG
GGTCTCTCTGGTTAGACCAGATCAGAGCCTGGGAGCTCTCTGGCTAACTAGGGAACCCACTGCTTAAGCCTCA
ATAAAGCTTGCCT

>AB-21D HIV-1 genome, derived from RNA genomic sequence
ACGCAGGACTCGGCTTGCTGAAGCGCGCACGGCAAGAGGCGAGGGGCGGCGACTGGTGAGTACGCCAAATTTT
GACTAGCGGAGGCTAGAAGGAGAGAGATGGGTGCGAGAGCGTCAGTATTAAGTGCGGGGGAATTAGATACGTG
GGAAAAAATTCGGTTAAGGCCAGGGGGAAAGAAAAAATATAGATTAAAACATATAGTATGGGCAAGCAGGGAG
CTAGAACGATTTGCAGTTAATCCTGGCCTGTTAGAAACATCAGCAGGCTGTAGACAAATACTGGGACAGCTAC
ATCCATCCCTTCAGACAGGATCAGAAGAACTTAGATCATTATATAATACAGTAGCAACCCTCTATTGTGTGCA
TCAAAAGATAGAGGTAAAAGACACCAAGGAAGCTTTAGAGAAGATAGAGGAAGAACAAAACAAAAGTAAGAAA
AAGGCACAACAAGCAGCAGCTGACACAGGAAACAGCAGCCAGGTCAGCCAAAATTACCCCATAGTACAGAACC
TCCAGGGGCAAATGGTACATCAGACCATGTCACCTAGAACTTTAAATGCATGGGTAAAAGTAATAGAAGAGAA
GGCTTTCAGCCCAGAAGTAATACCCATGTTTTCAGCATTATCAGAAGGAGCCACCCCACAAGATTTAAACACC
ATGCTAAACACAGTGGGGGGACATCAAGCAGCCATGCAAATGTTAAAAGATACCATCAATGAGGAAGCTGCGG
AATGGGATAGATTGCATCCAGTGCATGCAGGGCCTATTGCACCAGGCCAGATGAGAGAACCAAGGGGAAGTGA
CATAGCAGGAACTACTAGTACCCTTCAGGAACAAATAGGATGGATGACACATAATCCACCTATCCCAGTAGGA
GAAATTTATAAGAGATGGATAATCCTGGGATTAAATAAAATAGTAAGAATGTATAGCCCTACCAGCATTCTGG
ACATAAAACAAGGACCAAAAGAACCCTTTAGAGATTATGTAGACCGGTTCTATAAAACTCTAAGAGCCGAGCA
AGCTTCACAGGATGTAAAAAATTGGATGACAGAAACCTTGTTGGTCCAAAATGCGAATCCAGATTGTAAGACT
ATTTTAAAAGCATTGGGACCAGCAGCTACATTAGAAGAAATGATGACAGCATGTCAGGGAGTGGGGGGACCCA
GCCATAAAGCAAGAGTCTTGGCTGAAGCAATGAGCCAAGCAACAGGTTCAGCTACCATAATGATGCAGAGAGG
CAATTTTAGGAACCAAAGAAAGACTGTTAAGTGTTTCAATTGTGGCAAAGAGGGGCACATAGCCAGAAATTGC
AGGGCCCCTAGGAAAAAGGGCTGTTGGAAATGTGGAAAGGAAGGACACCAAATGAAGGATTGCACTGAGAGAC
AGGCTAATTTTTTAGGGAAGATCTGGCCTTCCCACAAGGGAAGGCCAGGGAATTTCCTTCAGAGCAGACCAGA
GCCAACAGCCCCACCAGAAGAGAGCTTCAGGTTTGGGGAAGCAACAACTCCCTCTCAGAAGCAGGAGACGATA
GACAAGGAACTGTATCCTTTAACCTCCCTCAAATCACTCTTTGGCAACGACCCCTTGTCACAGTAAGGATAGG
GGGGCAACTAAAAGAAGCTCTATTAGATACAGGAGCAGATGATACAGTATTAGAAGAAATGAATTTGCCAGGA
AGATGGAAACCAAAAATGATAGGGGGAATTGGAGGTTTTATCAAAGTAAGACAGTATGATCAGATACTCATAG
AAATCTGTGGACATAAAGCTATAGGTACAGTATTAGTAGGACCTACACCTGTCAACATAATTGGAAGGAATCT
GTTGACTCAGATTGGTTGCACTTTAAATTTTCCCATTAGTCCTATTGAAACTGTACCAGTAAAACTAAAGCCA
GGAATGGATGGCCCAAAAGTTAAACAATGGCCATTGACAGAAGAAAAAATAAAAGCATTAGTAGAAATTTGTA
CAGAAATGGAAAAGGAAGGAAAAATTTCAAAAATTGGGCCTGAAAATCCATACAATACTCCAGTATTTGCCAT
AAAGAAAAAAGACAGTACTAAATGGAGAAAATTGGTAGATTTCAGAGAACTTAATAAGAGAACTCAAGACTTC
TGGGAAGTTCAATTAGGAATACCACATCCTGCAGGGTTAAAAAAGAAAAAATCAGTAACAGTCCTGGATGTGG
GTGATGCATATTTTTCAGTCCCCTTAGATGAAAACTTTAGAAAGTATACTGCATTTACCATACCTAGTACAAA
CAATGAGACACCAGGGACTAGATATCAGTACAATGTGCTGCCACAGGGATGGAAAGGATCACCAGCAATATTC
CAAAGTAGCATGACAAAAATCTTAGAGCCTTTTAGAAAACAAAATCCAGACATAGTTATCTATCAATACATGG
ATGATTTGTATGTAGGATCCGACTTAGAAATAGGGCAGCATAGAATAAAAATAGAGGAACTGAGAGAACATCT
GTTGAGGTGGGGATTTACCACACCAGACAAAAAACATCAAAAAGAACCTCCATTCCTTTGGATGGGTTATGAA
CTCCATCCTGATAAATGGACAGTACAGCCTATAGTGCTGCCAGAGAAAGACAGCTGGACTGTCAATGACATAC
AGAAGTTAGTGGGAAAATTGAATTGGGCAAGTCAGATTTATCCAGGGATTAAAGTAAAGCAATTATGTAAGCT
CCTTAGGGGAACCAAAGCATTAACAGAAGTAATACCACTAACAGAAGAAGCAGAGCTAGAACTAGCAGAAAAC
AGGGAGATTCTAAAAGAACCAGTACATGGAGTGTATTATGACCCATCAAAAGACTTAGTAGCAGAAATACAGA
AGCAGGGGCAAGGCCAATGGACATATCAAATTTATCAAGAGCCATGTAAAAATCTGAAAACAGGAAAGTATGC
AAGAATGAGGGGTGCCCACACTAATGATATAAAACAGTTAACAGAGGCAGTGCAAAAAATAGCCACAGAAGGC
ATAATAATATGGGGAAAGACTCCTAAATTTAGACTACCCATACAAAAGGAAACATGGGAAGCATGGTGGATGG
AGTATTGGCAAGCCACCTGGATTCCTGAATGGGAATTTGTCAATACCCCTCCCTTAGTAAAATTATGGTATCA
GTTAGAGAAAGAACCCATAGAGGGAGCAGAAACCTTCTATGTAGATGGGGCAGCTAATAGGGAGACTAAATTA
GGAAAAGCAGGATATGTTACTGACAGAGGAAGACAAAAAGTTGTCTCCCTAACTGACACAACAAATCAGAAGA
CTGAGTTACAAGCAATTCATCTAGCGTTGCAGGATTCGGGACTAGAAGTAAACATAGTGACAGACTCACAATA
TGCATTAGGAATCATTCAAGCACAACCAGATAAAAGTGAATCAGAGTTAGTCAGTCAAATAATAGAGCAGTTA
ATAAAAAAGGAAAAAGTCTACCTGGCATGGGTACCAGCACACAAAGGAATTGGAGGAAATGAACAAGTAGATA
AATTAGTCAGTACTGGAATCAGGAGAGTACTATTTTTAGATGGAATAGATAAGGCCCAAGAAGAACATGAGAA
ATATCACAGTAATTGGAGAGCAATGGCTAGTGATTTTAACCTGCCACCTGTAATAGCAAAAGAGATAGTAGCC
TGCTGTGATAAATGTCAGCTAAAAGGAGAAGCCATGCATGGACAAGTAGACTGTAGTCCAGGAATATGGCAAC
TAGACTGTACACATTTAGAAGGAAAAATTGTCCTGGTAGCAGTTCATGTAGCCAGTGGATATATAGAAGCAGA
AGTCATTCCAGCAGAGACAGGGCAGGAAACAGCATACTTTCTCTTAAAATTAGCAGGAAGATGGCCAGTAAAA

ACAATACATACAGACAATGGCAGCAATTTCACCAGTAATGTGGTTAAGGCTGCCTGTTGGTGGGCAGGGATCA
AGCAGGAATTTGGCATTCCCTACAATCCCCAAAGTCAAGGAGTAGTAGAATCTATGAATAAAGAATTAAAGAA
AATTATAGGACAGGTAAGAGATCAGGCTGAACATCTTAGGACAGCAGTACAAATGGCAGTATTCATCCACAAT
TTTAAGAGAAAAGGGGGGATTGGGGGGTACAGTGCAGGGGAAAGAATAGTAGACATGATAGCAACAGACATAC
AAACTAAAGAATTACAAAAACAAATTACAAAAATTCAAAATTTTCGGGTTTATTACAGGGACAGCAGAGATCC
ACTTTGGAAAGGACCAGCAAAGCTTCTCTGGAAAGGTGAAGGGGCAGTAGTAATACAAGATAATAGTGATATA
AAAGTAGTGCCAAGAAGAAAAGCAAAGATCATTAGGGATTATGGAAAACAGATGGCAGGTGATGATTGTGTGG
CAAGTAGACAGGATGAGGATTAGAACATGGAAAAGTTTAGTAAAACACCATATGTATGTTTCACGGAAAGCTG
GGAAATGGTTTTATAGACATCACTATGAAAGCACTCATCCAAAAATAAGTTCAGAAGTACACATCCCACTAGG
GGATGCTAGATTGGTAATAACAACATATTGGGGTCTGCATACAGGAGAAAGAGACTGGCAGTTGGGCCATGGA
GTCTCCATAGAATGGAGGAAAAAGAGATATAGCACACAAGTAGACCCTGATCTAGCAGACCAACTAATCCATC
TGTATTATTTTGATTGTTTTTCAGAATCTGCTATAAGACATGCCATATTAGGACATATAGTTAGACCTAGTTG
TGAATATCAAGCAGGACATAACAAGGTAGGATCCCTACAATACTTGGCACTAACAGCACTAATAAAACCAAAG
AAGATAAAGCCACCTTTGCCTAGTGTTAAGAAACTGACAGAGGATAGATGGAACAAGCCCCAGAAGACCAAGG
GCCACAGAGGGAGCCATACAATGAATGGACGCTAGAGCTTTTAGAGGAGCTTAAGAGTGAAGCTGTTAGACAT
TTTCCTAGGGCATGGCTACATAGCTTAGGACAATATATCTATGAAACTTATGGGGATACTTGGGCAGGAGTGG
AGGCCATAATAAGAATGCTGCAACAACTGCTGTTTATTCATTTCAGAATTGGGTGTCGCCATAGCAGAATAGG
CATTATTCAACAGAGGAGAGCAAGACATGGAGCCAGTAGATCCTAGACTAGAACCCTGGAAGCATCCAGGAAG
TCAGCCTAGGACTCCTTGTACCAATTGCTATTGTAAAAAGTGTTGCCTTCATTGCCAAGTTTGTTTTATGAAA
AAAGGCTTAGGCATCTCCTATGGCAGGAAGAAGCGGAGACAGCGACGAAGACCTCCTCAAGACAGTGAGAATC
ATCAAGTTCCTCTATCAAAGCAGTAAGTAGTACATGTAATGCAACCTTTACATATAGTAGCAATAGTAGCATT
AGTAGTAGCAGCAATAATAGCAATAGTTGTGTGGACCATAGTAGGCATAGAATATAGGAAAATATTAAGACAA
AGAAAAATAGACAGGTTAATTGATAGAATAAGAGAAAGAGCAGAAGACAGTGGCAATGAAAGTGAAGGAGACC
AGGAAGAATTATCAGCACTTGTGGAGATGGGGCACCATGCTCCTTGGGATGTTGATGATCTGTAGGGCTGCAG
AACAGTTGTGGGTCACAGTCTATTATGGGGTACCTGTGTGGAAAGAAGCAAATACCACTCTATTTTGTGCATC
AGATGCTAAAGCATATGATACAGAGGTACATAATGTTTGGGCCACACATGCCTGTGTACCCACAGACCCCAAC
CCACAAGAAGTAGTATTGGAAAATGTGACAGAAGAATTTAACATGTGGAAAAATAACATGGTAGAACAGATGC
ATGAAGATATAATCAGTTTATGGGATCAAAGCCTAAAGCCATGTGTAAAATTAACCCCACTCTGTGTTACTTT
AGATTGCACTGATTTGAGGAATGCTACTAATGCCACTAATAGTAATGGGACAATAAAGGAAGAAATGAAAAAC
TGCTCTTTCAATATCACCACAAGCATAAGAGATAAGGTGCAGAAAGAATATGCACTTTTTTATAGACTTGATA
TAGTACAAATAGAGAATGATAATACTAACAATACTAACAATACTAGCTATAGGATGATAAATTGTAATACCTC
AGTCATTACACAGGCCTGTCCAAAGATATCCTTTGAGCCAATTCCCATACATTATTGTGCCCCGGCTGGTTTT
GCGATTCTAAAGTGTAACAATAAGACGTTCAATGGAAAAGGACCATGTAAAAATGTCAGCACAGTACAATGTA
CACATGGAATTAAGCCAGTAGTGTCAACTCAACTGCTGTTAAATGGCAGTCTAGCAGAAAAAGAGGTGGTAAT
TAGATCTGTCAATTTCACAGACAATGCTAAAACCATAATAGTACAGCTGAACAAATCTGTAGAAATTAATTGT
ACAAGACCCAACAACAATACAAGAAAAAGTATACATATAGGACCAGGGAGAGCATTTTATGCAACAGGAGACA
TAATAGGAGATATAAGACAAGCACATTGTAACATTAGTAGAGCAGAATGGAATAACACTTTAAGACAGATAGC
TAAAAAATTAAGAGAACAATTTGTGAATAAAACAATAGTCTTTAATCAATCCTCAGGAGGGGACCCAGAAATT
GTAATGCACAGTTTTAATTGTGGAGGGGAATTTTTCTACTGTAATTCAACACAATTGTTTAATAGTACTTGGA
ATGAAACCAACATTGACGGAAATGACACCACTAAAGGAGATAATATCACAGATGTCATCACACTCCCATGCAG
AATAAAACAAATTATAAACATGTGGCAGGAAGTAGGAAAAGCAATGTATGCCCCTCCCATCAGAGGACAAATT
AATTGTTTATCAAATATTACAGGGCTGCTATTAACAAGAGATGGTGGTAATCAGAGTGGGAACACCGAGATCT
TCAGACCTGTAGGGGGAAATATGAAGGACAATTGGAGAAGTGAATTATATAAATATAAAGTAGTACAAATTGA
ACCATTAGGAGTAGCACCCACCAAGGCAAAGAGAAGAGTGGTGCAGAGAGAAAAAAGAGCAGTGGGAACGATA
GGAGCCATGTTCCTTGGGTTCTTGGGAGCAGCAGGAAGCACTATGGGCGCAGCGTCAATGACGCTGACGGTAC
AGGCCAGACAATTATTGTCTGGTATAGTGCAACAGCAGAGCAATTTGCTGAGGGCTATTGAGGCGCAACAGCA
TATGTTGCAACTCACAGTCTGGGGCATCAAGCAGCTCCAGGCAAGAGTACTGGCTGTGGAAAGATACCTACAG
GATCAACGGCTCCTAGGGATTTGGGGTTGCTCTGGAAAACTCATCTGCACCACTGCTGTGCCTTGGAATACTA
GTTGGAGTAATAAAAATCTGACTCAGATTTGGGATAACATGACCTGGATGCAGTGGGAAAAAGAAATTAACAA
TTACACAGGAGTAATATACAACTTACTTGAAAAATCGCAGAACCAACAAGAAAAGAATGAACAAGAATTATTG
GAATTAGATGAGTGGGCAAGTTTGTGGAATTGGTTTGACATAACAAAATGGCTGTGGTATATAAAAATATTCA
TAATGATAGTAGGAGGCTTGGTAGGTTTGAGAATAGTTTTTGCTGTACTTTCTATAGTGAATAGAGTTAGGCA
GGGATACTCACCATTATCATTTCAGACCCTCCTCCCAGCCCCGAGGGGACCCGACAGGCCCGAAGGAATA?AA
GAAGAAGGTGGAGAGAGAGACAGAGGCAGATCAAATCGATTAGCAACTGGATTCTTGATACTTTTCTGGGACG

ACCTGCGGAGCCTGTGCCTCTTCAGCTACCACCGATTGAGAGACTTACTCTTGATTGTAGGGAGGATTGTGGG
AATTCTGGGACACAGGGGGTGGGAGATCCTCAAATATTGGTGGAATCTCCTGCAATATTGGAGTCAGGAACTA
AAGAATAGTGCTGTTAGCTTGCTCAATGCCACAGCTATCGCAGTAGCTGAGGGAACAGATAGGGTTATAGAAG
TAGTACGAAGAGTTTTTAGAGCTATTCTCCACATACCTACAAGAGTGAGACAGGGCTTGGAAAGGGCTTTGCT
ATAAGATGGGTGGCAAGTGGTCAAAACGTAGTCTGGGTGGATGGCCTAATGTAAGGGAAAGAATGAGAAGAAC
TGAGCCAGCAGCAGATGGGGTGGGAGCAGTATCTCGAGACCTGGAAAAACATGGGGCAATCACAAGTAGCAAT
ACAGCAACTAGTAATGCTGCCTGTGCCTGGCTAGAAGCACAAGAGGAAGAGGAGGTGGGTTTTCCAGTCAGAC
CTCAGGTACCTTTAAGACCAATGACTTACAAGGGAGCTTTAGATCTTAGCCACTTTTTAAGAGAAAAGGGGGG
ACTGGAAGGGCTAATTCACTCCCAGAAAAGACAAGAGATCCTTGATCTGTGGGTCTACCACACACAAGGCTAC
TTCCCTGATTGGCAGAACTACACACCAGGGCCAGGGATCAGATATCCCCTGACCTTTGGATGGTGCTTCAAGT
TAGTACCAGTTGAGCCAGACGAAGAAGAAAACAGCAGCTTGCTACACCCTATGAGCCAGCATGGGATGGAGGA
CACGGAGAGAGAAGTGTTAAAGTGGAAGTTTGACAGCCGCCTAGCATTTCATCACATGGCCCGAGAGCTGCAT
CCGGAGTATTACAAAGACTGCTGACACCGGGTTTTCTACAAGGGACTTTCCGCTGGGGACTTTCCAGGGAGGC
GTGGCCTGGGCGGGACTGGGGAGTGGCGAGCCCTCAGATGCTGCATATAAGCAGCTGCTTTTTGCCTGTACTG
GGTCTCTCTGGTTAGACCAGATCAGAGCCTGGGAGCTCTCTGGCTAACTAGGGAACCCACTGCTTAAGCCTCA
ATAAAGCTTGCCT

>AB-22B HIV-1 genome, derived from RNA genomic sequence
ACGCAGGACTCGGCTTGCTGAAGCGCGCACGGCAAGAGGCGAGGGGCGGCGACTGGTGAGTACGCCAAATTTT
GACTAGCGGAGGCTAGAAGGAGAGAGATGGGTGCGAGAGCGTCAGTATTAAGTGCGGGGGAATTAGATACGTG
GGAAAAAATTCGGTTAAGGCCAGGGGGAAAGAAAAAATATAGATTAAAACATATAGTATGGGCAAGCAGGGAG
CTAGAACGATTTGCAGTTAATCCTGGCCTGTTAGAAACATCAGCAGGCTGTAGACAAATACTGGGACAGCTAC
ATCCATCCCTTCAGACAGGATCAGAAGAACTTAGATCATTATATAATACAGTAGCAACCCTCTATTGTGTGCA
TCAAAAGATAGAGGTAAAAGACACCAAGGAAGCTTTAGAGAAGATAGAGGAAGAACAAAACAAAAGTAAGAAA
AAGGCACAACAAGCAGCAGCTGACACAGGAAACAGCAGCCAGGTCAGCCAAAATTACCCCATAGTACAGAACC
TCCAGGGGCAAATGGTACATCAGACCATGTCACCTAGAACTTTAAATGCATGGGTAAAAGTAATAGAAGAGAA
GGCTTTCAGCCCAGAAGTAATACCCATGTTTTCAGCATTATCAGAAGGAGCCACCCCACAAGATTTAAACACC
ATGCTAAACACAGTGGGGGGACATCAAGCAGCCATGCAAATGTTAAAAGATACCATCAATGAGGAAGCTGCGG
AATGGGATAGATTGCATCCAGTGCATGCAGGGCCTATTGCACCAGGCCAGATGAGAGAACCAAGGGGAAGTGA
CATAGCAGGAACTACTAGTACCCTTCAGGAACAAATAGGATGGATGACACATAATCCACCTATCCCAGTAGGA
GAAATTTATAAGAGATGGATAATCCTGGGATTAAATAAAATAGTAAGAATGTATAGCCCTACCAGCATTCTGG
ACATAAAACAAGGACCAAAAGAACCCTTTAGAGATTATGTAGACCGGTTCTATAAAACTCTAAGAGCCGAGCA
AGCTTCACAGGATGTAAAAAATTGGATGACAGAAACCTTGTTGGTCCAAAATGCGAATCCAGATTGTAAGACT
ATTTTAAAAGCATTGGGACCAGCAGCTACATTAGAAGAAATGATGACAGCATGTCAGGGAGTGGGGGGACCCA
GCCATAAAGCAAGAGTCTTGGCTGAAGCAATGAGCCAAGCAACAGGTTCAGCTACCATAATGATGCAGAGAGG
CAATTTTAGGAACCAAAGAAAGACTGTTAAGTGTTTCAATTGTGGCAAAGAGGGGCACATAGCCAGAAATTGC
AGGGCCCCTAGGAAAAAGGGCTGTTGGAAATGTGGAAAGGAAGGACACCAAATGAAGGATTGCACTGAGAGAC
AGGCTAATTTTTTAGGGAAGATCTGGCCTTCCCACAAGGGAAGGCCAGGGAATTTCCTTCAGAGCAGACCAGA
GCCAACAGCCCCACCAGAAGAGAGCTTCAGGTTTGGGGAAGCAACAACTCCCTCTCAGAAGCAGGAGACGATA
GACAAGGAACTGTATCCTTTAACCTCCCTCAAATCACTCTTTGGCAACGACCCCTTGTCACAGTAAGGATAGG
GGGGCAACTAAAAGAAGCTCTATTAGATACAGGAGCAGATGATACAGTATTAGAAGAAATGAATTTGCCAGGA
AGATGGAAACCAAAAATGATAGGGGGAATTGGAGGTTTTATCAAAGTAAGACAGTATGATCAGATACTCATAG
AAATCTGTGGACATAAAGCTATAGGTACAGTATTAGTAGGACCTACACCTGTCAACATAATTGGAAGGAATCT
GTTGACTCAGATTGGTTGCACTTTAAATTTTCCCATTAGTCCTATTGAAACTGTACCAGTAAAACTAAAGCCA
GGAATGGATGGCCCAAAAGTTAAACAATGGCCATTGACAGAAGAAAAAATAAAAGCATTAGTAGAAATTTGTA
CAGAAATGGAAAAGGAAGGGAAAATTTCAAAAATTGGGCCTGAAAATCCATACAATACTCCAGTATTTGCCAT
AAAGAAAAAAGACAGTACTAAATGGAGAAAATTGGTAGATTTCAGAGAACTTAATAAGAGAACTCAAGACTTC
TGGGAAGTTCAATTAGGAATACCACATCCTGCAGGGTTAAAAAAGAAAAAATCAGTAACAGTCCTGGATGTGG
GTGATGCATATTTTTCAGTCCCCTTAGATGAAAACTTTAGAAAGTATACTGCATTTACCATACCTAGTACAAA
CAATGAGACACCAGGGACTAGATATCAGTACAATGTGCTGCCACAGGGATGGAAAGGATCACCAGCAATATTC
CAAAGTAGCATGACAAAAATCTTAGAGCCTTTTAGAAAACAAAATCCAGACATAGTTATCTATCAATACATGG
ATGATTTGTATGTAGGATCCGACTTAGAAATAGGGCAGCATAGAATAAAAATAGAGGAACTGAGAGAACATCT
GTTGAGGTGGGGATTTACCACACCAGACAAAAAACATCAAAAAGAACCTCCATTCCTTTGGATGGGTTATGAA
CTCCATCCTGATAAATGGACAGTACAGCCTATAGTGCTGCCAGAGAAAGACAGCTGGACTGTCAATGACATAC
AGAAGTTAGTGGGAAAATTGAATTGGGCAAGTCAGATTTATCCAGGGATTAAAGTAAAGCAATTATGTAAGCT
CCTTAGGGGAACCAAAGCATTAACAGAAGTAATACCACTAACAGAAGAAGCAGAGCTAGAACTAGCAGAAAAC
AGGGAGATTCTAAAAGAACCAGTACATGGAGTGTATTATGACCCATCAAAAGACTTAGTAGCAGAAATACAGA
AGCAGGGGCAAGGCCAATGGACATATCAAATTTATCAAGAGCCATGTAAAAATCTGAAAACAGGAAAGTATGC
AAGAATGAGGGGTGCCCACACTAATGATATAAAACAGTTAACAGAGGCAGTGCAAAAAATAGCCACAGAAGGC
ATAATAATATGGGGAAAGACTCCTAAATTTAGACTACCCATACAAAAGGAAACATGGGAAGCATGGTGGATGG
AGTATTGGCAAGCCACCTGGATTCCTGAATGGGAATTTGTCAATACCCCTCCCTTAGTAAAATTATGGTATCA
GTTAGAGAAAGAACCCATAGAGGGAGCAGAAACCTTCTATGTAGATGGGGCAGCTAATAGGGAGACTAAATTA
GGAAAAGCAGGATATGTTACTGACAGAGGAAGACAAAAAGTTGTCTCCCTAACTGACACAACAAATCAGAAGA
CTGAGTTACAAGCAATTCATCTAGCGTTGCAGGATTCGGGACTAGAAGTAAACATAGTGACAGACTCACAATA
TGCATTAGGAATCATTCAAGCACAACCAGATAAAAGTGAATCAGAGTTAGTCAGTCAAATAATAGAGCAGTTA
ATAAAAAAGGAAAAAGTCTACCTGGCATGGGTACCAGCACACAAAGGAATTGGAGGAAATGAACAAGTAGATA
AATTAGTCAGTACTGGAATCAGGAGAGTACTATTTTTAGATGGAATAGATAAGGCCCAAGAAGAACATGAGAA
ATATCACAGTAATTGGAGAGCAATGGCTAGTGATTTTAACCTGCCACCTGTAATAGCAAAAGAGATAGTAGCC
TGCTGTGATAAATGTCAGCTAAAAGGAGAAGCCATGCATGGACAAGTAGACTGTAGTCCAGGAATATGGCAAC
TAGACTGTACACATTTAGAAGGAAAAATTGTCCTGGTAGCAGTTCATGTAGCCAGTGGATATATAGAAGCAGA
AGTCATTCCAGCAGAGACAGGGCAGGAAACAGCATACTTTCTCTTAAAATTAGCAGGAAGATGGCCAGTAAAA

ACAATACATACAGACAATGGCAGCAATTTCACCAGTAATGTGGTTAAGGCTGCCTGTTGGTGGGCAGGGATCA
AGCAGGAATTTGGCATTCCCTACAATCCCCAAAGTCAAGGAGTAGTAGAATCTATGAATAAAGAATTAAAGAA
AATTATAGGACAGGTAAGAGATCAGGCTGAACATCTTAGGACAGCAGTACAAATGGCAGTATTCATCCACAAT
TTTAAGAGAAAAGGGGGGATTGGGGGGTACAGTGCAGGGGAAAGAATAGTAGACATGATAGCAACAGACATAC
AAACTAAAGAATTACAAAAACAAATTACAAAAATTCAAAATTTTCGGGTTTATTACAGGGACAGCAGAGATCC
ACTTTGGAAAGGACCAGCAAAGCTTCTCTGGAAAGGTGAAGGGGCAGTAGTAATACAAGATAATAGTGATATA
AAAGTAGTGCCAAGAAGAAAAGCAAAGATCATTAGGGATTATGGAAAACAGATGGCAGGTGATGATTGTGTGG
CAAGTAGACAGGATGAGGATTAGAACATGGAAAAGTTTAGTAAAACACCATATGTATGTTTCACGGAAAGCTG
GGAAATGGTTTTATAGACATCACTATGAAAGCACTCATCCAAAAATAAGTTCAGAAGTACACATCCCACTAGG
GGATGCTAGATTGGTAATAACAACATATTGGGGTCTGCATACAGGAGAAAGAGACTGGCAGTTGGGCCATGGA
GTCTCCATAGAATGGAGGAAAAAGAGATATAGCACACAAGTAGACCCTGATCTAGCAGACCAACTAATCCATC
TGTATTATTTTGATTGTTTTTCAGAATCTGCTATAAGACATGCCATATTAGGACATATAGTTAGACCTAGTTG
TGAATATCAAGCAGGACATAACAAGGTAGGATCCCTACAATACTTGGCACTAACAGCACTAATAAAACCAAAG
AAGATAAAGCCACCTTTGCCTAGTGTTAAGAAACTGACAGAGGATAGATGGAACAAGCCCCAGAAGACCAAGG
GCCACAGAGGGAGCCATACAATGAATGGACGCTAGAGCTTTTAGAGGAGCTTAAGAGTGAAGCTGTTAGACAT
TTTCCTAGGGCATGGCTACATAGCTTAGGACAATATATCTATGAAACTTATGGGGATACTTGGGCAGGAGTGG
AGGCCATAATAAGAATGCTGCAACAACTGCTGTTTATTCATTTCAGAATTGGGTGTCGCCATAGCAGAATAGG
CATTATTCAACAGAGGAGAGCAAGACATGGAGCCAGTAGATCCTAGACTAGAACCCTGGAAGCATCCAGGAAG
TCAGCCTAGGACTCCTTGTACCAATTGCTATTGTAAAAAGTGTTGCCTTCATTGCCAAGTTTGTTTTATGAAA
AAAGGCTTAGGCATCTCCTATGGCAGGAAGAAGCGGAGACAGCGACGAAGACCTCCTCAAGACAGTGAGAATC
ATCAAGTTCCTCTATCAAAGC?AGTAAGTAGTACATGTAATGCAACCTTTACATATAGTAGCAATAGTAGCAT
TAGTAGTAGCAGCAATAATAGCAATAGTTGTGTGGACCATAGTAGGCATAGAATATAGGAAAATATTAAGACA
AAGAAAAATAGACAGGTTAATTGATAGAATAAGAGAAAGAGCAGAAGACAGTGGCAATGAAAGTGAAGGAGAC
CAGGAAGAATTATCAGCACTTGTGGAGATGGGGCACCATGCTCCTTGGGATGTTGATGATCTGTAGGGCTGCA
GAACAGTTGTGGGTCACAGTCTATTATGGGGTACCTGTGTGGAAAGAAGCAAATACCACTCTATTTTGTGCAT
CAGATGCTAAAGCATATGATACAGAGGTACATAATGTTTGGGCCACACATGCCTGTGTACCCACAGACCCCAA
CCCACAAGAAGTAGTATTGGAAAATGTGACAGAAGAATTTAACATGTGGAAAAATAACATGGTAGAACAGATG
CATGAAGATATAATCAGTTTATGGGATCAAAGCCTAAAGCCATGTGTAAAATTAACCCCACTCTGTGTTACTT
TAGATTGCACTGATTTGAGGAATGCTACTAATGCCACTAATAGTAATGGGACAATAAAGGAAGAAATGAAAAA
CTGCTCTTTCAATATCACCACAAGCATAAGAGATAAGGTGCAGAAAGAATATGCACTTTTTTATAGACTTGAT
ATAGTACAAATAGAGAATGATAATACTAACAATACTAACAATACTAGCTATAGGATGATAAATTGTAATACCT
CAGTCATTACACAGGCCTGTCCAAAGATATCCTTTGAGCCAATTCCCATACATTATTGTGCCCCGGCTGGTTT
TGCGATTCTAAAGTGTAACAATAAGACGTTCAATGGAAAAGGACCATGTAAAAATGTCAGCACAGTACAATGT
ACACATGGAATTAAGCCAGTAGTGTCAACTCAACTGCTGTTAAATGGCAGTCTAGCAGAAAAAGAGGTGGTAA
TTAGATCTGTCAATTTCACAGACAATGCTAAAACCATAATAGTACAGCTGAACAAATCTGTAGAAATTAATTG
TACAAGACCCAACAACAATACAAGAAAAAGTATACATATAGGACCAGGGAGAGCATTTTATGCAACAGGAGAC
ATAATAGGAGATATAAGACAAGCACATTGTAACATTAGTAGAGCAGAATGGAATAACACTTTAAGACAGATAG
CTAAAAAATTAAGAGAACAATTTGTGAATAAAACAATAGTCTTTAATCAATCCTCAGGAGGGGACCCAGAAAT
TGTAATGCACAGTTTTAATTGTGGAGGGGAATTTTTCTACTGTAATTCAACACAATTGTTTAATAGTACTTGG
AATGAAACCAACATTGACGGAAATGACACCACTAAAGGAGATAATATCACAGATGTCATCACACTCCCATGCA
GAATAAAACAAATTATAAACATGTGGCAGGAAGTAGGAAAAGCAATGTATGCCCCTCCCATCAGAGGACAAAT
TAATTGTTTATCAAATATTACAGGGCTGCTATTAACAAGAGATGGTGGTAATCAGAGTGGGAACACCGAGATC
TTCAGACCTGTAGGGGGAAATATGAAGGACAATTGGAGAAGC????????????????????????????
??
??
??
??
??
??
??
??
??
??
???ATCAAGTTCCTCTATCAAAGCA?ACCCTCCTCCCAGCCCCGAGGGGACCCGACAGGCCCGAAGGAATAGA
AGAAGAAGGTGGAGAGAGAGACAGAGGCAGATCAAATCGATTAGCAACTGGATTCTTGATACTTTTCTGGGAC

GACCTGCGGAGCCTGTGCCTCTTCAGCTACCACCGATTGAGAGACTTACTCTTGATTGTAGGGAGGATTGTGG
GAATTCTGGGACACAGGGGGTGGGAGATCCTCAAATATTGGTGGAATCTCCTGCAATATTGGAGTCAGGAACC
AAAG

>AB-22D HIV-1 genome, derived from RNA genomic sequence
ACGCAGGACTCGGCTTGCTGAAGCGCGCACGGCAAGAGGCGAGGGGCGGCGACTGGTGAGTACGCCAATTTTT
GACTAGCGGAGGCTAGAAGGAGAGAGATGGGTGCGAGAGCGTCAGTATTAAGTGCGGGGGAATTAGATACATG
GGAGAAAATTCGGTTAAGGCCAGGAGGAAAGAAAAAATATAGATTAAAACATATAGTATGGGCAAGCAGGGAG
CTAGAACGATTTGCAGTTAATCCTGGCCTGTTAGAAACATCAGCAGGATGTAGACAAATAATGGGACAGCTAC
ATCCATCCCTTCAGACAGGATCAGAAGAGCTTAGGTCATTATATAATACAATAGCAGTCCTCTATTGTGTACA
TCAAAAGATAGAGGTAAAAGACACCAAGGAAGCTTTAGAGAAGGTAGAGGAAGAGCAAAACAAAAGTAAGAAA
AAAGTACAGCAAGTGGCAGCTGACGCAGGAAACAGCAGCCCGGTCAGCCAAAATTACCCTATAGTACAGAACC
TCCAGGGGCAAATGGTACACCAGACCATGTCACCTAGAACTTTAAATGCATGGGTAAAAGTGATAGAAGAGAA
GGCTTTCAGCCCAGAAGTAATACCCATGTTTTCAGCATTATCAGAAGGAGCCACCCCACAAGATTTAAACACC
ATGCTAAACACAGTGGGGGGACATCAAGCAGCCATGCAAATGTTAAAAGATACCATCAATGAGGAGGCTGCAG
AATGGGATAGATTGCATCCAGTGCATGCAGGGCCTATTGCACCAGGCCAGATGAGAGAACCAAGGGGAAGTGA
CATAGCAGGAACTACTAGTACCCTTCAGGAACAAATAGGATGGATGACACATAATCCACCTATCCCAGTAGGA
GAAATCTATAAGAGATGGATAATCCTGGGATTAAATAAAATAGTAAGAATGTATAGCCCTACCAGCATTCTGG
ACATAAAACAAGGACCAAAAGAACCCTTTAGAGATTATGTAGACCGGTTCTATAAAACTCTAAGAGCCGAGCA
AGCTTCACAGGATGTAAAAAATTGGATGACAGAAACCTTGTTGGTCCAAAATGCGAATCCAGATTGTAAGACC
ATTTTAAAAGCATTAGGACCAGCAGCCACATTAGAAGAAATGATGACAGCATGTCAGGGAGTGGGAGGACCCA
GCCATAAAGCAAGAGTCTTGGCTGAAGCAATGAGCCAAGCAACAGGTTCAGCTAACATAATGATGCAAAGAGG
TAATTTTAGGAACCAAAGAAAGACTGCTAAGTGTTTCAATTGTGGCAAAGAAGGGCACATAGCCAGAAATTGC
AGGGCCCCTAGGAAAAAGGGCTGTTGGAAATGTGGAAAGGAAGGACACCAAATGAAGGATTGCACTGAAAGAC
AGGCTAATTTTTTAGGGAGAATCTGGCCTTCCCACAAGGGGAGGCCAGGGAATTTTCTTCAGAGCAGACCAGA
GCCATCAGCCCCACCAGAAGAGAGCTTCAGGTTTGGGGAGGAAGCAACAACTCCCTCTCAGAAGCAGGAGACG
ATAGACAAGGAACTGTATCCTTTAACCTCCCTCAAATCTCTCTTTGGCAACGACCCATCCTCACAGTAAGGGT
AGGGGGGCACCTAATAGAAGCTCTATTAGATACAGGAGCAGATGATACAGTGTTAGAAGAAATAAATTTACCA
GGAAGATGGAAACCAAAAATGATAGGGGGAATTGGAGGTTTTGTCAAAGTAAGACAGTATGAGCAGGTACCCA
TAGAAATCTGTGGGCATGAAGTCATAAGTACAGTATTAGTAGGACCTACACCTGCCAACGTAATTGGAAGAAA
TGTGATGTCTCAAATTGGTTGCACTCTAAATTTTCCCATTAGTCCTATTGAAACTGTACCAGTAAAATTAAAG
CCAGGAATGGATGGCCCAAAAGTTAAACAATGGCCATTGACAGAAGAAAAAATAAAAGCATTAATAGAAATTT
GTACAGAATTGGAAAAGGAAGGGAAAATTTCAAAAATTGGGCCTGAAAATCCATACAATACTCCAGTATTTGC
CATAAAGAAAAAAGACAGTACTAAATGGAGAAAATTGGTAGATTTCAGAGAACTTAATAAGAGAACTCAAGAC
TTCTGGGAAGTTCAATTGGGAATACCACATCCCGCAGGGTTAAAAAAGAAAAAATCAGTAACAGTCTTGGATG
TGGGTGATGCATATTTTTCAATTCCCTTGGATGAAGACTTTAGAAAGTATACTGCATTCACCATACCTAGTAC
AAACAATGAGACACCAGGGATTAGATATCAGTACAATGTGCTGCCACAGGGATGGAAAGGATCACCAGCAATA
TTCCAAAGTAGCATGACAAAAATCTTAGAGCCTTTTAGAAAACAAAATCCAGACATAGTTATCTACCAATACG
TGGATGATTTATATGTAGGATCAGACTTAGAAATAGGACAGCATAGAATAAAAATAGAGGAACTGAGAGAACA
TCTGTGGAGGTGGGGATTTTACACACCAGACAAAAAACATCAGAAAGAACCTCCATTCCTCTGGATGGGTTAT
GAACTCCATCCTGATAAATGGACAGTACAGCCTATAGTGCTGCCAGAAAAAGACAGCTGGACTGTCAATGACA
TACAGAAGTTAGTGGGAAAATTGAATTGGGCAAGTCAAATTTATCCAGGGATTAAAGTGAAGCAATTATGTAA
GCTCCTTAAGGGAACCAAGGCACTAACAGAGGTAGTACCACTAACACAAGAAGCAGAGCTAGAACTAGCAGAA
AATAGGGAGATTCTAAAAGAACCAGTACATGGAGTGTATTATGACCCATCAAAAGAATTAATAGCAGAAATAC
AGAAGCAGGGACAAGGCCAATGGACATATCAAATTTATCAAGAGCAAGGTAAAAATTTGAAAACAGGAAAGTA
TGCAAGAATGAGGAGTGCCCACACCAATGATATAAAACAGTTAACAGAGGCAGTGCAAAAGATAAGCATGGAA
AGCATAGTAATATGGGGAAAGACTCCTAAATTTAGACTACCCATACAAAAGGAAACATGGGAAGCATGGTGGA
TGGAGTATTGGCAAGCCACCTGGATTCCTGAGTGGGAGTTTGTCAATACCCCTCCCTTAGTAAAATTATGGTA
TCAGTTAGAGAAAGAACCCATAGTGGGAGCAGAAACTTTCTATGTAGATGGGGCAGCTAATAGAGAAACTAAA
TTAGGAAAAGCAGGATATGTTACTGACAGAGGAAGACAAAAGGTTGTCTCCCTGACTGACACAACAAATCAGA
AGACTGAGTTACAAGCAATTCATCTAGCCTTGCAGGATTCGGGATTAGAAGTAAACATAGTAACAGACTCACA
ATATGCATTAGGAATCATTCAAGCACAACCAGATCAAAGTGAATCAGAGTTAGTCAATCAGATAATAGAGCAG
TTAATAAAAAAGGAAAAAATCTACCTGGCATGGGTACCAGCGCACAAAGGAATTGGAGGAAATGAGCAGGTAG
ATAAATTAGTCAGTACTGGAGTCAGGAGAGTACTATTTTTAGATGGAATAGATAAGGCCCAAGAAGAACATGA
GAAATATCACAGTAATTGGAGAGCAATGGCTAGTGATTTTAACCTGCCACCTGTAATAGCAAAAGAAATAGTA
GCCTGCTGTGATAAATGTCAGCTAAAAGGAGAAGCCATGCATGGACAAGTAGACTGTAGTCCAGGAATATGGC
AACTAGATTGTACACATTTAGAAGGAAAAATTATCATAGTAGCAGTTCATGTAGCCAGTGGATATGTAGAAGC
AGAAGTCATTCCAGCAGAGACAGGACAGGAAACGGCATACTTTCTCTTAAAATTAGCAGGAAGATGGCCAGTA

AAAACAATACATACAGACAATGGCAGCAATTTCACCAGTAATGTGGTTAAGGCTGCCTGTTGGTGGGCAGGGA
TCAAGCAGGAATTTGGCATTCCCTACAATCCCCAGAGTCAAGGAGTAGTAGAATCCATGAATAAAGAATTAAA
GAAAATTATAGGACAAGTAAGAGATCAGGCTGAGCATCTTAAGACAGCAGTACAAATGGCAGTATTCATCCAC
AATTTTAAGAGAAAAGGGGGGATTGGGGGGTACAGTGCAGGGGAAAGAATAGTAGACATAATAGCAACAGACA
TACAAACTAAAGAGTTACAAAAACAAATTACAAAAGTTCAAAATTTTCGGGTTTATTACAGGGACAGCAGAGA
TCCACTTTGGAAAGGACCAGCAAAACTTCTCTGGAAAGGTGAAGGGGCAGTAGTAATACAAGAAAATAGTGAT
ATAAAAGTAGTGCCAAGAAGAAAAGCAAAGATCATTAGGGATTATGGAAAACAGATGGCAGGTGATGATTGTG
TGGCAAGTAGACAGGATGAGGATTAGAACATGGAAAAGTTTAGTAAAACACCATATGTATGTCTCACGGAAAG
CTGGGAAATGGTTTTATAGACATCACTATGAAAGCACTCATCCAAAAATAAGTTCAGAAGTACACATCCCAAT
AGGGGATGCTAGATTGGTAATAACAACATATTGGGGTCTGCAAACAGGAGAAAGAGACTGGCAGTTGGGCCAT
GGAGTCTCCATAGAATGGAGGAAAAGGAGATACAGCACACAAGTAGACCCTGATCTAGCAGACCAACTAATCC
ACCTGTATTATTTTGATTGTTTTTCAGAATCTGCTATAAGACATGCCATATTAGGACATATAGTTAGACCTAG
TTGTGAATATCAAGCAGGACATAACAAGGTAGGATCCCTACAATACTTGGCACTAACAGCACTGATAAAACCG
AAGAAGATAAAGCCACCTTTGCCTAGTGTTAAGAAACTGACAGAGGATAGATGGAACAAGCCCCGGAAGACCA
AGGGCCACAGAGGGAGCCATACAATGAATGGACACTAGAGCTTTTAGAGGAGCTTAAGAGTGAAGCTGTTAGA
CATTTTCCTAGGGCATGGCTACATAGCTTAGGACAATATATCTATGAAACTTATGGGGACACTTGGGCAGGAG
TGGAGGCCATAATAAGAATGCTGCAACAACTGCTATTTATTCATTTCAGAATTGGGTGTCGCCATAGCAGAAT
AGGCATTATTCGACAAAGGAGAGCAAGAAATGGAGCCAGTAGATCCTAGACTAGAGCCCTGGAAGCATCCAGG
AAGTCAGCCTAGGACTCCTTGTACCAATTGTTATTGTAAAAAGTGTTGCCTTCATTGCCAAGTTTGTTTTATG
AAAAAAGGCTTAGGCATCTCCTATGGCAGGAAGAAGCGGAGACAGCGACGAAGACCTCCTCCAGACAGTAAGA
ATCATCAAGTTCCTCCATCAAAGCAGTAAGTAGTACATGTAATGCAACCTTTAAATATAGTAGCAATAGTAGC
ATTAGTAGTAGCAGCAATAATAGCAATAGTTGTGTGGACCATAGTAGGCATAGAATATAGGAAAATATTAAGA
CAAAGGAAAATAGATAGGTTAATTGATAGGATAAGAGAAAGAGCAGAAGACAGTGGCAATGAAAGCGAAGGAG
ACCAGGAAGAATTATCAGCACTTGTGGAAATGGGGCACGATGCTCCTTGGAATGTTGATGATCTGTAGTGCTG
CAGAACAATTGTGGGTCACAGTCTATTATGGGGTACCTGTGTGGAAAGACGCAAATACCACTCTATTCTGTGC
ATCAGATGCTAAAGCATATGAGACAGAGGCACATAATGTTTGGGCCACACATGCCTGTGTACCCACAGACCCC
GACCCACAAGAAGTAAAATTGGCAAATGTGACAGAGGAATTTAACATGTGGGAAAACGACATGGTAGAACAGA
TGCATGAAGATATAATCAGTTTATGGGATCAAAGCCTAAAGCCATGTGTAAAATTAACCCCGCTCTGTGTCAC
TTTAAATTGCACTGATCTGGGGAATGTTAATACTACTACCCATAATATTACTACTAATGAGACAATAAAGGGA
GAAATGAAAAACTGCTCTTTCAATATTACCACAAGCATAAGAGATCAGGTGCAGAAAGAATATGCACTTTTTT
ATAAACTTGATATAGTAGAAATAAAGAATGATAATACTAACAATACTGACAGTACTAACTATACTAGCTATAT
AATGAGACATTGTAATGCCTCAGTCATTACACAGGCCTGTCCAAAGACATCCTTTGAGCCAATTCCCATACAT
TATTGTGCCCCGGCTGGTTTTGCGATTCTAAAGTGTAACAATAAGACATTCGATGGAACAGGGAACTGTACAA
ATGTCAGCACAGTACAATGTACACATGGAATTAAGCCAGTAGTGTCAACTCAGCTGCTGTTAAATGGCAGTTT
AGCAGAAGAAAAAGTGGTAATTAGATCTGTTAATTTCTCGAACAATGCTAAAACCATAATAGTACAGCTGAAC
AAATCTGTAGAAATTAACTGTACAAGACCCAACAACAATACAGGAAAAACTATACACCTGGGATGGAGGAGAT
CATTTTTTACAACAGAGACCATAATAGGAGATATAAGACGTGCACATTGTAACATTAGTAAAACAGCATGGAA
TAACACTTTAAGACAGATAGCTGGAGAATTAAGAAAACGATTTGAGGATAAAACAATAGTCTTTAATCGCTCC
TCAGGAGGGGACCCAGAAATTGTAATGCACAGTTTTAATTGTGGAGGGGAATTTTTCTACTGTGATACAACAC
AACTGTTTAATAGTACTTGGAATGAAACTGACATTAACAGAAATAACACAAATGAAGGAAATAAGACAGAGGT
CATCACACTCCCATGCAGAATAAAACAAATTGTAAACATGTGGCAGGGAATAGGAAAAGCAATGTATGCCCCT
CCCATCAGAGGACGAATTTATTGTGTATCAAATATTACAGGGCTGCTATTAACAAGAGATGGTGGTAATCAGA
GTGGGAGCAACACCACCGAGACCTTCAGACCTCAAGGGGGAGACATGAAGGACAATTGGAGGAGTGAATTATA
TAAATATAAAGTAGTACAACTTGAACCATTAGGAGTAGCACCCACCAAGGCAAAGAGAAGAGTGGTGCAGAGA
GAAAAAAGAGCAGTGGGAATGCTAGGAGCCATGTTCCTTGGGTTCTTGGGAGCAGCGGGAAGCACTATGGGCG
CAGCGTCAATGACGCTGACGGTACAGGCCAGACAATTAATGACTGGTATAGTGCAACAGCAGAGCAATTTGCT
GAGGGCTATTGAGGCACAACAGCGTATGTTGCAACTCACAGTCTGGGGCATTAAGCAGCTCCAGGCAAGAGTA
CTGGCTGTGGAAAGATACCTACAGGATCAACGGCTCCTAGGGATTTGGGGTTGCTCTGGAAAGCTCATCTGCA
CCACTGCTGTGCCTTGGAATAGTAGTTGGAGTAATAGAAATCTGACTCAGATTTGGGATAATATGACCTGGAT
ACAGTGGGAAAGAGAAATTAACAATTACACAGGAGTAATATACAACTTACTTGAAGAATCGCAAAACCAACAA
GAAAAGAATGAACAAGAATTGTTGGAATTGGATAAGTGGAACAGTTTATGGAATTGGTTTGACATAACACAAT
GGCTGTGGTATATAAAAATATTCATAATGATAGTAGGAGGCTTGATAGGTTTAAGAATAATTTTTGCTGTGCT
TTCTATAGTGAATAAAGTTAGGCAGGGATATTCACCATTATCATTTCAGACCCTCCTCCCAGTCCCGAGGGGA
CCCGACGGGCCAGAAGGAACAGAAGAAGAAGGTGGAGAGAGAGACAGAGGCAGATCAAACAGATTAGCAACTG

GATTCTTGACAATTTTCTGGGAAGACCTACGGAACCTGTGCCTCTTCCTCTACCACCGCTTGAGAGACTTACT
CTTGATTGTAGGGAGGATTGTGGAAATTCTGGGACGCAGGGGGTGGGAGATCCTCAAATATTGGTGGAATCTC
CTGCAATATTGGAGCCAGGAACTAAAGAATAGTGCTGTTAGCTTGCTCAATGCCACAGCTATTGCAGTAGCTG
AGGGAACAGATAGGGTTATAGAAGTAGTGCAAAGAGCTTTTAGAGCTATTCTCCATATACCTACAAGAATAAG
ACAGGGCTTGGAAAGGGCTTTGCTATAAGATGGGTGGCAAGTGGTCAAAACATAATGGATGGCCTAAGGTAAG
GGAAAGAATAGAAAGAACTGAGCCAGCAGCAGATGGGGTGGGAGCAGTTTCTCGAGACCTGGAAAAACATGGA
GCAATCACAAGTAGCAATACAGCAACTAATAATGCTGCCTGTGCCTGGCTGGAAGCACAAGAGGAAGAGGAGG
TGGGTTTTCCAGTCAGACCTCAGGTACCTTTAAGACCAATGACTTACAAGGGAGCTTTAGATCTTAGCCACTT
TTTAAGAGAAAAGGGGGGACTGGAAGGGTTAATTTACTCCCAGAAAAGACAAGAGATCCTTGATCTATGGGTC
TACCACACACAAGGCTACTTCCCTGATTGGCAGAACTACACACCAGGGCCAGGGATCAGATATCCCCTGACCT
TTGGATGGTGCTTCAAGCTAGTACCAGTTGAGCCAGACGAGGAAGAGAACAGCAGCCTGCTACACCCTATGAG
CCAGCATGGAATGGGGGACACGGAGAAAGAAGTGTTAAAGTGGAAGTTTGACAGCCACCTAGCATTTCGTCAC
ATGGCCCGAGAGCTGCATCCGGAGTATTACAAAGACTGCTGACACCGAGCTTTCTGCAAGGGACCTTCCGCTG
GGGACTTTCCAGGGAGGCGTGGCCTGGGCGGGACTGGGGAGTGGCGAGCCCTCAGATGCTGCATATAAGCAGC
TGCTCTTTGCCTGTACTGGGTCTCTCTGGTTAGACCAGATCCGAGCCTGGGAGCTCTCTGGCTAACTAGGGAA
CCCACTGCTTAAGCCTCAATAAAGCTTGCCT

>AB-32A HIV-1 genome, derived from RNA genomic sequence
ACGCAGGACTCGGCTTGCTGAAGCGCGCACGGCAAGAGGCGAGGGGCGGCGACTGGTGAGTACGCCAAATTTT
GACTAGCGGAGGCTAGAAGGAGAGAGATGGGTGCGAGAGCGTCAGTATTAAGTGCGGGGGAATTAGATACGTG
GGAAAAAATTCGGTTAAGGCCAGGGGGAAAGAAAAAATATAGATTAAAACATATAGTATGGGCAAGCAGGGAG
CTAGAACGATTTGCAGTTAATCCTGGCCTGTTAGAAACATCAGCAGGCTGTAGACAAATACTGGGACAGCTAC
ATCCATCCCTTCAGACAGGATCAGAAGAACTTAGATCATTATATAATACAGTAGCAACCCTCTATTGTGTGCA
TCAAAAGATAGAGGTAAAAGACACCAAGGAAGCTTTAGAGAAGATAGAGGAAGAACAAAACAAAAGTAAGAAA
AAGGCACAACAAGCAGCAGCTGACACAGGAAACAGCAGCCAGGTCAGCCAAAATTACCCCATAGTACAGAACC
TCCAGGGGCAAATGGTACATCAGACCATGTCACCTAGAACTTTAAATGCATGGGTAAAAGTAATAGAAGAGAA
GGCTTTCAGCCCAGAAGTAATACCCATGTTTTCAGCATTATCAGAAGGAGCCACCCCACAAGATTTAAACACC
ATGCTAAACACAGTGGGGGGACATCAAGCAGCCATGCAAATGTTAAAAGATACCATCAATGAGGAAGCTGCGG
AATGGGATAGATTGCATCCAGTGCATGCAGGGCCTATTGCACCAGGCCAGATGAGAGAACCAAGGGGAAGTGA
CATAGCAGGAACTACTAGTACCCTTCAGGAACAAATAGGATGGATGACACATAATCCACCTATCCCAGTAGGA
GAAATTTATAAGAGATGGATAATCCTGGGATTAAATAAAATAGTAAGAATGTATAGCCCTACCAGCATTCTGG
ACATAAAACAAGGACCAAAAGAACCCTTTAGAGATTATGTAGACCGGTTCTATAAAACTCTAAGAGCCGAGCA
AGCTTCACAGGATGTAAAAAATTGGATGACAGAAACCTTGTTGGTCCAAAATGCGAATCCAGATTGTAAGACT
ATTTTAAAAGCATTGGGACCAGCAGCTACATTAGAAGAAATGATGACAGCATGTCAGGGAGTGGGGGGACCCA
GCCATAAAGCAAGAGTCTTGGCTGAAGCAATGAGCCAAGCAACAGGTTCAGCTACCATAATGATGCAGAGAGG
CAATTTTAGGAACCAAAGAAAGACTGTTAAGTGTTTCAATTGTGGCAAAGAGGGGCACATAGCCAGAAATTGC
AGGGCCCCTAGGAAAAAGGGCTGTTGGAAATGTGGAAAGGAAGGACACCAAATGAAGGATTGCACTGAGAGAC
AGGCTAATTTTTTAGGGAAGATCTGGCCTTCCCACAAGGGAAGGCCAGGGAATTTCCTTCAGAGCAGACCAGA
GCCAACAGCCCCACCAGAAGAGAGCTTCAGGTTTGGGGAAGCAACAACTCCCTCTCAGAAGCAGGAGACGATA
GACAAGGAACTGTATCCTTTAACCTCCCTCAAATCACTCTTTGGCAACGACCCCTTGTCACAGTAAGGATAGG
GGGGCAACTAAAAGAAGCTCTATTAGATACAGGAGCAGATGATACAGTATTAGAAGAAATGAATTTGCCAGGA
AGATGGAAACCAAAAATGATAGGGGGAATTGGAGGTTTTATCAAAGTAAGACAGTATGATCAGATACTCATAG
AAATCTGTGGACATAAAGCTATAGGTACAGTATTAGTAGGACCTACACCTGTCAACATAATTGGAAGGAATCT
GTTGACTCAGATTGGTTGCACTTTAAATTTTCCCATTAGTCCTATTGAAACTGTACCAGTAAAACTAAAGCCA
GGAATGGATGGCCCAAAAGTTAAACAATGGCCATTGACAGAAGAAAAAATAAAAGCATTAGTAGAAATTTGTA
CAGAAATGGAAAAGGAAGGGAAAATTTCAAAAATTGGGCCTGAAAATCCATACAATACTCCAGTATTTGCCAT
AAAGAAAAAAGACAGTACTAAATGGAGAAAATTGGTAGATTTCAGAGAACTTAATAAGAGAACTCAAGACTTC
TGGGAAGTTCAATTAGGAATACCACATCCTGCAGGGTTAAAAAAGAAAAAATCAGTAACAGTCCTGGATGTGG
GTGATGCATATTTTTCAGTCCCCTTAGATGAAAACTTTAGAAAGTATACTGCATTTACCATACCTAGTACAAA
CAATGAGACACCAGGGACTAGATATCAGTACAATGTGCTGCCACAGGGATGGAAAGGATCACCAGCAATATTC
CAAAGTAGCATGACAAAAATCTTAGAGCCTTTTAGAAAACAAAATCCAGACATAGTTATCTATCAATACATGG
ATGATTTGTATGTAGGATCCGACTTAGAAATAGGGCAGCATAGAATAAAAATAGAGGAACTGAGAGAACATCT
GTTGAGGTGGGGATTTACCACACCAGACAAAAAACATCAAAAAGAACCTCCATTCCTTTGGATGGGTTATGAA
CTCCATCCTGATAAATGGACAGTACAGCCTATAGTGCTGCCAGAGAAAGACAGCTGGACTGTCAATGACATAC
AGAAGTTAGTGGGAAAATTGAATTGGGCAAGTCAGATTTATCCAGGGATTAAAGTAAAGCAATTATGTAAGCT
CCTTAGGGGAACCAAAGCATTAACAGAAGTAATACCACTAACAGAAGAAGCAGAGCTAGAACTAGCAGAAAAC
AGGGAGATTCTAAAAGAACCAGTACATGGAGTGTATTATGACCCATCAAAAGACTTAGTAGCAGAAATACAGA
AGCAGGGGCAAGGCCAATGGACATATCAAATTTATCAAGAGCCATGTAAAAATCTGAAAACAGGAAAGTATGC
AAGAATGAGGGGTGCCCACACTAATGATATAAAACAGTTAACAGAGGCAGTGCAAAAAATAGCCACAGAAGGC
ATAATAATATGGGGAAAGACTCCTAAATTTAGACTACCCATACAAAAGGAAACATGGGAAGCATGGTGGATGG
AGTATTGGCAAGCCACCTGGATTCCTGAATGGGAATTTGTCAATACCCCTCCCTTAGTAAAATTATGGTATCA
GTTAGAGAAAGAACCCATAGAGGGAGCAGAAACCTTCTATGTAGATGGGGCAGCTAATAGGGAGACTAAATTA
GGAAAAGCAGGATATGTTACTGACAGAGGAAGACAAAAAGTTGTCTCCCTAACTGACACAACAAATCAGAAGA
CTGAGTTACAAGCAATTCATCTAGCGTTGCAGGATTCGGGACTAGAAGTAAACATAGTGACAGACTCACAATA
TGCATTAGGAATCATTCAAGCACAACCAGATAAAAGTGAATCAGAGTTAGTCAGTCAAATAATAGAGCAGTTA
ATAAAAAAGGAAAAAGTCTACCTGGCATGGGTACCAGCACACAAAGGAATTGGAGGAAATGAACAAGTAGATA
AATTAGTCAGTACTGGAATCAGGAGAGTACTATTTTTAGATGGAATAGATAAGGCCCAAGAAGAACATGAGAA
ATATCACAGTAATTGGAGAGCAATGGCTAGTGATTTTAACCTGCCACCTGTAATAGCAAAAGAGATAGTAGCC
TGCTGTGATAAATGTCAGCTAAAAGGAGAAGCCATGCATGGACAAGTAGACTGTAGTCCAGGAATATGGCAAC
TAGACTGTACACATTTAGAAGGAAAAATTGTCCTGGTAGCAGTTCATGTAGCCAGTGGATATATAGAAGCAGA
AGTCATTCCAGCAGAGACAGGGCAGGAAACAGCATACTTTCTCTTAAAATTAGCAGGAAGATGGCCAGTAAAA

ACAATACATACAGACAATGGCAGCAATTTCACCAGTAATGTGGTTAAGGCTGCCTGTTGGTGGGCAGGGATCA
AGCAGGAATTTGGCATTCCCTACAATCCCCAAAGTCAAGGAGTAGTAGAATCTATGAATAAAGAATTAAAGAA
AATTATAGGACAGGTAAGAGATCAGGCTGAACATCTTAGGACAGCAGTACAAATGGCAGTATTCATCCACAAT
TTTAAGAGAAAAGGGGGGATTGGGGGGTACAGTGCAGGGGAAAGAATAGTAGACATGATAGCAACAGACATAC
AAACTAAAGAATTACAAAAACAAATTACAAAAATTCAAAATTTTCGGGTTTATTACAGGGACAGCAGAGATCC
ACTTTGGAAAGGACCAGCAAAGCTTCTCTGGAAAGGTGAAGGGGCAGTAGTAATACAAGATAATAGTGATATA
AAAGTAGTGCCAAGAAGAAAAGCAAAGATCATTAGGGATTATGGAAAACAGATGGCAGGTGATGATTGTGTGG
CAAGTAGACAGGATGAGGATTAGAACATGGAAAAGTTTAGTAAAACACCATATGTATGTTTCACGGAAAGCTG
GGAAATGGTTTTATAGACATCACTATGAAAGCACTCATCCAAAAATAAGTTCAGAAGTACACATCCCACTAGG
GGATGCTAGATTGGTAATAACAACATATTGGGGTCTGCATACAGGAGAAAGAGACTGGCAGTTGGGCCATGGA
GTCTCCATAGAATGGAGGAAAAAGAGATATAGCACACAAGTAGACCCTGATCTAGCAGACCAACTAATCCATC
TGTATTATTTTGATTGTTTTTCAGAATCTGCTATAAGACATGCCATATTAGGACATATAGTTAGACCTAGTTG
TGAATATCAAGCAGGACATAACAAGGTAGGATCCCTACAATACTTGGCACTAACAGCACTAATAAAACCAAAG
AAGATAAAGCCACCTTTGCCTAGTGTTAAGAAACTGACAGAGGATAGATGGAACAAGCCCCAGAAGACCAAGG
GCCACAGAGGGAGCCATACAATGAATGGACGCTAGAGCTTTTAGAGGAGCTTAAGAGTGAAGCTGTTAGACAT
TTTCCTAGGGCATGGCTACATAGCTTAGGACAATATATCTATGAAACTTATGGGGATACTTGGGCAGGAGTGG
AGGCCATAATAAGAATGCTGCAACAACTGCTGTTTATTCATTTCAGAATTGGGTGTCGCCATAGCAGAATAGG
CATTATTCAACAGAGGAGAGCAAGACATGGAGCCAGTAGATCCTAGACTAGAACCCTGGAAGCATCCAGGAAG
TCAGCCTAGGACTCCTTGTACCAATTGCTATTGTAAAAAGTGTTGCCTTCATTGCCAAGTTTGTTTTATGAAA
AAAGGCTTAGGCATCTCCTATGGCAGGAAGAAGCGGAGACAGCGACGAAGACCTCCTCAAGACAGTGAGAATC
ATCAAGTTCCTCTATCAAAGCAGTAAGTAGTACATGTAATGCAACCTTTACATATAGTAGCAATAGTAGCATT
AGTAGTAGCAGCAATAATAGCAATAGTTGTGTGGACCATAGTAGGCATAGAATATAGGAAAATATTAAGACAA
AGAAAAATAGACAGGTTAATTGATAGAATAAGAGAAAGAGCAGAAGACAGTGGCAATGAAAGTGAAGGAGACC
AGGAAGAATTATCAGCACTTGTGGAGATGGGGCACCATGCTCCTTGGGATGTTGATGATCTGTAGGGCTGCAG
AACAGTTGTGGGTCACAGTCTATTATGGGGTACCTGTGTGGAAAGAAGCAAATACCACTCTATTTTGTGCATC
AGATGCTAAAGCATATGATACAGAGGTACATAATGTTTGGGCCACACATGCCTGTGTACCCACAGACCCCAAC
CCACAAGAAGTAGTATTGGAAAATGTGACAGAAGAATTTAACATGTGGAAAAATAACATGGTAGAACAGATGC
ATGAAGATATAATCAGTTTATGGGATCAAAGCCTAAAGCCATGTGTAAAATTAACCCCACTCTGTGTTACTTT
AGATTGCACTGATTTGAGGAATGCTACTAATGCCACTAATAGTAATGGGACAATAAAGGAAGAAATGAAAAAC
TGCTCTTTCAATATCACCACAAGCATAAGAGATAAGGTGCAGAAAGAATATGCACTTTTTTATAGACTTGATA
TAGTACAAATAGAGAATGATAATACTAACAATACTAACAATACTAGCTATAGGATGATAAATTGTAATACCTC
AGTCATTACACAGGCCTGTCCAAAGATATCCTTTGAGCCAATTCCCATACATTATTGTGCCCCGGCTGGTTTT
GCGATTCTAAAGTGTAACAATAAGACGTTCAATGGAAAAGGACCATGTAAAAATGTCAGCACAGTACAATGTA
CACATGGAATTAAGCCAGTAGTGTCAACTCAACTGCTGTTAAATGGCAGTCTAGCAGAAAAAGAGGTGGTAAT
TAGATCTGTCAATTTCACAGACAATGCTAAAACCATAATAGTACAGCTGAACAAATCTGTAGAAATTAATTGT
ACAAGACCCAACAACAATACAAGAAAAAGTATACATATAGGACCAGGGAGAGCATTTTATGCAACAGGAGACA
TAATAGGAGATATAAGACAAGCACATTGTAACATTAGTAGAGCAGAATGGAATAACACTTTAAGACAGATAGC
TAAAAAATTAAGAGAACAATTTGTGAATAAAACAATAGTCTTTAATCAATCCTCAGGAGGGGACCCAGAAATT
GTAATGCACAGTTTTAATTGTGGAGGGGAATTTTTCTACTGTAATTCAACACAATTGTTTAATAGTACTTGGA
ATGAAACCAACATTGACGGAAATGACACCACTAAAGGAGATAATATCACAGATGTCATCACACTCCCATGCAG
AATAAAACAAATTATAAACATGTGGCAGGAAGTAGGAAAAGCAATGTATGCCCCTCCCATCAGAGGACAAATT
AATTGTTTATCAAATATTACAGGGCTGCTATTAACAAGAGATGGTGGTAATCAGAGTGGGAACACCGAGATCT
TCAGACCTGTAGGGGGAAATATGAAGGACAATTGGAGAAGTGAATTATATAAATATAAAGTAGTACAAATTGA
ACCATTAGGAGTAGCACCCACCAAGGCAAAGAGAAGAGTGGTGCAGAGAGAAAAAAGAGCAGTGGGAACGATA
GGAGCCATGTTCCTTGGGTTCTTGGGAGCAGCAGGAAGCACTATGGGCGCAGCGTCAATGACGCTGACGGTAC
AGGCCAGACAATTATTGTCTGGTATAGTGCAACAGCAGAGCAATTTGCTGAGGGCTATTGAGGCGCAACAGCA
TATGTTGCAACTCACAGTCTGGGGCATCAAGCAGCTCCAGGCAAGAGTACTGGCTGTGGAAAGATACCTACAG
GATCAACGGCTCCTAGGGATTTGGGGTTGCTCTGGAAAACTCATCTGCACCACTGCTGTGCCTTGGAATACTA
GTTGGAGTAATAAAAATCTGACTCAGATTTGGGATAACATGACCTGGATGCAGTGGGAAAAAGAAATTAACAA
TTACACAGGAGTAATATACAACTTACTTGAAAAATCGCAGAACCAACAAGAAAAGAATGAACAAGAATTATTG
GAATTAGATGAGTGGGCAAGTTTGTGGAATTGGTTTGACATAACAAAATGGCTGTGGTATATAAAAATATTCA
TAATGATAGTAGGAGGCTTGGTAGGTTTGAGAATAGTTTTTGCTGTACTTTCTATAGTGAATAGAGTTAGGCA
GGGATACTCACCATTATCATTTCAGACCCTCCTCCCAGCCCCGAGGGGACCCGACAGGCCCGAAGGAATA?AA
GAAGAAGGTGGAGAGAGAGACAGAGGCAGATCAAATCGATTAGCAACTGGATTCTTGATACTTTTCTGGGACG

ACCTGCGGAGCCTGTGCCTCTTCAGCTACCACCGATTGAGAGACTTACTCTTGATTGTAGGGAGGATTGTGGG
AATTCTGGGACACAGGGGGTGGGAGATCCTCAAATATTGGTGGAATCTCCTGCAATATTGGAGTCAGGAACTA
AAGAATAGTGCTGTTAGCTTGCTCAATGCCACAGCTATCGCAGTAGCTGAGGGAACAGATAGGGTTATAGAAG
TAGTACGAAGAGTTTTTAGAGCTATTCTCCACATACCTACAAGAGTGAGACAGGGCTTGGAAAGGGCTTTGCT
ATAAGATGGGTGGCAAGTGGTCAAAACGTAGTCTGGGTGGATGGCCTAATGTAAGGGAAAGAATGAGAAGAAC
TGAGCCAGCAGCAGATGGGGTGGGAGCAGTATCTCGAGACCTGGAAAAACATGGGGCAATCACAAGTAGCAAT
ACAGCAACTAGTAATGCTGCCTGTGCCTGGCTAGAAGCACAAGAGGAAGAGGAGGTGGGTTTTCCAGTCAGAC
CTCAGGTACCTTTAAGACCAATGACTTACAAGGGAGCTTTAGATCTTAGCCACTTTTTAAGAGAAAAGGGGGG
ACTGGAAGGGCTAATTCACTCCCAGAAAAGACAAGAGATCCTTGATCTGTGGGTCTACCACACACAAGGCTAC
TTCCCTGATTGGCAGAACTACACACCAGGGCCAGGGATCAGATATCCCCTGACCTTTGGATGGTGCTTCAAGT
TAGTACCAGTTGAGCCAGACGAAGAAGAAAACAGCAGCTTGCTACACCCTATGAGCCAGCATGGGATGGAGGA
CACGGAGAGAGAAGTGTTAAAGTGGAAGTTTGACAGCCGCCTAGCATTTCATCACATGGCCCGAGAGCTGCAT
CCGGAGTATTACAAAGACTGCTGACACCGGGTTTTCTACAAGGGACTTTCCGCTGGGGACTTTCCAGGGAGGC
GTGGCCTGGGCGGGACTGGGGAGTGGCGAGCCCTCAGATGCTGCATATAAGCAGCTGCTTTTTGCCTGTACTG
GGTCTCTCTGGTTAGACCAGATCAGAGCCTGGGAGCTCTCTGGCTAACTAGGGAACCCACTGCTTAAGCCTCA
ATAAAGCTTGCCT

>AB-33B HIV-1 genome, derived from RNA genomic sequence
ACGCAGGACTCGGCTTGCTGAAGCGCGCACGGCAAGAGGCGAGGGGCGGCGACTGGTGAGTACGCCAAATTTT
GACTAGCGGAGGCTAGAAGGAGAGAGATGGGTGCGAGAGCGTCAGTATTAAGTGCGGGGGAATTAGATACGTG
GGAAAAAATTCGGTTAAGGCCAGGGGGAAAGAAAAAATATAGATTAAAACATATAGTATGGGCAAGCAGGGAG
CTAGAACGATTTGCAGTTAATCCTGGCCTGTTAGAAACATCAGCAGGCTGTAGACAAATACTGGGACAGCTAC
ATCCATCCCTTCAGACAGGATCAGAAGAACTTAGATCATTATATAATACAGTAGCAACCCTCTATTGTGTGCA
TCAAAAGATAGAGGTAAAAGACACCAAGGAAGCTTTAGAGAAGATAGAGGAAGAACAAAACAAAAGTAAGAAA
AAGGCACAACAAGCAGCAGCTGACACAGGAAACAGCAGCCAGGTCAGCCAAAATTACCCCATAGTACAGAACC
TCCAGGGGCAAATGGTACATCAGACCATGTCACCTAGAACTTTAAATGCATGGGTAAAAGTAATAGAAGAGAA
GGCTTTCAGCCCAGAAGTAATACCCATGTTTTCAGCATTATCAGAAGGAGCCACCCCACAAGATTTAAACACC
ATGCTAAACACAGTGGGGGGACATCAAGCAGCCATGCAAATGTTAAAAGATACCATCAATGAGGAAGCTGCGG
AATGGGATAGATTGCATCCAGTGCATGCAGGGCCTATTGCACCAGGCCAGATGAGAGAACCAAGGGGAAGTGA
CATAGCAGGAACTACTAGTACCCTTCAGGAACAAATAGGATGGATGACACATAATCCACCTATCCCAGTAGGA
GAAATTTATAAGAGATGGATAATCCTGGGATTAAATAAAATAGTAAGAATGTATAGCCCTACCAGCATTCTGG
ACATAAAACAAGGACCAAAAGAACCCTTTAGAGATTATGTAGACCGGTTCTATAAAACTCTAAGAGCCGAGCA
AGCTTCACAGGATGTAAAAAATTGGATGACAGAAACCTTGTTGGTCCAAAATGCGAATCCAGATTGTAAGACT
ATTTTAAAAGCATTGGGACCAGCAGCTACATTAGAAGAAATGATGACAGCATGTCAGGGAGTGGGGGGACCCA
GCCATAAAGCAAGAGTCTTGGCTGAAGCAATGAGCCAAGCAACAGGTTCAGCTACCATAATGATGCAGAGAGG
CAATTTTAGGAACCAAAGAAAGACTGTTAAGTGTTTCAATTGTGGCAAAGAGGGGCACATAGCCAGAAATTGC
AGGGCCCCTAGGAAAAAGGGCTGTTGGAAATGTGGAAAGGAAGGACACCAAATGAAGGATTGCACTGAGAGAC
AGGCTAATTTTTTAGGGAAGATCTGGCCTTCCCACAAGGGAAGGCCAGGGAATTTCCTTCAGAGCAGACCAGA
GCCAACAGCCCCACCAGAAGAGAGCTTCAGGTTTGGGGAAGCAACAACTCCCTCTCAGAAGCAGGAGACGATA
GACAAGGAACTGTATCCTTTAACCTCCCTCAAATCACTCTTTGGCAACGACCCCTTGTCACAGTAAGGATAGG
GGGGCAACTAAAAGAAGCTCTATTAGATACAGGAGCAGATGATACAGTATTAGAAGAAATGAATTTGCCAGGA
AGATGGAAACCAAAAATGATAGGGGGAATTGGAGGTTTTATCAAAGTAAGACAGTATGATCAGATACTCATAG
AAATCTGTGGACATAAAGCTATAGGTACAGTATTAGTAGGACCTACACCTGTCAACATAATTGGAAGGAATCT
GTTGACTCAGATTGGTTGCACTTTAAATTTTCCCATTAGTCCTATTGAAACTGTACCAGTAAAACTAAAGCCA
GGAATGGATGGCCCAAAAGTTAAACAATGGCCATTGACAGAAGAAAAAATAAAAGCATTAGTAGAAATTTGTA
CAGAAATGGAAAAGGAAGGGAAAATTTCAAAAATTGGGCCTGAAAATCCATACAATACTCCAGTATTTGCCAT
AAAGAAAAAAGACAGTACTAAATGGAGAAAATTGGTAGATTTCAGAGAACTTAATAAGAGAACTCAAGACTTC
TGGGAAGTTCAATTAGGAATACCACATCCTGCAGGGTTAAAAAAGAAAAAATCAGTAACAGTCCTGGATGTGG
GTGATGCATATTTTTCAGTCCCCTTAGATGAAAACTTTAGAAAGTATACTGCATTTACCATACCTAGTACAAA
CAATGAGACACCAGGGACTAGATATCAGTACAATGTGCTGCCACAGGGATGGAAAGGATCACCAGCAATATTC
CAAAGTAGCATGACAAAAATCTTAGAGCCTTTTAGAAAACAAAATCCAGACATAGTTATCTATCAATACATGG
ATGATTTGTATGTAGGATCCGACTTAGAAATAGGGCAGCATAGAATAAAAATAGAGGAACTGAGAGAACATCT
GTTGAGGTGGGGATTTACCACACCAGACAAAAAACATCAAAAAGAACCTCCATTCCTTTGGATGGGTTATGAA
CTCCATCCTGATAAATGGACAGTACAGCCTATAGTGCTGCCAGAGAAAGACAGCTGGACTGTCAATGACATAC
AGAAGTTAGTGGGAAAATTGAATTGGGCAAGTCAGATTTATCCAGGGATTAAAGTAAAGCAATTATGTAAGCT
CCTTAGGGGAACCAAAGCATTAACAGAAGTAATACCACTAACAGAAGAAGCAGAGCTAGAACTAGCAGAAAAC
AGGGAGATTCTAAAAGAACCAGTACATGGAGTGTATTATGACCCATCAAAAGACTTAGTAGCAGAAATACAGA
AGCAGGGGCAAGGCCAATGGACATATCAAATTTATCAAGAGCCATGTAAAAATCTGAAAACAGGAAAGTATGC
AAGAATGAGGGGTGCCCACACTAATGATATAAAACAGTTAACAGAGGCAGTGCAAAAAATAGCCACAGAAGGC
ATAATAATATGGGGAAAGACTCCTAAATTTAGACTACCCATACAAAAGGAAACATGGGAAGCATGGTGGATGG
AGTATTGGCAAGCCACCTGGATTCCTGAATGGGAATTTGTCAATACCCCTCCCTTAGTAAAATTATGGTATCA
GTTAGAGAAAGAACCCATAGAGGGAGCAGAAACCTTCTATGTAGATGGGGCAGCTAATAGGGAGACTAAATTA
GGAAAAGCAGGATATGTTACTGACAGAGGAAGACAAAAAGTTGTCTCCCTAACTGACACAACAAATCAGAAGA
CTGAGTTACAAGCAATTCATCTAGCGTTGCAGGATTCGGGACTAGAAGTAAACATAGTGACAGACTCACAATA
TGCATTAGGAATCATTCAAGCACAACCAGATAAAAGTGAATCAGAGTTAGTCAGTCAAATAATAGAGCAGTTA
ATAAAAAAGGAAAAAGTCTACCTGGCATGGGTACCAGCACACAAAGGAATTGGAGGAAATGAACAAGTAGATA
AATTAGTCAGTACTGGAATCAGGAGAGTACTATTTTTAGATGGAATAGATAAGGCCCAAGAAGAACATGAGAA
ATATCACAGTAATTGGAGAGCAATGGCTAGTGATTTTAACCTGCCACCTGTAATAGCAAAAGAGATAGTAGCC
TGCTGTGATAAATGTCAGCTAAAAGGAGAAGCCATGCATGGACAAGTAGACTGTAGTCCAGGAATATGGCAAC
TAGACTGTACACATTTAGAAGGAAAAATTGTCCTGGTAGCAGTTCATGTAGCCAGTGGATATATAGAAGCAGA
AGTCATTCCAGCAGAGACAGGGCAGGAAACAGCATACTTTCTCTTAAAATTAGCAGGAAGATGGCCAGTAAAA

ACAATACATACAGACAATGGCAGCAATTTCACCAGTAATGTGGTTAAGGCTGCCTGTTGGTGGGCAGGGATCA
AGCAGGAATTTGGCATTCCCTACAATCCCCAAAGTCAAGGAGTAGTAGAATCTATGAATAAAGAATTAAAGAA
AATTATAGGACAGGTAAGAGATCAGGCTGAACATCTTAGGACAGCAGTACAAATGGCAGTATTCATCCACAAT
TTTAAGAGAAAAGGGGGGATTGGGGGGTACAGTGCAGGGGAAAGAATAGTAGACATGATAGCAACAGACATAC
AAACTAAAGAATTACAAAAACAAATTACAAAAATTCAAAATTTTCGGGTTTATTACAGGGACAGCAGAGATCC
ACTTTGGAAAGGACCAGCAAAGCTTCTCTGGAAAGGTGAAGGGGCAGTAGTAATACAAGATAATAGTGATATA
AAAGTAGTGCCAAGAAGAAAAGCAAAGATCATTAGGGATTATGGAAAACAGATGGCAGGTGATGATTGTGTGG
CAAGTAGACAGGATGAGGATTAGAACATGGAAAAGTTTAGTAAAACACCATATGTATGTTTCACGGAAAGCTG
GGAAATGGTTTTATAGACATCACTATGAAAGCACTCATCCAAAAATAAGTTCAGAAGTACACATCCCACTAGG
GGATGCTAGATTGGTAATAACAACATATTGGGGTCTGCATACAGGAGAAAGAGACTGGCAGTTGGGCCATGGA
GTCTCCATAGAATGGAGGAAAAAGAGATATAGCACACAAGTAGACCCTGATCTAGCAGACCAACTAATCCATC
TGTATTATTTTGATTGTTTTTCAGAATCTGCTATAAGACATGCCATATTAGGACATATAGTTAGACCTAGTTG
TGAATATCAAGCAGGACATAACAAGGTAGGATCCCTACAATACTTGGCACTAACAGCACTAATAAAACCAAAG
AAGATAAAGCCACCTTTGCCTAGTGTTAAGAAACTGACAGAGGATAGATGGAACAAGCCCCAGAAGACCAAGG
GCCACAGAGGGAGCCATACAATGAATGGACGCTAGAGCTTTTAGAGGAGCTTAAGAGTGAAGCTGTTAGACAT
TTTCCTAGGGCATGGCTACATAGCTTAGGACAATATATCTATGAAACTTATGGGGATACTTGGGCAGGAGTGG
AGGCCATAATAAGAATGCTGCAACAACTGCTGTTTATTCATTTCAGAATTGGGTGTCGCCATAGCAGAATAGG
CATTATTCAACAGAGGAGAGCAAGACATGGAGCCAGTAGATCCTAGACTAGAACCCTGGAAGCATCCAGGAAG
TCAGCCTAGGACTCCTTGTACCAATTGCTATTGTAAAAAGTGTTGCCTTCATTGCCAAGTTTGTTTTATGAAA
AAAGGCTTAGGCATCTCCTATGGCAGGAAGAAGCGGAGACAGCGACGAAGACCTCCTCAAGACAGTGAGAATC
ATCAAGTTCCTCTATCAAAGCAGTAAGTAGTACATGTAATGCAACCTTTACATATAGTAGCAATAGTAGCATT
AGTAGTAGCAGCAATAATAGCAATAGTTGTGTGGACCATAGTAGGCATAGAATATAGGAAAATATTAAGACAA
AGAAAAATAGACAGGTTAATTGATAGAATAAGAGAAAGAGCAGAAGACAGTGGCAATGAAAGTGAAGGAGACC
AGGAAGAATTATCAGCACTTGTGGAGATGGGGCACCATGCTCCTTGGGATGTTGATGATCTGTAGGGCTGCAG
AACAGTTGTGGGTCACAGTCTATTATGGGGTACCTGTGTGGAAAGAAGCAAATACCACTCTATTTTGTGCATC
AGATGCTAAAGCATATGATACAGAGGTACATAATGTTTGGGCCACACATGCCTGTGTACCCACAGACCCCAAC
CCACAAGAAGTAGTATTGGAAAATGTGACAGAAGAATTTAACATGTGGAAAAATAACATGGTAGAACAGATGC
ATGAAGATATAATCAGTTTATGGGATCAAAGCCTAAAGCCATGTGTAAAATTAACCCCACTCTGTGTTACTTT
AGATTGCACTGATTTGAGGAATGCTACTAATGCCACTAATAGTAATGGGACAATAAAGGAAGAAATGAAAAAC
TGCTCTTTCAATATCACCACAAGCATAAGAGATAAGGTGCAGAAAGAATATGCACTTTTTTATAGACTTGATA
TAGTACAAATAGAGAATGATAATACTAACAATACTAACAATACTAGCTATAGGATGATAAATTGTAATACCTC
AGTCATTACACAGGCCTGTCCAAAGATATCCTTTGAGCCAATTCCCATACATTATTGTGCCCCGGCTGGTTTT
GCGATTCTAAAGTGTAACAATAAGACGTTCAATGGAAAAGGACCATGTAAAAATGTCAGCACAGTACAATGTA
CACATGGAATTAAGCCAGTAGTGTCAACTCAACTGCTGTTAAATGGCAGTCTAGCAGAAAAAGAGGTGGTAAT
TAGATCTGTCAATTTCACAGACAATGCTAAAACCATAATAGTACAGCTGAACAAATCTGTAGAAATTAATTGT
ACAAGACCCAACAACAATACAAGAAAAAGTATACATATAGGACCAGGGAGAGCATTTTATGCAACAGGAGACA
TAATAGGAGATATAAGACAAGCACATTGTAACATTAGTAGAGCAGAATGGAATAACACTTTAAGACAGATAGC
TAAAAAATTAAGAGAACAATTTGTGAATAAAACAATAGTCTTTAATCAATCCTCAGGAGGGGACCCAGAAATT
GTAATGCACAGTTTTAATTGTGGAGGGGAATTTTTCTACTGTAATTCAACACAATTGTTTAATAGTACTTGGA
ATGAAACCAACATTGACGGAAATGACACCACTAAAGGAGATAATATCACAGATGTCATCACACTCCCATGCAG
AATAAAACAAATTATAAACATGTGGCAGGAAGTAGGAAAAGCAATGTATGCCCCTCCCATCAGAGGACAAATT
AATTGTTTATCAAATATTACAGGGCTGCTATTAACAAGAGATGGTGGTAATCAGAGTGGGAACACCGAGATCT
TCAGACCTGTAGGGGGAAATATGAAGGACAATTGGAGAAGTGAATTATATAAATATAAAGTAGTACAAATTGA
ACCATTAGGAGTAGCACCCACCAAGGCAAAGAGAAGAGTGGTGCAGAGAGAAAAAAGAGCAGTGGGAACGATA
GGAGCCATGTTCCTTGGGTTCTTGGGAGCAGCAGGAAGCACTATGGGCGCAGCGTCAATGACGCTGACGGTAC
AGGCCAGACAATTATTGTCTGGTATAGTGCAACAGCAGAGCAATTTGCTGAGGGCTATTGAGGCGCAACAGCA
TATGTTGCAACTCACAGTCTGGGGCATCAAGCAGCTCCAGGCAAGAGTACTGGCTGTGGAAAGATACCTACAG
GATCAACGGCTCCTAGGGATTTGGGGTTGCTCTGGAAAACTCATCTGCACCACTGCTGTGCCTTGGAATACTA
GTTGGAGTAATAAAAATCTGACTCAGATTTGGGATAACATGACCTGGATGCAGTGGGAAAAAGAAATTAACAA
TTACACAGGAGTAATATACAACTTACTTGAAAAATCGCAGAACCAACAAGAAAAGAATGAACAAGAATTATTG
GAATTAGATGAGTGGGCAAGTTTGTGGAATTGGTTTGACATAACAAAATGGCTGTGGTATATAAAAATATTCA
TAATGATAGTAGGAGGCTTGGTAGGTTTGAGAATAGTTTTTGCTGTACTTTCTATAGTGAATAGAGTTAGGCA
GGGATACTCACCATTATCATTTCAGACCCTCCTCCCAGCCCCGAGGGGACCCGACAGGCCCGAAGGAATA?AA
GAAGAAGGTGGAGAGAGAGACAGAGGCAGATCAAATCGATTAGCAACTGGATTCTTGATACTTTTCTGGGACG

ACCTGCGGAGCCTGTGCCTCTTCAGCTACCACCGATTGAGAGACTTACTCTTGATTGTAGGGAGGATTGTGGG
AATTCTGGGACACAGGGGGTGGGAGATCCTCAAATATTGGTGGAATCTCCTGCAATATTGGAGTCAGGAACTA
AAGAATAGTGCTGTTAGCTTGCTCAATGCCACAGCTATCGCAGTAGCTGAGGGAACAGATAGGGTTATAGAAG
TAGTACGAAGAGTTTTTAGAGCTATTCTCCACATACCTACAAGAGTGAGACAGGGCTTGGAAAGGGCTTTGCT
ATAAGATGGGTGGCAAGTGGTCAAAACGTAGTCTGGGTGGATGGCCTAATGTAAGGGAAAGAATGAGAAGAAC
TGAGCCAGCAGCAGATGGGGTGGGAGCAGTATCTCGAGACCTGGAAAAACATGGGGCAATCACAAGTAGCAAT
ACAGCAACTAGTAATGCTGCCTGTGCCTGGCTAGAAGCACAAGAGGAAGAGGAGGTGGGTTTTCCAGTCAGAC
CTCAGGTACCTTTAAGACCAATGACTTACAAGGGAGCTTTAGATCTTAGCCACTTTTTAAGAGAAAAGGGGGG
ACTGGAAGGGCTAATTCACTCCCAGAAAAGACAAGAGATCCTTGATCTGTGGGTCTACCACACACAAGGCTAC
TTCCCTGATTGGCAGAACTACACACCAGGGCCAGGGATCAGATATCCCCTGACCTTTGGATGGTGCTTCAAGT
TAGTACCAGTTGAGCCAGACGAAGAAGAAAACAGCAGCTTGCTACACCCTATGAGCCAGCATGGGATGGAGGA
CACGGAGAGAGAAGTGTTAAAGTGGAAGTTTGACAGCCGCCTAGCATTTCATCACATGGCCCGAGAGCTGCAT
CCGGAGTATTACAAAGACTGCTGACACCGGGTTTTCTACAAGGGACTTTCCGCTGGGGACTTTCCAGGGAGGC
GTGGCCTGGGCGGGACTGGGGAGTGGCGAGCCCTCAGATGCTGCATATAAGCAGCTGCTTTTTGCCTGTACTG
GGTCTCTCTGGTTAGACCAGATCAGAGCCTGGGAGCTCTCTGGCTAACTAGGGAACCCACTGCTTAAGCCTCA
ATAAAGCTTGCCT

>AB-34A HIV-1 genome, derived from RNA genomic sequence
ACGCAGGACTCGGCTTGCTGAAGCGCGCACGGCAAGAGGCGAGGGGCGGCGACTGGTGAGTACGCCAATTTTT
GACTAGCGGAGGCTAGAAGGAGAGAGATGGGTGCGAGAGCGTCAGTATTAAGTGCGGGGGAATTAGATACATG
GGAAAAAATTCGGTTAAGGCCAGGAGGAAAGAAAAAATATAGATTAAAACATATAGTATGGGCAAGCAGGGAG
CTAGAACGATTTGCAGTTAATCCTGGCCTATTAGAAACATCAGCAGGATGTAGACAAATAATGGGACAGCTAC
ATCCATCCCTTCAGACAGGATCAGAAGAACTTAGGTCATTATATAATACAGTAGCAGTCCTCTATTGTGTACA
TCAAAAGATAGAGGTAAAAGACACCAAGGAAGCTTTAGAGAAGGTAGAGGAAGAGCAAAACAAAAGTAAGAAA
AAGGTACAGCAAGCGGCAGCTGACGCAGGAAACAGCAGCCCGGTCAGCCAAAATTACCCTATAGTACAGAACC
TCCAGGGGCAAATGGTACACCAGACCATGTCACCTAGAACTTTAAATGCATGGGTAAAAGTGATAGAAGAGAA
GGCTTTCAGCCCAGAAGTAATACCCATGTTTTCAGCATTATCGGAAGGAGCCACCCCACAAGATTTAAACACC
ATGCTAAACACAGTGGGGGGACATCAAGCAGCCATGCAAATGTTAAAAGATACCATCAATGAGGAGGCTGCAG
AATGGGATAGATTGCATCCAGTGCATGCAGGGCCTATTGCACCAGGCCAGATGAGAGAACCAAGGGGAAGTGA
CATAGCAGGAACTACTAGTACCCTTCAGGAACAAATAGGATGGATGACACATAATCCACCTATCCCAGTAGGA
GAAATCTATAAGAGATGGATAATCCTGGGATTAAATAAAATAGTAAGAATGTATAGCCCTACCAGCATTCTGG
ACATAAAGCAAGGACCAAAAGAACCCTTTAGAGATTATGTAGACCGGTTCTATAAAACTCTAAGAGCCGAGCA
AGCTTCACAGGATGTAAAAAATTGGATGACAGAAACCTTGTTGGTCCAAAATGCGAATCCAGATTGTAAGACC
ATTTTAAAAGCATTGGGACCAGCAGCCACATTAGAAGAAATGATGACAGCATGTCAAGGAGTGGGAGGACCCA
GCCATAAAGCAAGAGTCTTGGCTGAAGCAATGAGCCAAGCAACAGGTCCAGCTAACATAATGATGCAGAGGGG
TAATTTTAGGAACCAAAGAAAGACTGTTAAGTGTTTCAATTGTGGCAAAGAGGGGCACATAGCCAGAAATTGC
AGGGCCCCTAGGAGAAAGGGCTGTTGGAAATGTGGAAAGGAAGGACACCAAATGAAGGATTGCACTGAGAGAC
AGGCTAATTTTTTAGGGAGAATCTGGCCTTCCCACAAGGGGAGGCCAGGGAATTTTCTTCAGAGCAGACCAGA
GCCATCAGCCCCACCAGAAGAGAGCTTCAGGTTTGGGGAGGAAGCAACAACTCCCCCTCAGAAGCAGGAGACG
ATAGACAAGGAACTGTATCCTTTAACCTCCCTCAAATCCCTCTTTGGCAACGACCCATCCTCACAGTAAGGGT
AGGGGGGCACCTAATAGAAGCTCTATTAGATACAGGAGCAGATGATACAGTGTTAGAAGAAATAAATTTACCA
GGAAGATGGAAACCAAAAATGATAGGGGGAATTGGAGGTTTTGTCAAAGTAAGACAATATGAGCAGGTACCCA
TAGAAATCTGTGGGCATGAAGTTATAAGTACAGTATTAGTAGGACCTACACCTGCCAACGTAATTGGAAGAAA
TGTGATGTCTCAAATTGGTTGCACTTTAAATTTTCCCATTAGTCCTATTGAAACTGTACCAGTAAAATTAAAG
CCAGGAATGGATGGCCCAAAAGTTAAACAATGGCCATTGACAGAAGAAAAAATAAAAGCATTAGTAGAAATTT
GTACAGAATTGGAAAAGGAAGGGAAAATTTCAAAAATTGGGCCTGAAAATCCGTACAATACTCCAGTATTTGC
CATAAAGAAAAAGAACAGTACTAAATGGAGAAAATTGGTAGATTTCAGAGAACTTAATAAGAGAACTCAAGAC
TTTTGGGAAGTTCAATTGGGAATACCACATCCCGCAGGGTTAAAAAAGAAAAAATCAGTAACAGTCTTGGATG
TGGGTGATGCATATTTTTCAGTTCCCTTGGATGAAGACTTTAGAAAGTATACTGCATTCACCATACCTAGTAC
AAACAATGAGACACCAGGGATTAGATATCAATACAATGTGCTGCCACAGGGATGGAAAGGATCACCAGCAATA
TTCCAAAGTAGCATGACAAAAATCTTAGAGCCTTTTAGAAAACAAAATCCAGACATAGTTATCTATCAATACG
TGGATGATTTGTATGTAAGCTCAGACTTAGAAATAGGGCAGCATAGAAAAAAAATAGAGGACCTGAGAGAACA
TCTGTGGAGGTGGGGATTTTACACACCAGACAAAAAACATCAGAAAGAACCTCCATTCCTCTGGATGGGTTAT
GAACTCCATCCTGATGAATGGACAGTACAGCCTATAGTGCTGCCAGAAAAAGACAGCTGGACTGTCAATGACA
TACAGAAGTTAGTGGGAAAATTGAATTGGGCAAGTCAAATTTATCCAGGGATTAAAGTAAAGCAATTATGTAA
ACTCCTTAAGGGAACCAAGGCACTAACAGAGGTAGTACCACTAACAGAGGAAGCAGAGCTAGAACTAGCAGAA
AACAGGGAAATTCTAAAAGAACCAGTATATGGAGTGTATTATGACCCATCAAAAGAATTAATAGCAGAAATAC
AGAAGCAGGGACAAGGCCAATGGACATATCAAATTTATCAAGAGCAAGGTAAAAATTTGAAAACAGGAAAATA
TGCAAGAATGAGGAGTGCCCACACTAATGATATAAAACAGTTAACAGAGGCAGTGCAAAAAATAAGCATGGAA
AGCATAGTAATATGGGGGAAGACTCCTAAATTTAGACTACCCATACAAAAGGAAACATGGGAAGCATGGTGGA
TGGAGTATTGGCAAGCCACCTGGATTCCTGAGTGGGAGTTTGTCAATACCCCTCCCTTAGTAAAATTATGGTA
TCAGTTAGAGAAAGAACCCATAGTAGGAGCAGAAACCTTCTATGTAGATGGGGCAGCTAATAGGGAAACTAAA
TTAGGAAAAGCAGGATATGTTACTGACAGAGGAAGACAAAAAGTTGTCTCCCTGACTGACACAACAAATCAGA
AGACTGAGTTACAAGCAATTCATCTAGCCTTGCAGGATTCGGGATTAGAAGTAAACATAGTAACAGACTCACA
ATATGCATTAGGAATCATTCAAGCACAACCAGATCAAAGTGAATCAGAGTTAGTCAATCAAATAATAGAGCAG
TTAATAAAAAAGGAAAAAATCTACTTGGCATGGGTACCAGCACATAAAGGGATTGGAGGAAATGAGCAGGTAG
ATAAATTAGTCAGTACTGGAGTCAGGAGAGTACTATTTTTAGATGGAATAGATAAGGCCCAAGAAGAACATGA
GAAATATCACAGTAATTGGAGAGCAATGGCTAGTGATTTTAACCTGCCACCTGTAATAGCAAAAGAAATAGTA
GCCTGCTGTGATAAATGTCAGCTAAAAGGAGAAGCCATGCATGGACAAGTAGACTGTAGTCCAGGAATATGGC
AACTAGATTGTACACATTTAGAAGGAAAAATTATCCTAGTGGCAGTTCATGTAGCCAGTGGATATGTAGAAGC
AGAAGTCATTCCAGCAGAGACAGGACAGGAAACAGCATACTTTCTCTTAAAATTAGCAGGAAGATGGCCAGTA

AAAACAATACATACAGACAATGGCAGCAATTTCACCAGCAATATAGTTAAAGCTGCCTGTTGGTGGGCAGGGA
TCAAGCAGGAATTTGGCATTCCCTACAATCCCCAAAGTCAAGGGGTAGTAGAATCCATGAATAAAGAATTAAA
GAAAATTATAGGACAGGTAAGAGATCAGGCTGAGCATCTTAGGACAGCAGTACAAATGGCAGTATTCATTCAC
AATTTTAAGAGAAAAGGGGGGATTGGGGGGTACAGTGCAGGGGAAAGAATAGTAGACATAATAGCAACAGACA
TACAAACTAAAGAGTTACAAAAACAAATTACAAAAGTTCAAAATTTTCGGGTTTATTACAGGGACAGCAGAGA
TCCACTTTGGAAAGGACCAGCAAAACTTCTCTGGAAAGGTGAAGGGGCAGTAGTAATACAAGAAAATAGTGAT
ATAAAAGTAGTGCCAAGAAGAAAAGCAAAGATTATTAGGGATTATGGAAAACAGATGGCAGGTGATGATTGTG
TGGCAAGTAGACAGGATGAGGATTAGAACATGGAAAAGTTTAGTAAAACACCATATGTATGTCTCACGGAAAG
CTGGGAAATGGTTTTATAGACATCACTATGAAAGCACTCATCCAAAAATAAGTTCAGAAGTACACATCCCACT
AGGGGATGCTAGATTGGTAATAACAACATATTGGGGTCTGCATACAG??????????????????????????
???
??????????????????????????GAATCTGCTATAAGACATGCCATATTAGGACATATAGTTAGACCTAG
TTGTGAATATCAAGCAGGACATAACAAGGTAGGATCCCTACAATACTTGGCACTAACAGCACTAATAAAACCA
AAGAAGATAAAGCCACCTTTGCCTAGTGTTAAGAAACTGACAGAGGATAGATGGAACCAA?????????????
???
???
???
???
???
???
???
???
???
???
???
???
???
???
???
???
???
???AGCCAT
ACATTATTGTGCCCCGGCTGGTTTTGCGATTCTAAAGTGTAACAATAAGACATTCGATGGAAAAGGGAACTGT
ACAAATGTCAGCATAGTACAATGTACACATGGAATTAAGCCAGTAGTGTCAACTCAGCTGCTGTTAAATGGCA
GTTTAGCAGAAGAAAAGGTGGTAATTAGATCTGTCAATTTCTCAAACAATGCTAAAACCATAATAGTACAGCT
GAACACATCTGTAGGAATTAACTGTACAAGACCAAACAACAACACAGGAAAAACTATACACCTGGGATGGAGG
AGATCATTTTTTACAACAGAGACCATAATAGGAGATATAAGACGAGCACATTGTAATATTAGTAGAACAGCAT
GGAATAACACTTTAAGACAGATAGCTGGAGAGTTAAGAAAACAATTTGGGAATAAAACAATAGCCTTTAATCG
CTCCTCAGGAGGGGACCCAGAGATTGTAATGCACAGTTTTAATTGTGGAGGGGAATTTTTCTACTGTGATACA
ACACAACTGTTTAATAGTACTTGGAATGGAACCGACATTTACAGAAATCGGAATGACACTGACATTAACAGAA
ATACCACAAATAATAAGACAGAGGTCATCACACTCCCATGCAGAATAAAACAAATTGTAAACATGTGGCAGGG
AATAGGAAAAGCAATGTATGCCCCTCCCATCAGAGGACGAATTTATTGTGTATCAAATATTACAGGGCTGCTA
TTAACAAGAGATGGTGGTAATCAGAGTGGGAGCAACACCACCGAGACCTTCAGACCTCAAGGGGGAGACATGA
AGGACAATTGGAGGAGTGAATTATATAAATATAAAGTAGTACAACTTGAACCATTAGGAGTAGCACCCACCAA
GGCAAAGAGAAGAGTGGTGCAGAGAGAAAAAAGAGCAGTGGGAATGCTAGGAGCCATGTTCCTTGGGTTCTTG
GGAGCAGCAGGAAGCACTATGGGCGCAGCGTCAATGACGCTGACGGTACAGGCCAGACAATTAATGTCTGGTA
TAGTGCAACAGCAGAGCAATTTGCTGAGGGCTATTGAGGCGCAACAGCGCATGTTGCAACTCACAGTCTGGGG
CATCAAGCAGCTCCAGGCAAGAGTACTGGCTGTGGAAAGATACCTACAGGATCAACGGCTCCTAGGGATTTGG
GGTTGCTCTGGAAAGCTCATCTGCACCACTGCTGTGCCTTGGAATAGTAGTTGGAGTAATAAAAATCTGACTC
AGATTTGGGATAACATGACCTGGATGCAGTGGGAAAAAGAAATTAACAATTACACAGGAGTAATATACAACTT
ACTTGAAGAATCGCAACACCAACAAGAAAAGAATGAACAAGAATTGTTGGAATTAGATAAGTGGGACAGTTTA
TGGAATTGGTTTGACATAACACAGTGGCTGTGGTATATAAAAATATTCATAATGATAGTAGGAGGCTTGATAG
GTTTAAGAATAATTTTTGCTGTGCTTTCTATAGTGAATAAAGTTAGGCAGGGATATTCACCATTATCATTTCA
GACCCTCCTCCCGGTCCCGAGGGGACCCGACGGGCCCGAAGGAACAGAAGAAGAAGGTGGAGAGAGAGACAGA

GGCAGATCAAACAGATTAGCAACTGGATTCTTGACAATTTTCTGGGAAGACCTACGGAACCTGTGCCTCTTCC
TTTACCACCGCTTGAGAGACTTACTCTTGATTGTAGGGAGGATTGTGGAAATTCTGGGACGCAGGGGGTGGGA
GATCCTCAAATATTGGTGGAACCTCCTGCAATATTGGAGTCAGGAACTAAAGAATAGTGCTGTTAGCTTGCTC
AATGCCACAGCTATTACAGTAGCTGAGGGAACAGATAGGGTTATAGAAGTAGTGCAAAGAGCTTTTAGAGCTA
TTCTCCATATACCTACAAGAATAAGACAGGGCTTGGAAAGGGCTTTGCTATAAGATGGGTGGTAAGTGGTCAA
AACATCTGAGTGGGTGGCCTAAGGTAAGGGAAAGAATAGAAAGAACTGAGCCAGCAGCAGATGGGGTGGGAGC
AGTTTCTCGAGACCTGGAAAAACATGGAGCAATCACAAGTAGCAATACAGCAACCACTAATGCTGCCTGTGCC
TGGCTAGAAGCACAAGAAGAAGAGGAGGTGGGTTTTCCAGTCAGACCTCAGGTACCTTTAAGACCCATGACTT
ACAAGGGAGCTTTAGATCTTAGCCACTTTTTAAGAGAAAAGGGGGGACTGGAAGGGTTAATTTACTCCCAGAA
AAGACAAGAGATCCTTGATCTGTGGGTCTACCACACACAAGGCTACTTCCCTGATTGGCAGAACTACACACCA
GGGCCAGGGATCAGATATCCCCTGACCTTTGGATGGTGCTTCAAGTTAGTACCAGTTGAGCCAGACGAGGAAG
AGAACAGCAGCCTGCTACACCCTATGAGCCAGCATGGAATGGGGGACACGGAGAAAGAAGTGTTAAAGTGGAA
GTTTGACAGCCACCTAGCATTTCGTCACATGGCCCGAGAGCTGCATCCGGAGTATTACAAAGACTGCTGACAC
CGAGCTTTCTGCAAGGGACTTTCCGCTGGGGACTTTCCAGGGAGGCGTGGCCTGGGCGGGACTGGGGAGTGGC
GAGCCCTCAGATGCTGCATATAAGCAGCTGCTTTTTGCCTGTACTGGGTCTCTCTGGTTAGCCCAGATCCGAG
CCTGGGAGCTCTCTGGCTAACTAGGGAACCCACTGCTTAAGCCTCAATAAAGCTTGCC

>AB-34B HIV-1 genome, derived from RNA genomic sequence
ACGCAGGACTCGGCTTGCTGAAGCGCGCACGGCAAGAGGCGAGGGGCGGCGACTGGTGAGTACGCCAATTTTT
GACTAGCGGAGGCTAGAAGGAGAGAGATGGGTGCGAGAGCGTCAGTATTAAGTGCGGGGGAATTAGATACATG
GGAAAAAATTCGGTTAAGGCCAGGAGGAAAGAAAAAATATAGATTAAAACATATAGTATGGGCAAGCAGGGAG
CTAGAACGATTTGCAGTTAATCCTGGCCTGTTAGAAACATCAGCAGGATGTAGACAAATACTGGGACAGCTAC
ATCCATCCCTTCAGACAGGATCAGAAGAACTTAGGTCATTATATAATACAGTAGCAACCCTCTATTGTGTACA
TCAAAAGATAGAGGTAAAAGACACCAAGGAAGCTTTAGAGAAGGTAGAGGAAGAGCAAAACAAAAGTAAGAAA
AAAGCACAGCAAGCGGCAGCTGACACAGGAAACAGCAGCCCGGTCAGCCAAAATTACCCTATAGTACAGAACC
TCCAGGGGCAAATGGTACACCAGACCATGTCACCTAGAACTTTAAATGCATGGGTAAAAGTGATAGAAGAGAA
GGCTTTCAGCCCAGAAGTAATACCCATGTTTTCAGCATTATCAGAAGGAGCCACCCCACAAGATTTAAACACC
ATGCTAAACACAGTGGGGGGACATCAAGCAGCCATGCAAATGTTAAAAGATACCATCAATGAGGAAGCTGCAG
AATGGGATAGATTGCATCCAGTGCATGCAGGGCCTATTGCACCAGGCCAGATGAGAGAACCAAGGGGAAGTGA
CATAGCAGGAACTACTAGTACCCTTCAGGAACAAATAGGATGGATGACACATAATCCACCTATCCCAGTAGGA
GAAATCTATAAGAGATGGATAATCCTGGGATTAAATAAAATAGTAAGAATGTATAGCCCTACCAGCATTCTGG
ACATAAAACAAGGACCAAAAGAACCCTTTAGAGATTATGTAGACCGGTTCTATAAAACTCTAAGAGCCGAGCA
AGCTTCACAGGATGTAAAAAATTGGATGACAGAAACCTTGTTGGTCCAAAATGCGAATCCAGATTGTAAGACC
ATTTTAAAAGCATTGGGACCAGCAGCTACATTAGAAGAAATGATGACAGCATGTCAGGGAGTGGGAGGACCCA
GCCATAAAGCAAGAGTCTTGGCTGAAGCAATGAGCCAAGCAACAGGTTCAGCTAACATAATGATGCAGAGAGG
CAATTTCAGGAACCAAAGAAAGACTGTTAAGTGTTTCAATTGTGGCAAAGAGGGGCACATAGCCAGAAATTGC
AGGGCCCCTAGGAAAAAGGGCTGTTGGAAATGTGGAAAGGAAGGACACCAAATGAAGGATTGCACTGAGAGAC
AGGCTAATTTTTTAGGGAAAATCTGGCCTTCCCACAAGGGGAGGCCAGGGAATTTTCTTCAGAGCAGACCAGA
ACCATCAGCCCCACCAGAAGAGAGCTTCAGGTTTGGGGAAGCAGCAACAACTCCCTCTCAGAAACAGGAGACG
ATAGACAAGGAACTGTATCCTTTAACCTCCCTCAAATCACTCTTTGGCAACGACCCATCGTCACAGTAAGGGT
AGGGGGGCAACTAATAGAAGCTCTATTAGATACAGGAGCAGATGATACAGTATTAGAAGAAATAAATTTACCA
GGAAGATGGAAACCAAAAATGATAGGGGGAATTGGAGGTTTTGTCAAAGTAAGACAGTATGATCAGGTACCCA
TAGAAATCTGTGGGCATAAAGTTATAGGTACAGTATTAGTAGGACCTACACCTACCAACGTAATTGGAAGAAA
TGTGATGACTCAAATTGGTTGCACTTTAAATTTTCCCATTAGTCCTATTGAAACTGTACCAGTAAAATTAAAG
CCAGGAATGGATGGCCCAAAAGTTAAACAATGGCCATTGACAGAAGAAAAAATAAAAGCATTAGTAGAAATTT
GTACAGAATTGGAAAAGGAAGGAAAAATTTCAAAAATTGGGCCTGAAAATCCATACAATACTCCAGTATTTGC
CATAAAGAAAAAAGACAGTACTAAATGGAGAAAATTGGTAGATTTCAGAGAACTTAATAAGAGAACTCAAGAC
TTCTGGGAAGTTCAATTGGGAATACCACATCCCGCAGGGTTAAAAAAGAAAAAATCAGTAACAGTCTTGGATG
TGGGTGATGCATATTTTTCAGTTCCCTTGGATGAAGACTTTAGAAAGTATACTGCATTCACCATACCTAGTAC
AAACAATGAGACACCAGGGACTAGATATCAGTACAATGTGCTGCCACAGGGATGGAAAGGATCACCAGCAATA
TTCCAAAGTAGCATGACAAAAATCTTAGAGCCTTTTAGAAAACAAAATCCAGACATAGTTATCTATCAATACG
TGGATGATTTGTATGTAGGATCAGACTTAGAAATAGGGCAGCATAGGATAAAAATAGAGGAACTGAGAGAACA
TCTGTTGAGGTGGGGATTTTACACACCAGACAAAAAACATCAGAAAGAACCTCCATTCCTCTGGATGGGTTAT
GAACTCCATCCTGATAAATGGACAGTACAGCCTATAGTGCTGCCAGAAAAAGACAGCTGGACTGTCAATGACA
TACAGAAGTTAGTGGGAAAATTGAATTGGGCAAGTCAGATTTATCCAGGGATTAAAGTAAAGCAATTATGTAA
GCTCCTTAGGGGAACCAAGGCACTAACAGAGGTAGTACCACTAACAGAGGAAGCAGAGCTAGAACTAGCAGAA
AACAGGGAAATTCTAAAAGAACCAGTACATGGAGTGTATTATGACCCATCAAAAGACTTAGTAGCAGAAATAC
AGAAGCAGGGACAAGGCCAATGGACATATCAAATTTATCAAGAGCAAGGTAAAAATTTGAAAACAGGAAAGTA
TGCAAGAATGAGGGGTGCCCACACTAATGATATAAAACAGTTAACAGAGGCAGTGCAAAAAATAAGCATGGAA
AGCATAGTAATATGGGGAAAGACTCCTAAATTTAAACTACCCATACAAAAGGAAACATGGGAAGCATGGTGGA
TGGAGTATTGGCAAGCCACCTGGATTCCTGAGTGGGAGTTTGTCAATACCCCTCCCTTAGTAAAATTATGGTA
TCAGTTAGAGAAAGAACCCATAGTAGGAGCAGAAACTTTCTATGTAGATGGGGCAGCTAATAGGGAAACTAAA
TTAGGAAAAGCAGGATATGTTACTGACAGAGGAAGACAAAAAGTTGTCTCCCTGACTGACACAACAAATCAGA
AGACTGAGTTACAAGCAATTCATCTAGCCTTGCAGGATTCGGGATTAGAAGTAAACATAGTAACAGACTCACA
ATATGCATTAGGAATCATTCAAGCACAACCAGATCAAAGTGAATCAGAGTTAGTCAATCAAATAATAGAGCAG
TTAATAAAAAAGGAAAAAATCTACTTGGCATGGGTACCAGCACATAAAGGGATTGGAGGAAATGAGCAGGTAG
ATAAATTAGTCAGTACTGGAGTCAGGAAAGTACTATTTTTAGATGGAATAGATAAGGCCCAAGAAGAACATGA
GAAATATCACAGTAATTGGAGAGCAATGGCTAGTGATTTTAACCTGCCACCTGTAATAGCAAAAGAAATAGTA
GCCTGCTGTGATAAATGTCAGCTAAAAGGAGAAGCCATGCATGGACAAGTAGACTGTAGTCCAGGAATATGGC
AACTAGATTGTACACATTTAGAAGGAAAAATTATCATAGTAGCAGTTCATGTAGCCAGTGGATATGTAGAAGC
AGAAGTCATTCCAGCAGAGACAGGACAGGAAACAGCATACTTTCTCTTAAAATTAGCAGGAAGATGGCCAGTA

AAAACAATACATACAGACAATGGCAGCAATTTCACCAGTAATGTGGTTAAGGCTGCCTGTTGGTGGGCAGGGA
TCAAGCAGGAATTTGGCATTCCCTACAATCCCCAAAGTCAAGGAGTAGTAGAATCCATGAATAAAGAATTAAA
GAAAATTATAGGACAGGTAAGAGATCAGGCTGAACATCTTAAGACAGCAGTACAAATGGCAGTATTCATCCAC
AATTTTAAGAGAAAAGGGGGGATTGGGGGGTACAGTGCAGGGGAAAGAATAGTAGACATAATAGCAACAGACA
TACAAACTAAAGAGTTACAAAAACAAATTACAAAAATTCAAAATTTTCGGGTTTATTACAGGGACAGCAGAGA
TCCACTTTGGAAAGGACCAGCAAAGCTTCTCTGGAAAGGTGAAGGGGCAGTAGTAATACAAGATAATAGTGAT
ATAAAAGTAGTGCCAAGAAGAAAAGCAAAGATCATTAGGGATTATGGAAAACAGATGGCAGGTGATGATTGTG
TGGCAAGTAGACAGGATGAGGATTAGAACATGGAAAAGTTTAGTAAAACACCATATGTATGTCTCACGGAAAG
CTGGGAAATGGTTTTATAGACATCACTATGAAAGCACTCATCCAAAAATAAGTTCAGAAGTACACATCCCAAT
AGGGGATGCTAGATTGGTAATAACAACATATTGGGGTCTGCAAACAGGAGAAAGAGACTGGCAATTGGGCCAT
GGAGTCTCCATAGAATGGAGGAAAGGGAGATATAGCACACAAGTAGACCCTGATCTAGCAGACCAACTAATCC
ATCTGTATTATTTTGATTGTTTTTCAGAATCTGCTATAAGACATGCCATATTAGGACATATAGTTAGACCTAG
TTGTGAATATCAAGCAGGACATAACAAGGTAGGATCCCTACAATACTTGGCACTAGCAGCATTAATAAAACCA
AAGAAGATAAAGCCACCTTTGCCTAGTGTTAAGAAACTGACAGAGGATAGATGGAACAAGCCCCGGAAGACCA
AGGGCCACAGAGGGAGCCATACAATGAATGGGCACTAGAGCTTTTAGAGGAGCTTAAGAGTGAAGCTGTTAGA
CATTTTCCTAGGGCATGGCTACATAGCTTAGGACAATATATCTATGAAACTTATGGGGACACTTGGGCAGGAG
TGGAGGCCATAATAAGAATACTGCAACAACTGCTGTTTATTCATTTCAGAATTGGGTGTCGCCATAGCAGAAT
AGGCATTATTCGACAGAGGAGAGCAAGAAATGGAGCCAGTAGATCCTAGACTAGAGCCCTGGAAGCATCCAGG
AAGTCAGCCTAGGACTCCTTGTACCAATTGCTATTGTAAAAAGTGTTGCCTTCATTGCCAAGTTTGTTTTATG
AAAAAAGGCTTAGGCATCTCCTATGGCAGGAAGAAGCGGAGACAGCGACGAAGACCTCCTCCAGACAGTAAGA
ATCATCAAGTTCCTCTATCAAAGCAGTAAGTAGTACATGTAATGCAACCTTTAAATACAGTAGCAATAGTAGC
ATTAGTAGTAGCAGCAATAATAGCAATAGTTGTGTGGACCATAGTAGGCATAGAATATAGGAAAATATTAAGA
CAAAGAAAAATAGATAGGTTAATTGATAGAATAAGAGAAAGAGCAGAAGACAGTGGCAATGAAAGCGAAGGAG
ACCAGGAAGAATTATCAGCACTTGTGGAAATGGGGCACGATGCTCCTTGGGATGTTGATGATCTGTAGTGCTG
CAGAACAATTGTGGGTCACAGTCTATTATGGGGTACCTGTGTGGAAAGACGCAAATACCACTCTATTTTGTGC
ATCAGATGCTAAAGCATATGAGACAGAGGCACATAATGTTTGGGCCACACATGCCTGTGTACCCACAGACCCC
AACCCACAAGAAGTAGTATTGGAAAATGTGACAGAGGAATTTAATATGTGGGAAAACAACATGGTAGAACAGA
TGCATGAAGATATAATCAGTTTATGGGATCAAAGCCTAAAGCCATGTGTAAAATTAACCCCGCTCTGTGTCAC
TTTAAATTGCACTGATCTGGGGAATGTTACTAATACCACTAATAGTACTACTAATGAGACAATAAAGGAAGAA
ATGAAAAACTGCTCTTTCAATATTACCACAAGCATAAGAGATAAAGTGCAGAAAGAATATGCACTTTTTTATA
AACTTGATATAGTAGAAATAAAGGATGATAATACTAACAATACTGACAATACTAGCTATATAATGAGACATTG
TAATGCCTCAGTCATTACACAGGCCTGTCCAAAGACATCCTTTGAGCCAATTCCCATACATTATTGTGCCCCG
GCTGGTTTTGCGATTCTAAAGTGTAACAATAAGACATTCGATGGAAAAGGGAACTGTACAAATGTCAGCACAG
TACAATGTACACATGGAATTAAGCCAGTAGTGTCAACTCAGCTGCTGTTAAATGGCAGTTTAGCAGAAGAAGA
GGTGGTAATTAGATCTGTCAATTTCTCAAACAATGCTAAAACCATAATAGTACAGCTGAACAAATCTGTAGAA
ATTAACTGTACAAGACCCAACAACAATACAGGAAAAACTATACATATGGGATGGAGGAGATCATTTTATACAA
CAGAGACCATAATAGGAGATATAAGACGAGCACATTGTAACATTAGTAGAACAGCATGGAATAACACTTTAAG
ACAGATAGCTGGAGAATTAAGAAAACAATTTGGGAATAAACCAATAGTCTTTAATCACTCCTCAGGAGGGGAC
CCAGAAATTGTAATGCATAGTTTTAATTGTGGAGGGGAATTTTTCTACTGTGATACAACACAACTGTTTAATA
GTACTTGGAATGAAACCGACATTAACAGAAATAACACAAGTGAAGGAAATAAGACAGATGTCATCACACTCCC
ATGCAGAATAAAACAAATTATAAACATGTGGCAGGGAATAGGAAAAGCAATGTATGCCCCTCCCATCAGAGGA
CGAATTAATTGTGTATCAAATATTACAGGGCTGCTATTAACAAGAGATGGTGGTAATCAGAGTGAACAGCGCA
GCGAAACCTTCAGACCTCAAGGGGGAGACATGAAGGATAATTGGAGAAGCGAATTATATAAATATAAAGTAGT
ACAACTTGAACCATTAGGAGTAGCACCCACCAAGGCAAAGAGAAGAGTGGTGCAGAGAGAAAAAAGAGCAGTG
GGACTGTTAGGAGCCATGTTCCTTGGGTTCTTGGGAGCAGCAGGAAGCACTATGGGCGCAGCGTCAATGACGC
TGACGGTACAGGCCAGACAATTAATGTCTGGTATAGTGCAACAGCAGAGCAATTTGCTGAGGGCTATTGAGGC
GCAACAGCGTATGTTGCAACTCACAGTCTGGGGCATCAAGCAGCTCCAGGCGAGAGTACTAGCTGTGGAAAGA
TACCTACAGGATCAACGGCTCCTAGGGATTTGGGGATGCTCTGGAAAGCTCATCTGCACCACTGCTGTGCCTT
GGAATAGTAGTTGGAGTAATAAAAATCTGACTCAGATTTGGGATAACATGACCTGGATGCAGTGGGAAAAAGA
AATTAACAATTACACAGGAGTAATATACAACTTACTTGAAAAATCGCAAAACCAACAAGAAAAGAATGAACAA
GAATTGTTGGAATTAGATAAGTGGGACAGTTTATGGAATTGGTTTGACATAACAAAATGGCTGTGGTATATAA
AAATATTCATAATGATAGTAGGAGGCTTGATAGGTTTAAGAATAATTTTTGCTGTGCTTTCTATAGTGAATAA
AGTTAGGCAGGGATACTCACCATTATCATTTCAGACCCTCCTCCCAGTCCCGAGGGGACCCGACGGCCCGAA
GGAACAGAAGAAGAAGGTGGAGAGAGAGACAGAGGCAGATCAAATCGATTAGCAACTGGATTCTTGACAATTT

TCTGGGAAGACCTACGAAGCCTGTGCCTCTTCCTCTACCACCGCTTGAGAGACTTACTCTTGATTGTAGGGAG
GATTGTGGAAATTCTGGGACGCAGGGGGTGGGAGATCCTCAAATATTGGTGGAATCTCCTGCAATATTGGAGT
CAGGAACTAAAGAATAGTGCTGTTAGCTTGCTCAATGCCACAGCTATTGCAGTAGCTGAGGGAACAGATAGGG
TTATAGAAGTAGTGCAAAGAGCTTTTAGAGCTATTCTCCATATACCTACAAGAATAAGACAGGGCTTGGAAAG
GGCTTTGCTATAAGATGGGTGGCAAGTGGTCAAAACATAATGGATGGCCTAAGGTAAGGGAAAGAATAGAAAG
AACTGAGCCAGCAGCAGATGGGGTGGGAGCAGTTTCTCGAGACCTGGAAAAACATGGAGCAATCACAAGTAGC
AATACAGCAACTAATAATGCTGCCTGTGCCTGGCTAGAAGCACAAGAGGAAGAGGAGGTGGGTTTTCCAGTCA
GACCTCAGGTACCTTTAAGACCAATGACTTACAAGGGAGCTTTAGATCTTAGCCACTTTTTAAGAGAAAAGGG
GGGACTGGAAGGGTTAATTTACTCCCAGAAAAGACAAGAGATCCTTGATCTGTGGGTCTACCACACACAAGGC
TACTTCCCTGATTGGCAGAACTACACACCAGGGCCAGGGATCAGATATCCCCTGACCTTTGGATGGTGCTTCA
AGTTAGTACCAGTTGAGCCAGACGAGGAAGAGAACAGCAGCCTGCTACACCCTATGAGCCAGCATGGAATGGA
GGACACGGAGAAAGAAGTGTTAAAGTGGAAGTTTGACAGCCACCTAGCATTTCGTCACATGGCCCGAGAGCTG
CATCCGGAGTATTACAAAGACTGCTGACACCGAGTTTTCTACAAGGGACTTTCCGCTGGGGACTTTCCGGGGA
GGCGTGGCCTGGGCGGGACTGGGGAGTGGCGAGCCCTCAGATGCTGCATATAAGCAGCTGCTTTTTGCCTGTA
CTGGGTCTCTCTGGTTAGACCAGATCCGAGCCTGGGAGCTCTCTGGCTAACTAGGGAACCCACTGCTTAAGCC
TCAATAAAGCTTGCCT

```
>AE-A7 HIV-1 genome, derived from RNA genomic sequence
ACTCGGCTTGCTGAAGTGCACGCAGCAAGAGGCGAGAGCGGCGACTGGTGAGTACGCCAAATTTCGACTAGCG
GAGGCTAGAAGGAGAGAGATGGGTGCGAGAGCGTCGATACTAAGTGGGGGAAAATTAGATGCATGGGAGAAAA
TTCGGTTAAGGCCAGGGGGAAAGAAAAAATATCGACTAAAACATTTAGTATGGGCAAGCAGGGAGTTGGAAAG
ATTTGCACTTAATCCTAGTCTTTTAGAAACAGCAGAAGGATGTCAACAACTGATAGAACAGTTACAATCAACT
CTCAGGACAGGATCAGAAGAACTTAAATCATTATTTAATACAATAGCAACCCTTTGGTGCGTACATCAAAAGA
TAGACATAAAAGACACCAAGGAAGCCTTAGATAAAATAGAGGAAGAACAAAATAGGAGCAAGCAAAAGACACA
GCAAAAAACACAGCAGGCAGCAGCTGCCGCAGGAAGCAGCAGCCAAAATTACCCTATAGTGCAAAATGCACAA
GGGCAAATGATACATCAGGCCATGTCACCTAGAACTTTGAACGCATGGGTGAAGGTAGTAGAGGAGAAGGCTT
TCAGCCCAGAAGTAATACCCATGTTTACAGCATTATCAGAAGGAGCCACCCCACAAGACTTAAATATGATGCT
AAACATAGTGGGGGGACACCAGGCAGCAATGCAGATGTTAAAAGATACCATCAATGAGGAAGCTGCAGAATGG
GACAGGACACATCCTGTACATGCAGGGCCTGCTCCACCAGGCCAGATAAGAGAACCAAGGGGAAGTGACATAG
CAGGAACTACTAGTACCCTTCAAGAACAAATAGGATGGATGACAAGCAATCCACCTATCCCAGTGGGAGAAAT
CTATAAAAGATGGATAGTCCTGGGATTAAATAAAATAGTAAGAATGTATAGCCCTGTCAGCATTTTGGATATA
AGACAAGGACCAAAAGAACCCTTTAGAGACTATGTAGATAGGTTCTATAAAACTTTAAGAGCTGAACAAGCTA
CACAGGAGGTAAAAAACTGGATGACAGAAACCTTGCTGGTCCAAAATGCGAATCCAGATTGTAAGACCATTCT
AAGAGGATTAGGACCAGGGGCTACATTAGAAGAAATGATGACAGCATGTCAGGGAGTGGGAGGACCTGGCCAT
AAAGCAAGGGTTTTGGCTGAGGCTATGAGTCAAGCGCAACAGTCCAACATAATGATGCAGAAGGGAAACTTTA
GGGGCCAGAGAACAATAAAGTGTTTCAATTGTGGCAAAGAAGGACACCTAGCCAGAAACTGCAAGGCCCCTAG
GAAAAGGGGTTGTTGGAAATGTGGTAAGGAAGGACACCAAATGAAAGACTGTACTGAAAGACAGGCTAATTTT
TTAGGGAAAATTTGGCCTTCCAACAAGGGGAGGCCAGGAAATTTTCCTCAGAGCAGACCGGAACCAAC??CCT
CCTCTCCGAAACAGGAACCGAGGGACAAGGGACTATATCCTCCTTTAACCTCCCTCAAATCACTCTTTGGCAA
CGACCAGT?GTCACAGTAAGAATAGAGGGAGAGGTAATAGAAGCCCTATTAGACACAGGGGCAGATGATACAG
TAATAGAAAAGATAAATTTACCAGGAAAATGGAAACCAAAAATGATAGGGGGAATTGGAGGTTTTATCAAAGT
AAGACAGTATGATCAAATACCCATAGAAATCTGCGGACACAAAGCTGTAGGTACAGTATTAGTAGGACCGACA
CCTGTCAACATAATTGGAAGAAATCTGTTGACTCAGATTGGCTGCACTTTAAATTTTCCTATTAGTCCTATTG
AAACTGTACCAGTAAAATTAAAGCCAGGAATGGATGGGCCAAAGGTTAAACAATGGCCATTGACAGAAGAAAA
AATAAAAGCATTAGTAGAAATTTGTACAGAAATGGAAAAGGAAGGAAAAATTTCAAAAATTGGGCCTGAAAAT
CCATACAATACTCCAGTATTTGCCATAAAGAAAAAAGACAGTACTAAATGGAGAAAATTAGTAGACTTTAGAG
AACTTAATAAGAGAACTCAAGACTTCTGGGAAGTTCAATTAGGAATACCACATCCTGCAGGGTTAAAAAAGAA
AAAATCAGTAACAGTACTGGATGTGGGTGATGCATACTTTTCAGTTCCATTAGATAAAGAATTCAGGAAGTAT
ACTGCATTTACCATACCTAGTATAAACAATGAGACACCAGGGATTAGATATCAGTACAATGTGCTTCCACAGG
GATGGAAAGGATCCCCAGCCATATTCCAAAGTAGTATGACAAAAATCTTAGAGCCTTTTAGAAAACAAAACCC
AGAAATGGTTATCTATCAATACATGGATGATTTATATGTAGGATCTGACTTAGAAATAGGGCAGCATAGAGCA
AAAATAGAGGAACTGAGACAGCATCTGTTGAGGTGGGGATTTACCACACCAGACAAAAAGCATCAGAAAGAAC
CTCCATTCCTTTGGATGGGTTATGAGCTCCATCCTGATAAATGGACAGTACAGCCTATAAAGCTGCCAGAAAA
AGACAGCTGGACTGTCAATGACATACAGAAGTTAGTGGGAAAATTGAATTGGGCAAGTCAGATCTATCCAGGG
ATTAAAGTAAGGCAATTATGTAAACTCATTAGGGGAACCAAAGCACTAACAGAAGTAGTACCACTAACAGCAG
AAGCAGAGCTAGAATTGGCAGAAAACAGGGAGATACTAAAAGAACCAGTACATGGAGTGTATTATGACCCATC
AAAAGACTTAATAGCAGAAATACAGAAGCAGGGGCAAGGCCAGTGGACATATCAAATTTATCAAGAGCCATTT
AAAAATTTGAAAACAGGAAAGTATGCAAGAATGAAGGGTGCCCACACTAATGATGTAAAACAGTTAACAGAGG
CAGTGCAAAAAATAGCCACAGAAAGCATAGTAATATGGGGAAAAACTCCTAAATTTAGATTACCCATACAAAA
AGAAACATGGGAAGCATGGTGGACAGAGTATTGGCAAGCCACCTGGATTCCTGAGTGGGAATTTGTCAATACC
CCTCCCTTAGTAAAATTATGGTACCAGCTAGAGAAAGAACCCATAATAGGAGCAGAAACTTTCTATGTAGATG
GGGCAGCTAATAGGGAAACTAAATTAGGAAAAGCAGGATATGTTACTGACAGAGGAAGACAAAAGGTTGTCTC
CCTAACTGACACAACAAATCAGAAGACTGAGTTACAAGCGATCCATCTAGCTTTGCAGGATTCGGGGCTAGAA
GTAAACATAGTAACAGACTCACAATATGCATTAGGAATCATTCAAGCACAACCAGATAAGAGTGAATCAGAGT
TAGTCAGTCAGATAATAGAGCAGTTAATAAAAAAGGAAAAGGTCTATTTAGCATGGGTACCAGCACACAAGGG
AATTGGAGGAAATGAACAAGTAGATAAATTAGTCAGTGCTGGAATCAGGAAAGTACTATTTTTGGATGGAATA
GATAAGGCCCAAGAAGAACATGAGAAATATCACAATAATTGGAGAGCAATGGCTAGTGATTTTAACCTGCCAC
CTGTAGTAGCAAAAGAAATAGTAGCCAGCTGTGATAAATGTCAGTTAAAAGGAGAAGCCATGCATGGACAAGT
AGACTGTAGTCCAGGAATATGGCAATTAGATTGCACACATTTAGAAGGAAAAATTATCCTGGTAGCAGTCCAT
GTAGCCAGTGGATATATAGAAGCAGAAGTTATCCCAGCAGAGACAGGGCAGGAAACAGCATACTTTATCTTAA
AATTAGCAGGAAGATGGCCAGTAAAAACAATACATACAGACAATGGCAGCAATTTTATCAGTAATACAGTTAA
```

GGCTGCCTGTTGGTGGGCGGGGATCAAGCAAGAATTTGGCATTCCCTACAATCCCCAAAGTCAAGGAGTAGTA
GAATCTATGAATAAAGAATTAAAGAAGATTATAGGACAGGTAAGAGATCAGGCTGAACATCTTAAAACAGCAG
TACAAATGGCAGTATTCATCCACAATTTTAAAAGAAAAGGGGGGATTGGGGGATACAGTGCAGGGGAAAGAAT
AGTAGACATAATAGCAACAGACATACAAACTAAAGAACTACAAAAACAAATTACAAAAATTCAAAATTTTCGG
GTTTATTACAGGGACAACAGAGATCCACTTTGGAAAGGACCAGCAAAGCTTCTTTGGAAAGGTGAAGGGGCAG
TAGTAATACAAGAGGATAGTGAAATAAAAGTAGTGCCAAGAAGAAAAGCAAAGATCATTAGGGATTATGGAAA
ACAGATGGCAGGTGATGATTGTGTGGCAAGTAGACAGGATGAAGATTAGAACATGGAATAGTTTAGTAAAACA
CCATATGTATATTTCAAGGAAAGCTCAGGGATGGGTTTATAGGCATCACTATGAAAGCACTAATCCAAGAGTA
AGCTCAGAAGTACACATCCCACTAGGGGATGATAAATTGGTGATAACAACATATTGGGGTCTGCATGCAGGAG
AAAGAGAGTGGCATTTGGGTCATGGAGCCTCCATAGAATGGAGGAAAAGAAGATATAGCACACAAGTAGACCC
TGACCTAGCAGACCAACTAATTCATCTGCATTACTTTGATTGTTTTTCAGAATCTGCTATAAGACATGCCATA
TTAGGACGTATAGTTAGTCCTAGGTGTGAATATCAAGCAGGACATAACAAGGTAGGATCTCTACAGTACTTGG
CACTAGCAGCATTAATAAAACCAAAAAAGAGAAAGCCACCTTTGCCTAGTGTTAGGAAACTGACAGAGGATAG
ATGGAACAAGCCCCAGAAGACCATGGGCCACAGAGGGAGCCATACAATGAATGGACACTAGAACTTTTAGAGG
AACTTAAGAATGAAGCTGTTAGACATTTTCCTAGGCCATGGCTCCATAGCTTAGGACAATATATCTATGAAAC
TTATGGGGATACTTGGACAGGAGTGGAAGCCATAATAAGAATTCTGCAACAACTGCTGTTTATTCATTTCAGA
ATTGGATGTCAACATAGCAGAATAGGTATTCTACCAAGGAGAGCAAGGAATGGAGCCAGTAGATCCTAGTTTA
GAGCCCTGGAAGCATCCAGGAAGCCGGCCTAAAACTGCTTGTACCAATTGTTATTGTAAAAAGTGTTGCTTTC
ATTGCCAAGTGTGTTTCACAACCAAAGCCTTAGGCATCTCCTATGGCAGGAAGAAGCGGAGACAGCGACGAAG
AGCTCCTCCAAGCAGTCAGAATCATCAAGTTTCTCTACCAAAGCAGTGAGTAATATATGTAATGCAATCCTTA
GAAATATATGCAATAGTAGCATTAGTAGTAGTAGCAATAATAGCAATAGTTGTGTGGACCATAGTGCTCCTAG
AATATAGGAAAATATTAAGACAAAGGAAAATAGACAGATTAATTGATAGAATAAGAGAAAGAGCAGAAGACAG
TGGCAATGAAAGTGAAGGGGATGAGGAGGAATTATCAGCCCTTGTGGAAATGGGGCATCATGCTCCTTGGGAT
GTTGATGATCTGTAGTGCTACAGAAAAATTGTGGGTCACAGTCTATTATGGGGTACCTGTGTGGAAAGAAGCA
ACCACCACTTTATTTTGTGCATCAGATGCTAAAGCATATCATACAGAGATGCATAATGTTTGGGCCACACATG
CCTGTGTACCCACAGACCCTAGCCCACAAGAAGTAGTATTGGGAAATGTGACAGAAAATTTTAACATGTGGAA
AAATAACATGGTAGAACAGATGCATGAAGATATAATCAGTTTGTGGGATCAAAGCCTAAAGCCATGTGTAAAA
TTAACCCCACTCTGTGTCACTTTAAATTGCACTGATGTGAGAAATAATACTAATACCACTATTAATAATTGGG
AAAAGGTGGACAAAGGGGAAATAAAAAACTGCTCTTTCAATGTCACCACAAGCATAAGAGATAAGATGCAACA
AGCATATTCACTTTTTTATAAACTTGATGTAGTGCAAATAGAGAATACAAGTAGCTATACATTGATAAATTGT
AACACCTCAGTCATTACACAGGCCTGTCCAAAGGTATCCTTTGAACCAATTCCCATACATTATTGTACCCCGG
CTGGTTTTGCAATTCTAAAGTGTAATGATAATAAGTTCAATGGAACAGGACCATGTACTAATGTTAGCACAGT
ACAATGTACACATGGAATTAAGCCAGTAGTGTCAACTCAACTGCTGTTGAATGGCAGTCTGGCAGAAGGAGGA
GAGGTAGTAATTAGATCTGAAAATTTCACAAACAATGCTAAAACCATAATAGTACAGCTGAATACATCTGTAG
AAATTAATTGTATAAGACCCAACAACAATACAAGAAAAAGTATAACTATAGGACCAGGGAGAGCATTTTATAC
AACAGACATAATAGGAGATATAAGACAAGCACATTGTAACCTTAGTAGAGCAAAATGGAATGACACTTTAAAA
CAGATAGTTACAAAATTAAAAGAACAATTTGAGAACAAGACAATAGTCTTTAATCAATCTTCAGGAGGGGACC
CAGAAATTGTAATGCACAGCTTTAATTGTGGAGGGGAATTTTTCTACTGTAATACAACACAGCTGTTTAACAG
TACTTGGAATGGTACTGACTGGAATGACACTACAGGGTTAGAGAACATCACACTCCCATGCAGAATAAAACAA
ATTGTAAACAGGTGGCAGGAAGTAGGAAAAGCAATGTATGCCCCTCCCATCAAAGGACAAATTAGATGTTCAT
CAAATATTACAGGGCTACTATTAACAAGAGATGGGGGAAACAGTAGTGAGATGACCGAGATCTTCAGACCTGG
AGGAGGAGATATGAGGGACAATTGGAGAAGTGAATTATATAAATATAAAGTAGTAAAAATTGAGCCATTAGGA
GTAGCACCCACCAAGGCAAAGAGAAGAGTGGTGCAGAGAGAAAAAAGAGCAGTGGGAACAATAGGAGCTATGT
TCCTTGGGTTCTTGGGAGCAGCAGGAAGCACTATGGGCGCAGCGTCAATGACGCTGACGGTACAGGCCAGACT
ATTATTGTCTGGTATAGTGCAACAGCAGAACAATTTGCTGAGAGCTATTGAGGCGCAACAGCATCTGTTGCAA
CTCACAGTCTGGGGCATCAAGCAGCTCCAGGCTAGAGTCCTGGCTGTGGAAAGATACCTAAGGGATCAACAGC
TCCTGGGGATTTGGGGTTGCTCTGGAAAACTCATTTGCACCACTAATGTGCCTTGGAATGCTAGTTGGAGTAA
TAAATCTCTGGAAAAGATTTGGAATAACATGACCTGGATGGAGTGGGACAGAGAAATTGACAATTACACAAAA
TTGATATACACCTTAATTGAAGAATCGCAAAACCAGCAGGAAAAGAATGAACACGACTTATTGGAGTTGGATA
AGTGGGACAGTTTGTGGAATTGGTTTGACATAACAAAATGGCTGTGGTATATAAAAATATTCATAATGATAGT
AGCAGGTTTAGTAGGTTTAAGAATAGTTTTTACTGTGCTTTCTATAGTGAATAGAGTTAGGCAGGGATATTCA
CCACTATCATTTCAGACCCACCACCCAGTCCCGCGGGGACCCGACAGGCCCGAAGGAATCGAAGAAGGAGGTG
GAGAGAGAGACAGAGACACATCCGGACCATTAGTGAACGGATTCTTAGCGATTATCTGGGTCGACCTGCGGAG
CCTGTGCCTCTTCAGCTACCACCGCTTGAGAGACTTACTCTTGATTGTAGCGAGGATTGTGGAACTTCTGGGA

CGCAGGGGGTGGGAGGCCCTGAAATATTGGTGGAATCTCCTGCAGTATTGGAGTCAGGAACTAAAGAATAGTG
CTGTTAGTCTGCTTAATGCCACAGCTATAGCAGTAGCTGAGGGGACAGATAGGATTATAGAAGTATTACAAAG
AGCTTGTAGAGCTATTCTCCACATACCTAGAAGAATAAGACAGGGCTTAGAAAGGGCTTTGCTATAAGATGGG
TGGTAAGTGGTCAAAACGTCGTGCGGGTGGATGGGAAGCTGTAAGGGAAAAAATAAGACAAACTGAGCCTGAG
CCAGCAGCAGAGGGGGTGGGAGCAGCATCTCGAGACCTGGAAAAATATGGAGCAATCACCAGTAGCAATACAG
CACATACCAACGCTGATTGTGCCTGGGTAGAAGCACAAGAGGAGGATGAAGAGGTGGGTTTCCCAGTCAGACC
TCAGGTACCTTTAAGACCTATGACTTTCAAGGGAGCGCTAGATCTTAGCCACTTTTTAAAAGAAAAGGGGGGA
CTGGAAGGGTTAATTCACTCCCAGAAAAGACAAGACATCCTTGATTTGTGGGTCTACAACACACAAGGCTATT
TCCCTGATTGGCAGAACTACACACCAGGGCCAGGGGAGAGATTTCCCCTGACCTTTGGATGGTGCTTCAAGCT
AGTACCAGTTGATCCAGATCAGGTAGAAGAGGCTAATAAAGGAGAGACCAACAGCTTGTTACACCCTATGAGC
CAGCATGGGATAGAGGACCCGGAGAAAGAAGTATTAATGTGGAAGTTTGACAGCCGCCTAGCATTTCATCACG
TGGCCAGAGAGAAGCATCCGGAGTACTTCAAGAACTGATGACATCGGGTTTTTCTACAAGGGACTTTCCGCTG
GGGACTTTCCAGGGGAGGTGTGGCCTGGGCGGGACAGGGGAGTGGCGAGCCCTCAGATGCTGCATATAAGCAG
CTGCTTTCTGCCTGTACTGGGTCTCTCTGGTTAGACCAGATTTGAGCCTGGGAGCTCTCTGGCTAACTAGGGA
ACCCACTGCTTAAGCCTCAATAAAGCTTGCCT

>AE-B4 HIV-1 genome, derived from RNA genomic sequence
ACTCGGCTTGCTGAAGCGCGCACGGCAAGAGGCGAGGGGGGCGACTGGTGAGTACGCCAAAAAATTTTTTGAC
TAGCGGAGGCTAGAAGGAGAGAGATGGGTGCGAGAGCGTCAATATTAAGCGGGGGAGAATTAGATAGATGGGA
AAAAATTCGGTTAAGGCCAGGGGGAAAGAAACAATATAGACTAAAACATATAGTATGGGCAAGCAGGGAACTA
GAACGATTCGCAGTTAATCCTGGCCTTCTAGAGACATCAGAGGGCTGTAGACAAATACTAGAACAGCTACAAC
CGTCCATTAAGACAGGATCAGAAGAACTTAAATCCTTATATAATACAGTAGCAACCCTCTATTGTGTGCATCA
AAAGATAGAGGTAAGAGACACCAAGGAAGCTTTAGATAAGATAGAGGAAGAGCAAAACAAAAGTAAGAAAAAG
GCACAGCAAGCAGCAGCTGACACAGGAAACAGCAGCAAGGTCAGTCAAAATTACCCTATAGTACAGAACCTTC
AGGGACAAATGGTACATCAGCCCATATCACCTAGAACTTTAAATGCATGGGTAAAAGTAGTAGAAGAGAAAGC
TTTCAGCCCAGAAGTAATACCCATGTTTTCAGCATTATCAGAAGGAGCCACCCCACAAGATATAAACACCATG
CTAAATACAGTGGGGGGACATCAAGCAGCCATGCAAATGTTAAAGGAGACCATCAATGAAGAGGCTGCAGAAT
GGGATAGATTGCACCCAGTGCATGCAGGGCCTGTAGCACCAGGCCAGCTGAGAGAACCAAGGGGAAGTGACAT
AGCAGGAACTACTAGTAACCTTCAGGAACAAATAGGATGGATGACAAATAATCCACCTATCCCAGTAGGAGAA
ATCTATAAAAAATGGATAATCATGGGATTAAATAAAATAGTAAGAATGTATAGCCCTACCAGCATCCTGGACA
TAAAACAAGGACCAAAGGAACCCTTTAGAGACTATGTAGACCGGTTTTATAAAACTCTAAGAGCCGAGCAAGC
TTCACAGGATGTAAAAAATTGGATGACAGAAACCTTGTTGGTCCAAAATGCGAACCCAGATTGTAAGACTATT
TTAAAAGCATTGGGACCAGCAGCTACACTAGAAGAAATGATGACTGCATGTCAGGGAGTGGGGGGACCCGGCC
ATAAAGCAAGAGTTTTGGCTGAAGCAATGAGCCAAGTAACAAATCCAGCTACCATAATGATACAGGGAGGCAA
ATTTAGGAACCAAAGAAAAACTATTAAGTGCTTCAACTGTGGCAAAGAGGGGCACTTGGCCAGAAATTGCAGG
GCCCCTAGAAAAAAGGGCTGTTGGAAATGTGGAAGGGAAGGACACCAAATGAAAGATTGCACTGAGAGGCAGG
CTAATTTTTTAGGGAAAATCTGGCCTTCCCACAAAGGAAGGCCAGGGAATTTTCTTCAGAACAGACCAGAGCC
AACAGCCCCACCAGAAGAGAGCCTCAGGGAAGAGACAGCAGCCCCCTCTCAGAAGCAGGGACCGATAGACAAG
GAACTGTATCCTTTAGCTTCCCTCAGATCACTCTTTGGCAACGACCCCTCGTCACAATAAAGATAGGGGGGCA
ACTAAAGGAAGCTCTATTAGATACAGGAGCAGATGATACAGTAGTAGAAGAAATGAATTTGTCAGGAAGATGG
AAACCAAAAATGATAGGAGGAATTGGAGGTTTTATCAAAGTAAGACAGTATGATCAAATACCCATAGAAATCT
GCGGACATAAAGCTGTAGGTACAGTATTAGTAGGACCGACACCTGTCAACATAATTGGAAGAAATCTGTTGAC
TCAGCTTGGCTGCACTTTAAATTTTCCTATTAGTCCTATTGAAACTGTACCAGTAAAATTAAAGCCAGGAATG
GATGGGCCAAAGGTTAAACAATGGCCATTGACAGAAGAAAAAATAAAAGCATTAGTAGAAATTTGTACAGAAA
TGGAAAAGGAAGGAAAAATTTCAAAAATTGGGCCTGAAAATCCATACAATACTCCAGTATTTGCCATAAAGAA
AAAAGACAGTACTAAATGGAGAAAATTAGTAGACTTTAGAGAACTTAATAAGAGAACTCAAGACTTCTGGGAA
GTTCAATTAGGAATACCACATCCTGCAGGGTTAAAAAAGAAAAAATCAGTAACAGTACTGGATGTGGGTGATG
CATATTTTTCAGTTCCATTAGATAAGGAATTCAGGAAGTATACTGCATTTACCATACCTAGTATAAACAATGA
GACACCAGGGATTAGATATCAATACAATGTGCTTCCACAGGGATGGAAAGGATCCCCAGCCATATTCCAAAGT
AGTATGACAAAAATCTTAGAGCCTTTTAGAAAACAAAATCCAGAAATAGTTATCTATCAATACATGGATGATT
TATATGTAGGATCTGACTTAGAAATAAGGCAGCATAGAGCAAAAATAGAGGAACTGAGACAACATCTGTTGAG
GTGGGGATTTACCACACCAGACAAAAAGCATCAGAAAGAACCTCCATTCCTTTGGATGGGTTATGAGCTCCAT
CCTGATAAATGGACAGTACAGCCTATAATGCTGCCAGAAAAAGACAGCTGGACTGTCAATGACATACAGAAGT
TAGTGGGAAAATTGAATTGGGCAAGTCAGATCTATCCAGGGATTAAAGTAAGGCAATTATGTAGACTCATTAG
GGGAACCAAAGCACTAACAGAAGTAGTACCACTAACAGCAGAAGCAGAGCTAGAATTGGCAGAAAACAGGGAG
ATACTAAAAGAACCAGTACATGGAGTGTATTATGACCCATCAAAAGACTTAATAGCAGAAATACAGAAGCAGG
GGCAAGGCCAGTGGACATATCAAATTTATCAAGAGCAATTTAAAAATTTGAAAACAGGAAAGTATGCAAGAAT
GAAGGGTGCCCACACTAATGATGTAAAACAGTTAACAGAGGTAGTGCAAAAAGTAGCCACAGAAAGCATAGTA
ATATGGGGAAAAACTCCTAAATTTAGATTACCCATACAAAAAGAAACATGGGATGCATGGTGGACAGAGTATT
GGCAAGCCACCTGGATTCCTGAGTGGGAATTTGTCAATACCCCTCCCTTAGTAAAATTATGGTACCAGTTAGA
AAAAGAACCCATAATAGGAGCAGAAACTTTCTATGTAGATGGGGCAGCTAATAGGGAGACTAAATTAGGAAAA
GCAGGATATGTTACTGACAGAGGAAGACAAAAGGTTGTCTCCCTAACTGACACAACAAATCAGAAGACTGAGT
TACAAGCAATCCATCTAGCTTTGCAGGATTCGGGACTAGAAGTAAACATAGTAACAGACTCACAATATGCATT
AGGAATCATTCAAGCACAACCAGATAAGAGTGAATCAGAGTTAGTCAGTCAAATAATAGAGCAGTTAATAAAA
AAGGAAAAGGTCTACTTAGCATGGGTACCGGCACACAAGGGAATTGGAGGAAATGAACAAGTAGATAAATTAG
TCAGTGCTGGAATCAGGAAAGTACTATTTTTGGATGGAATAGATAAGGCCCAAGATGAACATGAGAAATATCA
CAGTAATTGGAGAGCAATGGCTAGTGATTTTAACTTGCCACCTGTAGTAGCAAAAGAAATAGTAGCCAGCTGT
GATAAATGTCAGCTAAAAGGAGAAGCCATGCATGGACAAGTAGACTGTAGTCCAGGAATATGGCAATTAGATT
GCACACATTTAGAAGGAAAAATTATCCTGGTAGCAGTCCATGTAGCCAGTGGATATATAGAAGCAGAAGTTAT
CCCAGCAGAGACAGGGCAGGAAACAGCATACTTTATCTTAAAATTAGCAGGAAGATGGCCAGTAAAAACAATA

CATACAGACAATGGCAGCAATTTTATCAGTAATACAGTTAAGGCTGCCTGTTGGTGGGCGGGGATCAAGCAAG
AATTTGGCATTCCCTACAATCCCCAAAGTCAAGGAGTAGTAGAATCTATGAATAAAGAATTAAAGAAAATTAT
AGGACAGGTAAGAGATCAGGCTGAACATCTTAAAACAGCAGTACAAATGGCAGTATTCATCCACAATTTTAAA
AGAAAAGGGGGGATTGGGGGATACAGTGCAGGGGAAAGAATAGTAGACATAATAGCAACAGACATACAAACTA
AAGAACTACAAAAACAAATTACAAAAATTCAAAATTTTCGGGTTTATTACAGGGACAACAGAGATCCACTTTG
GAAAGGACCAGCAAAGCTTCTTTGGAAAGGTGAAGGGGCAGTAGTAATACAAGAGAATAGTGAAATAAAAGTA
GTGCCAAGAAGAAAAGTAAAGATCATTAGGGATTATGGAAAACAGATGGCAGGTGATGATTGTGTGGCAAGTA
GACAGGATGAAGATTAGAACATGGAATAGTTTAGTAAAACACCATATGTATGTTTCAAGGAAAGCTAAGGGAT
GGGTTTATAGGCATCACTATGAAAGCACTAATCCAAGAGTAAGCTCAGAAGTACACATCCCACTAGGGGATGC
TAGATTGGTGATAACAACATATTGGGGTCTGCATGCAGGAGAAAGAGAGTGGCATTTGGGTCATGGAGCCTCC
ATAGAATGGAGGAAAAAGAAATATAGCACACAAGTAGACCCTGACCTAGCAGACCAACTAATTCATCTGCATT
ACTTTGATTGTTTTTCAGAATCTGCTATAAGAAATGCCATATTAGGACGTATAGTTAGTCCTAGGTGTGAATA
TCAAGCAGGACATAACAAGGTAGGATCTCTACAGTACTTGGCACTAGCAGCATTAATAAAACCAAAAAAGATA
AAGCCACCTTTGCCTAGTATTAGGAAACTGACAGAGGATAGATGGAACAAGCCCCAGAAGACCATGGGCCACA
GAGGGAGCCATACAATGAATGGACACTAGAGCTTTTAGAGGAACTTAAGAATGAAGCTGTTAGACATTTTCCT
AGGCCATGGCTCCATAGCTTAGGACAATATATCTATGAGACTTATGGGGATACTTGGACAGGAGTGGAGGCCA
TAATAAGAATTCTGCAACAACTGCTGTTTATTCATTTCAGAATTGGATGTCGACATAGCAGGATAGGTATTCT
ACCGAGGAGAGCAAGGAATGGAGCCAGTAGATCCTAGTTTAGAGCCCTGGAAGCATCCAGGAAGCCGGCCTAA
AACTGCTTGTACCAATTGCTATTGTAAAAAGTGTTGCTTTCATTGCCAAGTGTGTTTCACGACCAAAGCCTTA
GGCATCTCCTATGGCAGGAAGAAGCGGAGACAGCGACGAAGAGCTCCTCAAGGCAGTCAGACTCATCAAGTTT
CTCTACCAAAGCAGTGAGTAATATATGTAATGCAACCCTTAGAAATATATGCAGTAGTAGCATTAGTAGTAGT
AGCAATAATAGCAATAGTTGTGTGGACCATAGTGCTCCTAGAATACAGGAAAATATTAAGACAAAAGAAAATA
GACAGGTTAATTGATAGAATAAGAGAAAGAGCAGAAGACAGTGGCAATGAAAGTGAAGGGGATGAGGAGGAAT
TATCAGCCCTTGTGGAAATGGGGCATCATGCTCCTAGGGATGTTGATGATCTGTAGTGCTACAGAAAAATTGT
GGGTCACAGTCTATTATGGGGTACCTGTGTGGAAAGAAGCAACCACCACTTTATTTTGTGCATCAGATGCTAA
AGCATTTGATACAGAGGCACATAATGTTTGGGCCACACATGCCTGTGTACCCACAGACCCTAGCCCACAAGAA
GTAGTATTGGAAAATGTGACAGAAAATTTTAACATGTGGAAAAATAACATGGTAGAACAGATGCATGAAGATA
TAATCAGTTTGTGGGATCAAAGCCTAAAGCCATGTGTAAAATTAACCCCACTCTGTGTCACTTTAAATTGCAC
TGATTTGGGAAATACTACTAATACCACTACTAGTAATTGGGAAAAGGTAGACAAAGGGGAAATAAAAAACTGC
TCTTTCAATGTCACCACAAGCCTAAGAGATAAGATGCAAAAAGCATATTCACTTTTTTATAAACTTGATGTAG
TACAAATAGAGAATACAAGTAGCTATACATTGATAAATTGTAACACCTCAGTCATTACACAGGCCTGTCCAAA
GGTATCCTTTGAACCAATTCCCATACATTATTGTACCCCGGCTGGTTTTGCGATTCTAAAGTGTAATGATAAT
AAGTTCAATGGAACAGGACCATGTACTAATGTTAGCACAGTACAATGTACACATGGAATTAAGCCAGTAGTGT
CAACTCAACTGCTGTTGAATGGCAGTCTGGCAGAAGGAGGAGAGGTAGTAATTAGATCTGAAAATTTCACAAA
CAATGCTAAAACCATAATAGTACAGCTGAATACATCTGTAGAAATTAATTGTACAAGACCCAACAACAATACA
AGAAAAAGTATAACTATAGGACCAGGGAGAGCATTTTATACAACAGGCATAATAGGAGATATAAGACAAGCAC
ATTGTAACCTTAGTAGCGCAAAATGGAATGACACTTTAAAACAGATAGTTATAAAGTTAAAAGAACAATTTGG
GAACAAGACAATAGTCTTTAATCAATCTTCAGGAGGGGACCCAGAAATTGTAATGCACAGCTTTAATTGTGGA
GGGGAATTTTTCTACTGTAATACAACACAGCTGTTTAACAGTACTTGGAATGGTACTGCCTGGAATGATACTA
CAGGGTCAGATAACAATGGAAATATCACACTCCCATGCAGAATAAAACAAATTGTAAACAGGTGGCAGGAAGT
AGGAAAAGCAATGTATGCCCCTCCCATCGAAGGACAAATTAGATGTTCATCAAATATTACAGGGCTACTATTA
ACAAGAGATGGTGGAAATAGTAATAACACGACTGAGATCTTCAGACCTGGAGGAGGAGATATGAGGGACAATT
GGAGAAGTGAATTATATAAATATAAAGTAGTAAAAATTGAGCCATTAGGAGTAGCACCCACCAAGGCAAAGAG
AAGAGTGGTGCAGAGAGAAAAAAGAGCAGTGGGAACAATAGGAGCTATGTTCCTTGGGTTCTTGGGAGCAGCA
GGAAGCACTATGGGCGCAGCATCAATGACGCTGACGGTACAGGCCAGACTATTATTGTCTGGTATAGTGCAAC
AGCAGAACAATTTGCTGAGAGCTATTGAGGCGCAACAGCATCTGTTGCAACTCACAGTCTGGGGCATCAAGCA
GCTCCAGGCTAGAGTCCTGGCTGTGGAAAGATACCTAAAGGATCAACAGCTCCTGGGGATTTGGGGTTGCTCT
GGAAAACTCATTTGCACCACTAATGTGCCTTGGAATGCTAGTTGGAGTAATAAATCTCTGGAAAAGATTTGGA
ATAACATGACCTGGATGGAGTGGGACAGAGAAATTGACAATTACACAAAATTGATATACACCTTAATTGAAGA
CTCGCAAAACCAGCAGGAAAAGAATGAACAAGACTTATTGGAGTTGGATAAGTGGGACAGTTTGTGGAATTGG
TTTGACATAACAAAATGGCTGTGGTATATAAAAATATTCATAATAATAGTAGCAGGTTTAGTAGGTTTAAGAA
TAGTTTTTGCTGTGCTTTCTATAGTGAATAGAGTTAGGCAGGGATATTCACCACTATCATTTCAGACCCACCT
CCCAGTCCCGCGGGGACCCGACAGGCCCGAAGGAATCGAAGAAGGAGGTGGAGAGAGAGACAGAGACACATCC
GGACCATTAGTGAACGGATTCTTAGCGATTATCTGGGTCGACCTGCGGAGCCTGTGCCTCTTCAGCTACCACC

GCTTGAGAGACTTACTCTTGATTGTAGCGAGGATTGTGGAACTTCTGGGACGCAGGGGGTGGGAGGCCCTGAA
ATATTGGTGGAATCTCCTGCAGTATTGGAGTCAGGAACTAAAGAATAGTGCTGTTAGTCTGCTTAATGCCACA
GCTATAGCAGTAGCTGAGGGGACAGATAGGATTATAGAAATATTACAAAGAGCTTGTAGAGCTATTCTCCACA
TACCTAGAAGAATAAGACAGGGCTTAGAAAGGGCTTTGCTATAAGATGGGTGGTAAGTGGTCAAAACGTAGTG
GGGGATGGGAAGCTGTAAGGGAAAAAATAAGACAAACTGAGCCAGCAGCAGAAGGGGTGGGAGCAGTATCTCG
AGACCTGGAAAAACATGGAGCAATCACCAGTAGCAATACAGCAAATACCAATGCTGATTGTGCCTGGCTAGAA
GCACAAGAGGAGGATGAAGAGGTGGGTTTTCCAGTCAGACCTCAGGTACCTTTAAGACCAATGACTTACAAGG
CAGCGCTAGATCTTAGCCACTTTTTAAAAGAAAAGGGGGGACTGGAAGGGCTAATTCATTCCCAGAAAAGACA
AGATATCCTTGATTTGTGGGTCTACAACACACAAGGCTATTTCCCTGATTGGCAGAACTACACACCAGGGCCA
GGGGAGAGATTTCCACTGACCTTTGGATGGTGCTTCAAGCTAGTACCAGTTGATCCAGATCAGGTAGAAGAGG
CTAATAAAGGAGAGAACAACAGCTTGTTACACCCTATGAGCCAGCATGGGATAGAGGACCCGGAGAAAGAAGT
ATTAATGTGGAAGTTTGACAGCCGCCTAGCATTTCATCACATGGCCAGAGAGAAGCATCCGGAGTACTTCAAG
AACTGATGACATCGGGTTTTCTACAAGGGACTTTCCGCTGGGGACTTTCCAGGGGAGGTGTGGTCTGGGCGGG
ACAGGGGAGTGGCGAGCCCTCAGATGCTGCATATAAGCAGCTGCTTTCTGCCTGTACTGGGTCTCTCTGGTTA
GACCAGATTTGAGCCTGGGAGCTCTCTGGCTAACTAGGGAACCCACTGCTTAAGCCTCAATAAAGCTTGCCT

```
>AE-B7 HIV-1 genome, derived from RNA genomic sequence
ACTCGGCTTGCTGAGCGCGCACAGCAAGAGGCGAGGGGCGGCGACTGGTGAGTACGCCAAAACTTTTTTGACT
AGCGGAGGCTAGAAGGAGAGAGATGGGTGCGAGAGCGTCAATATTAAGCGGGGGAGAATTAGATAGATGGGAA
AAAATTCGGTTAAGGCCAGGGGGAAAGAAACAATATAGACTAAAACATTTAGTATGGGCAAGCAGGGAACTAG
AACGATTCGCAGTTAATCCTGGCCTTTTAGAGACATCAGATGGCTGTAGACAAATACTAGAACAGCTACAACC
AGCCATTAAGACAGGATCAGAAGAACTTAAATCCTTATATAATACAGTAGCAACCCTCTATTGTGTGCATCAA
AAGATAGAGGTAAGAGACACCAAGGAAGCTTTAGATAAGATAGAGGAAGAGCAAAACAAAAGTAAGAAAAAGG
CACAGCAAGCAGCAGCTGACACAGGAAACAGCAGCAAGGTCAGTCAAAATTACCCTATAGTACAGAACCTTCA
GGGACAAATGGTACATCAACCCATATCACCTAGAACTTTAAATGCATGGGTAAAAGTAGTAGAAGAGAAGGCT
TTCAGCCCAGAAGTAATACCCATGTTTTCAGCATTATCAGAAGGAGCCACCCCACAAGATATAAACACCATGC
TAAATACAGTGGGGGGACATCAAGCAGCCATGCAAATGTTAAAGGAGACCATCAATGAAGAGGCTGCAGAATG
GGATAGATTGCACCCAGTGCATGCAGGGCCTGTAGCACCAGGCCAGCTGAGAGAACCAAGGGGAAGTGACATA
GCAGGAACTACTAGTAACCTGCAGGAACAAATAGGATGGATGACAAGTAATCCACCTATCCCAGTAGGAGAAA
TCTATAAAAAATGGATAATCATGGGATTAAATAAAATAGTAAGAATGTATAGCCCTACCAGCATTCTGGACAT
AAAACAAGGACCAAAGGAACCCTTTAGAGACTATGTAGACCGGTTCTATAAAACTCTAAGAGCCGAGCAAGCT
TCACAGGATGTAAAAAATTGGATGACAGAAACCTTGTTGGTCCAAAATGCAAACCCAGATTGTAAGACTATTT
TAAAAGCATTGGGACCAGCAGCTACACTAGAAGAAATGATGACAGCATGTCAGGGAGTGGGGGGACCCGGCCA
TAAAGCAAGAGTTTTGGCTGAAGCAATGAGCCAAGTAACAAATCCAGCTACCATAATGATACAGGGAGGCAAA
TTTAGGAACCAAAGAAAAACTATTAAGTGCTTCAACTGTGGCAAAGAGGGGCACTTGGCCAGAAATTGCAGGG
CCCCTAGAAAAAAGGGCTGTTGGAAATGTGGAAGGGAAGGACACCAAATGAAAGATTGTACTGAGAGACAGGC
TAATTTTTTAGGGAAAATCTGGCCTTCCCACAAAGGAAGGCCAGGGAATTTTCTTCAGAACAGACCAGAGCCA
ACAGCCCCACCAGAGGAGAGCCTCAGGGAAGAGACAGCAGCCCCCTCTCAGAGGCAGGGACCGATAGACAAGG
AACTGTATCCTTTAGCTTCCCTCAAATCACTCTTTGGCAACGACCCCTCGTCACAATAAAGATAGGGGGGCAA
CTAAAGGAAGCTCTATTAGATACAGGAGCAGATGATACAGTATTAGAAGAAATAAATTTGTCAGGAAGATGGA
AACCAAAAATGATAGGAGGAATTGGAGGTTTTATCAAAGTAAGACAGTATGATCAAATACCCATAGAAATCTG
CGGACACAAAGCTGTAGGTACAGTATTAGTAGGACCGACACCTGTCAACATAATTGGAAGAAATCTGTTGACT
CAGATTGGCTGCACTTTAAATTTTCCTATTAGTCCTATTGAAACTGTACCAGTAAAATTAAAGCCAGGAATGG
ATGGGCCAAAGGTTAAACAATGGCCATTGACAGAAGAAAAAATAAAAGCATTAGTAGAAATTTGTACAGAAAT
GGAAAAGGAAGGAAAAATTTCAAAAATTGGGCCTGAAAATCCATACAATACTCCAGTATTTGCCATAAAGAAA
AAAGACAGTACTAAATGGAGAAAATTAGTAGACTTTAGAGAACTTAATAAGAGAACTCAAGACTTCTGGGAAG
TTCAATTAGGAATACCACATCCTGCAGGGTTAAAAAAGAAAAAATCAGTAACAGTACTGGATGTGGGTGATGC
ATACTTTTCAGTTCCATTAGATAAAGAATTCAGGAAGTATACTGCATTTACCATACCTAGTATAAACAATGAG
ACACCAGGGATTAGATATCAGTACAATGTGCTTCCACAGGGATGGAAAGGATCCCCAGCCATATTCCAAAGTA
GTATGACAAAAATCTTAGAGCCTTTTAGAAAACAAAACCCAGAAATGGTTATCTATCAATACATGGATGATTT
ATATGTAGGATCTGACTTAGAAATAGGGCAGCATAGAGCAAAAATAGAGGAACTGAGACAGCATCTGTTGAGG
TGGGGATTTACCACACCAGACAAAAAGCATCAGAAAGAACCTCCATTCCTTTGGATGGGTTATGAGCTCCATC
CTGATAAATGGACAGTACAGCCTATAAAGCTGCCAGAAAAAGACAGCTGGACTGTCAATGACATACAGAAGTT
AGTGGGAAAATTGAATTGGGCAAGTCAGATCTATCCAGGGATTAAAAGTAAGGCAATTATGTA??AACTCATT
AGGGGAACCAAAGCACTAACAGAAGTAGTACCACTAACAGCAGAAGCAGAGCTAGAATTGGCAGAAAACAGGG
AGATACTAAAAGAACCAGTACATGGAGTGTATTATGACCCATCAAAAGACTTAATAGCAGAAATACAGAAGCA
GGGGCAAGGCCAGTGGACATATCAAATTTATCAAGAGCCATTTAAAAATTTGAAAACAGGAAAGTATGCAAGA
ATGAAGGGTGCCCACACTAATGATGTAAAACAGTTAACAGAGGCAGTGCAAAAAATAGCCACAGAAAGCATAG
TAATATGGGGAAAAACTCCTAAATTTAGATTACCCATACAAAAAGAAACATGGGAAGCATGGTGGACAGAGTA
TTGGCAAGCCACCTGGATTCCTGAGTGGGAATTTGTCAATACCCCTCCCTTAGTAAAATTATGGTACCAGCTA
GAGAAAGAACCCATAATAGGAGCAGAAACTTTCTATGTAGATGGGGCAGCTAATAGGGAAACTAAATTAGGAA
AAGCAGGATATGTTACTGACAGAGGAAGACAAAAGGTTGTCTCCCTAACTGACACAACAAATCAGAAGACTGA
GTTACAAGCGATCCATCTAGCTTTGCAGGATTCGGGGCTAGAAGTAAACATAGTAACAGACTCACAATATGCA
TTAGGAATCATTCAAGCACAACCAGATAAGAGTGAATCAGAGTTAGTCAGTCAGATAATAGAGCAGTTAATAA
AAAAGGAAAAGGTCTATTTAGCATGGGTACCAGCACACAAGGGAATTGGAGGAAATGAACAAGTAGATAAATT
AGTCAGTGCTGGAATCAGGAAAGTACTATTTTTGGATGGAATAGATAAGGCCCAAGAAGAACATGAGAAATAT
CACAATAATTGGAGAGCAATGGCTAGTGATTTTAACCTGCCACCTGTAGTAGCAAAAGAAATAGTAGCCAGCT
GTGATAAATGTCAGTTAAAAGGAGAAGCCATGCATGGACAAGTAGACTGTAGTCCAGGAATATGGCAATTAGA
TTGCACACATTTAGAAGGAAAAATTATCCTGGTAGCAGTCCATGTAGCCAGTGGATATATAGAAGCAGAAGTT
ATCCCAGCAGAGACAGGGCAGGAAACAGCATACTTTATCTTAAAATTAGCAGGAAGATGGCCAGTAAAAACAA
```

TACATACAGACAATGGCAGCAATTTTATCAGTAATACAGTTAAGGCTGCCTGTTGGTGGGCGGGGATCAAGCA
AGAATTTGGCATTCCCTACAATCCCCAAAGTCAAGGAGTAGTAGAATCTATGAATAAAGAATTAAAGAAGATT
ATAGGACAGGTAAGAGATCAGGCTGAACATCTTAAAACAGCAGTACAAATGGCAGTATTCATCCACAATTTTA
AAAGAAAAGGGGGGATTGGGGGATACAGTGCAGGGGAAAGAATAGTAGACATAATAGCAACAGACATACAAAC
TAAAGAACTACAAAAACAAATTACAAAAATTCAAAATTTTCGGGTTTATTACAGGGACAACAGAGATCCACTT
TGGAAAGGACCAGCAAAGCTTCTTTGGAAAGGTGAAGGGGCAGTAGTAATACAAGAGGATAGTGAAATAAAAG
TAGTGCCAAGAAGAAAAGCAAAGATCATTAGGGATTATGGAAAACAGATGGCAGGTGATGATTGTGTGGCAAG
TAGACAGGATGAAGATTAGAACATGGAATAGTTTAGTAAAACACCATATGTATATTTCAAGGAAAGCTCAGGG
ATGGGTTTATAGGCATCACTATGAAAGCACTAATCCAAGAGTAAGCTCAGAAGTACACATCCCACTAGGGGAT
GATAAATTGGTGATAACAACATATTGGGGTCTGCATGCAGGAGAAAGAGAGTGGCATTTGGGTCATGGAGCCT
CCATAGAATGGAGGAAAAGAAGATATAGCACACAAGTAGACCCTGACCTAGCAGACCAACTAATTCATCTGCA
TTACTTTGATTGTTTTTCAGAATCTGCTATAAGACATGCCATATTAGGACGTATAGTTAGTCCTAGGTGTGAA
TATCAAGCAGGACATAACAAGGTAGGATCTCTACAGTACTTGGCACTAGCAGCATTAATAAAACCAAAAAAGA
GAAAGCCACCTTTGCCTAGTGTTAGGAAACTGACAGAGGATAGATGGAACAAGCCCCAGAAGACCATGGGCCA
CAGAGGGAGCCATACAATGAATGGACACTAGAACTTTTAGAGGAACTTAAGAATGAAGCTGTTAGACATTTTC
CTAGGCCATGGCTCCATAGCTTAGGACAATATATCTATGAAACTTATGGGGATACTTGGACAGGAGTGGAAGC
CATAATAAGAATTCTGCAACAACTGCTGTTTATTCATTTCAGAATTGGATGTCAACATAGCAGAATAGGTATT
CTACCAAGGAGAGCAAGGAATGGAGCCAGTAGATCCTAGTTTAGAGCCCTGGAAGCATCCAGGAAGCCGGCCT
AAAACTGCTTGTACCAATTGTTATTGTAAAAAGTGTTGCTTTCATTGCCAAGTGTGTTTCACAACCAAAGCCT
TAGGCATCTCCTATGGCAGGAAGAAGCGGAGACAGCGACGAAGAGCTCCTCCAAGCAGTCAGAATCATCAAGT
TTCTCTACCAAAGCAGTGAGTAATATATGTAATGCAATCCTTAGAAATATATGCAATAGTAGCATTAGTAGTA
GTAGCAATAATAGCAATAGTTGTGTGGACCATAGTGCTCCTAGAATATAGGAAAATATTAAGACAAAGGAAAA
TAGACAGATTAATTGATAGAATAAGAGAAAGAGCAGAAGACAGTGGCAATGAAAGTGAAGGGGATGAGGAGGA
ATTATCAGCCCTTGTGGAAATGGGGCATCATGCTCCTTGGGATGTTGATGATCTGTAGTGCTACAGAAAAATT
GTGGGTCACAGTCTATTATGGGGTACCTGTGTGGAAAGAAGCAACCACCACTTTATTTTGTGCATCAGATGCT
AAAGCATATCATACAGAGATGCATAATGTTTGGGCCACACATGCCTGTGTACCCACAGACCCTAGCCCACAAG
AAGTAGTATTGGGAAATGTGACAGAAAATTTTAACATGTGGAAAAATAACATGGTAGAACAGATGCATGAAGA
TATAATCAGTTTGTGGGATCAAAGCCTAAAGCCATGTGTAAAATTAACCCCACTCTGTGTCACTTTAAATTGC
ACTGATGTGAGAAATAATACTAATACCACTATTAATAATTGGGAAAAGGTGGACAAAGGGGAAATAAAAAACT
GCTCTTTCAATGTCACCACAAGCATAAGAGATAAGATGCAACAAGCATATTCACTTTTTTATAAACTTGATGT
AGTGCAAATAGAGAATACAAGTAGCTATACATTGATAAATTGTAACACCTCAGTCATTACACAGGCCTGTCCA
AAGGTATCCTTTGAACCAATTCCCATACATTATTGTACCCCGGCTGGTTTTGCAATTCTAAAGTGTAATGATA
ATAAGTTCAATGGAACAGGACCATGTACTAATGTTAGCACAGTACAATGTACACATGGAATTAAGCCAGTAGT
GTCAACTCAACTGCTGTTGAATGGCAGTCTGGCAGAAGGAGGAGAGGTAGTAATTAGATCTGAAAATTTCACA
AACAATGCTAAAACCATAATAGTACAGCTGAATACATCTGTAGAAATTAATTGTATAAGACCCAACAACAATA
CAAGAAAAAGTATAACTATAGGACCAGGGAGAGCATTTTATACAACAGACATAATAGGAGATATAAGACAAGC
ACATTGTAACCTTAGTAGAGCAAAATGGAATGACACTTTAAAACAGATAGTTACAAAATTAAAAGAACAATTT
GAGAACAAGACAATAGTCTTTAATCAATCTTCAGGAGGGGACCCAGAAATTGTAATGCACAGCTTTAATTGTG
GAGGGGAATTTTTCTACTGTAATACAACACAGCTGTTTAACAGTACTTGGAATGGTACTGACTGGAATGACAC
TACAGGGTTAGAGAACATCACACTCCCATGCAGAATAAAACAAATTGTAAACAGGTGGCAGGAAGTAGGAAAA
GCAATGTATGCCCCTCCCATCAAAGGACAAATTAGATGTTCATCAAATATTACAGGGCTACTATTAACAAGAG
ATGGGGGAAACAGTAGTGAGATGACCGAGATCTTCAGACCTGGAGGAGGAGATATGAGGGACAATTGGAGAAG
TGAATTATATAAATATAAAGTAGTAAAAATTGAGCCATTAGGAGTAGCACCCACCAAGGCAAAGAGAAGAGTG
GTGCAGAGAGAAAAAAGAGCAGTGGGAACAATAGGAGCTATGTTCCTTGGGTTCTTGGGAGCAGCAGGAAGCA
CTATGGGCGCAGCGTCAATGACGCTGACGGTACAGGCCAGACTATTATTGTCTGGTATAGTGCAACAGCAGAA
CAATTTGCTGAGAGCTATTGAGGCGCAACAGCATCTGTTGCAACTCACAGTCTGGGGCATCAAGCAGCTCCAG
GCTAGAGTCCTGGCTGTGGAAAGATACCTAAGGGATCAACAGCTCCTGGGGATTTGGGGTTGCTCTGGAAAAC
TCATTTGCACCACTAATGTGCCTTGGAATGCTAGTTGGAGTAATAAATCTCTGGAAAAGATTTGGAATAACAT
GACCTGGATGGAGTGGGACAGAGAAATTGACAATTACACAAAATTGATATACACCTTAATTGAAGAATCGCAA
AACCAGCAGGAAAAGAATGAACACGACTTATTGGAGTTGGATAAGTGGGACAGTTTGTGGAATTGGTTTGACA
TAACAAAATGGCTGTGGTATATAAAAATATTCATAATGATAGTAGCAGGTTTAGTAGGTTTAAGAATAGTTTT
TACTGTGCTTTCTATAGTGAATAGAGTTAGGCAGGGATATTCACCACTATCATTTCAGACCCACCACCCAGTC
CCGCGGGGACCCGACAGGCCCGAAGGAATCGAAGAAGGAGGTGGAGAGAGAGACAGAGACACATCCGGACCAT
TAGTGAACGGATTCTTAGCGATTATCTGGGTCGACCTGCGGAGCCTGTGCCTCTTCAGCTACCACCGCTTGAG

AGACTTACTCTTGATTGTAGCGAGGATTGTGGAACTTCTGGGACGCAGGGGGTGGGAGGCCCTGAAATATTGG
TGGAATCTCCTGCAGTATTGGAGTCAGGAACTAAAGAATAGTGCTGTTAGTCTGCTTAATGCCACAGCTATAG
CAGTAGCTGAGGGGACAGATAGGATTATAGAAGTATTACAAAGAGCTTGTAGAGCTATTCTCCACATACCTAG
AAGAATAAGACAGGGCTTAGAAAGGGCTTTGCTATAAGATGGGTGGTAAGTGGTCAAAACGTCGTGCGGGTGG
ATGGGAAGCTGTAAGGGAAAAAATAAGACAAACTGAGCCTGAGCCAGCAGCAGAGGGGGTGGGAGCAGCATCT
CGAGACCTGGAAAAATATGGAGCAATCACCAGTAGCAATACAGCACATACCAACGCTGATTGTGCCTGGGTAG
AAGCACAAGAGGAGGATGAAGAGGTGGGTTTCCCAGTCAGACCTCAGGTACCTTTAAGACCTATGACTTTCAA
GGGAGCGCTAGATCTTAGCCACTTTTTAAAAGAAAAGGGGGGACTGGAAGGGTTAATTCACTCCCAGAAAAGA
CAAGACATCCTTGATTTGTGGGTCTACAACACACAAGGCTATTTCCCTGATTGGCAGAACTACACACCAGGGC
CAGGGGAGAGATTTCCCCTGACCTTTGGATGGTGCTTCAAGCTAGTACCAGTTGATCCAGATCAGGTAGAAGA
GGCTAATAAAGGAGAGACCAACAGCTTGTTACACCCTATGAGCCAGCATGGGATAGAGGACCCGGAGAAAGAA
GTATTAATGTGGAAGTTTGACAGCCGCCTAGCATTTCATCACGTGGCCAGAGAGAAGCATCCGGAGTACTTCA
AGAACTGATGACATCGGGTTTTTCTACAAGGGACTTTCCGCTGGGGACTTTCCAGGGGAGGTGTGGCCTGGGC
GGGACAGGGGAGTGGCGAGCCCTCAGATGCTGCATATAAGCAGCTGCTTTCTGCCTGTACTGGGTCTCTCTGG
TTAGACCAGATTTGAGCCTGGGAGCTCTCTGGCTAACTAGGGAACCCACTGCTTAAGCCTCAATAAAGCTTGC
CT

>AE-C1 HIV-1 genome, derived from RNA genomic sequence
ACTCGGCTTGCTGAGCGCGCACAGCAAGAGGCGAGGGGCGGCGACTGGTGAGTACGCCAAAAATTCTTTTGAC
TAGCGGAGGCTAGAAGGAGAGAGATGGGTGCGAGAGCGTCAATATTAAGCGGGGGAGAATTGGATAGATGGGA
AAAAATTCGGTTAAGGCCAGGGGGAAAGAAACAATATAGACTAAAACATTTAGTATGGGCAAGCAGGGAACTA
GAACGATTCGCAGTTAATCCTGGCCTTTTAGAGACATCAGATGGCTGTAGACAAATACTAGAACAGCTACAAC
CGGCCATTAAGACAGGATCAGAAGAACTTAAATCCTTATATAATACAGTAGCAACCCTCTATTGTGTGCATCA
AAAGATAGAGGTAAGAGACACCAAGGAAGCTTTAGATAAGATAGAGGAAGAGCAAAACAAAAGTAAGAAAAAG
GCGCAGCAAGCAGCAGCTGACACAGGAAACAGCAGCAAGGTCAGTCAAAATTACCCTATAGTACAGAACCTTC
AGGGACAAATGGTACATCAGCCCATATCACCTAGAACTTTAAATGCATGGGTAAAAGTAGTAGAAGAGAAGGC
TTTTAGCCCAGAAGTAATACCCATGTTTTCAGCATTATCAGAAGGAGCCACCCCACAAGATATAAACACCATG
CTAAATACAGTGGGGGGGCATCAAGCAGCCATGCAAATGTTAAAGGAGACCATCAATGAAGAGGCTGCAGAAT
GGGATAGATTGCACCCAGTGCATGCAGGGCCTGTAGCACCAGGCCAGCTGAGAGAACCAAGGGGAAGTGACAT
AGCAGGAACTACTAGTAACCTTCAGGAACAAATAGGATGGATGACAAGCAATCCACCTATCCCAGTAGGAGAA
ATCTATAAAAAATGGATAATCATGGGATTAAATAAAATAGTAAGAATGTATAGCCCTACTAGCATTCTGGACA
TAAAACAAGGACCAAAGGAACCCTTTAGAGACTATGTAGACCGGTTTTATAAAACTCTAAGAGCCGAGCAAGC
TTCACAGGATGTAAAAAATTGGATGACAGAAACCTTGTTGGTCCAAAATGCAAACCCAGATTGTAAGACTATT
TTAAAAGCATTGGGACCAGCAGCTACACTAGAAGAAATGATGACAGCATGTCAGGGAGTGGGGGGACCCGGCC
ATAAAGCAAGAGTTTTGGCTGAAGCAATGAGCCAAGTAACAAATCCAGCTACCATAATGATACAGGGAGGCAA
ATTTAGGAACCAAAGAAAAACTATTAAGTGCTTCAACTGTGGCAAAGAGGGGCACATAGCCAGAAATTGCAGG
GCCCCTAGAAAAAAGGGCTGTTGGAAATGTGGAAGGGAAGGACACCAAATGAAAGATTGTACTGAGAGACAGG
CTAATTTTTTAGGGAAAATCTGGCCTTCCCACAAAGGAAGGCCAGGGAATTTTCTTCAGAACAGACCAGAGCC
AACAGCCCCACCAGAGGAGAGCCTCAGGGAAGAGACAGCAGCCCCCTCTCAGAGGCAGGGACCGATAGACAAG
GAACTGTATCCTTTAGCTTCCCTCAAATCACTCTTTGGCAACGACCCCTCGTCACAATAAAGATAGGGGGGCA
ACTAAAGGAAGCTCTATTAGATACAGGAGCAGATGATACAGTATTAGAAGAAATGAATTTGTCAGGAAGATGG
AAACCAAAAATGATAGGAGGAATTGGAGGTTTTATCAAAGTAAGACAGTATGATCAAATACCCATAGAAATCT
GCGGACATAAAGCTGTAGGTACAGTATTAGTAGGACCGACACCTGTCAACATAATTGGAAGAAATCTGTTGAC
TCAGATTGGCTGCACTTTAAATTTTCCTATTAGTCCTATTGAAACTGTACCAGTAAAATTAAAGCCAGGAATG
GATGGGCCAAAGGTTAAACAATGGCCATTGACAGAAGAAAAAATAAAAGCATTAGTAGAAATTTGTACAGAAA
TGGAAAAGGAAGGAAAAATTTCAAAAATTGGGCCTGAAAATCCATACAATACTCCAGTATTTGCCATAAAGAA
AAAAGACAGTACTAAATGGAGAAAATTAGTAGACTTTAGAGAACTTAATAAGAGAACTCAAGACTTCTGGGAA
GTTCAATTAGGAATACCACATCCTGCAGGGTTAAAAAAGAAAAAATCAGTAACAGTACTGGATGTGGGTGATG
CATATTTTTCAGTTCCATTAGATAAAGAATTCAGGAAGTATACTGCATTTACCATACCTAGTATAAACAATGA
GACACCAGGGATTAGATATCAGTACAATGTGCTTCCACAGGGGTGGAAAGGATCCCCAGCCATATTCCAAAGT
AGTATGACAAAAATCTTAGAGCCTTTTAGAAAACAAAATCCAGAAATGGTTATCTATCAATACATGGATGATT
TATATGTAGGATCTGACTTAGAAATAGGGCAGCATAGAGCAAAAATAGAGGAACTGAGACAACATCTGTTGAG
GTGGGGATTTACCACACCAGACAAAAAGCATCAGAAAGAACCTCCATTCCTTTGGATGGGTTATGAGCTCCAT
CCTGATAAATGGACAGTACAGCCTATAAAGCTGCCAGAAAAAGACAGCTGGACTGTCAATGACATACAGAAGT
TAGTGGGAAAATTAAATTGGGCAAGTCAGATCTATCCAGGGATTAAAGTAAGGCAATTATGTAAACTCCTTAG
GGGAGCCAAAGCACTAACAGAAGTAGTACCACTAACAGCAGAAGCAGAGCTAGAATTGGCAGAAAACAGGGAG
ATACTAAAAGAACCAGTACATGGAGTGTATTATGACCCATCAAAAGACTTAATAGCAGAAATACAGAAGCAGG
GGCAAGGCCAGTGGACATATCAAATTTATCAAGAGCCATTTAAAAATTTGAAAACAGGAAAGTATGCAAGAAT
GAAGGGTGCCCACACTAATGATGTAAAACAGTTAACAGAGGCAGTGCAAAAAATAGCCACAGAAAGCATAATA
ATATGGGGAAAAACTCCTAAATTTAGATTACCCATACAAAAAGAAACATGGGAAGCATGGTGGACAGAGTATT
GGCAAGCCACCTGGATTCCTGAGTGGGAATTTGTCAATACCCCTCCCTTAGTAAAATTATGGTACCAGTTAGA
GAAAGAACCCATAATAGGAGCAGAAACTTTCTATGTAGATGGGGCAGCTAATAGGGAGACTAAATTAGGAAAA
GCAGGATATGTTACTGACAGAGGAAGACAAAAGGTTGTCTCCCTAACTGACACAACAAATCAGAAGACTGAGT
TACAAGCGATCCATCTAGCTTTGCAGGATTCGGGGCTAGAAGTAAACATAGTAACAGACTCACAATATGCATT
AGGAATCATTCAAGCACAACCAGATAAGAGTGAATCAGAGTTAGTCAGTCAAATAATAGAGCAGTTAATAAAA
AAGGAAAAGGTCTACTTAGCATGGGTACCAGCACACAAGGGAATTGGAGGAAATGAACAAGTAGATAAATTAG
TCAGTGCTGGAATCAGGAAAGTACTGTTTTTGGATGGAATAGATAAGGCCCAAGAAGAACATGAGAAATATCA
CAATAATTGGAGAGCAATGGCTAGTGATTTTAATCTGCCACCTATAGTAGCAAAAGAAATAGTAGCCAGCTGT
GATAAATGTCAGCTAAAAGGAGAAGCCATGCATGGACAAGTAGACTGTAGTCCAGGAATATGGCAATTAGATT
GCACACATTTAGAAGGAAAAATTATCCTGGTAGCAGTCCATGTAGCCAGTGGATATATAGAAGCAGAAGTTAT
CCCAGCAGAGACAGGGCAGGAAACAGCATACTTTATCTTAAAATTAGCAGGAAGATGGCCAGTAAAAACAATA

CATACAGACAATGGCAGCAATTTTATCAGTAATACAGTTAAGGCTGCCTGTTGGTGGGCGGGGATCAAGCAAG
AATTTGGTATTCCCTACAATCCCCAAAGTCAAGGAGTAGTAGAATCTATGAATAAAGAATTAAAGAAAATTAT
AGGACAGGTAAGAGATCAGGCTGAACATCTTAAAACAGCAGTACAAATGGCAGTATTCATCCACAATTTTAAA
AGAAAAGGGGGGATTGGGGGATACAGTGCAGGGGAGAGAATAGTAGACATAATAGCAACAGACATACAAACTA
AAGAACTACAAAAACAAATTACAAAAATTCAAAATTTTCGGGTTTATTACAGGGACAACAGAGATCCACTTTG
GAAAGGACCAGCAAAGCTTCTTTGGAAAGGTGAAGGGGCAGTAGTAATACAAGAGGATAGTGAAATAAAAGTA
GTGCCAAGAAGAAAAGCAAAGATCATTAGGGATTATGGAAAACAGATGGCAGGTGATGATTGTGTGGCAAGTA
GACAGGATGAAGATTAGAACATGGAATAGTTTAGTAAAACACCATATGTATATTTCAAGGAAAGCTAAGGAAT
GGGTTTATAAGCATCACTATGAAAGCACTAATCCAAGAGTAAGCTCAGAAGTACACATCCCACTAGGGGAGGC
TAAATTGGTGATAACAACATATTGGGGTCTGCATGCAGGAGAAAGAGAGTGGCATTTGGGTCATGGAGCCTCC
ATAGAATGGAGGAAAAAGAGATATAGCACACAAGTAGACCCTGACCTAGCAGACCAACTAATTCATCTGCATT
ACTTTGATTGTTTTTCAGAATCTGCTATAAGACATGCCATATTAGGACGTATAGTTAGTCCTAGGTGTGAATA
TCAAGCAGGACATAACAAGGTAGGATCTCTACAGTACTTGGCACTAGCAGCATTAATAAAACCAAAAAAGACA
AAGCCACCTTTGCCTAGTGTTAGGAAACTGACAGAGGATAGATGGAACAAGCCCCAGAAGACCATGGGCCACA
GAGGGAGCCATACAATGAATGGACACTAGAGCTTTTAGAGGAACTTAAGAATGAAGCTGTTAGACATTTTCCT
AGGCCATGGCTCCATGGCTTAGGACAATATATCTATGAAACTTATGGGGATACTTGGACAGGAGTGGAAGCCA
TAATAAGAATTCTGCAACAACTGCTGTTTATTCATTTCAGAATTGGATGTCAACATAGCAGAATAGGTATTCT
ACCGAGGAGAGCAAGGAATGGAGCCAGTAGATCCTAGTTTAGAGCCCTGGAAGCATCCAGGAAGCCGGCCTAA
AACTGCTTGTACCAATTGCTATTGTAAAAAGTGTTGCTTTCATTGCCAAGTGTGTTTCACAACCAAAGCCTTA
GGCATCTCCTATGGCAGGAAGAAGCGGAGACAGCGACGAAGAGCTCCTCAAGGCAGTCAGACTCATCAAGTTT
CTCTACCAAAGCAGTGAGTAATATATGTAATGCAATCCTTAGAAATATATGCAATAGTAGCATTAGTAGTAGT
AGCAATAATAGCAATAGTTGTGTGGACCATAGTGCTCCTAGAATATAGGAAAATATTAAGACAAAGGAAAATA
GACAGATTAATTGATAGAATAAGAGAAAGAGCAGAAGACAGTGGCAATGAAAGTGAAGGGGATGAGGAGGAAT
TATCAGCCCTTGTGGAAATGGGGCATCATGCTCCTTGGGATGTTGATGATCTGTAGTGCTACAGAAAAATTGT
GGGTCACAGTCTATTATGGGGTACCTGTGTGGAAAGAAGCAACCACCACTTTATTTTGTGCATCAGATGCTAA
AGCATATGATACAGAGATGCATAATGTTTGGGCCACACATGCCTGTGTACCCACAGACCCTAGCCCACAAGAA
GTAGTATTGGAAAATGTGACAGAAAATTTTAACATGTGGAAAAATAACATGGTAGAACAGATGCATGAAGATA
TAATCAGTTTGTGGGATCAAAGCCTAAAGCCATGTGTAAAATTAACCCCACTCTGTGTCACTTTAAATTGCAC
TGATGTGAGAAATAATACTAATACCACTGTTAGTAATTGGGAAAAAGTAGACAGAGGGGAAATAAAAAACTGC
TCTTTCAATGTCACCACAAGCATAAGAGATAAGATGCAACAAGCATATTCACTTTTTTATAAACTTGATGTAG
TACAAATAGAAAATACAAGTAGCTATACATTGATAAATTGTAACACCTCAGTCATTACACAGGCCTGTCCAAA
GGTATCCTTTGAACCAATTCCCATACATTATTGTACCCCGGCTGGTTTTGCGATTCTAAAGTGTAATGATAAT
AAGTTCAATGGAACAGGACCATGTACTAATGTTAGCACAGTACAATGTACACATGGAATTAAGCCAGTAGTGT
CAACTCAATTGCTGTTGAATGGCAGTCTGGCAGAAGGAGGAGAGGTAGTAATTAGATCTGAAAATTTCACAAA
CAATGCTAAAACCATAATAGTACAGCTGAATACATCTGTAGAAATTAATTGTATAAGACCCAACAACAATACA
AGAAAAAGTATAACTATAGGACCAGGGAGAGCATTTTATACAACAGACATAATAGGAGATATAAGACAAGCAC
ATTGTAACCTTAGTAGAGCAAAATGGAATGACACTTTAAAACAGATAGTTACAAAATTAAAAGAACAATTTGA
GAACAAGACAATAGTCTTTAATCAATCTTCAGGAGGGGACCCAGAAATTGTAATGCACAGCTTTAATTGTGGA
GGGGAATTTTTCTACTGTAATACAACACAGCTGTTTAACAGTACTTGGAATGGTACTGCCTGGAATGATACTA
CAGGGTCAGAGAACATCACACTCCCATGCAGAATAAAACAAATTGTAAACAGGTGGCAGGAAGTAGGAAAAGC
AATGTATGCCCCTCCCATCAAAGGACAAATTAGATGTTCATCAAATATTACAGGGCTACTATTAACAAGAGAT
GGTGGAAATAGTAATAACACGACCGAGATCTTCAGACCTGGAGGAGGAGATATGAGGGACAATTGGAGAAGTG
AATTATATAAATATAAAGTAGTAAAAATTGAACCATTGGGAGTAGCACCCACCAAGGCAAAGAGAAGAGTGGT
GCAGAGAGAAAAAAGAGCAGTGGGAACAATAGGAGCTATGTTCCTTGGGTTCTTGGGAGCAGCAGGAAGCACT
ATGGGCGCAGCGTCAATGACGCTGACGGTACAGGCCAGACTATTATTGTCTGGTATAGTGCAACAGCAGAACA
ATTTGCTGAGAGCTATTGAGGCGCAACAGCATTTGTTGCAACTCACAGTCTGGGGCATCAAGCAGCTCCAGGC
TAGAGTCCTGGCTGTGGAAAGATACCTAAGGGATCAACAGCTCCTGGGGATTTGGGGTTGCTCTGGAAAACTC
ATTTGCACCACTAATGTGCCTTGGAATGCTAGTTGGAGTAATAAATCTTTAGAAAAGATTTGGAATAACATGA
CCTGGATGGAGTGGGACAGAGAAATTGACAATTACACAAAATTGATATACACCTTAATTGAAGAATCGCAAAA
CCAGCAGGAAAAGAATGAACAAGACTTATTGGAGTTGGATAAGTGGGACAGTTTGTGGAATTGGTTTGACATA
ACAAAATGGCTGTGGTATATAAAAATATTCATAATGATAGTAGCAGGTTTAGTAGGTTTAAGAATAGTTTTTA
CTGTGCTTTCTATAGTGAATAGAGTTAGGCAGGGATATTCACCACTATCATTTCAGACCCACCACCCAGTCCC
GCGGGGACCCGACAGGCCCGAAGGAATCGAAGAAGGAGGTGGAGAGAGAGACAGAGACACATCCGGACCATTA
GTGAACGGATTCTTAGCGATTATCTGGGTCGACCTGCGGAGCCTGTGCCTCTTCAGCTACCACCGCTTGAGAG

ACTTACTCTTGATTGTAGCGAGGATTGTGGAACTTCTGGGACGCAGGGGGTGGGAGGCCCTGAAATATTGGTG
GAATCTCCTGCAGTATTGGAGTCAGGAACTAAAGAATAGTGCTGTTAGTCTGCTTAATGCCACAGCTATAGCA
GTAGCTGAGGGGACAGATAGGATTATAGAAGTATTACAAAGAGCTTGTAGAGCTATTCTCCACATACCTAGAA
GAATAAGACAGGGCTTAGAAAGGGCTTTGCTATAAGATGGGTGGTAAGTGGTCAAAACGTCGTGCGAGTGGAT
GGGAAGCTGTAAGGGAAAAAATAAGACAAACTGAGCCTGAGCCAGCAGCAGAGGGGGTGGGAGCAGCATCTCG
AGACCTGGAAAAATATGGAGCAATCACCAGTAGCAATACGGCACATACCAATGCTGATTGTGCCTGGGTAGAA
GCACAAGAGGAGGGTGAAGAGGTGGGTTTCCCAGTCAGACCTCAGGTACCTCTAAGACCAATGACTTTCAAGG
GAGCGCTAGATCTTAGCCACTTTTTAAAAGAAAAGGGGGGACTGGAAGGGTTAATTCACTCCCAGAAAAGACA
AGATATCCTTGATTTGTGGGTCTACAACACACAAGGCTATTTCCCTGATTGGCAGAACTACACACCAGGGCCA
GGGGAGAGATTCCCACTGACCTTTGGATGGTGCTTCAAGCTAGTACCAGTTGATCCAGATCAGGTAGAAGAGG
CTAATAAAGGAGAGAACAACAGCTTGTTACACCCTATGAGCCAGCATGGGATGGAGGACCCGGAGAAAGAAGT
ATTAATGTGGAAGTTTGACAGCCGCCTAGCATTTCATCACGTGGCCAGAGAGAAGCATCCGGAGTACTTCAAG
AACTGATGACATCGGGTTTTCTACAAGGGACTTTCCGCTGGGGACTTTCCAGGGAGGTGTGGCCTGGGCGGGA
CAGGGGAGTGGCGAGCCCTCAGATGCTGCATATAAGCAGCTGCTTTCTGCCTGTACTGGGTCTCTCTGGTTAG
ACCAGATTTGAGCCTGGGAGCTCTCTGGCTAGCTAGGGAACCCACTGCTTAAGCCTCAATAAAGCTTGCCT

```
>AE-C4 HIV-1 genome, derived from RNA genomic sequence
ACTCGGCTTGCTGAGCGCGCACAGCAAGAGGCGAGGGGCGGCGACTGGTGAGTACGCCAAAACTTTTTTGACT
AGCGGAGGCTAGAAGGAGAGAGATGGGTGCGAGAGCGTCAATATTAAGCGGGGGAGAATTAGATAGATGGGAA
AAAATTCGGTTAAGGCCAGGGGGAAAGAAACAATATAGACTAAAACATTTAGTATGGGCAAGCAGGGAACTAG
AACGATTCGCAGTTAATCCTGGCCTTTTAGAGACATCAGATGGCTGTAGACAAATACTAGAACAGCTACAACC
AGCCATTAAGACAGGATCAGAAGAACTTAAATCCTTATATAATACAGTAGCAACCCTCTATTGTGTGCATCAA
AAGATAGAGGTAAGAGACACCAAGGAAGCTTTAGATAAGATAGAGGAAGAGCAAAACAAAAGTAAGAAAAAGG
CACAGCAAGCAGCAGCTGACACAGGAAACAGCAGCAAGGTCAGTCAAAATTACCCTATAGTACAGAACCTTCA
GGGACAAATGGTACATCAACCCATATCACCTAGAACTTTAAATGCATGGGTAAAAGTAGTAGAAGAGAAGGCT
TTCAGCCCAGAAGTAATACCCATGTTTTCAGCATTATCAGAAGGAGCCACCCCACAAGATATAAACACCATGC
TAAATACAGTGGGGGGACATCAAGCAGCCATGCAAATGTTAAAGGAGACCATCAATGAAGAGGCTGCAGAATG
GGATAGATTGCACCCAGTGCATGCAGGGCCTGTAGCACCAGGCCAGCTGAGAGAACCAAGGGGAAGTGACATA
GCAGGAACTACTAGTAACCTGCAGGAACAAATAGGATGGATGACAAGTAATCCACCTATCCCAGTAGGAGAAA
TCTATAAAAAATGGATAATCATGGGATTAAATAAAATAGTAAGAATGTATAGCCCTACCAGCATTCTGGACAT
AAAACAAGGACCAAAGGAACCCTTTAGAGACTATGTAGACCGGTTCTATAAAACTCTAAGAGCCGAGCAAGCT
TCACAGGATGTAAAAAATTGGATGACAGAAACCTTGTTGGTCCAAAATGCAAACCCAGATTGTAAGACTATTT
TAAAAGCATTGGGACCAGCAGCTACACTAGAAGAAATGATGACAGCATGTCAGGGAGTGGGGGGACCCGGCCA
TAAAGCAAGAGTTTTGGCTGAAGCAATGAGCCAAGTAACAAATCCAGCTACCATAATGATACAGGGAGGCAAA
TTTAGGAACCAAAGAAAAACTATTAAGTGCTTCAACTGTGGCAAAGAGGGGCACTTGGCCAGAAATTGCAGGG
CCCCTAGAAAAAAGGGCTGTTGGAAATGTGGAAGGGAAGGACACCAAATGAAAGATTGTACTGAGAGACAGGC
TAATTTTTTAGGGAAAATCTGGCCTTCCCACAAAGGAAGGCCAGGGAATTTTCTTCAGAACAGACCAGAGCCA
ACAGCCCCACCAGAGGAGAGCCTCAGGGAAGAGACAGCAGCCCCCTCTCAGAGGCAGGGACCGATAGACAAGG
AACTGTATCCTTTAGCTTCCCTCAAATCACTCTTTGGCAACGACCCCTCGTCACAATAAAGATAGGGGGGCAA
CTAAAGGAAGCTCTATTAGATACAGGAGCAGATGATACAGTATTAGAAGAAATAAATTTGTCAGGAAGATGGA
AACCAAAAATGATAGGAGGAATTGGAGGTTTTATCAAAGTAAGACAGTATGATCAAATACCCATAGAAATCTG
CGGACACAAAGCTGTAGGTACAGTATTAGTAGGACCGACACCTGTCAACATAATTGGAAGAAATCTGTTGACT
CAGATTGGCTGCACTTTAAATTTTCCTATTAGTCCTATTGAAACTGTACCAGTAAAATTAAAGCCAGGAATGG
ATGGGCCAAAGGTTAAACAATGGCCATTGACAGAAGAAAAAATAAAAGCATTAGTAGAAATTTGTACAGAAAT
GGAAAAGGAAGGAAAAATTTCAAAAATTGGGCCTGAAAATCCATACAATACTCCAGTATTTGCCATAAAGAAA
AAAGACAGTACTAAATGGAGAAAATTAGTAGACTTTAGAGAACTTAATAAGAGAACTCAAGACTTCTGGGAAG
TTCAATTAGGAATACCACATCCTGCAGGGTTAAAAAAGAAAAAATCAGTAACAGTACTGGATGTGGGTGATGC
ATACTTTTCAGTTCCATTAGATAAAGAATTCAGGAAGTATACTGCATTTACCATACCTAGTATAAACAATGAG
ACACCAGGGATTAGATATCAGTACAATGTGCTTCCACAGGGATGGAAAGGATCCCCAGCCATATTCCAAAGTA
GTATGACAAAAATCTTAGAGCCTTTTAGAAAACAAAACCCAGAAATGGTTATCTATCAATACATGGATGATTT
ATATGTAGGATCTGACTTAGAAATAGGGCAGCATAGAGCAAAAATAGAGGAACTGAGACAGCATCTGTTGAGG
TGGGGATTTACCACACCAGACAAAAAGCATCAGAAAGAACCTCCATTCCTTTGGATGGGTTATGAGCTCCATC
CTGATAAATGGACAGTACAGCCTATAAAGCTGCCAGAAAAAGACAGCTGGACTGTCAATGACATACAGAAGTT
AGTGGGAAAATTGAATTGGGCAAGTCAGATCTATCCAGGGATTAAAGTAAGGCAATTATGTAAACTCATTAGG
GGAACCAAAGCACTAACAGAAGTAGTACCACTAACAGCAGAAGCAGAGCTAGAATTGGCAGAAAACAGGGAGA
TACTAAAAGAACCAGTACATGGAGTGTATTATGACCCATCAAAAGACTTAATAGCTCTCT?GCAGTGGCAGGG
GCAAGGCCAGTGGACATATCAAATTTATCAAGAGCCATTTAAAAATTTGAAAACAGGAAAGTATGCAAGAATG
AAGGGTGCCCACACTAATGATGTAAAACAGTTAACAGAGGCAGTGCAAAAAATAGCCACAGAAAGCATAGTAA
TATGGGGAAAAACTCCTAAATTTAGATTACCCATACAAAAAGAAACATGGGAAGCATGGTGGACAGAGTATTG
GCAAGCCACCTGGATTCCTGAGTGGGAATTTGTCAATACCCCTCCCTTAGTAAAATTATGGTACCAGCTAGAG
AAAGAACCCATAATAGGAGCAGAAACTTTCTATGTAGATGGGGCAGCTAATAGGGAAACTAAATTAGGAAAAG
CAGGATATGTTACTGACAGAGGAAGACAAAAGGTTGTCTCCCTAACTGACACAACAAATCAGAAGACTGAGTT
ACAAGCGATCCATCTAGCTTTGCAGGATTCGGGGCTAGAAGTAAACATAGTAACAGACTCACAATATGCATTA
GGAATCATTCAAGCACAACCAGATAAGAGTGAATCAGAGTTAGTCAGTCAGATAATAGAGCAGTTAATAAAAA
AGGAAAAGGTCTATTTAGCATGGGTACCAGCACACAAGGGAATTGGAGGAAATGAACAAGTAGATAAATTAGT
CAGTGCTGGAATCAGGAAAGTACTATTTTTGGATGGAATAGATAAGGCCCAAGAAGAACATGAGAAATATCAC
AATAATTGGAGAGCAATGGCTAGTGATTTTAACCTGCCACCTGTAGTAGCAAAAGAAATAGTAGCCAGCTGTG
ATAAATGTCAGTTAAAAGGAGAAGCCATGCATGGACAAGTAGACTGTAGTCCAGGAATATGGCAATTAGATTG
CACACATTTAGAAGGAAAAATTATCCTGGTAGCAGTCCATGTAGCCAGTGGATATATAGAAGCAGAAGTTATC
CCAGCAGAGACAGGGCAGGAAACAGCATACTTTATCTTAAAATTAGCAGGAAGATGGCCAGTAAAAACAATAC
```

ATACAGACAATGGCAGCAATTTTATCAGTAATACAGTTAAGGCTGCCTGTTGGTGGGCGGGGATCAAGCAAGA
ATTTGGCATTCCCTACAATCCCCAAAGTCAAGGAGTAGTAGAATCTATGAATAAAGAATTAAAGAAGATTATA
GGACAGGTAAGAGATCAGGCTGAACATCTTAAAACAGCAGTACAAATGGCAGTATTCATCCACAATTTTAAAA
GAAAAGGGGGGATTGGGGGATACAGTGCAGGGGAAAGAATAGTAGACATAATAGCAACAGACATACAAACTAA
AGAACTACAAAAACAAATTACAAAAATTCAAAATTTTCGGGTTTATTACAGGGACAACAGAGATCCACTTTGG
AAAGGACCAGCAAAGCTTCTTTGGAAAGGTGAAGGGGCAGTAGTAATACAAGAGGATAGTGAAATAAAAGTAG
TGCCAAGAAGAAAAGCAAAGATCATTAGGGATTATGGAAAACAGATGGCAGGTGATGATTGTGTGGCAAGTAG
ACAGGATGAAGATTAGAACATGGAATAGTTTAGTAAAACACCATATGTATATTTCAAGGAAAGCTCAGGGATG
GGTTTATAGGCATCACTATGAAAGCACTAATCCAAGAGTAAGCTCAGAAGTACACATCCCACTAGGGGATGAT
AAATTGGTGATAACAACATATTGGGGTCTGCATGCAGGAGAAAGAGAGTGGCATTTGGGTCATGGAGCCTCCA
TAGAATGGAGGAAAAGAAGATATAGCACACAAGTAGACCCTGACCTAGCAGACCAACTAATTCATCTGCATTA
CTTTGATTGTTTTTCAGAATCTGCTATAAGACATGCCATATTAGGACGTATAGTTAGTCCTAGGTGTGAATAT
CAAGCAGGACATAACAAGGTAGGATCTCTACAGTACTTGGCACTAGCAGCATTAATAAAACCAAAAAAGAGAA
AGCCACCTTTGCCTAGTGTTAGGAAACTGACAGAGGATAGATGGAACAAGCCCCAGAAGACCATGGGCCACAG
AGGGAGCCATACAATGAATGGACACTAGAACTTTTAGAGGAACTTAAGAATGAAGCTGTTAGACATTTTCCTA
GGCCATGGCTCCATAGCTTAGGACAATATATCTATGAAACTTATGGGGATACTTGGACAGGAGTGGAAGCCAT
AATAAGAATTCTGCAACAACTGCTGTTTATTCATTTCAGAATTGGATGTCAACATAGCAGAATAGGTATTCTA
CCAAGGAGAGCAAGGAATGGAGCCAGTAGATCCTAGTTTAGAGCCCTGGAAGCATCCAGGAAGCCGGCCTAAA
ACTGCTTGTACCAATTGTTATTGTAAAAAGTGTTGCTTTCATTGCCAAGTGTGTTTCACAACCAAAGCCTTAG
GCATCTCCTATGGCAGGAAGAAGCGGAGACAGCGACGAAGAGCTCCTCCAAGCAGTCAGAATCATCAAGTTTC
TCTACCAAAGCAGTGAGTAATATATGTAATGCAATCCTTAGAAATATATGCAATAGTAGCATTAGTAGTAGTA
GCAATAATAGCAATAGTTGTGTGGACCATAGTGCTCCTAGAATATAGGAAAATATTAAGACAAAGGAAAATAG
ACAGATTAATTGATAGAATAAGAGAAAGAGCAGAAGACAGTGGCAATGAAAGTGAAGGGGATGAGGAGGAATT
ATCAGCCCTTGTGGAAATGGGGCATCATGCTCCTTGGGATGTTGATGATCTGTAGTGCTACAGAAAAATTGTG
GGTCACAGTCTATTATGGGGTACCTGTGTGGAAAGAAGCAACCACCACTTTATTTTGTGCATCAGATGCTAAA
GCATATCATACAGAGATGCATAATGTTTGGGCCACACATGCCTGTGTACCCACAGACCCTAGCCCACAAGAAG
TAGTATTGGGAAATGTGACAGAAAATTTTAACATGTGGAAAAATAACATGGTAGAACAGATGCATGAAGATAT
AATCAGTTTGTGGGATCAAAGCCTAAAGCCATGTGTAAAATTAACCCCACTCTGTGTCACTTTAAATTGCACT
GATGTGAGAAATAATACTAATACCACTATTAATAATTGGGAAAAGGTGGACAAAGGGGAAATAAAAAACTGCT
CTTTCAATGTCACCACAAGCATAAGAGATAAGATGCAACAAGCATATTCACTTTTTTATAAACTTGATGTAGT
GCAAATAGAGAATACAAGTAGCTATACATTGATAAATTGTAACACCTCAGTCATTACACAGGCCTGTCCAAAG
GTATCCTTTGAACCAATTCCCATACATTATTGTACCCCGGCTGGTTTTGCAATTCTAAAGTGTAATGATAATA
AGTTCAATGGAACAGGACCATGTACTAATGTTAGCACAGTACAATGTACACATGGAATTAAGCCAGTAGTGTC
AACTCAACTGCTGTTGAATGGCAGTCTGGCAGAAGGAGGAGAGGTAGTAATTAGATCTGAAAATTTCACAAAC
AATGCTAAAACCATAATAGTACAGCTGAATACATCTGTAGAAATTAATTGTATAAGACCCAACAACAATACAA
GAAAAAGTATAACTATAGGACCAGGGAGAGCATTTTATACAACAGACATAATAGGAGATATAAGACAAGCACA
TTGTAACCTTAGTAGAGCAAAATGGAATGACACTTTAAAACAGATAGTTACAAAATTAAAAGAACAATTTGAG
AACAAGACAATAGTCTTTAATCAATCTTCAGGAGGGGACCCAGAAATTGTAATGCACAGCTTTAATTGTGGAG
GGGAATTTTTCTACTGTAATACAACACAGCTGTTTAACAGTACTTGGAATGGTACTGACTGGAATGACACTAC
AGGGTTAGAGAACATCACACTCCCATGCAGAATAAAACAAATTGTAAACAGGTGGCAGGAAGTAGGAAAAGCA
ATGTATGCCCCTCCCATCAAAGGACAAATTAGATGTTCATCAAATATTACAGGGCTACTATTAACAAGAGATG
GGGGAAACAGTAGTGAGATGACCGAGATCTTCAGACCTGGAGGAGGAGATATGAGGGACAATTGGAGAAGTGA
ATTATATAAATATAAAGTAGTAAAAATTGAGCCATTAGGAGTAGCACCCACCAAGGCAAAGAGAAGAGTGGTG
CAGAGAGAAAAAAGAGCAGTGGGAACAATAGGAGCTATGTTCCTTGGGTTCTTGGGAGCAGCAGGAAGCACTA
TGGGCGCAGCGTCAATGACGCTGACGGTACAGGCCAGACTATTATTGTCTGGTATAGTGCAACAGCAGAACAA
TTTGCTGAGAGCTATTGAGGCGCAACAGCATCTGTTGCAACTCACAGTCTGGGGCATCAAGCAGCTCCAGGCT
AGAGTCCTGGCTGTGGAAAGATACCTAAGGGATCAACAGCTCCTGGGGATTTGGGGTTGCTCTGGAAAACTCA
TTTGCACCACTAATGTGCCTTGGAATGCTAGTTGGAGTAATAAATCTCTGGAAAAGATTTGGAATAACATGAC
CTGGATGGAGTGGGACAGAGAAATTGACAATTACACAAAATTGATATACACCTTAATTGAAGAATCGCAAAAC
CAGCAGGAAAAGAATGAACACGACTTATTGGAGTTGGATAAGTGGGACAGTTTGTGGAATTGGTTTGACATAA
CAAAATGGCTGTGGTATATAAAAATATTCATAATGATAGTAGCAGGTTTAGTAGGTTTAAGAATAGTTTTTAC
TGTGCTTTCTATAGTGAATAGAGTTAGGCAGGGATATTCACCACTATCATTTCAGACCCACCACCCAGTCCCG
CGGGGACCCGACAGGCCCGAAGGAATCGAAGAAGGAGGTGGAGAGAGAGACAGAGACACATCCGGACCATTAG
TGAACGGATTCTTAGCGATTATCTGGGTCGACCTGCGGAGCCTGTGCCTCTTCAGCTACCACCGCTTGAGAGA

CTTACTCTTGATTGTAGCGAGGATTGTGGAACTTCTGGGACGCAGGGGGTGGGAGGCCCTGAAATATTGGTGG
AATCTCCTGCAGTATTGGAGTCAGGAACTAAAGAATAGTGCTGTTAGTCTGCTTAATGCCACAGCTATAGCAG
TAGCTGAGGGGACAGATAGGATTATAGAAGTATTACAAAGAGCTTGTAGAGCTATTCTCCACATACCTAGAAG
AATAAGACAGGGCTTAGAAAGGGCTTTGCTATAAGATGGGTGGTAAGTGGTCAAAACGTCGTGCGGGTGGATG
GGAAGCTGTAAGGGAAAAAATAAGACAAACTGAGCCTGAGCCAGCAGCAGAGGGGGTGGGAGCAGCATCTCGA
GACCTGGAAAAATATGGAGCAATCACCAGTAGCAATACAGCACATACCAACGCTGATTGTGCCTGGGTAGAAG
CACAAGAGGAGGATGAAGAGGTGGGTTTCCCAGTCAGACCTCAGGTACCTTTAAGACCTATGACTTTCAAGGG
AGCGCTAGATCTTAGCCACTTTTTAAAAGAAAAGGGGGGACTGGAAGGGTTAATTCACTCCCAGAAAAGACAA
GACATCCTTGATTTGTGGGTCTACAACACACAAGGCTATTTCCCTGATTGGCAGAACTACACACCAGGGCCAG
GGGAGAGATTTCCCCTGACCTTTGGATGGTGCTTCAAGCTAGTACCAGTTGATCCAGATCAGGTAGAAGAGGC
TAATAAAGGAGAGACCAACAGCTTGTTACACCCTATGAGCCAGCATGGGATAGAGGACCCGGAGAAAGAAGTA
TTAATGTGGAAGTTTGACAGCCGCCTAGCATTTCATCACGTGGCCAGAGAGAAGCATCCGGAGTACTTCAAGA
ACTGATGACATCGGGTTTTTCTACAAGGGACTTTCCGCTGGGGACTTTCCAGGGGAGGTGTGGCCTGGGCGGG
ACAGGGGAGTGGCGAGCCCTCAGATGCTGCATATAAGCAGCTGCTTTCTGCCTGTACTGGGTCTCTCTGGTTA
GACCAGATTTGAGCCTGGGAGCTCTCTGGCTAACTAGGGAACCCACTGCTTAAGCCTCAATAAAGCTTGCCT

>AE-C8 HIV-1 genome, derived from RNA genomic sequence

GGGGGAATTGGAGGTTTTATCAAAGTAAGACAGTATGATCAAATACCCATAGAAATCTGCGGACACAAAGCTG
TAGGTACAGTATTAGTAGGACCGACACCTGTCAACATAATTGGAAGAAATCTGTTGACTCAGATTGGCTGCAC
TTTAAATTTTCCTATTAGTCCTATTGAAACTGTACCAGTAAAATTAAAGCCAGGAATGGATGGGCCAAAGGTT
AAACAATGGCCATTGACAGAAGAAAAAATAAAAGCATTAGTAGAAATTTGTACAGAAATGGAAAAGGAAGGAA
AAATTTCAAAAATTGGGCCTGAAAATCCATACAATACTCCAGTATTTGCCATAAAGAAAAAAGACAGTACTAA
ATGGAGAAAATTAGTAGACTTTAGAGAACTTAATAAGAGAACTCAAGACTTCTGGGAAGTTCAATTAGGAATA
CCACATCCTGCAGGGTTAAAAAAGAAAAAATCAGTAACAGTACTGGATGTGGGTGATGCATACTTTTCAGTTC
CATTAGATAAAGAATTCAGGAAGTATACTGCATTTACCATACCTAGTATAAACAATGAGACACCAGGGATTAG
ATATCAGTACAATGTGCTTCCACAGGGATGGAAAGGATCCCCAGCCATATTCCAAAGTAGTATGACAAAAATC
TTAGAGCCTTTTAGAAAACAAAACCCAGAAATGGTTATCTATCAATACATGGATGATTTATATGTAGGATCTG
ACTTAGAAATAGGGCAGCGTAGAGCAAAAATAGAGGAACTGAGACAGCATCTGTTGAGGTGGGGATTTACCAC
ACCAGACAAAAAGCATCAGAAAGAACCTCCATTCCTTTGGATGGGTTATGAGCTCCATCCTGATAAATGGACA
GTACAGCCTATAAAGCTGCCAGAAAAAGACAGCTGGACTGTCAATGACATACAGAAGTTAGTGGGAAAATTGA
ATTGGGCAAGTCAGATCTATCCAGGGATTAAAGTAAGGCAATTATGTAAACTCATTAGGGGAACCAAAGCACT
AACAGAAGTAGTACCACTAACAGCAGAAGCAGAGCTAGAATTGGCAGAAAACAGGGAGATACTAAAAGAACCA
GTACATGGAGTGTATTATGACCCATCAAAAGACTTAATAGCAGAAATACAGAAGCAGGGGCAAGGCCAGTGGA
CATATCAAATTTATCAAGAGCCATTTAAAAATTTGAAAACAGGAAAGTATGCAAAAATGAAGGGTGCCCACAC
TAATGATGTAAAACAGTTAACAGAGGCAGTGCAAAAAATAGCCACAGAAAGCATAGTAATATGGGGAAAAACT
CCTAAATTTAGATTACCCATACAAAAAGAAACATGGGAAGCATGGTGGACAGAGTATTGGCAAGCCACCTGGA
TTCCTGAGTGGGAATTTGTCAATACCCCTCCCTTAGTAAAATTATGGTACCAGCTAGAGAAAGAACCCATAAT
AGGAGCAGAAACTTTCTATGTAGATGGGGCAGCTAATAGGGAAACTAAATTAGGAAAAGCAGGATATGTTACT
GACAGAGGAAGACAAAAGGTTGTCTCCCTAACTGACACAACAAATCAGAAGACTGAGTTACAAGCGATCCATC
TAGCTTTGCAGAATTCGGGGCTAGAAGTAAACATAGTAACAGACTCACAATATGCATTAGGAATCATTCAAGC
ACAACCAGATAAGAGTGAATCAGAGTTAGTCAGTCAGATAATAGAGCAGTTAATAAAAAAGGAAAAGGTCTAT
TTAGCATGGGTACCAGCACACAAGGGAATTGGAGGAAATGAACAAGTAGATAAATTAGTCAGTGCTGGAATCA
GGAAAGTACTATTTTTGGATGGAATAGATAAGGCCCAAGAAGAACATGAGAAATATCACAATAATTGGAGAGC
AATGGCTAGTGATTTTAACCTGCCACCTGTAGTAGCAAAAGAAATAGTAGCCAGCTGTGATAAATGTCAGTTA
AAAGGAGAAGCCATGCATGGACAAGTAGACTGTAGTCCAGGAATATGGCAATTAGATTGCACACATTTAGAAG
GAAAAATTATCCTGGTAGCAGTCCATGTAGCCAGTGGATATATAGAAGCAGAAGTTATCCCAGCAGAGACAGG
GCAGGAAACAGCATACTTTATCTTAAAATTAGCAGGAAGATGGCCAGTAAAAACAATACATACAGACAATGGC
AGCAATTTTATCAGTAATACAGTTAAGGCTGCCTGTTGGTGGGCGGGGATCAAGCAAGAATTTGGCATTCCCT
ACAATCCCCAAAGTCAAGGAGTAGTAGAATCTATGAATAAAGAATTAAAGAAGATTATAGGACAGGTAAGAGA
TCAGGCTGAACATCTTAAAACAGCAGTACAAATGGCAGTATTCATCCACAATTTTAAAAGAAAAGGGGGGATT
GGGGGATACAGTGCAGGGGAAAGAATAGTAGACATAATAGCAACAGACATACAAACTAAAGAACTACAAAAAC
AAATTACAAAAATTCAAAATTTTCGGGTTTATTACAGGGACAACAGAGATCCACTTTGGAAAGGACCAGCAAA
GCTTCTTTGGAAAGGTGAAGGGGCAGTAGTAATACAAGAGGATAGTGAAATAAAAGTAGTGCCAAGAAGAAAA
GCAAAGATCATTAGGGATTATGGAAAACAGATGGCAGGTGATGATTGTGTGGCAAGTAGACAGGATGAAGATT
AGAACATGGAATAGTTTAGTAAAACACCATATGTATATTTCAAGGAAAGCTCAGGGATGGGTTTATAGGCATC
ACTATGAAAGCACTAATCCAAGAGTAAGCTCAGAAGTACACATCCCACTAGGGGATGATAAATTGGTGATAAC
AACATATTGGGGTCTGCATGCAGGAGAAAGAGAGTGGCATTTGGGTCATGGAGCCTCCATAGAATGGAGGAAA
AGAAGATATAGCACACAAGTAGACCCTGACCTAGCAGACCAACTAATTCATCTGCATTACTTTGATTGTTTTT
CAGAATCTGCTATAAGACATGCCATATTAGGACGTATAGTTAGTCCTAGGTGTGAATATCAAGCAGGACATAA
CAAGGTAGGATCTCTACAGTACTTGGCACTAGCAGCATTAATAAAACCAAAAAAGAGAAAGCCACCTTTGCCT
AGTGTTAGGAAACTGACAGAGGATAGATGGAACAAGCCCCAGAAGACCATGGGCCACAGAGGGAGCCATACAA
TGAATGGACACTAGAACTTTTAGAGGAACTTAAGAATGAAGCTGTTAGACATTTTCCTAGGCCATGGCTCCAT
AGCTTAGGACAATATATCTATGAAACTTATGGGGATACTTGGACAGGAGTGGAAGCCATAATAAGAATTCTGC
AACAACTGCTGTTTATTCATTTCAGAATTGGATGTCAACATAGCAGAATAGATATTCTACCAAGGAGAGCAAG
GAATGGAGCCAGTAGATCCTAGTTTAGAGCCCTGGAAGCATCCAGGAAGCCGGCCTAAAACTGCTTGTACCAA
TTGTTATTGTAAAAAGTGTTGCTTTCATTGCCAAGTGTGTTTCACAACCAAAGCCTTAGGCATCTCCTATGGC
AGGAAGAAGCGGAGACAGCGACGAAGAGCTCCTCCAAGCAGTCAGAATCATCAAGTTTCTCTACCAAAGCAGT
GAGTAATATATGTAATGCAATCCTTAGAAATATATGCAATAGTAGCATTAGTAGTAGTAGCAATAATAGCAAT
AGTTGTGTGGACCATAGTGCTCCTAGAATATAGGAAAATATTAAGACAAAGGAAAATAGACAGATTAATTGAT
AGAATAAGAGAAAGAGCAGAAGACAGTGGCAATGAAAGTGAAGGGGATGAGGAGGAATTATCAGCCCTTGTGG

AAATGGGGCATCATGCTCCTTGGGATGTTGATGATCTGTAGTGCTACAGAAAAATTGTGGGTCACAGTCTATT
ATGGGGTACCTGTGTGGAAAGAAGCAACCACCACTTTATTTTGTGCATCAGATGCTAAAGCATATCATACAGA
GATGCATAATGTTTGGGCCACACATGCCTGTGTACCCACAGACCCTAGCCCACAAGAAGTAGTATTGGGAAAT
GTGACAGAAAATTTTAACATGTGGAAAAATAACATGGTAGAACAGATGCATGAAGATATAATCAGTTTGTGGG
ATCAAAGCCTAAAGCCATGTGTAAAATTAACCCCACTCTGTGTCACTTTAAATTGCACTGATGTGAGAAATAA
TACTAATACCACTATTAATAATTGGGAAAAGGTGGACAAAGGGGAAATAAAAAACTGCTCTTTCAATGTCACC
ACAAGCATAAGAGATAAGATGCAACAAGCATATTCACTTTTTTATAAACTTGATGTAGTGCAAATAGAGAATA
CAAGTAGCTATACATTGATAAATTGTAACACCTCAGTCATTACACAGGCCTGTCCAAAGGTATCCTTTGAACC
AATTCCCATACATTATTGTACCCCGGCTGGTTTTGCAATTCTAAAGTGTAATGATAATAAGTTCAATGGAACA
GGACCATGTACTAATGTTAGCACAGTACAATGTACACATGGAATTAAGCCAGTAGTGTCAACTCAACTGCTGT
TGAATGGCAGTCTGGCAGAAGGAGGAGAGGTAGTAATTAGATCTGAAAATTTCACAAACAATGCTAAAACCAT
AATAGTACAGCTGAATACATCTGTAGAAATTAATTGTATAAGACCCAACAACAATACAAGAAAAAGTATAACT
ATAGGACCAGGGAGAGCATTTTATACAACAGACATAATAGGAGATATAAGACAAGCACATTGTAACCTTAGTA
GAGCAAAATGGAATGACACTTTAAAACAGATAGTTACAAAATTAAAAGAACAATTTGAGAACAAGACAATAGT
CTTTAATCAATCTTCAGGAGGGGACCCAGAAATTGTAATGCACAGCTTTAATTGTGGAGGGGAATTTTTCTAC
TGTAATACAACACAGCTGTTTAACAGTACTTGGAATGGTACTGACTGGAATGACACTACAGGGTTAGAGAACA
TCACACTCCCATGCAGAATAAAACAAATTGTAAACAGGTGGCAGGAAGTAGGAAAAGCAATGTATGCCCCTCC
CATCAAAGGACAAATTAGATGTTCATCAAATATTACAGGGCTACTATTAACAAGAGATGGGGGAAACAGTAGT
GAGATGACCGAGATCTTCAGACCTGGAGGAGGAGATATGAGGGACAATTGGAGAAGTGAATTATATAAATATA
AAGTAGTAAAAATTGAGCCATTAGGAGTAGCACCCACCAAGGCAAAGAGAAGAGTGGTGCAGAGAGAAAAAAG
AGCAGTGGGAACAATAGGAGCTATGTTCCTTGGGTTCTTGGGAGCAGCAGGAAGCACTATGGGCGCAGCGTCA
ATGACGCTGACGGTACAGGCCAGACTATTATTGTCTGGTATAGTGCAACAGCAGAACAATTTGCTGAGAGCTA
TTGAGGCGCAACAGCATCTGTTGCAACTCACAGTCTGGGGCATCAAGCAGCTCCAGGCTAGAGTCCTGGCTGT
GGAAAGATACCTAAGGGATCAACAGCTCCTGGGGATTTGGGGTTGCTCTGGAAAACTCATTTGCACCACTAAT
GTGCCTTGGAATGCTAGTTGGAGTAATAAATCTCTGGAAAAGATTTGGAATAACATGACCTGGATGGAGTGGG
ACAGAGAAATTGACAATTACACAAAATTGATATACACCTTAATTGAAGAATCGCAAAACCAGCAGGAAAAGAA
TGAACACGACTTATTGGAGTTGGATAAGTGGGACAGTTTGTGGAATTGGTTTGACATAACAAAATGGCTGTGG
TATATAAAAATATTCATAATGATAGTAGCAGGTTTAGTAGGTTTAAGAATAGTTTTTACTGTGCTTTCTATAG
TGAATAGAGTTAGGCAGGGATATTCACCACTATCATTTCAGACCCACCACCCAGTCCCGCGGGGACCCGACAG
GCCCGAAGGAATCGAAGAAGGAGGTGGAGAGAGAGACAGAGACACATCCGGACCATTAGTGAACGGATTCTTA
GCGATTATCTGGGTCGACCTGCGGAGCCTGTGCCTCTTCAGCTACCACCGCTTGAGAGACTTACTCTTGATTG
TAGCGAGGATTGTGGAACTTCTGGGACGCAGGGGGTGGGAGGCCCTGAAATATTGGTGGAATCTCCTGCAGTA
TTGGAGTCAGGAACTAAAGAATAGTGCTGTTAGTCTGCTTAATGCCACAGCTATAGCAGTAGCTGAGGGGACA
GATAGGATTATAGAAGTATTACAAAGAGCTTGTAGAGCTATTCTCCACATACCTAGAAGAATAAGACAGGGCT
TAGAAAGGGCTTTGCTATAAGATGGGTGGTAAGTGGTCAAAACGTCGTGCGGGTGGATGGGAAGCTGTAAGGG
AAAAAATAAGACAAACTGAGCCTGAGCCAGCAGCAGAGGGGGTGGGAGCAGCATCTCGAGACCTGGAAAAATA
TGGAGCAATCACCAGTAGCAATACAGCACATACCAACGCTGATTGTGCCTGGGTAGAAGCACAAGAGGAGGAT
GAAGAGGTGGGTTTCCCAGTCAGACCTCAGGTACCTTTAAGACCTATGACTTTCAAGGGAGCGCTAGATCTTA
GCCACTTTTTAAAAGAAAAGGGGGGACTGGAAGGGTTAATTCACTCCCAGAAAAGACAAGACATCCTTGATTT
GTGGGTCTACAACACACAAGGCTATTTCCCTGATTGGCAGAACTACACACCAGGGCCAGGGGAGAGATTTCCC
CTGACCTTTGGATGGTGCTTCAAGCTAGTACCAGTTGATCCAGATCAGGTAGAAGAGGCTAATAAGGAGAGA
CCAACAGCTTGTTACACCCTATGAGCCAGCATGGGATAGAGGACCCGGAGAAAGAAGTATTAATGTGGAAGTT
TGACAGCCGCCTAGCATTTCATCACGTGGCCAGAGAGAAGCATCCGGAGTACTTCAAGAACTGATGACATCGG
GTTTTTCTACAAGGGACTTTCCGCTGGGGACTTTCCAGGGGAGGTGTGGCCTGGGCGGGACAGGGGAGTGGCG
AGCCCTCAGATGCTGCATATAAGCAGCTGCTTTCTGCCTGTACTGGGTCTCTCTGGTTAGACCAGATTTGAGC
CTGGGAGCTCTCTGGCTAACTAGGGAACCCACTGCTTAAGCCTCAATAAAGCTTGCCT
>AE-D7 HIV-1 genome, derived from RNA genomic sequence
GGGGGAATTGGAGGTTTTATCAAAGTAAGACAGTATGATCAAATACCCATAGAAATCTGCGGACACAAAGCTG
TAGGTACAGTATTAGTAGGACCGACACCTGTCAACATAATTGGAAGAAATCTGTTGACTCAGATTGGCTGCAC
TTTAAATTTTCCTATTAGTCCTATTGAAACTGTACCAGTAAAATTAAAGCCAGGAATGGATGGGCCAAAGGTT
AAACAATGGCCATTGACAGAAGAAAAAATAAAAGCATTAGTAGAAATTTGTACAGAAATGGAAAAGGAAGGAA
AAATTTCAAAAATTGGGCCTGAAAATCCATACAATACTCCAGTATTTGCCATAAAGAAAAAAGACAGTACTAA
ATGGAGAAAATTAGTAGACTTTAGAGAACTTAATAAGAGAACTCAAGACTTCTGGGAAGTTCAATTAGGAATA
CCACATCCTGCAGGGTTAAAAAAGAAAAAATCAGTAACAGTACTGGATGTGGGTGATGCATACTTTTCAGTTC

CATTAGATAAAGAATTCAGGAAGTATACTGCATTTACCATACCTAGTATAAACAATGAGACACCAGGGATTAG
ATATCAGTACAATGTGCTTCCACAGGGATGGAAAGGATCCCCAGCCATATTCCAAAGTAGTATGACAAAAATC
TTAGAGCCTTTTAGAAAACAAAACCCAGAAATGGTTATCTATCAATACATGGATGATTTATATGTAGGATCTG
ACTTAGAAATAGGGCAGCATAGAGCAAAAATAGAGGAACTGAGACAGCATCTGTTGAGGTGGGGATTTACCAC
ACCAGACAAAAAGCATCAGAAAGAACCTCCATTCCTTTGGATGGGTTATGAGCTCCATCCTGATAAATGGACA
GTACAGCCTATAAAGCTGCCAGAAAAAGACAGCTGGACTGTCAATGACATACAGAAGTTAGTGGGAAAATTGA
ATTGGGCAAGTCAGATCTATCCAGGGATTAAAGTAAGGCAATTATGTAAACTCATTAGGGGAACCAAAGCACT
AACAGAAGTAGTACCACTAACAGCAGAAGCAGAGCTAGAATTGGCAGAAAACAGGGAGATACTAAAAGAACCA
GTACATGGAGTGTATTATGACCCATCAAAAGACTTAATAGCAGAAATACAGAAGCAGGGGCAAGGCCAGTGGA
CATATCAAATTTATCAAGAGCCATTTAAAAATTTGAAAACAGGAAAGTATGCAAGAATGAAGGGTGCCCACAC
TAATGATGTAAAACAGTTAACAGAGGCAGTGCAAAAAATAGCCACAGAAAGCATAGTAATATGGGGAAAAACT
CCTAAATTTAGATTACCCATACAAAAAGAAACATGGGAAGCATGGTGGACAGAGTATTGGCAAGCCACCTGGA
TTCCTGAGTGGGAATTTGTCAATACCCCTCCCTTAGTAAAATTATGGTACCAGCTAGAGAAAGAACCCATAAT
AGGAGCAGAAACTTTCTATGTAGATGGGGCAGCTAATAGGGAAACTAAATTAGGAAAAGCAGGATATGTTACT
GACAGAGGAAGACAAAAGGTTGTCTCCCTAACTGACACAACAAATCAGAAGACTGAGTTACAAGCGATCCATC
TAGCTTTGCAGGATTCGGGGCTAGAAGTAAACATAGTAACAGACTCACAATATGCATTAGGAATCATTCAAGC
ACAACCAGATAAGAGTGAATCAGAGTTAGTCAGTCAGATAATAGAGCAGTTAATAAAAAAGGAAAAGGTCTAT
TTAGCATGGGTACCAGCACACAAGGGAATTGGAGGAAATGAACAAGTAGATAAATTAGTCAGTGCTGGAATCA
GGAAAGTACTATTTTTGGATGGAATAGATAAGGCCCAAGAAGAACATGAGAAATATCACAATAATTGGAGAGC
AATGGCTAGTGATTTTAACCTGCCACCTGTAGTAGCAAAAGAAATAGTAGCCAGCTGTGATAAATGTCAGTTA
AAAGGAGAAGCCATGCATGGACAAGTAGACTGTAGTCCAGGAATATGGCAATTAGATTGCACACATTTAGAAG
GAAAAATTATCCTGGTAGCAGTCCATGTAGCCAGTGGATATATAGAAGCAGAAGTTATCCCAGCAGAGACAGG
GCAGGAAACAGCATACTTTATCTTAAAATTAGCAGGAAGATGGCCAGTAAAAACAATACATACAGACAATGGC
AGCAATTTTATCAGTAATACAGTTAAGGCTGCCTGTTGGTGGGCGGGGATCAAGCAAGAATTTGGCATTCCCT
ACAATCCCCAAAGTCAAGGAGTAGTAGAATCTATGAATAAAGAATTAAAGAAGATTATAGGACAGGTAAGAGA
TCAGGCTGAACATCTTAAAACAGCAGTACAAATGGCAGTATTCATCCACAATTTTAAAAGAAAAGGGGGGATT
GGGGGATACAGTGCAGGGGAAAGAATAGTAGACATAATAGCAACAGACATACAAACTAAAGAACTACAAAAAC
AAATTACAAAAATTCAAAATTTTCGGGTTTATTACAGGGACAACAGAGATCCACTTTGGAAAGGACCAGCAAA
GCTTCTTTGGAAAGGTGAAGGGGCAGTAGTAATACAAGAGGATAGTGAAATAAAAGTAGTGCCAAGAAGAAAA
GCAAAGATCATTAGGGATTATGGAAAACAGATGGCAGGTGATGATTGTGTGGCAAGTAGACAGGATGAAGATT
AGAACATGGAATAGTTTAGTAAAACACCATATGTATATTTCAAGGAAAGCTCAGGGATGGGTTTATAGGCATC
ACTATGAAAGCACTAATCCAAGAGTAAGCTCAGAAGTACACATCCCACTAGGGGATGATAAATTGGTGATAAC
AACATATTGGGGTCTGCATGCAGGAGAAAGAGAGTGGCATTTGGGTCATGGAGCCTCCATAGAATGGAGGAAA
AGAAGATATAGCACACAAGTAGACCCTGACCTAGCAGACCAACTAATTCATCTGCATTACTTTGATTGTTTTT
CAGAATCTGCTATAAGACATGCCATATTAGGACGTATAGTTAGTCCTAGGTGTGAATATCAAGCAGGACATAA
CAAGGTAGGATCTCTACAGTACTTGGCACTAGCAGCATTAATAAAACCAAAAAAGAGAAAGCCACCTTTGCCT
AGTGTTAGGAAACTGACAGAGGATAGATGGAACAAGCCCCAGAAGACCATGGGCCACAGAGGGAGCCATACAA
TGAATGGACACTAGAACTTTTAGAGGAACTTAAGAATGAAGCTGTTAGACATTTTCCTAGGCCATGGCTCCAT
AGCTTAGGACAATATATCTATGAAACTTATGGGGATACTTGGACAGGAGTGGAAGCCATAATAAGAATTCTGC
AACAACTGCTGTTTATTCATTTCAGAATTGGATGTCAACATAGCAGAATAGGTATTCTACCAAGGAGAGCAAG
GAATGGAGCCAGTAGATCCTAGTTTAGAGCCCTGGAAGCATCCAGGAAGCCGGCCTAAAACTGCTTGTACCAA
TTGTTATTGTAAAAAGTGTTGCTTTCATTGCCAAGTGTGTTTCACAACCAAAGCCTTAGGCATCTCCTATGGC
AGGAAGAAGCGGAGACAGCGACGAAGAGCTCCTCCAAGCAGTCAGAATCATCAAGTTTCTCTACCAAAGCAGT
GAGTAATATATGTAATGCAATCCTTAGAAATATATGCAATAGTAGCATTAGTAGTAGTAGCAATAATAGCAAT
AGTTGTGTGGACCATAGTGCTCCTAGAATATAGGAAAATATTAAGACAAAGGAAAATAGACAGATTAATTGAT
AGAATAAGAGAAAGAGCAGAAGACAGTGGCAATGAAAGTGAAGGGGATGAGGAGGAATTATCAGCCCTTGTGG
AAATGGGGCATCATGCTCCTTGGGATGTTGATGATCTGTAGTGCTACAGAAAAATTGTGGGTCACAGTCTATT
ATGGGGTACCTGTGTGGAAAGAAGCAACCACCACTTTATTTTGTGCATCAGATGCTAAAGCATATCATACAGA
GATGCATAATGTTTGGGCCACACATGCCTGTGTACCCACAGACCCTAGCCCACAAGAAGTAGTATTGGGAAAT
GTGACAGAAAATTTTAACATGTGGAAAAATAACATGGTAGAACAGATGCATGAAGATATAATCAGTTTGTGGG
ATCAAAGCCTAAAGCCATGTGTAAAATTAACCCCACTCTGTGTCACTTTAAATTGCACTGATGTGAGAAATAA
TACTAATACCACTATTAATAATTGGGAAAAGGTGGACAAAGGGGAAATAAAAAACTGCTCTTTCAATGTCACC
ACAAGCATAAGAGATAAGATGCAACAAGCATATTCACTTTTTTATAAACTTGATGTAGTGCAAATAGAGAATA
CAAGTAGCTATACATTGATAAATTGTAACACCTCAGTCATTACACAGGCCTGTCCAAAGGTATCCTTTGAACC

AATTCCCATACATTATTGTACCCCGGCTGGTTTTGCAATTCTAAAGTGTAATGATAATAAGTTCAATGGAACA
GGACCATGTACTAATGTTAGCACAGTACAATGTACACATGGAATTAAGCCAGTAGTGTCAACTCAACTGCTGT
TGAATGGCAGTCTGGCAGAAGGAGGAGAGGTAGTAATTAGATCTGAAAATTTCACAAACAATGCTAAAACCAT
AATAGTACAGCTGAATACATCTGTAGAAATTAATTGTATAAGACCCAACAACAATACAAGAAAAAGTATAACT
ATAGGACCAGGGAGAGCATTTTATACAACAGACATAATAGGAGATATAAGACAAGCACATTGTAACCTTAGTA
GAGCAAAATGGAATGACACTTTAAAACAGATAGTTACAAAATTAAAAGAACAATTTGAGAACAAGACAATAGT
CTTTAATCAATCTTCAGGAGGGGACCCAGAAATTGTAATGCACAGCTTTAATTGTGGAGGGGAATTTTTCTAC
TGTAATACAACACAGCTGTTTAACAGTACTTGGAATGGTACTGACTGGAATGACACTACAGGGTTAGAGAACA
TCACACTCCCATGCAGAATAAAACAAATTGTAAACAGGTGGCAGGAAGTAGGAAAAGCAATGTATGCCCCTCC
CATCAAAGGACAAATTAGATGTTCATCAAATATTACAGGGCTACTATTAACAAGAGATGGGGGAAACAGTAGT
GAGATGACCGAGATCTTCAGACCTGGAGGAGGAGATATGAGGGACAATTGGAGAAGTGAATTATATAAATATA
AAGTAGTAAAAATTGAGCCATTAGGAGTAGCACCCACCAAGGCAAAGAGAAGAGTGGTGCAGAGAGAAAAAAG
AGCAGTGGGAACAATAGGAGCTATGTTCCTTGGGTTCTTGGGAGCAGCAGGAAGCACTATGGGCGCAGCGTCA
ATGACGCTGACGGTACAGGCCAGACTATTATTGTCTGGTATAGTGCAACAGCAGAACAATTTGCTGAGAGCTA
TTGAGGCGCAACAGCATCTGTTGCAACTCACAGTCTGGGGCATCAAGCAGCTCCAGGCTAGAGTCCTGGCTGT
GGAAAGATACCTAAGGGATCAACAGCTCCTGGGGATTTGGGGTTGCTCTGGAAAACTCATTTGCACCACTAAT
GTGCCTTGGAATGCTAGTTGGAGTAATAAATCTCTGGAAAAGATTTGGAATAACATGACCTGGATGGAGTGGG
ACAGAGAAATTGACAATTACACAAAATTGATATACACCTTAATTGAAGAATCGCAAAACCAGCAGGAAAAGAA
TGAACACGACTTATTGGAGTTGGATAAGTGGGACAGTTTGTGGAATTGGTTTGACATAACAAAATGGCTGTGG
TATATAAAAATATTCATAATGATAGTAGCAGGTTTAGTAGGTTTAAGAATAGTTTTTACTGTGCTTTCTATAG
TGAATAGAGTTAGGCAGGGATATTCACCACTATCATTTCAGACCCACCACCCAGTCCCGCGGGGACCCGACAG
GCCCGAAGGAATCGAAGAAGGAGGTGGAGAGAGAGACAGAGACACATCCGGACCATTAGTGAACGGATTCTTA
GCGATTATCTGGGTCGACCTGCGGAGCCTGTGCCTCTTCAGCTACCACCGCTTGAGAGACTTACTCTTGATTG
TAGCGAGGATTGTGGAACTTCTGGGACGCAGGGGGTGGGAGGCCCTGAAATATTGGTGGAATCTCCTGCAGTA
TTGGAGTCAGGAACTAAAGAATAGTGCTGTTAGTCTGCTTAATGCCACAGCTATAGCAGTAGCTGAGGGGACA
GATAGGATTATAGAAGTATTACAAAGAGCTTGTAGAGCTATTCTCCACATACCTAGAAGAATAAGACAGGGCT
TAGAAAGGGCTTTGCTATAAGATGGGTGGTAAGTGGTCAAAACGTCGTGCGGGTGGATGGGAAGCTGTAAGGG
AAAAAATAAGACAAACTGAGCCTGAGCCAGCAGCAGAGGGGGTGGGAGCAGCATCTCGAGACCTGGAAAAATA
TGGAGCAATCACCAGTAGCAATACAGCACATACCAACGCTGATTGTGCCTGGGTAGAAGCACAAGAGGAGGAT
GAAGAGGTGGGTTTCCCAGTCAGACCTCAGGTACCTTTAAGACCTATGACTTTCAAGGGAGCGCTAGATCTTA
GCCACTTTTTAAAAGAAAAGGGGGGACTGGAAGGGTTAATTCACTCCCAGAAAAGACAAGACATCCTTGATTT
GTGGGTCTACAACACACAAGGCTATTTCCCTGATTGGCAGAACTACACACCAGGGCCAGGGGAGAGATTTCCC
CTGACCTTTGGATGGTGCTTCAAGCTAGTACCAGTTGATCCAGATCAGGTAGAAGAGGCTAATAAAGGAGAGA
CCAACAGCTTGTTACACCCTATGAGCCAGCATGGGATAGAGGACCCGGAGAAAGAAGTATTAATGTGGAAGTT
TGACAGCCGCCTAGCATTTCATCACGTGGCCAGAGAGAAGCATCCGGAGTACTTCAAGAACTGATGACATCGG
GTTTTTCTACAAGGGACTTTCCGCTGGGGACTTTCCAGGGGAGGTGTGGCCTGGGCGGGACAGGGGAGTGGCG
AGCCCTCAGATGCTGCATATAAGCAGCTGCTTTCTGCCTGTACTGGGTCTCTCTGGTTAGACCAGATTTGAGC
CTGGGAGCTCTCTGGCTAACTAGGGAACCCACTGCTTAAGCCTCAATAAAGCTTGCCT

>K-A1 HIV-1 genome, derived from RNA genomic sequence
CCTATGAGCCTGCATGGGATAGAGGACCCGGAGAAAGAAGTGCTGATGTGGAAGTTTGACAGCCGCCTAGCAT
TCCATCACATGGCCCGAGAGCTGCATCCGGAGTACTACAAGAACTGATGACACCGAGTTTCTACAAGGGACTT
TCCGCTGGGGACTTTCCAGGGGAGGCGCGGCCTGGGCGGGACTGGGGAGTGGCGAGCCCTCAGATGCTGCATA
TAAGCAGCTGCTTTTTGCCTGTACTGGGTCTCTCTGGTTAGACCAGATCTGAGCCTGGGAGCTCTCTGGCTAA
CTAGGGAACCCACTGCTTAAGCCTCAATAAAGCTTGCCTTGAGTGCTTTAAGTAGTGTGTGCCCGTCTGTTGT
GTGACTCTGGTAACTAGAGATCCCTCAGACCCTTTAGTCAGTGTGGAAAATCTCTAGCAGTGGCGCCCGAACA
GGGACGCGAAAGCGAAAGTAAGACCGGAGGAGCTCTCTCGACGCAGGACTCGGCTTGCTGAAGTGCGCACGGC
AAGAGGCGAGGGGCGGCGACTGGTGAGTACGCCAAAATTTTTGACTAGCGGAGGCTAGAAGGAGAGAGATGG
GTGCGAGAGCGTCAGTATTAAGCGGGGGAGAATTAGATAAATGGGAAAAAATTCGGTTAAGGCCAGGGGGAAA
GAAAAGGTATAAGCTAAAACATATAGTATGGGCAAGCAGGGAGCTAGAACGATTCGCAGTCAACCCTGGCCTG
TTAGAAACATCAGAAGGCKGTAGACAAATACTGGACAGCTMCAAC????????????????????????????
??????????ATTTAATACAGTAGCAGTCCTCTATTGTGTACACCAAAGGATAGAGGTAAAGGATACCAAAGA
AGCTTTAGAGAAGGTAGAGGAAGAGCAAAACAAAAGTAAGAAAAAAGCACAGCAAGCAGCAGCAGCTGCAGCT
GACAC??
???
???
???
???
???
???
???
??TTGTAAGACTATCTTAAAAGCATTAGGACCA
GCAGCCACACTAGAAGAAATGATGAC???
???????????????????????AATTCAGCTACCGTAATGATGCAGAAAGGCAACTTTAGGAACCAAAGAAA
GGCTGTCAAGTGTTTCAATTGTGGCAAAGAAGGGCACATAGCCAGAAATTGCAGGGCCCCTAGGAAAAAGGGC
TGTTGGAAATGTGGAAAGGAAGGACAC??
???
???
???TCACAATAAAAGTAGG
AGGGCAGCTAAAGGAAGCTCTATTAGATACAGGAGCAGATGATACAGTATTAGAAGAAATCAATTTGCCAGGA
AGATGGAAACCAAAAATGATAGGGGGAATTGGAGGTTTTATCAAAGTAAGACAGTATGATC????????????
??????????GCATAAAGCTATAGGTACAGTATTAGTAGGACCTACACCTGTCAACATAATTGGAAGAAATCT
GTTGACTCAGCTTGGTTGCACTTTAAATTTTCCCATTAGTCCTATTGAAACTGTACCAGTAAAATTAAAGCCA
GGAATGGATGGCCCAAAAGTTAAACAGTGGCCATTGACAGAGGAAAAAATAAAAGCATTAGTAGAAATTTGTA
CAGAAATGGAAAAAGAAGGAAAAATTTCAAAAGTTAGGCCTGAAAATCCATACAATACTCCAGTATTTGCCAT
AAAGAAAAAAGATGGTACTAAATG?AGAAAACTAGTAG???????????????????????????????????
????????????????GAATACCACATCCTGCAGGTTTAAAAAAGAAAAATTCAGTAACAGTACTAGATGTAG
GTGATGCTTATTTTTCAGTTCCCTTAGATAAAGACTTCAGGAAGTATACTGCATTTACCATACCCAGTATAAA
TAATGAGACACCAGGGATTAGATATCAGTACAATGTACTTC????????????????????????????????
???
????????????????????????????????GGGCAGCACAGAATAAAAATAGAGGAACTAAGACAACATCT
GTTAAAGTGGAAATTCACCACACCAAACAAAAAACATCAAAAAAAACCTCCATTCCTTTGGATGGGTTATGAA
C??
??ATTATGTAAACT
CCTTAGGGGAGCCAAAGCACTAACAAAAGTAATAACACTAACAAAAAAAGCAGAACTA???????????????
??AAGTACAGA
AGCAGGGGCAAGGTCAATGGACATATCAAATTTATCAAGAGCCATTTAAAAATCTGAAAACAGGAAAGTATGC
AAGAATGAGGAGTGCCCACACTAATGATGTAAAACAGTTAACAGAGGCAGTGCAAAAAATATCCACAGAAAGC
ATAATAATCTGGGGAAAGACCCCTAAATTTAGACTACCTATACAAAAGGAAACATGGGAAACATGGTGGGCGG
AGTATTGGTTTGTCAATACCCCTCCCTTAGTGAAACTATGGTACCAGTTAGAAAAAGAACCCATAGCAGGAGC
AGAAACTTTCTATGTAGATGGGGCAGCCAATAGAGAAACTAAATTAGGAAAAGCAGGATATGTTACTGACAGA
GGAAGACAAAAAGTTGTCTCCCTAACTGACACAACAAATCAGAAAACTGAGTTGCAAGCTATTGATCTAGCTT
TGCAGGATTCAGGGTTAGAAGTAAATATAGTAAC???????????????????????????????????????

???
???
???TAATTGGAGAGCAATGGC
TAGTGATTTTAACCTACCACCTGTAGTAGCAAAAGAAATAGTAGCCTGTTGTGATAAATGTCAGCTAAAAGGA
GAAGCCATGCATGGACAAGTAGACTGTAGTCCAGGAATATGGCAACTAGATTGTACACATCTAGAAGGAAAA
TTATCATAGTAGCAGTTCATGTAGCCAGTGGATATATAGAGGCAGAAGTTATTCCAGCAGAGACAGGGCAGGA
AACAGCATACTTTCTCTTAAAATTAGCAGGGAGATGGCCAGTAAAAACAATACATACAGATAATGGCCCCAAT
TTCATTAGCTCCGCGGTYAAGGCCGCCTGTTGGTGGGCRGGGATCAAGCAGGAATTTGGCATTCCCTACAATC
CCCAAAGTCAAGGAGTAGTAGAG??
??????TCTTAAGACAGCACTACAAATGGCAGTATTCATCCACAATTTTAAGAAAAAAGGGGGGATTGGGGG
TACAGTGCAGGGGAAAGAATAATAGACATAATAGCATCAGACATACAGACTAAAGAAC???????????????
???
??TATA?AAATAGTACCAAGAAGAAAAGCAAAG
ATCATTAGAGATTATGGAAAACAGATGGCAGGTGATGATTGTGTGGCAGGTGGACAGGATGAGGATTAGAACA
TGGAAAAGTTTAGTAAAACACCACATGTATATTTCAAAGAAAGCTAAGGGATGGATTTATAAACATCACTATG
AAAGCACTCATCCAAGAATAAGTTCAGAAGTACACATCCCATTAGGGGATGCTAAATTGGTAATAATAACATA
TTGGGGTCTGCATACAGGAGAAAGAGACTGGCATTTGGGCCAGGGAGCCTCCATAGAATGGAGGATAAAGAGA
TATAGAACACAAGTAGACCCTGGCCTAGCAGACCAACTAATTCATATGCACTATTTTGATTGTTTTTCAGAAT
CTGCTATAAGAAATGCCATATTAGGACGTATAGTTAGTCCTAGGTGTGAATATCAAGCAGGACATAACAAGGT
AGGATCTCTACAATATTT???GCCTAGTGTA
GCGAAACTGACAGAGGATAGATGGAACAAGCCCCAGAGGACCAAGGGCCACAGAGGGAGCCATACAATGAATG
GGC??
?????????????????????TATGGAAATACTTGGACAGGAGTGGAAGCCCTAATAAGAACTCTGCAACAAC
TGCTGTTTATTCATTTCAGAATTGGGTGTCGGCATAGCAGAATAGGCATTGACATCCGACAGAGGAGAGCAAG
AAATGGATCCAGTAGATCCTAGACTAGAGCCCTGGAAGCATCCAGGAAGTCAGCCTAGGACTGCTTGTAATAG
TTGCTATTGTAAAAAGTGTTGCCTTCATTGCCAAGTTTGCTTCTTAAGAAAAGGCTTAGGCATCTCCTATGGC
AGGAAGAAGCGGAGACAGCGACGAAGAACTCCTTCAGAAAGTCAGACTCATCCGGCTTCTCTACCAAAGCAGT
AAGTTCTTTATCAAAGCAGTAAGTAGTATATGTAATGCAACCTGTACAAATATTAGCAATAGTAGCATTAGTA
GTAGTAGCAATAATAGCAATAGTTGTATGGACCATAGTAGCTATAGAATATAGA???????????????????
???
????????????????CTTGTGGAGATGGGGCAWCATGCTCCTTGGGATATTGATGATCTGTAGTGCTGGAGA
ACAATTGTGGGTCACAGTCTATTAT????????????????????AGCAACTACCACTCTATTTTGTGCCTCA
GATGCTAAAGCATATGATACAGAAAGTCATAATGTTTGGGCCACACATGCCTGTGTACCCACAGACCCCAACC
CACAAGAAGTAAGATTGAAAAATGTGACAGAAGAATTTAACATGGGAAAAAATAATATGGTAGACCAAATGCA
TGAGGATATAATCAGCTTATGGGATCAAAGCTTAAAGCCATGTGTAAAATTAACCCCACTTTGTGTTACTTTA
CATTGCACTGAGCCGACGAAGAGGAATAATACTAACAGCACTAATGCTCAGAACAACATTATTATAGTGAATA
ACACAGACATAAAAAACTGTTCTTTCAATGTCACCACAGACATAAGAGACAGAATGCAGAAAGAATTTGCACT
TTTTCACAAGCTTGATCTAGTACCAATAGATGATGATAATATTACTAATAAGAATAACTATAGTAACTATAGG
TTGATAAGTTGCAACACCTCAGTCATTAGACAGGCCTGTCCAAAGGTATCTTTTGAGCCAATTCCCATACATT
ATTGTGCCCCTGCTGGTTTTGCGATTCTAAAGTGTAACGAGGAAAAGTTCAATGGAACAGGACTATGCAGCGA
TGTCAGCACAGTACAATGTACACATGGAATTAAACCAGTAGTGTCAACTCAACTGCTGTTAAATGGCAGCCTA
GCAAAAGAAGAGGTAGTACTTAGGTCTGAAAATTTCACAGAAAATACTAAAACCATAATAGTGCAGCTGAAGG
ACCCTGTAGTAATTAATTGTACAAGACCCAACAACAATATAAGAAAAAGGATACGTATNNNNNAGGACCAGGG
GGCGCATCATTTTATGCAACAAACATA?ATGGGAGATATAAGAAAAGCATATTGTATCATTAATAGCACACAA
TGGAATAACACTTTACAACAGGTAGTTAAAAAATTAAGAGAACAATTTGGGAATAAAACAATAGTCTTTAATC
AATCCTCAGGAGGGGACCCAGAAGTTGTAAGGTATAGTTTTAATTGTGGAGGGGAATTTTTCTACTGTGATTC
CTCACAACTGTTTAATAG???????????????????????????????????????ATATTACTGGAATAAG
ACAAATATCACACTCCCATGTAGAATAAAACAAATAATAAACATGTGGCAGGAAGTAGGAAAAGCAATGTATG
CCCCTCCCATCAGTGGACAAATTAACTGTTCATCAAATATTACAGGGCTGCTATTAACAAGAGACGGTGGTAA
GGACGGTGATAATAGTACAGAGATTTTCAGACCGGGAGGAGGAAATATGAAGGACAATTGGAGAAGTGAATTA
TATAAATACAAAGTAGTAAAAATTGAACCATTAGGAATAGCACCCACCGAGGCAAAGAGAAGAGTGGTGCAGA
GAGAAAAAAGAGCAATAGGATTAGGAGCTTTGTTCCTTGGGTTCTTGGGAGCAGCAGGAAGCACTATGGGCGC
AGCGTCAATGACGCTGACGGTACAGGCCAGACAATTATTGTCTGGTATAGTGCAACAGCAGAACAATCTGCTG
AAGGCTATTGAGGCGCAACAGCATCTGTTGCAACTCACAGTCTGGGGCATCAAGCAGCTCCAGGCAAGAGTCC

TGGCTGTGGAAAGATACCTAAGAGATCAACAGCTCCTAGGGATTTGGGGTTGCTCTGGAAAACTCATCTGCAC
CACTAATGTGCCTTGGAACACTAGTTGGAGTAATAAGACTAAGGATGAGATTTGGAATAACAT?????GGATG
CAGTGGGAAAAAGAAATTGACAATTACACAGGCTTAATATACACTTTGCTTGAAGAATCACAGAACCAGCAGG
AAAAGAATGAACAAGAATTATTGGCATTGGATAAGTGGGCAAGTTTGTGGACTTGGCTTGACATAACAAACTG
GCTGTGGTATATAAAAATATTCATAATGATAGTAGGAGGCTTGATAGGTTTAAGAATAGTTTTTGCTGTACTT
TCTATAATAAATAGAGTTAGGCAGGGATACTCACCATTATCGTTGCAGACCCACCTCCCAGCTCCGAGGGGAC
TCGACAGGCCCGAAGGAATCGAAGAAGAAGGTGGAGAGACAGACAGAGGCAGATCAATTCGATTAGTGGATGG
CTTCTTAGCACTTTTCTGGGACGACCTGAGGAACCTGTGCCTCTTCAGCTACCACCGCTTGAGAGACTTACTC
TTGATTGTAACGAGGATTGTGGGACTTCTGGGACGCAGGGGGTGGGAACTCCTGAAATATTGGTGGAATCTCC
TGCAGTATTGGATTCAGGAACTAAAGAATAGTGCTACTAGCTTGCTGAACGCCACAGCTATAGCAGTTGCTGA
GGGGACAGATAGGATTATAGAAGTAGTACAAAGAATTCTTAGAGCTATCCTTCACATACCTAGAAGAATAAGA
CAGGGCTTTGAAAGGGCTTTGCTATAAGGGCTTTGCTATAAAATGGGTGGCAAGTGGTCAAAGATGATTGGAT
GGCCTGCTGTAAGGGAAAGAATGCACCGAGCTGAGCCAGCAGCAGAAGGGGTAGGAGCAGCATCTCGAGATCT
GGAGAGACATGGAGCACTTACAAGTAGCAATACAGCAGCTACTAATGCTGATTGTGCCTGGCTAGAAGCACAA
GAGGATGAGGAGGTGGGCTTTCCAGTCAGACCCCAGGTACCTTTAAGACCAATGACTTACAAGGGAGCGGTAG
ATCTCAGCCACTTTTTAAGAGAAAAGGGGGGACTGGAAGGGCTAATTTACTCCCAGAAGAGACAAGATATCCT
AGATCTGTGGGTCTACCACACACAAGGCTACTTCCCTGATTGGCACAACTACACACCAGGGCCAGGGGTCAGA
TATCCACTGACCTTTGGATGGTGCTTCAAGCTAGTACCAGTTGATCCAGACCAGGTAGAGAAGGCCAATGAAG
GGGAGAACAACAGCTTGCTGCACCCTATGAGCCTGCATGGGATAGAGGACCCGGAGAAAGAAGTGCTGATGTG
GAAGTTTGACAGCCGCCTAGCATTCCATCACATGGCCCGAGAGCTGCATCCGGAGTACTACAAGAACTGATGA
CACCGAGTTTCTACAAGGGACTTTCCGCTGGGGACTTTCCAGGGGAGGCGCGGCCTGGGCGGGACTGGGGAGT
GGCGAGCCCTCAGATGCTGCATATAAGCAGCTGCTTTTTGCCTGTACTGGGTCTCTCTGGTTAGACCAGATCT
GAGCCTGGGAGCTCTCTGGCTAACTAGGGAACCCACTGCTTAAGCCTCAATAAAGCTTGCCTTGAGTGCTTTA
AGTAGTGTGTGCCCGTCTGTTGTGTGACTCTGGTAACTAGAGATCCCTCAGACCCTTTAGTCAGTGTGGAAA?
????????

>K-B1 HIV-1 genome, derived from RNA genomic sequence
CCTATGAGCCAGCATGGGATGGAGGACCCGGAGGGAGAAGTATTAGTGTGGAAGCTTGAATGCTAGGCGGCTG
TCAAACACATGGCCCGAGAGCTGCATCCGGAGTACTATAAGAACTGATGACACCGAGTTTCTACAAGGGACTT
TCCGCTGGGGACTTTCCAGGGGAGGCGCGGCCTGGGCGGGACTGGGGAGTGGCGAGCCCTCAGATGCTGCATA
TAAGCAGCTGCTTTTTGCCTGTACTGGGTCTCTCTGGTTAGACCAGATCTGAGCCTGGGAGCTCTCTGGCTAA
CTAGGGAACCCACTGCTTAAGCCTCAATAAAGCTTGCCTTGAGTGCTTTAAGTAGTGTGTGCCCGTCTGTTGT
GTGACTCTGGTAACTAGAGATCCCTCAGACCCTTTAGTCAGTGTGGAAAATCTCTAGCAGTGGCGCCCGAACA
GGGACGCGAAAGCGAAAGTAAGACCGGAGGAGCTCTCTCGACGCAGGACTCGGCTTGCTGAAGCGCGCACAGC
AAGAGGCGAGGGGCGGCGACTGGTGAGTACGCCAAAATTTTGACTAGCGGAGGCTAGAAGGAGAG???????
???GTTAAGGCCAGGGGGAAK
AAAAGGTATAAATTAAAACATATAGTATGGGCAAGCAGGGAGCTAGAACGATTCGCAGTCAACCCTGGCCTGT
TAGAAACATCAGAAGGCTGTAGACAAATACTGGGACAGCTACAACCGTCCCTTCAGACAGGATCGGAAGAACT
TAGATCATTATTTAATACAATAGCAGTCCTCTATTGTGTACACCAAAGGATAGAGGTAAAAGATACCAAAGAA
GCTTTAGAGAAGGTAGAGGAAGAGCAAAACAAAAGTAAGAAAAAAGCACAGCAAGCAGCAGCAGCTGCAGCTG
ACACAGGAAACAGCGACAAGGTCAGCCAAAATTTTCCTATAGTGCAGAACCTACAGGGGCAAATGGTACATCA
GCCCCTATCACCTAGAACTTTAAATGCATGGGTAAAAGTAGTAGAAGAGAAGGCTTTTAGCCCAGAAGTAATA
CCCATGTTTGCAGCATTATCAGAAGGAGCCACCCCACAAGATTTAAACACCATGCTAAACACAGTGGGAGGAC
ATCAAGCAGCTATGCAAATGTTAAAAGAGACCATCAATGAGGAAGCTGCAGAATGGGATAGATTGCATCCAGT
GCATGCAGGGCCTGCTGCACCAGGCCAGATGAGAGAACCAAGGGGAAGTGACATAGCAGGAACTACTAGTACC
CTTCAGGAACAAATAGGCTGGATGACACATAATCCACCTATCCCAGTAGGAGAAATTTATAAAAAATGGATAA
TCATGGGATTAAATAAAATAGTAAGAATGTATAGCCCTACCAGCATTCTGGACATAAGACAAGGACCAAAGGA
ACCCTTTAGAGATTATGTAGACCGGTTCTATAAAACTCTAAGAGCTGAGCAAGCTTCACAGGAAGTAAAAAAT
TGGATGACAGAAACCTTGTTGGTCCAAAATGCAAACCCAGATTGTAAGACTATCCTAAAAGCATTAGGACCAG
CAGCCACACTAGAAGAAATGATGACAGCATGTCAGGGAGTAGGGGGACCCGGCCATAAAGCAAGAGTTTTGGC
CGAAGCAATGAGCCAGGTAACAAATTCAGCTACCGTAATGATGCAGAAAGGCAACTTTAGGAACCAAAGAAAG
GCTGTCAAGTGTTTCAATTGTGGCAAAGAAGGGCACATAGCCAGAAATTGCAGGGCCCCTAGGAAAAAGGGCT
GTTGGAAATGTGGAAAGGAAGG???
???
???
???
???
??AGCTA
TAGGTACAGTATTAATAGGACCTACACCTGTCAACATAATTGGAAGAAATCTGTTGACTCAGCTTGGTTG???
???
??????????????????????????AATAAAAGCATTAGTAGAAATTTGTACAGAAATGGAAAAGGAAGGAA
AAATTTCAAAAGTTGGGCCAGAA??
?????????????CTAGTAGATTTCAGAGAACTTAATAAGAGAACTCAAGACTTCTGGGAAGTTCAATTAGGA
ATACCACATCCTGCAGGGTTAAAAAAGAAAAAATCAGTAACAGTACTGGATGTAGGTGATGC???????????
??GTATAAATAATGAGACACCAGGGAT
TAGATATCAGTACAATGTACTTCCGCAGGGATGGAAAGGATCACCAGCAATATTCCAAAGTAGCATGACAAAA
ATCTTAGATCCTTTTAGAAAGCAAAATCCAGACATAGTTATTTACCAGTACATGGATGATTTGTATRTAGGAT
CTGACTTAGAAATAGGGCAGCACAGAATAAAAATAGAGGAACTAAGACAACATCTGTTGAAGTGGGGATTCAC
CACACCAGACAAGAAACATCAGAAAGAACCTCCATTCCTTTGGATGGGTTATGAACTCCATCCTGATAAATGG
ACAGTACAGCCTATAGAGCTGCCAGAAAAAGACAGCTGGACTGTCAATGACATACAGAAGTTAGTGGGAAAAT
TAAATTGGGCAAGTCAGATTTACGCAGGGATTAAAGTAAGGCAATTATGTAAACTCCTTAGGGGAGCCAAAGC
ACTAACAGAAGTAATAACACTAACAGAAGAAGCAGAACTAGAGCTGGCAGAAAACAGGGAGATTCTAAAAACA
CCAGTACATGGAGTGTACTATGACCCATCAAAAGACTTAGTAGCAGAAATACAGAAGCAGGGGCAAGGTCAAT
GGACATATCAAATTTATCAAGAGCCATTTAAAAATCTGAAAACAGGAAAGTATGCAAGAATGAGGAGTACCCA
CACTAATGATGTAAAACAGTTAACAGAGGCAGTGCAAAAAATATCCACAGAAAGCATAGTAATATGGGGAAAG
ACCCCTAAATTTAAACTACCTATACAAAAGGAAACATGGGAAACATGGTGGGCGGAGTATTGGCAAGCCACCT
GGATTCCTGAGTGGGAATTTGTC?????????????????????CTATGGTACCAGTTAGAAAAAGAACCCAT
AGTAGGAGCAGAAACTTTCTATGTAGATGGGGCAGCTAATAGAGAAACTAAATTAGGAAAAGCAGGCTATGTT
ACTGACAGAGGTAGACAAAAAGTTGTCTCCCTAACTGGCACAACAAATCAGAAAACTGAGTTACAAGCTATTA
ATCTAGCTTTGCAGGATTCAGGGTTAGAAGTAAATATAGTAACAGACTCACAATATGCACTGGGAATCCTTCA

AGCGCAACCAGATAAGAGTGAATCAGAGATAGTCAGTCATATAATAGAGCAGTTAATAAATAAGGAAAGGGTC
TACCTGGCATGGGTACCAGCACACAAAGGAATTGGGGGAAATGAACAAGTAGATAAATTGGTCAGTGCTGGAA
TCAGAAGAGTACTATTCTTAGATGGAATAGATAAGGCCCAAGAGGAGCATGAAAGATATCACAGTAATTGGAG
AGCAATGGCTAGTGATTTTAACCTACCACCTGTAGTAGCAAAAGAAATAGTAGCCTGCTGTGATAAATGTCAA
CTAAAAGGAGAAGCCATGCATGGACAAGTAG???????????GGAATATGGCAACTAGATTGTACACATCTAG
AAGGAAAAATTATCATAGTAGCAGTTCATGTAGCCAGTGGATATATAGAGGCAGAAGTTATTCCAGCAGAGAC
AGGGCAGGAAACAGCATACTTTCTCTTAAAATTAGCAGGGAGATGGC??????????????????????????
???
???
???
????????????????????GGAAAGAATAATAGACATAATAGCATCAGACATACAGACTAAAGAACTACAAA
AACAAATCACAAAAATTCAAAATTTTCGGGTTTATTACAGGGACAGCAGAGATCCACTTTGGAAAGGACCAGC
AAAGCTCCTTTGGAAAGGTGAAGGGGCAGTAGTAATACAAGATAATAGTGATATAAAAGTAGTGCCAAGAAGA
AAAGCAAAGATCATTAGAGATTATGGAAAACAGATGGCAGGTGATGATTGTGTGGCAGGTGGACAGGATGAGG
ATTAGAACATGGAAAAGTTTAGTAAAACACCAMATGTATAWTTCAAAGAAAGCTAAGRGATGGATWTATAAAC
ATCACTATGAAAGCACTCATCCAAGAATAAGTTCAGAAGTACACATCCCATTAGGGGATGCTAAATTGGTAAT
GATAACATATTGGGGTCTGCATACAGGAGAAAGAGACTGGCATTTGGGCCAGGGAGCCTCCATAGAATGGAGG
AAAGAGAGATATAGCACACAAGTAGACCCTGGCCTAGCAGACCAACTAATTCATATGCACTATTTTGATTGTT
TTTCAGAATCTGCTATAAGAAATGCCATATTAGGACGTATAGTTAGTCCTAGGTGTGAATATCAAGCAGGACA
TAACAAGGTAGGATCTCTACAATATTTGGCACTAACAGCATTAATAAAACCAAAAGGGATAAAGCCACCTTTG
CCTAGTGTAGCGAAACTGACAGAGGATAGATGGAACAAGCCCCAGAGGACCAAGGGCCACAGAGGGAGCCATA
CAATGAATGGGCACTAGGGATTCTAGAGGAACTTAAAAATGAAGCTGTTAGACATTTTCCTGGGCCCTGGCTC
CAAGGCTTAGGACAATATATTTATGTAACTTATGGAAATACTTGGACAGGAGTGGAAGCCCTAATAAGAACTC
TGCAACAACTGCTGTTTATTCATTTCAGAATTGGGTGTCGGCATAGCAGAATAGGCATTGACATCCGACAGAG
RAGAGC???GCCTAGGACTGC
TTGTAATAGTTGCTATTGTAAAAAGTGTTGCCTTCATTGCCAAGTTTGCTTCTTAAGAAAAGGCTTAGGCATC
TCCTATGGCAGGAAGAAGCGGAGACAGCGACGAAGAACTCCTTCAGAAAGTCAGACTCATCCGGCTTCTCTAC
CAAAGCAGTAAGTTCTTTATCAAAGCAGTAAGTAGTATATGTAATGCAACCTGTACAAATATTAGCAATAGTA
GCATTAGTAGTAGTAGCAATAATAGCAATAGTTGTATRGRYYRYAKTASMYATASAATATAGAAAAATATTAA
GACAAAGGAAAATAGACAGGTTAATTGAAAGAATAAGTGAAAGAGCAGAAGACAGTGGCAATGAGAGCGAAGG
GGATCAGGAAGAGTTGTCAGCACTTGTGGAGATGGGGCATCATGCTCCTTGGGATATTGATGATCTGTAGTGC
TAGAGAACAATTGTGGGTCACAGTCTATTATGGGGTACCTGTGTGGAAAGAAGCAACTACCACTCTATTTTGT
GCCTCAGATGCTAAAGCATATGATACAGAAAGTCATAATGTTTGGGCCACACATGCCTGTGTACCCACAGACC
CCAACCCACAAGAAGTAAGATTGAAAAMTGTGACAGAAGAATTTAACATGGGAAAAAATAATATGGTAGATCA
AATGCATGAGGATATAATCAGCTTATGGGATCAAAGCTTAAAGCCATGTGTAAAATTAACCCCACTCTGTGTT
AT?TCACTATA??ATAATGTTGCTCTGAGCATTAGTGCTGTTAGTATTATTCCTCTTCGTCGGCTCAGTGCAA
TGTA?AAGTA?A??CACAAAGTGGGGTTAATTTTACACATGGCTTTAAGCGTACCAATAGATGATGATAATAA
TACTAAGAACTATAGTAACTATARGTTGATAAGTTGCAACACCTCAGTCATTAGACAGGCCTGTCCAAAGGTA
TCTTTTGAGCCAATTCCCATACATTATTGTACTGTGCTGACATCGCTGMWTMKWMMKKKTMWMKWGRAMWWKT
YCWMKKKAMMMGGACTATGCAGCGATGTCAGCACAGTACAATGTACACATGGAATTAAACCAGTAGTGTCAAC
TCAACTGCTGTTAAATGGCAGCCTAGCAAAAGAAGAGGTAGTACTTAGGTCTGAAAATTTCACAGAAAATACT
AAAACCATAATAGTGCAGCTGAAGGACCCTGTAGTAATTAATTGTACAAGACCCAACAACAATATAAGAAAAA
GGATACGTATAGGACCAGGGGGCGCATCATTTTATGCAACAAACATAATGGGAGATATAAGAAAAGCATATTG
TATCATTAATAGCACACAATGGAATAACACTTTACAACAGGTAGTTAAAAAATTAAGAGAAAAAAGATG??AG
GATT?AACAATAGTCTTTAATCAATCCTCAGGAGGGGACCCAGAAGTTGTAAGGTATAGTTTTAATTGTGGAG
GGGAATTTTTCTACTGTGATTCCTCACAACTGTTTAATAGTACCTGGAATATTAGTGATATTAATAGTGATAT
TAGTAATATTACTAAAAATAAGACAAATATCACACTCCCATGTAAAATAAAACAAATAATAAACATGTGGCAG
AAAGTAAAAAAAGCAATGTATGMCC??
??????GTAATAACGGTGATAATGAAAACAGTACAGAGATTTTCAGACCAGGAGGAGGAAATATGAAGGACAA
TTGGAGAAG???GAATAGCACCCACCGAGGCAAAG
AGAAGAGTGGTGCAGAGAGAAAAAAGAGCAATAGGATTAGGAGCTTTGTTCCTTGGGTTCTTGGGAGCAGCAG
GAAGCACTATGGGCGCAGCGTCAATGACGCTGACGGTACAGGCCAGACAATTATTGTCTGGTATAGTGCAACA
GCAGAACAATCTGCTGAAGGCTATTGAGGCGCAACAGCATCTGTTGCAACTCACAGTCTGGGGCATCAAGCAG
CTCCAGGCAAGAGTCCTGGCTGTGGAAAGATACCTAAGAGATCAACAGCTCCTAGGGATTTGGGGTTGCTCTG

GAAAACTCATCTGCACCACTAATGTGCCTTGGAATACTAGTTGGAGTAATAAGACTAAGGATGAGATTTGGAA
TAACATGACCTGGATGCAGTGGGAAAAAGAAATTGACAATTACACAGGCTTAATATACACTTTGCTTGAAGAA
TCACAGAACCAGCAGGAAAAGAATGAACAAGAATTATTGGCATTGGATAAGTGGGCAAGTTTGTGGACTTGGC
TTGACATAACAAACTGGCTGTGGTATATAAAAATATTCATAATGATAGTAGGAGGCTTGATAGGTTTAAGAAT
AGTTTTTGCTGTACTTTCTATAATAAATAGAGTTAGGCAGGGATACTCACCATTATCGTTGCAGACCCACCTC
CCAGCTCCGAGGGGACTCGACAGGCCCGAAGGAATCGAAGAAGAAGGTGGAGAGACAGACAGAGGCAGATCAA
TTCGATTAGTGGATGGCTTCTTAGCACTTTTCTGGGACGACCTGAGGAACCTGTGCCTCTTCAGCTACCACCG
CTTGAGAGACTTACTCTTGATTGTAACGAGGATTGTGGGACTTCTGGGACGCAGGGGGTGGGAACTCCTGAAA
TATTGGTGGAATCTCCTGCAGTATTGGATTCAGGAACTAAAGAATAGTGCTACTAGCTTGCTGAACGCCACAG
CTATAGCAGTTGCTGAGGGGACAGATAGGATTATAGAAGTACAAAGAGTTCTTAGAG?TATCCTTCACATACC
TAGAAGAATAAGACAGGGCTTCGAAAGGGCTTTGCTATAAGGGCTGTGCTATAAAATGGGTGGCAAGTGGTCA
AAGATGATTGGATGGCCTGCTGTAAGGGAAAGAATGCAGCAAGCTGAGCCAGCAGCAGAAGGGGTAGGAGCAG
CATCTCGAGATCTGGAGAGACATGGAGCGCTTACAAGTAGCAATACAGCAGCTACTAATGCTGACTGTGCCTG
GCTAGAAGCACAAGAGGATGAGGAGGTGGGCTTTCCAGTCAGACCCCAGGTACCTTTAAGACCAATGACTTAC
AAGGGAGCGGTAGATCTCAGCCACTTTTTAAGAGAAAAGGGGGGACTGGAAGGGCTAATTTACTCCCAGAAGA
GACAAGATATCCTAGATCTGTGGGTTTACCACACACAAGGCTACTTCCCTGATTGGCAAAACTACACACCAGG
GCCAGGGGTCAGATATCCACTGACCTTTGGATGGTGCTTCAAGCTAGTACCAGTTGATCCAGACCAGGTAGAG
AAGGCCAATGAAGGGGAGAACAACAGCTTGCTGCACCCTATGAGCCTGCATGGGATAGAGGACCCGGAGAAAG
AAGTGCTGATGTGGAAGTTTGACAGCCGCCTAGCATTCAAGCACATGGCCCGAGAGCTGCATCCGGAGTACTA
TAAGAACTGATGACACCGAGTTTCTACAAGGGACTTTCCGCTGGGGACTTTCCAGGGGAGGCGCGGCCTGGGC
GGGACTGGGGAGTGGCGAGCCCTCAGATGCTGCATATAAGCAGCTGCTTTTTGCCTGTACTGGGTCTCTCTGG
TTAGACCAGATCTGAGCCTGGGAGCTCTCTGGCTAACTAGGGAACCCACTGCTTAAGCCTCAATAAAGCTTGC
CTTGAGTGCTTTAAGTAGTGTGTGCCCGTCTGTTGTGTGACTCTGGTAACTAGAGATCCCTCAGACCCTTTAG
TCAGTGTGGAAAATCTGCAGC

>K-B3 HIV-1 genome, derived from RNA genomic sequence
CCTATGAGCCTGCATGGGATAGAGGACCCGGAGAAAGAAGTGCTGATGTGGAAGTTTGACAGCCGCCTAGCAT
TCAAGCACATGGCCCGAGAGCTGCATCCGGAGTACTATAAGAACTGATGACACCGAGTTTCTACAAGGGACTT
TCCGCTGGGGACTTTCCAGGGGAGGCGCGGCCTGGGCGGGACTGGGGAGTGGCGAGCCCTCAGATGCTGCATA
TAAGCAGCTGCTTTTTGCCTGTACTGGGTCTCTCTGGTTAGACCAGATCTGAGCCTGGGAGCTCTCTGGCTAA
CTAGGGAACCCACTGCTTAAGCCTCAATAAAGCTTGCCTTGAGTGCTTTAAGTAGTGTGTGCCCGTCTGTTGT
GTGACTCTGGTAACTAGAGATCCCTCAGACCCTTTAGTCAGTGTGGAAAATCTCTAGCAGTGGCGCCCGAACA
GGGACGCGAAAGCGAAAGTAAGACCGGAGGAGCTCTCTCGACGCAGGACTCGGCTTGCTGAAGCGCGCACGGC
AAGAGGCGAGGGGCGGCGACTGGTGAGTACGCCAAAATTTTTGACTAGCGGAGGCTAGAAGGAGAGAGATGGG
TGCGAGAGCGTCAGTATTAAGCGGGGGAGAATTAGATAAATGGGAAAAAATTCGGTTAAGGCCAGGGGGAAAG
AAAAGGTATAAATTAAAACATATAGTATGGGCAAGCAGAGAGCTAGAACGATTCGCAGTCAACCCTGGCCTGT
TAGAAACATCAGAAGGCTGTAGACAAATACTGGGACAGCTACAACCGTCCCTTCAGACAGGATCAGAAGAACT
TAGATCATTATTCAATACAGTAGCAGTCCTCTATTGTGTACACCAAAGGATAGAGGTAAAAGATACCAAAGAA
GCTTTAGAGAAGGTAGAGGAAGAGCAAAACAAAAGTAAGAAAAAGGCACAGCAAGCAGCAGCAGCTGCGGCTG
ACACAGGAAATGGCGGCAAGGTCAGCCAAAATTTTCCTATAGTGCAGAACCTACAGGGGCAAATGGTACATCA
GCCCCTATCACCTAGAACTTTAAATGCATGGGTAAAAGTAGTAGAAGAGAAGGCTTTTAGCCCAGAAGTAATA
CCCATGTTTGCAGCATTATCAGAAGGAGCCACCCCACAAGATTTAAACACCATGCTAAACACAGTGGGAGGAC
ATCAAGCAGCTATGCAAATGTTAAAAGAGACCATCAATGAGGAAGCTGCAGAATGGGATAGATTGCATCC???
???CTACTAGTACC
CTTCAGGAACAAATAGGCTGGATGACACATAATCCACCTATCCCAGTAGGAGAAATTTATAAAAAATGGATAA
TCATGGGATTAAATAAAATAGTAAGAATGTATAGCCCTACCAGCATTCTGGACATAAGACAAGGACCAAAGGA
ACCCTTTAGAGACTATGTAGACCGGTTCTATAAAACTCTAAGAGCTGAGCAGGCTTCACAAGAGGTAAAAAAT
TGGATGACAGAAAMC???
????????????????AATGATGACAGCATGTCAGGGAGTAGGGGGACCCGGCCATAAAGCGAGAGTTTTGGC
CGAAGCAATGAGCCAGGTAACAAATTCAGCTACCGTAATGATGCAGAAAGGCAACTTTAGGAACCAAAGAAAG
GCTGTCAAGTGTTTCAATTGTGGCAAAGAAGGGCACATAGCCAGAAATTGCAGGGCCCCTAGGAAAAAGGGTT
GTTGGAAATGTGGAAAGGAAGGACACCAAATGAAGGATTGTGTTGAGAGACAGGCTAATTTTTTAGGGAAAAT
CTGGCCTTCCCACAAGGGAAGGCCAGGGAATTTCCTTCAGAGCAGACCAGAGCCAACAGCCCCACCAGAGGAG
AGCCTCAGGTTTGGGGAGGAGACAACAACTCCCTATCAGAAACAGGAGCAGATGGACAAGGAGAAGTATCCTT
TGACTTCCCTCAGATCACTCTTTGGCAACGACCCCTTGTCACAATAAAAGTAGGAGGGCAGCTAAAGGAAGCT
CTATTAGATACAGGAGCAGATGATACAGTACTAGAAGAAATGAATTTGCCAGGAAGATGGAAACCAAAAATGA
TAGGGGGAATTGGAGGTTTTATCAAAGTAAAACAGTATGATCAGATACCCATAGAAATCTGTGGGCATAAAGC
TATAGGTACAGTATTAGTAGGACCTACACCTGTCAACATAATTGGAAGAAATCTGTTGACTCAGCTTGGTTGC
ACTTTAAATTTTCCCATTAGTCCTATTGAAACTGTACCAGTAAAATTAAAGCCAGGAATGGATGGCCCAAAAG
TTAAACAGTGGCCATTGACAGAGGAAAAAATAAAAGCATTAGTAGAAATTTGTACAGAAATGGAAAAGGAAGG
AAAAATTTCAAAAATTGGGCCAGAAAATCCATACAATACTCCAGTATTTGCCATAAAGAAAAAAGATGGTACT
AAATGGAGAAAACTAGTAGATTTCAGAGAACTTAATAAGAGAACTCAAGACTTCTGGGAAGTTCAATTAGGAA
TACCACACCCTGCAGGGTTAAAAAAGAAAAAATCAGTAACAGTACTGGATGTAGGTGATGCTTATTTTTCAGT
TCCCTTAGATAAAGACTTCAGGAAGTATACTGCATTTACCATACCCAGTATAAATAATGAGACACCAGGGATT
AGATATCAGTACAATGTACTTCCGCAGGGATGGAAAGGATCACCAGCAATATTCCAAAGTAGCATGACAAAAA
TCTTAGATCCTTTTAGAAAGCAAAATCCAGACATAGTTATTTACCAATACATGGATGATTTGTATGTAGGATC
TGACTTAGAAATAGGGCAGCACAGAATAAAAATAGAGGAACTAAGACAACATCTGTTGAAGTGGGGATTCACC
ACACCAGACAAGAAACATCAGAAAGAACCTCCATTCCTTTGGATGGGTTATGAACTCCATCCTGATAAATGGA
CAGTACAGCCTATAGAGCTGCCAGAAAAAGACAGCTGGACTGTCAATGACATACAGAAGTTAGTGGGAAAATT
AAATTGGGCAAGTCAGATTTACGCAGGGATTAAAGTAAGGCAATTATGTAAACTCCTTAGGGGAGCCAAAGCA
CTAACAGAAGTAATAACACTAACAGAAGAAGCAGAACTAGAGCTGGCAGAAAACAGGGAGATTCTAAAAACAC
CAGTACATGGAGTGTACTATGACCCATCAAAAGACTTAGTAGCAGAAATACAGAAGCAGGGGCAAGGTCAATG
GACATATCAAATTTATCAAGAGCCATTTAAAAATCTGAAAACAGGAAAGTATGCAAGAATGAGGAGTACCCAC
ACTAATGATGTAAAACAGTTAACAGAGGCAGTGCAAAAAATATCCACAGAAAGCATAGTAATATGGGGAAAGA
CCCCTAAATTTAAACTACCTATACAAAAGGAAACATGGGAAACATGGTGGGCGGAGTATTGGCAAGCCACCTG
GATTCCTGAGTGGGAATTTGTCAATACCCCTCCCCTAGTGAAACTATGGTACCAGTTAGAAAAAGAACCCATA
GCAGGAGCAGAAACTTTCTATGTAGATGGGGCAGCTAATAGAGAAACTAAATTAGGAAAAGCAGGATATGTTA
CTGACAGAGGAAGACAAAAAGTTGTCTCTCTAACTGACACAACAAATCAGAAAACTGAGTTACAAGCTATTAA
TCTAGCTTTGCAGGATTCAGGGTTAGAAGTAAATATAGTAACAGACTCACAATATGCACTGGGAATCCTTCAA

GCACAACCAGATAAGAGTGAATCAGAGATAGTCAGTCATATAATAGAGCAGTTAATAAATAAGGAAAGGGTCT
ACCTGGCATGGGTACCAGCACACAAAGGAATTGGGGGAAATGAACAAGTAGATAAATTGGTCAGTAATGGAAT
CAGAAGAGTACTATTCTTAGATGGAATAGATAAGGCCCAAGAGGAGCATGAAAGATATCACAGTAATTGGAGA
GCAATGGCTAGTGATTTTAACCTACCACCTGTAGTAGCAAAAGAAATAGTAGCCTGCTGTGATAAATGTCAAC
TAAAAGGAGAAGCCATGCATGGACAAGTAGACTGTAGTCCAGGAATATGGCAACTAGATTGTACACATCTAGA
AAGAAAAATTATCATAGTAGCAGTTCATGTAGCCAGTGGATATATAGAGGCAGAAGTTATTCCAGCAGAGACA
GGGCAGGAAACAGCATACTTTCTCTTAAAATTAGCAGGGAGATGGCCAGTAAAAACAATACATACAGATAATG
GCCCCAATTTCATTAGCTCCGCGGTCAAGGCCGCCTGTTGGTGGGCAGGGATCAAGCAGGAATTTGGCATTCC
CTACAATCCCCAAAGTCAAGGAGTAGTAG??
???
???
???
???
???
???
???
???CCTCCATAGAGTGGAGGA
AAGAGAAATATAGCACACAAATAGACCCTGGCCTAGCAGACCAACTAATTCATATGCACTATTTTGATTGTTT
TTCAGAATCTGCTATAAGAAATGCCATATT???
???
??GGGCCACAGAGGGAGCCATAC
AATGAATGGGCACTAGGGATTTTAGAGGAACTTAAAAATGAAGCTGTTAGACATTTTCCTGGGCCCTGGCTCC
AGGGCTTAGGACAATACATTTATGTAACTTATGGAAATACTTGGACAGGAGTGGAAGCCCTAATAAG??????
???????????????????????????????????????GCATAGCAGAATAGGCATTGACATCCGACAGAGG
AGAGCAAGAAATGGATCCAGTAGATCCTAGACTAGAGCCCTGGAAGCATCCAGGAAGTCAGCCTAGGACTGCT
TGTAATAGTTGCTATTGTAAAAAGTGTTGCCTTCATTGCCAAGTTTGCTTCTTAAGAAAAGGCTTAGGCATCT
CCTATGGCAGGAAGAAGCGGAGACAGCGACGAAGAACTCCTTCAGAAAGTCAGACTCATCCGGCTTCTCTACC
AAAGCAGTAAGTTCTTTATCAAAGCAGTAAGTAGTATATGTAATGCAACCTGTACAAATATTAGCAATAGTAG
CATTAGTAGTGGTAGCAATAATAGCAATAGTTGTATGGACCATAGTAGCCATAGAATATAGAAAAATATTAAG
ACAAAGAAAAATAGACAGGTTAATTGAAAGAATAAGTGAAAGAGCAGAAGACAGTGGCAATGAGAGCGAGGGG
GATCAGGAAGAGTTGTCAGCACTTGWGGAG???
??CTGTAGTGCTGGAGAACAATTGTGGGTCACAGTCTATTATGGGGTACCTGTGTGGAAAGAAGCAACTACCA
CTCTATTTTGTGCCTCAGATGCTAAAGCAT?????????????????????????????ACACATGCCTGTGT
ACCCACAGACCCCAACCCACAAGAAGTAAGATTGAAAAATGTGACAGAAGAATTTAACATGGAAAAAATAAT
ATGGTAGATCAAATGCATGAGGATATAATCAGC??
???
???
??TGATAATATTACTAATAAGAATA
ACTATAGTACCTATAGGTTGATAAGTTGCAACACCTCAGTCATTAGACAGGCCTGTCCAAAGGTATCTTTTGA
GCCAATTCCCATACATTATTGTGCCCCTGCTGGTTTTGCGCTTCTAAAGTGTAACGAGGAAAAGTTCAATGGA
ACAGGACTATGCAGCAATGTCAGCACAGTACAATGTACACATGGAATTAAACCAGTAGTGTCAACTCAACTGC
TGTTAAATGGCAGCCTAGCAAAAGAAGAGGTAGTACTTAGGTCTGAAAATTTCACAGAAAATACTAAAACCAT
AATAGTGCAGCTGAAGGACCCTGTAGTAATTAATTGTACAAGACCCAACAACAATATAAGAAAAAGRAWACGT
AT???
?????????????????GAATAACACTTTACAACAGGTAGTTAAAAAATTAAGAGAACAATTTGGAATAAAAC
AATAGTCTTTAATCAATCCTCAGGAGGGGACCCAGAAGTTGTAAGGTATAGTTTTAATTGTGGAGGGGAATTT
TTCTACTGTGATTCCTCACAACTGTTTAATAGTACTTGGAATATTAGT?????????????????????????
????????????????????ATCACACTCCCATGTAGAATAAAACAAATAATAAACATGTGGCAGGAAGTAGG
AAAAGCAATGTATGCCCCTCCCATCAGTGGACTAATTAACTGTTCATCAAATATTACAGGGCTGCTATTAACA
AGAGACGGTGGTAATGACAATAGTAATGAAAACAGTACAGAGACTTTCAGACCAGGAGGAGGAAATATGAAGG
ACAATTGGAGAAKTGAATTATATAAATA?????????????????????ATTAGGAATAGCACCCACCGAGGC
AAAGAGAAGAGTGGTGCAGAGAGAAAAAAGAGCAATAGGATTAGGAGCTTTGTTCCTTGGGT???????????
???????????????????????????????????????GTACAGGCCAGACAATTATTGTCTGGTATAGTGC
AACAGCAGAACAATCTGCTGAAGGCTATTGAGGCGCAACAACATCTGTTGCAACTCACAGTCTGGGGCATCAA

GCAGCTCCAGGCAAGAGTCCTGGCTGTGGAAAGATACCTAAGAGATCAACAGCTCCTAGGGATTTGGGGTTGC
TCTGGAAAACTCATCTGCACCACTAATGTGCCTTGGAATACTAGTTGGAGTAATAAGACTAAGGATGAGATTT
GGAATAACATGACCTGGATGCAGTGGGAAAAAGAAATTGACAATTACACAGGCTTAATATACACTTTGCTTGA
AGAATCACAGAACCAGCAGGAAAAGAATGAACAAGAATTATTGGCATTGGATAAGTGGGCAAGTTTGTGGACT
TGGCTTGACATAACAAACTGGCTGTGGTATATAAAAATATTCATAATGATAGTAGGAGGCTTGATAGGTTTAA
GAATAGTTTTTGCTGTACTTTCTATAATAAATAGAGTTAGGCAGGGATACTCACCATTATCGTTGCAGACCCA
CCTCCCAGCTCCGAGGGGACTCGACAGGCCCGAAGGAATCGAAGAAGAAGGTGGAGAGACAGACAGAGGCAGA
TCAATTCGATTAGTGGATGGCTTCTTAGCACTTTTCTGGGACGACCTGAGGAACCTGTGCCTCTTCAGCTACC
ACCGCTTGAGAGACTTACTCTTGATTGTAACGAGGATTGTGGGACTTCTGGGACGCAGGGGGTGGGAACTCCT
GAAATATTGGTGGAATCTCCTGCAGTATTGGATTCAGGAACTAAAGAATAGTGCTACTAGCTTGCTGAACGCC
ACAGCTATAGCAGTTGCTGAGGGAACAGATAGGATTATAGAAGTAGTACAAAGAGTTCTTAGAGCTATCCTTC
ACATACCTAGAAGAATAAGACAGGGCTTCGAAAGGGCTTTGCTATAAGGGCTTTGCTATAAAATGGGTAACAA
ATGGTCAAAGATGATTGGATGGCCTGCTGTAAGGGAAAGAATGCACCGAGCTGAGCCAGCAGCAGAAGGGGTA
GGAGCAGCATCTCGAGACCTGGAGAGACATGGGGCACTCACAAGTAGCAATACAGCAGCAACTAATGCTGATT
GTGCCTGGCTAGAAGCACAAGAGGATGAGGAGGTGGGCTTTCCAGTCAGACCCCAGGTACCTTTAAGACCAAT
GACTTACAAGGGAGCGGTAGATCTTAGCCACTTTTTAAGAGAAAAGGGGGGACTGGAAGGGCTAATTTACTCC
CAGAAGAGACAAGATATCCTAGATCTGTGGGTCTACCACACACAAGGCTACTTCCCTGATTGGCACAACTACA
CACCAGGGCCAGGGGTCAGATATCCACTGACCTTTGGATGGTGCTTCAAGCTAGTACCAGTTGATCCAGACCA
GGTAGAGAAGGCCAATGAAGGGGAGAACAACAGCTTGCTGCACCCTATGAGCCTGCATGGGATAGAGGACCCG
GAGAAAGAAGTGCTGATGTGGAAGTTTGACAGCCGCCTAGCATTCAAGCACATGGCCCGAGAGCTGCATCCGG
AGTACTATAAGAACTGATGACACCGAGTTTCTACAAGGGACTTTCCGCTGGGGACTTTCCAGGGGAGGCGCGG
CCTGGGCGGGACTGGGGAGTGGCGAGCCCTCAGATGCTGCATATAAGCAGCTGCTTTTTGCCTGTACTGGGTC
TCTCTGGTTAGACCAGATCTGAGCCTGGGAGCTCTCTGGCTAACTAGGGAACCCACTGCTTAAGCCTCAATAA
AGCTTGCCTTGAGTGCTTTAAGTAGTGTGTGCCCGTCTGTTGTGTGACTCTGGTAACTAGAGATCCCTCAGAC
CCTTTAGTCAGTGTGGAAAATCTCTAGC

>K-B4 HIV-1 genome, derived from RNA genomic sequence
CCTATGAGCCTGCATGGGATAGAGGACCCGGAGAAAGAAGTGCTGATGTGGAAGTTTGACAGCCGCCTAGCAT
TCAGTCACATGGCCCGAGAGCTGCATCCGGAGTACTACAAGAACTGATGACACCAAGTTTCTACAAGGGACTT
TCCACTGGGGACTTTCCAGGGGAGGCGCGGCCTGGGCGGGACTGGGGAGTGGCGAGCCCTCAGATGCTGCATA
TAAGCAGCTGCTTTTTGCCTGTACTGGGTCTCTCTTGTTAGACCAGATCTGAGCCTGGGAGCTCTCTGGCTAA
CTAGGGAACCCACTGCTTAAGCCTCAATAAAGCTTGCCTTGAGTGCTTTAAGTAGTGTGTGCCCGTCTGTTGT
GTGACTCTGGTAACTAGAGATCCCTCAGACCCTTTAGTC??????????????????????????????????
???
???
????????CKTCAGTATTAAGCGGGGGAAAATTAGATAAATGGGAAAAAATTCGGTTAAGGCCAGGGGGAAAG
AAAAGGTATAAGCTAAAACATATAGTATGGGCAAGCAGGGAGCTAGAACGATTCGCAGTCAACCCTGGCCTGT
TAGAAACATCAGAAGGCTGTAGACAAATACTGGGACAGCTACAACCGTCCCTTCAGACAGGATCAGAAGAACT
TAGATCATTATTTAATACAATAGCAG??????????????????????????????????GGATACCAAAGAA
GCTTTAGAGAAGGTAGAGGAAGAGCAAAACAAAAGTAAGAAAAAAGCACAGCAAGCAGCAGCAGCTGCGGCTG
ACACAGGAAATGGCGGCAAGGTCAGCCAAAATTTTCCTATAGTGCAGAACCTACAGGGGCAAATGGTACATCA
GCCCCTATCACCTAGAACTTTAAATGCATGGGTAAAAGTAGTAGAAGAGAAGGCTTTTAGCCCAGAAGTAATA
CCCATGTTTGCAGCATTATCAGAAGGAGCCACCCCACAAGATTTAAACACCATGCTAAACACAGTGGGAGGAC
ATCAAGCAGCTATGCAAATGTTAAAAGAGACCATCAATGAGGAAGCTGCARAATGGGATAGATTGCATCCAGT
GCATGCAGGGCCTGCTGCACCAGGCCAGATGAGAGAACCAAGGGGAAGTGACATAGCAGGAACTACTAGTACC
CTTCAGGAACAAATAGGCTGGATGACACATAATCCACCTATCCCAGTAGGAGAAATTTATAAAAAATGGATAA
TCATGGGATTAAATAAAATAGTAAGAATGTATAGCCC????????????????????????????????????
???GAGGTAAAAAAT
TGGATGACAGAGACCTTGTTGGTCCAAAATGCGAACCCAGATTGTAAGACTATCTTAAAAGCATTAGGACCAG
CAGCCACACTAGAAGAAATGATGACAGCATGTCAGGGAGTAGGGGGACCCGGCCATAAAGCAAGAGTTTTGGC
CGAAGCAATGAGCCAGGTAACAAATTCAGCTACCGTAATGATGCAGAAAGGCAACTTTAGGAACCAAAGAAAG
GTTGTCAAGTGTTTCAATTGTGGCAAAGAAGGGCATATAGCCAGAAATTGCAGGGCCCCTAGGAAGAAGGGCT
GTTGGAAATGTGGAAAGGAAGGACACCAAATGAAGGATTGTGTTGAGAGACAGGCTAATTTTTTAGGGAAAAT
CTGGCCTTCCCACAAAGGAAGGCCAGGGCCAGAGCCAACAGCCCCACTAGAGCCAACAGCCCCACCAGAGGAG
AGCCTCAGGTTTGGGGAGGAGACAACAACTCCCTATCAGRAACAGGAGCAGATGGACAAGGAGAAGTATCCTT
TGACTTCCCTCAGATCACTCTTTGGCAACGACCCCTTGTCACAATAAAAGTAGGAGGGCAGCTAAAGGAAGCT
CTATTAGATACAGGAGCAGATGATACAGTACTAGAAGAAATGAATTTGCCAGGAAGATGGAAACCAAAAATGA
TAGGGGGAATTGGAGGTTTTATCAAAGTAAGACAGTATGATCAGATAGCCATAGAAATCTGTGGACATAAAGC
TATAGGTACAGTATTAGTAGGACCTACACCTGTCAACATAATTGGAAGAAATCTGTTGACTCAGCTTGGTTGC
ACTTTAAATTTTCCCATTAGTCCTATTGAAACTGTACCAGTAAAATTAAAGCCAGGAATGGATGGCCCAAAAG
TTAAACAGTGGCCATTGACAGAGGAAAAAATAAAAGCATTAGTAGAAATTTGTACAGAAATGGAAAAGGAAGG
AAAAATTTCAAAAGTTGGGCCAGAAAATCCATACAATACTCCAGTATTTGCCATAAAGAAAAAAGATGGTACT
AAATGGAGAAAACTAGTAGATTTCAGAGAACTTAATAAGAGAACTCAAGACTTCTGGGAAGTTCAATTAGGAA
TACCACATCCTGCAGGGTTAAAAAAGAAAAAATCAGTAACAGTACTGGATGTAGG??????????????????
???
???GCAATATTCCAAAGTAGCATGACCAAAA
TCTTAGATCCTTTTAGAAAGCAAAATCCAGACATAGTCATTTACCAGTACATGGATGATTTGTATGTAGGATC
TGACTTAGAAATAGGGCAGCACAGAATAAAAATAGAGGAACTAAGACAACATCTGTTGAAGTGGGGATTCACC
ACACCAGACAAGAAACATCAGAAAGAACCTCCATTCCTTTGGATGGGTTATGAACTCCATCCTGATAAATGGA
CAGTACAGCCTATAGAGCTGCCAGAAAAAGAAAGCTGGACTGTCAATGACATACAGAAGTTAGTGGGAAAATT
AAATTGGGCAAGTCAGATTTATGCAGGGATTAAAGT?????????????????????????????????????
???AAAAACAC
CAGTACATGGAGTGTACTATGACCCATCAAAAGACTTAGTAGCAGAAATACAGAAGCAGGGGCAAGGTCAATG
GACATATCAAATTTATCAAGAGC??
?????????GTAAAACAGTTAACAGAGGCAGTGCAAAAAATATCCACAGAAAGCATAATAATATGGGAAAGA
CCCCTAAATTTARACTACCTATACAAAAGGAAACATGGGAAACATGGTGGGMGGRGTWTTGGCA?????????
???
???
??CTATTGA
TCTAGCTTTGCAGGATTCAGGGTTAGAAGTAAATATAGTAACAGACTCACAATATGCATTGGGAATCCTTCAA

GCACAACCAGACAAGAGTGAATCAGAGATAGTCAGTCATATAATAGAGCAGTTAATAAATAAGGAAAGGGTCT
ACCTGGCATGGG???
???
???AGAAATAGTAGCCTGCTGTGATAAATGTCAGC
TAAAAGGAGAGGCCATGCATGGACAAGTAGACTGTAGTCCAGGAATATGGCAACTAGATTGTACACATCTAGA
AGGAAAAATTATCATAGTAGCAGTTCATGTAGCCAGTGGATATATAGAGGCAGAAGTTATTCCAGCAGAGACA
GGGCAGGAAACAGCATACTTTCTCTTAAAATTAGCAGGGAGATGGCCAGTAAAAACAATACATACAGATAATG
GCCCCAATTTCATCAGTTCAGCGGTCAAGGCCGCCTGTTGGTGGGCAGGGATCAAGCAGGAATTTGGCATTCC
CTACAATCCCCAAAGTCAAGGAGTAGTAGAGTCTATGAATAAGGAATTAAAGACAATTATAGGACAGGTAAGA
G??
???
???
???
???
???
???CATTAGGGGATGCTAAATTGGTAATA
ATAACATATTGGGGTCTGCATACAGGAGAAAGAGACTGGCATTTGGGCCAGGGAGCCTCCATAGAGTGGAGGA
AAGAGAGATATAGCACACAAGTAGACCCTGGCCTAGCAGACCAACTAATTCATATGCACTATTTTGATTGTTT
TTCAGAATCTGCTATAAGAAATGCCATATTAGGACGTATAGTTAGTCCTAGGTGTGAATATCAAGCAGGACAT
AACAAGGTAGGATCTCTACAATATTTGGCACTAACAGCATTAATA????????????????????????????
???????????AAACTAACAGAGGATAGATGGAACAAGCCCCAGAGGACCAAGGACCACAGAGGGAGCCATAC
?????????GCACTAGGGATTTTAGAGGAACTTAAAAATGAAGCTGTTAGACATTTTCCTGGCCCTGGCTCC
AGGGCTTAGGACATATTTATGTAACTTATGGAAATACTTGGACAGGAGTGGAAGCCCTAATAAGAACTCTGCA
ACAACTGCTGTTTATTCATTTCAGAATTGGGTGTCGGCATAGCAGAATAGGCATTGACATCCGACAGAGGAGA
GCAAGAAATGGATCCAGTAGATCCTAGACTAGAGCCCTGGAAGCATCCAGGAAGTCAGCCTAGGACTGCTTGT
AATAGTTGCTATTGTAAAAAGTGTTGCCTTCATTGCCAAGTTTGCTTCTTAAGAAAAGGCTTAGGCATCTCCT
ATGGCAGGAAGAAGCGGAGACAGCGACGAAGAACTCCTTCAGAAAGTCAGACTCATCCGGCTTCTCTACCAAA
GCAGTAAGTTCTTTATCAAAGCAGTAAGTAGTATATGTAATGCAACCTGTACAAATATTAGCAATAGTAGCAT
TAGTAGTAGTAGCAATAATAGCAATAGTTGTATGGACCATAGTAGCCATAGAATATAGAAAAATATTAAGACA
AAGGAAAATAGACAGGTTAATTGAAAGAATAAGTGAAAGAGCAGAAGACAGTGGCAATGAGAGCGAAGGGGAT
CAGGAAGAGTTGTCAGCACTTGTGGAGATGGGGCATCATGCTCCTTGGGATATTGATGATCTGTAGTGCTGGA
GAACAATTGTGGGTCACAGTCTATTATGGGGTACCTGTGTGGAAAGAAGCAACTACCACTCTATTTTGTGCCT
CAGATGCTAAAGCATATGATACAGAAAGTCATAATGTTTGGGCCACACATGCCTGTGTACCCACAGACCCCAA
CCCACAAGAAGTAAGATTGAAAAATGTGACAGAAGAATTTAACATGGGAAAAAATAATATGGTGGACCAAATG
CATGAGGATATAATCAGTTTATGGGATCAAAGCTTAAAGCCATGTGTAAAATTAACCCCACTTTGTGTTACTT
TACATTGCACTGAGCCGACGAAGAGGAATAATACTAACAGCACTAATGCTCAGAGCAACATTATTATAGTGAA
TAACACAGACATAAAAAACTGTTCTTTCAATGTCACCACAGACATAAGAGACAGAATGCAGAAAGAATTTGCA
CTTTTTCACAAGCTTGATATAGTAC??
??ACA
TTATTGTGCCCCTGCTGGTTTTGCGATTCTAAAGTGTAACGAGGAAAAGTTCAATGRAACAGGACTATGCAGC
GATGTCAGCACAGTACAATGTACACATGGAATTAAACCAGTAGTGTCAACTCAACTGCTGTTAAATGGCAGCC
TAGCAAAAAAAAGAGGTAGTACTTAGGTCTGAAAATTTCACAGAAAATACTAAAACCATAATAGTGCAGCTG??
???
???????????????????????CATAATGGGAGATATAAGAAAAGCATATTGTATCATTAATAGCACACAAT
GGAATAACACTTTACAACAGGTAGTTAAAAAATTAAGAGAACAATTTGGGAATAAAACAATAGTCTTTAATCA
ATCCTCAGGAGGGGACCCAGAAGTTGTAAGGTATAGTTTTAATTGTGGAGGGGAATTTTTCTACTGTGATTCC
TCACAACTGTTTAATAGTACTTGGAATATTAGTGATATTAATAGTAATATTACTGGGAATAAGCCAA??????
????????????????????????????????ATAATAAACATGTGGCAGGAAGTAGGAAAAGCAATGTATGC
CCCTCCCATCAGTGGACAAATTAACTGTTCATCAAATATTACAGGGCTGCTATTAACAAGAGACGGTGGTAAG
GACGGTGATAATAGTACAGAGACTTTCAGACCAGGGGGGGGAAATATGAAGGACAATTGGAGAAGTGAATTAT
ATAAATACAAAGTAGTAAAAATTGAACCATTAGGAATAGCACCCACCGAGGCAAAGAGAAGAGTGGTGCAGAG
AGAAAAAAGAGCAATAGGATTAGGAGCTTTGTTCCTTGGGTTCTTGGGAGCAGCAGGAAGCACTATGGGCGCA
GCGTCAATGACGCTGACGGTACAGGCCAGACAATTATTGTCTGGTATAGTGCAACAGCAGAACAATCTGCTGA
AGGCTATTGAGGCGCAACAGCATCTGTTGCAACTCACAGTCTGGGGCATCAAGCAGCTCCAGGCAAGAGTCCT

GGCTGTGGAAAGATACCTAAGAGATCAACAGCTCCTAGGGATTTGGGGTTGCTCTGGAAAACTCATCTGCACC
ACTAATGTGCCTTGGAATACTAGTTGGAGTAATAAGACTAAGGATGAGATTTGGAATAACATGACCTGGATGC
AGTGGGAAAAAGAAATTGACAATTACACAGGCTTAATATACACTTTGCTTGAAGAATCACAGAACCAGCAGGA
AAAGAATGAACAAGAATTATTGGCATTAGATAAGTGGGCAAGTTTGTGGACTTGGCTTGACATAACAAACTGG
CTGTGGTATATAAAAATATTCATAATGATAGTAGGAGGCTTGATAGGTTTAAGAATAGTTTTTGCTGTACTTT
CCATAATAAATAGAGTTAGGCAGGGATACTCACCATTATCGTTACAGACCCACCTCCCAGCTCCGAGGGGACT
CGACAGGCCCGAAGGAATCGAAGAAGAAGGTGGAGAGACAGACAGAGGCAGATCAATTCGATTAGTGGATGGC
TTCTTAGCACTTTTCTGGGACGACCTGAGGAACCTGTGCCTCTTCAGCTACCACCGCTTGAGAGACTTACTCT
TGATTGTAACGAGGATTGTGGGACTTCTGGGACGCAGGGGGTGGGAACTCCTGAAATATTGGTGGAATCTCCT
GCAGTATTGGATTCAGGAACTAAAGAATAGTGCTACTAGCTTGCTGAACGCCACAGCTATAGCAGTTGCTGAG
GGGACAGATAGGATTATAGAAGTAGTACAAAGAGTTCTTAGAGCTATCCTTCACATACCTAGAAGAATAAGAC
AGGGCTTCGAAAGGGCTTTGCTATAAAGGCTTTGCTATAAAATGGGTAGCAAGTGGTCAAAGATGATTGGATG
GCCTGCTGTAAGGGAAAGAATGCAACGAGCTGAGCCAGCAGCAGAAGGGGTAGGAGCAGCATCTCGAGACCTG
GAGAGACATGGGGCACTCACAAGTAGCAATACAGCAGCTACTAATGCTGATTGTGCCTGGCTAGAAGCACAAG
AGGATGAGGAGGTGGGCTTTCCAGTCAGACCCCAGGTACCTTTAAGACCAATGACTTACAAGGGAGCGGTAGA
TCTCAGCCACTTTTTAAGAGAAAAGGGGGGACTGGAAGGGCTAATTTACTCCCAGAAGAGACAAGATATCCTA
GATCTGTGGGTCTATCACACACAAGGCTACTTCCCTGATTGGCAAAACTACACACCAGGGCCAGGGGTCAGAT
ATCCACTGACCTTTGGATGGTGCTTCAAGCTAGTACCAGTTGATCCAGACCAGGTAGAGAAGGCCAATGAAGG
GGAGAACAACAGCTTGCTGCACCCTATGAGCCTGCATGGGATAGAGGACCCGGAGAAAGAAGTGCTGATGTGG
AAGTTTGACAGCCGCCTAGCATTCAGTCACATGGCCCGAGAGCTGCATCCGGAGTACTACAAGAACTGATGAC
ACCAAGTTTCTACAAGGGACTTTCCACTGGGGACTTTCCAGGGGAGGCGCGGCCTGGGCGGGACTGGGGAGTG
GCGAGCCCTCAGATGCTGCATATAAGCAGCTGCTTTTTGCCTGTACTGGGTCTCTCTTGTTAGACCAGATCTG
AGCCTGGGAGCTCTCTGGCTAACTAGGGAACCCACTGCTTAAGCCTCAATAAAGCTTGCCTTGAGTGCTTTAA
GTAGTGTGTGCCCGTCTGTTGTGTGACTCTGGTAACTAGAGATCCCTCAGACCCTTTAGTCAGTGTGGAAAAT
CTCTAG?

>K-B6 HIV-1 genome, derived from RNA genomic sequence
CCACTGACCTTTGGATGGTGCTTCAAGCTAGTACCAGTTGATCCAGACCAGGTAG?????????????ACTAC
AAGAACTGATGACACCGAGTTTCTACAAGGGACTTTCCGCTGGGGACTTTCCAGGGRGGCGTGGCCTGGGCGG
GACTGGGGAGTGGCGAGCCCTCAGATGCTGCATATAAGCAGCTGCTTTTTGCCTGTACTGGGTCTCTCTGGTT
AGACCAGATCTGAGCCTGGGAGCTCTCTGGCTAACTAGGGAACCCACTGCTTAAGCCTCAATAAAGCTTGCCT
TGAGTGCTTCAAGTAGTGTGTGCCCGTCTGTTGTGTGACTCTGGTAACTAGAGATCCCTCAGACCCTTTTAGT
CAGTGTGGAAAATMTCTAGCAGTGGCGCCCGAACAGGGACGCGAAAGCGAAAGTAAGACCGGAGGAGCTCTCT
CGACGCAGGACTCGGCTTGCTGAAGCGCGCACGGCAMGAGGCGAGGGGCGGCGACTGGYGAGTACGCCAAAAT
TTTTGACTAGCGGAGGCTAGAAGGAGAGAGATGGGTGCGAGAGCGTC?GTATTAAGCGGGGGAGAATTAGATA
AATGGGAAAAAATTCGGTTAAGGCCAGGGGGAAAGAAAAGGTATAAGCTAAAACATATAGTATGGGCAAGCAG
GGAGCTAGAACGATTCGCAGTCAACCCTGGCCTGTTAGAAACATCAGAAGGCTGTAGACAAATACTGGGACAG
CTACAACCGTCCCTTCAGACAGGATCAGAAGAACTTAGATCATTATTTAATAC?????????CCTCTATTGTG
TGCATCAAAGGATAGATGTAAAAGACACCAAGGAAGCCTTAGATAAGATAGAGGAAGAGCAAAACAAAAGTAA
GAAAAAGGCACAGCAAGCA?????????GCAGCTGACACAGGAAACAACAKCCAGGTCAGCCAAAATTACCC?
???
???????????????CTTTAGCCCAGAAGTAATACCCATGTTTGCAGCATTATCAGAAGGAGCCACCCCACA
AGATTTAAACACCATGCTAAACACAGTGGGAGGACATCAAGCAGCTATGCAAATGTTAAAAGAGACCATCAAT
GAGGAAGCTGCAGAATGGGATAGATTGCATCCAGTGCATGCAGGGCCTGCTGCACCAGGCCAGATGAGAGAAC
CAAGGGGAAGTGACATARCAGGAACTACTAGTACCCTTCAAGAACAARTAGGATGGATGACACATAATCCACC
TATCCCAGTAGGAGAAATHTATAAAAGATGGATAATCCTGGGATTAAATAAAATAGTAAGAATGTATAGCCCT
ACCAGCATTCTGGACATAAGACAAGGACCAAAGGAACCCTTTAGAGATTATGTAGACCGGTTCTATAAAACTC
TAAGAGCTGAGCAAGCTTCACAGGAGGTAAAAAATTGGATGACAGAGACCTTATTGGTCCAAAATGCAAACCC
AGATTGTAAGACTATCTTAAAAGCATTAGGACCAGCAGCAACACTAGAAGAAATGATGACAGCATGTCAGGGA
GTAGGGGGACCCGGCCATAAAGCAAGAGTTTTGGCCGAGGCAATGAGCCAGGTAACAAATTCAGCTACCGTAA
TGATGCAGAAAGGCAACTTTAGGAACCAAAGAAAGGTTGTCAAGTGTTTCAATTGTGGCAAAGAAGGGCACAT
AGCCAGAAATTGCAGGGCCCCTAGGAAAAAGGGCTGTCGGAAATGTGGAAAGGAAGGACACCAAATGAARRAT
TGTRYTGAGAGACAGGCTAATTTTTTAGGGAARATCTGGCCTTCCCACAAGGGAAGGCCAGGGAATTTYCTTC
AGAGCAGACCAGAGCCAACAGCCCCACCAGAAGAGAGCCTCAGGTTTGGGGAGGAGACAACAACTCCCTATCA
GAAAC??
???
??????????????GGAAGATGGAAACCAAAAATGATAGGGGGAATTGGAGGTTTTATCAAGTAAGACAATAT
GATCAGGTACCCATAGAAATCTGTGGGCATAAAGCTATAGGTACAGTWTYARTAGGACCTACACCTGTCAACA
TAATTGRAAGAAATCTGTTGACTCAACTTGGTTGCACTTTAAATTTTCCCATTAGTCCTATTGAAACTGTACC
AGTAAAATTAAAGCCAGGAATGGATGGCCCAAAAGTTAAACAGTGGCCATTGACAGAGGAAAAAATAAAAGCA
TTAGTAGAAATTTGTACAGAAATGGAAAAGGAAGGAAAAATTTCAAAAGTTGGGCCTGAAAATCCATACAATA
CTCCAGTATTTGCCATAAAGAAAAAGGATGGTACTAAATGGAGAAAACTAGTAGATTTCAGAGAACTTAATAA
GAGAACTCAAGACTTCTGGGAAGTTCAATTAGGAATACCACATCCTGCAGGGTTAAAAAAGAAAAAATCAGTA
ACAGTACTGGATGTAGGTGATGCTT?????????????????????????CTTCAGGAAGTATACTGCATTTA
CCATACCCAGCATAAATAATGAGACACCAGGGATTAGATATCAGTACAATGTACTTCCGCAGGGATGGAAAGG
ATCACCAGCAATATTCCAAAGTAGCATGACAAAAATCTTAGATCCTTTTAGAAAGCAAAATCCAGACATAGTT
ATTTACCAGTACATGGATGATTTGTATGTAGGATCTGACTTAGAAATAGGGCAGCACAGAATAAAAATAGAGG
AACTAAGACAACATCTGTTGAAGTGGGGATTCACCACACCAGACAAGAAACATCAGAAAGAACCTCCATTCCT
TTGGATGGGTTATKAMCTTCATCCTGATAAATGGACAGTACAGCCT???????????????????????????
???
???
???
??TCTGA
AAACAGGAAAGTATGCAAGAATGAGGAGTACCCACACTAATGATGTAAAACAGTTAACAGAGGCAGTGCAAAA
AATATCCACAGAAAGCATAGTAATATGGGGAAAGACCCCTAAATTT???????????????????????????
???CCCCTCCCTTAG
TGAARYTATGGTACCAGTTAGARAAAGAACCCATARTAGGAGCAGAAACTTTCTATGTAGATGGGGCAGCYAA
TAGRGAAACTAAATTAGGAAAAGCAGGMTATGTWACTGACAGAGGWAGACAAAAAGTTGTCYCYCTAACTGAC
ACAACAAATCAGAAAACTGAGTTACAAGCTATTGCTCTAGCTTTGCAGGATTCRGGRTTAGAAG?????????
????????????????????????????CATTCAAGCACAACCAGATAAGAGTGAATCAGAGATAGTCAGTCA

AATAATAGAGCAGTTAATAAAAAAGGAAAAAGTCTACCTGGCATGGGTACCAGCACACAAAGGAATTGGRGGA
AATGAACAAGTAGAYAAATTGGTCAGTACTGGAATCAGAAGAGTACTATTCCTAGATGGAATAGATAAGGCCC
AAGAGGAGCATGAAAGATATCACAGTAATTGGAGAGCAATGGCTAGTGATTTTAACCTACCACCTGTAGTAGC
AAAAGAAATAGTAGCCTGCTGTGATAAATGTCAGCTAAAAGGAGAAGCCATGCATGGACAAGTAGACTGTAGT
CCAGGAATATGGCAACTARATTGTACACATCTAGAAGGAAAAATWATCATAGTAGCAGTTCATGTAGCCAGTG
GATATATAGAGGCAGAAGTT??
??
??ATGA
ATAAGGAATTAAAGACAATTATAGGACAGGTAAGAGATCAGGCTGAACATCTTAAGACAGCAATACAAATGGC
AGTATTCATCCACAATTTTAAGAAAAAAGGGGGGATTGGGGGGTACAGTGCAGGGGAAAGAATAATAGACATA
ATAGCAWCAGACATACARACTAAAGAAYTACAAAAACAAATTACAAAAATTCAAAATTTTCGGGTCTATTACA
GGGACAGCAGAGATCCACTTTGGAAAGGACCAGCAAAGCTCCTTTGGAAAGGTGAAGGGGCAGTAGTAATACA
AGATAATAGTGATATAAAAGTAGTGCCAAGAAGAAAAGCAAAGATCATTAGAGATTATGGAAAACAGATGGCA
GGTGATAATTGTGTGGCAGGTGGACAGGATGAGGATTAGAACATGGAAAAGTTTAGTAAAACACCACATGTAT
ATTTCAAAGAAAGCTAAGGGATGGATTTATAAACATCACTATGAAAGCACTCATCCAAGAATAAGTTCAGAAG
TACACATCCCATTAGGGGATGCTAAATTGGTAATAATAACATATTGGGGTCTGCATACAGGAGAAAGAGACTG
GCATTTGGGCCAGGGAGCCTCCATAGAATGGAGGATAAAGAGATATAAAACACAAGTAAACCCTGGCCTAGCA
GACCAACTAATTCATATGCACTATTTTGAT?????????????????????????CCATATTAGGACGTA
TAGTTAGTCCTAGGTGTGAATATCAAGCAGGACATAACAAGGTAGGATCTCTACAATATTTGGCACTAACAGC
ATTAATAAAACCAAAAGGGAGAAAGCCACCTT??????????????????????????????????????
????????????????????????????ATACAATGAATGGACACTAGAGCTTTTAGAGGAACTTAAGAG
TGAAGCTGTTAGACATTTTCCTAGGATATGGCTCCATAACTTAGGACAACATATCTATGAAACTTACGGGGAT
ACTTGGGCAGGAGTGGAAGCCATAATAAGAATTCTGCAACAACTGCTGTTTATCCATTTCAGAATTGGGTGTC
GACATAGCAGAATAGGCATTGACATCCGACAGAGGAGAGCAAGAAATGGATCCAGTAGATCCTAGATTAGAGC
CCTGGAAGCATCCAGGAAGTCAGCCTAGGACTGCTTGTAATAGTTGCTATTGTAAAAAGTGTTGCCTTCATTG
CCAAGTTTGCTTCTTAAGAAAAGGCTTAGGCATCTC??????????????????????????????????
??
??????CAATAGTAGCATTAGTAGTAGTAGCAATAATAGCAATAGTTGTATGGACCATAGTAGCCATAGAATA
TAGAAAAATATTAAGACAAAGAAAAATAGACAGGTTAATTGAAAGAATAAGTGAAAGAGCAGAAGACAGTGGC
AATGAGAGCGAGGGGGATCAGGAAGAGTTGTCAGCACTTGTGGAGATGGGGCATCATGCTCCTTGGGATATTG
ATGATCTGTAGTGCTGGAGAACAATTGTGGGTCACAGTCTATTATGGGGTACCTGTGTGGAAAGAAGCAACTA
CCACTCTATTTTGTGCCTCAGATGCTAAAGCATATGATACAGAAAGTCATAATGTTTGGGCCACACATGCCTG
TGTACCCACAGACCCCAACCCACAAGAAGTAAGATTGAAAAATGTGACAGAAGAATTTAACATGGGAAAAAAT
??
??
??
??
??ATTGTGCCCC
TGCTGGTTTTGCGATTCTAAAGTGTAACGAGGAAAAGTTCAATGGAACAGGACTATGCAGCGATGTCAGCACA
GTACAATGTACACATGGAATTAAACCAGTAGTGTCAACTCAACTGCTGTTAAATGGCAGCCTAGCAAAAGAAG
AGGTAGTACTTAGGTCTGAAAATTTCACAGAAAATACTAAAACCATAATAGTACAGCTGAACACATCTGTAGA
AATTAATTGTACAAGACCCAACAACAATACAAGAAAAAGTATACGTATACGTATAGGACCAGGGGGCGCATCA
TTTTATGCAACAAACATAATGGGAGATATAAGAAAAGCATATTGTATCATTAATAGCACACAATGGAATAACA
CTTTACAACAAGTAGTTAAAAAATTAAGAGAACAATTTGGGAATAAAACAATAGTCTTTAATCA?????????
?????ACCCAGAAGTTGTAAGGTATAGTTTTAATTGTGGAGGGGAATTTTTCTACTGTGATTCCTCACAACTG
TTTAATAGTACTTGGAATATTAGTGATATTAATAGTAATATTACTGGGAATAAGACAAATATCACACTCCCAT
GTAGAATAAAACAAATAATAAACATGTGGCAGGAAGTAGGAAAAGCAATGTATGCCCCTCCCATCAGTGGACA
AATTAGATGTTCATCAAATATTACTGGGCTGCTATTAACAAGAGAYGGTGGTAATAACRRYRATRRTGAAAAC
AGTACAGAGATTTTCAGACCGGGAGGAGGAAATATGAAGGACAATTGGAGAAGTGAATTATAT??ATACAAAG
TAGTAAAAATTGAACCATTAGGAATAGCACCCACCGAGGCAAAGAGAAGAGTGGTGCAGAGAGAAAAAAGAGC
AATAGGATTAGGAGCTTTGTTCCTTGGGTTCTTGGGAGCAGCAGGAAGCACTATGGGCGCAGCGTCAATGACG
CTGACGGTACAGGCCAGACAATTATTGTCTGGTAT???????????????????????????????????
???????????????GCGCCTCACAGTCTGGGGCATCAAACAGCTCCAGGCAAGAATCCTGGCTGTGGAAAG
ATACCTAAAGGATCAACAGCTCCTGGGGATTTGGGGTTGCTCTGGAAAACTCATCTGCACCACTAACGTGCCT

TGGAATACTAGTTGGAGTAATAAGACTAAGGATRAGATTTGGAATAACATGACCTGGATGCAGTGGAAAAAG
AGATTGACAATTACACAGGCTTAATATACACTTTGCTTGAAGAATCACAGAACCAGCAGGAAAAGAATGAACA
AGAATTATTGGCATTGGATAAGTGGGCAAGTTTGTGGACTTGGCTTGACATAACAAACTGGCTGTGGTATATA
AAAATATTCATAATGATAGTAGGAGGCTTGATAGGTTTAAGAATAGTTTTTGCTGTACTTTCTATAATAAATA
GAGTTAGGCAGGGATACTCACCATTATCGTTGCAGACCCACCTCCCAGCTCCGAGGGGACTCGACAGGCCCGA
AGGAATCGAAGAAGAAGGTGGAGAGACAGACAGAGGCAGATCAATTCGATTAGTGGATGGCTTCTTRGCACTT
TTCTGGGACGACCTGAGGAACCTGTGCCTCTTCAGCTACCACCGCTTGAGAGACTTACTCTTGATTGTAACGA
GGATTGTGGGACTTCTGGGACGCAGGGGGTGGGAACTCCTGAAATATTGGTGGAATCTCCTGCAGTATTGGAT
TCAGGAACTAAAGAATAGTGCTACTAGCTTGCTGAACGCCACAGCTATAGCAGTTGCTGAGGGGACAGATAGG
ATTATAGAAGTAGTACAAAGAATTCTTAGAGCTATCCTTCACATACCTAGAAGAATAAGACAGGGCTTCGAAA
GGGCTTTGCTATAAAATGGGTGGCAAGTGGTCAAAGATGATTGGATGGCCTGCTGTAAGGGAAAGAATGCATC
GAGCTGAGCCAGCAGCAGAAGGGGTAGGAGCAGCATCTCGAGATCTGGAGAGACATGGAGCGCTTACAAGTAG
CAATACAGCAGCTACTAATGCTGACTGTGCCTGGCTAGAAGCACAAGAGGATGAGGAGGTGGGCTTTCCAGTC
AGACCCCAGGTACCTTTAAGACCAATGACTTACAAGGGAGCGGTAGATCTCAGCCACTTTTTAAGAGAAAAGG
GGGGACTGGAAGGGCTAATTTACTCCCAGAAGAGACAAGATATCCTAGATCTGTGGGTCTATCACACACAAGG
CTACTTCCCTGATTGGCAAAACTACACACCAGGGCCAGGGGTCAGATATCCACTGACCTTTGGATGGTGCTTC
AAGCTAGTACCAGTTGATCCAGACCAGGTAGAGAAGGCCAATGAAGGGGAGAACAACAGCTTGCTGCACCCTA
TGAGCCTGCATGGGATAGAGGACCCGGAGAAAGAAGTGCTGATGTGGAAGTTTGACAGCCGCCTAGCATTCAG
TCACATGGCCCGAAAGCTGCATCCGGAGTACTACAAGAACTGATGACACCGAGTTTCTACAAGGGACTTTCCG
CTGGGGACTTTCCAGGG???????????????GGACTGGGGAGTGGCGAGCCCTCAGATGCTGCATATAAGC
AGCTGCTTTTTGCCTGTACTGGGTCTCTCTTGTTAGACCAGATCTGAGCCTGGGAGCTCTCTGGCTAACTAGG
GAACCCACTGCTTAAGCCTCAATAAAGCTTGCCTTGAGTGCTTTAAGTAGTGTGTGCCCGTCTGTKGTGTGAC
TC??

>K-C1 HIV-1 genome, derived from RNA genomic sequence
CCTATGAGCCTGCATGGGATAGAGGACCCGGAGAAAGAAGTGCTGATGTGGAAGTTTGACAGCCGCCTAGCAT
TCAGTCACATGGCCCGAGAGCTGCATCCGGAGTACTACAAGAACTGATGACACCGAGTTTCTACAAGGGACTT
TCCGCTGGGGACTTTCCAGGGGAGGCGCGGCCTGGGCGGGACTGGGGAGTGGCGAGCCCTCAGATGCTGCATA
TAAGCAGCTGCTTTTTGCCTGTACTGGGTCTCTCTGGTTAGACCAGATCTGAGCCTGGGAGCTCTCTGGCTAA
CTAGGGAACCCACTGCTTAAGCCTCAATAAAGCTTGCCTTGAGTGCTTTAAGTAGTGTGTGCCCGTCTGTTGT
GTGACTCTGGTAACTAGAGATCCCTCAGACCCTTTAGTCAGTGTGGAAAATCTCTAGC???????????????
???
???
???
???????????????????ATATAGTATGGGCAAGCAGGGAGCTAGAACGATTCGCAGTCAACCCTGGCCTGT
TAGAAACATCAGAAGGCTGTAGACAAATACTGGGACAGCTACAACCGTCCCTTCAGACAGGATCAGAAGAACT
TAGATCATTATTTAATACAGTAGCAGTCCTCTATTGTGTACACCAAAGGATAGAGGTAAAAGATACCAAAGAA
GCTTTAGAGAAGGTAGAGGAAGAGCAAAACAAAAGTAAGAAAAAGGCACAGCAAGCAGCAGCAGCTGCGGCTG
ACACAGGAAATGGCGGCAAGGTCAGCCAAAATTTTCCTATAGTGCAGAACCTACAGGGGCAAATGGTACATCA
GCCCCTATCACCTAGAACTTTAAATGCATGGGTAAAAGTAGTAGAAGAAAAAGCTTTCAGCCCAGAAGTAATA
CCCATGTTTGCAGCATTATCAGAAGGAGCCACCCCACAAGATTTAAACACCATGCTAAACACAGTGGGAGGAC
ATYAAGCAGCTATGCAAATGTTAAAAGAGACCATCAATGAGGAAGCTGCAGAATGGGATAGATTGCATCCAGT
GCATGCAGGGCCTGCTGCACCAGGCCAGATGAGAGAACCAAGGGGAAGTGACATAGCAGGAACTACTAGTACC
CTTCAGGAACAAATAGGCTGGATGACACATAATCCACCTATC???????????????????????????????
???
?CCCTTTAGAGATTATGTAGACCGGTTCTATAAAACTCTAAGAGCTGAGCAAGCTTCACAGGAGGTAAAAAAT
TGGATGACAGAGACCTTATTGGTCCAAAATGCAAACCCAGATTGTAAGACTATCTTAAAAGCATTAGGACCAG
CAGCAACACTAGAAGAAATGATGACAGCATGTCAGGGAGTAGGGGGACCCGGCCATAAAGCAAGAGTTTTGGC
CGAGGCAATGAGCCAGGTAACAAATTCAGCTAYCGTAATGATGCAGAAATGTAACTTTAGGAACCAAAGAAAG
GTTGTCAAGTGTTTCAATTGTGGCAAAGAAGGGCACATAGCCAGAAATTGCAGGGCCCCTAGAAAAAAGGGCT
GTTGGAAATGTGGAAAGGAAGGACACCAAATGAAAGATTGTGTTGAGAGACAGGCTAATTTTTTAGGGAAAAT
CTGGCCTTCCCACAAGGGAAGGCCAGGGAATTTCCTTCAGAGCAGACCAGAGCCAACAGCCCCACTAGAGCCA
ACAGCCCCACCAGAGGAGAGCCTCAGGTTTGGGGAGGAGACAACAACTCCCTATCAGAAACAGGAGCAGATGG
ACAAGGAGAAGTATCCTTTGACTTCCCTCAGATCACTCTTTGGCAACGACCCCTTGTCACAATAAAAGTAGGG
GGGCAGTTAAAGGAAGCTCTATTAGATACAGGAGCAGATGATACAGTATTAGAAGAAATGAATTTGCCAGGAA
GATGGAAACCAAAAATGATAGGGGGAATTGGAGGTTTTATCAAAGTAAGACAGTATGATCAGATAGCCATAGA
AATCTGTGGGCATAAAGCTATAGGTACAGTATTAGTAGGACCTACACCTGTCAACATAATTGGAAGAAATCTG
TTGACTCAGCTTGGTTGCACTTTAAATTTTCCCATTAGTCCTATTGAAACTGTACCAGTAAAATTAAAGCCAG
RAATGGATGGCCCAAAAGTTAAWCAGTGGCCATTGACAGAGGAAAAAATAAAAGCATTAGTAGAAATTTGTAC
AGAAATGGAAAAGGAAGGAAAAATTTCAAAAGTTGGGCCAGAAAATCCATACAACACTCCAGTATTTGCCATA
AAGAAAAAAGATGGTACTAAATGGAGAAAATTAGTAGATTTCAGAGAACTTAATAAGAGAACTCAAGACTTCT
GGGAAGTTCAATTAGGAATACCACACCCTGCAGGT???????????????????TAACAGTACTGGATGTAGG
TGACGCTTATTTTTCAGTTCCCTTAGATAAAGACTTCAGGAAGTATACTGCATTTACCATACCCAGTATAAAT
AATGAGAC???
???
??ATCTG
TTGAAGTGGGGGTTCACCACACMAGACAAGAAACATCAGAAAGAACCTCCATTCCTTTGGATGGGTTATGAAC
TCCATCCTGATAAATGGACAGTACAGCCTATAGAGCTGCCAGAAAAAGACAGCTGGACTGTCAATGACATACA
GAAGTTAGTGGGAAAATTAAATTGGGCAAGTCAAATTTATGCAGGGATTAAAGTAAGGCAATTATGTAAACTC
CTTAGGGGAGCCAAAGCACTAACAGAAGTAATAACACTAACAGCAGAAGCAGAACTAGAGCTGGCAGAAAACA
GGGAGATTCTAAAAACACCAGTGCATGGAGTGTACTATGACCCATCAAAAGACTTAGTAGCAGAAATACAGAA
GCAGGGGCAAGGTCAATGGACATATCAAATTTATCAAGAGCCATTTAAGAATCTGAAAACAGGAAAGTATGCA
AGAATGAGGAGTACCCACACTAATGATGTAAAACAGTTAACAGAGGCAGTGCAAAAAATATCCACAGAAAGCA
TAGTAATATGGGGAAAGACCCCTAAATTTAGACTACCTATACAAAAGGAAACATGGGAAACATGGTGGGCGGA
GTATTGGCAAGCCACCTGGATTCCTGAGTGGGAATTTGTCAATACCCCTCCCTTAGTGAAACTATGGTACCAG
TTAGAAAAAGAACCCATAGCAGGAGCAGAAACTTTCTATGTAGATGGGGCAGCCAATAGAGAAACTAAATTAG
GAAAAGCAGGATATGTTACTGACAGAGGAAGACAAAAAGTTGTCTCCCTAACTGACACAACAAATCAGAAAAC
TGAGTTACAAGCTATTGCTCTAGCTTTGCAGGATTCAGGGTTAGAAGTAAATATAGTAACAGACTCACAATAT

GCACTGGGAATCATTCAAGCACAGCCAGATAAGAGTGAATCAGAGATAGTCAGTCATATAATAGAGCAGTTAA
TAAATAAGGAAAGGGTCTACCTGGCATGGGTACCAGCACACAAAGGAATTGGGGGAAATGAACAAGTAGACAA
ATTGGTCAGTACTGGAATCAGAAGAGTACTATTCCTAG???????????????????????????????????
??
??CAAGTAGACTGTAGTCCAGGAATATGGCAACT
AGATTGTACACATCTAGAAGGAAAAATTATCATAGTAGCAGTTCATGTAGCCAGTGGATATATAGAGGCAGAA
GTTATTCCAGCAGAGACAGGGCAGGAAACAGCATACTTTCTCTTAAAATTAGCAGGGAGATGGCCAGTAAAAA
CAATACATACAGATAATGGCCCCAATTTCATTAGCTCCGCGGTCAAGGCCGCCTGTTGGTGGGCAGGGATCAA
GCAGGAATTTGGCATTCCCTACAATCCCCAAAGTCAAGGAGTAGTAGAGTCTATGAATAAGGAATTAAAGACA
ATTATAGGACAGGTAAGAGATCAGGCTGAACATCTTAAGACAGCACTACAAATGGCAGTATTCATCCACAATT
TTAAGAAAAAAGGGGGGATTGGGGGGTACAGTGCAGGGGAAAGAATAATAGACATAATAGCATCAGACATACA
GACTAAAGAACTACAAAAACAAATTACAAAAATTCAAAATTTTCGGGTTTATTACAGGGACAGCAGAGATCCA
CTTTGGAAAGGACCAGCAAAGCTCCTTTGGAAAGGTGAAGGGGCAGTAGTAATACAAGATAATAGTGATATAA
AAGTAGTGCCAAGAAGAAAAGCAAAGATCATTAGAGATTATGGAAAACAGATGGCAGGTGATGATTGTGTGGC
AGGTGGACAGGATGAGGATTAGAACATGGAAAAGTTTAGTAAAACACCACATGTATATTTCAAAGAAAGCTAA
GGGATGGATTTATAAACATCACTATGAAAGCACTCATCCAAGAATAAGTTCAGAAGTACACATCCCATTAGGG
GATGCTAAATTGGTAATAATAACATATTGGGGTCTGCATACAGGAGAAAGAGACTGGCATTTGGGCCAGGGAG
CCTCCATAGAATGGAGGATAAAGAGATATAGAACACAAGTAGACCCTGGCCTAGCRGACCAACTAATTCWTAT
RSMMKATTYTGATTGTTTTTCAGAATCTGCTATAAGAAATGCCATATTAGGACGTATAGTTAGTCCTAGGTGT
GAATATCAAGCAGGACATAACAAGGTAGGATCTCTACAATATTTGGCACTAACAGCATTAATAAAACCAAAAG
GGAGAAAGCCACCTTTGCCTAGTGTAGCGAAACTGACAGAGGATAGATGGAACAAGCCCCAGAG?????????
?????????????????????????????????????TTTTAGAGGAACTTAAAAATGAAGCTGTTAGACATT
TTCCTGGGCCCTGGCTCCAGGGCTTAGGACAATATATTTATGTAACCTATGGAAATACTTGGACAGGAGTGGA
AGCCCTAATAAGAACTCTGCAACAACTGCTGTTTATTCATTTCAGAATTGGGTGTCGACATAGCAGAATAGGC
ATTGACATCCGACAGAGGAGAGCAAGAAATGGATCCAGTAGATCCTAGACTAGAGCCCTGGAAGCATCCAGGA
AGTCAGCCTAGGACTGCTTGTAATAGTTGCTATTGTAAAAAGTGTTGCCTTCATTGCCAAGTTTGCTTCTTAA
GAAAAGGCTTAGGCATCTCCTATGGCAGGAAGAAGCGGAGACAGCGACGAAGAACTCCTTCAGAAAGTCAGAC
TCATCCGGCTTCTCTACCAAAGCAGTAAGTTCTTTATCAAAGCAGTAAGTAGTATATGTAATGCAACCTGTAC
AAATATTAGCAATAGTAGCATTAGTAGTGGTAGCAATAATAGCAATAGTTGTATGG??????????????AGA
ATATAGAAAAATATTAAGACAAAGAMAAATAGACAGGKTAATTGAAAGAATAAGTGAAAGAGCAGAAGACAGT
GGCAATGAGAGCGAGGGGGATCAGGAAGAGTTGTCAGCACTTGTGGAGATGGGGCATCATGCTCCTTGGGATA
TTGATGATCTGTAGTGCTGGAGAACAATTGTGGGTCACAGTCTATTATGGGGTACCTGTGTGGAAAGAAGCAA
CTACCACTCTATTTTGTGCCTCAGATGCTAAAGCATATGATACAGAAAGTCATAATGTTTGGGCCACACATGC
CTGTGTACCCACAGACCCCAACCCACAAGAAGTAAGATTGAAAAATGTGACAGAAGAATTTAACATGGGAAAA
AATAATATGGTAGATCAAATGCATGAGGATATAATCAGCTTATGGGATCAAAGCTTAAAGCCATGTGTAAAAT
TAACCCCACTTTGTGTTACTTTACAT??
??
??
??
??
??????????????????????TAGTCCTGTTCCATGGAATTAAACCAGTAGTGTCAACTCAACTGCTGTTAA
ATGGCAGCCTAGCAAAAGAAGAGGTAGTACTTAGGTCTGAAAATTTCACAGAAAATACTAAAACCATAATAGT
GCAGCTGAAGGACCCTGTAGTAATTAATTGTACAAGACCCAACAACAATATAAGAAAAAGGATACGTATAGGA
CCAGGGGGCGCATCATTTTATGCAACAAACATAATGGGAGATATAAGAAAAGCATATTGTATCATTAATAGCA
CA??
??
??
????????????????????CTCCCATGTAGAATAAAACAAATAATAAACATGTGGCAGGAAGTAGGAAAAGC
AATGTATGCCCCTCCCATCAGTGGACTAATTAACTGTTCATCAAATATTACAGGGCTGCTATTAACAAGAGAC
GGTGGTAATGACAATAGTAATGAAAACAGTACAGAGACTTTCAGACCAGGAGGAGGAAATATGAAGGACAATT
GGAGAAGTGAATTATATAAATACAAAGTAGTAAAAATTGAACCATTAGGAATAGCACCCACCGAGGCAAAGAG
AAGAGTGGTGCAGAGAGAAAAAAGAGCAATAGGATTAGGAGCTTTGTTCCTTGGGTTCTTGGGAGCAGCAGGA
AGCACTATGGGCGCAGCGTCAATGACGCTGACGGTACAGGCCAGACAATTATTGTCTGGTATAGTGCAACAGC
AGAACAATCTGCTGAGGGCTATTGAGGCGCAACAGCATCTGTTGCAACTCACAGTCTGGGGCATCAAGCAGCT

CCAGGCAAGAGTCCTGGCTGTGGAAAGATACCTAAGAGATCAACAGCTCCTAGGGATTTGGGGTTGCTCTGGA
AAACTCATCTGCACCACTAATGTGCCTTGGAATACTAGTTGGAGTAATAAGACTAAGGATGAGATTTGGAATA
ACATGACCTGGATGCAGTGGGAAAAAGAAATTGACAATTACACAGGCTTAATATACACTTTGCTTGAAGAATC
ACAGAACCAGCAGGAAAAGAATGAACAAGAATTATTGGCATTGGATAAGTGGGCAAGTTTGTGGACTTGGCTT
GACATAACAAACTGGCTGTGGTATATAAAAATATTCATAATGATAGTAGGAGGCTTGATAGGTTTAAGAATAG
TTTTTGCTGTACTTTCTATAATAAATAGAGTTAGGCAGGGATACTCACCATTATCGTTGCAGACCCACCTCCC
AGCTCCGAGGGGACTCGACAGGCCCGAAGGAATCGAAGAAGAAGGTGGAGAGACAGACAGAGGCAGATCAATT
CGATTAGTGGATGGCTTCTTAGCACTTTTCTGGGACGACCTGAGGAACCTGTGCCTCTTCAGCTACCACCGCT
TGAGAGACTTACTCTTGATTGTAACGAGGATTGTGGGACTTCTGGGACGCAGGGGGTGGGAACTCCTGAAATA
TTGGTGGAATCTCCTGCAGTATTGGATTCAGGAACTAAAGAATAGTGCTACTAGCTTGCTGAACGCCACAGCT
ATAGCAGTTGCTGAGGGGACAGATAGGATTATAGAAGTAGTACAAAGAATTCTTAGAGCTATCCTTCACATAC
CTAGAAGAATAAGACAGGGCTTCGAAAGGGCTTTGCTATAAGGGCTTTGCTATAAAATGGGTGGCAAGTGGTC
AAAGATGATTGGATGGCCTGCTGTAAGGGAAAGAATGCAACGAGCTGAGCCAGCAGCAGAAGGGGTAGGAGCA
GCATCTCGAGATCTGGAGAGACATGGAGCACTTACAAGTAGCAATACAGCAGCTACTAATGCTGATTGTGCCT
GGCTAGAAGCACAAGAAGATGAGGAGGTGGGCTTTCCAGTCAGACCCCAGGTACCTTTAAGACCAATGACTTA
CAAGGGAGCGGTAGATCTTAGCCACTTTTTAAGAGAAAAGGGGGGACTGGAAGGGCTAATTTACTCCCAGAAG
AGACAAGATATCCTAGATCTGTGGGTCTACCACACACAAGGCTACTTCCCTGATTGGCAAAACTACACACCAG
GGCCAGGAGTCAGATATCCACTGACCTTTGGATGGTGCTTCAAGCTAGTACCAGTTGATCCAGACCAGGTAGA
GAAGGCCAATGAAGGAGAGAACAACAGCTTGCTGCATCCTATGAGCCTGCATGGGATAGAGGACCCGGAGAAA
GAAGTGCTGATGTGGAAGTTTGACAGCCGCCTAGCATTCAGTCACATGGCCCGAGAGCTGCATCCGGAGTACT
ACAAGAACTGATGACACCGAGTTTCTACAAGGGACTTTCCGCTGGGGACTTTCCAGGGGAGGCGCGGCCTGGG
CGGGACTGGGGAGTGGCGAGCCCTCAGATGCTGCATATAAGCAGCTGCTTTTTGCCTGTACTGGGTCTCTCTG
GTTAGACCAGATCTGAGCCTGGGAGCTCTCTGGCTAACTAGGGAACCCACTGCTTAAGCCTCAATAAAGCTTG
CCTTGAGTGCTTTAAGTAGTGTGTGCCCGTCTGTTGTGTGACTCTGGTAACTAGAGATCCCTCAGACCCTTTA
GTCAGTGTGGAAAATCTCTAGC

>K-C3 HIV-1 genome, derived from RNA genomic sequence
CCTATGAGCCAGCATGGGATGGAGGACCCGGAGGGAGAAGTATTAGTGTGGAAGTTTGACAGCCTCCTAGCAT
ACAGTCACATGGCCCGAGAGCTGCATCCGGAGTACTACAAGAACTGATGACACCGAGTTTCTACAAGGGACTT
TCCGCTGGGGACTTTCCAGGGGAGGCGCGGCCTGGGCGGGACTGGGGAGTGGCGAGCCCTCAGATGCTGCATA
TAAGCAGCTGCTTTTTGCCTGTACTGGGTCTCTCTGGTTAGACCAGATCTGAGCCTGGGAGCTCTCTGGCTAA
CTAGGGAACCCACTGCTTAAGCCTCAATAAAGCTTGCCTTGAGTGCTTTAAGTAGTGTGTGCCCGTCTGTTGT
GTGACTCTGGTAACTAGAGATCCCTCAGACCCTTTAGTCAGTGTGGAAAATCTCTAGCAGTGGCGCCCGAACA
GGGACGCGAAAGCGAAAGTAAGACCGGAGGAGCTCTCTCGACGCAGGACTCGGCTTGCTGAAGCGCGCACGGC
AAGAGG???
????????????????????????????GAATTAGATAAATGGGAAAAAATTCGGTTAAGGCCAGGGGGAAAG
AAAAGGTATAAGCTAAAACATATAGTATGGGCAAGCAGGGAGCTAGAACGATTCGCAGTCAACCCTGGCCTGT
TAGAAACATCAGAAGGCTGTAGACAAATACTGGGACAGCTACAACC???????????????????????????
?????????????????????????GTCCTCTATTGTGTACACCAAAGGATAGMGGTAAAAGATACCAAAGAA
GCTTTAGAGAAGGTAGAGGAAGAGCAAAACAAAAGTAAGAAAAAGGCACAGCAAGCAGCAGCAGCTGCAGCTG
ACACAGGAAACGACGGCAAGGTCAGCCAAAATTTCCCCATAGTGCAGAATCTCCAGGGGCAAATGGTACATCA
GSCCWTATCACCTAGAACTTTAAATGCATGGGTAAAAGTAGTAGAAGAGAAGGC???????????????????
?????????????????????????GAG??TTCCTGC????????????????????????????????????
???
???
????????????????????TATGTCACATAATCCACCTATCCCAGTAGGAGAAATTTATAAAAAATGGATAA
TCATGGGATTAAATAAAATAG??
??????????????????????????????????ACTCTAAGAGCCGAGCAAGCTTCACAAGAGGTAAAAAAT
TGGATGACAGAAACCTTGTTGGTCCAAAATGCGAACCCAGATTGTAAGAC???????????????????????
??????????????????????????????GTCAGGGAGTGGGGGGACCCGGCCATAAAGCAAGAGTTTTGGC
TGAAGCAATGAGCCAAGTAACAAATCCAGCTACCATAATGATACAGAAAGGCAATTTTAGGAACCAAAGAAAG
ACTGTTAAGTGTTTC??CT
GTTGGAAATGTGGAAAGGAAGGACACCAAATGAAGGATTGTGTTGAGAGACAGGCTAATTTTTTAGGGAAAAT
CTGGCCTTCCCACAAGGGAAGGCCAGGGAATTTCCTGCAGAGCAGACCAGAGCCAACAGCCCCACCAGAGGGG
AGCCTCAGGTTTGGGGAGGAGACAACAACTCCCTATCAGAAACAGGAGCAGATGGACAAGGAGAAGTATCCTT
TGACTTCCCTCAGATCACTCTTTGGCAACGACCCCTTGTCACAATAAAAGTA?????????????????????
??????????????????????ATACAGTATTAGAAGAAATGAATTTGCCAGGAAGATGGAAACCAAAAATGA
TAGGGGGAATTGGAGGTTTTATCAAAGTAAGACAGTATGATCAGATACTC???????????????????????
??????????GTATTAGTAGGACCTACACCTGTCAACATAATTGGAAGAAATCTGTKGACTCAGCTTGGTTGC
ACTTTAAATTTTCCCATCAGTCCTATTG???
???
??????????????????????????????????????CTCCAGTATTTGCCATAAAGAAAAAAGACAGTACT
AAATGGAGAAAATTAGTAGATTTCAGAGAACTTAATAAGAGAACTCAAGATTTCTGGGAAGTTCAATTAGGAA
TACCACATCCTGCAGGGTTAAAAC???
???????GATAAAGACTTCAGGAAGTATACTGCATTTACCATACCCAGTATAAATAATGAGACACCAGGGATT
AGATATCAGTACAATGTACTTCCGCAGGGATGGAAAGGATCACCAGCAATATTCCAAAGTAGCATGACAAAAA
TCTTAGATCCTTTTAGAAAGCAAAATCCAGACATAGTTATTTACCAGTACAT?????????????????????
???
???
???
???
??????GAAGTAGTACCACTAACAGAAGAAGCAGAGCTAGAACTGGCAGAAAACAGGGAGATTCTAAAAGAAC
CGGTACATGGAGTGTATTAT???
???
??TAGTAATATGGGAAAAA
CCCCTAAATTTAAACTACCTATACAAAAGGAAACATGGGAAGCATGGTGGACAGAGTATTGGCAAGCCACCT?
GATTCCTGAGT?GGAATTTGTCAATACCCCTCCCCTAGTGAAACTAT?GTACCAGTTAGAAAAAGAACCCATA
GCAAGAGCAGAAACTTTCTATGTAGATAGGGCAGCTAATAGAGAAACTAAATTAAGAAAAGCAAGATATGTTA
CTGACAGAGGTAGGCAAAAAGTTGTCTCTCTAACTGACACAACAAATCAGAAAACTAAGTTACAAGCTATTAA
TCTAGCTTTGCAAGHTTCAAAGTTAGAAGTCAATATAGTAACAGACTCACAATATGCACTGGGAATCCTTCAA

GCACAACCAGATAAGAGTGAATCAGAAATAGTCAGTCATATAATAGAGCAGTTAATAAATAAGGAAAGGGTCT
ACCTGGCATGGGTACCAGCACACA??????????????AATGAACAAGTAGATAAATTGGTCAGTACTGGAAT
CAGAAGAGTACTGTTCTTAGATGGAATAGATAAGGCCCAAGAGGAGCATGAAAGATATCACAGTAATTGGAGA
GCAATGGCTAGTGATTTTAACCTACCACCTGTAGTAGCAAAAG??????????????????????????????
???
???
???
???
???ATTATAGGACAGGTAAGA
GATCAGGCTGAACATCTTAAGACAGCACTCCAAATGGCAGTATKCATCCACAATTTTAAGAAAAAAGGGGGGA
TTGGGGGTACAGTG???
???????????????????????????GGTTTATTACAGGGACAGCAGAGATCCACTTTGGAAAGGACCAGCA
AAGCTCCTTTGGAAAGGTGAAGGGGCAGTAGTAATACAAGATAATAGTGATATAAAAGTAGTACCAAGAAGMM
MMRMWAWGWTMWTWWKASMWWWTKRRMAWCMSMTRRYRGGWKRTGWWYKTSTGRMMKKWKKMYWGGATGAGGA
TTAGAACATGGAAAAGTCTAGTAAAACACCACATGTATAGTTCAAAGAAAGCTAAGGGATGGGTTTATAAACA
TCACTATGAAAGCACTCATCCAAGAATAAGTTCAGAAGTACACATCCCACTAGGGGATGCTAAATTRGTAATA
ACAACATATTGGGGTCTGCATACAGGAGAAAGAGACTGGCATTTGGGTCAGGGAGTCTCCATAGAATGGAGGA
AAAAGAGATATAGCACACAAGTAGACCCTGACCTAGCAGACCAACTAATTCATCTGCACTATTTTGATTGTTT
TTCAGAATCTGCTATAAGAAATGCCATATTAGGACKKAYWSTTAKWCMYAKSWRWRRAWRYCAAAGAACAGAA
AG??AGGTAGGATCTCTACAATACTTGGCACTAGCAGCATTAATAAAACCAAAACAGATAAAGCCACCTTTGC
CTAGTGTTAGGAAACTGACAGAGGATAGATGGAACAAGCCCCAGAGGACCAAGGGCCACAGAGGGAGCCATAC
AATGAATGGGCACTAGGGATTTTAGAGGAACTTAAAAATGAAGCTGTTAGACATTKTCCTRRGCCCTGGMKCC
AGGGCYYAGGAMAATRTATTTA??GAACTCT
GCAACAACTGCTGTTTATTCATTTCAGAATTGGGTGTCGGCATAGCAGAATAGGCATTGACATCCGACAGAGG
AGAGCAAGAAATGGATCCAGTAGATCCTAGACTAGAGCCCTGGAAGCATCCAGGAAGTCAGCCTAGGACTGCT
TGTAATAGTTGCTATTGTAAAAAGTGTTGCCTTCATTGCCAAGTTTGCTTCTTAAGAAAAGGCTTAGGCATCT
CCTATGGCAGGAAGAAGCGGAGACAGCGACGAAGAACTCCTTCAGAAAGTCAGACTCATCCGGCTTCTCTACC
AAAGCAGYCAGTTCTTTATCAAAGCAGTAASTCGWMWRKSYMRARSGACYYGTAATAGTAGCAATAGTAGCAT
TAGTAGTAGCAATAATAATAGCAATAGTTGTGTGGTCCATAGTAATCATAGAATATAGGAAAATATTAAGACA
AAGAAAAATAGACAGGTTAAKTGATAGACTAAKAGAAAGAGCAGAAGACAGTGGCAATGAGTT?ATTCTTTTG
TCACATTTTTCAATCTTACTTCTTGGSRMKAYRGRKCATCAWKMTCCTTGGGATATTGATGATCTGTAGTGCT
RSAGAACAATTGTGGGTCACAGTCTATTATGGGGTACCTGTGTGGAAAGAAGCAACTACCACTCTATTTTGTG
CCTCAGATGCTAAAGCATATGATACAGAAAGTCATAATGTTTGGGCCACACATGCCTGTGTACCCACAGACCC
CAACCCACAAGAAGTARKATTGRWAAATGTGACAGAARAWTTTAACATGKGRAAAAATRAYATGGTAGAWCAR
ATGCATGAGGATATAATCAGCTTATGGGATCAAARC??AAAGCCATGTGTAAAATTAACCCCACTTTGTGTTA
CTTTGAATTGTACTGAGCAGCTAAATAATACAAACAGCAATATCAACAGCAGTACAGTGGAGAACAARGAGGA
CAAAGGAGAGATACAAAACTGTTCTTTCTATGTCACCACAGACATAAGAGACAGAACACGGAAAGAATTTGCA
CTTTTTCACAAGCTTGATATAGTACCAATAGATGATGATAATAATATTACTAGTAACTATAGTAAMTATASSW
YRRYMAGTTGCAACACCTCAGTCATTAGACAGGCCTTGCCCACTTATCCAATGCCAATAATTCCCATACATTA
TTGTGCCCCTGCTGGTTTTGCGATTCTAAAATGTAACGAGGAAAAGTTCAATGGAACAGGACTGTGCAACAAT
GTCAGCACAGTACAATGT??????????????????GTAGTGTCAACTCAACTGCTGTTAAATGGCAGCCTAG
CAAAAAAAGAGGTAGTACTTAGGTCTAAAAATTTCACAGAGGTGTTGCAACTTATCAACCTA?TAGTT?????
???
???
???
???
???
???
???
???
????????????????????????CATTAAAAATAGCACCCACTAAGGCAAAAAAAAAAGTGGTGCAGAGAAA
AAAAAAAGCAATAGAATTAAAAGCTTTGTTCCTTAAATTCTTAGAAGCAGCAAAAAGCACTATAAGCGCAGCG
TCAATGACGCTGACGGTACAAGCCAGACAATTATTGTCTGGTATAGTGCAACAGCAGAACAATCTGCTGAAGG
CTATTRAGGCGCAACAGCATCTGTTGCAACTCACAGTCTGGRGCATCAAGCAGCTCCAGGCAAGAGTCCTGGC

TGTGGAAAGATACCTAAGAGATCAACAGCTCCTAGGGATTTGGGGTTGCTCTGGAAAACTCATCTGCACCACT
AATGTGCCTTGGAATACTAGTTGGAGTAATAAGACTAAGGATGAGATTTGGAATAACATGACCTGGATGCAGT
GGGAAAAAGAAATTGACAATTACACAGGCTTAATATACACTTTGCTTGAAGAATCACAGAACCAGCAGGAAAA
GAATGAACAAGAATTATTGGCATTGGATAAGTGGGCAAGTTTGTGGACTTGGCTTGACATAACAAACTGGCTG
TGGTATATAAAAATATTCATAATGATAGTAGGAGGCTTGATAGGTTTAAGAATAGTTTTTGCTGTACTTTCTA
TAATAAATAGAGTTAGGCAGGGATACTCACCATTATCGTTGCAGACCCACCTCCCAGCTCCGAGGGGACTCGA
CAGGCCCGAAGGAATCGAAGAAGAAGGTGGAGAGACAGACAGAGGCAGATCAATTCGATTAGTGGATGGCTTC
TTAGCACTTTTCTGGGACGACCTGAGGAACCTGTGCCTCTTCAGCTACCACCGCTTGAGAGACTTACTCTTGA
TTGTAACGAGGATTGTGGGACTTCTGGGACGCAGGGGGTGGGAACTCCTGAAATATTGGTGGAATCTCCTGCA
GTATTGGATTCAGGAACTAAAGAATAGTGCTGTTAACTTGCTCAATGCCACAGCCATAGCAGTAGCTGAGGGG
ACAGATAGGGTTATAGAAGTATTACAAGCAGCTTATAGAGCTATTCGCCACATACCTAGAAGAATAAGACAGG
ACTTGGAAAGGATTTTGCTATAAGCCCTTTGCTATAAAATGGGTGGCAAGTGGTCAAAGATGATTGGATGGCC
TGCTGTAAGGGAAAGAATGCACCGAGCTGAGCCAGCAGCAGAAGGGGTAGGAGCAGCATCTCGAGACCTGGAG
AGACATGGGGCACTCACAAGTAGCAATACAGCAGCTACTAATGCTGATTGTGCCTGGCTAGAAGCACAAGAGG
ATGAGGAGGTGGGCTTTCCAGTCAGACCCCAGGTACCTTTAAGACCAATGACTTACAAGGGAGCGATAGATCT
CAGCCACTTTTTAAGAGAAAAGGGGGGACTGGAAGGGCTAATTTACTCCCAGAAGAGACAAGATATCCTAGAT
CTGTGGGTCTATCACACACAAGGCTACTTCCCTGATTGGCAAAACTACACACCAGGGCCAGGGGTCAGATATC
CACTGACCTTTGGATGGTGCTTCAAGCTAGTACCAGTTGACCCAGACCAGGTAGAGAAGGCCAATGAAGGGGA
GAACAACAGCTTGCTGCACCCTATGAGCCTGCATGGGATAGAGGACCCGGAGAAAGAAGTGCTGATGTGGAAG
TTTGACAGCCGCCTAGCATACAGTCACATGGCCCGAGAGCTGCATCCGGAGTACTACAAGAACTGATGACACC
GAGTTTCTACAAGGGACTTTCCGCTGGGGACTTTCCAGGGGAGGCGCGGCCTGGGCGGGACTGGGGAGTGGCG
AGCCCTCAGATGCTGCATATAAGCAGCTGCTTTTTGCCTGTACTGGGTCTCTCTGGTTAGACCAGATCTGAGC
CTGGGAGCTCTCTGGCTAACTAGGGAACCCACTGCTTAAGCCTCAATAAAGCTTGCCTTGAGTGCTTTAAGTA
GTGTGTGCCCGTCTGTTGTGTGACTCTGGTAACTAGAGATCCCTCAGACCCTTTAGTCAGTGTGGAAAATCTC
TAGC

>K-C5 HIV-1 genome, derived from RNA genomic sequence
CCTATGAGCCTGCATGGGATAGAGGACCCGGAGAAAGAAGTGCTGATGTGGAAGTTTGACAGCCGCCTAGCAT
TCCATCACATGGCCCGAGAGCTGCATCCGGAGTACTACAAGAACTGATGACACCGAGTTTCTACAAGGGACTT
TCCGCTGGGGACTTTCCAGGGGAGGCGCGGCCTGGGCGGGACTGGGGAGTGGCGAGCCCTCAGATGCTGCATA
TAAGCAGCTGCTTTTTGCCTGTACTGGGTCTCTCTGGTTAGACCAGATCTGAGCCTGGGAGCTCTCTGGCTAA
CTAGGGAACCCACTGCTTAAGCCTCAATAAAGCTTGCCTTGAGTGCTTTAAGTAGTGTGTGCCCGTCTGTTGT
GTGACTCTGGTAACTAGAGATCCCTCAGACCCTTTTAGTC?????????????????????????????????
???
???
???????????????????AAGCGGAGGAGAACTAGATAAATGGGAAAAAATTCGGTTAAGGCCAGGGGGAAA
GAAAAGGTATAAACTAAAACATATAGTATGGGCAAGCAGGGAGCTAGAACGATTCGCAGTTAATCCTGGCCTT
TTAGAGACATCAGAAGGCTGTAGACAAATACTGGACAGCTACAACCG??????????????????????????
???GATCATTATTTAATACAATAGCAGTCCTCTATTGTGTACATCAAAGAATAGAGGTAAAAGATACCAAAGA
AGCTTTAGAGAAGGTAGAGGAAGAGCAAAACAAAAGTAAGAAAAAAGCACAGCAAACAGCAGCAGCTGCAGCT
GACACAGGAAACGGCGGCAAGGTCAGCCAAAATTTTCCTATAGTGCAGAACCTACAGGGGCAAATGGTACATC
AGCCCCTATCACCTAGAACTTTAAATGCATGGGTAAAAGTAGTAGAAGAGAAGGCTTTTAGCCCAGAAGTAAT
ACMCATGTTTGCAGCATTATCAGAAGGAGCCACCCCACAAGATTTAAACACCATGCTAAACACAGTGGGAGGA
CATCAAGCAGCTATGCAAATGTTAAAAGAGACCATCAATGAGGAAGCTGCAGAATGGGATAGATTGCATCCAG
TGCATGCAGGGCCTGTTGCACCAGGCCAGATGAGAGAACCAAGGGGAAGTGACATAGCAGGAACTACTAGTAC
CCTTCAGGAACAAATAGGCTGGATGACACATAATCCACCTATCCCAGT?????????????????????????
????????????????????????????TGTATAGCCCTACCAGCATTCTGGACATAAGACAAGGACCAAAGG
AACCCTTTAGAGACTATGTAGACCGATTCTATAAAACTCTAAGAGCCGAGCAAGCTTCACAAGAGGTAAAAAA
TTGGATGACAGAAACCTTGTTGGTCCAAAATGCGAACCCAGATTGTAAGACTATCTTAAAAGCATTAGGACCA
GCAGCCACACTAGAAGAAATGATGACAGCATGTCAGGGAGTAGGGGGACCCGGCCATAAAGCGAGAGTTTTGG
CCGAAGCAATGAGCCAGGTAACAAATTCAGCTACCGTAATGATGCAGAAAGGCAACTTTAGGAACCAAAGAAA
GGCTGTCAAGTGTTTCAATTGTGGCAAAGAAGGGCACATAGCCAAAAATTGCAGGGCCCCTAGGAAAAAGGGC
TGTTGGAAATGTGGAAAGGAAGGACACCAAATGAAAGATTGTACTG???????GGCTAATTTTTTAGGGAAAA
TCTGGCCTTCCCACAAGGGAAGGCCAGGGAATTTCCTTCAGAGCAGACCAGAGCCAACAGCCCCACTAGAGAG
CCTCAGGTTTGGGGAGGAGACAACAACTCCCTATCAGAAACAGGAGCAGATGGAC??????????????????
??????????????????????????????????TGTCACAATAAAAGTAGGAGGGCAGCTAAAGGAAGCTCT
ATTAGATACAGGAGCAGATGATACAGTATTAGAAGAAATGAATTTGCCAGGAAGATGGAAACCAAAAATGATA
GGGGGAATTGGAGGTTTTATCAAAGTAAAACAGTATGATCAGATACCCATAGAAATCTGTGGGCATAAAGCTA
TAGGTACAGTATTAATAGGACCTACACCTGTCAACATAATTGGAAGAAATCTGTTGACTCAGCTTGGTTGCAC
TTTAAATTTTCCCATTAGTCCTATTGAAACTGTACCAGTAAAATTAAAGCCAGGAATGGATGGCCCAAAAGTT
AAACAGTGGCCATTGACAGAGGAAAAAATAAAAGCATTAGTAGAAA????????GAAATAAAAAAGGAAGGAA
AAATTTCAAAAGTAGGGCCTGAAAACCCATACAATACTCC?????????????????????????????????
???
???????????????????????????????????????GTACTGGATGTAGGTGATGCTTATTTTTCAGTTC
CCTTAGATAAAGACTTCAGGAAGTACATTGTATTTACCATACCCAGCATAAATAATGAGACACCAGGGATTAG
ATATCAGTACAATGTACTTCCGCAGGGATGGAAAGGATCACCAGCAATATTCCAAAGTAGCATGACCAAAATC
TTAGATCCTTTTAGAAAGCAAAATCCAGACATAGTTATTTACCAATACATGGATGATTTGTATGTAGGATCTG
ACTTAGAAATAGGGCAGCACAGAGCAAAAATAGAGGAACTAAGACAACATCTKTTGAAGTGGGGATTCACCAC
ACCAGACAAAAAACATCAGAAMGAACCTCCATKCMTTTGGATGGGTTATGAACTCCATCCTGATAAATGGACA
GTACAGCCTATAGAGCTGCCAGAAAAAGACAGCTGGACTGTCAATGACATACAGAAGTTAGTGGGAAAATTAA
ATTGGGCAAGTCAGATTTACGCAGGGATTAAAGTAAGGCAATTATGTAAACTCCTTAGGGGAGCCAAAGCACT
AACAGAAGTAATAACACTAACAGAAGAAGCAGAACTAGAGCTGGCAGAAAACAGGRAGATTMTAAAAACACCA
GTACATGGAGTGTACTATGACCCATCAAAAGACTTAGTAGCAGAAATACAGAAGCAGGGGCAAGGTCAATGGA
CATATCAAATTTATCAAGAGC?ATTTAAAAATCTGAAAACAGGAAAGTATGCAAGAATGAGGAGTGCCCACAC
T??
???
???
???GTTACT
GACAGAGGAAGACAAAAAGTTGTCTCCCTAACTGACACAACAAATCAGAAAACTGAGTTACAAGCTATTGATC
TAGCTTTGCAAGATTCAGGGTTAAAAGTAAATATAGTAACAGACTCACAATATGCACTGAAAATCCTTCAAGC

ACAACCAGATAAAAGTAAATCAGAAATAGTCAGTCATATAATAAAGC??????????????????????????
???GTAATGGAATCA
GAAGAGTACTATTCTTAGATGGAATAGATAAGGCCCAAGAGGAGCATGAAAGATATCACAGTAATTGGAGAGC
AATGGCTAGTGATTTTAACCTACCACC??
???AGATTGTACACATCTAGAGG
GAAAAATTATCATAGTAGCAGTTCATGTAGCCAGTGGATATATAGAGGCAGAAGTTATTCCAGCAGAGACAGG
GCAGGAAACAGCATACTTTCTCTTAAAATTAGCAGGGAGATGGCCAGTAAAAACAATACATACAGATAATGGC
CCCAATTTCATCAGTTCCGCGGTTAAGGCCGCCTGTTGGTGGGCAGGGATCAAGCAGGAATTTGGCATTCCCT
ACAATCCCCAAAGTCAAGGAGTAGTAGAGTMTATGAATAAGGAAKTAAAGACAATTATAGGAMAGGTAAGAGA
TMAGGCKGAACATCTTAAGACAGCACTACAAATGGCAGTATTCATCCACAATTTTAAGAAAAAAGGGGGGATT
GGGGGGTACAGTGCAGGGGAAAGAATAATAGACATAATAGCATCAGACATAC?????????????????????
???????????????????????????????????CAGGACAGCAGAGATCCACTTTGGAAAGGACCAGCAAA
GCTCYTTTGGAAAGGTGAAGGGGCAGTAGTAATACAAGATAATAGTGATATAAAAGTAGTACCAAGAAGAAAA
GCAAAGATCATTAGAGATTATGGAAAACAGATGGCAGGTGATGATTGTGTGGCAGGTGGACAGGATGAGGATT
AGAACATGGAAAAGTTTAGTAAAACACCACATGTATATTTCAAAGAAAGCTAAAGGATGGTTTTATAAACATC
ACTATGAAAGCACTCATCCAAGAATAAGTTCAGAAGTACACATCCCATTAGGGGATGCTAAATTGGTAATAAT
AACATATTGGGGTCTGCATACAGGAGAAAGAGACTGGCATTTGGGCCAGGGAGCCTCCATAGAGTGGAGGAAA
GAGAAATATAGCACACAAATAGACCCTGGCCTAGCAGACCAACTAATTCATATGCACTATTTTGATT??????
???????????????????????ATATTAGGACGTATAGTTAGTCCTAGGTGTGAATATCAAGCAGGACATAA
CAAGGTAGGATCTCTACAATATTTGGCACTAACAGCATTAATAAAACCAAAAGGGATAAAGCCACCTTTGCCT
AGTGTAGCGAAACTGACAGAGGATAGATGGAACAAGCCCCAGAGGACCAAGGGCCACAGAGGGAGCCATACAA
TGAATGGGCACTAGGGATTTTAGAGGAACTTAAAAATGAAGCTGTTAGACATTTTCCTGGGCCCTGGCTCCAG
GGCTTAGGACAATACATTTATGTAACTTATGGAAATACTTGGACAGGAGTGGAAGCCCTAATAAGAACTCTGC
AACAACTGCTGTTTATTCATTTCAGAATTGGGTGTCGGCATAGCAGAATAGGCATTGACATCCGACAGAGGAG
AGCAAGAAATGGATCCAGTAGATCCTAGACTAGAGCCCTGGAAGCATCCAGGAAGTCAGCCTAGGACTGCTTG
TAATAGTTGCTATTGTAAAAAGTGTTGCCTTCATTGCCAAGTTTGCTTCTTAAGAAAAGGCTTAGGCATCTCC
TATGGCAGGAAGAAGCGGAGACAGCGACGAAGAACTCCTTCAGAAAGTCAGACTCATCCGGCTTCTCTACCAA
AGCAGTAAGTTCTTTATCAAAGCAGTAAGTAGTATATGTAATGCAACCTGTACAAATATTAGCAATAGTAGCA
TTAGTAGTAGTAGCAATAATAGCAATAGTTGTAWGGACCATARTAGCCATAGAATATAGAAA???????????
???
????????????????????????TGTGGAGATGGGGCATCATGCTCCTTGGGATATTGATGATCTGTAGTGC
TGGAGAACAATTGTGGGTCACAGTCTATTA?????????????????????????????CCACTCTATTTTGT
GCCTCAGATGCTAAAGCATATGATACAGAAAGTCATAATGTTTGGGCCACACATGCCTGTGTACCCACAGACC
CCAACCCACAAGAAGTAAGATTGAAAAATGTGACAGAAGAATTTAACATGGGAAAAAATAATATGGTAGATCA
AATGCATGAGGATATAATCAGCTTATGGGATCAAAGCTTAAAGCCATGTGTAAAATTAACCCCACTTTGTGTT
ACTTTACATTGCACTGAGCCGACGAAGAGGAATAATACTAACAGCACTGATGCTCAGAGC?????????????
???
???AGTAAC
TATAGGTTGATAAGTTGCAACACCTCAGTCATTAGACAGGCCTGTCCAAAGGTATCTTTTGAGCCAATTCCCA
TACATTATTGTGCCCCTGCTGGTTTTGCGATTCTAAAGTGTAACGAGGAAAAATTCAATGGAACAGGACTATG
CAGCGATGTCAGCACAGTACAATGTACACATGGAATTAAACCAGTAGTGTCAACTCAACTGCTGTG???????
??CCATAATAGTACAAC
TGACAGAACCTGTAGTAATCAATTGTATAAGACCCAACAACAACACAAGAAAAAGGATACGTATAGGACCAGG
GGGCGCATCATTTTATGCAACAAACATAATAGGAGATATAAGAAAAGCATATTGTATCATTAATAGCACACAA
TGGAATAACACTTTACAACAGGTAGTTAAAAAATTAGAGAACAATTTGGGAATAAAACAATAGTCTTTAAT?
???
???
???????????????????????????????????AATAAACATGTGGCAGGAAGTAGGAAAAGCAATGTATG
CCCCTCCCATCAGTGGACAAATTAACTGTTCATCAAATATTACAGGGCTGCTATTAACAAGAGACGGTGGTAA
TAAAGGTGATAATGAAAACAGTACAGAGACTTTCAGACCAGGAGGAGGAAATATGAAGGACAATTGGAGAAGT
GAATTATATAAATACAAAGTAGTAAAAATTGAACCATTAGGAATAGCAC????????????????????????
???
?????????????????????????GTACAGGCCAGACAATTATTGTCTGGTATAGTGCAACAGCAGAACAAT
CTGCTGAGGGCTATTGAGGCGCAACAGCATCTGTTGCAACTCACAGTCTGGGGCATCAAGCAGCTCCAGGCAA

GAGTCCTGGCTGTGGAAAGATACCTAAGAGATCAACAGCTCCTAGGGATTTGGGGTTGCTCTGGAAAACTCAT
CTGCACCACTAATGTGCCTTGGAATACTAGTTGGAGTAATAAGACTAAGGATGAGATTTGGAATAACATGACC
TGGATGCAGTGGGAAAAAGAAATTGACAATTACACAGGCTTAATATACACTTTGCTTGAAGAATCACAGAACC
AGCAGGAAAAGAATGAACAAGAATTATTGGCATTGGATAAGTGGGCAAGTTTGTGGACTTGGCTTGACATAAC
AAACTGGCTGTGGTATATAAAAATATTCATAATGATAGTAGGAGGCTTGATAGGTTTAAGAATAGTTTTTGCT
GTACTTTCTATAATAAATAGAGTTAGGCAGGGATACTCACCATTATCGTTGCAGACCCACCTCCCAGCTCCGA
GGGGACTCGACAGGCCCGAAGGAATCGAAGAAGAAGGTGGAGAGACAGACAGAGGCAGATCAATTCGATTAGT
GGATGGCTTCTTAGCACTTTTCTGGGACGACCTGAGGAACCTGTGCCTCTTCAGCTACCACCGCTTGAGAGAC
TTACTCTTGATTGTAACGAGGATTGTGGGACTTCTGGGACGCAGGGGGTGGGAACTCCTGAAATATTGGTGGA
ATCTCCTGCTGTATTGGATTCAGGAACTAAAGAATAGTGCTACTAGCTTGCTGAACGCCACAGCTATAGCAGT
TGCTGAGGGGACAGATAGGATTATAGAAGTAGTACAAAGAATTCTTAGAGCTATCCTTCACATACCTAGAAGA
ATAAGACAGGGCTTCGAAAGGGCTTTGCTATAAGGGCTTTGCTATAAAATGGGTGGCAAGTGGTCAAAGATGA
TTGGATGGCCTGCTGTAAGGGAAAGGATGCAACGAGCTGAGCCAGCAGCAGAAGGGGTAGGAGCAGCATCTCG
AGATCTGGAGAGACATGGAGCACTTACAAGTAGCAATACAGCAGCTACTAATGCTGATTGTGCCTGGCTAGAA
GCACAAGAAGATGAGGAGGTGGGCTTTCCAGTCAGACCCCAGGTACCTTTAAGACCAATGACTTACAAGGGAG
CGGTAGATCTTAGCCACTTTTTAAGAGAAAAGGGGGGACTGGAAGGGCTAATTTACTCCCAGAAGAGACAAGA
TATCCTAGATCTGTGGGTCTACCACACACAAGGCTACTTCCCTGATTGGCACAACTACACACCAGGGCCAGGG
GTCAGATATCCACTGACCTTTGGATGGTGCTTCAAGCTAGTACCAGTTGATCCAGACCAGGTAGAGAAGGCCA
ATGAAGGGGAGAACAACAGCTTGCTGCACCCTATGAGCCTGCATGGGATAGAGGACCCGGAGAAAGAAGTGCT
GATGTGGAAGTTTGACAGCCGCCTAGCATTCCATCACATGGCCCGAGAGCTGCATCCGGAGTACTACAAGAAC
TGATGACACCGAGTTTCTACAAGGGACTTTCCGCTGGGGACTTTCCAGGGGAGGCGCGGCCTGGGCGGGACTG
GGGAGTGGCGAGCCCTCAGATGCTGCATATAAGCAGCTGCTTTTTGCCTGTACTGGGTCTCTCTGGTTAGACC
AGATCTGAGCCTGGGAGCTCTCTGGCTAACTAGGGAACCCACTGCTTAAGCCTCAATAAAGCTTGCCTTGAGT
GCTTTAAGTAGTGTGTGCCCGTCTGTTGTGTGACTCTGGTAACTAGAGATCCCTCAGACCCTTTAGTC?????
??????????????

>K-C6 HIV-1 genome, derived from RNA genomic sequence
CCTATGAGCCTGCATGGGATAGAGGACCCGGAGAAAGAAGTGCTGATGTGGAAGTTTGACAGCCGCCTAGCAT
TCCATCACATGGCCCGAGAGCTGCATCCGGAGTACTACAAGAACTGATGACACCGAGTTTCTACAAGGACTT
TCCGCTGGGGACTTTCCAGGGGAGGCGCGGCCTGGGCGGGACTGGGGAGTGGCGAGCCCTCAGATGCTGCATA
TAAGCAGCTGCTTTTTGCCTGTACTGGGTCTCTCTGGTTAGACCAGATCTGAGCCTGGGAGCTCTCTGGCTAA
CTAGGGAACCCACTGCTTAAGCCTCAATAAAGCTTGCCTTGAGTGCTTCAAGTAGTGTGTGCCCGTCTGTTGT
GTGACTCTGGTAACTAGAGATCCCTCAGACCTTTTAGTCAGTGTGGAAAATCTCTAGCAGTGGCGCCCGAACA
GGGACGCGAAAGCGAAAGTAAGACCGGAGGAGCTCTCTCGACGCAGGACTCGGCTTGCTGAAGCGCGCACGGC
AAGAGGCGAGGGGCGGCGACTGGTGAGTACGCCAAAATTTTTGACTAGCGGAGGCTAGAAGGAGAGAGATGGG
TGCGAGAGCGTCAGTATTAAGCGGGGGAGAATTAGATAAAWGGGARAAAAWTCGGWTAAGGCMAGGGGGAMAG
AAAAGGTATAAGCTAAAACATATAGTATGGGCAAGCAGGGAGCTAGAACGATTCGCAGTCAACCCTGGCCTGT
TAGAAACATCAGAAGGCTGTAGACAAATACTGGACAGCTACAAC?????????????????????????????
???
???
?????GGAAATGGCGGCAAGGTCAGCCAAAATTTTCCTATAGTGCAGAACCTACAGGGGCAAATGGTACATCA
GCCCCTATCACCTAGAACTTTAAATGCATGGGTAAAAGTAGTAGAAGAAAAAGCTTTCAGCCCAGAAGTAATA
CCCATGTTTGCAGCATTATCAGAAGGAGCCACCCCACAAGATTTAAACACCATGCTAAACACAGTGGGAGGAC
ATCAAGCAGCTATGCAAATGTTAAAAGAGACCATCAATGAGGAAGCTGCAGAATGGGATAGATTGCATCCAGT
G????????????GTTGCACCAGGTCAAATGAGAGAACCAAGGGGAAGTGACATAGCAGGAACTACTAGTACC
CTTCAGGAACAAATAGGCTGGATGACACATAATCCACCTATCCCAGTAGGAGAAATTTATAAAAAATGGATAA
TCATGGGATTAAATAAAATAGTAAGAATGTATAGCCCTACCAGCATTCTGGACATAAGACAAGGACCAAAGGA
ACCCTTTAGAGATTATGTAGACCGGTTCTATAAAACTMTAAGAGCTGAGCAGGCTTCACAAGAGGTAAAAAAT
TGGATGACAGAAACCTTGTTGGTCCAAAATGCGAACCCAGATTGTAAGACTATCCTAAAAGCATTAGGACCAG
CAGCCACACTAGAAGAAATRATGACAGCATGTCAGGRAGTAGGGGGACCCGGCCATAAAGCAARAGTTTTGGC
CGAAGCAATGAGCCAGGTAACAAATTCAGCTACMGTAATGATGCAGAAAGGCAACTTTAGGAACCAAAGAAAG
GCTGTCAAGTGTTTCAATTGTGGCAAAGAAGGGCACATAGCCAGAAATTGCAGGGCCCCTAGGAAAAAGGGCT
GTTGGAAATGTGGAAAGGAAGGACACCAAATGAAGGATTGTGTTGAGAGACAGGCTAATTTTTTAGGGAARAT
CTGGCCTTCCCACAAGGGAAGGCCAGAGAATTTCCTTCAGAGCAGACCAGAGCCAACAGCCCCACTAGAGCCA
ACAGCCCCTCCAGAGGAGAGCCTCAGGTTTGGGGAGGAGACAACAACTCCCTATCAGAAACAGGAGCAGATGG
ACAAGGAGAAGTATCCTTTGACTTCCCTCAGATCACTCTTTGGCAACGACCCCTTGTCACAATAAAAGTAGGA
GGGCAGCTAAAGGAAGCTCTATTAGATACAGGAGCARATGATACAGTATTAGAAGAAATGAATTTGCCAGGAA
GATGGAAACCAAAAATGATAGGGGGAATTGGAGGTTTTATCAAAGTAAAACAGTATGATCAGATAGCCATAGA
AATCTGTGGGCATAAAACTATAGGTACAGTATTAGTAGGACCTACACCTGTCAACATAATTGGAAGAAATCTG
TTGACTCAGCTTGGTTGCACTTTAAATTTTCCCATTAGTCCTATTGAAACTGTACCRGTAAAATTAAAGCCAG
GAATGGATGGCCCAAAAGTTAAACAGTGGCCATTGACAGAGGAAAAAATAAAAGCATTAGTAGAAATTTGTAC
AGAAATGGAAAAGGAAGGAAAAATTTCAAAAGTAGGGCCTGAAAATCCATACAATACTCCAGTATTTGCCATA
AAGAAAAAAGATGGTACTAAATGGAGAAAACTAGTAGATTTCAGAGAACTTAATAAGAGAACTCAAGACTTCT
GGGAAGTTCAATTAGGAATACCACATCCTGCAGGGTTAAAAAAGAAAAAATCAGTAACAGTACTGGATGTAGG
TGATGCTTATTTTTCAGTTCCCTTAGATAAAGACTTCAGGAAGTATACTGCATTTACCATACCCAGTATAAAT
AATGAGACACCAGGGATTAGATATCAGTACAATGTACTTCCGCAGGGATGGAAAGGATCACCAGCAATATTCC
AAAGTAGCATGACCAAAATCTTAGATCCTTTTAGAAAGCAAAATCCAGACATAGTTATTTACCAGTACATGGA
TGATTTGTATGTAGGATCTGACTTAGAAATAGGGCAGCACAGAATAAAAATAGAGGAACTAAGACAACATCTG
TTGAAGTGGGGATTCACCACACCAGACAAAAAACATCAGAAAGAACCTCCATTCCTTTGGATGGGTTATGAAC
TCCATCCTGATAAATGGACAGTACAGCCTATAGAGCTGCCAGAAAAAGACAGCTGGACTGTCAATGACATACA
GAAGTTAGTGGGAAAATTAAATTGGGCAAGTCAAATTTATGCAGGGATTAAAGTAAGGCAATTATGTAAACTC
CTTAGGGGAGCCAAGGCACTAACAGAAGTAATAACACTAACAGCAGAAGCAGAACTAGAGCTGGCAGAAAACA
GGGAGATTTTAAAAACACCAGTGCATGGAGTGTACTATGACCCATCAAAAGACTTAGTAGCAGAAATACAAAA
GCAGGGGCAAGGTCAATGGACATATCAAATTTATCAAGAGCCATTTAGAAATCTGAAAACAGGAAAGTATGCA
AGAATGAGGAGTGCCCACACTAATGATGTAAAACAGTTAACAGAGGCAGTGCAAAAGATATCCACAGAAAGCA
TAGTAATATGGGGAAAGACCCCTAAATTTAAACTACCTATACAAAAGGAAACATGGGAAACATGGTGGGCGGA
GTATTGGCAAGCCACCTGGATTCCTGAGTGGA???
???AG
GAAAAGCAGGATATGTTACTGACAGAGGTAGACAAAAAGTTGTCTCTCTAACTGACACAACAATCAGAAAAC
TGAGTTACAAGCTATTGATCTAGCTTTGCAGGATTCAGGGTTAGAAGTAAATATAGTAACAGACTCACAATAT

GCATTGGGAATCATTCAAGCACAACCAGATAAGAGTAAATCAGAAATAGTCAGTCATATAATAGAGCAGTTAA
TAAATAAGGAAAGGGTCTACCTGGCATGGGTACCAGCACACAAAGGAATTGGGGGAAATGAACAAGTAGATAA
ATTGGTCAGTACTGGAATCAGAAGAGTACTATTCCTAGATGGAATAGATAAGGCCCAAGAGGAGCATGAAAGA
TATCACAGTAATTGGAGAGCAATGGCTAGTGATTTTAACCTACCACCTGTAGTAGCAAAAGAAATAGTAGCCT
GCTGTGATAAATGTCAACTAAAAGGAGAAGCCATGCATGGACAAGTAGACTGTAGTCCAGGAATATGGCAACT
AGATTGTACACATCTAGAAGGAAAAATTATCATAGTAGCAGTTCATGTAGCC??????????????????GAA
GTTATTCCAGCAGAGACAGGGCAGGAAACAGCATACTTTCTCTTAAAATTAGCAGGGAGATGGCCAGTAAAAA
CAATACATACAGATAATGGCCCCAATTTCATCAGCTCCGCGGTCAAGGCCGCCTGTTGGTGGGCAGGGATCAA
GCAGGAATTTGGCATTCCCTACAATCCCCAAAGTCAAGGAGTAGTAGAGTCTATGAATAAGGAATTAAAGACA
ATTATAGGACAGGTAAGAGATCAGGCTGAACATCTTAAGACAGCACTACAAATGGCAGTATTCATCCACAATT
TTAAGAAAAAAGGGGGGATTGGGGGGTACAGTGCAGGGGAAAGAWTAATAGACATAATAGCATCAGACATACA
GACTAAAGAACTACAAAAACAAATTACAAAAATTCAAAATTTTCGGGTTTATTACAGGGACAGCAGAGATCCA
CTTTGGAAAGGACCAGCAAAGCTCCTTTGGAAAGGTGAAGGGGCAGTAGTAATACAAGATAATAGTGATATAA
AAGTAGTACCAAGAAGAAAAGCAAAGATCATTAGAGATTATGGAAAACAGATGGCAGGTGATGATTGTGTGGC
AGGTGGACAGGATGAGGATTAGAACATGGAAAAGTTTAGTAAAACACCACATGTATATTTCAAAGAAAGCTAA
GGGATGGTTTTATAAACATCACTATGAAAGCACTCATCCAAGAATAAGTTCAGAAGTACACATCCCATTAGGG
GATGCTAAATTGGTAATAATAACATATTGGGGTCTGCATACAGGAGAAAGAGACTGGCATTTGGGCCAGGGAG
CCTCTATAGAATGGAGGAAAGAGAGATATAGCACACAAGTAGACCCTGGCCTAGCAGACCAACTAATTCATAT
GCACTATTTTGATTGTTTTTCAGAATCTGCTATAAGAAATGCCATATTAGGACGTATAGTTAGTCCTAGGTGT
GAATATCAAGCAGGACATAAAAAGGTAGGATCTCTACAATATTTGGCACTAACAGCATTAATAAAACCAAAAG
GGATAAAGCCACCTTTGCCTAGTGTAGCGAAACTGACAGAGGATAGATGGAACAAGCCCCAGAGGACCAAGGG
CCACAGAGGGAGCCATACAATGAATGGGCACTAGGGATTTTAGAGGAACTTAAAAATGAAGCTGTTAGACATT
TTCCTGGGCCCTGGCTCCAGGGCTTAGGACAATATATTTA???AACTTATGGAAATACCTGGACAGGAGTGGA
AGCCCTAATAAGAACTCTGCAACAACTGCTGTTTATTCATTTCAGAATTGGGTGTCGGCATAGCAGAATAGGC
ATTGACATCCGACAGAGGAGAGCAAGAAATGGATCCAGTAGATCCTAGACTAGAGCCCTGGAAGCATCCAGGA
AGTCAGCCTAGGACTGCTTGTAATAGTTGCTATTGTAAAAAGTGTTGCCTTCATTGCCAAGTTTGCTTCTTAA
GAAAAGGCTTAGGCATCTCCTATGGCAGGAAGAAGCGGAGACAGCGACGAAGAACTCCTTCAGAAAGTCAGAC
TCATCCGGCTTCTCTACCAAAGCAGTAAGTTCTTTATCAAAGCAGTAAGTAGTATATGT??????????????
???
????????????????????????GAAAATAGACAGGTTAATTGAAAGAATAAGTGAAAGAGCAGAAGACAGT
GGCAATGAGAGCGAGGGGGATCAGGAAGAGTTGTCAGCACTTGTGGAGATGGGGCATCATGCTCCTTGGGATA
TTGATGATCTGTAGTGCTGGAGAACAATTGTGGGTCACAGTCTATTATGGGGTACCTGTGTGGAAAGAAGCAA
CCACCACTCTATTTTGTGCCTCAGATGCTAAAGCATATGATACAGAAAGTCATAATGTTTGGGCCACACATGC
CTGTGTACCCACAGACCCCAACCCACAAGAAGTAAGATTGAAAAATGTGACAGAAGAATTTAACATGGGAAAA
AATAATATGGTAGATCAAATGCATGAGGATATAATCAGCTTATGGGATCAAAGCTTAAAGCCATGTGTAAAAT
TAACCCCACTTTGTGTTACTTTACATTGCACTGAGCCGACGAAGAGGAATAATACTAACAGC???????????
??????????????????????????????????????AAACTGTTCTTTCAATATCACCACAGACATAAGAGAC
AGAATGCAGAAAGAATTTGCACTTTTTCACAAGCTTAATCTARTAMTATTACTAGTAACTATA?GTAACTATA
GGTTG?TAAGTTGCAACACCTCAGTCATTAGACAGGCCTGTCCAAAGGTATCTTTTGAGCCAATTCCCATACA
TTATTGTGCCCCTGCTGGTTTTGCGATTCTAAAGTGTAACGAGGAAAAGTTCAATGGAACAGGACTATGCAGC
GAYGTCAGCACAGTACAATGTACACATGGAATTAAACCAGTAGTGTCAACTCAACTGCTGTTAAATGGCAGCC
TAGCAAAAGAAGAGGTAGTACTTAGGTCTGAAAATTTCACAGAAAATACTAAAACCATAATAGTGCAGCTGAA
GGACCCTGTAGTAATTAATTGTACAAGACCCAACAACAATATAAGAAAAAGGATACGTATAGGTATAGGACCA
GGGGGCGCATCATTTTATGCAACAAACATAATGGGAGATATAAGAAAAGCATATTGTATCATTAATAGCACAC
AATGGAATAACACTTTACAACAGGTAGTTAAAAAATTAAGAGAACAATTTGGGAATAAAACAATAGTCTTTAA
TCAATCCTCAGGAGGGGACCCAGAAGTTGTAAGGTATAGTTTTAATTGTGGAGGGGAATTTTTCTACTGTGAT
TC???
?????????????????????????????AAAACAAATAATAAACATGTGGCAGGAAGTAGGAAAAGCAATGTA
TGCCCCTCCCATCAGTGGACTAATTAACTGTTCATCAAATATTACAGGGCTGCTATTAACAAGAGACGGTGGT
AATAATGAAAACAGTACAGAGACTTTCAGACCAGGAGGAGGAAATATGAAGGACAATTGGAGAAGTGAATTAT
ATAAATACAAAGTAGTAAAAATTGAACCATTAGGAATAGCACCCACCGAGGCAAAGAGAAGAGTGGTGCAGAG
AGAAAAAAGAGCAATAGGATTAGGAGCTTTGTTCCTTGGGTTCTTGGGAGCAGCAGGAAGCACTATGGGCGCA
GCGTCAATGACGCTGACGGTACAGGCCAGACAATTATTGTCTGGTATAGTGCAACAGCAGAACAACCTGCTGA
GGGCTATTGAGGCGCAACAGCATCTGTTGCAACTCACAGTCTGGGGCATCAAGCAGCTCCAGGCAAGAGTCCT

GGCTGTGGAAAGATACCTAAGAGATCAACAGCTCCTAGGGATTTGGGGTTGCTCTGGAAAACTCATCTGCACC
ACTAATGTGCCTTGGAATACTAGTTGGAGTAATAAGACTAAGGATGAGATTTGGAATAACATGACCTGGATGC
AGTGGGAAAAAGAAATTGACAATTACACAGGCTTAATATACACTTTGCTTGAAGAATCACAGAACCAGCAGGA
AAAGAATGAACAAGAATTATTGGCATTGGATAAGTGGGCAAGTTTGTGGACTTGGCTTGACATAACACACTGG
CTGTGGTATATAAAAATATTCATAATGATAGTAGGAGGCTTGATAGGCTTAAGAATAGTTTTTGCTGTGCTTT
CTATAATAAATAGAGTTAGGCAGGGATACTCACCATTATCGTTGCAGACCCACCTCCCAGCTCCGAGGGGACT
CGACAGGCCCGAAGGAATCGAAGAAGAAGGTGGAGAGACAGACAGAGGCAGATCAATTCGATTAGTGGATGGC
TTCTTAGCACTTTTCTGGGACGACCTGAGGAACCTGTGCCTCTTCAGCTACCACCGCTTGAGAGACTTACTCT
TGATTGTAACGAGGATTGTGGGACTTCTGGGACGCAGGGGGTGGGAACTCCTGAAATATTGGTGGAATCTCCT
GCAGTATTGGATTCAGGAACTAAAGAATAGTGCTACTAGCTTGCTGAACGCCACAGCTATAGCAGTTGCTGAG
GGGACAGATAGGATTATAGAAGTAGTACAAAGAATTCTTAGAGCTATCCTTCACATACCTAGAAGAATAAGAC
AGGGCTTCGAAAGGGCTTTGCTATAAGGGCTTTGCTATAAAATGGGTGGCAAGTGGTCAAAGATGATTGGATG
GCCTGCTGTAAGGGAAAGAATGCACCGAGCTGAGCCAGCAGCAGAAGGGGTAGGAGCAGCATCTCGAGACCTG
GAGAGACATGGGGCACTCACAAGTAGCAATACAGCAGCTACTAATGCTGATTGTGCCTGGCTAGAAGCACAAG
AGGATGAGGAGGTGGGCTTTCCAGTCAGACCCCAGGTACCTTTAAGACCAATGACTTACAAGGGAGCGGTAGA
TCTCAGCCACTTTTTAAGAGAAAAGGGGGGACTGGAAGGGCTAATTTACTCCCAGAAGAGACAAGATATCCTA
GATCTGTGGGTCTATCACACACAAGGCTACTTCCCTGATTGGCAAAACTACACACCAGGGCCAGGGGTCAGAT
ATCCACTGACCTTTGGATGGTGCTTCAAGCTAGTACCAGTTGATCCAGACCAGGTAGAGAAGGCCAATGAAGG
GGAGAACAACAGCTTGCTGCACCCTATGAGCCTGCATGGGATAGAGGACCCGGAGAAAGAAGTGCTGATGTGG
AAGTTTGACAGCCGCCTAGCATTCAGTCACATGGCCCGAGAGCTGCATCCGGAGTACTACAAAAACTGATGAC
ACCGAGTTTCTACAAGGGACTTTCCGCTGGGGACTTTCCAGGGGAGGCGCGGCCTGGGCGGGACTGGGGAGTG
GCGAGCCCTCAGATGCTGCATATAAGCAGCTGCTTTCTGCCTGTACTGGGTCTCTCTGGTTAGACCAGATCTG
AGCCTGGGAGCTCTCTGGCTAACTAGGGAACCCACTGCTTAAGCCTCAATAAAGCTTGCCTTGAGTGCTTCAA
GTAGTGTGTGCCCGTCTGTTGTGTGACTCTGGTAACTAGAGATCCCTCAGACCTTTTAGTCAGTGTGGAAAA?
???????

>K-D1 HIV-1 genome, derived from RNA genomic sequence
???????????GCATGGATGGAGGACCCGGAGAAAGAAGTGCTGATGTGGAAGTTTAACAGCCGCCTAGCAT
TCAGTCACATGGCCCGAGAGCTGCATCCGGAGTACTACAAGAACTGATGACACCGAGTTTCTACAAGGGACTT
TCCGCTGGGGACTTTCCAGGGGAGGCGCGGCCTGGGCGGGACTGGGGAGTGGCGAGCCCTCAGATGCTGCATA
TAAGCAGCTGCTTTCTGCCTGTACTGGGTCTCTCTGGTTAGACCAGATCTGAGCCTGGGAGCTCTCTGGCTAA
CTAGGGAACCCACTGCTTAAGCCTCAATAAAGCTTGCCTTGAGTGCTTTAAGTAGTGTGTGCCCGTCTGTTGT
GTRACTCTGGTAACTAGAGATCCCTCAGACCCCTTTAGTCAGTGTGGAAAATCTCTAGCAGTGGCGCCCGAAC
AGGGACGCGAAAGCGAAAGTAAGACCGGAGGAGCTCTCTCGACGCAGGACTCGGCTTGCTGAAGCGCGCACGG
CAAGAGGCGAGGGGCGGCGACTGGTGAGTACGCCAAAATTTTTGACTAGCGGAGGCTAGAAGGAGAGAGATGG
GKGCGAGAGCGTCAGTATTAAGCGGGGGAGAATTAGAT??????????????????????????????????
???CAGTCAACCCTGGCCTG
TTAGAAACATCAGAAGGCTGTAGACAAATACTGGRACAGCTACAACCGTCCCTTCAGACAGGATCAGAAGAAC
TTAGATCATTATTTAATACAGTAGCAGTCCTCTATTGTGTACACCAAAGGATAGAGGTAAA???????????
??
??
??????????????????????????CATGGGTAAAAGTAGTAGAAGAAAAGGCTTTTAGCCCAGAAGTAAT
ACCCATGTTTGCAGCATTATCAGAAGGAGCCACCCCACAAGATTTAAACACCATGCTAAACACAGT???????
??
??
???CAGTAGGAGAAATTTATAAAAAATGGATA
ATCATGGGATTAAATAAAATAGTAAGAATGTATAGCCCTACCAGCATTCTGGACATAAG??????????????
??????????????ATGTAGACCGGTTCTATAAAACTCTAAGAGCTGAGCAAGCTTCACAGGAGGTAAAAAA
TTGGATGACAGAGACCTTATTGGTCCAAAATGCAAACCCAGATTGTAAGACTATCTTAAAAGCATTAGGACCA
GCAGCAACACTAGAAGAAATGATGACAGCATGTCAGGGAGTAGGGGGACCCGGMCATAAAGCAAGAGTTTTGG
CCGAGGCAATGAGCCAGGTAACAAATTCAGCTACCGTAATGATGCAGAAAGGCAACTTTAGGAACCAAAGAAA
GGTTGTCAAGTGTTTCAATT??
?GTTGGAAATGTGGAAAGGAAGGACACCAAATGAAGGATTGTGTTGAGAGACAGGCTAATTT???????????
???AGAGCCAACAGCCCACCAGAGGA
GAGCCTCAGGTTTGGGGAGGAGACAACAACTCCCTATCAGAAACAGGAGCAGATGGACAAGGAGAAATATCCT
TTKAATTCMCTCAGATCACTCTTTGGCAACGACCMCTTGTCACAATAAAAGTAGGAGGGCAACTAAAGGAAGM
TCWATTAGATACAGGAGCAGATGATACAGTATTAGAAGAAATGAATTTGCCAGGAAGATGGAAACCAAAAATG
ATAGGGGGAATTGGAGGTTTTATCAAAGTAAGACAATATGATCAGGTACCCATAGAAATCTGTGGGCATAAAG
CTATAGGTACAGTATTAGTAGGACCTACACCTGTCAACATAATTGGAAGAAATCTGTTGACTCAGCTTGGTTG
CACTTTAAATTTTCCCATTAGTCCTATTGAAACTGTACCAGTAAAATTAAAGCCAGGAATGGATGGCCCAAAA
GTTAAACAGTGGCCATTGACAGAGGAAAAAATAAAAGCATTAGTAGAAATTTGTACAGAAATGGAAAAGGAAG
GAAAAATTTCAAAAGTTGGGCCTGAAAATCCATACAATACTCCAGTATTTGCCATAAAGAAAAAAGATGGTAC
TAAGTGGAGAAAACTAGTAGATTTCAGAGAACTTAATAAGAGAACTCAAGACTTCTGGGAAGTTCAATTAGGA
ATACCACATCCTACAGAGTTAAAAAAAAAAAAAATCAGTAACAGTACTGGATATAAATAATACTTATTTTTCAG
TTCCCTTAAATAAAGACTTCAGAAAGTATACTACATTTACCATACCCAGTATAAATAA???????????????
????????????????????????????????TGGAAAGGATCCCCAGCAATATTCCAAAGTAGCATGACAAAA
ATCTTAGATCCTTTTAGAAAGCAAAATCCAGACATAGTTATTTACCAATACATGGATGATTTGTATGTAGGAT
CTGACTTAGAAATAGGGCAGCACAGAATAAAAATAGAGGAACTAAGACAACATCTGTTGAAGTGGGGATTCAC
CACACCAGACAAGAAACATCAGAAAGAACCTCCATTCCTTTGGATGGGTTATGAACTCCATCCTGATAAATGG
ACAGTACAGCCAGAAAAAGAC???
??
???????????????????AGCAGAACTAGAGCTGCAGAAAACAGGGAGATTCTAAAAACACCAGTGCATGGA
GTGTACTATGACCCATCAAAAGACTTAGTAGCAGAAATACAGAAGCAGGGGCAAGGTCAATGGACATATCAAA
TTTATCAAGAGCCATTTAAAAATCTGAAAACAGGAAAGTATGCAAAAATGAGGAGTGCCCACACTAATGATGT
AAAACAGTTAACAGAGGCAGTGCAAAAAATATCCACAGAAAGCATAATAATATGGGGAAAGACCCCTAAATTT
AAACTACCTATACAAAAGGAAACATGGGAAACATGGTGGGCGGAGTATTGGCAAGCCACCTGGATTCCTGAGT
GGGAATTTGTCAATACCCCTCCCTTAGTGAAACTATGGTACCAGTTAGAAAAAGAACCCATAGTAGGAGCAGA
AACTTTCTATGTAGATGGGGCAGCTAATAGAGAAACTAAATTAGGAAAAGCAGGGTATGTTACTGACAGAGGA
AGACAAAAAGTTGTCTCCCTAACTGACACAACAAATCAGAAAACTGAGTTACAAGCTATTGATCTAGCTTTGC
AGGATTCAGGGTTAGAAGTAAATATAGTAACAGACTCACAATATGCACTGGGAATCATTCAAGCACAACCAGA

TAAGAGTGAATCAGAGATAGTCAGTCATATAATAGAGC??????????????????GGGTCTACCTGGCATGG
GTACCAGCACACAAAGGAATTGGGGGAAATGAACAAGTAGATAAATTGGTCAGTAATGGAATCAGAAGAGTAC
TATTCTTAGATGGAATAGATAAGGCCCAAGAGGAGCATGAA????????????????????????????????
???
????????????????????????????????TGCATGGCAACTAGATTGTACACATCTAGAAGGAAAAATTA
TCATAGTAGCAGTTCATGTAGCCAGTGGATATATAGAGGCAGAAGTTATTCCAGCRGAGACAGGGCAGGAAAC
AGCATACTTTCTCTTAAAATTAGCAGGGAGATGGCCAGTAAAAACAATACATACAGATAATGGCCCCAATTTC
ATTAGCTCCGCGGTCAAGGCCGCCTGTTGGTGGGCAGGGATCAAGCAGGAATTTGGCATTCCCTACAATCCCC
AAAGTCAAGRAGTAGTAGAGTCTATGAA???
???GGGGGGATTGGGGGGTAC
AGTGCAGGGGAAAGAATAGTAGACATAATAGCAACAGACATACARACTAAAGAACTACAAAAACAAATTACAA
AAATTCAAAATTTTCGGGTTTATTACAGGGWCAGCAGAGATCCACTTTGGAAAGGACCAGCAAAGCTCCTTTG
GAAAGGTGAAGGGGCAGTAGTAATACAAGATAATAGTGATATAAAAGTAGTACCAAGAAGAAAAGCAAAGATC
ATTAGAGATTATGGAAAACAGATGGCAGGTGATGATTGTGTGGCAGGTGGACAGGATGAGGATTAGAACATGG
AAAAGTTTAGTAAAACACCACATGTATATTTCAAAGAAAGCTAAGGGATGGTTTTATAAACATCACTATGAAA
GCACTCATCCAAGAATAAGTTCAGAAGTACACATCCCATTAGGGGATGCTAAATTGGTAATAATAACATATTG
GGG??
??????CAAATAGACCCTGGCCTAGCAGACCAACTAATTCATATGCACTATTTTGATTGTTTTTCAGAATCTG
CTATAAGAAATGCCATATTAGGACGTATAGTTAGTCCTAGGTGTGAATATCAAGCAGGACATAACAAGGTAGG
ATCTCTACAATATTTGGCACTAACAGCATTAATAAAACCAAAAGGGAGAAAGCCACCTTTGCCTAGTGTAGCG
AAACTGACAGAGGATAGATGGAACAAGCCCCAGAGGACCAAGGGCCACAGAGGGAGCCATACAATGAATGGGC
ACTAGGGATTTTAGAGGAACTTAAAAATGAAGCTGTTAGACATTTTCCTGGGCCCTGGCTCCAGGGCTTAGGA
CAATACATTTATGT????????????????????????????????????AATAAGAACTCTGCAACAACTGC
TGTTTATTCATTTCAGAATTGGGTGTCGGCATAGCAGAATAGGCATTGACATCCGACAGAGGAGAGCAAGAAA
TGGATCCAGTAGATCCTAGATTAGAGCCCTGGAAGCATCCAGGAAGTCAGCCTAGGACTGCTTGTAATAGTTG
CTATTGTAAAAAGTGTTGCCTTCATTGCCAAGTTTGCTTCTTAAGAAAAGGCTTAGGCATCTCCTATGGCAGG
AAGAAGCGGAGACAGCGACGAAGAACTCCTTCAGA??????????????????????????????????????
???
????????????????TAGTTGTATGGACCATAGTAGCCATAGAATATAGAAAAATATTAAGACAAAGAAAAA
TAGACAGGTTAATTGAAAGAATAAGTGAAAGAGCAGAAGACAGTGGCAATGAGAGCGAGGGGGATCAGGAAGA
GTTGTCAGCACTTGTGGAGATGGGGCATCATGCTCCTTGGGATATTGATGATCTGTAGTGCTGGAGAACAATT
GTGGGTCACAGTCTATTATGGGGTACCTGTGTGGAAAGAAGCAACTACCACTCTATTTTGTGCCTCAGATGCT
AAAGCATATGATACAGAAAGTCATAATGTTTGGGCCACACATGCCTGTGTACCCACAGACCCCAACCCACAAG
AAGTAAGATTGAAAAATGTGACAGAAGAATTTAACATGGGAAAAAATAATATGGTAGATCAAATGCATGAGGA
TATAATCAGCTTATGGGAACAAAGCTTAAAGCCATGTGAAAA???????????????????????????????
???GACATAAAAA
ACTGTTCTTTCAATGTCACCACAGACATAAGAGACAGAATGCAGAAAGAATTTGCACTTTTTCACAAGCTTGA
TCTAGTACCAATAGATGATGATAATATTACTAATAAGAATAACTATAGTAACTATAGGTTGATAAGTTGCAAC
ACCTCAGTCATTAGACAGGCCTGTCCAAAGGTATCTTTTGAGCCAATTCCCATACATTATTGTGCCCCTGCTG
GTTTTGCGATTCTAAAGTGTAACGAGGAAAAGTTCAATGGAACAGGACTATGCAGCGATGTCAGCACAGTACA
ATGTACACATGGAATTAAACCAGTAGTGTCAACTCAACTGCTGTTAAATGGCAGCCTAGCAAAAGAAGAGGTA
GTACTTAGGTCTGAAAATTTCACAGAAAATACTAAAACCATAATAGTGCAGCTGAAGGACCCTGTAGTAATTA
ATTGTACAAGACCCAACAACAATATAAGAAAAAGGATACGTATAGGACCAGGGGGCGCATCATTTTATGCAAC
AAACATAATGGGAGATATAAGAAAAGCTTATTGTATCATTAATAGCACACAATGGAATAACACTTTACAACAG
GTAGTTAAAAAATTAAGAGAACAATTTGGAAATAAAACAATAGTCTTTAATCAATCCTCAGGAGGGGACCCAA
AAGTTGTAAGGTATAGTTTTAATTGTAGAAAGAAATTTTTCTACTGTGATTCCTCACAACTGTTTAATAGTAC
T?AAAATATTAGTAATATTAATAGTAATATTACTGGAAATAAAACAAATATCACACTCCCATGTAAAATAAAA
CAAATAATAAACATGTGGCAGGAAGTAAAAAAAGCAATGTATGCCCCTCCCATCAGTAAACTAATTAACTGTT
CATCAAATATTACAGGGCTGCTATTAACAAGAGACGGTGGTAAGGACGGTGATAATAGTACAGAGACTTTCAG
ACCAGGAGGAGGAAATATGAAGGACAATTGGAGAAGTGAATTATATAAATACAAAGTAGTAAAAATTGAACCA
TTAGGAATAGCACCCACCGAGGCAAAGAGAAGAGTGGTGCAGAGAGAAAAAAGAGCAATAGGATTAGGAGCTT
TGTTCCTTGGGTTCTTGGGAGCAGCAGGAAGCACTATGGGCGCAGCGTCAATGACGCTGACGGTACAGGCCAG
ACAATTATTGTCTGGTATAGTGCAACAGCAGAACAATCTGCTGAAGGCTATTGAGGCGCAACAGCATCTGTTG
CAACTCACAGTCTGGGGCATCAAGCAGCTCCAGGCAAGAGTCCTGGCTGTGGAAAGATACCTAAGAGATCAAC

AGCTCCTAGGGATTTGGGGTTGCTCTGGAAAACTCATCTGCACCACTAACGTGCCTTGGAATACTAGTTGGAG
TAATAAGACTAAGGATGAGATTTGGAATAACATGACCTGGATGCAGTGGGAAAAAGAGATTGACAATTACACA
GGCTTAATATACACTTTGCTTGAAGAATCACAGAACCAGCAGGAAAAGAATGAACAAGAATTATTGGCATTGG
ATAAGTGGGCAAGTTTGTGGACTTGGCTTGACATAACAAACTGGCTGTGGTATATAAAAATATTCATAATGAT
AGTAGGAGGCTTGATAGGCTTAAGAATAGTTTTTGCTGTGCTTTCTATAATAAATAGAGTTAGGCAGGGATAC
TCACCATTATCGTTGCAGACCCACCTCCCAGCTCCGAGGGGACTCGACAGGCCCGAAGGAATCGAAGAAGAAG
GTGGAGAGACAGACAGAGGCAGATCAATTCGATTAGTGGATGGCTTCTTAGCACTTTTCTGGGACGACCTGAG
GAACCTGTGCCTCTTCAGCTACCACCGCTTGAGAGACTTACTCTTGATTGTAACGAGGATTGTGGGACTTCTG
GGACGCAGGGGGTGGGAACTCCTGAAATATTGGTGGAATCTCCTGCAGTATTGGATTCAGGAACTAAAGAATA
GTGCTACTAGCTTGCTGAACGCCACAGCTATAGCAGTTGCTGAGGGGACAGATAGGATTATAGAAGTAGTACA
AAGAATTCTTAGAGCTATCCTTCACATACCTAGAAGAATAAGACAGGGC??ATAAGGGCTTTGCTATAAAATG
GGTGGCAAGTGGTCAAAGATGATTGGATGGCCTGCTGTAAGGGAAARAATGCATCRAGCTGAGCCAGCAGCAG
AAGGGGTAGGAGCAGCATCTCGAGACCTGGAGAGACATGGGGCACTCACAAGTAGCAATACAGCAGCTACTAA
TGCTGATTGTGCCTGGTTAGAAGCACAAGAGGATGAGGAGGTGGGCTTTCCAGTCAGACCCCAGGTACCTTTA
AGACCAATGACTTACAAGGGAGCGGTAGATCTCAGCCACTTTTTAAGAGAAAAGGGGGGACTGGAAGGGCTAA
TTTACTCCCAGAAGAGACAAGATATCCTAGATCTGTGGGTCTATCACACACAAGGCTACTTCCCTGATTGGCA
AAACTACACACCAGGGCCAGGGGTCAGATATCCACTGACCTTTGGATGGTGCTTCAAGCTAGTACCAGTTGAC
CCAGACCAGGTAGAGAAGGCCAATGAAGGGGAGAACAACAGCTTGCTGCACCCTATGAGCCTGCATGGGATGG
AGGACCCGGAGAAAGAAGTGCTGATGTGGAAGTTTGACAGCCGCCTAGCATTCAGTCACATGGCCCGAGAGCT
GCATCCGGAGTACTACAAGAACTGATGACACCGAGTTTCTACAAGGGACTTTCCGCTGGGGACTTTCCAGGGG
AGGCGCGGCCTGGGCGGGACTGGGGAGTGGCGAGCCCTCAGATGCTGCATATAAGCAGCTGCTTTCTGCCTGT
ACTGGGTCTCTCTGGTTAGACCAGATCTGAGCCTGGGAGCTCTCTGGCTAACTAGGGAACCCACTGCTTAAGC
CTCAATAAAGCTTGCCTTGAGTGCTTTAAGTAGTGTGTGCCCGTCTGTTGTGTGACTCTGGTAACTAGAGATC
CCTCAGACCCTTTAGTC???????????????????

>M-1A6 HIV-1 genome, derived from RNA genomic sequence
CGGCAAGCTTTATTGAGGCTTAAGCAGTGTGTGCCCGTCTGTTGTGTGACTCTGGTAACTAGAGATCCCTCAG
ACCCTTTTAGTCAGTGTGGAAAATCTCTAGCAGTGGCGCCCGAACAGGGACTTGAAAGCGAAAGGGAAACCAG
AGGAGCTCTCTCGACGCAGGACTCGGCTTGCTGAAGCGCGCACGGCAAGAGGCGAGGGGCGGCGACTGGTGAG
TACGCCAAAAATTTTGACTAGCGGAGGCTAGAAGGAGAGAGATGGGTGCGAGAGCGTCAGTTT?AAGCGGGGG
AGAATTAGATCGATGGGAAAAAATTCGGTTAAGGCCAGGGGGAAAGAAAAAATATAAATTAAAACATATAGTA
TGGGCAAGCAGGGAGCTAGAACGATTCGCAGTTAATCCTGGCCTGTTAGAAACATCAGAAGGCTGTAGACAAA
TACTGGGACAGCTACAACCATCCCTTCAGACAGGATCAGAAGAACTTAGATCATTATATAATACAGTAGCAAC
CCTCTATTGTGTGCATCAAAGGATAGAGATAAAAGACACCAAGGAAGCTTTAGACAAGATAGAGGAAGAGCAA
AACAAAAGTAAGAAAAAAGCACAGCAAGCAGCAGCTGACACAGGACACAGCAATCAGGTCAGCCAAAATTACC
CTATAGTGCAGAACATCCAGGGGCAAATGGTACATCAGGCCATATCACCTAGAACTTTAAATGCATGGGTAAA
AGTAGTAGAAGAGAAGGCTTTCAGCCCAGAAGTGATACCCATGTTTTCAGCATTATCAGAAGGAGCCACCCCA
CAAGATTTAAACACCATGCTAAACACAGTGGGGGGACATCAAGCAGCCATGCAAATGTTAAAAGAGACCATCA
ATGAGGAAGCTGCAGAATGGGATAGAGTGCATCCAGTGCATGCAGGGCCTATTGCACCAGGCCAGATGAGAGA
ACCAAGGGGAAGTGACATAGCAGGAACTACT??AGCTAGTACCCTTCAGGAACAAATAGGATGGATGACAAAT
AATCCACCTATCCCAGTAGGAGAAATTTATAAAAGATGGATAATCCTGGGATTAAATAAAATAGTAAGAATGT
ATAGCCCTACCAGCATTCTGGACATAAGACAAGGACCAAAAGAACCCTTTAGAGACTATGTAGACCGGTTCTA
TAAAACTCTAAGAGCCGAGCAAGCTTCACAGGAGGTAAAAAATTGGATGACAGAAACCTTGTTGGTCCAAAAT
GCGAACCCAGATTGTAAGACTATTTTAAAAGCATTGGGACCAGCGGCTACACTAGAAGAAATGATGACAGCAT
GTCAGGGAGTAGGAGGACCCGGCCATAAGGCAAGAGTTTTGGCTGAAGCAATGAGCCAAGTAACAAATTCAGC
TACCATAATGATGCAGAGAGGCAATTTTAGGAACCAAAGAAAGATTGTTAAGTGTTTCAATTGTGGCAAAGAA
GGGCACACAGCCAGAAATTGCAGGGCCCCTAGGAAAAAGGGCTGTTGGAAATGTGGAAAGGAAGGACACCAAA
TGAAAGATTGTACTGAGAGACAGGCTAATTTTTTAGGGAAGATCTGGCCTTCCCACAAGGGAAGGCCAGGGAA
TTTTCTTCAGAGCAGACCAGAGCCAACAGCCCCACCAGAAGAGAGCTTCAGGTTTGGGGAAGAGACAACAACT
CCCTCTCAGAAGCAGGAGCCGATAGACAAGGAACTGTATCCTTTAGCTTCCCTCAGATCACTCTTTGGCAGCG
ACCCCTCGTCACAATAAAGATAGGGGGGCAATTAAAGGAAGCTCTATTAGATACAGGAGCAGATGATACAGTA
TTAGAAGAAATGAATTTGCCAGGAAGATGGAAACCAAAAATGATAGGGGGAATTGGAGGTTTTATCAAAGTAA
GACAGTATGATCAGATACTCATAGAAATCTGCGGACATAAAGCTATAGGTACAGTATTAGTAGGACCTACACC
TGTCAACATAATTGGAAGAAATCTGTTGACTCAGATTGGCTGCACTTTAAATTTTCCCATTAGTCCTATTGAG
ACTGTACCAGTAAAATTAAAGCCAGGAATGGATGGCCCAAAAGTTAAACAATGGCCATTGACAGAAGAAAAAA
TAAAAGCATTAGTAGAAATTTGTACAGAAATGGAAAAGGAAGGAAAAATTTCAAAAATTGGGCCTGAAAATCC
ATACAATACTCCAGTATTTGCCATAAAGAAAAAAGACAGTACTAAATGGAGAAAATTAGTAGATTTCAGAGAA
CTTAATAAGAGAACTCAAGATTTCTGGGAAGTTCAATTAGGAATACCACATCCTGCAGGGTTAAAACAGAAAA
AATCAGTAACAGTACTGGATGTGGGCGATGCATATTTTTCAGTTCCCTTAGATAAAGACTTCAGGAAGTATAC
TGCATTTACCATACCTAGTATAAACAATGAGACACCAGGGATTAGATATCAGTACAATGTGCTTCCACAGGGA
TGGAAAGGATCACCAGCAATATTCCAGTGTAGCATGACAAAAATCTTAGAGCCTTTTAGAAAACAAAATCCAG
ACATAGTCATCTATCAATACATGGATGATTTGTATGTAGGATCTGACTTAGAAATAGGGCAGCATAGAACAAA
AATAGAGGAACTGAGACAACATCTGTTGAGGTGGGGATTTACCACACCAGACAAAAAACATCAGAAAGAACCT
CCATTCCTTTGGATGGGTTATGAACTCCATCCTGATAAATGGACAGTACAGCCTATAGTGCTGCCAGAAAAGG
ACAGCTGGACTGTCAATGACATACAGAAATTAGTGGGAAAATTGAATTGGGCAAGTCAGATTTATGCAGGGAT
TAAAGTAAGGCAATTATGTAAACTTCTTAGGGGAACCAAAGCACTAACAGAAGTAGTACCACTAACAGAAGAA
GCAGAGCTAGAACTGGCAGAAAACAGGGAGATTCTAAAAGAACCGGTACATGGAGTGTATTATGACCCATCAA
AAGACTTAATAGCAGAAATACAGAAGCAGGGGCAAGGCCAATGGACATATCAAATTTATCAAGAGCCATTTAA
AAATCTGAAAACAGGAAAGTATGCAAGAATGAAGGGTGCCCACACTAATGATGTGAAACAATTAACAGAGGCA
GTACAAAAAATAGCCACAGAAAGCATAGTAATATGGGGAAAGACTCCTAAATTTAAATTACCCATACAAAAGG
AAACATGGGAAGCATGGTGGACAGAGTATTGGCAAGCCACCTGGATTCCTGAGTGGGAGTTTGTCAATACCCC
TCCCTTAGTGAAGTTATGGTACCAGTTAGAGAAAGAACCCATAATAGGAGCAGAAACTTTCTATGTAGATGGG
GCAGCCAATAGGGAAACTAAATTAGGAAAAGCAGGATATGTAACTGACAGAGGAAGACAAAAAGTTGTCCCCC
TAACGGACACAACAAATCAGAAGACTGAGTTACAAGCAATTCATCTAGCTTTGCAGGATTCGGGATTAGAAGT
AAACATAGTGACAGACTCACAATATGCATTGGGAATCATTCAAGCACAACCAGATAAGAGTGAATCAGAGTTA
GTCAGTCAAATAATAGAGCAGTTAATAAAAAAGGAAAAAGTCTACCTGGCATGGGTACCAGCACACAAAGGAA
TTGGAGGAAATGAACAAGTAGATAAATTGGTCAGTGCTGGAATCAGGAAAGTACTATTTTTAGATGGAATAGA
TAAGGCCCAAGAAGAACATGAGAAATATCACAGTAATTGGAGAGCAATGGCTAGTGATTTTAACCTACCACCT
GTAGTAGCAAAAGAAATAGTAGCCAGCTGTGATAAATGTCAGCTAAAAGGGGAAGCCATGCATGGACAAGTAG

ACTGTAGCCCAGGAATATGGCAGCTAGATTGTACACATTTAGAAGGAAAAGTTATCTTGGTAGCAGTTCATGT
AGCCAGTGGATATATAGAAGCAGAAGTAATTCCAGCAGAGACAGGGCAAGAAACAGCATACTTCCTCTTAAAA
TTAGCAGGAAGATGGCCAGTAAAAACAGTACATACAGACAATGGCAGCAATTTCACCAGTACTACAGTTAAGG
CCGCCTGTTGGTGGGCGGGGATCAAGCAGGAATTTGGCATTCCCTACAATCCCCAAAGTCAAGGAGTAATAGA
ATCTATGAATAAAGAATTAAAGAAAATTATAGGACAGGTAAGAGATCAGGCTGAACATCTTAAGACAGCAGTA
CAAATGGCAGTATTCATCCACAATTTTAAAAGAAAAGGGGGGATTGGGGGGTACAGTGCAGGGGAAAGAATAG
TAGACATAATAGCAACAGACATACAAACTAAAGAATTACAAAAACAAATTACAAAAATTCAAAATTTTCGGGT
TTATTACAGGGACAGCAGAGATCCAGTTTGGAAAGGACCAGCAAAGCTCCTCTGGAAAGGTGAAGGGGCAGTA
GTAATACAAGATAATAGTGACATAAAAGTAGTGCCAAGAAGAAAAGCAAAGATCATCAGGGATTATGGAAAAC
AGATGGCAGGTGATGATTGTGTGGCAAGTAGACAGGATGAGGATTAACACATGGAAAAGATTAGTAAAACACC
ATT?GTATATTTCAAGGAAAGCTAAGGACTGGTTTTATAGACATCACTATGAAAGTACTAATCCAAAAATAAG
TTCAGAAGTACACATCCCACTAGGGGATGATAAATTGGTGATAACAACATATTGGGGTCTGCATGCAGGAGAA
AGG???
??
??
??
??
??
??
??????????????????????????TATTCTACCAAGGAGAGCAAGGAATGGAGCCAGTAGATCCTAGT
TTAGAGCCCTGGAAGCATCCAGGAAGCCGGCCTAAAACTGCTTGTACCAATTGTTATTGTAAAAAGTGTTGCT
TTCATTGCCAAGTGTGTTTCACAACCAAAGCCTTAGGCATCTCCTATGGCAGGAAGAAGCGGAGACAGCGACG
AAGAGCTCCTCCAAGCAGTCAGAATCATCAAGTTTCTCTACCAAAGCAGTGAGTAATATATGTAATGCAATCC
TTAGAAATATATGC???????????????????????????????????????AAGGAAAATAGACAGAT
TAATTGATAGAATAAGAGAAAGAGCAGAAGACAGTGGCAATGAAAGTGAAGGGGATGAGGAGGAATTATCAGC
CCTTGTGGAAATGGGGCATCATGCTCCTTGGGATGTTGATGATCTGTAGTGCTACAGAAAAATTGTGGGTCAC
AGTCTATTATGGGGTACCTGTGTGGAAAGAAGCAACCACCACTTTATTTTGTGCATCAGATGCTAAAGCATAT
CATACAGAGATGCATAATGTTTGGGCCACACATGCCTGTGTACCCACAGACCCTAGCCCACAAGAAGTAGTAT
TGGGAAATGTGACAGAAAATTTTAACATGTGGAAAAATAACATGGTAGAACAGATGCATGAAGATATAATCAG
TTTGTGGGATCAAAGCCTAAAGCCATGTGTAAAATTAACCCCACTCTGTGTCACTTTAAATTGCACTGATGGA
ATACAAG?TAGCTATACATTGATAAATTGTAACACCTCAGTCATTACACAGGCCTGTCCAAAGGTATCCTTTG
AACCAATTCCCATACATTATTGTACCCCGGCTGGTTTTGCAATTCTAAAGTGTAATGATAATAAGTTCAATGG
AACAGGACCATGTACTAATGTTAGCACAGTACAATGTACACATGGAATTAAGCCAGTAGTGTCAACTCAACTG
CTGTTGAATGGCAGTCTGGCAGAAGGAGGAGAGGTAGTAATTAGATCTGAAAATTTCACAAACAATGCTAAAA
CCATAATAGTACAGCTGAATACATCTGTAGAAATTAATTGTATAAGACCCAACAACAATACAAGAAAAAGTAT
AACTATAGGACCAGGGAGAGCATTTTATACAACAGACATAATAGGAGATATAAGACAAGCACATTGTAACCTT
AGTAGAGCAAAATGGAATGACACTTTAAAACAGATAGTTACAAAATTAAAAGAACAATTTGAGAACAAGACAA
TAGTCTTTAATCAATCTTCAGGAGGGGACCCAGAAATTGTAATGCACAGCTTTAATTGTGGAGGGGAATTTTT
CTACTGTAATACAACACAGCTGTTTAACAGTACTTGGAATGGTACTGACTGGAATGACACTACAGGGTTAGAG
AACATCACACTCCCATGCAGAATAAAACAAATTGTAAACAGGTGGCAGGAAGTAGGAAAAGCAATGTATGCCC
CTCCCATCAAAGGACAAATTAGATGTTCATCAAATATTACAGGGCTACTATTAACAAGAGATGGGGGAAACAG
TAGTGAGATGACCGAGATCTTCAGACCTGGAGGAGGAGATATGAGGGACAATTGGAGAAGTGAATTATATAAA
TATAAAGTAGTAAAAATTGAACCATTAGGAGTAGCACCCACCAAGGCAAAGAGAAGAGTGGTGCAGAGAGAAA
AAAGAGCAGTGGGAATAGGAGCTTTGTTCCTTGGGTTCTTGGGAGCAGCAGGAAGCACTATGGGCGCAGCGTC
AATGACGCTGACGGTACAGGCCAGACAATTATTGTCTGGTATAGTGCAGCAGCAGAACAATTTGCTGAGCTTT
ATTGAGGCGCAACAGCATCTGTTGCAACTCACAGTCTGGGGCATCAAGCAGCTCCAGGCAAGAATCCTGGCTG
TGGAAAGATACCTAAAGGATCAACAGCTCCTGGGGATTTGGGGTTGCTCTGGAAAACTCATTTGCACCACTGC
TGTGCCTTGGAATGCTAGTTGGAGTAATAAATCTCTGGAACAGATTTGGAATCACACGACCTGGATGGAGTGG
GACAGAGAAATTAACAATTACACAAGCTTAATACACTCCTTAATTGAAGAATCGCAAAACCAGCAAGAAAAGA
ATGAACAAGAATTATTGGAATTAGATAAATGGGCAAGTTTGTGGAATTGGTTTAACATAACAAATTGGCTGTG
GTATATAAAATTATTCATAATGATAGTAGGAGGCTTGGTAGGTTTAAGAATAGTTTTTGCTGTACTTTCTATA
GTGAATAGAGTTAGGCAGGGATATTCACCATTATCGTTTCAGACCCACCTCCCAACCCCGAGGGGACCCGACA
GGCCCGAAGGAATA?AAGAAGAAGGTGGAGAGAGAGACAGAGACAGATCCATTCGATTAGTGAACGGATCTCG
ACGGTATCGCCGAATTCACAAATGGCGCCTGTGCCTCTTCAGCTACCACCGCTTGAGAGACTTACTCTTGATT

GTAGCGAGGATTGTGGAACTTCTGGGACGCAGGGGGTGGGAGGCCCTGAAATATTGGTGGAATCTCCTGCAGT
ATTGGAGTCAGGAACTAAAGAATAGTGCTGTTAGTCTGCTTAATGCCACAGCTATAGCAGTAGCTGAGGGGAC
AGATAGGATTATAGAAGTATTACAAAGAGCTTGTAGAGCTATTCTCCACATACCTAGAAGAATAAGACAGGGC
TTAGAAAGGGCTTTGCTATAAGATGGGTGGTAAGTGGTCAAAACGTCGTGCGGGTGGATGGGAAGCTGTAAGG
GAAAAAATAAGACAAACTGAGCCCTGGGGAGCAGGAAATATCTGTGGGCTTGTGACTCGAGACCTAGAAAAAC
ATGGAGCAATCACAAGTAGCAATACAGCAGCTACCAATGCTGATTGTGCCTGGCTAGAAGCACAAGAGGAGGA
GGAGGTGGGTTTTCCAGTCACACCTCAGGTACCTTTAAGACCAATGACTTACAAGGCAGCTGTAGATCTTAGC
CACTTTTTAAAAGAAAAGGGGGGACTGGAAGGGCTAATTCACTCCCAACGAAGACAAAATCGTCGAGAGATGC
TGCATTACAACACACAAGGCTATTTCCCTGATTGGCAGAACTACACACCAGGGCCAGGGGAGAGATTTCCCCT
GACCTTTGGATGGTGCTTCAAGCTAGTACCAGTTGATCCAGATCAGGTAGAAGAGGCTAATAAAGGAGAGACC
AACAGCTTGTTACACCCTATGAGCCAGCATGGGATAGAGGACCCGGAG

```
>M-1D1 HIV-1 genome, derived from RNA genomic sequence
AATAAAGCTTGCCTTGAGTGCTTCAAGTAGTGTGTGCCCGTCTGTTGTGTGACTCTGGTAACTAGAGATCCCT
CAGACCCTTTTAGTCAGTGTGGAAAATCTCTAGCAGTGGCGCCCGAACAGGGACTTGAAAGCGAAAGGGAAAC
CAGAGGAGCTCTCTCGACGCAGGACTCGGCTTGCTGAAGCGCGCACGGCAAGAGGCGAGGGGCGGCGACTGGT
GAGTACGCCAAAAATTTTGACTAGCGGAGGCTAGAAGGAGAGAGATGGGTGCGAGAGCGTCAGTTT?AAGCGG
GGGAGAATTAGATCGATGGGAAAAAATTCGGTTAAGGCCAGGGGGAAAGAAAAAATATAAATTAAAACATATA
GTATGGGCAAGCAGGGAGCTAGAACGATTCGCAGTTAATCCTGGCCTGTTAGAAACATCAGAAGGCTGTAGAC
AAATACTGGGACAGCTACAACCATCCCTTCAGACAGGATCAGAAGAACTTAGATCATTATATAATACAGTAGC
AACCCTCTATTGTGTGCATCAAAGGATAGAGATAAAAGACACCAAGGAAGCTTTAGACAAGATAGAGGAAGAG
CAAAACAAAAGTAAGAAAAAAGCACAGCAAGCAGCAGCTGACACAGGACACAGCAATCAGGTCAGCCAAAATT
ACCCTATAGTGCAGAACATCCAGGGGCAAATGGTACATCAGGCCATATCACCTAGAACTTTAAATGCATGGGT
AAAAGTAGTAGAAGAGAAGGCTTTCAGCCCAGAAGTGATACCCATGTTTTCAGCATTATCAGAAGGAGCCACC
CCACAAGATTTAAACACCATGCTAAACACAGTGGGGGGACATCAAGCAGCCATGCAAATGTTAAAAGAGACCA
TCAATGAGGAAGCTGCAGAATGGGATAGAGTGCATCCAGTGCATGCAGGGCCTATTGCACCAGGCCAGATGAG
AGAACCAAGGGGAAGTGACATAGCAGGAACTACT??AGCTAGTACCCTTCAGGAACAAATAGGATGGATGACA
AATAATCCACCTATCCCAGTAGGAGAAATTTATAAAAGATGGATAATCCTGGGATTAAATAAAATAGTAAGAA
TGTATAGCCCTACCAGCATTCTGGACATAAGACAAGGACCAAAAGAACCCTTTAGAGACTATGTAGACCGGTT
CTATAAAACTCTAAGAGCCGAGCAAGCTTCACAGGAGGTAAAAAATTGGATGACAGAAACCTTGTTGGTCCAA
AATGCGAACCCAGATTGTAAGACTATTTTAAAAGCATTGGGACCAGCGGCTACACTAGAAGAAATGATGACAG
CATGTCAGGGAGTAGGAGGACCCGGCCATAAGGCAAGAGTTTTGGCTGAAGCAATGAGCCAAGTAACAAATTC
AGCTACCATAATGATGCAGAGAGGCAATTTTAGGAACCAAAGAAAGATTGTTAAGTGTTTCAATTGTGGCAAA
GAAGGGCACACAGCCAGAAATTGCAGGGCCCCTAGGAAAAAGGGCTGTTGGAAATGTGGAAAGGAAGGACACC
AAATGAAAGATTGTACTGAGAGACAGGCTAATTTTTTAGGGAAGATCTGGCCTTCCCACAAGGGAAGGCCAGG
GAATTTTCTTCAGAGCAGACCAGAGCCAACAGCCCCACCAGAAGAGAGCTTCAGGTTTGGGGAAGAGACAACA
ACTCCCTCTCAGAAGCAGGAGCCGATAGACAAGGAACTGTATCCTTTAGCTTCCCTCAGATCACTCTTTGGCA
GCGACCCCTCGTCACAATAAAGATAGGGGGGCAATTAAAGGAAGCTCTATTAGATACAGGAGCAGATGATACA
GTATTAGAAGAAATGAATTTGCCAGGAAGATGGAAACCAAAAATGATAGGGGGAATTGGAGGTTTTATCAAAG
TAAGACAGTATGATCAGATACTCATAGAAATCTGCGGACATAAAGCTATAGGTACAGTATTAGTAGGACCTAC
ACCTGTCAACATAATTGGAAGAAATCTGTTGACTCAGATTGGCTGCACTTTAAATTTTCCCATTAGTCCTATT
GAGACTGTACCAGTAAAATTAAAGCCAGGAATGGATGGCCCAAAAGTTAAACAATGGCCATTGACAGAAGAAA
AAATAAAAGCATTAGTAGAAATTTGTACAGAAATGGAAAAGGAAGGAAAAATTTCAAAAATTGGGCCTGAAAA
TCCATACAATACTCCAGTATTTGCCATAAAGAAAAAAGACAGTACTAAATGGAGAAAATTAGTAGATTTCAGA
GAACTTAATAAGAGAACTCAAGATTTCTGGGAAGTTCAATTAGGAATACCACATCCTGCAGGGTTAAAACAGA
AAAAATCAGTAACAGTACTGGATGTGGGCGATGCATATTTTTCAGTTCCCTTAGATAAAGACTTCAGGAAGTA
TACTGCATTTACCATACCTAGTATAAACAATGAGACACCAGGGATTAGATATCAGTACAATGTGCTTCCACAG
GGATGGAAAGGATCACCAGCAATATTCCAGTGTAGCATGACAAAAATCTTAGAGCCTTTTAGAAAACAAAATC
CAGACATAGTCATCTATCAATACATGGATGATTTGTATGTAGGATCTGACTTAGAAATAGGGCAGCATAGAAC
AAAAATAGAGGAACTGAGACAACATCTGTTGAGGTGGGGATTTACCACACCAGACAAAAAACATCAGAAAGAA
CCTCCATTCCTTTGGATGGGTTATGAACTCCATCCTGATAAATGGACAGTACAGCCTATAGTGCTGCCAGAAA
AGGACAGCTGGACTGTCAATGACATACAGAAATTAGTGGGAAAATTGAATTGGGCAAGTCAGATTTATGCAGG
GATTAAAGTAAGGCAATTATGTAAACTTCTTAGGGGAACCAAAGCACTAACAGAAGTAGTACCACTAACAGAA
GAAGCAGAGCTAGAACTGGCAGAAAACAGGGAGATTCTAAAAGAACCGGTACATGGAGTGTATTATGACCCAT
CAAAAGACTTAATAGCAGAAATACAGAAGCAGGGGCAAGGCCAATGGACATATCAAATTTATCAAGAGCCATT
TAAAAATCTGAAAACAGGAAAGTATGCAAGAATGAAGGGTGCCCACACTAATGATGTGAAACAATTAACAGAG
GCAGTACAAAAAATAGCCACAGAAAGCATAGTAATATGGGGAAAGACTCCTAAATTTAAATTACCCATACAAA
AGGAAACATGGGAAGCATGGTGGACAGAGTATTGGCAAGCCACCTGGATTCCTGAGTGGGAGTTTGTCAATAC
CCCTCCCTTAGTGAAGTTATGGTACCAGTTAGAGAAAGAACCCATAATAGGAGCAGAAACTTTCTATGTAGAT
GGGGCAGCCAATAGGGAAACTAAATTAGGAAAAGCAGGATATGTAACTGACAGAGGAAGACAAAAAGTTGTCC
CCCTAACGGACACAACAAATCAGAAGACTGAGTTACAAGCAATTCATCTAGCTTTGCAGGATTCGGGATTAGA
AGTAAACATAGTGACAGACTCACAATATGCATTGGGAATCATTCAAGCACAACCAGATAAGAGTGAATCAGAG
TTAGTCAGTCAAATAATAGAGCAGTTAATAAAAAAGGAAAAAGTCTACCTGGCATGGGTACCAGCACACAAAG
GAATTGGAGGAAATGAACAAGTAGATAAATTGGTCAGTGCTGGAATCAGGAAAGTACTATTTTTAGATGGAAT
AGATAAGGCCCAAGAAGAACATGAGAAATATCACAGTAATTGGAGAGCAATGGCTAGTGATTTTAACCTACCA
CCTGTAGTAGCAAAAGAAATAGTAGCCAGCTGTGATAAATGTCAGCTAAAAGGGGAAGCCATGCATGGACAAG
```

TAGACTGTAGCCCAGGAATATGGCAGCTAGATTGTACACATTTAGAAGGAAAAGTTATCTTGGTAGCAGTTCA
TGTAGCCAGTGGATATATAGAAGCAGAAGTAATTCCAGCAGAGACAGGGCAAGAAACAGCATACTTCCTCTTA
AAATTAGCAGGAAGATGGCCAGTAAAAACAGTACATACAGACAATGGCAGCAATTTCACCAGTACTACAGTTA
AGGCCGCCTGTTGGTGGGCGGGGATCAAGCAGGAATTTGGCATTCCCTACAATCCCCAAAGTCAAGGAGTAAT
AGAATCTATGAATAAAGAATTAAAGAAAATTATAGGACAGGTAAGAGATCAGGCTGAACATCTTAAGACAGCA
GTACAAATGGCAGTATTCATCCACAATTTTAAAAGAAAAGGGGGGATTGGGGGGTACAGTGCAGGGGAAAGAA
TAGTAGACATAATAGCAACAGACATACAAACTAAAGAATTACAAAAACAAATTACAAAAATTCAAAATTTTCG
GGTTTATTACAGGGACAGCAGAGATCCAGTTTGGAAAGGACCAGCAAAGCTCCTCTGGAAAGGTGAAGGGGCA
GTAGTAATACAAGATAATAGTGACATAAAAGTAGTGCCAAGAAGAAAAGCAAAGATCATCAGGGATTATGGAA
AACAGATGGCAGGTGATGATTGTGTGGCAAGTAGACAGGATGAGGATTAACACATGGAAAAGATTAGTAAAAC
ACCATTA?TATATTTCAAGGAAAGCTAAGGACTGGTTTTATAGACATCACTATGAAAGTACTAATCCAAAAAT
AAGTTCAGAAGTACACATCCCACTAGGGGATGCTAAATTAGTAATAACAACATATTGGGGTCTGCATACAGGA
GAAAGAGACTGGCATTTGGGTCAGGGAGTCTCCATAGAATGGAGGAAAAAGAGATATAGCACACAAGTAGACC
CTGACCTAGCAGACCAACTAATTCATCTGCACTATTTTGATTGTTTTTCAGAATCTGCTATAAGAAATACCAT
ATTAGGACGTATAGTTAGTCCTAGGTGTGAATATCAAGCAGGACATAACAAGGTAGGATCTCTACAGTACTTG
GCACTAGCAGCATTAATAAAACCAAAACAGATAAAGCCACCTTTGCCTAGTGTTAGGAAACTGACAGAGGACA
GATGGAACAAGCCCCAGAAGACCAAGGGCCACAGAGGGAGCCATACAATGAATGGACACTAGAGCTTTTAGAG
GAATTCTGCAACAACTGCTGTTTATCCATTTCAGAATTGGGTGTCCAT?GCTTAGGACAATATATCTATGAAA
CTTA?TGGCCATACAATGAATGGACACTAGAGCTTTTAGAG?GAATTCTGCAACAACTGCTGTTTATCCATTT
CAGAATTGGGTGTCGACATAGCAGAATAGGCGTTACTCGACAGAGGAGAGCAAGAATGGAGCCAGTAGATCCT
AGACTAGAGCCCTGGAAGCATCCAGGAAGTCAGCCTAAAACTGCTTGTACCAATTGCTATTGTAAAAAGTGTT
GCTTTCATTGCCAAGTTTGTTTCATGACAAAAGCCTTAGGCATCTCCTATGGCAGGAAGAAGCGGAGACAGCG
ACGAAGAGCTCATCAGAACAGTCAGACTCATCAAGCTTCTCTATCAAAGCAGTAAGTAGTACATGTAATGC??
TTTCTTTCAATAGTTGTGTGGTCCATAGTAATCATAGAATATAGGAAAATATTAAGACAAAGAAAAATAGACA
GGTTAATTGATAGACTAATAGAAAGAGCAGAAGACAGTGGCAATGAGAGTGAAGGAGAAGTGTCAGCACTTGT
GGAGATGGGGGTGGAGATGGGGCACCATGCTCCTTGGGATATTGATGATCTGTAGTGCTACAGAAAAATTGTG
GGTCACAGTCTATTATGGGGATCTTCAGACCTGGGAAGCAACCACCACTTTATTTTGTGCATCAGATGCTAAA
GCATATGATACAGAGATGCATAATGTTTGGGCCACACATGCCTGTGTACCCACAGACCCTAGCCCACAAGAAG
TAGTATTGG??
???
???
???
???
??????????????AATTCCCATACATTATTGTACCCCGGCTGGTTTCGCGATTCTTAAATGTAATGATAATA
AGTTCAATGGAACAGGACCATGTACTAATGTTAGCACAGTACAATGTACACATGGAATTAAGCCAGTAGTGTC
AACTCAACTGCTGTTGAATGGCAGTCTGGCAGAAGGAGGAGAGGTAGTAATTAGATCTGAAAATTTCACAAAC
AATGCTAAAACCATAATAATACAGCTGAATACATCTGTAG?????????????????????????????????
???
??GTTACAAAATTAAAAGAACAATTTGAG
AACAAGACAATAGTCTTTAATCAATCTTCAGGAGGGGACCCAGAAATTGTAATGCACAGCTTTAATTGTGGAG
GGGAATTTTTCTACTGCAATACAACACAGCTGTTTAACAGTACTTGGAATGGTACTGCCTGGAATGATACTAC
AGGGTCAGAGAACATCACACTCCCATGCAGAATAAAACAAATTGTAAACAGGTGGCAGGAAGTAGGAAAAGCA
ATGTATGCCCCTCCCATCAAAGGACAAATTAGATGTTCATCAAATATTACAGGGCTACTATTAA?GAAAAATT
GTGGGTCACAGTCTATTATGGGGATCTTCAGACCTGGAGGAGGAGATATGAGGGACAATTGGAGAAGTGAATT
ATATAAATATAAAGTAGTAAAAATTGAACCATTAGGAGTAGCACCCACCAAGGCAAAGAGAAGAGTGGTGCAG
AGAGAAAAAAGAGCAGTGGGAATAGGAGCTTTGTTCCTTGGGTTCTTGGGAGCAGCAGGAAGCACTATGGGCG
CAGCGTCAATGACGCTGACGGTACAGGCCAGACAATTATTGTCTGGTATAGTGCAGCAGCAGAACAATTTGCT
GAGCTTTATTGAGGCGCAACAGCATCTGTTGCAACTCACAGTCTGGGGCATCAAGCAGCTCCAGGCAAGAATC
CTGGCTGTGGAAAGATACCTAAAGGATCAACAGCTCCTGGGGATTTGGGGTTGCTCTGGAAAACTCATTTGCA
CCACTGCTGTGCCTTGGAATGCTAGTTGGAGTAATAAATCTCTGGAACAGATTTGGAATCACACGACCTGGAT
GGAGTGGGACAGAGAAATTAACAATTACACAAGCTTAATACACTCCTTAATTGAAGAATCGCAAAACCAGCAA
GAAAAGAATGAACAAGAATTATTGGAATTAGATAAATGGGCAAGTTTGTGGAATTGGTTTAACATAACAAATT
GGCTGTGGTATATAAAATTATTCATAATGATAGTAGGAGGCTTGGTAGGTTTAAGAATAGTTTTTGCTGTACT
TTCTATAGTGAATAGAGTTAGGCAGGATATTCACCATTATCGTTTCAGACCCACCTCCCAACCCCGAGGGGA

CCCGACAGGCCCGAAGGAATA?AAGAAGAAGGTGGAGAGAGAGACAGAGACAGATCCATTCGATTAGTGAACG
GATCTCGACGGTATCGCCGAATTCACAAATGGCAGCTGTGCCTCTTCAGCTACCACCGCTTGAGAGACTTACT
CTTGATTGTAGCGAGGATTGTGGAACTTCTGGGACGCAGGGGGTGGGAGGCCCTGAAATATTGGTGGAATCTC
CTGCAGTATTGGAGTCAGGAACTAAAGAATAGTGCTGTTAGTCTGCTTAATGCCACAGCTATAGCAGTAGCTG
AGGGGACAGATAGGATTATAGAAATATTACAAAGAGCTGGTAGAGCTATTCTCCACATACCTAGAAGAATAAG
ACAGGGCTTAGAAAGGGCTTTGTTATAAGATGGGTGGTAAGTGGTCAAAACGTCGTGCGGGTGGATGGGAAGC
TGTAAGGGAAAAAATAAGACAAACGATCCACTGGGGAGCAGGAAATATCTGTGGGCTTGTGACTCGAGACCTA
GAAAAACATGGAGCAATCACAAGTAGCAATACAGCAGCTACCAATGCTGATTGTGCCTGGCTAGAAGCACAAG
AGGAGGAGGAGGTGGGTTTTCCAGTCACACCTCAGGTACCTTTAAGACCAATGACTTACAAGGCAGCTGTAGA
TCTTAGCCACTTTTTAAAAGAAAAGGGGGGACTGGAAGGGCTAATTCACTCCCAACGAAGACAAAATCGTCGA
GAGATGCTGCATATAAGCAGCCAAGGCTATTTCCCTGATTGGCAGAACTACACACCAGGGCCAGGGGAGAGAT
TCCCACTGACCTTTGGATGGTGCTTCAAGTTAGTACCAGTTGATCCAGATCAGGTAGAAGAGGCTAATAAAGG
AGAGAACAACAGCTTGTTACACCCTATGAGCCAGCATGGGATGGAGGACCCGGAG

>M-2B5 HIV-1 genome, derived from RNA genomic sequence
AGGCAAGCTTTATTGAGGCTTAAGCAGTGTGTGCCCGTCTGTTGTGTGACTCTGGTAACTAGAGATCCCTCAG
ACCCTTTTAGTCAGTGTGGAAAATCTCTAGCAGTGGCGCCCGAACAGGGACTTGAAAGCGAAAGGGAAACCAG
AGGAGCTCTCTCGACGCAGGACTCGGCTTGCTGAAGCGCGCACGGCAAGAGGCGAGGGGCGGCGACTGGTGAG
TACGCCAAAAATTTTGACTAGCGGAGGCTAGAAGGAGAGAGATGGGTGCGAGAGCGTCAGTATT?AGCGGGGG
AGAATTAGATCGATGGGAAAAAATTCGGTTAAGGCCAGGGGGAAAGAAAAAATATAAATTAAAACATATAGTA
TGGGCAAGCAGGGAGCTAGAACGATTCGCAGTTAATCCTGGCCTGTTAGAAACATCAGAAGGCTGTAGACAAA
TACTGGGACAGCTACAACCATCCCTTCAGACAGGATCAGAAGAACTTAGATCATTATATAATACAGTAGCAAC
CCTCTATTGTGTGCATCAAAGGATAGAGATAAAAGACACCAAGGAAGCTTTAGACAAGATAGAGGAAGAGCAA
AACAAAAGTAAGAAAAAAGCACAGCAAGCAGCAGCTGACACAGGACACAGCAATCAGGTCAGCCAAAATTACC
CTATAGTGCAGAACATCCAGGGGCAAATGGTACATCAGGCCATATCACCTAGAACTTTAAATGCATGGGTAAA
AGTAGTAGAAGAGAAGGCTTTCAGCCCAGAAGTGATACCCATGTTTTCAGCATTATCAGAAGGAGCCACCCCA
CAAGATTTAAACACCATGCTAAACACAGTGGGGGGACATCAAGCAGCCATGCAAATGTTAAAAGAGACCATCA
ATGAGGAAGCTGCAGAATGGGATAGAGTGCATCCAGTGCATGCAGGGCCTATTGCACCAGGCCAGATGAGAGA
ACCAAGGGGAAGTGACATAGCAGGAACTACT??AGCTAGTACCCTTCAGGAACAAATAGGATGGATGACAAAT
AATCCACCTATCCCAGTAGGAGAAATTTATAAAAGATGGATAATCCTGGGATTAAATAAAATAGTAAGAATGT
ATAGCCCTACCAGCATTCTGGACATAAGACAAGGACCAAAAGAACCCTTTAGAGACTATGTAGACCGGTTCTA
TAAAACTCTAAGAGCCGAGCAAGCTTCACAGGAGGTAAAAAATTGGATGACAGAAACCTTGTTGGTCCAAAAT
GCGAACCCAGATTGTAAGACTATTTTAAAAGCATTGGGACCAGCGGCTACACTAGAAGAAATGATGACAGCAT
GTCAGGGAGTAGGAGGACCCGGCCATAAGGCAAGAGTTTTGGCTGAAGCAATGAGCCAAGTAACAAATTCAGC
TACCATAATGATGCAGAGAGGCAATTTTAGGAACCAAAGAAAGATTGTTAAGTGTTTCAATTGTGGCAAAGAA
GGGCACACAGCCAGAAATTGCAGGGCCCCTAGGAAAAAGGGCTGTTGGAAATGTGGAAAGGAAGGACACCAAA
TGAAAGATTGTACTGAGAGACAGGCTAATTTTTTAGGGAAGATCTGGCCTTCCCACAAGGGAAGGCCAGGGAA
TTTTCTTCAGAGCAGACCAGAGCCAACAGCCCCACCAGAAGAGAGCTTCAGGTTTGGGGAAGAGACAACAACT
CCCTCTCAGAAGCAGGAGCCGATAGACAAGGAACTGTATCCTTTAGCTTCCCTCAGATCACTCTTTGGCAGCG
ACCCCTCGTCACAATAAAGATAGGGGGGCAATTAAAGGAAGCTCTATTAGATACAGGAGCAGATGATACAGTA
TTAGAAGAAATGAATTTGCCAGGAAGATGGAAACCAAAAATGATAGGGGGAATTGGAGGTTTTATCAAAGTAA
GACAGTATGATCAGATACTCATAGAAATCTGCGGACATAAAGCTATAGGTACAGTATTAGTAGGACCTACACC
TGTCAACATAATTGGAAGAAATCTGTTGACTCAGATTGGCTGCACTTTAAATTTTCCCATTAGTCCTATTGAG
ACTGTACCAGTAAAATTAAAGCCAGGAATGGATGGCCCAAAAGTTAAACAATGGCCATTGACAGAAGAAAAAA
TAAAAGCATTAGTAGAAATTTGTACAGAAATGGAAAAGGAAGGAAAAATTTCAAAAATTGGGCCTGAAAATCC
ATACAATACTCCAGTATTTGCCATAAAGAAAAAAGACAGTACTAAATGGAGAAAATTAGTAGATTTCAGAGAA
CTTAATAAGAGAACTCAAGATTTCTGGGAAGTTCAATTAGGAATACCACATCCTGCAGGGTTAAAACAGAAAA
AATCAGTAACAGTACTGGATGTGGGCGATGCATATTTTTCAGTTCCCTTAGATAAAGACTTCAGGAAGTATAC
TGCATTTACCATACCTAGTATAAACAATGAGACACCAGGGATTAGATATCAGTACAATGTGCTTCCACAGGGA
TGGAAAGGATCACCAGCAATATTCCAGTGTAGCATGACAAAAATCTTAGAGCCTTTTAGAAAACAAAATCCAG
ACATAGTCATCTATCAATACATGGATGATTTGTATGTAGGATCTGACTTAGAAATAGGGCAGCATAGAACAAA
AATAGAGGAACTGAGACAACATCTGTTGAGGTGGGGATTTACCACACCAGACAAAAAACATCAGAAAGAACCT
CCATTCCTTTGGATGGGTTATGAACTCCATCCTGATAAATGGACAGTACAGCCTATAGTGCTGCCAGAAAAGG
ACAGCTGGACTGTCAATGACATACAGAAATTAGTGGGAAAATTGAATTGGGCAAGTCAGATTTATGCAGGGAT
TAAAGTAAGGCAATTATGTAAACTTCTTAGGGGAACCAAAGCACTAACAGAAGTAGTACCACTAACAGAAGAA
GCAGAGCTAGAACTGGCAGAAAACAGGGAGATTCTAAAAGAACCGGTACATGGAGTGTATTATGACCCATCAA
AAGACTTAATAGCAGAAATACAGAAGCAGGGGCAAGGCCAATGGACATATCAAATTTATCAAGAGCCATTTAA
AAATCTGAAAACAGGAAAGTATGCAAGAATGAAGGGTGCCCACACTAATGATGTGAAACAATTAACAGAGGCA
GTACAAAAAATAGCCACAGAAAGCATAGTAATATGGGGAAAGACTCCTAAATTTAAATTACCCATACAAAAGG
AAACATGGGAAGCATGGTGGACAGAGTATTGGCAAGCCACCTGGATTCCTGAGTGGGAGTTTGTCAATACCCC
TCCCTTAGTGAAGTTATGGTACCAGTTAGAGAAAGAACCCATAATAGGAGCAGAAACTTTCTATGTAGATGGG
GCAGCCAATAGGGAAACTAAATTAGGAAAAGCAGGATATGTAACTGACAGAGGAAGACAAAAAGTTGTCCCCC
TAACGGACACAACAAATCAGAAGACTGAGTTACAAGCAATTCATCTAGCTTTGCAGGATTCGGGATTAGAAGT
AAACATAGTGACAGACTCACAATATGCATTGGGAATCATTCAAGCACAACCAGATAAGAGTGAATCAGAGTTA
GTCAGTCAAATAATAGAGCAGTTAATAAAAAAGGAAAAAGTCTACCTGGCATGGGTACCAGCACACAAAGGAA
TTGGAGGAAATGAACAAGTAGATAAATTGGTCAGTGCTGGAATCAGGAAAGTACTATTTTTAGATGGAATAGA
TAAGGCCCAAGAAGAACATGAGAAATATCACAGTAATTGGAGAGCAATGGCTAGTGATTTTAACCTACCACCT
GTAGTAGCAAAAGAAATAGTAGCCAGCTGTGATAAATGTCAGCTAAAAGGGGAAGCCATGCATGGACAAGTAG

ACTGTAGCCCAGGAATATGGCAGCTAGATTGTACACATTTAGAAGGAAAAGTTATCTTGGTAGCAGTTCATGT
AGCCAGTGGATATATAGAAGCAGAAGTAATTCCAGCAGAGACAGGGCAAGAAACAGCATACTTCCTCTTAAAA
TTAGCAGGAAGATGGCCAGTAAAAACAGTACATACAGACAATGGCAGCAATTTCACCAGTACTACAGTTAAGG
CCGCCTGTTGGTGGGCGGGGATCAAGCAGGAATTTGGCATTCCCTACAATCCCCAAAGTCAAGGAGTAATAGA
ATCTATGAATAAAGAATTAAAGAAAATTATAGGACAGGTAAGAGATCAGGCTGAACATCTTAAGACAGCAGTA
CAAATGGCAGTATTCATCCACAATTTTAAAAGAAAAGGGGGGATTGGGGGGTACAGTGCAGGGGAAAGAATAG
TAGACATAATAGCAACAGACATACAAACTAAAGAATTACAAAAACAAATTACAAAAATTCAAAATTTTCGGGT
TTATTACAGGGACAGCAGAGATCCAGTTTGGAAAGGACCAGCAAAGCTCCTCTGGAAAGGTGAAGGGGCAGTA
GTAATACAAGATAATAGTGACATAAAAGTAGTGCCAAGAAGAAAAGCAAAGATCATCAGGGATTATGGAAAAC
AGATGGCAGGTGATGATTGTGTGGCAAGTAGACAGGATGAGGATTAACACATGGAAAAGATTAGTAAAACACC
ATTA?TATATTTCAAGGAAAGCTAAGGACTGGTTTTATAGACATCACTATGAAAGTACTAATCCAAAAATAAG
TTCAGAAGTACACATCCCACTAGGGGATGCTAAATTAGTAATAACAACATATTGGGGTCTGCATACAGGAGAA
AGAGACTGGCATTTGGGTCAGGGAGTCTCCATAGAATGGAGGAAAAAGAGATATAGCACACAAGTAGACCCTG
ACCTAGCAGACCAACTAATTCATCTGCACTATTTTGATTGTTTTTCAGAATCTGCTATAAGAAATACCATATT
AGGACGTATAGTTAGTCCTAGGTGTGAATATCAAGCAGGACATAACAAGGTAGGATCTCTACAGTACTTGGCA
CTAGCAGCATTAATAAAACCAAAACAGATAAAGCCACCTTTGCCTAGTGTTAGGAAACTGACAGAGGACAGAT
GGAACAAGCCCCAGAAGACCAAGGGCCACAGAGGGAGCCATACAATGAATGGACACTAGAGCTTTTAGAGGAA
CTTAAGAGTGAAGCTGTTAGACATTTTCCTAGGATATGGCTCCATAACTTAGGACAACATATCTATGAAACTT
ACGGGGATACTTGGGCAGGAGTGGAAGCTTTTAGAGGAATTCTGCAACAACTGCTGTTTATCCATTTCAGAAT
TGGGTGTCGACATAGCAGAATAGGCGTTACTCGACAGAGGAGAGCAAGAATGGAGCCAGTAGATCCTAGACTA
GAGCCCTGGAAGCATCCAGGAAGTCAGCCTAAAACTGCTTGTACCAATTGCTATTGTAAAAAGTGTTGCTTTC
ATTGCCAAGTTTGTTTCATGACAAAAGCCTTAGGCATCTCCTATGGCAGGAAGAAGCGGAGACAGCGACGAAG
AGCTCATCAGAACAGTCAGACTCATCAAGCTTCTCTATCAAAGCAGTAAGTAGTACATGTAATGC??TTTCTT
TCAATAGTTGTGTGGTCCATAGTAATCATAGAATATAGGAAAATATTAAGACAAAGAAAAATAGACAGGTTAA
TTGATAGACTAATAGAAAGAGCAGAAGACAGTGGCAATGAGAGTGAAGGAGAAGTGTCAGCACTTGTGGAGAT
GGGGGTGGAGATGGGGCACCATGCTCCTTGGGATATTGATGATCTGTAGTGCTACAGAAAAATTGTGGGTCAC
AGTCTATTATGGGGTACCTGTGTGGAAGGAAGCAACCACCACTCTATTTTGTGCATCAGATGCTAAAGCATAT
GATACAGAGGTACATAATGTTTGGGCCACACATGCCTGTGTACCCACAGACCCCAACCCACAAGAAGTAGTAT
TGGTAAATGTGACAGAAAATTTTAACATGTGGAAAAATGACATGGTAGAACAGATGCATGAGGATATAATCAG
TTTATGGGATCAAAGCCTAAAGCCATGTGTAAAATTAACCCCACTCTGTGTTAGTTTAAAGTGCACTGATTTG
AAGAATGATACTAATACCAATAGTAGTAGCGGGAGAATGATAATGGAGAAAGGAGAGATAAAAAACTGCTCTT
TCAATATCAGCACAAGCATAAGAGATAAGGTGCAGAAAGAATATGCATTCTTTTATAAACTTGATATAGTACC
AATAGATAATACCAGCTATAGGTTGATAAGTTGTAACACCTCAGTCATTACACAGGCCTGTCCAAAGGTATCC
TTTGAGCCAATTCCCATACATTATTGTGCCCCGGCTGGTTTTGCGATTCTAAAATGTAATAATAAGACGTTCA
ATGGAACAGGACCATGTACAAATGTCAGCACAGTACAATGTACACATGGAATCAGGCCAGTAGTATCAACTCA
ACTGCTGTTAAATGGCAGTCTAGCAGAAGAAGATGTAGTAATTAGATCTTCAGACCTCACAAACAATGCTAAA
ACCATAATAGTACAGCTGAATACATCTGTAGAAATTAATTGTACAAGACCCAACAACAATACAAGAAAAAGTA
TAACTATAGGACCAGGGAGAGCATTTTATACAACAGGCATAATAGGAGATATAAGACAAGCACATTGTAACCT
TAGTAGCGCAAAATGGAATGACACTTTAAAACAGATAGTTATAAAGTTAAAAGAACAATTTGGGAACAAGACA
ATAGTCTTTAATCAATCTTCAGGAGGGGACCCAGAAATTGTAATGCACAGCTTTAATTGTGGAGGGGAATTTT
TCTACTGTAATACAACACAGCTGTTTAACAGTACTTGGAATGGTACTGCCTGGAATGATACTACAGGGTCAGA
TAACAATGGAAATATCACACTCCCATGCAGAATAAAACAAATTGTAAACAGGTGGCAGGAAGTAGGAAAAGCA
ATGTATGCCCCTCCCATCGAAGGACAAATTAGATGTTCATCAAATATTACAGGGCTACTATTAACAAGAGATG
GTGGAAATAGTAATAACACGACGGGGATCTTCAGACCTGGAGGAGGAGATATGAGGGACAATTGGAGAAGTGA
ATTATATAAATATAAAGTAGTAAAAATTGAACCATTAGGAGTAGCACCCACCAAGGCAAAGAGAAGAGTGGTG
CAGAGAGAAAAAAGAGCAGTGGGAATAGGAGCTTTGTTCCTTGGGTTCTTGGGAGCAGCAGGAAGCACTATGG
GCGCAGCGTCAATGACGCTGACGGTACAGGCCAGACAATTATTGTCTGGTATAGTGCAGCAGCAGAACAATTT
GCTGAGGGCTATTGAGGCGCAACAGCATCTGTTGCAACTCACAGTCTGGGGCATCAAGCAGCTCCAGGCAAGA
ATCCTGGCTGTGGAAAGATACCTAAAGGATCAACAGCTCCTGGGGATTTGGGGTTGCTCTGGAAAACTCATTT
GCACCACTGCTGTGCCTTGGAATGCTAGTTGGAGTAATAAATCTCTGGAACAGATTTGGAATCACACGACCTG
GATGGAGTGGGACAGAGAAATTAACAATTACACAAGCTTAATACACTCCTTAATTGAAGAATCGCAAAACCAG
CAAGAAAAGAATGAACAAGAATTATTGGAATTAGATAAATGGGCAAGTTTGTGGAATTGGTTTAACATAACAA
ATTGGCTGTGGTATATAAAATTATTCATAATGATAGTAGGAGGCTTGGTAGGTTTAAGAATAGTTTTTGCTGT
ACTTTCTATAGTGAATAGAGTTAGGCAGGGATATTCACCATTATCGTTTCAGACCCACCTCCCAACCCCGAGG

GGACCCGACAGGCCCGAAGGAATA?AAGAAGAAGGTGGAGAGAGAGACAGAGACAGATCCATTCGATTAGTGA
ACGGATCTCGACGGTATCGCCGAATTCACAAATGGCGCCTGTGCCTCTTCAGCTACCACCGCTTGAGAGACTT
ACTCTTGATTGTAGCGAGGATTGTGGAACTTCTGGGACGCAGGGGGTGGGAGGCCCTGAAATATTGGTGGAAT
CTCCTGCAGTATTGGAGTCAGGAACTAAAGAATAGTGCTGTTAGTCTGCTTAATGCCACAGCTATAGCAGTAG
CTGAGGGGACAGATAGGATTATAGAAATATTACAAAGAGCTTGTAGAGCTATTCTCCACATACCTAGAAGAAT
AAGACAGGGCTTAGAAAGGGCTTTGCTATAAGATGGGTGGTAAGTGGTCAAAACGTAGTGGGGGATGGGAAGC
TGTAAGGGAAAAAATAAGACAAACT?AGGGAGCAGGAAATATCTGTGGGCTTGTGACTCGAGACCTAGAAAAA
CATGGAGCAATCACAAGTAGCAATACAGCAGCTACCAATGCTGATTGTGCCTGGCTAGAAGCACAAGAGGAGG
AGGAGGTGGGTTTTCCAGTCACACCTCAGGTACCTTTAAGACCAATGACTTACAAGGCAGCTGTAGATCTTAG
CCACTTTTTAAAAGAAAAGGGGGGACTGGAAGGGCTAATTCACTCCCAACGAAGACAAAATCGTCGAGAGATG
CTGCATTACAACACACAAGGCTATTTCCCTGATTGGCAGAACTACACACCAGGGCCAGGGGAGAGATTTCCAC
TGACCTTTGGATGGTGCTTCAAGCTAGTACCAGTTGATCCAGATCAGGTAGAAGAGGCTAATAAAGGAGAGAA
CAACAGCTTGTTACACCCTATGAGCCAGCATGGGATAGAGGACCCGGAG

>M-2D2 HIV-1 genome, derived from RNA genomic sequence
AATAAAGCTTGCCTTGAGTGCTTCAAGCAGTGTGTGCCCGTCTGTTGTGTGACTCTGGTAACTAGAGATCCCT
CAGACCCTTTTAGTCAGTGTGGAAAATCTCTAGCAGTGGCGCCCGAACAGGGACTTGAAAGCGAAAGGGAAAC
CAGAGGAGCTCTCTCGACGCAGGACTCGGCTTGCTGAAGCGCGCACGGCAAGAGGCGAGGGGCGGCGACTGGT
GAGTACGCCAAAAATTTTGACTAGCGGAGGCTAGAAGGAGAGAGATGGGTGCGAGAGCGTCAGTTT?AAGCGG
GGGAGAATTAGATCGATGGGAAAAAATTCGGTTAAGGCCAGGGGGAAAGAAAAAATATAAATTAAAACATATA
GTATGGGCAAGCAGGGAGCTAGAACGATTCGCAGTTAATCCTGGCCTGTTAGAAACATCAGAAGGCTGTAGAC
AAATACTGGGACAGCTACAACCATCCCTTCAGACAGGATCAGAAGAACTTAGATCATTATATAATACAGTAGC
AACCCTCTATTGTGTGCATCAAAGGATAGAGATAAAAGACACCAAGGAAGCTTTAGACAAGATAGAGGAAGAG
CAAAACAAAAGTAAGAAAAAAGCACAGCAAGCAGCAGCTGACACAGGACACAGCAATCAGGTCAGCCAAAATT
ACCCTATAGTGCAGAACATCCAGGGGCAAATGGTACATCAGGCCATATCACCTAGAACTTTAAATGCATGGGT
AAAAGTAGTAGAAGAGAAGGCTTTCAGCCCAGAAGTGATACCCATGTTTTCAGCATTATCAGAAGGAGCCACC
CCACAAGATTTAAACACCATGCTAAACACAGTGGGGGGACATCAAGCAGCCATGCAAATGTTAAAAGAGACCA
TCAATGAGGAAGCTGCAGAATGGGATAGAGTGCATCCAGTGCATGCAGGGCCTATTGCACCAGGCCAGATGAG
AGAACCAAGGGGAAGTGACATAGCAGGAACTACT??AGCTAGTACCCTTCAGGAACAAATAGGATGGATGACA
AATAATCCACCTATCCCAGTAGGAGAAATTTATAAAAGATGGATAATCCTGGGATTAAATAAAATAGTAAGAA
TGTATAGCCCTACCAGCATTCTGGACATAAGACAAGGACCAAAAGAACCCTTTAGAGACTATGTAGACCGGTT
CTATAAAACTCTAAGAGCCGAGCAAGCTTCACAGGAGGTAAAAAATTGGATGACAGAAACCTTGTTGGTCCAA
AATGCGAACCCAGATTGTAAGACTATTTTAAAAGCATTGGGACCAGCGGCTACACTAGAAGAAATGATGACAG
CATGTCAGGGAGTAGGAGGACCCGGCCATAAGGCAAGAGTTTTGGCTGAAGCAATGAGCCAAGTAACAAATTC
AGCTACCATAATGATGCAGAGAGGCAATTTTAGGAACCAAAGAAAGATTGTTAAGTGTTTCAATTGTGGCAAA
GAAGGGCACACAGCCAGAAATTGCAGGGCCCCTAGGAAAAAGGGCTGTTGGAAATGTGGAAAGGAAGGACACC
AAATGAAAGATTGTACTGAGAGACAGGCTAATTTTTTAGGGAAGATCTGGCCTTCCCACAAGGGAAGGCCAGG
GAATTTTCTTCAGAGCAGACCAGAGCCAACAGCCCCACCAGAAGAGAGCTTCAGGTTTGGGGAAGAGACAACA
ACTCCCTCTCAGAAGCAGGAGCCGATAGACAAGGAACTGTATCCTTTAGCTTCCCTCAGATCACTCTTTGGCA
GCGACCCCTCGTCACAATAAAGATAGGGGGGCAATTAAAGGAAGCTCTATTAGATACAGGAGCAGATGATACA
GTATTAGAAGAAATGAATTTGCCAGGAAGATGGAAACCAAAAATGATAGGGGGAATTGGAGGTTTTATCAAAG
TAAGACAGTATGATCAGATACTCATAGAAATCTGCGGACATAAAGCTATAGGTACAGTATTAGTAGGACCTAC
ACCTGTCAACATAATTGGAAGAAATCTGTTGACTCAGATTGGCTGCACTTTAAATTTTCCCATTAGTCCTATT
GAGACTGTACCAGTAAAATTAAAGCCAGGAATGGATGGCCCAAAAGTTAAACAATGGCCATTGACAGAAGAAA
AAATAAAAGCATTAGTAGAAATTTGTACAGAAATGGAAAAGGAAGGAAAAATTTCAAAAATTGGGCCTGAAAA
TCCATACAATACTCCAGTATTTGCCATAAAGAAAAAAGACAGTACTAAATGGAGAAAATTAGTAGATTTCAGA
GAACTTAATAAGAGAACTCAAGATTTCTGGGAAGTTCAATTAGGAATACCACATCCTGCAGGGTTAAAACAGA
AAAAATCAGTAACAGTACTGGATGTGGGCGATGCATATTTTTCAGTTCCCTTAGATAAAGACTTCAGGAAGTA
TACTGCATTTACCATACCTAGTATAAACAATGAGACACCAGGGATTAGATATCAGTACAATGTGCTTCCACAG
GGATGGAAAGGATCACCAGCAATATTCCAGTGTAGCATGACAAAAATCTTAGAGCCTTTTAGAAAACAAAATC
CAGACATAGTCATCTATCAATACATGGATGATTTGTATGTAGGATCTGACTTAGAAATAGGGCAGCATAGAAC
AAAAATAGAGGAACTGAGACAACATCTGTTGAGGTGGGGATTTACCACACCAGACAAAAAACATCAGAAAGAA
CCTCCATTCCTTTGGATGGGTTATGAACTCCATCCTGATAAATGGACAGTACAGCCTATAGTGCTGCCAGAAA
AGGACAGCTGGACTGTCAATGACATACAGAAATTAGTGGGAAAATTGAATTGGGCAAGTCAGATTTACCCAGG
AATTAAAGTAAGGCAATTATGTAAACTCCTCAGGGGAACCAAAGCACTAACAGAAGTAGTACCACTAACAGAC
GAAGCAGAGCTAGAACTAGCAGAAAACAGGGAGATACTAAAACAGCCAATGCATGGAGTGTATTATGACCCAT
CAAAAGAATTAGTAGCAGAAATACAGAAACAGGAACATGGCCAGTGGACATATCAAATTTATCAAGAGCCATT
TCAAAACCTGAAAACAGGAAAATATGCAAGAATGAGGGGTACTCACACTAATGATGTAAAACAATTAACAGAG
GCAGTGCAGAAAATAGCCACAGAAAGCATAGTAATATGGGGAAAGACTCCTAAGTTTAGACTACCCATACAAA
AAGAAACATGGGAAACATGGTGGACAGAGTATTGGCAAGCCACCTGGATTCCTGAGTGGGAGTTTGTTAATAC
CCCTCCCTTAGTAAAATTATGGTACCAGTTAGAGAAGGACCCCATAGTAGGAGCAGAAACTTTTTATGTAGAT
GGGGCAGCCAATAGAGAAACTAAATTAGGAAGAGCAGGATATGTTACTGACAGAGGAAGACAAAAGGTTGTCT
CCCTAACTGACACAACAAATCAAAAGACTGAGTTACAAGCAATTAATCTAGCTCTGCAGGATTCAGGATCAGA
GGTAAACATAGTAACAGACTCACAATATGCACTAGGAATTATTCAAGCACAACCAGATAAAAGTGAATCAGAG
TTAGTTAGTCAAATAATAGAGCAGTTAATAAAAAAGGAAAAGATCTATCTGGCATGGGTGCCAGCACACAAAG
GAATTGGGGGAAATGAACAAGTAGATAAATTAGTCAGTTCTGGAATCAGGAAAGTACTATTTTTAGATGGAAT
AGAGAAGGCCCAGGAAGACCATGAGAAATATCACAGTAATTGGAGAGCAATGGCTAGTGATTTTAACCTACCA
CCTATAGTAGCAAAAGAAATAATAGCCTGCTGTGATAAATGTCAGCTAAAAGGAGAAGCCATGCATGGACAAG

TAGACTGTAGCCCAGGAATATGGCAACTAGATTGTACACATTTAGAAGGAAAAATTATCCTGGTAGCAGTTCA
TGTAGCCAGTGGATATATAGAAGCAGAAGTAATTCCAGCAGAGACAGGGCAAGAAACAGCATACTTCCTCTTA
AAATTAGCAGGAAGATGGCCAGTAAAAACAGTACATACAGACAATGGCAGCAATTTCACCAGTACTACAGTTA
AGGCCGCCTGTTGGTGGGCGGGGGTCAAGCAGGAATTTGGCATTCCCTACAATCCCCAAAGTCAAGGGGTAGT
AGAATCTATGAATAAAGAACTAAAGAAAATTATAGGACAGGTAAGAGATCAGGCTGAACATCTTAAGACAGCA
GTACAAATGGCAGTATTCATCCACAATTTTAAAAGAAAAGGGGGGATTGGGGGGTACAGTGCAGGGGAAAGAA
TAGTAGACATAATAGCAACAGACATACAAACTAAAGAATTACAAAAACAAATTACAAAAATTCAAAATTTTCG
GGTTTATTACAGGGACAGCAGAGATCCACTTTGGAAAGGACCAGCAAAACTTCTCTGGAAAGGTGAAGGGGCA
GTAGTAATACAAGATAATAGTGACATAAAAGTAGTGCCAAGAAGAAAAGCAAAGATCATCAGGGATTATGGAA
AACAGATGGCAGGTGATGATTGTGTGGCAAGTAGACAGGATGAGGATTAACACATGGAAAAGATTAGTAAAAC
ACCATTA?TATATTTCAAGGAAAGCTAAGGACTGGTTTTATAGACATCACTATGAAAGTACTAATCCAAAAAT
AAGTTCAGAAGTACACATCCCACTAGGGGATGCTAAATTAGTAATAACAACATATTGGGGTCTGCATACAGGA
GAAAGAGACTGGCATTTGGGTCAGGGAGTCTCCATAGAATGGAGGAAAAAGAGATATAGCACACAAGTAGACC
CTGACCTAGCAGACCAACTAATTCATCTGCACTATTTTGATTGTTTTTCAGAATCTGCTATAAGAAATACCAT
ATTAGGACGTATAGTTAGTCCTAGGTGTGAATATCAAGCAGGACATAACAAGGTAGGATCTCTACAGTACTTG
GCACTAGCAGCATTAATAAAACCAAAACAGATAAAGCCACCTTTGCCTAGTGTTAGGAAACTGACAGAGGACA
GATGGAACAAGCCCCAGAAGACCAAGGGCCACAGAGGGAGCCATACAATGAATGGACACTAGAGCTTTTAGAG
GAATTCTGCAACAACTGCTGTTTATCCATTTCAGAATTGATGGCTCCATGGCTTAGGACAATATATCTACCAC
AGAGGGAGCCATACAATGAATGGACACTAGAGCTTTTAGAG?GAATTCTGCAACAACTGCTGTTTATCCATTT
CAGAATTGGGTGTCGACATAGCAGAATAGGCGTTACTCGACAGAGGAGAGCAAGAAATGGAGCCAGTAGATCC
TAGACTAGAGCCCTGGAAGCATCCAGGAAGTCAGCCTAAAACTGCTTGTACCAATTGCTATTGTAAAAAGTGT
TGCTTTCATTGCCAAGTTTGTTTCATGACAAAAGCCTTAGGCATCTCCTATGGCAGGAAGAAGCGGAGACAGC
GACGAAGAGCTCATCAGAACAGTCAGACTCATCAAGCTTCTCTATCAAAGCAGTAAGTAGTACATGTAATGC?
?TTTCTTTCAATAGTTGTGTGGTCCATAGTAATCATAGAATATAGGAAAATATTAAGACAAAGAAAAATAGAC
AGGTTAATTGATAGACTAATAGAAAGAGCAGAAGACAGTGGCAATGAGAGTGAAGGAGAAGTGTCAGCACTTG
TGGAGATGGGGGTGGAGATGGGGCACCATGCTCCTTGGGATATTGATGATCTGTAGTGCTACAGAAAAATTGT
GGGTCACAGTCTATTATGGGGATCTTCAGACCTGGGAAGCAACCACCACTTTATTTTGTGCATCAGATGCTAA
AGCATATGATACAGAGATGCATAATGTTTGGGCCACACATGCCTGTGTACCCACAGACCCTAGCCCACAAGAA
GTAGTATTGGAAAATGTGACAGAAAATTTTAACATGTGGAAAAATAACATGGTAGAACAGATGCATGAAGATA
TAATCAGTTTGTGGGATCAAAGCCTAAAGCCATGTGTAAAATTAACCCCACTCTGTGTCACTTTAAATTGCAC
TGATGTGAGAAATTGTAACACCTCAGTCATTACACAGGCCTGTCCAAAGGTATCCTTTGAACCAATTCCCATA
CATTATTGTACCCCGGCTGGTTTTGCGATTCTAAAGTGTAATGATAATAAGTTCAATGGAACAGGACCATGTA
CTAATGTTAGCACAGTACAATGTACACATGGAATTAAGCCAGTAGTGTCAACTCAATTGCTGTTGAATGGCAG
TCTGGCAGAAGGAGGAGAGGTAGTAATTAGATCTGAAAATTTCACAAACAATGCTAAAACCATAATAGTACAG
CTGAATACATCTGTAGAAATTAATTGTATAAGACCCAACAACAATACAAGAAAAAGTATAACTATAGGACCAG
GGAGAGCATTTTATACAACAGACATAATAGGAGATATAAGACAAGCACATTGTAACCTTAGTAGAGCAAAATG
GAATGACACTTTAAAACAGATAGTTACAAAATTAAAAGAACAATTTGAGAACAAGACAATAGTCTTTAATCAA
TCTTCAGGAGGGGACCCAGAAATTGTAATGCACAGCTTTAATTGTGGAGGGGAATTTTTCTACTGTAATACAA
CACAGCTGTTTAACAGTACTTGGAATGGTACTGCCTGGAATGATACTACAGGGTCAGAGAACATCACACTCCC
ATGCAGAATAAAACAAATTGTAAACAGGTGGCAGGAAGTAGGAAAAGCAATGTATGCCCCTCCCATCAAAGGA
CAAATTAGATGTTCATCAAATATTACAGGGCTACTATTAACAAGAGATGGTGGAAATAGTAAGTCTATTATGG
GGATCTTCAGACCTGGAGGAGGAGATATGAGGGACAATTGGAGAAGTGAATTATATAAATATAAAGTAGTAAA
AATTGAACCATTAGGAGTAGCACCCACCAAGGCAAAGAGAAGAGTGGTGCAGAGAGAAAAAAGAGCAGTGGGA
ATAGGAGCTTTGTTCCTTGGGTTCTTGGGAGCAGCAGGAAGCACTATGGGCGCAGCGTCAATGACGCTGACGG
TACAGGCCAGACAATTATTGTCTGGTATAGTGCAGCAGCAGAACAATTTGCTGAGCTTTATTGAGGCGCAACA
GCATCTGTTGCAACTCACAGTCTGGGGCATCAAGCAGCTCCAGGCAAGAATCCTGGCTGTGGAAAGATACCTA
AAGGATCAACAGCTCCTGGGGATTTGGGGTTGCTCTGGAAAACTCATTTGCACCACTGCTGTGCCTTGGAATG
CTAGTTGGAGTAATAAATCTCTGGAACAGATTTGGAATCACACGACCTGGATGGAGTGGGACAGAGAAATTAA
CAATTACACAAGCTTAATACACTCCTTAATTGAAGAATCGCAAAACCAGCAAGAAAAGAATGAACAAGAATTA
TTGGAATTAGATAAATGGGCAAGTTTGTGGAATTGGTTTAACATAACAAATTGGCTGTGGTATATAAAATTAT
TCATAATGATAGTAGGAGGCTTGGTAGGTTTAAGAATAGTTTTTGCTGTACTTTCTATAGTGAATAGAGTTAG
GCAGGGATATTCACCATTATCGTTTCAGACCCACCTCCCAACCCCGAGGGGACCCGACAGGCCCGAAGGAATA
?AAGAAGAAGGTGGAGAGAGAGACAGAGACAGATCCATTCGATTAGTGAACGGATCTCGACGGTATCGCCGAA
TTCACAAATGGCAGCTGTGCCTCTTCAGCTACCACCGCTTGAGAGACTTACTCTTGATTGTAGCGAGGATTGT

GGAACTTCTGGGACGCAGGGGGTGGGAGGCCCTGAAATATTGGTGGAATCTCCTGCAGTATTGGAGTCAGGAA
CTAAAGAATAGTGCTGTTAGTCTGCTTAATGCCACAGCTATAGCAGTAGCTGAGGGGACAGATAGGATTATAG
AAGTATTACAAAGAGCTTGTAGAGCTATTCTCCACATACCTAGAAGAATAAGACAGGGCTTAGAAAGGGCTTT
GCTATAAGATGGGTGGTAAGTGGTCAAAACGTCGTGCGAGTGGATGGGAAGCTGTAAGGGAAAAAATAAGACA
AACGATCCACTGGGGAGCAGGAAATATCTGTGGGCTTGTGACTCGAGACCTAGAAAAACATGGAGCAATCACA
AGTAGCAATACAGCAGCTACCAATGCTGATTGTGCCTGGCTAGAAGCACAAGAGGAGGAGGAGGTGGGTTTTC
CAGTCACACCTCAGGTACCTTTAAGACCAATGACTTACAAGGCAGCTGTAGATCTTAGCCACTTTTTAAAAGA
AAAGGGGGGACTGGAAGGGCTAATTCACTCCCAACGAAGACAAAATCGTCGAGAGATGCTGCATATAAGCAGC
CAAGGCTATTTCCCTGATTGGCAGAACTACACACCAGGGCCAGGGGAGAGATTCCCACTGACCTTTGGATGGT
GCTTCAAGCTAGTACCAGTTGATCCAGATCAGGTAGAAGAGGCTAATAAAGGAGAGAACAACAGCTTGTTACA
CCCTATGAGCCAGCATGGGATGGAGGACCCGGAG

>M-2D3 HIV-1 genome, derived from RNA genomic sequence
AATAAAGCTTGCCTTGAGTGGCTTAAGCAGTGTGTGCCCGTCTGTTGTGTGACTCTGGTAACTAGAGATCCCT
CAGACCCTTTTAGTCAGTGTGGAAAATCTCTAGCAGTGGCGCCCGAACAGGGACTTGAAAGCGAAAGGGAAAC
CAGAGGAGCTCTCTCGACGCAGGACTCGGCTTGCTGAAGCGCGCACGGCAAGAGGCGAGGGGCGGCGACTGGT
GAGTACGCCAAAAATTTTGACTAGCGGAGGCTAGAAGGAGAGAGATGGGTGCGAGAGCGTCAGTTT?AAGCGG
GGGAGAATTAGATCGATGGGAAAAAATTCGGTTAAGGCCAGGGGGAAAGAAAAAATATAAATTAAAACATATA
GTATGGGCAAGCAGGGAGCTAGAACGATTCGCAGTTAATCCTGGCCTGTTAGAAACATCAGAAGGCTGTAGAC
AAATACTGGGACAGCTACAACCATCCCTTCAGACAGGATCAGAAGAACTTAGATCATTATATAATACAGTAGC
AACCCTCTATTGTGTGCATCAAAGGATAGAGATAAAAGACACCAAGGAAGCTTTAGACAAGATAGAGGAAGAG
CAAAACAAAAGTAAGAAAAAAGCACAGCAAGCAGCAGCTGACACAGGACACAGCAATCAGGTCAGCCAAAATT
ACCCTATAGTGCAGAACATCCAGGGGCAAATGGTACATCAGGCCATATCACCTAGAACTTTAAATGCATGGGT
AAAAGTAGTAGAAGAGAAGGCTTTCAGCCCAGAAGTGATACCCATGTTTTCAGCATTATCAGAAGGAGCCACC
CCACAAGATTTAAACACCATGCTAAACACAGTGGGGGGACATCAAGCAGCCATGCAAATGTTAAAAGAGACCA
TCAATGAGGAAGCTGCAGAATGGGATAGAGTGCATCCAGTGCATGCAGGGCCTATTGCACCAGGCCAGATGAG
AGAACCAAGGGGAAGTGACATAGCAGGAACTACT??AGCTAGTACCCTTCAGGAACAAATAGGATGGATGACA
AATAATCCACCTATCCCAGTAGGAGAAATTTATAAAAGATGGATAATCCTGGGATTAAATAAAATAGTAAGAA
TGTATAGCCCTACCAGCATTCTGGACATAAGACAAGGACCAAAAGAACCCTTTAGAGACTATGTAGACCGGTT
CTATAAAACTCTAAGAGCCGAGCAAGCTTCACAGGAGGTAAAAAATTGGATGACAGAAACCTTGTTGGTCCAA
AATGCGAACCCAGATTGTAAGACTATTTTAAAAGCATTGGGACCAGCGGCTACACTAGAAGAAATGATGACAG
CATGTCAGGGAGTAGGAGGACCCGGCCATAAGGCAAGAGTTTTGGCTGAAGCAATGAGCCAAGTAACAAATTC
AGCTACCATAATGATGCAGAGAGGCAATTTTAGGAACCAAAGAAAGATTGTTAAGTGTTTCAATTGTGGCAAA
GAAGGGCACACAGCCAGAAATTGCAGGGCCCCTAGGAAAAAGGGCTGTTGGAAATGTGGAAAGGAAGGACACC
AAATGAAAGATTGTACTGAGAGACAGGCTAATTTTTTAGGGAAGATCTGGCCTTCCCACAAGGGAAGGCCAGG
GAATTTTCTTCAGAGCAGACCAGAGCCAACAGCCCCACCAGAAGAGAGCTTCAGGTTTGGGGAAGAGACAACA
ACTCCCTCTCAGAAGCAGGAGCCGATAGACAAGGAACTGTATCCTTTAGCTTCCCTCAGATCACTCTTTGGCA
GCGACCCCTCGTCACAATAAAGATAGGGGGGCAATTAAAGGAAGCTCTATTAGATACAGGAGCAGATGATACA
GTATTAGAAGAAATGAATTTGCCAGGAAGATGGAAACCAAAAATGATAGGGGGAATTGGAGGTTTTATCAAAG
TAAGACAGTATGATCAGATACTCATAGAAATCTGCGGACATAAAGCTATAGGTACAGTATTAGTAGGACCTAC
ACCTGTCAACATAATTGGAAGAAATCTGTTGACTCAGATTGGCTGCACTTTAAATTTTCCCATTAGTCCTATT
GAGACTGTACCAGTAAAATTAAAGCCAGGAATGGATGGCCCAAAAGTTAAACAATGGCCATTGACAGAAGAAA
AAATAAAAGCATTAGTAGAAATTTGTACAGAAATGGAAAAGGAAGGAAAAATTTCAAAAATTGGGCCTGAAAA
TCCATACAATACTCCAGTATTTGCCATAAAGAAAAAAGACAGTACTAAATGGAGAAAATTAGTAGATTTCAGA
GAACTTAATAAGAGAACTCAAGATTTCTGGGAAGTTCAATTAGGAATACCACATCCTGCAGGGTTAAAACAGA
AAAAATCAGTAACAGTACTGGATGTGGGCGATGCATATTTTTCAGTTCCCTTAGATAAAGACTTCAGGAAGTA
TACTGCATTTACCATACCTAGTATAAACAATGAGACACCAGGGATTAGATATCAGTACAATGTGCTTCCACAG
GGATGGAAAGGATCACCAGCAATATTCCAGTGTAGCATGACAAAAATCTTAGAGCCTTTTAGAAAACAAAATC
CAGACATAGTCATCTATCAATACATGGATGATTTGTATGTAGGATCTGACTTAGAAATAGGGCAGCATAGAAC
AAAAATAGAGGAACTGAGACAACATCTGTTGAGGTGGGGATTTACCACACCAGACAAAAAACATCAGAAAGAA
CCTCCATTCCTTTGGATGGGTTATGAACTCCATCCTGATAAATGGACAGTACAGCCTATAGTGCTGCCAGAAA
AGGACAGCTGGACTGTCAATGACATACAGAAATTAGTGGGAAAATTGAATTGGGCAAGTCAGATTTATGCAGG
GATTAAAGTAAGGCAATTATGTAAACTTCTTAGGGGAACCAAAGCACTAACAGAAGTAGTACCACTAACAGAA
GAAGCAGAGCTAGAACTGGCAGAAAACAGGGAGATTCTAAAAGAACCGGTACATGGAGTGTATTATGACCCAT
CAAAAGACTTAATAGCAGAAATACAGAAGCAGGGGCAAGGCCAATGGACATATCAAATTTATCAAGAGCCATT
TAAAAATCTGAAAACAGGAAAGTATGCAAGAATGAAGGGTGCCCACACTAATGATGTGAAACAATTAACAGAG
GCAGTACAAAAAATAGCCACAGAAAGCATAGTAATATGGGGAAAGACTCCTAAATTTAAATTACCCATACAAA
AGGAAACATGGGAAGCATGGTGGACAGAGTATTGGCAAGCCACCTGGATTCCTGAGTGGGAGTTTGTCAATAC
CCCTCCCTTAGTGAAGTTATGGTACCAGTTAGAGAAAGAACCCATAATAGGAGCAGAAACTTTCTATGTAGAT
GGGGCAGCCAATAGGGAAACTAAATTAGGAAAAGCAGGATATGTAACTGACAGAGGAAGACAAAAAGTTGTCC
CCCTAACGGACACAACAAATCAGAAGACTGAGTTACAAGCAATTCATCTAGCTTTGCAGGATTCGGGATTAGA
AGTAAACATAGTGACAGACTCACAATATGCATTGGGAATCATTCAAGCACAACCAGATAAGAGTGAATCAGAG
TTAGTCAGTCAAATAATAGAGCAGTTAATAAAAAAGGAAAAAGTCTACCTGGCATGGGTACCAGCACACAAAG
GAATTGGAGGAAATGAACAAGTAGATAAATTGGTCAGTGCTGGAATCAGGAAAGTACTATTTTTAGATGGAAT
AGATAAGGCCCAAGAAGAACATGAGAAATATCACAGTAATTGGAGAGCAATGGCTAGTGATTTTAACCTACCA
CCTGTAGTAGCAAAAGAAATAGTAGCCAGCTGTGATAAATGTCAGCTAAAAGGGGAAGCCATGCATGGACAAG

TAGACTGTAGCCCAGGAATATGGCAGCTAGATTGTACACATTTAGAAGGAAAAGTTATCTTGGTAGCAGTTCA
TGTAGCCAGTGGATATATAGAAGCAGAAGTAATTCCAGCAGAGACAGGGCAAGAAACAGCATACTTCCTCTTA
AAATTAGCAGGAAGATGGCCAGTAAAAACAGTACATACAGACAATGGCAGCAATTTCACCAGTACTACAGTTA
AGGCCGCCTGTTGGTGGGCGGGGATCAAGCAGGAATTTGGCATTCCCTACAATCCCCAAAGTCAAGGAGTAAT
AGAATCTATGAATAAAGAATTAAAGAAAATTATAGGACAGGTAAGAGATCAGGCTGAACATCTTAAGACAGCA
GTACAAATGGCAGTATTCATCCACAATTTTAAAAGAAAAGGGGGGATTGGGGGGTACAGTGCAGGGGAAAGAA
TAGTAGACATAATAGCAACAGACATACAAACTAAAGAATTACAAAAACAAATTACAAAAATTCAAAATTTTCG
GGTTTATTACAGGGACAGCAGAGATCCAGTTTGGAAAGGACCAGCAAAGCTCCTCTGGAAAGGTGAAGGGGCA
GTAGTAATACAAGATAATAGTGACATAAAAGTAGTGCCAAGAAGAAAAGCAAAGATCATCAGGGATTATGGAA
AACAGATGGCAGGTGATGATTGTGTGGCAAGTAGACAGGATGAGGATTAACACATGGAAAAGATTAGTAAAAC
ACCATTA?TATATTTCAAGGAAAGCTAAGGACTGGTTTTATAGACATCACTATGAAAGTACTAATCCAAAAAT
AAGTTCAGAAGTACACATCCCACTAGGGGATGCTAAATTAGTAATAACAACATATTGGGGTCTGCATACAGGA
GAAAGAGACTGGCATTTGGGTCAGGGAGTCTCCATAGAATGGAGGAAAAAGAGATATAGCACACAAGTAGACC
CTGACCTAGCAGACCAACTAATTCATCTGCACTATTTTGATTGTTTTTCAGAATCTGCTATAAGAAATACCAT
ATTAGGACGTATAGTTAGTCCTAGGTGTGAATATCAAGCAGGACATAACAAGGTAGGATCTCTACAGTACTTG
GCACTAGCAGCATTAATAAAACCAAAACAGATAAAGCCACCTTTGCCTAGTGTTAGGAAACTGACAGAGGACA
GATGGAACAAGCCCCAGAAGACCAAGGGCCACAGAGGGAGCCATACAATGAATGGACACTAGAGCTTTTAGAG
GAATTCTGCAACAACTGCTGTTTATCCATTTCAGAATTGGGTGTCGA??????????????????????????
???
?????????????CATAGCAGAATAGGCGTTACTCGACAGAGGAGAGCAAGAAATGGAGCCAGTAGATCCTAG
ACTAGAGCCCTGGAAGCATCCAGGAAGTCAGCCTAAAACTGCTTGTACCAATTGCTATTGTAAAAAGTGTTGC
TTTCATTGCCAAGTTTGTTTCATGACAAAAGCCTTAGGCATCTCCTATGGCAGGAAGAAGCGGAGACAGCGAC
GAAGAGCTCATCAGAACAGTCAGACTCATCAAGCTTCTCTATCAAAGCAGTAAGTAGTACATGTAATGC??TT
TCTTTCAATAGTTGTGTGGTCCATAGTAATCATAGAATATAGGAAAATATTAAGACAAAGAAAAATAGACAGG
TTAATTGATAGACTAATAGAAAGAGCAGAAGACAGTGGCAATGAGAGTGAAGGAGAAGTGTCAGCACTTGTGG
AGATGGGGGTGGAGATGGGGCACCATGCTCCTTGGGATATTGATGATCTGTAGTGCTACAGAAAAATTGTGGG
TCACAGTCTATTATGGGGATCTTCAGACCTGG???
???
???
???
???
???
???
???
???
???
???
???
???
??????????????????????????CAGGAGGGGACCCAGAAATTGTAATGCACAGCTTTAATTGTGGAGGG
GAATTTTTCTACTGTAATACAACACAGCTGTTTAACAGTACTTGGAATGGTACTGACTGGAATGACACTACAG
GGTTAGAGAACATCACACTCCCATGCAGAATAAAACAAATTGTAAACAGGTGGCAGGAAGTAGGAAAAGCAAT
GTATGCCCCTCCCATCAAAGGA??GTCTATTATGGGGATCTTCAGACCTGGAGGAGGAGATATGAGGGACAAT
TGGAGAAGTGAATTATATAAATATAAAGTAGTAAAAATTGAACCATTAGGAGTAGCACCCACCAAGGCAAAGA
GAAGAGTGGTGCAGAGAGAAAAAAGAGCAGTGGGAATAGGAGCTTTGTTCCTTGGGTTCTTGGGAGCAGCAGG
AAGCACTATGGGCGCAGCGTCAATGACGCTGACGGTACAGGCCAGACAATTATTGTCTGGTATAGTGCAGCAG
CAGAACAATTTGCTGAGCTTTATTGAGGCGCAACAGCATCTGTTGCAACTCACAGTCTGGGGCATCAAGCAGC
TCCAGGCAAGAATCCTGGCTGTGGAAAGATACCTAAAGGATCAACAGCTCCTGGGGATTTGGGGTTGCTCTGG
AAAACTCATTTGCACCACTGCTGTGCCTTGGAATGCTAGTTGGAGTAATAAATCTCTGGAACAGATTTGGAAT
CACACGACCTGGATGGAGTGGGACAGAGAAATTAACAATTACACAAGCTTAATACACTCCTTAATTGAAGAAT
CGCAAAACCAGCAAGAAAAGAATGAACAAGAATTATTGGAATTAGATAAATGGGCAAGTTTGTGGAATTGGTT
TAACATAACAAATTGGCTGTGGTATATAAAATTATTCATAATGATAGTAGGAGGCTTGGTAGGTTTAAGAATA
GTTTTTGCTGTACTTTCTATAGTGAATAGAGTTAGGCAGGGATATTCACCATTATCGTTTCAGACCCACCTCC
CAACCCCGAGGGGACCCGACAGGCCCGAAGGAATA?AAGAAGAAGGTGGAGAGAGAGACAGAGACAGATCCAT

TCGATTAGTGAACGGATCTCGACGGTATCGCCGAATTCACAAATGGCAGCTGTGCCTCTTCAGCTACCACCGC
TTGAGAGACTTACTCTTGATTGTAGCGAGGATTGTGGAACTTCTGGGACGCAGGGGGTGGGAGGCCCTGAAAT
ATTGGTGGAATCTCCTGCAGTATTGGAGTCAGGAACTAAAGAATAGTGCTGTTAGTCTGCTTAATGCCACAGC
TATAGCAGTAGCTGAGGGGACAGATAGGATTATAGAAGTATTACAAAGAGCTTGTAGAGCTATTCTCCACATA
CCTAGAAGAATAAGACAGGGCTTAGAAAGGGCTTTGCTATAAGATGGGTGGTAAGTGGTCAAAACGTCGTGCG
GGTGGATGGGAAGCTGTAAGGATCTGTGGGCTTGTGACTCGAGACCTAGAAAAACATGGAGCAATCACAAGTA
GCAATACAGCAGCTACCAATGCTGATTGTGCCTGGCTAGAAGCACAAGAGGAGGAGGAGGTGGGTTTTCCAGT
CACACCTCAGGTACCTTTAAGACCAATGACTTACAAGGCAGCTGTAGATCTTAGCCACTTTTTAAAAGAAAAG
GGGGGACTGGAAGGGCTAATTCACTCCCAACGAAGACAAAATCGTCGAGAGATGCTGCATATAAGCAGCCAAG
GCTATTTCCCTGATTGGCAGAACTACACACCAGGGCCAGGGGAGAGATTTCCCCTGACCTTTGGATGGTGCTT
CAAGCTAGTACCAGTTGATCCAGATCAGGTAGAAGAGGCTAATAAAGGAGAGACCAACAGCTTGTTACACCCT
ATGAGCCAGCATGGGATAGAGGACCCGGAG

>S-1A2 HIV-1 genome, derived from RNA genomic sequence
GCAGTGGCGCCCGAACAGGGACCTGAAAGCGAAAGGAAGACCGGAGGAGCTCTCTCGACGCAGGACTCGGCTT
GCTGAAGCGCGCACGGCAAGAGGCGAGGGGCGGCGACTGGTGAGTACGCCAAAAATTTTGACTAGCGGAGGCT
AGAAGGAGAGAGATGGGTGCGAGAGCGTCAATATTAAGCGGGGGAGAATTAGATAGATGGGAAAAAATTCGGT
TAAGGCCAGGGGGAAAGAAAAAATATAGACTAAAACATTTAGTATGGGCAAGCAGGGAGCTAGAACGATTCGC
AATCAACCCTGGCCTGTTAGAAACATCAGAAGGCTGTAAACAAATACTGGGACAGTTACATCCGTCCCTCCAG
ACAGGATCAGAGGAGCTTAAATCATTATATAATACAATAGCAGTTCTCTATTGTGTACATCAGAGGATAGAGG
TAAAAGACACCAAGGAAGCTTTAGAGAAGATAGAGGAAGAGCAAAACAAAAGTAAGAAAAAAGCACAGCAAGC
GGCAGCTGCTGACACAGGAACCAGCAGCAGCAGCACGGTCAGCCAAAATTACCCTATAGTGCAGAACATTCAG
GGGCAAATGGTACATCAGCCCATATCACCTAGAACTTTAAATGCATGGGTAAAAGTAGTAGAAGAGAAGGCTT
TCAGCCCAGAAGTAATACCCATGTTTTCAGCATTATCAGAAGGAGCCACCCCACAAGATTTAAACACCATGTT
AAACACAGTGGGGGGACATCAGGCAGCCATGCAAATGTTAAAAGAGACCATCAATGAGGAAGCTGCAGAATGG
GATAGATTACACCCAGTGCATGCAGGGCCTGTTGCACCAGGCCAGTTTAGAGAACCAAGGGGAAGTGACATAG
CAGGAACTACTAGTACCCTCCAGGAACAAATAGCATGGATGACACATAATCCACCTATCCCAGTAGGAGACAT
CTATAAAAAATGGATAATCATGGGATTAAATAAAATAGTGAGGATGTATAGCCCTACCAGCATTCTGGACATA
AAACAAGGACCAAAAGAACCCTTTAGAGATTATGTGGACCGGTTCTATAAAGTTTTAAGAGCCGAGCAAGCTA
CACAGGAGGTAAAAAATTGGATGACAGAAACCTTGTTGATCCAAAATGCGAACCCAGATTGTAAGACTATTCT
AAAAGCATTAGGGCCAGCAGCTTCACTAGAAGAAATGATGACAGCATGTCAGGGAGTGGGAGGACCCAGCCAT
AAAGCAAGAGTTTTGGCTGAAGCAATGAGCCAAGCAACAAATTCAGCTGTTGTGATGATGCAGAGAGGCCATG
TTAGGAACCAAAGAAAAGTCATTAAGTGTTTCAATTGTGGCAAAGAGGGGCACATAGCCAAAAATTGCAGAGC
CCCTAGGAAAAAGGGCTGTTGGAAATGTGGAAAGGAAGGACACCAAATGAAAGATTGTACTGAGAGACAGGCC
AATTTTTTAGGGAAGATCTGGCCTTCCCACAAGGGGAGGCCAGGGAATTTTCTTCAGAACAGGCCAGAGAGCA
GACTAGAGCCAACAGCCCCACCAGAGGAGAGCTTCAGGTTTGGGGAAGAGACAGCGGCTCCCTCT?AGAAGCA
GGAGCCGATAGACAAGGAACTGTATCCTTTAGCTTCCCTCAGATCACTCTTTGGCAACGACCCCTCGTCACAG
TAAAGATAGGGGGGCAATTAAAGGAAGCTCTATTAGATACAGGAGCAGATGATACAGTATTAGAAGACATGGA
TTTGCCAGGAAGATGGAAGCCAAAAATGATAGGGGGAATTGGAGGTTTTATCAAAGTAAGACAATATGATCAG
ATACCTGTAGAAATCTGTGGACATAAAGCTATAGGTACAGTATTAGTAGGACCTACACCTGTCAACATAATTG
GAAGAAATCTGTTGACTAAGATTGGTTGCACTTTAAATTTTCCCATAAGTCCTATTGAAACTGTACCAGTAAA
ATTAAAGCCAGGAATGGATGGCCCAAAAGTTAAACAATGGCCATTAACAGAAGAAAAAATAAAAGCATTGATA
GAAATTTGTACAGAAATGGAGAAGGAAGGAAAGATTTCAAAAATTGGGCCTGAAAATCCATACAATACTCCAG
TATTTGCCATAAAGAAAAAAGACAGTACTAAGTGGAGAAAATTAGTAGACTTCAGAGAACTTAATAAGAAAAC
TCAAGACTTCTGGGAAGTTCAGTTAGGAATACCACATCCCGCAGGGTTAAAAAAGAGAAAATCAGTAACAGTA
CTGGATGTGGGTGATGCATATTTTTCAGTTCCCTTAGATGAAGATTTCAGGAAATATACTGCATTTACCATAC
CTAGTACAAATAATGAGACACCAGGGATTAGATATCAATACAATGTGCTTCCACAGGGATGGAAAGGATCACC
AGCAATATTCCAAAGTAGCATGACAAAGATCTTAGAGCCTTTCAGAAAACAAAATCCAGACATAATTATCTAC
CAATACATGGATGATTTGTATGTAGGATCTGACTTAGAAATAGAGCAGCATAGAACAAAAATAGAGGAGTTAA
GACAGCATCTGTTGAGGTGGGGACTTACCACACCAGACAAAAAGCATCAGAAAGAACCTCCATTCCTTTGGAT
GGGTTATGAACTCCATCCTGACAAATGGACAGTACAGCCTATAGTGCTGCCAGAAAAGGACAGCTGGACTGTC
AATGACATACAGAAGTTAGTGGGAAAATTAAATTGGGCAAGTCAGATTTATGCAGGGATTAAGGTAAAGCAAT
TATGTAAACTCCTTAGGGGAGCCAAAGCACTGACAGAAGTAATACCACTAACAGAGGAAGCAGAACTAGAACT
GGCAGAAAACAGGGAGATTCTAAAAGAACCAGTACATGGAGTGTATTATGACCCATCAAAAGATTTAATAGCA
GAAGTACAGAAGCAGGGGCTAGGCCAATGGACATATCAAATTTATCAAGAGCCATTTAAAAATCTGAAAACAG
GAAAATATGCAAGAATGAGGGGTGCCCACACTAATGATGTAAAACAGTTAACAGAGGCAGTGCAAAAGATAGC
TACAGAAAGCATAGTAATATGGGGAAAGACTCCTAAATTTAAGCTCCCCATACAAAAAGAAACATGGGAAACA
TGGTGGACAGAATATTGGCAAGCCACCTGGATTCCTGAATGGGAGTTTGTCAATACCCCTCCCTTAGTAAAAC
TATGGTATCAGTTAGAGAAAGAACCTATAGAAGGAGCAGAAACTTTCTATGTAGATGGGGCAGCTAACAGGGA
GACTAGAATAGGAAAAGCAGGATATGTTACTAACAGAGGAAGACAAAAAGTTGTCTCCCTAACTGACACAACA
AATCAGAAGACTGAGTTACAGGCAATTTATCTGGCTTTGCAGGATTCGGGATTAGAAGTAAACATAGTAACAG
ACTCACAATATGCATTAGGAATCATTCAAGCACAACCAGATAAAAGTGAATCAGAGTTAGTCAGTCAAATAAT
AGAGCAGTTAATAAAAAAGGAAAAGGTCTACTTGGCATGGGTACCCGCACACAAAGGAATTGGAGGAAATGAG
CAAGTAGATAAGCTAGTCAGTGCCGGAATCAGGAAGGTGCTGTTTTTAGATGGAATAGATAAGGCACAAGCGG
ACCATGAAAAATATCACAGTAATTGGAGAGCAATGGCTAGTGAGTTTAACCTGCCACCTGTAATAGCAAAAGA
AATAGTAGCCTGCTGTGATAAATGTCAACTAAAAGGAGAAGCCATGCATGGGCAAGTAGACTGTAGCCCAGGA
ATATGGCAACTAGATTGTACACATTTAGAAGGAAAAATTATCCTGGTGGCAGTACATGTAGCTAGTGGATATA

TAGAAGCAGAAGTTATTCCAGCAGAGACAGGGCAGGAAACAGCTTACTTTCTGTTAAAATTAGCAGGAAGATG
GCCAGTAAAAATGATACATACAGACAATGGCGGCAATTTCACCAGTGCTGCAATGAAGGCCGCCTGTTGGTGG
GCAGGGATCAAGCAGGAATTTGGCATTCCCTACAATCCCCAAAGTCAAGGAGTAGTAGAATCTATGAATAAAG
AATTAAAGAAAATTATAGGACAGGTAAGAGATCAGGCTGAACATCTTAAGACAGCAGTACAAATGGCAGTATT
CATCCACAATTTTAAAAGAAAAGGGGGGATTGGGGAGTACAGTGCAGGGGAAAGAATAATAGACATAATAGCA
ACAGACATACAAACTAAAGAATTACAAAAACAAATTACAAAAATTCAAAATTTTCGGGTTTATTACAGGGACA
GCAGAGATCCACTTTGGAAAGGACCAGCAAAGCTTCTCTGGAAAGGTGAAGGGGCAGTAGTAATACAAGATAA
TAGTGACATAAAAGTAGTGCCAAGAAGAAAAGCAAAGATCATTAGGGATTATGGAAAACAGATGGCAGGTGAT
GATTGTGTGGCAAGTAGACAGGATGAGGATTAAAACATGGAAGAGTTTAGTAAAACACCATATGTATATCTCA
AAGAAAGCTAAGGGATGGGTTTATAGACATCACTATGAAAGCACTCATCCAAGAATAAGTTCAGAAGTATACA
TCCCACTAGGGGATGCTAAATTAGTAATAACAACATATTGGGGTCTGCATACAGGAGAAAGAGATTGGCATTT
AGGTCAGGGAGTCTCCATAGAATGGAGGAAGGAGAGATATAGCACACAGGTAGACCCTGGCCTAGCAGACCAA
CTAATTCATATGTATTACTTTGATTGTTTTTCAGAATCTGCTATAAGACATGCCATATTAGGACATAGAGTTA
GCCCTAGTTGTGAATATCAAGCAGGACATAATAAGGTAGGATCTCTACAGTACTTGGCACTAACAGCATTAAT
AACACCAAGAAAAACAAAGCCACCTTTGCCTAGTGTTACGAAACTGACAGAGGATAGATGGAACAAGCCCCGG
AAGACCAAGGGCCACAGAGGGAGCCATACAATGAATGGACACTAGAACTTTTAGAGGAGCTTAAGAGTGAAGC
TGTTAAACACTTTCCTAGGGCATGGCTCCATAGCTTAGGGCAATATATCTATGAAACTTATGGGGATACTTGG
ACAGGAGTGCAAGCCTTAATAAGAATGCTGCAACAACTGCTGTTTATTCATTTCAGAATTGGGTGTCGACACA
GCAGAATAGGCATTCAACACAGGAGGCCAAGAAATGGAGCCAGTAGATCCTAGTCTAGAACCCTGGAAAAATC
CAGGAAGTCAGCCTAGGACTGCTTGTAACACTTGCTATTGTAAAAAGTGTTGCTTTCATTGCCAAGTTTGTTT
CATAAAAAAAGCTCTAGGCATCTCCTATGGCAGGAAGAAGCGGAGACAGCGACGAAGAACTCCTCAAAACAGT
GAGACTCATCAAGCTTCTCTATCAAAGCAGTAAGTAGTTCATGTAATGCAAGCTTTAGTGATATCAGCAATAG
TAGGATTAGTAGTAGCAGCTATACTAGCAATAATTGTATGGACCTTAGTACTGATAGAATATAGGAAAATATT
AAGACAAAGGAAAATAGACAGGTTAATTGATAGAATAAGAGAAAGAGCAGAAGATAATGGCAATGAGAGTGAA
GGGGATCAGGAGAAATTGTCAGCACTTGTGGAAATGGGGCACGATGCTCCTTGGGATATTGATGATCTGTAGT
GCTTCAGAACAATTGTGGGTCACAGTCTATTATGGGGTACCTGTGTGGAAAGAAGCAAACACCACTCTATTTT
GTGCATCAGATGCTAAAGCATATGATACAGAGGTGCATAATGTTTGGGCCACACATGCATGTGTACCCACAGA
CCCTAACCCACAAGAAGTAACATTGGAAAATGTAACAGAAAAGTTTGACATGTGGAATAATAACATGGTAGAA
CAGATGCATGAGGATGTAATCAGTTTATGGGATCAAAGCTTAAAGCCATGTGTAAAACTAACCCCACTCTGTG
TTACTTTAGATTGCACTAATGCGACTGTTATTGGTACCGATACTCATACCAATAGTAGTGTCAGGGAAGACAT
GAAAAACTGCACTTTCAATATCACCACAAGCTTAAGAGATAAGATGCAAAGGGTATATTCACTTTTTTATAAA
GAGGATATAGAACCAATAAAGAATCCTGATGGTCCTAATGCCAACACTAGTAGTAATGATACTGATAGTCCTA
ATACCAGCTATAGGTTGACAAAGTGTAACACCTCAATCATTACACAAGCCTGTCCAAAGGTATCCTTTGAGCC
AATTCCCATACATTATTGTGCCCCGGCTGGTTTTGCAATTATAAAATGTAAGGATAAGAACTTCACTGGAACA
GGACTATGTAAAAATGTCAGCACAGTACAGTGTACACATGGAATTAGGCCAGTAGTGTCAACTCAACTATTGT
TAAATGGTAGTCTAGCAGAAGAAGAGATAATAATTAGAACTAAAAACCTTACAGATAATACTAAAAACATAAT
AGTACAGCTGAATAAAACTGTGACAATTCTCTGTATAAGACCCGGCAACAACACAAGAAAAAGCCTACACATG
GGACCAAAGAGAGCATTTTATGCATCAAATGGTATAATAGGGGACACAAGACAAGCACATTGTAACATTAGTA
AAGACGGATGGTATAAAACTTTAGAAAAGGTAGCTAGCAAAATAAGAGAACAATTTAGTAAAATAAACAGAAG
TTATGAGAATGATACAATAAGCTTTAGGCCACCATCCCCAGGAGGGGACCCAGAAATTATGATGCACAGTTTT
AATTGTGGAGGGGAATTTTTCTACTGTGATACAACACAACTGTTTAATAGTACTTGGCATAAGAATAACACCA
ATAGCACCGATGGAAGGGGGCAAGAACACATCACACTCCCATGCAGAATAAGACAAATTGTAAACATGTGGCA
GGAAGTAGGAAAAGCAATGTATGCTCCTCCCATCAGAGGAAACATCACATGTTCATCAAATATTACAGGGCTG
CTATTAACATGGGATGGTGGGAATGGGAATGAGACCAACAATCAGACCTTCAGACCTATAGGAGGAGATATGA
GGGACAATTGGAGAAGTGAATTATATAAATACAAAGTAGTAAAAATTGAACCATTAGGAATAGCACCCACCAA
GGCAAAAAGAAGAGTGGTGCAGAGAGAAAAAAGAGCAGTGGGATTATTAGGAGCTGTGTTCCTTGGGTTCTTG
GGAGCAGCAGGAAGCACTATGGGCGCAGCGTCAATAACGCTGACGGCACAAGCCAGACAGTTAATGTCTGGTA
TAGTGCAACAGCAGAACAATCTGCTGAGAGCTATTGAGGCGCAACAGCATATGTTGCAACTCACAGTCTGGGG
CATCAAGCAGCTCCAGGCAAGAGTCCTGGCTCTGGAAAGATACCTAGCGGATCAACAGCTCCTAGGACTTTGG
GGTTGCTCTGGAAAACTCATCTGCCCCACTGCTGTGCCTTGGAATGTTAGTTGGAGTAATAAATCTCTGGAAG
ACATTTGGAATAACATGACCTGGATGCAGTGGGAAAGAGAAATTAATAATTATACAGATATAATATACTCCTT
ACTTGAAGGAGCGCAGATCCAGCAAGAGAAGAATGAAGAAGACTTATTAAAATTAGATCAATGGGCAAATTTG
TGGAATTGGTTTGACATAACCCAATGGCTGTGGTATATAAGAATATTCATAATGGTAGTAGGAGGCTTAATAG
GTTTAAGAATAGTTTTTGCTGTACTTTCTATAGTAAATAGAGTTAGGCAGGGATACTCACCCTTGTCGTTACA

GATCCCCCTCCCAATCCCGAGGGAACCCGACAAGCCCGACGGAATCGAAGAAGAAGGTGGAGAGAGAGACAGA
GACAGATCCGCAAGATTAGTGACCGGATTCTTGCCACTTATCTGGGACGATCTGAGGAGCCTGTGCCTCTTCA
GCTACCACCGCTTGAGAGACTTACTCTTGATTGTAACGAGGATTGTGGAACTTCTGGGACGCAGGGGGTGGGA
AATCCTCAAATATTGGTGGAATCTCCTGACCTATTGGAGCCAGGAACTAAAGAATAGTGCTGTTAGCTTGCTT
AACGCCACAGCCATAACAGTAGCTGAGGGAACAGACAGGGTTATAGAAGCATTACTAAGAGCTTGGAGGGCTC
TTCTCCACATACCTAGAAGAATAAGACAGGGCTTAGAAAGGGCTTTGCTATAAAATGGGTGGCAAATGGTCCA
AACGTAGTGGGGGTGGATGGTCTACTATAAGGGAAAGAATGAGACAAACTCCACCTGAACCTGAACCAGCAGC
AGAGGGGGTGGGAGCAGTATCTCGAGACCTAGAAAAACATGGAGCAATCACAAGTAGCAATACAGCAGCTACC
AATGCTGCTTGTGCCTGGCTAGAAGCACAAGAGGAGGAGGAGGTGGGCTTTCCAGTCAGACCCCAGGTACCTT
TAAGACCAATGACCTACAAGGGAGCTCTGGATCTTAGCCACTTTTTAAAAGAAAAGGGGGGACTGGAAGGGCT
AATTTACTCCCAAAAGAGACAAGATATTCTTGATCTGTGGGTCTATCACACACAAGGCTACTTCCCTGATTGG
CAGTGTTACACACCAGGGCCAGGGACTAGATTCCCATTGACCTTTGGGTGGTGCTTCAAGCTAGTACCAGTCG
AGCCAGAGAAGATAGAAGAGGCCACTGAAGGAGAGAACAACATACTGTTACACCCTATGAATCTGCATGGGAT
GGATGACCCAGAGAGAGAAGTGTTACAGTGGAAGTTTGACAGTCACCTAGCATTCCGTCACGTGGCCAAAGAG
CTGCATCCGGAGTACTTCAAGAACTGAACTGCTGACACCGAGCACTGAACTGCTGACATCGAGCTTTCTACAA
GGGACTTTCCGCTGGGGACTTTCCAGGGGAGGCGTGGCCTGGGCGGGACTGGGGAGTGGCGAGCCCTCAGATG
CTGCATATAAGCAGCCGCTTTTCGCTTGTACTGGGTCTCTCTGGTTAGACCAGATCTGAGCCTGGGAGCTCTC
TGGCTAGCTAGGGAACCCACTGCTTAAGCCTCAATAAAGCTTGCCT

>S-1B1 HIV-1 genome, derived from RNA genomic sequence
GCAGTGGCGCCCGAACAGGGACCTGAAAGCGAAAGGAAAACCGGAGGAGCTCTCTCGACGCAGGACTCGGCTT
GCTGAAGCGCGCACGGCAAGAGGCGAGGGGCGGCGACTGGTGAGTACGCCAAAAATCTTGACTAGCGGAGGCT
AGAAGGAGAGAGATGGGTGCGAGAGCGTCAATATTAAGCGGGGGAGAATTAGATAGATGGGAAAAAATTCGGT
TAAGGCCAGGAGGAAAGAAAAAATATAGACTAAAACATTTAGTATGGGCAAGCAGGGAGCTAGAACGATTCGC
AGTCAATCCTGGTCTGTTAGAAACATCAGAAGGCTGTAGACAAATACTGGGACAGCTACATCCGTCCCTCCAG
ACAGGATCAGAGGAGCTTAAATCATTATATAATACAATAGCAGTTCTCTATTGTGTGCATCAAAGGATAGAGG
TAAAAGACACCAAGGAAGCCTTAGAGAAGATAGAGGAAGAGCAAAACAAATGTAAGAAAAAGGCACAGCAAGC
GGCAGCTGCTGACACAGGAACCAGCAGCAGCAGCACGGTCAGTCAAAATTACCCTATAGTGCAGAACATTCAG
GGGCAAATGGTACATCAGCCCATATCACCTAGAACTTTAAATGCATGGGTAAAAGTAGTAGAAGAGAAGGCTT
TCAGCCCAGAAGTAATACCCATGTTTTCAGCATTATCAGAAGGAGCCACCCCACAAGATTTAAACACCATGTT
AAACACAGTGGGGGGACATCAGGCAGCCATGCAAATGTTAAAAGAGACCATCAATGAGGAAGCTGCAGAATGG
GATAGATTACACCCAGTGCATGCAGGGCCTGTTGCACCAGGCCAGTTTAGAGAACCAAGGGGAAGTGACATAG
CAGGAACTACTAGTACCCTCCAGGAACAAATAGCATGGATGACACATAATCCACCTATCCCAGTAGGAGACAT
CTATAAAAAATGGATAATCATGGGACTAAATAAAATAGTGAGGATGTACAGCCCTACCAGCATTCTGGACATA
AAACAAGGACCAAAAGAACCCTTTAGAGATTATGTAGACCGGTTCTATAAGGTTTTAAGAGCCGAGCAAGCTA
CACAGGAGGTAAAAAATTGGATGACAGAAACCTTGTTGGTCCAAAATGCGAACCCAGATTGTAAGACTATTCT
AAAAGCATTAGGGCCAGCAGCTTCACTAGAAGAAATGATGACAGCATGTCAGGGAGTGGGAGGTCCCAGCCAT
AAAGCAAGAGTTTTGGCTGAAGCAATGAGCCAAGCAACAAATTCAGCTGTTGTGATGATGCAGAGAGGCAATG
TTAGGAGCCAAAGAAAAATTATTAAGTGTTTCAATTGTGGCAAAGAGGGGCACATAGCTAAAAATTGCAGAGC
CCCTAGGAAAAAGGGCTGTTGGAAATGTGGAAAGGAAGGACACCAAATGAAAGATTGTACTGAGAGACAGGCA
AATTTTTTAGGGAAAATCTGGCCTTCCCACAAGGGGAGGCCAGGGAATTTTCTTCAGAACAGACCAGAGAGCA
GACTAGAGCCAACAGCCCCACCAGAGGAGAGCTTCAGGTTTGGGGAAGAGACAGCGGCTCCCTCTCAGAAGCA
GGAGCCGATAGACAAGGAACTGTATCCTTTAGCTTCCCTCAGATCACTCTTTGGCAACGACCCCTCGTCACAG
TAAAGATAGGGGGGCAATTAAAGGAAGCTCTATTAGATACAGGAGCAGATGATACAGTATTAGAAGACATGGA
TTTGCCAGGAAGATGGAAGCCAAAAATGATAGGGGGAATTGGAGGTTTTATCAAAGTAAGACAATATGATCAG
ATACCTGTAGAAATCTGTGGACATAAAGCTATAGGTACAGTATTAGTAGGACCTACACCTGTCAACATAATTG
GAAGAAATCTGTTGACTAAGATTGGTTGCACTTTAAATTTTCCCATAAGTCCTATTGAAACTGTACCAGTAAA
ATTAAAGCCAGGAATGGATGGCCCAAAAGTTAAACAATGGCCATTGACAGAAGAGAAAATAAAAGCATTGATA
GAAATTTGTACAGAAATGGAGAAGGAAGGAAAGATTTCAAAAATTGGGCCTGAAAATCCATACAATACTCCAG
TATTTGCCATAAAGAAAAAAGACAGTACTAAGTGGAGAAAATTAGTAGACTTCAGAGAACTTAATAAGAAAAC
TCAAGACTTCTGGGAAGTTCAATTAGGAATACCACATCCCGCGGGGTTAAAAAAGAGAAAATCAGTAACAGTA
CTGGATGTGGGTGATGCATATTTTTCAATTCCCTTAGATGAAGATTTCAGGAAATATACTGCATTTACCATAC
CCAGTACAAATAATGAGACACCAGGGATTAGGTATCAATACAATGTGCTCCCACAGGGATGGAAAGGATCACC
AGCAATATTCCAAAGTAGCATGACAAAGATCTTAGAGCCTTTTAGAAAACAAAATCCAGACATAATTATTTAC
CAATACATGGATGATTTGTATGTAGGATCTGACTTAGAAATAGAGCAGCATAGAACAAAAATAGAGGAATTGA
GACAGCATCTGTTGAGGTGGGGACTCACCACACCCGACAAAAAGCATCAGAAAGAACCTCCATTCCTTTGGAT
GGGTTATGAACTCCATCCTGACAAATGGACAGTACAGCCTATAGTGCTGCCAGAAAAGGACAGCTGGACTGTC
AATGACATACAGAAGTTAGTGGGAAAATTAAATTGGGCAAGTCAGATTTATGCAGGGATTAAAGTAAAGCAAT
TATGTAAACTCCTTAGGGGAGCCAAAGCACTGACAGAAGTAATACCACTGACAGAGGAAGCAGAACTAGAACT
GGCAGAAAACAGGGAGATTCTAAAAGAACCAGTACATGGAGTATATTATGACCCATCAAAAGACTTAATAGCA
GAAGTACAAAAGCAGGGGCTAGGCCAATGGACATATCAAATTTATCAAGAGCCATTTAAAAATCTGAAAACAG
GAAAATATGCAAGAATAAGGGGTGCCCACACTAATGATGTAAAACAATTAACAGAGGCAGTGCAAAAGATAGC
TACAGAAAGCATAGTAATATGGGGAAAGACTCCTAAATTTAAGCTCCCCATACAAAAGGAAACATGGGAAACA
TGGTGGACAGAATATTGGCAAGCCACCTGGATTCCTGAATGGGAGTTTGTCAATACCCCTCCCTTAGTAAAAT
TATGGTATCAGTTAGAGAAAGAACCTATAGAAGGAGCAGAAACTTTCTATGTAGATGGGGCAGCTAACAGGGA
GACTAGATTAGGGAAAGCAGGATATGTTACGAACAGAGGAAGACAAAAAGTTGTCTCCCTAACTGACACAACA
AATCAGAAGACTGAGTTACAGGCAATTTATCTGGCTTTGCAGGATTCAGGATTAGAAGTGAACATAGTAACAG
ACTCACAATATGCATTAGGAATCATTCAAGCACAACCAGATAAAAGTGAATCAGAGTTAGTCAGTCAAATAAT
AGAGCAGTTAATAAAAAAGGAAAAGGTCTACTTGGCATGGGTACCCGCACACAAAGGAATTGGAGGAAATGAG
CAAGTAGATAAGCTAGTCAGTGCCGGAATCAGGAAGGTGCTGTTTTTAGATGGAATAGATAAGGCACAAGCGG
ACCATGAAAAATATCACAGTAATTGGAGAGCAATGGCTAGTGAGTTTAACCTGCCACCTGTAGTAGCAAAAGA
AATAGTAGCCTGCTGTGATAAATGTCAACTAAAAGGAGAAGCCATGCATGGGCAAGTAGACTGTAGTCCAGGA
ATATGGCAACTAGATTGTACACATTTAGAAGGAAAATTATCCTGATGGCAGTGCATGTAGCTAGTGGATATA

TAGAAGCAGAAGTTATCCCAGCAGAGACAGGGCAGGAAACAGCTTACTTTCTGTTAAAATTAGCAGGAAGATG
GCCAGTAAAAATGATACATACAGACAATGGCGGCAATTTCACCAGTACTGCAATGAAGGCCGCCTGTTGGTGG
GCAGGGATCAAGCAGGAATTTGGCATTCCCTACAATCCCCAAAGTCAAGGAGTAGTAGAATCTATGAATAAAG
AATTAAAGAAAATTATAGGACAGGTAAGAGATCAGGCTGAACATCTTAAGACAGCAGTACAAATGGCAGTATT
CATCCACAATTTTAAAAGAAAAGGGGGGATTGGGGAGTACAGTGCAGGGGAAAGAATAATAGACATAATAGCA
ACAGACATACAAACTAAAGAATTACAAAAACAAATCACAAAAATTCAAAATTTTCGGGTTTATTACAGGGACA
GCAGAGATCCACTTTGGAAAGGACCAGCAAAGCTTCTCTGGAAAGGTGAAGGGGCAGTAGTAATACAAGATAA
TAGTGACATAAAAGTAGTGCCAAGAAGAAAAGCAAAGATCATTAGGGATTATGGAAAACAGATGGCAGGTGAT
GATTGTGTGGCAGGTAGACAGGATGAGGATTAAAACATGGAAGAGTTTAGTAAAACACCATATGTATATCTCA
AAGAAAGCTAAGGGATGGGTTTATAGACATCACTATGAAAGCACTCATCCAAGAATAAGTTCAGAAGTATACA
TCCCACTAGGGGATGCTAAATTAGTAATAACAACATATTGGGGTCTGCATACAGGAGAAAGAGATTGGCATTT
AGGTCAGGGAATCTCCATAGAATGGAGGAAGGAGAGATATAGCACACAGGTAGACCCTGGCCTAGCAGACCAA
CTAATTCATATGTATTACTTTGATTGTTTTTCAGAATCTGCTATAAGACATGCCATATTAGGACATAGAGTTA
GCCCTAGTTGTGAATATCAAGCAGGACATAACAAGGTAGGATCGCTACAGTACTTGGCACTAACAGCATTAAT
AACACCAAGAAAAACAAAGCCACCTTTGCCTAGTGTTACGAAACTGACAGAGGATAGATGGAACAAGCCCCGG
AAGACCAAGGGCCACAGAGGGAGCCATACAATGAATGGACACTAGAACTTTTAGAGGAGCTTAAGAGTGAAGC
TGTTAAACACTTTCCTAGGGCATGGCTCCATAGCTTAGGGCAATATATCTATGAAACTTATGGGGATACTTGG
ACAGGAGTGCAAGCCTTAATAAGAACGCTGCAACAACTGCTGTTTATTCATTTCAGAATTGGGTGTCGACACA
GCAGAATAGGCATTCAACACAGGAGGCCAAGAAATGGAGCCAGTAGATCCTAGCCTAGAACCCTGGAAGCATC
CAGGAAGTCAGCCTAGGACTGCTTGTAACACTTGCTATTGTAAAAAGTGTTGCTTTCATTGCCAAGTTTGTTT
CATAAAAAAAGCTCTAGGCATCTCCTATGGCAGGAAGAAGCGGAGACAGCGACGAAGAACTCCTCAAAACAGT
GAGACTCATCAAGCTTCTCTATCAAAGCAGTAAGTAGTTCATGTAATGCAAGCTTTAGTGATATCAGCAATAG
TAGGATTAGTAGTAGCAGCTATACTAGCAATAATTGTATGGACCTTAGTACTGATAGAATATAGGAAAATATT
AAGACAAAGGAAAATAGACAGGTTAATTGATAGAATAAGAGAAAGAGCAGAAGATAGTGGCAATGAGAGTGAA
GGGGATCAGGAGGAATTGTCGGCACTTGTGGAAATGGGGCACGATGCTCCTTGGGATATTGATGATCTGTAGT
GCTTCAGAACAATTGTGGGTCACAGTCTATTATGGGGTACCTGTGTGGAAAGAAGCAAACACCACTCTATTTT
GTGCATCAGATGCTAAAGCATATGATACAGAGGTGCATAATGTTTGGGCCACACATGCATGTGTACCCACAGA
CCCTAACCCACAAGAAGTAACATTGAAAAATGTAACAGAAAATTTTGACATGTGGAATAATAACATGGTAGAA
CAGATGCATGAGGATGTAATCAGTTTATGGGATCAAAGCTTAAAGCCATGTGTAAAACTAACCCCACTCTGTG
TTACTTTAGATTGCACTAATGCGACTGTTATTGGTACCGCTAATACCAATAGTAGTGTCAGGGAAGACATGAA
AAACTGCACTTTCAATATCACCACAAGCTTAAGAGATAAGATGCAAAGGGTATATTCACTTTTTTATAAAGAG
GATATAGAACCAATAAAGAATCTTGATGATCCTAATGCCAATAGTACTAATAGTTCTAGTACCAGCTATAGGT
TGACAAAGTGTAACACCTCAGTCATTACACAAGCCTGTCCAAAGGTATCCTTTGAGCCAATTCCCATACATTA
TTGTGCCCCGGCTGGTTTTGCAATCATAAAATGTAAGGATAAGAACTTCACTGGAACAGGACTATGTAAAAAT
GTCAGCACAGTACAGTGTACACATGGAATTAGGCCAGTAGTATCAACTCAACTATTGTTAAATGGTAGTCTAG
CAGAAGAAGAGATAATAATTAGAACTAAAAACCTTACAGATAATACTAAAAACATAATAGTACAGCTGAATAA
AACTGTGGAAATTCGCTGTATAAGACCCGGCAACAACACAAGAAAAAGCCTACACATGGGACCAAAGAGAGCA
TTTTATGCATCAAATGGTATAATAGGGGACACAAGACAGGCACATTGTAACGTTAGTGAAAAAGACTGGGAGG
AGACTTTAGCAAAAGTAGCTAGCAAAATAAGAGAACAATTTAGTAAAATAAACAGAAGTTATGAGAATGATAC
AATAACCTTTAGGCCACCATCCCCAGGAGGGGACCCAGAAATTATGATGCACAGTTTTAATTGTGGAGGGGAA
TTTTTCTACTGTGATACAATACGACTGTTTAATAGCGAATGGAATACGAGTAGCGCTTGGGATTGGAATAGCA
CCAAGAATGGCACCAACATCACACTCCAGTGCAGAATAAGACAAATTGTAAACATGTGGCAGGAAGTAGGAAA
AGCAATGTATGCTCCTCCCATCAGAGGAAACATCACATGTTTATCAAATATTACAGGGCTGCTATTAACATGG
GATGGTGGGAATGGGAATGAGACCAACAATCAGACCTTCAGACCTATAGGAGGAGATATGAGGGACAATTGGA
GAAGTGAATTATATAAATACAAAGTAGTAAAAATTGAACCATTAGGAATAGCACCCACCAAGGCAAAAAGAAG
AGTGGTGCAGAGAGAAAAAAGAGCAGTGGGATTATTAGGAGCTGTGTTCCTTGGGTTCTTGGGAGCAGCAGGA
AGCACTATGGGCGCAGCGTCAATAACGCTGACGGCACAAGCCAGACAGTTAATGTCTGGTATAGTGCAACAGC
AGAACAATCTGCTGAGAGCTATTGAGGCGCAACAGCATATGTTGCAACTCACAGTCTGGGGCATCAAGCAGCT
CCAGGCAAGAGTCCTGGCTCTGGAAAGATACCTAGCGGATCAACAGCTCCTGGGAATTTGGGGTTGCTCTGGA
AAACTCATCTGCACCACTGCTGTGCCTTGGAATGTTAGTTGGAGTAATAAATCTCTGGAAGACATTTGGAATA
ACATGACCTGGATGCAGTGGGAAAGAGAAATTAATAATTATACAGATATAATATACTCCTTACTTGAAGGAGC
GCAGATCCAGCAAGAGAAGAATGAAGAAGACTTATTAAAATTAGATCAATGGGCAAATTTGTGGAATTGGTTT
GACATAACCCAATGGCTGTGGTATATAAGAATATTCATAATGGTAGTAGGAGGCTTAATAGGTTTAAGAATAG
TTTTTGCTGTACTTTCTATAGTGAATAGAGTTAGGCAGGGATACTCACCCTTGTCGTTACAGATCCCCCTCCC

AATCCAGAGGGAACCCGACAAGCCCGAAGGAATA?AAGAAGAAGGTGGAGAGAGAGGCAGAGACAGATCCGCA
AGATTAGTGACCGGATTCTTGCCACTTATCTGGGACGATCTGAGGAGCCTGTGCCTCTTCAGCTACCACCGCT
TGAGAGACTTACTCTTGATTGTAACGAGGATTGTGGAACTTCTGGGACGCAGGGGGTGGGAAATCCTCAAATA
TTGGTGGAATCTCCTGACCTATTGGGGCCAGGAACTAAAGAATAGTGCTGTTAGCTTGCTCAACGCCACAGCC
ATAACAGTAGCTGAGGGAACAGACAGGGTTATAGAAGCATTACTAAGGGCTTGGAGGGCTATTCTCCACATAC
CTAGAAGAATAAGACAGGGCTTAGAAAGGGCTTTGCTATAAGATGGGTGGCAAATGGTCCAAATGTAGTGGGG
GTGGATGGTCTACTATAAGGGAAAGAATGAGACAAACTCCACCTGAACCTGAACCAGCAGCAGAAGGGGTGGG
AGCAGTATCTCGAGACCTAGAAAGACATGGAGCAATCACAAGTAGCAATACAGCAGCTACCAATGCTGCTTGT
GCCTGGCTAGAAGCACAAGAGGAGAAGGAGGTGGGCTTTCCAGTCAGACCCCAGGTACCTTTAAGACCAATGA
CCTACAAGGGAGCTTTGGATCTTAGCCACTTTTTAAAAGAAAAGGGGGGACTGGAAGGGCTAATTTACTCCCA
AAAGAGACAAGATATTCTTGATCTGTGGGTCTATCACACACAAGGCTACTTCCCTGATTGGCAGTGCTACACA
CCAGGGCCAGGGACTAGATTCCCATTGACCTTTGGGTGGTGCTTCAAGCTAGTACCAGTCGAGCCAGAGAAGA
TAGAAGAGGCCACTGAAGGAGAGAACAACATACTGTTACACCCTATGAATCTGCATGGGATGGATGACCCAGA
GAGAGAAGTGCTACAGTGGAAGTTTGACAGTCACCTAGCATTCCGTCACGTGGCCAAAGAGCTGCATCCGGAG
TACTTCAAGAACTGAACTGCTGACATCGAGCACTGAACTGCTGACATCGAGCTTTCTACAAGGGACTTTCCGT
TGGGGACTTTCCAGGGGAGGCGTGGCCTGGGCGGGACTGGGGAGTGGCGAGCCCTCAGACGCTGCATATAAGC
AGCCGCTTTTCGCTTGTACTGGGTCTCTCTGGTTAGACCAGATCTGAGCCTGGGAGCTCTCTGGCTAGCTAGG
GAACCCACTGCTTAAAGCCTCAATAAAGCTTGCCT

>S-2B1 HIV-1 genome, derived from RNA genomic sequence
GCAGTGGCGCCCGAACAGGGACCTGAAAGCGAAAGGAAGACCGGAGGAGCTCTCTCGACGCAGGACTCGGCTT
GCTGAAGCGCGCACGGCAAGAGGCGAGGGGCGGCGACTGGTGAGTACGCCAAAAATTTTGACTAGCGGAGGCT
AGAAGGAGAGAGATGGGTGCGAGAGCGTCAATATTAAGCGGGGGAGAATTAGATAGATGGGAAAAAATTCGGT
TAAGGCCAGGGGGAAAGAAAAAATATAGACTAAAACATTTAGTATGGGCAAGCAGGGAGCTAGAACGATTCGC
AGTCAACCCTGGCCTGTTAGAAACATCAGAAGGCTGTAGACAAATACTGGGACAGCTACATCCGTCCCTCCAG
ACAGGATCAGAGGAGCTTAAATCATTATATAATACAATAGCAGTCCTCTATTGTGTGCATCAAAGGATAGAGG
TAAAAGACACCAAGGAAGCTTTAGAGAAGATAGAGGAAGAGCAAAACAAATGTAAGAAAAAAGCACAGCAAGC
GACAGCTGCTGACACAGGAACCAGCAGCAGCAGCACGGTCAGCCAAAATTACCCTATAGTGCAGAACATTCAG
GGGCAAATGGTACATCAGCCCATATCACCTAGAACTTTAAATGCATGGGTAAAAGTAGTAGAAGAGAAGGCTT
TCAGCCCAGAAGTAATACCCATGTTTTCAGCATTATCAGAAGGAGCCACCCCACAAGATTTAAACACCATGTT
AAACACAGTGGGGGGACATCAGGCAGCCATGCAAATGTTAAAAGAGACCATCAATGAGGAAGCTGCAGAATGG
GATAGATTACACCCAGTGCATGCAGGGCCTGTTGCACCAGGCCAGTTTAGAGAACCAAGGGGAAGTGACATAG
CAGGAACTACTAGTACCCTCCAGGAACAAATAGCATGGATGACACATAATCCACCTATCCCAGTAGGAGACAT
CTATAAAAAATGGATAATCATGGGATTAAATAAAATAGTGAGGATGTATAGCCCTACCAGCATTCTGGACATA
AAACAAGGACCAAAAGAACCCTTTAGAGATTATGTAGACCGGTTCTATAAAGTTTTAAGAGCCGAGCAAGCTA
CACAGGAGGTAAAAAATTGGATGACAGAAACCTTGTTGGTCCAGAATGCGAACCCAGATTGTAAGACTATTCT
AAAAGCATTAGGGCCAGCAGCTTCACTAGAAGAAATGATGACAGCATGTCAGGGAGTGGGAGGTCCCAGCCAT
AAAGCAAGAGTTTTGGCTGAAGCAATGAGCCAAGCAACAAATTCAGCTGTTGTGATGATGCAGAGAGGCAATG
TTAGGAGCCAAAGAAAAGTTATTAAGTGTTTCAATTGTGGCAAAGAGGGGCACATAGCTAAAAATTGCAGAGC
CCCTAGGAAAAAGGGCTGTTGGAAATGTGGAAAGGAAGGACACCAAATGAAAGATTGTACTGAGAGACAGGCC
AATTTTTTAGGGAAAATCTGGCCTTCCCACAAGGGGAGGCCAGGGAATTTTCTCCAGAACAGACCAGAGAGCA
GACTAGAGCCAACAGCCCCACCAGAGGAGAGCTTCAGGTTTGGGGAAGAGACAGCGGCTCCCTCTCAGAAGCA
GGAGCCGATAGACAAGGAACTGTATCCTTTAGCTTCCCTCAGATCACTCTTTGGCAACGACCCCTCGTCACAG
TAAAGATAGGGGGGCAATTAAAAGAAGCTCTACTAGATACAGGAGCAGATGATACAGTATTAGAAGACATGGA
TTTGCCAGGAAGATGGAAGCCAAAAATGATAGGGGGAATTGGAGGTTTTATCAAAGTAAGACAATATGATCAG
ATACCTGTAGAAATCTGTGGACATAAAGCTATAGGTACAGTATTAGTAGGACCTACACCTGTCAACATAATTG
GAAGAAATCTGTTGACTAAGATTGGTTGCACTTTAAATTTTCCCATAAGTCCTATTGAAACTGTACCAGTAAA
ATTAAAGCCAGGAATGGATGGCCCAAAAGTTAAACAATGGCCATTGACAGAAGAGAAAATAAAAGCATTGATA
GAAATTTGTACAGAAATGGAAAAGGAAGGAAAAATTTCAAAAATTGGGCCTGAAAATCCATACAATACTCCAG
TATTTGCCATAAAGAAAAAAGACAGTACTAAGTGGAGAAAATTAGTAGACTTCAGAGAACTTAATAAGAAAAC
TCAAGACTTCTGGGAAGTTCAATTAGGAATACCACATCCCGCAGGGTTAAAAAAGAGAAAATCAGTAACAGTA
CTGGATGTGGGTGATGCATATTTTTCAGTTCCCTTAGATGAAGATTTCAGGAAGTATACTGCATTTACCATAC
CTAGTACAAATAATGAGACACCAGGGATTAGATATCAATACAATGTGCTCCCACAGGGATGGAAAGGATCACC
AGCAATATTCCAAAGTAGCATGACAAAGATCTTAGAGCCTTTTAGAAAACAAAATCCAGATATAATTATTTAC
CAATACATGGATGATTTGTATGTAGGATCTGACTTAGAAATAGAGCAGCATAGAACAAAAATAGAGGAATTGA
GACAGCATCTGTTAAGGTGGGGACTTACCACACCAGACAAAAAGCATCAGAAAGAACCTCCATTCCTTTGGAT
GGGTTATGAACTCCATCCTGACAAATGGACAGTACAGCCTATAGTGCTGCCAGAAAAGGACAGCTGGACTGTC
AATGACATACAGAAGTTAGTGGGAAAATTAAATTGGGCAAGTCAGATTTATGCAGGGATTAAAGTAAAGCAAT
TATGTAAACTCCTTAGGGGAGCCAAAGCACTGACAGAAGTAATACCACTAACAGAGGAAGCAGAATTAGAACT
GGCAGAAAACAGGGAGATTCTAAGAGAACCAGTACATGGAGTGTATTATGACCCATCAAAAGATTTAATAGCA
GAAGTACAGAAGCAGGGGCTAGGCCAATGGACATATCAAATTTATCAAGAGCCATTTAAAAATCTGAAAACAG
GAAAATATGCAAGAATGAGGGGTGCCCACACTAATGATGTAAAACAATTAACAGAGGCAGTGCAAAAAATAGC
TACAGAAAGCATAGTAATATGGGGAAAGACTCCTAAATTTAAGCTCCCCATACAAAAAGAAACATGGGAAACA
TGGTGGACAGAATATTGGCAAGCCACCTGGATTCCTGAATGGGAGTTTGTCAATACCCCTCCCTTAGTAAAAC
TATGGTACCAGTTAGAGAAAGAACCTATAGAAGGAGCAGAAACTTTCTATGTAGATGGGGCAGCTAACAGGGA
GACTAGATTAGGAAAAGCAGGATATGTTACTAACAGAGGAAGACAAAAAGTTGTCTCCCTAACTGACACAACA
AATCAGAAGACTGAGTTACAGGCAATTTACCTGGCTTTGCAGGATTCGGGATTAGAAGTGAACATAGTAACAG
ACTCACAATATGCATTAGGAATCATTCAAGCACAACCAGATAAAAGTGAATCAGAGTTAGTCAGCCAAATAAT
AGAGCAGTTAATAAAAAAGGAAAAGGTCTACTTGGCATGGGTACCCGCACACAAAGGAATTGGAGGAAATGAG
CAAGTAGATAAGCTAGTCAGTGCTGGAATCAGGAAGGTACTGTTTTTAGATGGAATAGATAAGGCACAAGCGG
ACCATGAAAAATATCACAGTAATTGGAGAGCAATGGCTAGTGAGTTTAACCTGCCACCTGTAGTAGCAAAAGA
AATAGTAGCCTGCTGTGATAAATGTCAGTTAAAAGGAGAAGCCATGCATGGGCAAGTAGACTGTAGTCCAGGA
ATATGGCAACTAGATTGTACACATTTAGAAGGAAAATTATCCTGGTGGCAGTGCATGTAGCCAGTGGATATA

TAGAAGCAGAAGTTATTCCAGCAGAGACAGGGCAGGAAACAGCTTACTTTCTGTTAAAATTAGCAGGAAGATG
GCCAGTAAAAATGATACATACAGACAATGGCGGCAATTTCACCAGTGCTGCAATGAAGGCCGCCTGTTGGTGG
GCAGGGATCAAGCAGGAATTTGGCATTCCCTACAATCCCCAAAGTCAAGGAGTAGTAGAATCTATGAATAAAG
AATTAAAGAAAATTATAGGACAGGTAAGAGATCAGGCTGAACATCTTAAGACAGCAGTACAAATGGCAGTATT
CATCCACAATTTTAAAAGAAAAGGGGGGATTGGGGAGTACAGTGCAGGGGAAAGAATAATAGACATAATAGCA
ACAGACATACAAACTAAAGAATTACAAAAACAAATTACAAAAATTCAAAATTTTCGGGTTTATTACAGGGACA
GCAGAGATCCACTTTGGAAAGGACCAGCAAAGCTTCTCTGGAAAGGTGAAGGGGCAGTAGTAATACAAGATAA
TAGTGACATAAAAGTAGTGCCAAGAAGAAAAGCAAAGATCATTAGGGATTATGGAAAACAGATGGCAGGTGAT
GATTGTGTGGCAGGTAGACAGGATGAGGATTAAAACATGGAAGAGTTTAGTAAAACACCATATGTATATCTCA
AAGAAAGCTAAGGGATGGGTTTATAGACATCACTATGAAAGCACTCATCCAAGAATAAGTTCAGAAGTATACA
TCCCACTAGGGGATGCTAAATTAGTAATAACAACATATTGGGGTCTGCATACAGGAGAAAGAGATTGGCATTT
AGGTCAGGGAGTCTCCATAGAATGGAGGAAGGAGAGATATAGCACACAGGTAGACCCTGGCCTAGCAGACCAA
CTAATTCATATGTATTACTTTGATTGTTTTTCAGAATCTGCTATAAGGCATGCCATATTAGGACATAGAGTTA
GCCCTAGTTGTGAATATCAAGCAGGACATAACAAGGTAGGATCTCTACAGTACTTGGCACTAATAGCATTAAT
AACACCAAGAAAAACAAAGCCACCTTTGCCTAGTGTTACGAAACTGACAGAGGATAGATGGAACAAGCCCCGG
AAGACCAAGGGCCACAGAGGGAGCCATACAATGAATGGACACTAGAACTTTTAGAGGAGCTTAAGAGTGAAGC
TGTTAAACACTTTCCTAGGGCATGGCTCCATAGCTTAGGGCAATATATCTATGAAACTTATGGGGATACTTGG
ACAGGAGTGCAAGCCTTAATAAGAATGCTGCAACAACTGCTGTTTATTCATTTCAGAATTGGGTGTCGACACA
GCAGAATAGGCATTCAACACAGGAGGCCAAGAAATGGAGCCAGTAGATCCTAGTCTAGAACCCTGGAAGCATC
CAGGAAGTCAGCCTAGGACTGCTTGTAACACTTGCTATTGTAAAAAGTGTTGCTTTCATTGCCAAGTTTGTTT
CATAAAAAAAGCTCTAGGCATCTCCTATGGCAGGAAGAAGCGGAGACAGCGACGAAGAACTCCTCAAAACAGT
GAGACTCATCAAGCTTCTCTATCAAAGCAGTAAGTAGTTCATGTAATGCAAGCTTTAGTGATATCAGCAATAG
TAGGATTAGTAGTAGCAGCTATACTAGCAATAATTGTATGGACCTTAGTACTGATAGAATATAGGAAAATATT
AAGACAAAGGAAAATAGACAGGTTAATTGATAGAATAAGAGAAAGAGCAGAAGATAGTGGCAATGAGAGTGAA
GGGGATCAGGAGGAATTGTCAGCACTTGTGGAAATGGGGCACGATGCTCCTTGGGATATTGATGATCTGTAGT
GCTTCAGAACAATTGTGGGTCACAGTCTATTATGGGGTACCTGTGTGGAAAGAAGCAAACACCACTCTATTTT
GTGCATCAGATGCTAAAGCATATGCTACAGAGGTGCATAATGTTTGGGCCACACATGCATGTGTACCCACAGA
CCCTAACCCACAAGAAGTAACATTGGAAAATGTAACAGAAAAGTTTGACATGTGGAATAATAACATGGTAGAA
CAGATGCATGAGGATGTAATCAGTTTATGGGACCAAAGCCTAAAGCCATGTGTAAAACTAACTCCACTCTGTG
TTACTTTAAATTGCACTAATGTAAATGTTAATGGTTCCACTAGGGTTGCTCCTACCAATAGTGGTATCAGGAA
TATAACGGAAGAAATGAAAAACTGCACTTTCAATATCACCACAAGCTTAAGAGATAAGATGCAAAGGGTATAT
TCACTTTTTTATAAAGAGGATATAGAACCAATAGAGAATAGTACTGATAGTCTTAGTGCCAGCTATAGGTTGA
CAAAGTGTAACACCTCAATCATTACACAAGCCTGTCCAAAGGTATCCTTTGAGCCAATTCCCATACATTATTG
TGCCCCGGCTGGTTTTGCAATTATAAAATGTAAGGATAAGAACTTCACTGGAACAGGACTATGTAAAAATGTC
AGCACAGTACAGTGTACACATGGAATTAGGCCAGTAGTATCAACTCAACTATTGCTAAATGGTAGTCTAGCAG
AAGAAGAGATAATAATTAGAACTAAAAACCTTACAGATAATACTAAAAACATAATAGTACAGCTGAATAAAAC
TGTAGAAATTCGCTGTATAAGACCCGGCAACAACACAAGAAAAAGCCTACACATGGGACCAAGGAGAGCATTT
TATGCATTAAATGGTATAATAGGGGACATAAGACAAGCACATTGTAACATTAGTAAAAACGAATGGTATGAAA
CTTTAGAAAAGGTAGCTAGCAAAATAAGAGAACAATTTAGTAAAATAAACAGAAGTTATGAGAATGATACAAT
AAGCTTTAGGCCACCATTCCCAGGAGGGGACCCAGAAATTATAATGCACAGTTTTAATTGTGGAGGAGAATTT
TTCTACTGTAATACAACAGAACTGTTTAATAGCGATTGGAATACGAGTATCGCTTGGAATAGCACCAAGAATG
GCACCAACATCACACTCCCATGCAGAATAAGACAAATTGTAAACATGTGGCAGGAAGTAGGAAAAGCAATGTA
TGCTCCTCCCATCAGAGGAAACATCACATGTTTATCAAATATTACAGGGCTGCTATTAACATGGGATGGTGGG
AATGGGAATGAGACCAACAAGCAGACCTTCAGACCTATAGGAGGAGATATGAGGGACAATTGGAGAAGTGAAT
TATATAAATACAAAGTAGTAAAAATTGAACCATTAGGAATAGCACCCACCAAGGCAAAAAGAAGAGTGGTGCA
GAGAGAAAAAAGAGCAGTGGGATTATTAGGAGCTGTGTTCCTTGGGTTCTTGGGAGCAGCAGGAAGCACTATG
GGCGCAGCGTCAATAACGCTGACGGCACAAGCCAGACAGTTAATGTCTGGTATAGTGCAACAGCAGAACAATC
TGCTGAGAGCTATTGAGGCGCAACAGCATATGTTGCAACTCACAGTCTGGGGCATCAAGCAGCTCCAGGCAAG
AGTCCTGGCTCTGGAAAGATACCTAGCGGATCAACAGCTCCTAGGACTTTGGGGTTGCTCTGGAAAACTCATC
TGCCCCACTGCTGTGCCTTGGAATGTTAGTTGGAGTAATAAATCTCTGGGAGACATTTGGGATAACATGACCT
GGATGCAGTGGGAAAGAGAAATTAATAATTATACAGATATAATATACTCCTTACTTGAAGGAGCGCAGACCCA
GCAAGAGAAGAATGAAGAAGACTTATTAAAATTAGATCAATGGGCAAATTTGTGGAATTGGTTTGACATAACC
CAATGGCTGTGGTATATAAGAATATTCATAATGGTAGTAGGAGGCTTGATAGGTTTAAGAATAGTTTTTGCTG
TACTTTCTATAGTGAATAGAGTTAGGCAGGGATACTCACCCTTGTCGTTACAGATCCCCCTCCCAGTCCAGAG

GGAACCCGACAAGCCCGAAGGAATA?AAGAAGAAGGTGGAGAGAGAGACAGAGACAGATCCGCAAGATTAGTG
ACCGGATTCTTGCCACTTATCTGGGACGATCTGAGGAGCCTGTGCCTCTTCAGCTACCACCGCTTGAGAGACT
TACTCTTGATTGTAACGAGGATTGTGGAACTTCTGGGACGCAGGGGGTGGGAAATCCTCAAATATTGGTGGAA
TCTCCTGACCTATTGGAGCCAGGAACTAAAGAATAGTGCTGTTAGCTTGCTTAACGTCACAGCCATAACAGTA
GCTGAGGGAACAGACAGGGTTATAGAAGCATTACTAAGAGCTTGGAGGGCTATTCTCCACATACCTAGAAGAA
TAAGACAGGGCTTAGAAAGGGCTTTGCTATAAGATGGGTGGCAAATGGTCCAAACGTAGTGGGGGTGGATGGT
CTACTATAAGGGAAAGAATGAGACAAACTCCACCTGAACCTGAACCAGCAGCAGAGGGGGTGGGAGCAGTATC
TCGAGACCTAGAAAGACATGGAGCAATCACAAGTAGCAATACAGCAGCTACCAATGCTGCTTGTGCCTGGCTA
GAAGCACAAGAGGAGGAGGAGGTGGGCTTTCCAGTCAGACCCCAGGTACCTTTAAGACCAATGACTTACAAGG
GAGCTTTGGATCTTAGCCACTTTTTAAAAGAAAAGGGGGGACTGGAAGGGCTAATTTACTCCCAAAAGAGACA
AGATATTCTTGATCTGTGGGTCTATCACACACAAGGCTACTTCCCTGATTGGCAGTGCTACACACCAGGGCCA
GGGACTAGATTCCCATTGACCTTTGGGTGGTGCTTCAAGCTAGTACCAGTCGAGCCAGAGAAGATAGAAGAGG
CCACTGAAGGAGAGAACAACATACTGTTACACCCTATGAATCTGCATGGGATGGATGACCCAGAGAGAGAAGT
GCTACAGTGGAAGTTTGACAGTCACCTAGCATTCCGTCACATGGCCAAAGAGCTGCATCCGGAGTACTTCAAG
AACTGAACTGCTGACATCGAGCTTTCTACAAGGGACTTTCCGCTGGGGACTTTCCAGGGGAGGCGTGGCCTGG
GCGGGACTGGGGAGTGGCGAGCCCTCAGATGCTGCATATAAGCAGCCGCTTTTCGCTTGTACTGGGTCTCTCT
GGTTAGACCAGATCTGAGCCTGGGAGCTCTCTGGCTAGCTAGGGAACCCACTGCTTAAGCCTCAATAAAGCTT
GCCT

>S-3B4 HIV-1 genome, derived from RNA genomic sequence
GCAGTGGCGCCCGAACAGGGACTTGAAAGCGAAAGAGAAACCGGAGGAGCTCTCTCGACGCAGGACTCGGCTT
GCTGAAGCGCGCACGGCAAGAGGCGAGGGGCGGCGACTGGTGAGTACGCCAAAAATTTTGACTAGCAGAGGCT
AGAAGGAGAGAGATGGGTGCGAGAGCGTCAATATTAAGCGGGGGAGAATTAGATAGATGGGAAAAAATTCGGT
TAAGGCCAGGAGGAAAGAAAAAATATAGACTAAAACATTTAGTATGGGCAAGCAGGGAGCTAGAACGATTCGC
AGTCAACCCTGGCCTGTTAGAAACATCAGAAGGTTGTAGACAAATACTGGGACAGCTACATCCGTCCCTCCAG
ACAGGATCAGAGGAGCTTAAATCATTATATAATACAATAGCAGTTCTCTATTGTGTGCATCAAAGGATAGAGG
TAAAAGACACCAAGGAAGCTTTAGAGAAGATAGAGGAAGAGCAAAACAAATGTAAGAAAAAAGCACAGCAAGC
GGCAGCTGCTGACACAGGAACTAGCAGCAACAGCCCGGTCAGCCAAAATTACCCTATAGTGCAGAACATTCAG
GGGCAAATGGTACATCAGCCCATATCACCTAGAACTTTAAATGCGTGGGTAAAAGTAGTAGAAGAAAAGGCTT
TCAGCCCAGAAGTAATACCCATGTTTTCAGCATTATCAGAAGGAGCCACCCCACAAGATTTAAACACCATGTT
AAACACAGTGGGGGGACATCAGGCAGCCATGCAAATGTTAAAAGAGACCATCAATGAGGAAGCTGCAGAATGG
GATAGATTACACCCAGTGCATGCAGGGCCTGTTGCACCAGGCCAGTTTAGAGAACCAAGGGGAAGTGACATAG
CAGGAACTACTAGTACCCTCCAGGAACAAATAGCATGGATGACACATAATCCACCTATCCCAGTAGGAGACAT
CTATAAAAAATGGATAATCATGGGACTAAATAAAATAGTGAGGATGTATAGCCCTACCAGCATTCTGGACATA
AAACAAGGACCAAAAGAACCCTTTAGAGATTATGTGGACCGGTTCTATAAAGTTTTAAGAGCCGAGCAAGCTA
CACAGGAGGTAAAAAATTGGATGACGGAAACCTTGTTGGTCCAAAATGCGAACCCAGATTGTAAGACTATTCT
AAAAGCATTAGGGCCAGCAGCTTCACTAGAAGAAATGATGACAGCATGTCAGGGAGTGGGAGGACCCAGCCAT
AAAGCAAGAGTTTTGGCTGAAGCAATGAGCCAAGCAACAAATTCAGCTGTTGTGATGATGCAGAGGGGCAATG
TTAGGAACCAAAGAAAAGTTATTAAGTGTTTCAATTGTGGCAAAGAGGGGCACATAGCCAAAAATTGCAGAGC
CCCTAGGAAAAAGGGCTGTTGGAAATGTGGAAAGGAAGGACACCAAATGAAAGATTGTACTGAGAGACAGGCC
AATTTTTTAGGGAAAATCTGGCCTTCCCACAAGGGGAGGCCAGGGAATTTTCTTCAGAACAGACCAGAGAGCA
GACTAGAGCCAACAGCCCCACCAGAGGAGAGCTTCAGGTTTGGGGAAGAGACAGCGGCTCCCTCTCAGAAGCA
GGAGCCGATAGACAAGGAACTGTATCCTTTAGCTTCCCTCAAATCACTCTTTGGCAACGACCCCTCGTCACAG
TAAAGATAGGGGGGCAATTAAAGGAAGCTCTACTAGATACAGGAGCAGATGATACAGTACTAGAAGACATGGA
TTTGCCAGGAAGATGGAAGCCAAAAATGATAGGGGGAATTGGAGGTTTTATCAAAGTAAGACAATATGATCAG
ATACCTGTAGAAATCTGTGGACATAAAGCTATAGGTACAGTATTAGTAGGACCTACACCTGTCAACATAATTG
GAAGAAATCTGTTGACTAAGATTGGTTGCACTTTAAATTTTCCCATAAGTCCTATTGAAACTGTACCAGTAAA
ATTAAAGCCAGGAATGGATGGCCCAAAAGTTAAACAATGGCCATTGACAGAAGAGAAAATAAAAGCATTGATA
GAAATTTGTACAGAAATGGAAAAGGAAGGAAAAATTTCAAAAATTGGGCCTGAAAATCCATACAATACTCCAG
TATTTGCCATAAAGAAAAAAGATAGTACTAAGTGGAGAAAATTAGTAGACTTCAGAGAACTTAATAAGAAAAC
TCAAGACTTCTGGGAAGTTCAATTAGGAATACCACATCCCGCAGGGTTAAAAAAGAGAAGATCAGTAACAGTA
CTGGATGTGGGTGATGCATATTTTTCAGTTCCCTTAGATGAAAATTTCAGGAAATATACTGCATTTACCATAC
CTAGTACAAATAATGAGACACCAGGGATTAGGTATCAATACAATGTGCTTCCACAGGGATGGAAAGGATCACC
AGCAATATTCCAAAGTAGCATGACAAAGATCTTAGAGCCTTTCAGAAAACAAAATCCAGACATAATTATCTAC
CAATACATGGATGATTTGTATGTAGGATCTGATTTAGAAATAGAACAGCATAGAACAAAAATAGAGGAATTGA
GACAGCATTTGTTGAGGTGGGGACTTACCACACCAGACAAAAAGCATCAGAAAGAACCTCCATTCCTTTGGAT
GGGTTATGAACTCCATCCTGACAAATGGACAGTACAGCCTATAGTGCTGCCAGAAAAGGACAGCTGGACTGTC
AATGACATACAGAAGTTAGTGGGAAAATTAAATTGGGCAAGTCAGATTTATGCAGGGATTAAAGTAAAGCAAT
TATGTAAACTCCTTAGGGGAGCCAAAGCATTGACAGAAGTAATACCACTAACAGAGGAAGCAGAACTAGAACT
GGCAGAAAACAGGGAGATTCTAAAAGAACCAGTACATGGAGTGTATTATGACCCATCAAAAGACTTAATAGCA
GAAGTACAGAAGCAGGGGCTAGGCCAATGGACATATCAAATTTATCAAGAGCCATTTAAAAATCTGAAAACAG
GAAAATATGCAAGAATGAGGGGTGCCCACACTAATGATGTAAAACAGTTAACAGAGGCAGTGCAAAAGATAGC
TACAGAAAGCATAGTAATATGGGGAAAGACTCCTAAATTTAAGCTCCCCATACAAAAAGAAACATGGGAAACA
TGGTGGACAGAGTATTGGCAAGCCACCTGGATTCCTGAATGGGAGTTTGTCAATACCCCTCCCTTAGTGAAAC
TATGGTACCAGTTAGAGAAAGAGCCTATAGAAGGAGCAGAAACTTTCTATGTAGATGGGGCAGCTAACAGGGA
GACTAGATTAGGAAAGGCAGGATATGTTACTAACAGAGGAAGACAAAAGGTTGTCTCCCTAACTGACACAACA
AATCAGAAAACTGAGTTACAGGCAATTTATCTGGCTTTGCAGGATTCGGGATTAGAAGTGAACATAGTAACAG
ACTCACAATATGCATTAGGAATCATTCAAGCACAACCAGATAAAAGTGAATCAGAGTTAGTCAGTCAAATAAT
AGAGCAGTTAATAAAAAAGGAAAAGGTCTACTTGGCATGGGTACCCGCACACAAAGGAATTGGAGGAAATGAG
CAAGTAGATAAGCTAGTCAGTGCCGGAATCAGGAAGGTGCTGTTTTTAGATGGAATAGATAAGGCACAAGCGG
ACCATGAAAAATATCACAGTAATTGGAGAGCAATGGCTAGTGAGTTTAACCTGCCACCTGTAGTAGCAAAAGA
AATAGTAGCCTGCTGTGATAAATGTCAACTAAAAGGAGAAGCCATGCATGGGCAAGTAGACTGTAGTCCAGGA
ATATGGCAGCTAGATTGTACACATTTAGAAGGAAAAATTATCCTGGTGGCAGTGCATGTAGCTAGTGGATATA

TAGAAGCAGAAGTTATTCCAGCAGAGACAGGGCAGGAAACAGCTTACTTTCTGTTAAAATTAGCAGGAAGATG
GCCAGTAAAAATGATACATACAGACAATGGCACCAATTTCACCAGTACTGCAATGAAGGCCGCCTGTTGGTGG
GCAGGGATCAAGCAGGAATTTGGCATTCCCTACAATCCCCAAAGTCAAGGAGTAGTAGAATCTATGAATAAAG
AATTAAAGAAAATTATAGGACAGGTAAGAGATCAGGCTGAACATCTTAAGACAGCAGTACAAATGGCAGTATT
CATCCACAATTTTAAAAGAAAAGGGGGGATTGGGGAGTACAGTGCAGGGGAAAGAATAATAGACATAATAGCA
ACAGACATACAAACTAAAGAGTTACAAAAACAAATTACAAAAATTCAAAATTTTCGGGTTTATTACAGGGACA
GCAGAGACCCACTTTGGAAAGGACCAGCAAAGCTTCTCTGGAAAGGTGAAGGGGCAGTAGTAATACAAGATAA
TAGTGACATAAAAGTAGTACCAAGAAGAAAAGCAAAGATCATTAGGGATTATGGAAAACAGATGGCAGGTGAT
GATTGTGTGGCAGGTAGACAGGATGAGGATTAAAACATGGAAAAGTTTAGTAAAACACCATATGTATATCTCA
AAGAAAGCTAAGGGATGGGTTTATAGACATCACTATGAAAGCACTCATCCAAGAATAAGTTCAGAAGTATACA
TCCCACTAGGGGATGCTAAATTAGTAATAACAACATATTGGGGTCTGCATACAGGAGAAAGAGATTGGCATTT
GGGTCAGGGAGTCTCCATAGAATGGAGGAAGGAGAGATATAGCACACAGGTAGACCCTGGCCTAGCAGACCAA
CTAATTCATATGTATTACTTTGATTGTTTTTCAGAATCTGCTATAAGACATACCATATTAGGACATAGAATTA
GCCCTAGTTGTGAATATCAAGCAGGACATAACAAGGTAGGATCTCTACAGTACTTGGCACTAACAGCATTAAT
AACACCAAGAAAAACAAGGCCACCTTTGCCTAGTGTTACGAAACTGACAGAGGATAGATGGAACAAGCCCCGG
AAGACCAAGGGCCACAGAGGGAGCCATACAATGAATGGACACTAGAACTTTTAGAGGAGCTTAAGAGTGAAGC
TGTTAAACACTTTCCTAGGGCATGGCTCCATAGCTTAGGGCAATATATCTATGAAACTTATGGGGATACTTGG
ACAGGAGTGCAAGCCTTAATAAGAATGCTGCAACAACTGCTGTTTATTCATTTCAGAATTGGGTGTCGACACA
GCAGAATAGGCATTCAACACAGGAGGCCAAGAAATGGAGCCAGTAGATCCTAGCCTAGAACCCTGGAAGCATC
CAGGAAGTCAGCCTAGGACTGCTTGTAACACTTGCTATTGTAAAAAGTGTTGCTTTCATTGCCAAGTTTGTTT
CATAAAAAAAGCTCTAGGCATCTCCTATGGCAGGAAGAAGCGGAGACAGCGACGAAGAACTCCTCAAAACAGT
GAGACTCATCAAGCTTCTCTATCAAAGCAGTAAGTAGTACATGTAATGCAAGCTTTAGTGATATCAGCAATAG
TAGGATTAGTAGTAGCAGCAATACTAGCAATAATTGTGTGGACCTTAGTACTAATAGAATATAGGAAAATATT
AAGACAAAGGAAAATAGACAGGTTAATTGATAGAATAAGAGAAAGAGCAGAGGATAGTGGCAATGAGAGTGAA
GGGGATCAGGAGGAATTGTCAGCACTTGTGGAAATGGGGCACGATGCTCCTTGGGATATTGATGATCTGTAGT
GCTTCAGAACAATTGTGGGTCACAGTCTATTATGGGGTACCTGTGTGGAAAGAAGCAAACACCACTCTATTTT
GTGCATCAGATGCTAAAGCATATGCTACAGAGGTGCATAATGTTTGGGCCACACATGCATGTGTACCCACAGA
CCCTAACCCACAAGAAGTAACATTGAAAAATGTAACAGAAAAATTTGACATGTGGAATAATAACATGGTAGAA
CAGATGCATGAGGATGTAATCAGTTTATGGGACCAAAGCCTAAAGCCATGTGTAAAACTAACTCCACTCTGTG
TCACTTTAAATTGCACTAATGTGAATGTTACTGGTTCCACTAAGGTTAATTATACCAATAGTAGTATCAGGAA
TATCACGGAAGAAATGAAAAACTGCACTTTCGATATCACCACAAGTTTAAGAGATAAGATGCAAAGGGTATAT
TCACTTTTTTATAAAGAGGATATAGAACTAATAGAGAATAGTACTGATAGTCTTAGTGCCAGCTATAGGTTGA
CAAAGTGTAACACCTCAGTCATTACACAAGCCTGTCCAAAGGTATCCTTTGAGCCGATTCCCATACATTATTG
TGCCCCGGCTGGTTTTGCAATTATAAAATGTAAGGATAAGAACTTCACTGGAACAGGACTATGTACAAATGTC
AGCACAGTACAGTGTACACATGGAATTAGGCCAGTAGTATCAACTCAACTATTGTTAAATGGTAGTCTAGCAG
AAGAAGAGATAATAATTAGAACTAAAAACCTTACAGATAATACTAAAAACATAATAGTACAGCTGAATAAAAC
TGTGACAATTCTCTGTATAAGACCCGGCAACAATACAAGGAAAAGCCTACCTATAGGACCAGGGAGAGCATTT
TATGCAACAAATGATATAATAGGAGACATAAGACAAGCACATTGTAACATTAGTGAAGACGAATGGTATAAAA
CTTTAGCAAAGGTAGCTAGCAAAATAAGAGAACAATTTAGTAAAATAAACAGAAGTCATGAGAATGATACAAT
AGTCTTTAATCCACCATCCCCAGGAGGGGACCCAGAAATTACAATGCACAGTTTTAATTGTGGAGGGGAATTT
TTCTACTGTAATACAACACAACTGTTTAATAGCACTTGGGATAGGAATAGCACTGGGGATAAGAATAACACCA
ATAGCACCATCACACTCCCATGCAGAATAAAACAAATTGTAAACATGTGGCAGGAAGTAGGCAAAGCAATGTA
TGCTCCTCCCATCAGAGGAAACATCACATGTTCATCAAATATTACAGGGCTGCTATTAACAAGGGATGGTGGG
AATGGGAATGAGACCAACAAGCAGACCTTCAGACCTATAGGAGGAGATATGAGGGACAATTGGAGAAGTGAAT
TATATAAATACAAAGTAGTAAAAATTGAACCATTAGGAATAGCACCCACCAAGGCAAAAAGAAGAGTGGTGCA
GAGAGAAAAAAGAGCAGTGGGATTAGGAGCTGTGTTCCTTGGGTTCTTGGGAGCAGCAGGAAGCACTATGGGC
GCAGCGTCAATAACGCTGACGGCACAAGCCAGACAGTTAATGTCTGGTATAGTGCAACAGCAGAACAATCTGC
TGAGAGCTATTGAGGCGCAACAGCATATGTTGCAACTCACAGTCTGGGGCATCAAGCAGCTCCAGGCAAGAGT
CCTGGCTCTGGAAAGATACCTAGCGGATCAACAGCTCCTGGGACTTTGGGGTTGCTCTGGAAAACTCATCTGC
ACCACTGCTGTGCCTTGGAATGTTAGTTGGAGTAATAAATCTCTGGGACAAATTTGGAATAACATGACCTGGA
TGCAGTGGGAAAGAGAAATTAATAACTATACAGATATAATATACTCCTTACTTGAAGGAGCGCAGACCCAGCA
AGAGAAGAATGAAGAAGACTTACTAAAATTAGATCAATGGGCAAATTTGTGGAATTGGTTTGACATAACCCAA
TGGCTGTGGTATATAAGAATATTCATAATGGTAGTAGGAGGCTTAATAGGTTTAAGAATAGTTTTTGCTGTAC
TTTCTATAGTGAATAGAGTTAGGCAGGGATACTCACCCTTGTCGTTACAGATCCCCCTCCCAATCCCGAGGGA

ACCCGACAAGCCCGAAGGAATCGAAGAAGAAGGTGGAGAGAGAGGCAGAGACAGATCCGCAAGATTAGTGACC
GGATTCTTGCCACTTATCTGGGACGATCTGAGGAGCCTGTGCCTCTTCAGCTACCACCGCTTGAGAGACTTAC
TCTTGATTGTAACGAGGATTGTGGAACTTCTGGGACGCAGGGGGTGGGAAATCCTCAAATATTGGTGGAATCT
CCTGACCTACTGGGGCCAGGAACTAAAGAATAGTGCTGTTAGCTTGCTTAACGCCACAGCCATAACAGTAGCC
GAGGGAACAGACAGGGTTATAGAAGCATTACTAAGAGCTTGGAGGGCTATTCTCAACATACCTAGAAGAATAA
GACAGGGCTTAGAAAGGGCTTTGCTATAAAATGGGGGGCAAGTGGTCAAAACGTAGTGGGGGTGGATGGTCTA
CGATAAGGGAAAGAATGAGACAAACTCCACCTGAACCTGAACCAGCAGCAGAGGGGGTGGGAGCAGTATCTCG
AGACCTAGAAAAACATGGAGCAATCACAAGTAGCAATACAGCAGCTACCAATGCTGCTTGTGCCTGGCTAGAA
GCACAAGAGGAGGAGGAGGAGGTGGGCTTTCCAGTCAGACCCCAGGTACCTTTAAGACCAATGACTTACAAGG
GAGCTTTGGATCTTAGCCACTTTTTAAAAGAAAAGGGGGGACTGGAAGGGCTAATTTACTCCCAAAAGAGACA
AGATATTCTTGATCTGTGGGTCTATCACACACAAGGCTACTTCCCTGATTGGCAGTGTTACACACCAGGGCCA
GGGACTAGATTCCCATTGACCTTTGGGTGGTGCTTCAAGCTAGTACCAGTCGAGCCAGAGAAGATAGAAGAGG
CCACCGAAGGAGAGAACAACATACTGTTACACCCTATGAATCTGCATGGGATGGATGACCCAGAGAGAGAAGT
GTTACAGTGGAAGTTTGACAGTCACCTAGCGTTCCGTCACGTGGCCAAAGAGCTGCATCCGGAGTACTTCAAG
AACTGAACTGCTGACACTGAGCACTGAACTGCTGACATCGAGCTTTCTACAAGGGACTTTCCGCTGGGGACTC
TCCAGGGGAGGAGTGGCCTGGGCGGGACTGGGGAGTGGCGAGCCCTCAGATGCTGCATATAAGCAGCCGCTTT
TCGCTTGTACTGGGTCTCTCTGGTTAGACCAGATCTGAGCCTGGGAGCTCTCTGGCTAACTAGGGAACCCACT
GCTTAAGCCTCAATAAAGCTTGCCTCTG

>T-13A HIV-1 genome, derived from RNA genomic sequence
GTGGCGCCCGAACAGGGACTAGAAAGCGAAAGTAAGACTAGAGGAGCTCTCTCGACGCAGGACTCGGCTTGCT
GAAGCGCGCACGGCAAGAGGCGAGGGGCGGCGACTGGTGAGTACGCCAAAAATTAAAATTTTGACTAGCGGAG
GCTAGAAGGAGAGAGATGGGTGCGAGAGCGTCAGTATTAAGCGGGGGAGAATTGGATAGATGGGAAAAAATTC
GGTTACGGCCAGGGGGAAAGAAAAAGTATAAATTGAAACATATAGTATGGGCAAGCAGGGAGCTAGAACGATT
CGCAGTCAACCCTGGCCTTTTAGAGACAGCAGGAGGCTGTAGACAAATATTGGAACAGCTACAACCATCCCTT
CAGACAGGATCAGAAGAACTTAAATCATTATATAATACAATAGCAACCCTCTATTGTGTGCATCAAAAGATAG
ATGTAAAAGACACCAAGGAAGCTTTAGACAAAATAGAGGAAGAGCAAAACAAAAGTAAGAAAAAGGCACAGCA
AGCAGCAGCAGCAGCTGACACAGGAAGCAGCAGCAAGGTCAGCCAAAATTATCCTATAGTACAAAATCTACAG
GGGCAAATGGTACATCAGCCCATATCACCTAGAACTTTAAATGCATGGGTAAAAGTAGTAGAAGAGAAGGCTT
TCAGTCCAGAGGTAATACCCATGTTTTCAGCATTATCAGAAGGAGCCACCCCACAAGATTTAAACACCATGCT
AAATACAGTGGGGGGACATCAAGCAGCCATGCAAATGTTAAAAGAGACCATCAATGAGGAAGCTGCAGAATGG
GATAGATTACACCCAGTGCATGCAGGGCCTATTGCACCAGGCCAGATGAGAGAACCAAGGGGAAGTGACATAG
CAGGAACTACTAGTACCCTTCAAGAACAAATAGGATGGATGACAAATAATCCACCTATCCCAGTAGGAGAAAT
CTATAAGAGATGGATAATCCTGGGATTAAATAAAATAGTAAGAATGTATAGCCCTACCAGCATTTTGGACATA
AGACAAGGACCAAAAGAACCCTTTAGAGATTATGTAGACAGGTTCTATAAAACGCTAAGAGCAGAGCAAGCCT
CACAGGAGGTAAAAAATTGGATGACAGAAACCTTGTTGGTCCAAAATGCGAACCCAGATTGTAAGACTATCTT
AAAAGCATTAGGACCAGCAGCTACACTAGAAGAAATGATGACAGCATGTCAGGGAGTGGGGGGACCCGGCCAT
AAAGCAAGAGTTTTGGCTGAAGCAATGAGCCAAGTAACAAATTCAGCTACCATAATGATGCAGAAAGGCAATT
TTAGGAGCCAAAGAAAGACTGTTAAGTGTTTCAATTGTGGCAAAGAGGGGCACATAGCCAAAAATTGCAGGGC
TCCTAGGAAAAAGGGCTGTTGGAAATGTGGAAGGGAAGGACACCAAATGAAAGATTGTACTGAGAGACAGGCT
AATTTTTTAGGGAAAATCTGGTCTTCCCACAAGGGAAGGCCAGGGAATTTTCTTCAGAGCAGACCAGAGCCAA
CAGCCCCACCAGCAGAGAGCTTCAGGTTTGGGGAAGAGACAGCAATTCCCCCTCAGAAGCAGGAGCCGATAGA
CAAGGAGCTATATCCTTTAGCTTCCCTCAGATCACTCTTTGGCAACGACCCCTCGTCACAATAAGGATAGGGG
GGCAACTAAAAGAAGCTCTATTAGATACAGGAGCAGATGATACAGTATTAGAAGACATGAGTTTGCCAGGAAG
ATGGAAACCAAAAATGATAGGGGGAATTGGAGGTTTTATCAAAGTAAGACAGTATGATCAAATACCAGTAGAA
ATCTGTGGACATAAAGCTGTAGGTACAGTATTAGTAGGACCCACACCTGTCAACATAATTGGAAGAAATCTGT
TGACTCAGATTGGCTGCACTTTAAATTTTCCCATTAGTCCTATTGAAACTGTACCAGTAAAATTAAAGCCAGG
AATGGATGGCCCAAAAGTTAAACAATGGCCATTGACAGAAGAGAAAATAAAAGCATTAGTAGAAATTTGTACA
GAAATGGAAAAGGAAGGGAAAATTTCAAAAATTGGGCCTGAAAATCCATATAATACTCCAGTATTTGCCATAA
AGAAAAAAGACAGTACTAAATGGAGAAAATTAGTAGACTTCAGGGAACTTAATAAGAGAACTCAAGACTTCTG
GGAAGTTCAATTAGGAATACCACATCCAGCAGGGTTAAAAAAGAAAAAATCAGTAACCGTACTGGATGTGGGT
GATGCATATTTCTCAGTTCCCTTAGATAAAGACTTCAGGAAGTATACTGCATTTACCATACCTAGTATAAACA
ATGAGACACCAGGAATTAGATATCAGTACAATGTGCTTCCACAAGGATGGAAAGGATCACCAGCAATATTCCA
AAGTAGCATGACAAAAATTTTAGAGCCTTTTAGAAAACAAAATCCAGACATAGTTATCTATCAATACATGGAT
GATTTGTATGTAGGATCTGACTTAGAAATAGGGCAACATAGAGCAAAAATAGAGGAACTGAGACAACATCTGT
TGAGGTGGGGATTTACCACACCAGACAAAAAGCATCAGAAAGAACCTCCATTCCTTTGGATGGGTTATGAACT
CCATCCTGATAAATGGACAGTACAGCCTATAGTGCTGCCAGAAAAAGACAGCTGGACTGTCAATGACATACAG
AAGTTAGTGGGAAAATTAAATTGGGCAAGTCAAATTTATGCAGGGATCAAAGTAAAGCAATTATGTAAACTCC
TTAGGGGAACCAAATCACTAACAGAAGTAGTACAACTGACAGAAGAAGCAGAGCTCGAACTGGCAGAAAACAG
GGAGATTCTAAAAGAACCAGTACATGGAGTGTATTATGACCCATCAAAAGACTTAATAGCAGAAATACAGAAG
CAGGGAAATGGCCAATGGACATATCAAATTTATCAAGAGCCATATAAAAATCTGAAAACAGGAAAGTATGCAA
GAATGAGGGGTGCCCACACTAATGATGTAAAACAATTAACAGAGGCAGTGCAAAAAATAGCCAATGAAAGCAT
AGTAATATGGGGAAAGATTCCTAAATTTAAATTACCCATACAGAAAGAAACATGGGAAGCATGGTGGATGGAG
TATTGGCAAGCCACCTGGATTCCTGAGTGGGAGTTTGTCAATACCCCTCCCTTAGTGAAATTATGGTATCAGT
TAGAGAAAGAGCCCATAGTAGGAGCAGAAACTTTCTATGTAGATGGGGCAGCTAATAGGGAAACTAAATTGGG
AAAAGCAGGATATGTTACTGACAGAGGAAGACAAAAAGTTGTCTCCCTAACAGACACAACAAATCAGAAGACT
GAGTTACAAGCAATTTATCTAGCTTTGCAGGATTCGGGATTAGAAGTAAACATAGTAACAGACTCACAATATG
CATTAGGAATCATTCAAGCACAACCAGATAAGAGTGAATCAGAGTTAGTTAATCAAATAATAGAGCAGTTAAT
AAAAAAGGAAAAGGTTTACCTGGCATGGGTACCAGCACACAAAGGAATTGGAGGAAATGAACAAGTAGATAAA
TTAGTCAGTGCTGGAATTAGGAAAGTACTATTTTTAGATGGAATAGATAAGGCCCAAGATGAACATGAGAAAT
ATCACAATAATTGGAGAGCAATGGCTAGTGATTTTAACCTGCCACCTATAGTAGCAAAAGAAATAGTAGCCAG
CTGTGATAAATGTCAGCTAAAAGGAGAAGCCATGCATGGACAAGTAGACTGTAGTCCAGGAATATGGCAGCTA
GATTGTACACACTTAGAAGGAAAAGTTATCCTGGTAGCAGTTCATGTGGCCAGTGGATATATAGAAGCAGAAG

TTATTCCAGCAGAAACAGGGCAAGAAACAGCATACTTTATCTTAAAATTAGCAGGAAGATGGCCAGTAAAAAT
TATACACACAGACAATGGCCCCAATTTCATCAGTACTGCGGTTAAGGCTGCCTGTTGGTGGGCAGGGATCAAG
CAGGAATTTGGTATTCCCTACAATCCCCAAAGTCAAGGAGTAGTAGAATCTATGAATAATGAATTAAAGAAAA
TTATAGGACAGGTAAGAGATCAAGCTGAACATCTTAAGACAGCAGTACAAATGGCAGTATTCATCCACAATTT
TAAAAGAAAAGGGGGGATTGGGGGGTACAGTGCAGGGGAAAGAATAGTAGACATAATATCATCAGACATACAA
ACTAAAGAATTACAAAAACAGATTACAAAAATTTCAAAATTTCGGGTTTATTACAGGGACAGCAGAGATCCAC
TTTGGAAAGGACCAGCAAAGCTTCTCTGGAAAGGTGAAGGGGCAGTAGTAATACAAGATAATAGTGACATAAA
AGTAGTGCCAAGAAGAAAAGCAAAGATCATTAGGGATTATGGAAAACAGATGGCAGGTGATGATTGTGTGGCA
AGTAGACAGGATGAGGATTAGATCATGGAAAAGTTTAGTAAAACATCATATGTATATTTCAGGAAAAGCTAGG
AAATGGTTTTATAGACATCACTATGAAAGCACTCATCCAAGAATAAGTTCAGAAGTTCACATCCCACTAGGGG
ATGCTAAATTAGTAGTAACAACATATTGGGGTCTGCATACAGGAGAAAGAGACTGGCATTTGGGTCAAGGAGT
CTCCATAGAATGGAGGAAAAGGAGATATAGCACACAAGTAGACCCTGACCTAGCAGACCAATTAATTCATCTG
TATTATTTTGATTGTTTTTCAGAAGCTGCTATAAGAAATGCCATATTAGGACATATAGTTAGACCTAGGTGTG
AGTATCAAGCAGGACATAACAAGGTAGGATCTCTACAGTACTTGGCACTAACAGCATTAATAACACCAAAAAA
GATAAAGCCACCTTTGCCTAGTGTTAAGAAACTGACAGAGGATAGATGGAACAAGCCCCAGAGAACCAAGGGC
CACAGAGGGAGCCATACAATGAATGGACACTAGAGATTTTAGAGGAACTGAAAAATGAAGCTGTTAGACATTT
TCCTAGGGCATGGCTCCATAGCTTAGGACAATATATCTATGAAACTTATGGGGATACTTGGGCAGGAGTGGAA
GCCTTATTAAGAATTCTGCAACAAATGCTGTTTATTCATTTCAGAATTGGATGTCACCATAGCAGAATAGGCA
TTATTCTACAGAGGAGAACAAGAAATGGAGCCAGTAGATCCTAGACTAGAGCCCTGGAAGCATCCAGGAAGTC
AGCCTAAAACTGCTTGTACCAAATGCTATTGTAAAAAGTGTTGCTTGCATTGCCAAGTTTGCTTCATAACAAA
AGGCTTAGGCATCTCCTATGGCAGGAAGAAGCGGAGACAGCGACGAAGACCTCCTCAAAACAGTGAGGCTCAT
CAAGTTTCTCTATCAAAGCAGTAAGTAGTACATGTAATGCAACCTCTACAAATAGCAGCAATAGTATCATTAG
TAGTAGTAGCAATAATAGCAATAGTTGTGTGGACCATAGTGTTTATAGAATATAGGAAAATATTAAGACAAAG
AAAGATAGACAGGATACTTGATAGGATAAGAGACAGAGCAGAAGACAGTGGCAATGAGAGCGAAGGAGATCAG
GAAGAATTGTCAGCTCTTGTTGACATGGGGCACGATGCTCCTTGGAATGTTGATGATCTGTAGTGCTACAGAA
AAATTGTGGGTTACAGTGTATTATGGGGTACCTGTGTGGAAAGAAGCAACCACCACTCTATTTTGTGCATCAG
ATGCTAAAGCATATGACACAGAAGCACATAATGTTTGGGCCACACATGCCTGTGTACCCACAGACCCTAGCCC
ACAAGAAGTAAACTTGACAAATGTGACAGAAAATTTTAACATGTGGAAAAATAACATGGTAGAACAGATGCAT
GAGGATATAATCAGTTTATGGGATCAAAGTCTAAAGCCATGTGTAAAATTAACCCCACTCTGTGTCACTTTAA
ATTGCACTGATGTGACGAATGGTACTGATGTGACGAATGGTACTATTGGGAACAGAACGGTAGACACAGAAAT
GGAGGGAGAAATAAAAAACTGCTCTTTCAATATTACCACAAGTATAACAAATAAGTTGCAGAAAGAATATGCC
CTTTTTTATAAACTTGATGTAGTACCAATAGATAAGAATGATAGTAATGATAAGAATAATAATTATAGTAGTT
ATATGTTGATAAATTGTAACACCTCAGTCATTACACAGGCCTGTCCAAAGGTATCCTTTGAACCAATTCCCAT
ACATTATTGTGCCCCGGCTGGTTTTGCGATTCTAAAGTGTAAGGATAAGATGTTCAATGGAACAGGACCATGC
AAAAATGTCAGCACAGTACATTGTACACATGGAATTAGGCCAGTAGTGTCAACTCAATTGCTGTTAAATGGCA
GTCTAGCAGAAGGAGGGGTAGTAATTAAATCTGAAAACTTCTCGGACAATGCTAAGAACATAATAGTACAGCT
GAACGAATCTGTAGAAATTAATTGTACAAAACCCAATAAGTATATAACAAAACAAGGTATATACTCAGTACGG
GGAAGAACATTATATGCAACAGGAAAGATAACAGGAAATATAAAAAAGGCACATTGTAACCTTAGTGAAACAA
ATTGGGAAAACACTTTAAAACAGATAGCTATAAAATTAGGAGAACAATATGGGAGGAATAAAACAATAGCCTT
TAGGAACTCCTCAGGAGGGGACCCAGAAATTGTAATGCACAGTTTTAATTGTGGAGGGGAATTTTTCTACTGT
GATACAACACAGCTGTTTAATAGTACTTGGGTGGCTGGAAATAATACTTATAGGAAAAATAATACTTATAGTA
GTAATAATACTGCAGGCAATATCACACTCACATGCAGAATAAAACAAATTATAAACAGGTGGCAGGAAGTAGG
AAAAGCAATGTATGCCCCTCCCATCGAAGGACTAATTAAATGTAGCTCAAATATTACAGGGCTGATATTAACA
AGAGATGGAGGCAACGAAAGCCAGTCTGACCCTGAGACCTTCAGACCTGCAGGGGGAGATATGAAGGACAATT
GGAGAAGTGAATTATATAAATATAAAGTAGTAAGAATTGAGCCATTAGGAGTAGCACCCACCGAGGCAAAGAG
AAGAGTGGTGCAGAGAGAAAAAAGAGCAGTGGGACTAGGAGTGATGTTCCTTGGGTTCTTGGGAGCAGCAGGA
AGCGCTATGGGCGCAGCGTCAGTGACGCTGACGGTACAGGCCAGACAATTATTGTCTGGTATAGTGCAACAGC
AGAACAATCTGCTGAGGGCTATTGAGGCGCAACAGCATATGTTGCAACTCACAGTCTGGGGCATTAAGCAGCT
CCAGGCAAGAGTCCTGGCTGTGGAAACATACCTAAAGGATCAACAGCTCCTAGGGATTTGGGGTTGCTCTGGA
AAACTCATCTGCACCACTGCTGTGCCTTGGAATGCTAGTTGGAGTAATAAATCTCTGAGTGACATTTGGAATA
ACACGACCTGGATGCAGTGGGACAAGGAAATTAACAATTACACAAGCTTAATATACACCTTACTTGAAGAATC
GCAGTACCAACAAGAAAAAAATGAACAAGAACTATTGGAATTGGATAAGTGGGCAAGTTTATGGAATTGGTTT
GACATATCGAATTGGCTGTGGTATATAAAAATATTCATAATGATAGTAGGAGGCTTAGTAGGTTTAAGAATAG
TTTTTGCTGTGCTTTCTATAGTGAATAGAGTTAGGCAGGGATACTCACCATTGTCATTCCAGACCCGCCTCCC

AGCTCAGAGGGGACCCGACAGGCCCGAAGGAATCGAAGAAGAAGGTGGAGAGAGAGACAGAGGCAGATCCGAA
CGATTAGTGACTGGATTCTTAGCACTCATCTGGGACGACCTGCGGAGCCTGTGCCTCTTCAGCTACCACCGCT
TGAGAGACTTAATCTTGATTGCAGCGAGGATTCTGGAACTTCTGGGACGCAGGGGGTGGGAAATCCTCAAATA
TTGGTGGAGTCTCCTGCAGTATTGGAGTCAGGAACTAAAGAATAGTGCTGTTAGCTTGCTTAATGTCACAGCT
ATTGCAGTAGCTGAGGGAACAGATAGGATTATAGAAGTAGTACAAAGGTTTGGTAGAGCTATCCTTCACATAC
CTACAAGAATAAGACAGGGCTTAGAAAGGGCTTTGCTATAACATGGGTGGCAAGTGGTCAAAACGTAGTAAGG
GTGGTGAATGGCCTGCTGTGAGGGAAAAAATGAAACAAGCTGAGCCAGCAGCAGAAGGGGTGGGAGCAGCATC
TCGAGACTTGGCAAAATATGGAGCACTCACAAGTAGCAATACAACAACTAATAATGCTGCTTGTGCCTGGCTA
GAAGCACAAGAGGAGGAGGAAGTGGGCTTTCCAGTCAGACCTCAGGTACCTTTAAGACCAATGACTTACAAGG
CAGCGTTTGATCTTGGCTTCTTTTTAAAAGAAAAGGGGGGACTGGAAGGGCTAATTTACTCCCAAAAGAGACA
AGACATCCTTGATTTGTGGGTCTACCACACACAAGGCTACTTCCCTGACTGGCAGAACTACACACCAGGGCCA
GGGGTCAGATATCCACTGACCTTTGGATGGTGCTTCAAGCTAGTACCAGTTGAGCCAAAGCAGGTAGAAGAGG
CCAATGAAGGAGAGAACAACACCCTGTTACACCCTATGAGCCTGCATGGGATGGATGATCCAGAGAGAGAAGT
GTTAGTGTGGAAGTTTGACAGCCGCCTAGCATTGCATCACATGGCCAAAGAGCTGCATCCGGAGTACTTCAAA
GACTGCTGACATCGAGCTTTCTACAAGGGACTTTCCGCTGGGGACTTTCCAGGGAGGTGTGGCCTGGGCGGGA
CTGGGGAGTGGCGAGCCCTCAGATGCTGCATATAAGCAGCTGCTTTCTGCCTGTACTGGGTCTCTCTGGTTAG
ACCAGATCTGAGCCTGGGAGCTCTCTGGCTAACTAGGGAACCCACTGCTTAAGCCTCAATAAAGCTTGCC

```
>T-16A HIV-1 genome, derived from RNA genomic sequence
GTGGCGCCCGAACAGGGACTAGAAAGCGAAAGTAAGACCAGAGGAGCTCTCTCGACGCAGGACTCGGCTTGCT
GAAGCGCGCACGGCAAGAGGCGAGGGGCGGCGACTGGTGAGTACGCCAAAAATAAAATTTTGACTAGCGGAGG
CTAGAAGGAGAGAGATGGGTGCGAGAGCGTCAGTATTAAGCGGGGGAGAATTAGATAGATGGGAAAAAATTCG
GTTACGGCCAGGGGGAAAGAAAAAGTATAAATTAAAACATATAGTATGGGCAAGCAGGGAACTAGAACGATTC
GCAGTTAACCCTGGCCTTTTAGAGACATCAGGAGGCTGTAGACAAATATTGGAACAGCTACAACCATCCCTTC
AGACAGGATCAGAAGAACTTAAATCATTATATAATACAATAGCAACCCTCTATTGTGTGCATCAAAAGATAGA
TGTAAAAGACACCAAGGAAGCTTTAGACAAAATAGAGGAAGAGCAAAACAAAAGTAAGAAAAAGGCACAGCAA
GCAGCAGCAGCAGCTGACACAGGAAGCAGCAGCAAGGTCAGCCAAAATTATCCTATAGTGCAAAATCTACAGG
GGCAAATGGTACATCAGCCCATATCACCTAGAACTTTAAATGCATGGGTAAAAGTAGTAGAAGAGAAGGCTTT
CAGTCCAGAAGTAATACCCATGTTTTCAGCATTATCAGAAGGAGCCACCCCACAAGATTTAAACACCATGCTA
AATACAGTGGGGGGACATCAAGCAGCCATGCAAATGTTAAAAGAGACCATCAATGAGGAAGCTGCAGAATGGG
ATAGATTACACCCAGTGCATGCAGGGCCTATTGCACCAGGCCAGATGAGAGAACCAAGGGGAAGTGACATAGC
AGGAACTACTAGTACCCTTCAGGAACAAATAGGATGGATGACAAATAATCCACCTATCCCAGTAGGAGAAATC
TATAAAAGATGGATAATCCTGGGATTAAATAAAATAGTAAGAATGTATAGCCCTACCAGCATTTTGGACATAA
GACAAGGACCAAAGGAACCCTTTAGAGACTATGTAGACAGGTTCTATAAGACTCTAAGAGCCGAGCAAGCTTC
ACAGGAAGTAAAAAATTGGATGACAGAAACCTTGTTGGTCCAAAATGCGAACCCAGATTGTAAGACTATCTTA
AAAGCATTGGGGCCAGCAGCTACACTAGAAGAAATGATGACAGCATGTCAGGGAGTGGGGGGACCCGGCCATA
AAGCAAGAGTCTTGGCTGAAGCAATGAGCCAAGTAACAAATTCAGCTACCATAATGATGCAGAAAGGCAATTT
TAGGAGCCAAAGAAAGACTGTTAAGTGTTTCAATTGTGGCAAAGAAGGGCACATAGCCAAAAATTGCAGGGCT
CCTAGGAAAAAGGGCTGTTGGAAATGTGGAAGGGAAGGACACCAAATGAAAGATTGTACTGAGAGACAGGCTA
ATTTTTTAGGGAAAATCTGGTCTTCCCACAAGGGAAGGCCAGGGAATTTTCTTCAGAGCAGACCAGAGCCAAC
AGCCCCACCAGCAGAGAGCTTCAGGTTTGGGGAGGAGACAGCAACTCCCCCTCAGAAGCAGGAGCCGATAGAC
AAGGAACTATATCCTTTAGCTTCCCTCAGATCACTCTTTGGCAACGACCCCTCGTCACAATAAGAATAGGGGG
GCAACTAAAAGAAGCTCTATTAGATACAGGAGCAGATGATACAGTATTAGAAGACATGAGTTTGCCAGGAAGA
TGGAAACCAAAAATGATAGGGGGAATTGGAGGTTTTATCAAAGTAAGACAGTATGATCAGATACCAGTAGAAA
TCTGTGGACATAAAGCTGTAGGTACAGTATTAGTAGGACCCACACCTGTCAACATAATTGGAAGAAATCTGTT
GACTCAGATTGGCTGCACTTTAAATTTTCCCATTAGTCCTATTGAAACTGTACCAGTAAAATTAAAGCCAGGA
ATGGATGGCCCAAAAGTTAAACAATGGCCATTGACAGAAGAGAAAATAAAAGCATTAGTAGAAATTTGTACAG
GAATGGAAAAAGAAGGGAAAATTTCAAAAATTGGGCCTGAAAATCCATACAATACTCCAGTATTTGCCATAAA
GAAAAAAGACAGTACTAAATGGAGAAAATTAGTAGACTTCAGAGAACTTAATAAGAGAACTCAAGACTTCTGG
GAAGTTCAATTAGGAATACCACATCCAGCAGGGTTAAAAAAGAAAAAATCAGTAACTGTACTGGATGTGGGTG
ATGCATATTTTTCAGTTCCCTTAGATAAAGACTTCAGGAAGTATACTGCATTTACCATACCTAGTATAAACAA
TGAGACACCAGGGATTAGATATCAGTACAATGTGCTTCCACAAGGATGGAAAGGATCACCAGCAATATTCCAA
AGTAGCATGACAAAAATCTTAGAGCCTTTTAGAAAACAAAATCCAGACATAGTTATCTATCAATACATGGATG
ATTTGTATGTAGGATCTGACTTAGAAATAGGGCAACATAGAACAAAAATAGAGGAACTGAGACAACATCTGTT
GAGGTGGGGATTTACCACACCAGACAAAAAGCATCAGAAAGAACCTCCATTCCTTTGGATGGGTTATGAACTC
CATCCTGATAAATGGACAGTACAGCCTATAGTGCTGCCAGAAAAAGACAGCTGGACTGTCAATGACATACAGA
AGTTAGTGGGAAAATTGAATTGGGCAAGTCAGATTTATGCAGGGATTAAAGTAAAGCAATTATGTAAACTCCT
TAGGGGAACCAAATCACTAACAGAAGTAGTACCACTGACAGAAGAAGCAGAGCTAGAACTGGCAGAAAACAGG
GAGATTCTAAAAGAACCAGTACATGGAGTGTATTATGATCCATCAAAAGACTTAATGGCAGAAATACAGAAGC
AGGAGAATGGTCAGTGGACATATCAAATTTATCAGGAGCCACATAAGAATCTGAAAACAGGAAAGTATGCAAG
AATGAGGGGTGCCCACACTAATGATGTAAGACAATTAACAGAGGCAGTGCAAAAAATAGCCAATGAAAGCATA
GTAATATGGGGAAAGATTCCTAAATTTAAATTACCCATACAAAAGGAAACATGGGAAGCATGGTGGATGGAGT
ATTGGCAAGCCACCTGGATTCCTGAGTGGGAGTTTGTCAATACCCCTCCCTTAGTGAAATTATGGTATCAGTT
AGAGAAAGAACCCATAGTAGGAGCAGAAACTTTCTATGTAGATGGGGCAGCTAATAGGGAAACTAAATTGGGA
AAAGCAGGATATGTTACTGACAGAGGAAGACAAAAGGTTGTCTCCCTAACGGACACAACAAATCAGAAGACTG
AGTTACAAGCAATTTATCTAGCTTTGCAGGATTCGGGATTAGAAGTAAATGTAGTAACAGACTCACAATATGC
ATTAGGAATCATTCAAGCACAACCAGATAAGAGTGAATCAGAGTTAGTCAATCAGATAATAGAGCAGTTAATA
AAAAAGGAAAAGGTCTACCTGGCATGGGTACCAGCACACAAAGGAATTGGAGGAAATGAACAAGTAGATAAAT
TAGTCAGTGCTGGAATTAGGAAAGTACTATTTTTAGATGGAATAGATAAGGCCCAAGATGAACATGAGAAATA
TCACAGTAATTGGAGAGCAATGGCTAGTGATTTTAACCTGCCACCTATAGTAGCAAAAGAAATAGTAGCTAGC
TGTGACAAATGTCAGCTAAAAGGAGAAGCCATGCATGGACAAGTAGACTGTAGTCCAGGAATATGGCAGCTAG
ACTGTACACACTTAGAAGGAAAAGTTATCCTGGTAGCAGTTCATGTGGCCAGTGGATATATAGAAGCAGAAGT
```

TATTCCAGCAGAGACAGGGCAAGAAACAGCATACTTTATCTTAAAATTAGCAGGAAGATGGCCAGTAAAAATT
ATACATACAGACAATGGCCCCAATTTCATCAGTACTGCGGTTAAAGCCGCCTGTTGGTGGGCAGGGATCAAGC
AGGAATTTGGTATTCCCTACAATCCCCAAAGTCAAGGAGTAGTGGAATCTATGAATAATGAATTAAAGAAAAT
TATAGGACAGGTAAGAGATCAAGCTGAACATCTTAAGACAGCAGTACAAATGGCAGTATTCATCCACAATTTT
AAAAGAAAAGGGGGGATTGGGGGGTACAGTGCAGGGGAAAGAATAGTAGACATAATATCAACAGACATACAAA
CTAAAGAATTACAAAAACAGATTACAAAAATTTCAAAATTTCGGGTTTATTACAGGGACAGCAGAGATCCACT
TTGGAAAGGACCAGCAAAGCTTCTCTGGAAAGGTGAAGGGGCAGTAGTAATACAAGATAATAGTGATATAAAA
GTAGTGCCAAGAAGAAAAGCAAAGATCATTAGGGATTATGGAAAACAGATGGCAGGTGATGATTGTGTGGCAA
GTAGACAGGATGAGGATTAGATCATGGAAAAGCTTAGTAAAACACCATATGTATATTTCAGGGAAAGCTAAGA
AATGGTTTTATAGACATCACTATGAAAGCACTCATCCAAGAATAAGTTCAGAAGTACACATCCCACTAGGGGA
TGCTAAATTAGTAGTAACAACATATTGGGGTCTGCATACAGGAGAAAGAGATTGGCATTTGGGTCAGGGAGTC
TCCATAGAATGGAGGAAAAGGAGATATAGCACACAAGTAGACCCTGACCTAGCAGACCAACTAATTCATTCGT
ATTATTTTGATTGTTTTTCAGAATCTGCTATAAGAAATGCCATATTAGGACATATAGTTAGACCTAGGTGTGA
GTATCAAGCAGGACATAACAAGGTAGGATCTCTACAGTACTTGGCACTAACAGCATTAATAACACCAAAAAAG
ATAAAGCCACCTTTGCCTAGTGTTAAGAAACTGACAGAGGATAGATGGAACAAGCCCCAGAGGACCAAGGGCC
ACAAAGGGAGCCATACAATGAATGGACACTAGAGCTTTTAGAGGAACTAAAAAATGAAGCTGTTAGACATTTT
CCTAGGACATGGCTCCATGGCTTAGGACAATATATCTATGAAACTTATGGGGATACTTGGGCAGGAGTGGAAG
CCATAATAAGAATTCTGCAACAACTGCTGTTTATTCATTTCAGAATTGGATGTCACCATAGCAGAATAGGCAT
TATTCTACAGAGGAGAGGAAGAAATGGAGCCAGTAGATCCTAGACTAGAGCCCTGGAAGCATCCAGGAAGTCA
GCCTAAAACTGCTTGTACCAAATGCTATTGTAAAAAGTGTTGCTTGCATTGCCAAGTTTGCTTCATAACAAAA
GGCTTAGGCATCTCCTATGGCAGGAAGAAGCGGAGACAGCGACGAAGACCTCCTCAAAACAGTGAGGCTCATC
AAGTTTCTCTATCAAAGCAGTAAGTAGTACATGTAATGCAACCTTTACAAATAGCAGCAATAGTATCATTAGT
AGTAGTAGCAATAATAGCAATAGTTGTGTGGACTATAGTGTTTATAGAATATAGGAAAATATTAAGACAAAGA
AAAATAGACAGGTTACTTGATAGGATAAGAGACAGAGCAGAAGACAGTGGCAATGAGAGCGAAGGAGATCAGG
AAGAATTGTCAGCTCTTATTGACATGGGGCACGATGCTCCTTGGGATGTTGATGATCTGTAGTGCTACAGAAA
AATTGTGGGTTACAGTGTATTATGGGGTACCTGTGTGGAAAGAAGCAACCACCACTCTATTTTGTGCATCAGA
TGCCAAAGCATATGACACAGAGGTACATAATGTTTGGGCCACACATGCCTGTGTACCCACAGACCCTAGCCCA
CAAGAAGTAAACTTAACAAATGTGACAGAAAATTTTAACATGTGGAAAAATAACATGGTAGAACAGATGCATG
AGGATATAATCAGTTTATGGGATCAAAGCCTAAAGCCATGTGTAAAATTAACCCCACTCTGTGTCACTTTAAA
TTGCAGTGATGTGACAAATAGTAGTATCACCAATAGTAGTAGTAGTGAGAGTACCAATAATAATAGAACGGTA
GACAAAGAGATGGAGGGAGAAATAAAAAACTGCTCTTTTAATATTACCACAAGTATAACAAATAAGTTGCAGA
AAGAATATGCCCTTTTTTATAAACTTGATGTAGTACCAATAGATAAGAATAATAGTAATAATAAGAATGATAG
TAATAATAGTTATATGTTGATAAATTGTAACACCTCAGTCATTACACAGGCCTGTCCAAAGGTATCCTTTGAA
CCAATTCCCATACATTATTGTGCCCCGGCTGGTTTTGCGATTCTAAAGTGTAAGGATAAGATGTTCAATGGAA
CAGGACCATGCAAAAATGTCAGCACAGTACATTGTACACATGGAATTAGGCCAGTAGTGTCAACTCAATTGCT
GTTAAATGGCAGTCTAGCAGAAGGAGGGGTAGTAGTTAAATCTGAAAATTTCACGAACAATGCTAAAAACATA
ATAGTACAGCTAAACGAATCTGTAGAAATTGAGTGTGAAAGACCCAATAACAATACGAGAAAAGGTATACACA
CAGCATGGGGAAGAGCATTATATGCAACAGGAGAGATAATAGGAAATATAAGAAAAGCATATTGTAACCTTAA
TAAAACAAATTGGGAAAACACTTTAAAACAGATAGTTATCAAATTAGGAGAACAATATGAGAATAAAACAATA
GCCTTTAAGAACTCCTCAGGAGGGGACCCAGAAATTGTAATGCACAGTTTTAATTGTGGAGGGGAATTTTTCT
ACTGTGATACATCACAGCTGTTTAATAGTACTTGGCAGCTTAATAAGACTGATAATACTTATAGTAACAATAA
TACTGAAAAGTTAAATAGCACTGAAGGTGGCAATATCACACTCACATGCAGAATAAAACAAATTATAAACAGA
TGGCAGGAAGTAGGAAAAGCAATGTATGCCCCTCCCATCAGAGGACTAATCAGATGTAGATCAAATATTACAG
GGCTGATATTAACAAGAGATGGAGGCAAGGACAACACAAGCGAGTCTAACACCACTGAGATCTTCAGACCTGG
AGGAGGAGATATGAGGGACAATTGGAGAAGTGAATTATATAAATATAAAGTAGTAAGAATTGAGCCATTAGGA
GTAGCACCCACCAAGGCAAAGAGAAGAGTGGTGCAGAGAGAAAAAAGAGCAGTAGGACTAGGAGCTCTGTTCC
TTGGGTTCTTGGGAGCAGCAGGAAGCACTATGGGCGCAGCGTCAGTGACGCTGACGGTACAGGCCAGACAATT
ATTGTCTGGTATAGTGCAACAGCAGAACAATCTGCTGAGGGCTATTGAGGCGCAACAACATCTGTTGCAACTC
ACAGTCTGGGGCATCAAGCAGCTCCAGGCAAGAGTCCTGGCTGTGGAAACATACCTAAAGGATCAACAGCTCC
TAGGGATTTGGGGTTGCTCTGGAAAACTCATCTGCACCACTGCTGTGCCTTGGAATGCTAGTTGGAGTAATAA
ATCTCTGAGTGACATTTGGGATAACATGACCTGGATGCAGTGGGACAAGGAAATTAACAATTACACAAACTTA
ATATACACCTTACTTGAAAAATCGCAGAACCAACAAGAAAAAAATGAACAAGAACTATTGGAGTTGGATAAGT
GGGCAAGTCTGTGGAATTGGTTTGACATATCAAATTGGCTGTGGTATATAAAAATATTCATAATGATAGTAGG
AGGCTTAGTAGGTTTAAGAATAATTCTTGCTGTGCTTTCTATAGTGAATAGAGTTAGGCAGGGATACTCACCA

CTGTCATTCCAGACCCGCCTCCCAGCCCAGAGGGGACCCGACAGGCCCGAAGGAATCGAAGAAGAAGGTGGAG
AGAGAGACAGAGACAGATCCGTACGATTAGTGACTGGATTCTTAGCACTCATCTGGGACGACCTGCGGAGCCT
GTGCCTCTTCAGCTACCACCACTTGAGAGACTTACTCTTGATTGCAGCGAGGATTGTGGAACTTCTGGGACGC
AGGGGGTGGGAAATCCTCAAGTATTGGTGGAGTCTCCTGCAGTATTGGAGTCAGGAACTAAAGAATAGTGCTG
TTAGCTTGCTTAATGCCACAGCTATCGCAGTAGCTGAGGGAACAGATAGGATTATAGAAGTAGTACAAAGGTT
TGGTAGAGCTATCCTTCACATACCTACAAGAATAAGACAGGGCTTAGAAAGGGCTTTGCTATAAGATGGGTGG
CAAGTGGTCAAAACGTAGCAAGGGTGGTGAATGGCCTGCTGTGAGGGAAAAAATGAAACAAGCTGAGCCAGCA
GCAGAAGGGGTGGGAGCAGCATCTCGAGACTTGGCAAAATATGGAGCACTTACAAGTAGCAATACAGCAACTA
ATAATGCTGCTTGTGCCTGGCTAGAAGCACAAGCACAAGAGGAGGAGGAAGTGGGCTTTCCAGTCAGACCTCA
GGTACCTTTAAGACCAATGACTTACAAGGGAGCGTTCGATCTTAGCTTCTTTTTAAAAGAAAAGGGGGGACTG
GAAGGGCTAATTCACTCCCAAAAGAGACAAGACATCCTTGATCTGTGGATCTACCACACACAAGGCTACTTCC
CTGATTGGCAGAACTACACACCAGGGCCAGGGATCAGATATCCACTGACCTTTGGATGGTGCTTCAAGCTAGT
ACCAGTTGATCCAAAGCAGGTGGAAGAGGCTAATGAAGGAGAGAACAACACCTTGTTACACCCTATGAGCCTG
CATGGGATGGATGATCCAGAGAGAGAAGTGTTAGTGTGGAAGTTTGACAGCCGCCTAGCACGTCATCACATGG
CCAAAGAGCTGCATCCGGAATACTTCAAAGACTGCTGACATCGAGCTTTCTACAAGGGACTTTCCGCTGGGGA
CTTTCCAGGGAGGTGTGGCCTGGGCGGACTGGGGAGTGGCGAGCCCTCAGATGCTGCATATAAGCAGCTGCT
TTCTGCCTGTACTGGGTCTCTCTGGTTAGACCAGATCTGAGCCTGGGAGCTCTCTGGCTAACTAGGGAACCCA
CTGCTTAAGCCTCAATAAAGCTTGCC

>T-21D HIV-1 genome, derived from RNA genomic sequence
GTGGCGCCCGAACAGGGACTAGAAAGCGAAAGTAAGACCAGAGGAGCTCTCTCGACGCAGGACTCGGCTTGCT
GAAGCGCGCACGGCAAGAGGCGAGGGGCGGCGACTGGTGAGTACGCCAAAAATAAAATTTTGACTAGCGGAGG
CTAGAAGGAGAGAGATGGGTGCGAGAGCGTCAGTATTAAGCGGGGGAGAATTAGATAGATGGGAAAAAATTCG
GTTACGGCCAGGGGGAAAGAAAAAGTATAAATTAAAACATATAGTATGGGCAAGCAGGGAACTAGAACGATTC
GCAGTTAACCCTGGCCTTTTAGAGACATCAGGAGGCTGTAGACAAATATTGGAACAGCTACAACCATCCCTTC
AGACAGGATCAGAAGAACTTAAATCATTATATAATACAATAGCAACCCTCTATTGTGTGCATCAAAAGATAGA
TGTAAAAGACACCAAGGAAGCTTTAGACAAAATAGAGGAAGAGCAAAACAAAAGTAAGAAAAAGGCACAGCAA
GCAGCAGCAGCAGCTGACACAGGAAGCAGCAGCAAGGTCAGCCAAAATTATCCTATAGTGCAAAATCTACAGG
GGCAAATGGTACATCAGCCCATATCACCTAGAACTTTAAATGCATGGGTAAAAGTAGTAGAAGAGAAGGCTTT
CAGTCCAGAAGTAATACCCATGTTTTCAGCATTATCAGAAGGAGCCACCCCACAAGATTTAAACACCATGCTA
AATACAGTGGGGGGACATCAAGCAGCCATGCAAATGTTAAAAGAGACCATCAATGAGGAAGCTGCAGAATGGG
ATAGATTACACCCAGTGCATGCAGGGCCTATTGCACCAGGCCAGATGAGAGAACCAAGGGGAAGTGACATAGC
AGGAACTACTAGTACCCTTCAGGAACAAATAGGATGGATGACAAATAATCCACCTATCCCAGTAGGAGAAATC
TATAAAAGATGGATAATCCTGGGATTAAATAAAATAGTAAGAATGTATAGCCCTACCAGCATTTTGGACATAA
GACAAGGACCAAAGGAACCCTTTAGAGACTATGTAGACAGGTTCTATAAGACTCTAAGAGCCGAGCAAGCTTC
ACAGGAAGTAAAAAATTGGATGACAGAAACCTTGTTGGTCCAAAATGCGAACCCAGATTGTAAGACTATCTTA
AAAGCATTGGGGCCAGCAGCTACACTAGAAGAAATGATGACAGCATGTCAGGGAGTGGGGGGACCCGGCCATA
AAGCAAGAGTCTTGGCTGAAGCAATGAGCCAAGTAACAAATTCAGCTACCATAATGATGCAGAAAGGCAATTT
TAGGAGCCAAAGAAAGACTGTTAAGTGTTTCAATTGTGGCAAAGAAGGGCACATAGCCAAAAATTGCAGGGCT
CCTAGGAAAAAGGGCTGTTGGAAATGTGGAAGGGAAGGACACCAAATGAAAGATTGTACTGAGAGACAGGCTA
ATTTTTTAGGGAAAATCTGGTCTTCCCACAAGGGAAGGCCAGGGAATTTTCTTCAGAGCAGACCAGAGCCAAC
AGCCCCACCAGCAGAGAGCTTCAGGTTTGGGGAGGAGACAGCAACTCCCCCTCAGAAGCAGGAGCCGATAGAC
AAGGAACTATATCCTTTAGCTTCCCTCAGATCACTCTTTGGCAACGACCCCTCGTCACAATAAGAATAGGGGG
GCAACTAAAAGAAGCTCTATTAGATACAGGAGCAGATGATACAGTATTAGAAGACATGAGTTTGCCAGGAAGA
TGGAAACCAAAAATGATAGGGGGAATTGGAGGTTTTATCAAAGTAAGACAGTATGATCAGATACCAGTAGAAA
TCTGTGGACATAAAGCTGTAGGTACAGTATTAGTAGGACCCACACCTGTCAACATAATTGGAAGAAATCTGTT
GACTCAGATTGGCTGCACTTTAAATTTTCCCATTAGTCCTATTGAAACTGTACCAGTAAAATTAAAGCCAGGA
ATGGATGGCCCAAAAGTTAAACAATGGCCATTGACAGAAGAGAAAATAAAAGCATTAGTAGAAATTTGTACAG
GAATGGAAAAAGAAGGGAAAATTTCAAAAATTGGGCCTGAAAATCCATACAATACTCCAGTATTTGCCATAAA
GAAAAAAGACAGTACTAAATGGAGAAAATTAGTAGACTTCAGAGAACTTAATAAGAGAACTCAAGACTTCTGG
GAAGTTCAATTAGGAATACCACATCCAGCAGGGTTAAAAAAGAAAAAATCAGTAACTGTACTGGATGTGGGTG
ATGCATATTTTTCAGTTCCCTTAGATAAAGACTTCAGGAAGTATACTGCATTTACCATACCTAGTATAAACAA
TGAGACACCAGGGATTAGATATCAGTACAATGTGCTTCCACAAGGATGGAAAGGATCACCAGCAATATTCCAA
AGTAGCATGACAAAAATCTTAGAGCCTTTTAGAAAACAAAATCCAGACATAGTTATCTATCAATACATGGATG
ATTTGTATGTAGGATCTGACTTAGAAATAGGGCAACATAGAACAAAAATAGAGGAACTGAGACAACATCTGTT
GAGGTGGGGATTTACCACACCAGACAAAAAGCATCAGAAAGAACCTCCATTCCTTTGGATGGGTTATGAACTC
CATCCTGATAAATGGACAGTACAGCCTATAGTGCTGCCAGAAAAAGACAGCTGGACTGTCAATGACATACAGA
AGTTAGTGGGAAAATTGAATTGGGCAAGTCAGATTTATGCAGGGATTAAAGTAAAGCAATTATGTAAACTCCT
TAGGGGAACCAAATCACTAACAGAAGTAGTACCACTGACAGAAGAAGCAGAGCTAGAACTGGCAGAAAACAGG
GAGATTCTAAAAGAACCAGTACATGGAGTGTATTATGATCCATCAAAAGACTTAATGGCAGAAATACAGAAGC
AGGAGAATGGTCAGTGGACATATCAAATTTATCAGGAGCCACATAAGAATCTGAAAACAGGAAAGTATGCAAG
AATGAGGGGTGCCCACACTAATGATGTAAGACAATTAACAGAGGCAGTGCAAAAAATAGCCAATGAAAGCATA
GTAATATGGGGAAAGATTCCTAAATTTAAATTACCCATACAAAAGGAAACATGGGAAGCATGGTGGATGGAGT
ATTGGCAAGCCACCTGGATTCCTGAGTGGGAGTTTGTCAATACCCCTCCCTTAGTGAAATTATGGTATCAGTT
AGAGAAAGAACCCATAGTAGGAGCAGAAACTTTCTATGTAGATGGGGCAGCTAATAGGGAAACTAAATTGGGA
AAAGCAGGATATGTTACTGACAGAGGAAGACAAAAGGTTGTCTCCCTAACGGACACAACAAATCAGAAGACTG
AGTTACAAGCAATTTATCTAGCTTTGCAGGATTCGGGATTAGAAGTAAATGTAGTAACAGACTCACAATATGC
ATTAGGAATCATTCAAGCACAACCAGATAAGAGTGAATCAGAGTTAGTCAATCAGATAATAGAGCAGTTAATA
AAAAAGGAAAAGGTCTACCTGGCATGGGTACCAGCACACAAAGGAATTGGAGGAAATGAACAAGTAGATAAAT
TAGTCAGTGCTGGAATTAGGAAAGTACTATTTTTAGATGGAATAGATAAGGCCCAAGATGAACATGAGAAATA
TCACAGTAATTGGAGAGCAATGGCTAGTGATTTTAACCTGCCACCTATAGTAGCAAAAGAAATAGTAGCTAGC
TGTGACAAATGTCAGCTAAAAGGAGAAGCCATGCATGGACAAGTAGACTGTAGTCCAGGAATATGGCAGCTAG
ACTGTACACACTTAGAAGGAAAAGTTATCCTGGTAGCAGTTCATGTGGCCAGTGGATATATAGAAGCAGAAGT

TATTCCAGCAGAGACAGGGCAAGAAACAGCATACTTTATCTTAAAATTAGCAGGAAGATGGCCAGTAAAAATT
ATACATACAGACAATGGCCCCAATTTCATCAGTACTGCGGTTAAAGCCGCCTGTTGGTGGGCAGGGATCAAGC
AGGAATTTGGTATTCCCTACAATCCCCAAAGTCAAGGAGTAGTGGAATCTATGAATAATGAATTAAAGAAAAT
TATAGGACAGGTAAGAGATCAAGCTGAACATCTTAAGACAGCAGTACAAATGGCAGTATTCATCCACAATTTT
AAAAGAAAAGGGGGGATTGGGGGGTACAGTGCAGGGGAAAGAATAGTAGACATAATATCAACAGACATACAAA
CTAAAGAATTACAAAAACAGATTACAAAAATTTCAAAATTTCGGGTTTATTACAGGGACAGCAGAGATCCACT
TTGGAAAGGACCAGCAAAGCTTCTCTGGAAAGGTGAAGGGGCAGTAGTAATACAAGATAATAGTGATATAAAA
GTAGTGCCAAGAAGAAAAGCAAAGATCATTAGGGATTATGGAAAACAGATGGCAGGTGATGATTGTGTGGCAA
GTAGACAGGATGAGGATTAGATCATGGAAAAGCTTAGTAAAACACCATATGTATATTTCAGGGAAAGCTAAGA
AATGGTTTTATAGACATCACTATGAAAGCACTCATCCAAGAATAAGTTCAGAAGTACACATCCCACTAGGGGA
TGCTAAATTAGTAGTAACAACATATTGGGGTCTGCATACAGGAGAAAGAGATTGGCATTTGGGTCAGGGAGTC
TCCATAGAATGGAGGAAAAGGAGATATAGCACACAAGTAGACCCTGACCTAGCAGACCAACTAATTCATTCGT
ATTATTTTGATTGTTTTTCAGAATCTGCTATAAGAAATGCCATATTAGGACATATAGTTAGACCTAGGTGTGA
GTATCAAGCAGGACATAACAAGGTAGGATCTCTACAGTACTTGGCACTAACAGCATTAATAACACCAAAAAAG
ATAAAGCCACCTTTGCCTAGTGTTAAGAAACTGACAGAGGATAGATGGAACAAGCCCCAGAGGACCAAGGGCC
ACAAAGGGAGCCATACAATGAATGGACACTAGAGCTTTTAGAGGAACTAAAAAATGAAGCTGTTAGACATTTT
CCTAGGACATGGCTCCATGGCTTAGGACAATATATCTATGAAACTTATGGGGATACTTGGGCAGGAGTGGAAG
CCATAATAAGAATTCTGCAACAACTGCTGTTTATTCATTTCAGAATTGGATGTCACCATAGCAGAATAGGCAT
TATTCTACAGAGGAGAGGAAGAAATGGAGCCAGTAGATCCTAGACTAGAGCCCTGGAAGCATCCAGGAAGTCA
GCCTAAAACTGCTTGTACCAAATGCTATTGTAAAAAGTGTTGCTTGCATTGCCAAGTTTGCTTCATAACAAAA
GGCTTAGGCATCTCCTATGGCAGGAAGAAGCGGAGACAGCGACGAAGACCTCCTCAAAACAGTGAGGCTCATC
AAGTTTCTCTATCAAAGCAGTAAGTAGTACATGTAATGCAACCTTTACAAATAGCAGCAATAGTATCATTAGT
AGTAGTAGCAATAATAGCAATAGTTGTGTGGACTATAGTGTTTATAGAATATAGGAAAATATTAAGACAAAGA
AAAATAGACAGGTTACTTGATAGGATAAGAGACAGAGCAGAAGACAGTGGCAATGAGAGCGAAGGAGATCAGG
AAGAATTGTCAGCTCTTATTGACATGGGGCACGATGCTCCTTGGGATGTTGATGATCTGTAGTGCTACAGAAA
AATTGTGGGTTACAGTGTATTATGGGGTACCTGTGTGGAAAGAAGCAACCACCACTCTATTTTGTGCATCAGA
TGCCAAAGCATATGACACAGAGGTACATAATGTTTGGGCCACACATGCCTGTGTACCCACAGACCCTAGCCCA
CAAGAAGTAAACTTAACAAATGTGACAGAAAATTTTAACATGTGGAAAAATAACATGGTAGAACAGATGCATG
AGGATATAATCAGTTTATGGGATCAAAGCCTAAAGCCATGTGTAAAATTAACCCCACTCTGTGTCACTTTAAA
TTGCAGTGATGTGACAAATAGTAGTATCACCAATAGTAGTAGTAGTGAGAGTACCAATAATAATAGAACGGTA
GACAAAGAGATGGAGGGAGAAATAAAAAACTGCTCTTTTAATATTACCACAAGTATAACAAATAAGTTGCAGA
AAGAATATGCCCTTTTTTATAAACTTGATGTAGTACCAATAGATAAGAATAATAGTAATAATAAGAATGATAG
TAATAATAGTTATATGTTGATAAATTGTAACACCTCAGTCATTACACAGGCCTGTCCAAAGGTATCCTTTGAA
CCAATTCCCATACATTATTGTGCCCCGGCTGGTTTTGCGATTCTAAAGTGTAAGGATAAGATGTTCAATGGAA
CAGGACCATGCAAAAATGTCAGCACAGTACATTGTACACATGGAATTAGGCCAGTAGTGTCAACTCAATTGCT
GTTAAATGGCAGTCTAGCAGAAGGAGGGGTAGTAGTTAAATCTGAAAATTTCACGAACAATGCTAAAAACATA
ATAGTACAGCTAAACGAATCTGTAGAAATTGAGTGTGAAAGACCCAATAACAATACGAGAAAAGGTATACACA
CAGCATGGGGAAGAGCATTATATGCAACAGGAGAGATAATAGGAAATATAAGAAAAGCATATTGTAACCTTAA
TAAAACAAATTGGGAAAACACTTTAAAACAGATAGTTATCAAATTAGGAGAACAATATGAGAATAAAACAATA
GCCTTTAAGAACTCCTCAGGAGGGGACCCAGAAATTGTAATGCACAGTTTTAATTGTGGAGGGGAATTTTTCT
ACTGTGATACATCACAGCTGTTTAATAGTACTTGGCAGCTTAATAAGACTGATAATACTTATAGTAACAATAA
TACTGAAAAGTTAAATAGCACTGAAGGTGGCAATATCACACTCACATGCAGAATAAAACAAATTATAAACAGA
TGGCAGGAAGTAGGAAAAGCAATGTATGCCCCTCCCATCAGAGGACTAATCAGATGTAGATCAAATATTACAG
GGCTGATATTAACAAGAGATGGAGGCAAGGACAACACAAGCGAGTCTAACACCACTGAGATCTTCAGACCTGG
AGGAGGAGATATGAGGGACAATTGGAGAAGTGAATTATATAAATATAAAGTAGTAAGAATTGAGCCATTAGGA
GTAGCACCCACCAAGGCAAAGAGAAGAGTGGTGCAGAGAGAAAAAAGAGCAGTAGGACTAGGAGCTCTGTTCC
TTGGGTTCTTGGGAGCAGCAGGAAGCACTATGGGCGCAGCGTCAGTGACGCTGACGGTACAGGCCAGACAATT
ATTGTCTGGTATAGTGCAACAGCAGAACAATCTGCTGAGGGCTATTGAGGCGCAACAACATCTGTTGCAACTC
ACAGTCTGGGGCATCAAGCAGCTCCAGGCAAGAGTCCTGGCTGTGGAAACATACCTAAAGGATCAACAGCTCC
TAGGGATTTGGGGTTGCTCTGGAAAACTCATCTGCACCACTGCTGTGCCTTGGAATGCTAGTTGGAGTAATAA
ATCTCTGAGTGACATTTGGGATAACATGACCTGGATGCAGTGGGACAAGGAAATTAACAATTACACAAACTTA
ATATACACCTTACTTGAAAAATCGCAGAACCAACAAGAAAAAAATGAACAAGAACTATTGGAGTTGGATAAGT
GGGCAAGTCTGTGGAATTGGTTTGACATATCAAATTGGCTGTGGTATATAAAAATATTCATAATGATAGTAGG
AGGCTTAGTAGGTTTAAGAATAATTCTTGCTGTGCTTTCTATAGTGAATAGAGTTAGGCAGGGATACTCACCA

CTGTCATTCCAGACCCGCCTCCCAGCCCAGAGGGGACCCGACAGGCCCGAAGGAATCGAAGAAGAAGGTGGAG
AGAGAGACAGAGACAGATCCGTACGATTAGTGACTGGATTCTTAGCACTCATCTGGGACGACCTGCGGAGCCT
GTGCCTCTTCAGCTACCACCACTTGAGAGACTTACTCTTGATTGCAGCGAGGATTGTGGAACTTCTGGGACGC
AGGGGGTGGGAAATCCTCAAGTATTGGTGGAGTCTCCTGCAGTATTGGAGTCAGGAACTAAAGAATAGTGCTG
TTAGCTTGCTTAATGCCACAGCTATCGCAGTAGCTGAGGGAACAGATAGGATTATAGAAGTAGTACAAAGGTT
TGGTAGAGCTATCCTTCACATACCTACAAGAATAAGACAGGGCTTAGAAAGGGCTTTGCTATAAGATGGGTGG
CAAGTGGTCAAAACGTAGCAAGGGTGGTGAATGGCCTGCTGTGAGGGAAAAAATGAAACAAGCTGAGCCAGCA
GCAGAAGGGGTGGGAGCAGCATCTCGAGACTTGGCAAAATATGGAGCACTTACAAGTAGCAATACAGCAACTA
ATAATGCTGCTTGTGCCTGGCTAGAAGCACAAGCACAAGAGGAGGAGGAAGTGGGCTTTCCAGTCAGACCTCA
GGTACCTTTAAGACCAATGACTTACAAGGGAGCGTTCGATCTTAGCTTCTTTTTAAAAGAAAAGGGGGGACTG
GAAGGGCTAATTCACTCCCAAAAGAGACAAGACATCCTTGATCTGTGGATCTACCACACACAAGGCTACTTCC
CTGATTGGCAGAACTACACACCAGGGCCAGGGATCAGATATCCACTGACCTTTGGATGGTGCTTCAAGCTAGT
ACCAGTTGATCCAAAGCAGGTGGAAGAGGCTAATGAAGGAGAGAACAACACCTTGTTACACCCTATGAGCCTG
CATGGGATGGATGATCCAGAGAGAGAAGTGTTAGTGTGGAAGTTTGACAGCCGCCTAGCACGTCATCACATGG
CCAAAGAGCTGCATCCGGAATACTTCAAAGACTGCTGACATCGAGCTTTCTACAAGGGACTTTCCGCTGGGGA
CTTTCCAGGGAGGTGTGGCCTGGGCGGGACTGGGGAGTGGCGAGCCCTCAGATGCTGCATATAAGCAGCTGCT
TTCTGCCTGTACTGGGTCTCTCTGGTTAGACCAGATCTGAGCCTGGGAGCTCTCTGGCTAACTAGGGAACCCA
CTGCTTAAGCCTCAATAAAGCTTGCC

>T-23C HIV-1 genome, derived from RNA genomic sequence
GTGGCGCCCGAACAGGGACTAGAAAGCGAAAGTAAGACCAGAGGAGCTCTCTCGACGCAGGACTCGGCTTGCT
GAAGCGCGCACGGCAAGAGGCGAGGGGCGGCGACTGGTGAGTACGCCAAAAATAAAATTTTGACTAGCGGAGG
CTAGAAGGAGAGAGATGGGTGCGAGAGCGTCAGTATTAAGCGGGGGAGAATTAGATAGATGGGAAAAAATTCG
GTTACGGCCAGGGGGAAAGAAAAAGTATAAATTAAAACATATAGTATGGGCAAGCAGGGAACTAGAACGATTC
GCAGTTAACCCTGGCCTTTTAGAGACATCAGGAGGCTGTAGACAAATATTGGAACAGCTACAACCATCCCTTC
AGACAGGATCAGAAGAACTTAAATCATTATATAATACAATAGCAACCCTCTATTGTGTGCATCAAAAGATAGA
TGTAAAAGACACCAAGGAAGCTTTAGACAAAATAGAGGAAGAGCAAAACAAAAGTAAGAAAAAGGCACAGCAA
GCAGCAGCAGCAGCTGACACAGGAAGCAGCAGCAAGGTCAGCCAAAATTATCCTATAGTGCAAAATCTACAGG
GGCAAATGGTACATCAGCCCATATCACCTAGAACTTTAAATGCATGGGTAAAAGTAGTAGAAGAGAAGGCTTT
CAGTCCAGAAGTAATACCCATGTTTTCAGCATTATCAGAAGGAGCCACCCCACAAGATTTAAACACCATGCTA
AATACAGTGGGGGGACATCAAGCAGCCATGCAAATGTTAAAAGAGACCATCAATGAGGAAGCTGCAGAATGGG
ATAGATTACACCCAGTGCATGCAGGGCCTATTGCACCAGGCCAGATGAGAGAACCAAGGGGAAGTGACATAGC
AGGAACTACTAGTACCCTTCAGGAACAAATAGGATGGATGACAAATAATCCACCTATCCCAGTAGGAGAAATC
TATAAAAGATGGATAATCCTGGGATTAAATAAAATAGTAAGAATGTATAGCCCTACCAGCATTTTGGACATAA
GACAAGGACCAAAGGAACCCTTTAGAGACTATGTAGACAGGTTCTATAAGACTCTAAGAGCCGAGCAAGCTTC
ACAGGAAGTAAAAAATTGGATGACAGAAACCTTGTTGGTCCAAAATGCGAACCCAGATTGTAAGACTATCTTA
AAAGCATTGGGGCCAGCAGCTACACTAGAAGAAATGATGACAGCATGTCAGGGAGTGGGGGGACCCGGCCATA
AAGCAAGAGTCTTGGCTGAAGCAATGAGCCAAGTAACAAATTCAGCTACCATAATGATGCAGAAAGGCAATTT
TAGGAGCCAAAGAAAGACTGTTAAGTGTTTCAATTGTGGCAAAGAAGGGCACATAGCCAAAAATTGCAGGGCT
CCTAGGAAAAAGGGCTGTTGGAAATGTGGAAGGGAAGGACACCAAATGAAAGATTGTACTGAGAGACAGGCTA
ATTTTTTAGGGAAAATCTGGTCTTCCCACAAGGGAAGGCCAGGGAATTTTCTTCAGAGCAGACCAGAGCCAAC
AGCCCCACCAGCAGAGAGCTTCAGGTTTGGGGAGGAGACAGCAACTCCCCCTCAGAAGCAGGAGCCGATAGAC
AAGGAACTATATCCTTTAGCTTCCCTCAGATCACTCTTTGGCAACGACCCCTCGTCACAATAAGAATAGGGGG
GCAACTAAAAGAAGCTCTATTAGATACAGGAGCAGATGATACAGTATTAGAAGACATGAGTTTGCCAGGAAGA
TGGAAACCAAAAATGATAGGGGGAATTGGAGGTTTTATCAAAGTAAGACAGTATGATCAGATACCAGTAGAAA
TCTGTGGACATAAAGCTGTAGGTACAGTATTAGTAGGACCCACACCTGTCAACATAATTGGAAGAAATCTGTT
GACTCAGATTGGCTGCACTTTAAATTTTCCCATTAGTCCTATTGAAACTGTACCAGTAAAATTAAAGCCAGGA
ATGGATGGCCCAAAAGTTAAACAATGGCCATTGACAGAAGAGAAAATAAAAGCATTAGTAGAAATTTGTACAG
GAATGGAAAAAGAAGGAAAATTTCAAAAATTGGGCCTGAAAATCCATACAATACTCCAGTATTTGCCATAAA
GAAAAAAGACAGTACTAAATGGAGAAAATTAGTAGACTTCAGAGAACTTAATAAGAGAACTCAAGACTTCTGG
GAAGTTCAATTAGGAATACCACATCCAGCAGGGTTAAAAAAGAAAAAATCAGTAACTGTACTGGATGTGGGTG
ATGCATATTTTTCAGTTCCCTTAGATAAAGACTTCAGGAAGTATACTGCATTTACCATACCTAGTATAAACAA
TGAGACACCAGGGATTAGATATCAGTACAATGTGCTTCCACAAGGATGGAAAGGATCACCAGCAATATTCCAA
AGTAGCATGACAAAAATCTTAGAGCCTTTTAGAAAACAAAATCCAGACATAGTTATCTATCAATACATGGATG
ATTTGTATGTAGGATCTGACTTAGAAATAGGGCAACATAGAACAAAAATAGAGGAACTGAGACAACATCTGTT
GAGGTGGGGATTTACCACACCAGACAAAAAGCATCAGAAAGAACCTCCATTCCTTTGGATGGGTTATGAACTC
CATCCTGATAAATGGACAGTACAGCCTATAGTGCTGCCAGAAAAAGACAGCTGGACTGTCAATGACATACAGA
AGTTAGTGGGAAAATTGAATTGGGCAAGTCAGATTTATGCAGGGATTAAAGTAAAGCAATTATGTAAACTCCT
TAGGGGAACCAAATCACTAACAGAAGTAGTACCACTGACAGAAGAAGCAGAGCTAGAACTGGCAGAAAACAGG
GAGATTCTAAAAGAACCAGTACATGGAGTGTATTATGATCCATCAAAAGACTTAATGGCAGAAATACAGAAGC
AGGAGAATGGTCAGTGGACATATCAAATTTATCAGGAGCCACATAAGAATCTGAAAACAGGAAAGTATGCAAG
AATGAGGGGTGCCCACACTAATGATGTAAGACAATTAACAGAGGCAGTGCAAAAAATAGCCAATGAAAGCATA
GTAATATGGGGAAAGATTCCTAAATTTAAATTACCCATACAAAAGGAAACATGGGAAGCATGGTGGATGGAGT
ATTGGCAAGCCACCTGGATTCCTGAGTGGGAGTTTGTCAATACCCCTCCCTTAGTGAAATTATGGTATCAGTT
AGAGAAAGAACCCATAGTAGGAGCAGAAACTTTCTATGTAGATGGGGCAGCTAATAGGGAAACTAAATTGGGA
AAAGCAGGATATGTTACTGACAGAGGAAGACAAAAGGTTGTCTCCCTAACGGACACAACAAATCAGAAGACTG
AGTTACAAGCAATTTATCTAGCTTTGCAGGATTCGGGATTAGAAGTAAATGTAGTAACAGACTCACAATATGC
ATTAGGAATCATTCAAGCACAACCAGATAAGAGTGAATCAGAGTTAGTCAATCAGATAATAGAGCAGTTAATA
AAAAAGGAAAAGGTCTACCTGGCATGGGTACCAGCACACAAAGGAATTGGAGGAAATGAACAAGTAGATAAAT
TAGTCAGTGCTGGAATTAGGAAAGTACTATTTTTAGATGGAATAGATAAGGCCCAAGATGAACATGAGAAATA
TCACAGTAATTGGAGAGCAATGGCTAGTGATTTTAACCTGCCACCTATAGTAGCAAAAGAAATAGTAGCTAGC
TGTGACAAATGTCAGCTAAAAGGAGAAGCCATGCATGGACAAGTAGACTGTAGTCCAGGAATATGGCAGCTAG
ACTGTACACACTTAGAAGGAAAAGTTATCCTGGTAGCAGTTCATGTGGCCAGTGGATATATAGAAGCAGAAGT

TATTCCAGCAGAGACAGGGCAAGAAACAGCATACTTTATCTTAAAATTAGCAGGAAGATGGCCAGTAAAAATT
ATACATACAGACAATGGCCCCAATTTCATCAGTACTGCGGTTAAAGCCGCCTGTTGGTGGGCAGGGATCAAGC
AGGAATTTGGTATTCCCTACAATCCCCAAAGTCAAGGAGTAGTGGAATCTATGAATAATGAATTAAAGAAAAT
TATAGGACAGGTAAGAGATCAAGCTGAACATCTTAAGACAGCAGTACAAATGGCAGTATTCATCCACAATTTT
AAAAGAAAAGGGGGGATTGGGGGGTACAGTGCAGGGGAAAGAATAGTAGACATAATATCAACAGACATACAAA
CTAAAGAATTACAAAAACAGATTACAAAAATTTCAAAATTTCGGGTTTATTACAGGGACAGCAGAGATCCACT
TTGGAAAGGACCAGCAAAGCTTCTCTGGAAAGGTGAAGGGGCAGTAGTAATACAAGATAATAGTGATATAAAA
GTAGTGCCAAGAAGAAAAGCAAAGATCATTAGGGATTATGGAAAACAGATGGCAGGTGATGATTGTGTGGCAA
GTAGACAGGATGAGGATTAGATCATGGAAAAGCTTAGTAAAACACCATATGTATATTTCAGGGAAAGCTAAGA
AATGGTTTTATAGACATCACTATGAAAGCACTCATCCAAGAATAAGTTCAGAAGTACACATCCCACTAGGGGA
TGCTAAATTAGTAGTAACAACATATTGGGGTCTGCATACAGGAGAAAGAGATTGGCATTTGGGTCAGGGAGTC
TCCATAGAATGGAGGAAAAGGAGATATAGCACACAAGTAGACCCTGACCTAGCAGACCAACTAATTCATTCGT
ATTATTTTGATTGTTTTTCAGAATCTGCTATAAGAAATGCCATATTAGGACATATAGTTAGACCTAGGTGTGA
GTATCAAGCAGGACATAACAAGGTAGGATCTCTACAGTACTTGGCACTAACAGCATTAATAACACCAAAAAAG
ATAAAGCCACCTTTGCCTAGTGTTAAGAAACTGACAGAGGATAGATGGAACAAGCCCCAGAGGACCAAGGGCC
ACAAAGGGAGCCATACAATGAATGGACACTAGAGCTTTTAGAGGAACTAAAAAATGAAGCTGTTAGACATTTT
CCTAGGACATGGCTCCATGGCTTAGGACAATATATCTATGAAACTTATGGGGATACTTGGGCAGGAGTGGAAG
CCATAATAAGAATTCTGCAACAACTGCTGTTTATTCATTTCAGAATTGGATGTCACCATAGCAGAATAGGCAT
TATTCTACAGAGGAGAGGAAGAAATGGAGCCAGTAGATCCTAGACTAGAGCCCTGGAAGCATCCAGGAAGTCA
GCCTAAAACTGCTTGTACCAAATGCTATTGTAAAAAGTGTTGCTTGCATTGCCAAGTTTGCTTCATAACAAAA
GGCTTAGGCATCTCCTATGGCAGGAAGAAGCGGAGACAGCGACGAAGACCTCCTCAAAACAGTGAGGCTCATC
AAGTTTCTCTATCAAAGCAGTAAGTAGTACATGTAATGCAACCTTTACAAATAGCAGCAATAGTATCATTAGT
AGTAGTAGCAATAATAGCAATAGTTGTGTGGACTATAGTGTTTATAGAATATAGGAAAATATTAAGACAAAGA
AAAATAGACAGGTTACTTGATAGGATAAGAGACAGAGCAGAAGACAGTGGCAATGAGAGCGAAGGAGATCAGG
AAGAATTGTCAGCTCTTATTGACATGGGGCACGATGCTCCTTGGGATGTTGATGATCTGTAGTGCTACAGAAA
AATTGTGGGTTACAGTGTATTATGGGGTACCTGTGTGGAAAGAAGCAACCACCACTCTATTTTGTGCATCAGA
TGCCAAAGCATATGACACAGAGGTACATAATGTTTGGGCCACACATGCCTGTGTACCCACAGACCCTAGCCCA
CAAGAAGTAAACTTAACAAATGTGACAGAAAATTTTAACATGTGGAAAAATAACATGGTAGAACAGATGCATG
AGGATATAATCAGTTTATGGGATCAAAGCCTAAAGCCATGTGTAAAATTAACCCCACTCTGTGTCACTTTAAA
TTGCAGTGATGTGACAAATAGTAGTATCACCAATAGTAGTAGTAGTGAGAGTACCAATAATAATAGAACGGTA
GACAAAGAGATGGAGGGAGAAATAAAAAACTGCTCTTTTAATATTACCACAAGTATAACAAATAAGTTGCAGA
AAGAATATGCCCTTTTTTATAAACTTGATGTAGTACCAATAGATAAGAATAATAGTAATAATAAGAATGATAG
TAATAATAGTTATATGTTGATAAATTGTAACACCTCAGTCATTACACAGGCCTGTCCAAAGGTATCCTTTGAA
CCAATTCCCATACATTATTGTGCCCCGGCTGGTTTTGCGATTCTAAAGTGTAAGGATAAGATGTTCAATGGAA
CAGGACCATGCAAAAATGTCAGCACAGTACATTGTACACATGGAATTAGGCCAGTAGTGTCAACTCAATTGCT
GTTAAATGGCAGTCTAGCAGAAGGAGGGGTAGTAGTTAAATCTGAAAATTTCACGAACAATGCTAAAAACATA
ATAGTACAGCTAAACGAATCTGTAGAAATTGAGTGTGAAAGACCCAATAACAATACGAGAAAAGGTATACACA
CAGCATGGGGAAGAGCATTATATGCAACAGGAGAGATAATAGGAAATATAAGAAAAGCATATTGTAACCTTAA
TAAAACAAATTGGGAAAACACTTTAAAACAGATAGTTATCAAATTAGGAGAACAATATGAGAATAAAACAATA
GCCTTTAAGAACTCCTCAGGAGGGGACCCAGAAATTGTAATGCACAGTTTTAATTGTGGAGGGGAATTTTTCT
ACTGTGATACATCACAGCTGTTTAATAGTACTTGGCAGCTTAATAAGACTGATAATACTTATAGTAACAATAA
TACTGAAAAGTTAAATAGCACTGAAGGTGGCAATATCACACTCACATGCAGAATAAAACAAATTATAAACAGA
TGGCAGGAAGTAGGAAAAGCAATGTATGCCCCTCCCATCAGAGGACTAATCAGATGTAGATCAAATATTACAG
GGCTGATATTAACAAGAGATGGAGGCAAGGACAACACAAGCGAGTCTAACACCACTGAGATCTTCAGACCTGG
AGGAGGAGATATGAGGGACAATTGGAGAAGTGAATTATATAAATATAAAGTAGTAAGAATTGAGCCATTAGGA
GTAGCACCCACCAAGGCAAAGAGAAGAGTGGTGCAGAGAGAAAAAAGAGCAGTAGGACTAGGAGCTCTGTTCC
TTGGGTTCTTGGGAGCAGCAGGAAGCACTATGGGCGCAGCGTCAGTGACGCTGACGGTACAGGCCAGACAATT
ATTGTCTGGTATAGTGCAACAGCAGAACAATCTGCTGAGGGCTATTGAGGCGCAACAACATCTGTTGCAACTC
ACAGTCTGGGGCATCAAGCAGCTCCAGGCAAGAGTCCTGGCTGTGGAAACATACCTAAAGGATCAACAGCTCC
TAGGGATTTGGGGTTGCTCTGGAAAACTCATCTGCACCACTGCTGTGCCTTGGAATGCTAGTTGGAGTAATAA
ATCTCTGAGTGACATTTGGGATAACATGACCTGGATGCAGTGGGACAAGGAAATTAACAATTACACAAACTTA
ATATACACCTTACTTGAAAAATCGCAGAACCAACAAGAAAAAAATGAACAAGAACTATTGGAGTTGGATAAGT
GGGCAAGTCTGTGGAATTGGTTTGACATATCAAATTGGCTGTGGTATATAAAAATATTCATAATGATAGTAGG
AGGCTTAGTAGGTTTAAGAATAATTCTTGCTGTGCTTTCTATAGTGAATAGAGTTAGGCAGGGATACTCACCA

CTGTCATTCCAGACCCGCCTCCCAGCCCAGAGGGGACCCGACAGGCCCGAAGGAATCGAAGAAGAAGGTGGAG
AGAGAGACAGAGACAGATCCGTACGATTAGTGACTGGATTCTTAGCACTCATCTGGGACGACCTGCGGAGCCT
GTGCCTCTTCAGCTACCACCACTTGAGAGACTTACTCTTGATTGCAGCGAGGATTGTGGAACTTCTGGGACGC
AGGGGGTGGGAAATCCTCAAGTATTGGTGGAGTCTCCTGCAGTATTGGAGTCAGGAACTAAAGAATAGTGCTG
TTAGCTTGCTTAATGCCACAGCTATCGCAGTAGCTGAGGGAACAGATAGGATTATAGAAGTAGTACAAAGGTT
TGGTAGAGCTATCCTTCACATACCTACAAGAATAAGACAGGGCTTAGAAAGGGCTTTGCTATAAGATGGGTGG
CAAGTGGTCAAAACGTAGCAAGGGTGGTGAATGGCCTGCTGTGAGGGAAAAAATGAAACAAGCTGAGCCAGCA
GCAGAAGGGGTGGGAGCAGCATCTCGAGACTTGGCAAAATATGGAGCACTTACAAGTAGCAATACAGCAACTA
ATAATGCTGCTTGTGCCTGGCTAGAAGCACAAGCACAAGAGGAGGAGGAAGTGGGCTTTCCAGTCAGACCTCA
GGTACCTTTAAGACCAATGACTTACAAGGGAGCGTTCGATCTTAGCTTCTTTTTAAAAGAAAAGGGGGGACTG
GAAGGGCTAATTCACTCCCAAAAGAGACAAGACATCCTTGATCTGTGGATCTACCACACACAAGGCTACTTCC
CTGATTGGCAGAACTACACACCAGGGCCAGGGATCAGATATCCACTGACCTTTGGATGGTGCTTCAAGCTAGT
ACCAGTTGATCCAAAGCAGGTGGAAGAGGCTAATGAAGGAGAGAACAACACCTTGTTACACCCTATGAGCCTG
CATGGGATGGATGATCCAGAGAGAGAAGTGTTAGTGTGGAAGTTTGACAGCCGCCTAGCACGTCATCACATGG
CCAAAGAGCTGCATCCGGAATACTTCAAAGACTGCTGACATCGAGCTTTCTACAAGGGACTTTCCGCTGGGGA
CTTTCCAGGGAGGTGTGGCCTGGGCGGGACTGGGGAGTGGCGAGCCCTCAGATGCTGCATATAAGCAGCTGCT
TTCTGCCTGTACTGGGTCTCTCTGGTTAGACCAGATCTGAGCCTGGGAGCTCTCTGGCTAACTAGGGAACCCA
CTGCTTAAGCCTCAATAAAGCTTGCC

>T-31B HIV-1 genome, derived from RNA genomic sequence
GTGGCGCCCGAACAGGGACTAGAAAGCGAAAGTAAGACCAGAGGAGCTCTCTCGACGCAGGACTCGGCTTGCT
GAAGCGCGCACGGCAAGAGGCGAGGGGCGGCGACTGGTGAGTACGCCAAAAATTAAAATTTTGACTAGCGGAG
GCTAGAAGGAGAGAGATGGGTGCGAGAGCGTCAGTATTAAGCGGGGGAGAATTGGATAGATGGGAAAAAATTC
GGTTACGGCCAGGGGGAAAGAAAAAGTATAAATTAAAACATGTAGTATGGGCAAGCAGGGAACTAGAACGATT
CGCAGTTAACCCTGGCCTTTTAGAGACAGCAGGAGGCTGTAGACAAATATTGGAACAGCTACAACCATCCCTT
CAGACAGGATCAGAAGAACTTAAATCATTATATAATACAATAGCAACCCTCTATTGTGTGCATCAAAAGATAG
ATGTAAAAGACACCAAGGAAGCTTTAGACAAGATAGAGGAAGAGCAAAACAAAAGTAAGAAAAAAGCACAGCA
AGCAGCAGCAGCAGCTGACACAGGAAGCAGCAGCAAGGTCAGCCAAAATTATCCTATAGTGCAAAATCTACAG
GGGCAAATGGTACATCAGCCCATATCACCTAGAACTTTAAATGCATGGGTAAAAGTAGTAGAAGAGAAGGCTT
TCAGTCCAGAGGTAATACCCATGTTTTCAGCATTATCAGAAGGAGCCACCCCACAAGATTTAAACACCATGCT
AAATACAGTGGGGGGACATCAAGCAGCTATGCAAATGTTAAAAGAGACCATCAATGAGGAGGCTGCAGAATGG
GATAGATTACACCCAGTGCATGCAGGGCCTATTGCACCAGGCCAGATGAGAGAACCAAGGGGAAGTGACATAG
CAGGAACTACTAGTACCCTTCAGGAACAAATAGGATGGATGACAAATAATCCACCTATCCCAGTAGGAGAAAT
CTATAAGAGATGGATAATTCTGGGATTAAATAAAATAGTAAGAATGTATAGCCCTACCAGCATTTTGGACATA
AGACAAGGACCAAAAGAACCCTTTAGAGATTATGTAGACAGGTTCTATAAAACGCTAAGAGCAGAGCAAGCCT
CACAGGAGGTAAAAAATTGGATGACAGAAACCTTGTTGGTCCAAAATGCGAACCCAGATTGTAAGACTATCTT
AAAAGCATTAGGACCAGCAGCTACACTAGAAGAAATGATGACAGCATGTCAAGGAGTGGGAGGACCCGGCCAT
AAAGCAAGAGTTTTGGCTGAAGCAATGAGCCAAGTAACAAATTCAGCTACCATAATGATGCAGAAAGGCAATT
TTAGGAGCCAAAGAAAGACTGTTAAGTGTTTCAATTGTGGCAAAGAGGGGCACATAGCCAAAAATTGCAGGGC
TCCTAGGAAAAAGGGCTGTTGGAAATGTGGAAGGGAAGGACACCAAATGAAAGATTGTACTGAGAGACAGGCT
AATTTTTTAGGGAAAATCTGGTCTTCCCACAAGGGAAGGCCAGGGAATTTTCTTCAGAGCAGACCAGAGCCAA
CAGCCCCACCAGCAGAGAGCTTCAGGTTTGGGGAAGAGACAGCAATTCCCCCTCAGAAGCAGGAGCCGATAGA
CAAGGAGCTATATCCTTTAGCTTCCCTCAGATCACTCTTTGGCAACGACCCCTCGTCACAATAAGGATAGGGG
GGCAACTAAAAGAAGCTCTATTAGATACAGGAGCAGATGATACAGTATTAGAAGACATGAGTTTGCCAGGAAG
ATGGAAACCAAAAATGATAGGGGGAATTGGAGGTTTTATCAAAGTAAGACAGTATGATCAGATACCAGTAGAA
ATCTGTGGACATAAAGCTGTAGGTACAGTATTAGTAGGACCCACACCTGTCAACATAATTGGAAGAAATCTGT
TGACTCAGATTGGCTGCACTTTAAATTTTCCCATTAGTCCTATTGAAACTGTACCAGTAAAATTAAAGCCAGG
AATGGATGGCCCAAAAGTTAAACAATGGCCATTGACAGAAGAGAAAATAAAAGCATTAGCAGAAATTTGTACA
GAAATGGAAAGGGAAGGGAAAATTTCAAAAATTGGGCCTGAAAATCCATATAATACTCCAGTATTTGCCATAA
AGAAAAAAGACAGTACTAAATGGAGAAAATTAGTAGACTTCAGAGAACTTAATAAGAGAACTCAAGACTTCTG
GGAAGTTCAATTAGGAATACCACATCCAGCAGGGTTAAAAAAGAAAAAATCAGTAACCGTACTGGATGTGGGT
GATGCATATTTCTCAGTTCCCTTAGATAAAGACTTCAGGAAGTATACTGCATTTACCATACCTAGTATAAACA
ATGAGACACCAGGAATTAGATATCAGTACAATGTGCTTCCACAAGGATGGAAAGGATCACCAGCAATATTCCA
AAGTAGCATGACAAAAATTTTAGAGCCTTTTAGAAAACAAAATCCAGACATAGTTATCTATCAATACATGGAT
GATTTGTATGTAGGATCTGACTTAGAGATAGGGCAACATAGAGCAAAAATAGAGGAACTGAGACAACATCTGT
TGAGGTGGGGATTTACCACACCAGACAAAAAGCATCAGAAAGAACCTCCATTCCTTTGGATGGGTTATGAACT
CCATCCTGATAAATGGACAGTACAGCCTATAGTGCTGCCAGAAAAAGACAGCTGGACTGTCAATGACATACAG
AAGTTAGTGGGAAAATTAAATTGGGCAAGTCAGATTTATGCAGGGATTAAAGTAAAGCAATTATGTAAACTCC
TTAGGGGAACCAAATCACTAACAGAAGTAGTACCACTGACAGAAGAAGCAGAGCTAGAACTGGCAGAAAACAG
GGAGATCCTAAAAGAGCCAGTACATGGAGTATATTATGACCCATCAAAAGACTTAATAGCAGAAATACAGAAG
CAGGGAAATGGCCAATGGACATATCAAATTTATCAAGAGCCATATAAAAATCTGAAAACAGGAAAGTATGCAA
GAATGAGGGGTGCCCACACTAATGATGTAAAACAATTAACAGAGGCAGTGCAAAAAATAGCCAATGAAAGCAT
AGTAATATGGGGAAAGATTCCTAAATTTAAATTACCCATACAGAAAGAAACATGGGAAGCATGGTGGATGGAG
TATTGGCAAGCCACCTGGATTCCTGAGTGGGAGTTTGTCAATACCCCTCCCTTAGTGAAATTATGGTATCAGT
TAGAGAAAGAGCCCATAGTAGGAGCAGAAACTTTCTATGTAGATGGGGCAGCTAATAGGGAAACTAAATTGGG
AAAAGCAGGATATGTTACTGACAGAGGAAAACAAAAAGTTGTCTCCCTAACAGACACAACAAATCAGAAGACT
GAGTTACAAGCAATTTATCTAGCTTTGCAGGATTCGGGATTAGAAGTAAACATAGTAACAGACTCACAATATG
CATTAGGAATCATTCAAGCACAACCAGATAAGAGTGAGTCAGAGTTAGTCAATCAAATAATAGAGCAGTTAAT
AAAAAAGGAAAAGGTCTACCTGGCATGGGTACCAGCACACAAAGGAATTGGAGGAAATGAACAAGTAGATAAA
TTAGTCAGTGCTGGAATTAGGAAAGTACTATTTTTGGATGGAATAGATAAGGCCCAAGATGAACATGAGAAAT
ATCACAATAATTGGAGAGCAATGGCTAGTGATTTTAACCTGCCACCTATAGTAGCAAAAGAAATAGTAGCCAG
CTGTGATAAATGTCAGCTAAAAGGAGAAGCCATGCATGGACAAGTAGACTGTAGTCCAGGAATATGGCAGCTA
GATTGTACACACTTAGAAGGAAAAGTTATCCTGGTAGCAGTTCATGTGGCCAGTGGATATATAGAAGCAGAAG

TTATTCCAGCAGAAACAGGGCAAGAAACAGCATACTTTATCTTAAAATTAGCAGGAAGATGGCCAGTAAAAAT
TATACACACAGACAATGGCCCCAATTTCATCAGTACTGCGGTTAAGGCCGCCTGTTGGTGGGCAGGGATCAAG
CAGGAATTTGGTATTCCCTACAATCCCCAAAGTCAAGGAGTAGTAGAATCTATGAATAATGAATTAAAGAAAA
TTATAGGACAGGTAAGAGATCAAGCTGAACATCTTAAGACAGCAGTACAAATGGCAGTATTCATCCACAATTT
TAAAAGAAAAGGGGGGATTGGGGGGTACAGTGCAGGGGAAAGAATAGTAGACATAATATCATCAGACATACAA
ACTAAAGAATTACAAAAACAGATTACAAAAATTTCAAAATTTCGGGTTTATTACAGGGACAGCAGAGATCCAC
TTTGGAAAGGACCAGCAAAGCTTCTCTGGAAAGGTGAAGGGGCAGTAGTAATACAAGATAATAGTGACATAAA
AGTAGTGCCAAGAAGAAAAGCAAAGATCATTAGGGATTATGGAAAACAGATGGCAGGTGATGATTGTGTGGCA
AGTAGACAGGATGAGGATTAGATCATGGAAAAGTTTAGTAAAACATCATATGTATATTTCAGGAAAAGCTAGG
AAATGGTTTTATAGACATCACTATGAAAGCACTCATCCAAGAATAAGTTCAGAAGTTCACATCCCACTAGGGG
ATGCTAAATTAGTAGTAACAACATATTGGGGTCTGCATACAGGAGAAAGAGATTGGCATTTGGGTCAGGGAGT
CTCCATAGAATGGAGGAAAAGGAGATATAGCACACAAGTAGACCCTGACCTAGCAGACCAACTAATTCATTCG
TATTATTTTGATTGTTTTTCAGAATCTGCTATAAGAAATGCCATATTAGGACATATAGTTAGACCTAGGTGTG
AGTATCAAGCAGGACATAACAAGGTAGGATCTCTACAGTACTTGGCACTAACAGCATTAATAACACCAAAAAA
GATAAAGCCACCTTTGCCTAGTGTTAAGAAACTGACAGAGGATAGATGGAACAAGCCCCAGAGGACCAAGGGC
CACAGAGGGAGCCATACAATGAATGGACACTAGAGCTTTTAGAGGAACTGAAAAATGAAGCTGTTAGACATTT
TCCTAGGGCATGGCTCCATAGCTTAGGACAATATATCTATGAAACTTATGGGGATACTTGGGCAGGAGTGGAA
GCCTTATTAAGAATTCTGCAACAAATGCTGTTTATTCATTTCAGAATTGGATGTCACCATAGCAGAATAGGCA
TTATTCTACAGAGGAGAACAAGAAATGGAGCCAGTAGATCCTAGACTAGAGCCCTGGAAGCATCCAGGAAGTC
AGCCTAAAACTGCTTGTACCAAATGCTATTGTAAAAAGTGTTGCTTGCATTGCCAAGTTTGCTTCATAACAAA
AGGCTTAGGCATCTCCTATGGCAGGAAGAAGCGGAGACAGCGACGAAGACCTCCTCAAAACAGTGAGGCTCAT
CAAGTTTCTCTATCAAAGCAGTAAGTAGTACATGTAATGCAACCTCTACAAATAGCAGCAATAGTATCATTAG
TAGTAGTAGCAATAATAGCAATAGTTGTGTGGACCATAGTGTTTATAGAATATAGGAAAATATTAAGACAAAG
AAAAATAGACAGGATACTTGATAGGATAAGGGACAGAGCAGAAGACAGTGGCAATGAGAGCGAGGGAGATCAG
GAAGAATTGTCAGCTCTTGTTGACATGGGGCACGATGCTCCTTGGAATGTTGATGATCTGTAGTGCTACAGAA
AAATTGTGGGTTACAGTGTATTATGGGGTACCTGTGTGGAAAGAAGCAACCACCACTCTATTTTGTGCATCAG
ATGCTAAAGCATATGACACAGAAGCACATAATGTTTGGGCCACACATGCCTGTGTACCCACAGACCCTAGCCC
ACAAGAAGTAAACTTGACAAATGTGACAGAAAATTTTAACATGTGGAAAAATAACATGGTAGAACAGATGCAT
GAGGATATAATCAGTTTATGGGATCAAAGTCTAAAGCCATGTGTAAAATTAACCCCACTCTGTGTCACTTTAA
ATTGCACTGATGTGACGGATGGTACTACTGGGAACAGAACGGTAGACACAGAGATGGAGGGAGAAATAAAAAA
CTGCTCTTTCAATATTACCACAAGTATAACAAATAAGTTGCAGAAAGAATATGCCCTTTTTTATAAACTTGAT
GTAGTACCAATAGATAAGAATGATAGAAATAATAGTAATGATCAGAATAATAGTTATAGTAGTTATATGTTGA
TAAATTGTAACACCTCAGTCATTACACAGGCCTGTCCAAAGGTATCCTTTGAACCAATTCCCATACATTATTG
TGCCCCGGCTGGTTTTGCGATTCTAAAGTGTAAGGATAAGATGTTCAATGGAACAGGACCATGCAAAAATGTC
AGCACAGTACATTGTACACATGGAATTAGGCCAGTAGTGTCAACTCAATTGCTGTTAAATGGCAGTCTAGCAG
AAGGAGGGGTAGTAATTAAATCTGAAAACTTCTCGGACAATGCTAAGAACATAATAGTACAGCTGAACGAATC
TGTAGAAATTAATTGTACAAAACCCAATAAGTATATAAAAAAACAAGGTATATACTCAGTACGGGGAAGAACA
TTATATGCAACAGGAGAAATAACAGGAAATATAAAAAAAGCACATTGTAACCTTAGTGAGACAAATTGGGAAA
ACACTTTAAAACAGATAGCTATAAAATTAGGAGAACAATATGGGAGGAATAAAACAATAGCCTTTAGGAACTC
CTCAGGAGGGGACCCAGAAATTGTAATGCACAGTTTTAATTGTGGAGGGGAATTTTTCTACTGTGATACAACA
CAGCTGTTTAATAGTACTTGGCAGCCTAATAGTACTGAGAAGACTTATAGTAGTAATGATACTAAAGGGTCAA
ATGGCACTATCACACTCACATGCAGAATAAAACAAATTATAAACAGGTGGCAGGAAGTAGGAAAAGCAATGTA
TGCCCCTCCCATCGAAGGACTAATTAAATGTAGCTCAAATATTACAGGGCTGATATTAACAAGAGATGGAGGC
AACGAAAGCCAGTCTAACCCTGAGACCTTCAGACCTGCAGGGGGAGATATGAAGGACAATTGGAGAAGTGAAT
TATATAAATATAAAGTAGTAAGAATTGAGCCATTAGGAGTAGCACCCACCGAGGCAAAGAGAAGAGTGGTGCA
GAGAGAAAAAAGAGCAGTGGGACTAGGAGTGATGTTCCTTGGGTTCTTGGGAGCAGCAGGAAGCGCTATGGGC
GCAGCGTCAGTGACGCTGACGGTACAGGCCAGACAATTATTGTCTGGTATAGTGCAACAGCAGAACAATCTGC
TGAGGGCTATTGAGGCGCAACAGCATATGTTGCAACTCACAGTCTGGGGCATTAAGCAGCTCCAGGCAAGAGT
CCTGGCTGTGGAAACATACCTAAAGGATCAACAGCTCCTAGGGATTTGGGGTTGCTCTGGAAAACTCATCTGC
ACCACTGCTGTGCCTTGGAATGCTAGTTGGAGTAATAAATCTCTGAGTGACATTTGGAATAACACGACCTGGA
TGCAGTGGGACAAGGAAATTAACAATTACACAAGCTTAATATACACCTTACTTGAAGAATCGCAGTACCAACA
AGAAAAAAATGAACAAGAACTATTGGAATTGGATAAGTGGGCAAGTTTATGGAATTGGTTTGACATATCAAAT
TGGCTGTGGTATATAAAAATATTCATAATGATAGTAGGAGGCTTAGTAGGTTTAAGAATAGTTTTTGCTGTGC
TTTCTATAGTGAATAGAGTTAGGCAGGGATACTCACCATTGTCATTCCAGACCCGCCTCCCAGCTCAGAGGGG

ACCCGACAGGCCCGAAGGAATCGAAGAAGAAGGTGGAGAGAGAGACAGAGGCAGATCCGAACGATTAGTGACT
GGATTCTTAGCACTCATCTGGGACGACCTGCGGAGCCTGTGCCTCTTCAGCTACCACCGCTTGAGAGACTTAA
TCTTGATTGCAGCGAGGATTCTGGAACTTCTGGGACGCAGGGGGTGGGAAATCCTCAAATATTGGTGGAGTCT
CCTGCAGTATTGGAGTCAGGAACTAAAGAATAGTGCTGTTAGCTTGCTTAATGTCACAGCTATTGCAGTAGCT
GAGGGAACAGATAGGATTATAGAAGTAGTACAAAGGTTTGGTAGAGCTATCCTTCACATACCTACAAGAATAA
GACAGGGCTTAGAAAGGGCTTTGCTATAAAATGGGTGGCAAGTGGTCAAAACGTAGTAAGGGTGGTGAATGGC
CTGCTGTGAGGGAAAAAATGAAACAAGCTGAGCCAGCAGCAGAAGGGGTGGGAGCAGCATCTCGAGACTTGGC
AAAATATGGAGCACTCACAAGTAGCAATACAACAACTAATAATGCTGCTTGTGCCTGGCTAGAAGCACAAGAG
GAGGAGGAAGTGGGCTTTCCAGTCAGACCTCAGGTACCTTTAAGACCAATGACTTACAAGGCAGCGTTTGATC
TTGGCTTCTTTTTAAAAGAAAAGGGGGGACTGGAAGGGCTAATTTACTCCCAAAAGAGACAAGACATCCTTGA
TTTGTGGGTCTACCACACACAAGGCTACTTCCCTGACTGGCAGAACTACACACCAGGGCCAGGGGTCAGATAT
CCACTGACCTTTGGATGGTGCTTCAAGCTAGTACCAGTTGAGCCAAAGCAGGTAGAAGAGGCCAATGAAGGAG
ATAACATCAACTTGTTACACCCTATGAGCCAGCATGGGATGGATGATCCAGAGAGAGAAGTGTTAGTGTGGAA
GTTTGACAGCCGCCTAGCATTGCATCACATGGCCAAAGAGCTGCATCCGGAGTACTTCAAGGACTGCTGACAT
CGAGCTTTCTACAAGGGACTTTCCACTGGGGACTTTCCAGGGAGGTGTGGCCTGGGCGGGACAGGGGAGTGGC
GAGCCCTCAGATGCTGCATATAAGCAGCTGCTTTTTGCCTGTACTGGGTCTCTCTGGTTAGACCAGATCTGAG
CCTGGGAGCTCTCTGGCTGACTAGGGAACCCACTGCTTAAGCCTCAATAAAGCTTGCC

>T-32D HIV-1 genome, derived from RNA genomic sequence
GTGGCGCCCGAACAGGGACTAGAAAGCGAAAGTAAGACCAGAGGAGCTCTCTCGACGCAGGACTCGGCTTGCT
GAAGCGCGCACGGCAAGAGGCGAGGGGCGGCGACTGGTGAGTACGCAAAAAATTAAAATTTTGACTAGCGGAG
GCTAGAAGGAGAGAGATGGGTGCGAGAGCGTCAGTATTAAGCGGGGGAGAATTGGATAGATGGGAAAAAATTC
GGTTACGGCCAGGGGGAAAGAAAAAGTATAAATTAAAACATGTAGTATGGGCAAGCAGGGAACTAGAACGATT
CGCAGTTAACCCTGGCCTTTTAGAGACAGCAGGAGGCTGTAGACAAATATTGGAACAGCTACAACCATCCCTT
CAGACAGGATCAGAAGAACTTAAATCATTATATAATACAATAGCAACCCTCTATTGTGTGCATCAAAAGATAG
ATGTAAAAGACACCAAGGAAGCTTTAGACAAGATAGAGGAAGAGCAAAACAAAAGTAAGAAAAAGGCACAGCA
AGCAGCAGCAGCAGCTGACACAGGAAGCAGCAGCAAGGTCAGCCAAAATTATCCTATAGTGCAAAATCTACAG
GGGCAAATGGTACATCAGCCCATATCACCTAGAACTTTAAATGCATGGGTAAAAGTAGTAGAAGAGAAGGCTT
TCAGTCCAGAAGTAATACCCATGTTTTCAGCATTATCAGAAGGAGCCACCCCACAAGATTTAAACACCATGCT
AAATACAGTGGGGGGACATCAAGCAGCCATGCAAATGTTAAAAGAGACCATCAATGAGGAAGCTGCAGAATGG
GATAGATTACACCCAGTGCATGCAGGGCCTATTGCACCAGGCCAGATGAGAGAACCAAGGGGAAGTGACATAG
CGGGAACTACTAGTACCCTTCAAGAACAAATAGGATGGATGACAAATAATCCACCTATCCCAGTAGGAGAAAT
ATATAAAAGATGGATAATTCTGGGATTAAATAAAATAGTAAGAATGTATAGCCCTACCAGCATTTTGGACATA
AGACAAGGACCAAAAGAACCCTTTAGAGATTATGTAGATAGGTTCTATAAAACTCTAAGAGCAGAGCAAGCCT
CACAGGAGGTAAAAAATTGGATGACAGAAACCTTGTTGGTCCAAAATGCGAACCCAGATTGTAAGACTATCTT
AAAAGCATTAGGACCAGCAGCTACACTAGAAGAAATGATGACAGCATGTCAGGGAGTGGGGGGACCCGGCCAT
AAAGCAAGAGTTTTGGCTGAAGCAATGAGCCAAGTAACAAATTCAGCTGCCATAATGATGCAGAAAGGCAATT
TTAGGAGCCAAAGAAAGACTGTTAAGTGTTTCAATTGTGGCAAAGAGGGGCACATAGCCAAAAATTGCAGGGC
TCCTAGGAAAAAGGGCTGTTGGAAATGTGGAAGGGAAGGACACCAAATGAAAGATTGTACTGAGAGACAGGCT
AATTTTTTAGGGAAAATCTGGTCTTCCCACAAGGGAAGGCCAGGGAATTTTCCTCAGAGCAGACCAGAGCCAA
CAGCCCCACCAGCAGAGAGCTTCAGATTTGGGGAGGAGACAGCAACTCCCCCTCAGAAGCAGGAGCCGACAGA
CAAGGAGCTATATCCTTTAACCTCCCTCAGATCACTCTTTGGCAACGACCCCTCGTCACAATAAGGATAGGGG
GGCAACTAAAAGAAGCTCTATTAGATACAGGAGCAGATGATACAGTATTAGAAGAAATGAGTTTGCCAGGAAG
ATGGAAACCAAAAATGATAGGGGGAATTGGAGGTTTTATCAAAGTAAGACAGTATGATCAGATACCAGTAGAA
ATCTGTGGGCATAAAGCTGTAGGTACAGTATTAGTAGGACCTACACCTGTCAACATAATTGGAAGAAATCTGT
TGACTCAGATTGGCTGCACTTTAAATTTTCCTATTAGTCCTATTGAAACTGTACCAGTAAAATTAAAGCCAGG
AATGGATGGCCCAAAAGTTAAACAATGGCCATTGACAGAAGAGAAAATAAAAGCATTAGTAGAAATTTGTACA
GAAATGGAAAAGGAAGGAAAAATTTCAAAAATTGGGCCTGAAAACCCATACAATACTCCAGTATTTGCCATAA
AGAAAAAAGACAGTACTAAATGGAGAAAATTAGTAGATTTCAGAGAACTTAATAAGAGAACTCAAGACTTCTG
GGAAGTTCAATTAGGAATACCACATCCAGCAGGGTTAAAAAAGAAAAAATCAGTAACCGTACTGGATGTGGGT
GATGCATATTTCTCAGTTCCCTTAGATAAAGACTTCAGGAAGTATACTGCATTTACCATACCTAGTATAAACA
ATGAGACACCAGGAATTAGATATCAGTACAATGTGCTTCCACAAGGATGGAAAGGATCACCAGCAATATTCCA
AAGTAGCATGACAAAAATTTTAGAGCCTTTTAGAAAACAAAATCCAGACATAGTTATCTATCAATACATGGAT
GATTTGTATGTAGGATCTGACTTAGAAATAGGGCAACATAGAGCAAAAATAGAGGAACTGAGACAACATCTGT
TGAGGTGGGGATTTACCACACCAGACAAAAAGCATCAGAAAGAACCTCCATTCCTTTGGATGGGTTATGAACT
CCATCCTGATAAATGGACAGTACAGCCTATAGTGCTGCCAGAAAAAGACAGCTGGACTGTCAATGACATACAG
AAGTTAGTGGGAAAATTAAATTGGGCAAGTCAGATTTATGCAGGGATTAAAGTAAAGCAATTATGTAAACTCC
TTAGGGGAACCAAATCACTAACAGAAGTAGTACCACTGACAGAAGAAGCAGAGCTAGAACTGGCAGAAAACAG
GGAGATTCTAAAAGAACCAGTACATGGAGTGTATTATGACCCATCAAAAGACTTAATAGCAGAAATACAGAAG
CAGGAGAATGGTCAGTGGACATATCAAATTTATCAGGAGCCACATAAGAATCTGAAAACAGGAAAGTATGCAA
GAATGAGGGGTGCCCACACTAATGATGTAAGACAATTAACAGAGGCAGTGCAAAAAATAGCCAATGAAAGCAT
AGTAATATGGGGAAAGATTCCTAAGTTTAAATTACCCATACAGAAAGAAACATGGGAAGCATGGTGGATGGAG
TATTGGCAGGCCACCTGGATTCCTGAGTGGGAGTTTGTCAATACCCCTCCCTTAGTGAAATTATGGTATCAGT
TAGAGAAAGAGCCCATAGTAGGAGCAGAAACTTTCTATGTAGATGGGGCAGCTAATAGGGAAACTAAATTGGG
AAAAGCAGGATATGTTACTGACAGAGGAAGACAAAAAGTTGTCTCCCTAACAGATACAACAAATCAGAAGACT
GAGTTACAAGCAATTTATCTAGCTTTGCAGGATTCGGGATCAGAAGTAAATATAGTAACAGACTCACAATATT
CATTAGGAATCATTCAAGCACAACCAGATAAGAGTGAATCAGAGTTAGTTAATCAAATAATAGAGCAGTTAAT
AAAAAAGGAAAAGGTTTACCTGGCATGGGTACCAGCACACAAAGGAATTGGAGGAAATGAACAAGTAGATAAA
TTAGTCAGTGCTGGAATTAGGAAAGTACTATTTTTGGATGGAATAGATAAGGCCCAAGATGAACATGAGAAAT
ATCACAATAATTGGAGAGCAATGGCTAGTGATTTTAACCTGCCACCTATAGTAGCAAAAGAAATAGTAGCCAG
CTGTGATAAATGTCAGCTAAAAGGAGAAGCCATGCATGGACAAGTAGACTGTAGTCCAGGAATATGGCAGCTA
GATTGTACACACTTAGAAGGAAAAGTTATCCTGGTAGCAGTTCATGTGGCCAGTGGATATATAGAAGCAGAAG

TTATTCCAGCAGAAACAGGGCAAGAAACAGCATACTTTATCTTAAAATTAGCAGGAAGATGGCCAGTAAAAAT
TATACACACAGACAATGGCCCCAATTTCATCAGTACTGCGGTTAAGGCCGCCTGTTGGTGGGCAGGGATCAAG
CAGGAATTTGGTATTCCCTACAATCCCCAAAGTCAAGGAGTAGTAGAATCTATGAATAATGAATTAAAGAAAA
TTATAGGACAGGTAAGAGATCAAGCTGAACATCTTAAGACAGCAGTACAAATGGCAGTATTCATCCACAATTT
TAAAAGAAAAGGGGGGATTGGGGGGTACAGTGCAGGGGAAAGAATAGTAGACATAATATCATCAGACATACAA
ACTAAAGAATTACAAAAACAGATTACAAAAATTTCAAAATTTCGGGTTTATTACAGGGACAGCAGAGATCCAC
TTTGGAAAGGACCAGCAAAGCTTCTCTGGAAAGGTGAAGGGGCAGTAGTAATACAAGATAATAGTGACATAAA
AGTAGTGCCAAGAAGAAAAGCAAAGATCATTAGGGATTATGGAAAACAGATGGCAGGTGATGATTGTGTGGCA
AGTAGACAGGATGAGGATTAGATCATGGAAAAGTTTAGTAAAACATCATATGTATATTTCAGGAAAAGCTAGG
AAATGGTTTTATAGACATCACTATGAAAGCACTCATCCAAGAATAAGTTCAGAAGTTCACATCCCACTAGGGG
ATGCTAAATTAGTAGTAACAACATATTGGGGTCTGCATACAGGAGAAAGAGATTGGCATTTGGGTCAGGGAGT
CTCCATAGAATGGAGGAAAAGGAGATATAGCACACAAGTAGACCCTGACCTAGCAGACCAACTAATTCATTCG
TATTATTTTGATTGTTTTTCAGAATCTGCTATAAGAAATGCCATATTAGGACATATAGTTAGACCTAGGTGTG
AGTATCAAGCAGGACATAACAAGGTAGGATCTCTACAGTACTTGGCACTAACAGCATTAATAACACCAAAAAA
GATAAAGCCACCTTTGCCTAGTGTTAAGAAACTGACAGAGGATAGATGGAACAAGCCCCAGAGGACCAAGGGC
CACAGAGGGAGCCATACAATGAATGGACACTAGAGCTTTTAGAGGAACTGAAAAATGAAGCTGTTAGACATTT
TCCTAGGGCATGGCTCCATAGCTTAGGACAATATATCTATGAAACTTATGGGGATACTTGGGCAGGAGTGGAA
GCCTTATTAAGAATTCTGCAACAAATGCTGTTTATTCATTTCAGAATTGGATGTCACCATAGCAGAATAGGCA
TTATTCTACAGAGGAGAACAAGAAATGGAGCCAGTAGATCCTAGACTAGAGCCCTGGAAGCATCCAGGAAGTC
AGCCTAAAACTGCTTGTACCAAATGCTATTGTAAAAAGTGTTGCTTGCATTGCCAAGTTTGCTTCATAACAAA
AGGCTTAGGCATCTCCTATGGCAGGAAGAAGCGGAGACAGCGACGAAGACCTCCTCAAAACAGTGAGGCTCAT
CAAGTTTCTCTATCAAAGCAGTAAGTAGTACATGTAATGCAACCTTTACAAATAGCAGCAATAGTATCATTAG
TAGTAGTAGCAATAATAGCAATAGTTGTGTGGACCATAGTGTTTATAGAATATAGGAAAATATTAAGACAAAG
AAAAATAGACAGGTTACTTGATAGGATAAGGGACAGAGCAGAAGACAGTGGCAATGAGAGCGAAGGAGATCAG
GAAGAATTGTCAGCTCTTGTTGACATGGGGCACGATGCTCCTTGGAATGTTGATGATCTGTAGTGCTACAGAA
AAATTGTGGGTTACAGTGTATTATGGGGTACCTGTGTGGAAAGAAGCAACCACCACTCTATTTTGTGCATCAG
ATGCTAAAGCATATGACACAGAAGCACATAATGTTTGGGCCACACATGCCTGTGTACCCACAGACCCTAGCCC
ACAAGAAGTAGACTTGACAAATGTGACAGAAAATTTTAACATGTGGAAAAATAACATGGTAGAACAGATGCAT
GAGGATATAATCAGTTTATGGGATCAAAGTCTAAAGCCATGTGTAAAATTAACCCCACTCTGTGTCACTTTAA
ATTGCACTGATGTGACGAATGGTACTGATGTGACGAATGGTACTACTGGGAACAGAACGGTAGACACAGAAAT
GGAGGGAGAAATAAAAAACTGCTCTTTCAATATTACCACAAGTATAACAAATAAGTTGCAGAAAGAATATGCC
CTTTTTTATAAACTTGATGTAGTGCCAATAGATGAGAATGATAGTAATAATGGTAGTAATAGTCATAAGAATA
ATAATTATAGTAGTTATATGCTGATAAATTGTAACACCTCAGTCATTACACAAGCCTGTCCAAAGGTATCCTT
TGAACCAATTCCCATACATTATTGTGCCCCGGCTGGTTTTGCGATTCTAAAGTGTAAGGATAAGATGTTCAAT
GGAACAGGACCATGCAAAAATGTCAGCACAGTACATTGTACACATGGAATTAGGCCAGTAGTGTCAACTCAAT
TGCTGTTAAATGGCAGTCTAGCAGAAGGAGGGGTAGTAATTAAATCTGAAAACTTCTCGGACAATGCTAAGAA
CATAATAGTACAGCTGAACGAATCTGTAGAAATTAATTGTACAAAACCCAATAAGTATATAAAAAAACAAGGT
ATATACTCAGTACGGGGAAGAACATTATATGCAACAGGAGAGATAACAGGAAATATAAAAAAGGCACATTGTA
ACCTTAGTGTAACAAATTGGGAAGACACTTTAAAACAGATAGCTATAAAATTAGGGGAACAATATGGGAGGAA
TAAAACAATAGCCTTTAGGAGCTCCTCAGGAGGGGACCCAGAAATTGTAATGCACAGTTTTAATTGTGGAGGG
GAATTTTTCTACTGTGATACATCACAGCTGTTTAATAGTACGTGGCAGCTTAATAGTACTGCAAATACTAATA
CTTATAGTAGTAATGATACTAAAGGGTCAAATGGCACTATCACACTCACATGCAGAATAAAACAAATTATAAA
CAGGTGGCAGGAAGTAGGAAAAGCAATGTATGCCCCTCCCATTAAAGGACAAATTAACTGTACATCAAATATT
ACAGGGCTGATATTAACAAGAGATGGAGGCAATAGCAACAAAAATGGGTCTGAAGGGGTTGACAACACAACTG
AGACCTTCAGACCTGCAGGGGGAGATATGAAGGACAATTGGAGAAGTGAATTATATAAATATAAAGTAGTAAG
AATTGAGCCATTAGGAGTAGCACCCACCGAGGCAAAGAGAAGAGTGGTGCAGAGAGAAAAAAGAGCAGTGGGA
CTAGGAGTGATGTTCCTTGGGTTCTTGGGAGCAGCAGGAAGCGCTATGGGCGCAGCGTCAGTGACGCTGACGG
TACAGGCCAGACAATTATTGTCTGGTATAGTGCAACAGCAGAACAATCTGCTGAGGGCTATTGAGGCGCAACA
GCATATGTTGCAACTCACAGTCTGGGGCATTAAGCAGCTCCAGGCAAGAGTCCTGGCTGTGGAAACATACCTA
AAGGATCAACAGCTCCTAGGGATTTGGGGTTGCTCTGGAAAACTCATCTGCACCACTGCTGTGCCTTGGAATG
CTAGTTGGAGTAATAAATCTCTGAGTGACATTTGGAATAACACGACCTGGATGCAGTGGGACAAGGAAATTAA
CAATTACACAAGCCTAATATACACCTTACTTGAAGAATCGCAGTACCAACAAGAAAAAAATGAACAAGAACTA
TTGGAATTGGATAAGTGGGCAAGTTTATGGAATTGGTTTGACATATCAAATTGGCTGTGGTATATAAAAATAT
TCATAATGATAGTAGGAGGCTTAGTAGGTTTAAGAATAGTTTTTGCTGTGCTTTCTATAGTGAATAGAGTTAG

GCAGGGATACTCACCATTGTCATTCCAGACCCGCCTCCCAGCTCAGAGGGGACCCGACAGGCCCGAAGGAATC
GAAGAAGAAGGTGGAGAGAGAGACAGAGGCAGATCCGAACGATTAGTGACTGGATTCTTAGCACTCATCTGGG
ACGACCTGCGGAGCCTGTGCCTCTTCAGCTACCACCGCTTGAGAGACTTAATCTTGATTGCAGCGAGGATTCT
GGAACTTCTGGGACGCAGGGGGTGGGAAATCCTCAAATATTGGTGGAGTCTCCTGCAGTATTGGAGTCAGGAA
CTAAAGAATAGTGCTGTTAGCTTGCTTAATGTCACAGCTATTGCAGTAGCTGAGGGAACAGATAGGATTATAG
AAGTAGTACAAAGGTTTGGTAGAGCTATCCTTCACATACCTACAAGAATAAGACAGGGCTTAGAAAGGGCTTT
GCTATAAAATGGGTGGCAAGTGGTCAAAACGTAGTAAGGGTGGTGAATGGCCTGCTGTGAGGGAAAAAATGAA
ACAAGCTGAGCCAGCAGCAGAAGGGGTGGGAGCAGCATCTCGAGACTTGGCAAAATATGGAGCACTCACAAGT
AGCAATACAACAACTAATAATGCTGCTTGTGCCTGGCTAGAAGCACAAGAGGAGGAGGAAGTGGGCTTTCCAG
TCAGACCTCAGGTACCTTTAAGACCAATGACTTACAAGGCAGCGTTTGATCTTGGCTTCTTTTTAAAAGAAAA
GGGGGGACTGGAAGGGCTAATTCACTCCCAAAAGAGACAAGACATCCTTGATCTGTGGGTTTACCACACACAA
GGCTACTTCCCTGATTGGCAGAACTACACACCAGGGCCAGGGGTCAGATATCCACTGACCTTTGGATGGTGCT
TCAAGCTAGTACCAGTTGAGCCAAAGCAGGTAGAAGAGGCCAATGAAGGAGATAACATCAACTTGTTACACCC
TATGAGCCAGCATGGGATGGATGATCCAGAGAGAGAAGTGTTAGTGTGGAAGTTTGACAGCCGCCTAGCATTG
CATCACATGGCCAAAGAGCTGCATCCGGAGTACTTCAAGGACTGCTGACATCGAGCTTTCTACAAGGGACTTT
CCACTGGGGACTTTCCAGGGAGGTGTGGCCTGGGCGGGACAGGGGAGTGGCGAGCCCTCAGATGTTGCATATA
AGCAGCTGCTTTTTGCCTGTACTGGGTCTCTCTGGTTAGACCAGATCTGAGCCTGGGAGCTCTCTGGCTAGCT
AGGGAACCCACTGCTTAAGCCTCAATAAAGCTTGCC

>V-A6 HIV-1 genome, derived from RNA genomic sequence
AGCAGTGGCGCCCGAACAGGGACTTGAAAGCGAAAGTAGAACCAGAGGAGATCTCTCGACGCAGGACTCGGCT
TGCTGAAGCGCGCACAGCAAGAGGCGAGAGGCGGCGACTGGTGAGTACGCCAAAACTATAATTTTGACTAGCG
GAGGCTAGAAGGAGAGAGATGGGTGCGAGAGCGTCAGTATTAAGCGGGGGAAAATTAGACAAATGGGAAAAAA
TTCGGTTAAGGCCAGGGGGAAAGAAAACATATAAATTAAAACATATAGTATGGGCAAGCAGGGAGCTAGAACG
ATTCGCAGTTAATCCTGGCCTTTTAGAAACATCAGAAGGCTGTAGACAGATCCTGGGACAGCTACAACCATCC
CTTCAGACAGGATCAGAAGAACTTAAATCATTATATAATGCAGTAGCAACCCTCTATTGTGTGCATCAAAATA
TAGATGTAAGAGACACCAAGGAAGCTTTAGACAAGATAGAGGAAGAGCAAAACAAAAGTAAGAAAAGGGCACA
GCAAGTAGCAGCTGACACAGAAAACAGCAGCAAGGTCAGCCAAAATTATCCTATAGTGCAGAACCTTCAGGGG
CAAATGGTACATCAGGCACTATCACCTAGAACCTTAAATGCATGGGTAAAGGTAATAGAAGAGAAGGCTTTCA
GCCCAGAAGTAATACCCATGTTTTCAGCATTATCAGAAGGAGCCACCCCACAAGATTTAAACACCATGTTAAA
CACAGTGGGGGGACATCAAGCAGCTATGCAAATGTTAAAAGAGACCATCAATGAGGAAGCTGCAGAATGGGAT
AGATTGCATCCAGTGCATGCAGGGCCTATTGCACCAGGCCAGATGAGAGAACCAAGGGGAAGTGACATAGCAG
GAACTACTAGTACCCTTCAGGAACAAATAGGATGGATGACACATAATCCACCTATCCCAGTAGGAGAAATCTA
TAAAAGATGGATAATCATGGGATTAAATAAAATAGTAAGAATGTATAGCCCTATCAGCATTCTGGACATAAGA
CAAGGACCAAAAGAACCCTTTAGAGACTATGTAGACCGGTTCTATAAAACTCTAAGAGCCGAGCAAGCTTCAC
AGGAGGTAAAAAATTGGATGACAGAAACTTTGTTGGTCCAAAATGCGAACCCAGATTGTAAGACTATTTTAAA
AGCATTAGGGCCAGGAGCTACATTAGAAGAAATGATGACAGCATGTCAGGGAGTGGGGGGACCCGGCCATAAA
GCAAGAGTTTTGGCTGAAGCAATGAGCCAAGTAACAAATTCAGCTACCATAATGATGCAGAAAGGCAATTTTA
GGAACCAAAGAAAAACTGTTAAGTGTTTCAATTGTGGCAAAGAAGGGCACATAGCCAGAAATTGCAGGGCCCC
TAGGAAAAGGGGCTGTTGGAAATGTGGAAAGGAAGGACACCAAATGAAAGATTGTACTGAGAGACAGGCTAAT
TTTTTAGGGAAGATCTGGCCTTCCCACAAGGGAAGGCCAGGGAATTTCCTTCAGAGCAGACCAGAGCCAACAG
CCCCACCAGCAGAGAGCTTCAGGTTTGGGGAAGAGACAACGAATCCCCCTCAGAAGCAGGAGCCGATAGACAA
GGAAGTATATCCTTTAACCTCCCTCAGATCACTCTTTGGCAACGACCCCTCGTCACAGTAAAGATAGGGGGGC
AACTAAAGGAAGCTCTATTAGATACAGGAGCAGATGATACAGTACTAGAAGAAATAAGTTTGCCAGGAAGATG
GAAACCAAAAATGATAGGGGGAATTGGAGGTTTTATCAAAGTAAGACAGTATGATCAGATACCCATAGAAATC
TGTGGGCATAAAGCTATAGGTACAGTATTAGTAGGACCTACACCTGTCAACATAATTGGAAGAAATCTGTTGA
CTCAGATTGGCTGCACTTTAAATTTTCCCATTAGTCCTATTGAAACTGTACCAGTAAAATTAAAGCCAGGAAT
GGATGGCCCAAGAGTTAAACAATGGCCATTGACAGAAGAAAAAATAAAAGCATTAGTAGAAATTTGTACAGAG
ATGGAAAAGGAAGGAAAAATTTCAAAAATTGGGCCTGAAAATCCATACAATACTCCAGTATTTGCCATAAAGA
AAAAAGACAGTACTAAATGGAGAAAATTAGTAGATTTCAGAGAACTTAATAAGAGAACTCAAGACTTCTGGGA
AGTTCAATTAGGAATACCACATCCCGCAGGGTTAAAAAAGAAAAAATCAGTAACAGTACTGGATGTGGGTGAT
GCATATTTTTCAGTTCCCTTAGATAAGGACTTTAGGAAGTATACTGCATTTACCATACCTAGTACAAACAATG
AGACACCAGGGATTAGATATCAGTACAACGTGCTTCCACAGGGATGGAAAGGATCACCAGCAATATTCCAAAG
CAGCATGACAAAGATCTTAGAACCTTTTAGAAAACAAAATCCAGACCTGGTTATCTATCAATACATGGATGAT
TTGTATGTAGGATCTGACTTAGAAATAGGGCAACATAGAACAAAAATAGAGGAACTGAGACAACATCTGTTGA
GGTGGGGATTTACCACACCAGACAAAAAACATCAGAAAGAACCTCCATTCCTTTGGATGGGTTATGAACTCCA
TCCTGATAAATGGACAGTACAGCCTATAATGCTGCCAGAAAAAGATAGCTGGACTGTCAATGACATACAGAAG
TTAGTGGGAAAATTGAATTGGGCAAGTCAGATTTATCCAGGGATTAAAGTAAGACAATTATGTAAACTCCTTA
GGGGAACCAAAGCACTAACAGACGTAATACCACTAACAGAAGAAGCAGAGCTAGAACTGGCAGAAAACAGGGA
GATTCTAAAAGAACCAGTACATGGAGTGTATTATGACCCATCAAAAGACTTAATAGCAGAAATACAGAAGCAA
GGGAATGGCCAATGGACATATCAAATATATCAAGAGCCATTTAAAAATCTGAAAACAGGAAAGTATGCAAAAA
TGAGGGGTGCCCACACTAATGATGTAAAACAGTTAACAGAGGCAGTGCAAAAAATAGCCACAGAGAGCATAGT
AATATGGGGAAAGATTCCTAAATTTAAATTACCCATACAAAAAGAAACATGGGAAGCATGGTGGACAGAGTAT
TGGCAAGCTACCTGGATTCCTGAGTGGGAGTTTGTCAATACCCCTCCCTTAGTGAAATTATGGTACCAGTTAG
AAAAAGAACCCATAGTAGGAGCAGAAACTTTCTATGTAGATGGGGCAGCTAATAGGGAGACTAAATTAGGAAA
AGCAGGATATGTTACTGACAGAGGAAGACAAAAGGTTGTCCCCCTAGCGGACACAACAAATCAGAAGACTGAG
TTACAAGCAATTCATCTAGCTTTGCAGGATTCGGGATTAGAGGTAAACATAGTAACAGACTCACAATATGCAT
TAGGAATCATTCAAGCACAACCAGATAAGAGTGAATCAGAGTTAGTCAGTCAAATAATAGAGCAGTTAATAAA
AAAGGAAAAGGTCTACCTGGCATGGGTACCAGCACACAAAGGAATTGGAGGAAATGAACAAGTAGATAAATTA
GTCAGTGCTGGAATTAGGAAAATACTATTTTTAGATGGAATAGATAAGGCCCAAGAAGACCATGAGAAATATC
ACAGTAATTGGAGAGCAATGGCTAATGAATTTAACCTGCCGCCTGTAGTAGCAAAAGAAATAGTAGCCAGCTG
TGATAAATGTCAGCTAAAAGGAGAAGCCATGCATGGACAAGTAGACTGTAGTCCAGGAATATGGCAGCTAGAT
TGCACACACTTAGAAGGAAAAATTATCCTGGTAGCAGTTCATGTAGCCAGTGGATATATAGAAGCAGAAGTTA

TTCCAGCAGAGACAGGGCAAGAAACAGCATACTTTATCTTAAAATTAGCAGGAAGATGGCCAGTAAAAACAAT
ACATACAGACAATGGCGGCAATTTCATCAGCAATACGGTTAAGGCCGCCTGTTGGTGGGCAGGGATCAAGCAG
GAATTTGGCATTCCCTACAATCCCCAAAGTCAAGGAGTAGTAGAATCTATGAATAAAGAATTAAAGAAAATTA
TAGGACAGGTAAGAGATCAGGCCGAACATCTTAAGACAGCAGTACAAATGGCAGTATTCATCCACAATTTTAA
AAGAAAAGGGGGGATTGGGGAGTACAGTGCAGGAGAAAGAATAGTAGACATAATAGCAACAGACATACAAACT
AAAGAATTACAAAACCAAATTACAAAACTTTCAAAATTTCGGGTTTATTACAGGGACAGCAGAGATCCACTGT
GGAAAGGACCAGCAAAGCTCCTCTGGAAAGGTGAAGGGGCAGTAGTAATACAAGATAATAGTGAGATAAAAGT
AGTGCCAAGAAGAAAAGCAAAGATCATTAGGGATTATGGAAAACAGATGGCAGGTGATGATTGTGTGGCAAGT
AGACAGGATGAGGATTAGAACATGGAAAAGTTTAGTAAAACACCATATGCATGTTTCAGGGAAAGCTAAGAAA
TGGTTCTATAGACATCACTATGAAAGCACTCATCCAAGAATAAGTTCAGAAGTACACATCCCACTAGGGGATG
CTGAATTAGTAATAACAACATATTGGGGTCTGCATACAGGAGAAAGAGACTGGCATTTGGGTCAGGGAGTCTC
CATAGAATGGAGGAAAAGGAGATATAGCACACAAGTAGACCCTAACCTAGCAGACCAACTAATTCATCTGTAT
TACTTTGATTGTTTTTCAGAATCTGCTATAAGAAATGCCATATTAGGACGTATAGTTAGACCTAGGTGTGATT
ATCAAGCAGGACATAACAAGGTAGGATCCCTACAGTACTTGGCATTAGCAGCATTAATAACACCAAAAAAGAT
AAAACCACCTTTGCCTAGTGTTAGGAAACTGACAGAGGATAGATGGAACAAGCCCCAGGAGACCAAGGGCCAC
AAAGGGAGCCATACAATGAATGGACACTAGAACTTTTAGAGGAACTTAAAAGTGAAGCTGTTAGACATTTTCC
TAGGCAATGGCTCCATAACTTAGGACAATATATCTATGAAACTTATGGGGATACTTGGGCAGGAGTGGAAGCC
ATTATAAGAATTCTGCAACAACTGCTGTTTATTCATTTCAGAATTGGATGTCATCATAGCAGAATAGGCATTC
TCCGACGAAGGGGAACAAGGAATGGAGCCAGTAGATCCTAGACTAGAGCCCTGGAAGCATCCAGGAAGCCAGC
CTAGGACTGCTTGTAGCAAATGCTATTGTAAAAAGTGTTGCTTTCATTGCCAAGTTTGTTTCATAACAAAAGG
CTTAGGCATCTCCTATGGCAGGAAGAAGCGGAGACAGCGACGAAGAACTCATCAAGACAGTCAGAATCATCAA
GTTTCTCTACCAAAGCAGTAAGTAGTATATGTAATGCAATCTGTACAAATATTAGCAATAGTAGCATTAGTAG
TAGTAGCAATAATAGCAATAGTTGTGTGGACCATAGTGTTAATAGAATATAGGAAAATATTAAGACAGAGAAA
AATAGACAGGTTAATTGATAGAATAATAGAAAGAGCAGAAGACAGTGGCAATGAGAGTGAAGGAGATCAGGAG
GAATTGTCAGCTCTTGTGGAGATGGGGCACCATGCTCCTTGGGATGTTAATGATCTGTAGTGCTACAGAGCAG
TTATGGGTCACAGTGTATTATGGGGTACCTGTGTGGAAAGAAGCAACCACCACTCTATTTTGTGCGTCAGATG
CTAAAGCATATGACACAGAGGCACATAATGTTTGGGCCACACATGCCTGTGTACCCACAGACCCTAACCCACA
AGAAGTAGTATTGGAAAATGTGACAGAAAATTTTAACATGTGGAAAAATAAGATGGTAGAACAGATGCAGGAG
GATATAATCAGTTTATGGGATGAAAGCCTAAAGCCATGTGTAAAACTAACCCCACTCTGTGTCACTTTAAATT
GCACTCATTTGCAGAATGTTAATATTACTAATGAGATGAAAGAAGAAATAAAAAACTGCTCCTTCAATATCAC
CACAGGCATAGGAAATCAGAGGAAGCAAGACTATGCTTTTTTCTCTAATCTTGATATAAGACCAATAGGAAAT
GATAATACAAGCTATATGTTAATAAATTGTAACACCTCAGTCATTAAACAGGCCTGTCCAAAGGTATCCTTTG
AACCAGTTCCCATACATTATTGTGCCCCGGCTGGTTTTGCGATTCTAAAGTGTAATGATAAGAAATTCAATGG
AACGGGACCATGTACAAATGTCAGCACAGTACAATGTACACATGGAATTAGGCCAGTAGTGTCAACTCAACTG
CTGCTAAATGGCAGTCTAGCAGAAGAAGAGGTAGTAATTAGATCTGAGAATTTCACGAACAATGCTAAAATCA
TAATAGTACAGCTGAACGAATCTGTAGAAATTAATTGTACAAGACCCAACAACAATACAAGAAAAGGTATACA
TATGGGACCGGGGAGAGTATTTTATACAACAGGAGAAATAGTAGGAGATATAAGACAGGCACATTGTAACATC
AGTGGAGCAAAATGGGAAAACACTTTAAAACAGATAGTTAAAAAATTACAAGAACAATTTGAGAATAAAACAA
TAACTTTTACTCAATCCTCAGGAGGGGACCCAGAAATTGTAATGCATAGTTTTAATTGTGGAGGGGAATTTTT
CTACTGTAATACAACACAGCTGTTTAATAGTACTTGGAATAATACTGGGAATAAAATTGGAGGGTCACCAAAC
ATTACAGGAAATATCACACTCCCATGCAGAATAAAACAAATTATAAACAGGTGGCAGGAAGTAGGAAAAGCGA
TGTATGCCCCTCCCATCGGAGGACTAATTAGATGTTCGTCAAGTATTACAGGGCTGCTAATAACAAGAGATGG
TGGTAATGGGTCCGAGACCAACGAGACCTTCAGACCTGGAGGAGGAGATATGAGGGACAATTGGAGAAGTGAA
TTATATAAATATAAAGTAGTAAGAATTGAGCCATTAGGAGTAGCACCCACCAAGGCAAAGAGAAGAGTGGTGC
AGAGAGAAAAAAGAGCAGTGGGAACGCTAGGAGCTATGTTCCTTGGGTTCTTGGGAGCAGCAGGAAGCACTAT
GGGCGCAGCGTCATTGACGCTGACGGTACAGGCCAGACTATTATTGTCTGGTATAGTGCAACAGCAGAACAAT
TTGCTGAGGGCTATTGAGGCGCAACAGCATCTGTTGCAACTCACAGTCTGGGGCATCAAGCAGCTCCAGGCAA
GAGTCCTGGCTGTGGAAAGATACCTAAAGGATCAACAGCTCCTAGGGATTTGGGGTTGCTCTGGAAAACTCAT
CTGCACCACTGCTGTGCCTTGGAATACTAGTTGGAGTAATAAATCTTATGGGGAAATTTGGAATAACATGACA
TGGATGCAGTGGGAGAGAGAAATTGACAATTACACACAATTAATATACACCTTAATTGAAGATTCGCAAAACC
AACAAGAAAAGAATGAACTAGAATTATTAGAATTGGATAAGTGGGCAAGTTTGTGGAATTGGTTTGACATAAC
AACATGGCTGTGGTATATAAAAATATTCATAATGATAATAGTAGGATTAGTAGGTTTAAGAATAATTTTTACT
GTGCTTTCTATAGTGAATAGAGTTAGGCAGGGATACTCACCATTATCATTCCAGACCCGCCTCCCAGCCCAGA
GGGGACCCGACAGGCCCGGAGGAATCGAAGAAGAAGGTGGAGAGAAAGACAGAGACAGATCCGGACGATTAGT

GAATGGATTCTTAGCACTCATCTGGGACGATCTGCGGAGCCTGTGCCTCTTCAGCTACCACCGCTTGAGAGAC
TTACTCTTGATTGTAGCGAGGAGTGTGGAACTTCTGGGACGCAGGGGGTGGGAGGCCCTCAAGTATTGGTGGA
ATCTCCTACAGTATTGGAGTCAGGAACTAAAGAATAGTGCTGTTAGTTTGCTTAATACCACAGCTATAGCAGT
AGCTGAAGGGACAGATAGGATTTTAGAAGTAGTACAAAGAATTTGGAGAGGTGTTCTCCACATACCTAGAAGA
ATAAGACAGGGCTTAGAAAGGCTTTTGCTATAAAATGGGTGGCAAGTGGTCAAAACGTATGAGTGATGGATGG
GCTGTTGTAAGGGAAAGAATGAGACGAGCTGCGCCAGCTGAGCCAGCAGCAGATGGGGTAGGAGCAGCATCTC
GAGACTTGGAAAGACATGGAGCAATCACAAGCAGCAATACAGCAACTAGTAATGCTGCTTGTGCATGGGTAGA
AGCACAAGAGGAGGAGGAAGAGGTGGGTTTTCCAGTCAGGCCTCAGGTACCTGTAAGACCAATGACTTACAAG
GCAGCTGTAGATCTTAGCCACTTTTTAAAAGAAAAGGGGGGACTGGAAGGGCTAGTTTACTCCCAACAAAGGC
AAGATATCCTTGATCTGTGGGTCTACAACACACAGGGCTTCTTCCCTGACTGGCAGAATTACACACCAGGGCC
AGGGGTCAGATATCCACTGACCCTTGGATGGTGCTTCAAGCTAGTACCAGTTGAGCCAGAGCAGGTAGAAGAG
GCCAATGAAGGAGAGAACAACAGTCTATTACACCCTATGAGCCTGCATGGGATGGATGACCCAGAGCATGAAA
CATTAGTGTGGAAGTTTGACAGCCGCCTAGCATTTCATCACGTGGCCCGAGAGCTGCATCCGGAATATTACAA
AGACTGCTGACATTGAGCTTTCTACAAGGGACTTTCCGCTGGGGACTTTCCAGGGAGGTGTGGCCGGGGCGGG
ACGGGGAGTGGCGAACCCTCAGATGCTGCATATAAGCAGCCGCTTTCTGCTTGTACTGGGTCTCTCTTGTTAG
ACCAGATCAGAGCCTGGGAGCTCTCTGGCTAACTAGGGAACCCACTGCTTAAGCCTCAATAAAGCTTGCCT

>V-B3 HIV-1 genome, derived from RNA genomic sequence
AGCAGTGGCGCCCGAACAGGGACTTGAAAGCGAAAGTAGAACCAGAGGAGGTCTCTCGACGCAGGACTCGGCT
TGCTGAAGCGCGCACAGCAAGAGGCGAGGGGCGGCGACTGGTGAGTACGCCAAAACTATAATTTTGACTAGCG
AAGGCTAGAAGGAGAGAGATGGGTGCGAGAGCGTCAGTATTAAGCGGGGGAAAATTAGATAAATGGGAAAAAA
TTCGGTTAAGGCCAGGGGGAAAGAAAACATATAAATTAAAACATATAGTATGGGCAAGCAGGGAGCTAGAACG
ATTCGCAGTTAATCCTGGCCTTTTAGAGACATCAGAAGGCTGTAGACAGATCCTGGGACAGCTACAACCATCC
CTTCAGACAGGATCAGAAGAACTTAAATCATTATATAATGCAGTAGCAACCCTCTATTGTGTGCATCAAAATA
TAGATGTAAGAGACACCAAGGAAGCTTTAGACAAGATAGAGGAAGAGCAAAACAAAAGTAAGAAAAGGCACA
GCAAGTAGCAGCTGACACAGAAAACAGCAGCAAGGTCAGCCAAAATTATCCTATAGTGCAGAACCTTCAGGGG
CAAATGGTACATCAGGCAATATCACCTAGAACCTTAAATGCATGGGTAAAGGTAGTAGAAGAGAAGGCTTTCA
GCCCAGAAGTAATACCCATGTTTTCAGCATTATCAGAAGGAGCCACCCCACAAGATTTAAACACCATGTTAAA
CACAGTGGGGGGACATCAAGCAGCTATGCAAATGTTAAAAGAGACCATCAATGAGGAAGCCGCAGAATGGGAT
AGATTGCATCCAGTGCATGCAGGGCCTATTGCACCAGGCCAGATGAGAGAACCAAGGGGAAGTGACATAGCAG
GAACTACTAGTACCCTTCAGGAACAAATAGGATGGATGACACATAATCCACCTATCCCAGTAGGAGAAATCTA
TAAAAGATGGATAATCATGGGATTAAATAAAATAGTAAGAATGTATAGTCCTACCAGCATTCTGGACATAAGA
CAAGGACCAAAAGAACCCTTTAGAGATTATGTAGACCGGTTCTATAAAACTCTAAGAGCCGAGCAAGCTTCAC
AGGAGGTAAAAAATTGGATGACAGAAACTTTGTTGGTCCAAAATGCGAACCCAGATTGTAAGACTATTTTAAA
AGCATTAGGGCCAGCAGCTACATTAGAAGAAATGATGACAGCATGTCAGGGAGTGGGGGGACCCGGCCATAAA
GCAAGAGTTTTGGCTGAAGCAATGAGCCAAGTAACAAATTCAGCTACCATAATGATGCAGAAAGGCAATTTTA
GGAACCAAAGAAAAACTGTTAAGTGTTTCAATTGTGGCAAAGAAGGGCACATAGCCAGAAATTGCAGGGCCCC
TAGGAAAAAGGGCTGTTGGAAATGTGGAAAGGAAGGACACCAAATGAAAGATTGTACTGAGAGACAGGCTAAT
TTTTTAGGGAAGATCTGGCCTTCCCACAAGGGAAGGCCAGGGAATTTTCTTCAGAGCAGACCAGAGCCAACAG
CCCCACCAGAAGAGAGCTTCAGGTTTGGGGAAGAGACAACGAATCCCCCTCAGAAGCAGGAGCCGATAGACAA
GGAAATGTATCCTTTAACTTCCCTCAGATCACTCTTTGGCAACGACCCCTCGTCACAGTAAAGATAGGGGGGC
AACTAAAGGAAGCTCTATTAGATACAGGAGCAGATGATACAGTACTAGAAGAAATAAGTTTGCCAGGAAGATG
GAAACCAAAAATGATAGGGGGAATTGGAGGTTTTATCAAAGTAAGACAGTATGATCAGATACCCATAGAAATC
TGCGGACATAAAGCTATAGGTACAGTATTAGTAGGACCTACACCTGTCAACATAATTGGAAGAAATCTGTTGA
CTCAGATTGGCTGCACTTTAAATTTTCCCATTAGTCCTATTGAAACTGTACCAGTAAAATTAAAGCCAGGAAT
GGATGGCCCAAGAGTTAAACAATGGCCATTGACAGAAGAAAAAATAAAAGCATTAGTAGAAATTTGTACAGAG
ATGGAAAAGGAAGGAAAAATTTCAAAAATTGGGCCTGAAAATCCATACAATACTCCAGTATTTGCCATAAAGA
AAAAAGACAGTACTAAATGGAGAAAATTAGTAGATTTCAGAGAACTTAATAAGAGAACTCAAGACTTCTGGGA
AGTTCAATTAGGAATACCACATCCCGCAGGGTTAAAAAAGAAAAAATCAGTAACAGTACTGGATGTGGGTGAT
GCATATTTTTCAGTTCCCTTAGATAAAGACTTTAGGAAGTATACTGCATTTACCATACCTAGTACAAACAATG
AGACACCAGGGATTAGATATCAGTACAACGTGCTTCCACAGGGATGGAAAGGATCACCAGCAATATTCCAAAG
CAGCATGACAAAGATCTTAGAACCTTTTAGAAAACAAAATCCAGACATGGTTATCTATCAATACATGGATGAT
TTGTATGTAGGATCTGACTTAGAAATAGGGCAACATAGAACAAAAATAGAGGAACTGAGACAACATCTGTTGA
AGTGGGGATTTACCACACCAGACAAAAAACATCAGAAAGAACCTCCATTCCTTTGGATGGGTTATGAACTCCA
TCCTGATAAATGGACAGTACAGCCTATAATGCTGCCAGAAAAAGATAGCTGGACTGTCAATGACATACAGAAG
TTAGTGGGAAAATTGAATTGGGCAAGCCAGATTTATCCAGGGATTAAAGTAAGACAATTATGTAAACTCCTTA
GGGGAACCAAAGCACTAACAGACGTAGTACAACTAACAGAAGAAGCAGAGCTAGAACTGGCAGAAAACAGGGA
GATTCTAAAAGAACCAGTACATGGAGTGTATTATGACCCATCAAAAGACTTAATAGCAGAAATACAGAAGCAA
GGGTATGGCCAATGGACATATCAAATATATCAAGAGCCATTTAAAAATCTGAAAACAGGAAAGTATGCAAAAA
TGAGGGGTGCCCACACTAATGATGTAAAACAGTTAACAGAGGCAGTGCAAAAAATAGCCACAGAGAGCATAGT
AATATGGGGAAAGATTCCTAAATTTAAATTACCCATACAAAAAGAAACATGGGAAGCATGGTGGATAGAGTAT
TGGCAAGCTACCTGGATTCCTGAGTGGGAGTTTGTCAATACCCCTCCCTTAGTGAAATTATGGTACCAGTTAG
AGAAAGAACCCATAGTAGGAGCAGAAACTTTCTATGTAGATGGGGCAGCTAATAGGGAGACTAAATTAGGAAA
AGCAGGATATGTTACTGACAGAGGAAGACAAAAGGTTGTCCCCCTAGCGGACACAACAAATCAGAAGACTGAG
TTACAAGCAATTCATCTAGCTTTGCAGGATTCGGGATTAGAGGTAAACATAGTAACAGACTCACAATATGCAT
TAGGAATCATTCAAGCACAACCAGATAAGAGTGAATCAGAGTTAGTCAGTCAAATAATAGAGCAGTTAATAAA
AAAGGAAAAGGTCTACCTGGCATGGGTACCAGCACACAAAGGAATTGGAGGAAATGAACAAGTAGATAAATTA
GTCAGTGCTGGAATTAGGAAAATACTATTTTTAGATGGAATAGATAAGGCCCAAGAAGACCATGAGAAATATC
ACAGTAATTGGAGAGCAATGGCTAATGAATTTAACCTGCCACCTGTAGTAGCAAAAGAAATAGTAGCCAGCTG
TGATAAATGTCAGCTAAAAGGAGAAGCCATGCATGGACAAGTAGACTGTAGTCCAGGAATATGGCAGCTAGAT
TGCACACACTTAGAAGGAAAAATTATCCTGGTAGCAGTTCATGTAGCCAGTGGATATATAGAAGCAGAAGTTA

TTCCAGCAGAGACAGGGCAAGAAACAGCATACTTTATCTTAAAATTAGCAGGAAGATGGCCAGTAAAAACAAT
ACATACAGACAATGGCGGCAATTTCATCAGCAATACGGTTAAGGCCGCCTGTTGGTGGGCAGGGATCAAGCAG
GAATTTGGCATTCCCTACAATCCCCAAAGTCAAGGAGTAGTAGAATCTATGAATAAAGAATTAAAGAAAATTA
TAGGACAGGTAAGAGATCAGGCCGAACATCTTAAGACAGCAGTACAAATGGCAGTATTCATCCACAATTTTAA
AAGAAAAGGGGGGATTGGGGGGTACAGTGCAGGAGAAAGAATAGTAGACATAATAGCAACAGACATACAAACT
AAAGAATTACAAAACCAAATTACAAAACTTTCAAAATTTCGGGTTTATTACAGGGACAGCAGAGATCCACTGT
GGAAAGGACCAGCAAAGCTCCTCTGGAAAGGTGAAGGGGCAGTAGTAATACAAGATAATAGTGAGATAAAAGT
AGTGCCAAGAAGAAAAGCAAAGGTCCTTAGGGATTATGGAAAACAGATGGCAGGTGATGATTGTGTGGCAAGT
AGACAGGATGAGGATTAGAACATGGAAAAGTTTAGTAAAACACCATATGTATGTTTCAGGGAAAGCTAGGAAA
TGGTTCTATAGACATCACTATGAAAGCACTCATCCAAGAATAAGTTCAGAAGTACACATCCCACTAGGGGATG
CTGAATTAGTAATAACAACATATTGGGGTCTGCATACAGGAGAAAGAGACTGGCATTTGGGTCAGGGAGTCTC
CATAGAATGGAGGAAAAAGAGATATAGCACACAAGTAGACCCTAACCTAGCAGACCAACTAATTCATCTGTAT
TACTTTGATTGTTTTTCAGAATCTGCTATAAGAAATGCCATATTAGGACGTATAGTTAGACCTAGGTGTGATT
ATCAAGCAGGACATAACAAGGTAGGATCCCTACAGTACTTGGCATTAGCAGCATTAATAACACCAAAAAAGAT
AAAACCACCTTTGCCTAGTGTTAGGAAACTGACAGAGGATAGATGGAACAAGCCCCAGGAGACCAAGGGCCAC
AGAGGGAGCCATACAATGAATGGACACTAGAGCTTTTAGAGGAACTTAAAAGTGAAGCTGTTAAACATTTTCC
TAGGCCATGGCTCCATAGCTTAGGACAATATATCTATGAAACTTATGGGGATACTTGGGCAGGAGTGGAAGCC
ATTATAAGAATTCTGCAACAAATGCTGTTTATTCATTTCAGAATTGGATGTCATCATAGCAGAATAGGTATTC
TTCGACGAAGGGGAACAAGGAATGGAGCCAGTAGATCCTAGACTAGAGCCCTGGAAGCATCCAGGGAGCCAGC
CTAGGACTGCTTGTAGCAAATGCTATTGTAAAAAGTGTTGCTTTCATTGCCAAGTTTGTTTCATAACAAAAGG
CTTAGGCATCTCCTATGGCAGGAAGAAGCGGAGACAGCGACGAAGAACTCATCAAGACAGTCAGACTCATCAA
GTTTCTCTACCAAAGCAGTAAGTAGTATATGTAATGCAATCTGTACAAATATTAGCAATAGTAGCATTAGTAG
TAGGAGCAATAATAGCAATAGTTGTGTGGACCATAGTGTTAATAGAATATAGGAAGATATTAAGACAAAGAAA
AATAGACAGGTTAATTGATAGAATAATAGAAAGAGCAGAAGACAGTGGCAATGAGAGTGAAGGAGATCAGGAG
GAATTGTCAGCTCTTGTGGAGATGGGGCACCATGCTCCTTGGGATGTTAATGATCTGTAGTGCTACAGAACAG
TTATGGGTCACAGTGTATTATGGGGTACCTGTGTGGAAAGAAGCAACCACCACTCTATTTTGTGCGTCAGATG
CTAAAGCATATGACACAGAGGCACATAATGTTTGGGCCACACATGCCTGTGTACCCACAGACCCTAACCCACA
AGAAGTAGTATTGGAAAATGTGACAGAAAATTTTAACATGTGGAAAAATAAGATGGTAGAACAGATGCAGGAG
GATATAATCAGTTTATGGGATGAAAGCCTAAAGCCATGTGTAAAACTAACCCCACTCTGTGTCACTTTAAATT
GCACTCATTTGCAGAATGTTACTTACAATAGTACTTGGGAAGGGATGAAGGAAGAAATAAAAAACTGCTCCTT
CAATATCACCACAGGCATAGGAAATAAGATGAAGAAAGAATATGCTTTTTTCTATAATCTTGATATAAGACCA
ATAGAAAATGATAATACAGATAATACAAGCTATATGTTAATAAATTGTAACACCTCAGTCATTAAACAGGCCT
GTCCAAAGGTATCCTTTGAACCAGTTCCCATACATTATTGTGCCCCGGCTGGTTTTGCGATTCTAAAGTGTAA
TGATAAGAAATTCAATGGAACGGGACCATGTACAAATGTCAGCACAGTACAATGTACACATGGAATTAGGCCA
GTAGTGTCAACTCAACTGCTGCTAAATGGCAGTCTAGCAGAAGAAGAGGTAGTAATTAGATCTGAAAATTTCA
CGAACAATGCTAAAATCATAATAGTACAGCTGAACGAATCTGTAGAAATTAATTGTACAAGACCCAACAACAA
TACAAGAAAAAGGATAACTATGGGACCGGGGAGAGTACTTTATGCAACAGGAGAAATAGTAGGAGATATAAGA
CAAGCACATTGTAACATCAGTGGAGCAAAATGGGAAAACACTTTAAAACAGATAGTTAAAAAATTACAAGAAC
AATTTGAGAATAAAACAATAATCTTTAATCAATCCTCAGGAGGGGACCCAGAAATTGTAATGCATAGTTTTAA
TTGTGGAGGGGAATTTTTCTACTGTAATACAACACAGCTGTTTAATAGTACTTGGAATAGTACTGGGAATAAA
ATTGAAGGGTCACCAAACATTACAGGAAATATCACACTCCCATGCAGAATAAAACAAATTATAAACAGGTGGC
AGGAAGTAGGAAAAGCGATGTATGCCCCTCCCATCAGAGGACTAATTAGATGTTCGTCAAATATTACAGGGCT
GCTAATAACAAGAGATGGTGGTAATGGGTCCGAGACCAAAAATAAGTCCGAGACCTTCAGACCTGGAGGAGGA
GATATGAGGGACAATTGGAGAAGTGAATTATATAAATATAAAGTAGTAAGAATTGAGCCATTAGGAGTAGCAC
CCACCAAGGCAAAGAGAAGAGTGGTGCAGAGAGAAAAAAGAGCAGTGGGAACGCTAGGAGCTATGTTCCTTGG
GTTCTTGGGAGCAGCAGGAAGCACTATGGGCGCAGCGTCATTGACGCTGACGGTACAGGCCAGACTATTATTG
TCTGGTATAGTGCAACAGCAGAACAATTTGCTGAGGGCTATTGAGGCGCAACAGCATCTGTTGCAACTCACAG
TCTGGGGCATCAAGCAGCTTCAGGCAAGAGTCCTGGCTGTGGAAAGATACCTAAAGGATCAACAGCTCCTAGG
GATTTGGGGTTGCTCTGGAAAACTCATCTGCACCACTGCTGTGCCTTGGAATACTAGTTGGAGTAATAAATCT
TATGAGGAGATTTGGAATAACATGACATGGATGCAGTGGGAGAGAGAAATTGACAATTACACACAATTAATAT
ACACCTTAATTGAAGATTCGCAAAACCAACAAGAAAAGAATGAACTAGAATTATTAGAATTGGATAAGTGGGC
AAGTTTGTGGAATTGGTTTGACATAACAAAATGGCTGTGGTATATAAAAATATTCATAATGATAATAGGAGGA
TTAGTAGGTTTAAGAATAATTTTTACTGTGCTTTCTATAGTGAATAGAGTTAGGCAGGGATACTCACCATTAT
CATTCCAGACCCGCCTCCCAGCCCAGAGGGGACCCGACAGGCCCGAAGGAATCGAAGAAGAAGGTGGAGAGAA

AGACAGAGACAGATCCGGACGATTAGTGACTGGATTCTTAGCACTCATCTGGGACGATCTGCGGAGCCTGTGC
CTCTTCAGCTACCACCGCTTGAGAGACTTACTCTTGATTGTAGCGAGGATTGTGGAACTTCTGGGACGCAGGG
GGTGGGAGGCCCTCAAGTATTGGTGGAATCTCCTACAGTATTGGAGTCAGGAACTAAAGAATAGTGCTGTTAG
TTTGCTTAATACCACAGCTATAGCAGTAGCTGAGGGGACAGATAGGATTTTAGAAGTAGTACAAAGAATTTGG
AGAGCTGTTCTCCACATACCTAGAAGAATAAGACAGGGCTTAGAAAGGCTTTTGCTATAAGATGGGTGGCAAG
TGGTCAAAACGTATGAGTGATGGATGGTCTGTTGTAAGGGAAAGAATGAGACGAGCTGCGCCAGCTGAGCCAG
CAGCAGATGGGGTGGGAGCAGCATCTCGAGACTTGGATAGACATGGAGCAATCACAAGCAGTAATACAGCAAC
TAGTAATGCTGCTTGTGCATGGGTAGAAGCACAAGAGGAGGAGGAAGAGGTGGGTTTTCCAGTCAGGCCTCAG
GTACCTGTAAGACCAATGACTTACAAGGCAGCTGTAGATCTTAGCCACTTTTTAAAAGAAAAGGGGGGACTGG
AAGGGCTAGTTTACTCCCAAAAAAGGCAAGATATCCTTGATCTGTGGGTCTACCACACACAGGGCTTCTTCCC
TGACTGGCAGAATTACACACCAGGGCCAGGGATCAGATATCCACTGACCCTTGGATGGTGCTTCAAGCTAGTA
CCAGTTGAGCCAGAGCAGGTAGAAGAGGCCAATGAAGGAGAGAACAACAGTCTATTACACCCTATGAGCCTGC
ATGGGATGGATGACCCGGAGCATGAAGTATTAGTGTGGAGGTTTGACAGCCGCCTAGCATTTCATCACATGGC
CCGAGAGCTGCATCCGGAATATTACAAAGACTGCTGACATTGAGCTTTCTACAAGGGACTTTCCGCTGGGGAC
TTTCCAGGGAGGTGTGGCCGGGGCGGACGGGGAGTGGCGAACCCTCAGATGCTGCATATAAGCAGCTGCTTT
CTGCTTGTACTGGGTGTCTCTGGTTAGACCAGATCAGAGCCTGGGAGCTCTCTGGCTAACTAGGGAACCCACT
GCTTAAGCCTCAATAAAGCTTGCCT

>V-C4 HIV-1 genome, derived from RNA genomic sequence
AGCAGTGGCGCCCGAACAGGGACTTGAAAGCGAAAGTAGAACCAGAGGAGATCTCTCGACGCAGGACTCGGCT
TGCTGAAGTGCGCACAGCAAGAGGCGAGGGGCGGCGACTGGTGAGTACGCCAAAACTTTTGACTAGCGGAGGC
TAGAAGGAGAGAGATGGGTGCGAGAGCGTCAGTATTAAGCGGGGGAAAATTAGACAAATGGGAAAAAATTCGG
TTAAGGCCAGGGGGAAAGAAAACATATCAATTAAAACATATAGTATGGGCAAGCAGGGAGCTAGAACGATTCG
CAGTTAATCCTGGCCTTTTAGAAACATCAGAAGGCTGTAGACAGATCCTGGGACAGCTACAACCATCCCTTCA
GACAGGATCAGAAGAACTTAAATCATTATATAATGCAGTAGCAACCCTCTATTGTGTGCATCAAAATATAGAT
GTAAGAGACACCAAGGAAGCTTTAGACAAGATAGAGGAAGAGCAAAACAAAAGTAAGAAAAGGCACAGCAAG
TAGCAGCTGACACAGAAAACAGCAGCAAGGTCAGCCAAAATTATCCTATAGTGCAGAACCTTCAGGGGCAAAT
GGTACATCAGGCACTATCACCTAGAACCTTAAATGCATGGGTAAAGGTAATAGAAGAGAAGGCTTTCAGCCCA
GAAGTAATACCCATGTTTTCAGCATTATCAGAAGGAGCCACCCCACAAGATTTAAACACCATGTTAAACACAG
TGGGGGGACATCAAGCAGCTATGCAAATGTTAAAAGAGACCATCAATGAGGAAGCTGCAGAATGGGATAGATT
GCATCCAGTGCATGCAGGGCCTATTGCACCAGGCCAGATGAGAGAACCAAGGGGAAGTGACATAGCAGGAACT
ACTAGTACCCTTCAGGAACAAATAGGATGGATGACACATAATCCACCTATCCCAGTAGGAGAAATCTATAAAA
GATGGATAATCATGGGATTAAATAAAATAGTAAGAATGTATAGTCCTATCAGCATTCTGGACATAAGACAAGG
ACCAAAAGAACCCTTTAGAGACTATGTAGACCGGTTCTATAAAACTCTAAGAGCCGAGCAAGCTTCACAGGAG
GTAAAAAATTGGATGACAGAAACTTTGTTGGTTCAAAATGCAAACCCAGATTGTAAGACTATTTTAAAAGCAT
TAGGGCCAGGAGCTACATTAGAAGAAATGATGACAGCATGTCAGGGAGTGGGGGGACCCGGCCATAAAGCAAG
AGTTTTGGCTGAAGCAATGAGCCAAGTAACAAATTCAGCTACCATAATGATGCAGAAAGGCAATTTTAGGAAC
CAAAGAAAAACTGTTAAGTGTTTCAATTGTGGCAAAGAAGGGCACATAGCCAGAAATTGCAGGGCCCCTAGGA
AAAAGGGCTGTTGGAAATGTGGAAAGGAAGGACACCAAATGAAAGATTGTACTGAGAGACAGGCTAATTTTTT
AGGGAAGATCTGGCCTTCCCACAAGGGGAGGCCAGGGAATTTTCTTCAGAGCAGACCAGAGCCAACAGCCCCA
CCAGCAGAGAGCTTCAGGTTTGGGGAAGAGACAACGAATCCCCCTCAGAAGCAGGAGCCGATAGACAAGGAGA
TGTATCCTTTAACCTCCCTCAGATCACTCTTTGGCAACGACCCCTCGTCACAGTAAAGATAGGGGGGCAACTA
AAGGAAGCTCTATTAGATACAGGAGCAGATGATACAGTACTAGAAGAAATAAATTTGCCAGGAAGATGGAAAC
CAAAAATGATAGGGGGAATTGGAGGTTTTATCAAAGTAAGACAGTATGATCAGATACCCATAGAAATCTGTGG
ACATAAAGCTATAGGTACAGTATTAGTAGGACCTACACCTGTCAACATAATTGGAAGAAATCTGTTGACTCAG
ATTGGCTGCACTTTAAATTTTCCCATTAGTCCTATTGAAACTGTACCAGTAAAATTAAAGCCAGGAATGGATG
GCCCAAGAGTTAAACAATGGCCATTGACAGAAGAAAAAATAAAAGCATTAGTAGAAATTTGTACAGAGATGGA
AAAGGAAGGAAAAATTTCAAAAATTGGGCCTGAAAATCCATACAATACTCCAGTATTTGCCATAAAGAAAAAA
GACAGTACTAAATGGAGAAAATTAGTAGATTTCAGAGAACTTAATAAGAGAACTCAAGACTTCTGGGAAGTTC
AATTAGGAATACCACATCCCGCAGGGTTAAAAAAGAAAAAATCAGTAACAGTACTGGATGTGGGTGATGCATA
TTTTTCAGTTCCCTTAGATAAAGACTTTAGGAAGTATACTGCATTTACCATACCTAGTACAAACAATGAGACA
CCAGGGATTAGATATCAGTACAACGTGCTTCCACAGGGATGGAAAGGATCACCAGCAATATTCCAAAGCAGCA
TGACAAAGATCTTAGAACCTTTTAGAAAACAAAATCCAGACCTGGTTATCTATCAATACATGGATGATTTGTA
TGTAGGATCTGACTTAGAAATAGGGCAACATAGAACAAAAATAGAGGAACTGAGACAACATCTGTTGAAGTGG
GGATTTACCACACCAGACAAAAAACATCAGAAAGAACCTCCATTCCTTTGGATGGGTTATGAACTCCATCCTG
ATAAATGGACAGTACAGCCTATAATGTTGCCAGAAAAAGATAGCTGGACTGTCAATGACATACAGAAGTTAGT
GGGAAAATTGAATTGGGCAAGTCAGATTTATCCAGGGATTAAAGTAAGACAATTATGTAAACTCCTTAGGGGA
ACCAAAGCACTAACAGATGTAATACCACTAACAGAAGAAGCAGAGCTAGAACTGGCAGAAAACAGGGAGATTC
TAAAAGAACCAGTACATGGAGTGTATTATGACCCATCAAAAGACTTAATAGCAGAAATACAGAAGCAAGGGAA
TGGCCAATGGACATATCAAATATATCAAGAGCCATTTAAAAATCTGAAAACAGGAAAGTATGCAAAAATGAGG
GGTGCCCACACTAATGATGTAAAACAGTTAACAGAGGCAGTGCAAAAAATAGCCACAGAGAGCATAGTAATAT
GGGGAAAAATTCCTAAATTTAAATTACCCATACAAAAAGAAACATGGGAAGCATGGTGGATAGAGTATTGGCA
AGCTACCTGGATTCCTGAGTGGGAGTTTGTCAATACCCCTCCCTTAGTGAAATTATGGTACCAGTTAGAAAAA
GAACCCATAGTAGGAGCAGAAACTTTCTATGTAGATGGGGCAGCTAATAAGGAAACTAAATTAGGAAAAGCAG
GATATGTTACTGACAGGGGAAGACAAAAGGTTGTCCCCCTAGCAGACACAACAAATCAGAAGACTGAGTTACA
AGCAATTCATCTAGCTTTGCAGGATTCGGGATTAGAGGTAAACATAGTAACAGACTCACAATATGCATTAGGA
ATCATTCAAGCACAACCAGATAAGAGTGAATCAGAGTTAGTCAGTCAAATAATAGAGCAGTTAATAAAAAAGG
AAAAGGTCTACCTGGCTTGGGTACCAGCACATAAAGGAATTGGAGGAAATGAACAAGTAGATAAATTAGTCAG
TGCTGGAATTAGAAAAATACTATTTTTAGATGGAATAGATAAGGCCCAAGAAGACCATGAGAAATATCACAGT
AATTGGAGAGCAATGGCTAATGAATTTAACCTACCACCTGTAGTAGCAAAAGAAATAGTAGCCAGCTGTGATA
AATGTCAGCTAAAAGGAGAAGCCATGCATGGACAAGTAGACTGTAGTCCAGGAATATGGCAGCTAGATTGCAC
ACACTTAGAAGGAAAAATTATCCTGGTAGCAGTTCATGTAGCCAGTGGATATATAGAAGCAGAAGTTATTCCA

GCAGAGACAGGGCAAGAAACAGCATACTTTATCTTAAAATTAGCAGGAAGATGGCCAGTAAAAACAATACATA
CAGACAATGGCGGCAATTTCATCAGCAATACGGTTAAGGCCGCCTGTTGGTGGGCAGGGATCAAGCAGGAATT
TGGCATTCCCTACAATCCCCAAAGTCAAGGAGTAGTAGAATCTATGAATAAAGAATTAAAGAAAATTATAGGA
CAGGTAAGAGATCAGGCCGAACATCTTAAGACAGCAGTACAAATGGCAGTATTCATCCACAATTTTAAAAGAA
AAGGGGGGATTGGGGAGTACAGTGCAGGAGAAAGAATAGTAGACATAATAGCAACAGACATACAAACTAAAGA
ATTACAAAACCAAATTACAAAACTTTCAAAATTTCGGGTTTATTACAGGGACAGCAGAGATCCACTGTGGAAA
GGACCAGCAAAGCTCCTCTGGAAAGGTGAAGGGGCAGTAGTAATACAAGATAATAGTGAGATAAAAGTAGTGC
CAAGAAGAAAAGCAAAGATCATTAGGGATTATGGAAAACAGATGGCAGGTGATGATTGTGTGGCAAGTAGACA
GGATGAGGATTAGAACATGGAAAAGTTTAGTAAAACACCATATGCATGTTTCAGGGAAAGCTAAGAAATGGTT
CTATAGACATCACTATGAAAGCACTCATCCAAGAATAAGTTCAGAAGTACACATCCCACTAGGGGATGCTGAA
TTAGTAATAACAACATATTGGGGTCTGCATACAGGAGAAAGAGACTGGCATTTGGGGCAGGGAGTCTCCATAG
AATGGAGGAAAAAGAGATATAGCACACAAGTAGACCCTAACCTAGCAGACCAACTAATTCATCTGTATTACTT
TGATTGTTTTTCAGAATCTGCTATAAGAAATGCCATATTAGGACGTATAGTTAGACCTAGGTGTGATTATCAA
GCAGGACATAACAAGGTAGGATCCCTACAGTACTTGGCATTAGCAGCATTAATAACACCAAAAAAGATAAAAC
CACCTTTGCCTAGTGTTAGGAAACTGACAGAGGATAGATGGAACAAGCCCCAGGAGACCAAGGGCCACAGAGG
GAGCCATACAATGAATGGACACTAGAGCTTTTAGAGGAACTTAAAAGTGAAGCTGTTAGACATTTTCCTAGGG
CATGGCTCCAGAGCTTAGGACAATATATCTATGAAACTTATGGGGATACTTGGGCAGGAGTGGAAGCCATTAT
AAGAATCCTGCAACAACTGCTGTTTATTCATTTCAGAATTGGATGTCATCATAGCAGAATAGGCATTCTCCGA
CGAAGGGGAACAAGGAATGGAGCCAGTAGATCCTAGACTAGAGCCCTGGAAGCATCCAGGAAGCCAGCCTAGG
ACTGCTTGTAGCAAATGCTATTGTAAAAAGTGTTGCTTTCATTGCCAAGTTTGTTTCATAACAAAAGGCTTAG
GCATCTCCTATGGCAGGAAGAAGCGGAGACAGCGACGAAGAACTCCTCAAGACAGTCAGACTCATCAAGTTTC
TCTACCAAAGCAGTGAGTAGTATATGTAATGCAATCTGTACAGATATTAGCAATAGTAGCATTAGTAGTAGTA
GCAATAATAGCAATAGTTGTGTGGACCATAGTGTTAATAGAATATAGGAAAATATTAAGACAAAGAAAAATAG
ACAGGATAATTGATAGAATAATAGAAAGAGCAGAAGACAGTGGCAATGAGAGTGAAGGAGATCAGGAGGAATT
GTCAGCTCTTGTGGAGATGGGGCACCATGCTCCTTGGGATGTTAATGATCTGTAGTGCTACAGAACAGTTATG
GGTCACAGTGTATTATGGGGTACCTGTGTGGAAAGAAGCAACCACCACTCTATTTTGTGCGTCAGATGCTAAA
GCATATGACACAGAGGCACATAATGTTTGGGCCACACATGCCTGTGTACCCACAGACCCTAACCCACAAGAAG
TAGTATTGGAGAATGTGACAGAAAATTTTAACATGTGGAAAAATAAGATGGTAGAACAGATGCAGGAGGATAT
AATCAGTTTATGGGATGAAAGCCTAAAGCCATGTGTAAAACTAACCCCACTCTGTGTCACTTTAAATTGCACT
CATTTGACTTACAATAATACTTGGAATGGGATGAAGGAAGAAATAAAAAACTGTTCCTTCAATATCACCACAG
GCATAGGAAATCAGAGGAAGCAAGACTATGCTCTTTTCTATAATCTTGATATAAGACCAGTAGGAAATAATAA
TACAGATAATACAAGCTATATGTTAATAAATTGTAACACCTCAGTCATTAAACAGGCCTGTCCAAAGGTATCC
TTTGAACCAGTTCCCATACATTATTGTGCCCCGGCTGGTTTTGCGATTCTAAAGTGTAATGATAAGAAATTCA
ATGGAACGGGACCATGTACAAATGTCAGCACAGTACAATGTACACATGGAATTAGGCCAGTAGTGTCAACTCA
ACTGCTGCTAAATGGCAGTCTAGCAGAAGAAGAGGTAGTAATTAGATCTGAAAATTTCACGAACAATGCTAAA
ATCATAATAGTACAGCTGAACGAATCTGTAGAAATTAATTGTACAAGACCCAACAACCACACAAGAAAAAGGA
TAACTATGGGACCGGGGAGAGTATTTTATACAACAGGAGAAATAGTAGGAGATGTAAGACGAGCACATTGTAA
CATCAGTGGAGCAAAATGGGAAAACACTTTAAAACAGATAGTTAAAAAATTACAAGAACAATTTGAGAATACA
ACAATAACCTTTACTCAATCCTCAGGAGGGGACCCAGAAATTGTAATGCATAGTTTTAATTGTGGAGGGGAAT
TTTTCTACTGTAATACAACACAGCTGTTTAATAGTACTTGGAATAGTACTGGGAATAAAACTAAAGGGTCACC
AAACATTACAGGAAATATCACACTCCCATGCAGAATAAAACAAATTATAAACAGGTGGCAGGAAGTAGGAAAA
GCGATGTATGCCCCTCCCATCGGAGGACTAATTAGATGTTCGTCAAATATTACAGGGCTGCTAATAACAAGAG
ATGGTGGTAATGGGTCCGAGACCAATAAGTCTGAGACCTTCAGACCTGGAGGAGGAGATATGAGGGACAATTG
GAGAAGTGAATTATATAAATATAAAGTAGTAAGAATTGAGCCATTAGGAGTAGCACCCACCAAGGCAAAGAGA
AGAGTGGTGCAGAGAGAAAAAAGAGCAGTGGGAACGCTAGGAGCTATGTTCCTTGGGTTCTTGGGAGCAGCAG
GAAGCACTATGGGCGCAGCGTCATTGACGCTGACGGTACAGGCCAGACTATTGTTGTCTGGTATAGTGCAACA
GCAGAACAATTTGCTGAGGGCTATTGAGGCGCAACAGCATCTGTTGCAACTCACAGTCTGGGGCATCAAGCAA
CTCCAGGCAAGAGTCCTGGCTGTGGAAAGATACCTAAAGGATCAACAGCTCCTAGGGATTTGGGGTTGCTCTG
GAAAACTCATCTGCACCACTGCTGTGCCTTGGAATACTAGTTGGAGTAATAAATCTTATGGGGAGATTTGGAA
TAACATGACATGGATGCAGTGGGAGAGAGAAATTGACAATTACACACAATTAATATACACCTTAATTGAAGAT
TCGCAAAACCAACAAGAAAAGAATGAACTAGAATTATTAGAATTGGATAAGTGGGCAAATTTGTGGAACTGGT
TTGACATAACAACATGGCTGTGGTATATAAAAATATTCATAATGATAATAGGAGGATTAGTAGGTTTAAGAAT
AATTTTTACTGTGCTTTCTATAGTGAATAGAGTTAGGCAGGGATACTCACCATTATCATTCCAGACCCGCCTC
CCAACCCAGAGGGGACCCGACAGGCCCGAAGGAATCGGAGAAGAAGGTGGAGAGAAAGACAGAGACAGATCCG

GACGATTAGTGAATGGATTCTTAGCACTCATCTGGGACGATCTGCGGAGCCTGTGCCTCTTCAGCTACCACCG
CTTGAGAGACTTACTCTTAATTGTAGCGAGGACTGTGGAACTTCTGGGACGCAGGGGGTGGGAGGCCCTCAAG
TATTGGTGGAATCTCCTACAGTATTGGAGTCAGGAACTAAAGAATAGTGCTGTTAGTTTGCTTAATACCACAG
CTATAGCAGTAGCTGAGGGGACAGATAGGATTTTAGAAGTAGTACAAAGAATTTGGAGAGGTGTTCTCCACAT
ACCTACAAGAATAAGACAGGGCTTAGAAAGGCTTTTGCTATAAGATGGGTGGCAAATGGTCAAAAAGTATGAG
GGAGGGATGGTCTGTTGTAAGGGAAAGAATGAGACGAGCTGCGCCAGCTGCTGCGCCAGCTGAGCCAGCAGCA
GATGGGGTGGGAGCAGCATCTCGAGACTTGGATAGACATGGAGCAATCACAAGCAGCAATACAGCAACTAGTA
ATGCTGCTTGTGCATGGGTAGAAGCACAAGAGGAGGAGGAAGAGGTGGGTTTTCCAGTCAGGCCTCAGGTACC
TGTAAGACCAATGACTTACAAGGCAGCTGTAGATCTTAGCCACTTTTTAAAAGAAAAGGGGGGACTGGAAGGG
CTAGTTTACTCCCAACAAAGGCAAGATATCCTTGATCTGTGGGTCTACAACACACAGGGCTTCTTCCCTGACT
GGCAGAATTACACACCAGGGCCAGGGACCAGATTTCCACTGACCTTTGGATGGTGCTTCAAGTTAGTACCAGT
TGATCCAGAGCAGGTAGAAGAAGCCAATGAAGGAGAGAACAACAGTCTATTACATCCTATAAGCCTGCATGGA
ATGGATGACCCAGAGCATGAAGTATTAGTGTGGAAGTTTGACAGCCGCCTAGCATTTCATCACATGGCCCGAG
AGCTGCATCCGGAATATTACAAAGACTGCTGACATTGAGCTTTCTACAAGGGACTTTCCGCTGGGGACTTTCC
AGGGAGGTGTGGCCGGGGCGGGACGGGGAGTGGCGAACCCTCAGATACTGCATATAAGCAGCTGCTTTCTGCT
TGTACTGGGTCTCTCTTGTTAGACCAGATCAGAGCCTGGGAGCTCTCTGGCTAACTAGGGAACCCACTGCTTA
AGCCTCAATAAAGCTTGCCT

>V-C5 HIV-1 genome, derived from RNA genomic sequence
AGCAGTGGCGCCCGAACAGGGACTTGAAAGCGAAAGTAGAACCAGAGGAGGTCTCTCGACGCAGGACTCGGCT
TGCTGAAGCGCGCACAGCAAGAGGCGAGGGGCGGCGACTGGTGAGTACGCCAAAACTATAATTTTGACTAGCG
AAGGCTAGAAGGAGAGAGATGGGTGCGAGAGCGTCAGTATTAAGCGGGGGAAAATTAGATAAATGGGAAAAAA
TTCGGTTAAGGCCAGGGGGAAAGAAAACATATAAATTAAAACATATAGTATGGGCAAGCAGGGAGCTAGAACG
ATTCGCAGTTAATCCTGGCCTTTTAGAGACATCAGAAGGCTGTAGACAGATCCTGGGACAGCTACAACCATCC
CTTCAGACAGGATCAGAAGAACTTAAATCATTATATAATGCAGTAGCAACCCTCTATTGTGTGCATCAAAATA
TAGATGTAAGAGACACCAAGGAAGCTTTAGACAAGATAGAGGAAGAGCAAAACAAAAGTAAGAAAAGGGCACA
GCAAGTAGCAGCTGACACAGAAAACAGCAGCAAGGTCAGCCAAAATTATCCTATAGTGCAGAACCTTCAGGGG
CAAATGGTACATCAGGCAATATCACCTAGAACCTTAAATGCATGGGTAAAGGTAGTAGAAGAGAAGGCTTTCA
GCCCAGAAGTAATACCCATGTTTTCAGCATTATCAGAAGGAGCCACCCCACAAGATTTAAACACCATGTTAAA
CACAGTGGGGGGACATCAAGCAGCTATGCAAATGTTAAAAGAGACCATCAATGAGGAAGCCGCAGAATGGGAT
AGATTGCATCCAGTGCATGCAGGGCCTATTGCACCAGGCCAGATGAGAGAACCAAGGGGAAGTGACATAGCAG
GAACTACTAGTACCCTTCAGGAACAAATAGGATGGATGACACATAATCCACCTATCCCAGTAGGAGAAATCTA
TAAAAGATGGATAATCATGGGATTAAATAAAATAGTAAGAATGTATAGTCCTACCAGCATTCTGGACATAAGA
CAAGGACCAAAAGAACCCTTTAGAGATTATGTAGACCGGTTCTATAAAACTCTAAGAGCCGAGCAAGCTTCAC
AGGAGGTAAAAAATTGGATGACAGAAACTTTGTTGGTCCAAAATGCGAACCCAGATTGTAAGACTATTTTAAA
AGCATTAGGGCCAGCAGCTACATTAGAAGAAATGATGACAGCATGTCAGGGAGTGGGGGGACCCGGCCATAAA
GCAAGAGTTTTGGCTGAAGCAATGAGCCAAGTAACAAATTCAGCTACCATAATGATGCAGAAAGGCAATTTTA
GGAACCAAAGAAAAACTGTTAAGTGTTTCAATTGTGGCAAAGAAGGGCACATAGCCAGAAATTGCAGGGCCCC
TAGGAAAAAGGGCTGTTGGAAATGTGGAAAGGAAGGACACCAAATGAAAGATTGTACTGAGAGACAGGCTAAT
TTTTTAGGGAAGATCTGGCCTTCCCACAAGGGAAGGCCAGGGAATTTTCTTCAGAGCAGACCAGAGCCAACAG
CCCCACCAGAAGAGAGCTTCAGGTTTGGGGAAGAGACAACGAATCCCCCTCAGAAGCAGGAGCCGATAGACAA
GGAAATGTATCCTTTAACTTCCCTCAGATCACTCTTTGGCAACGACCCCTCGTCACAGTAAAGATAGGGGGGC
AACTAAAGGAAGCTCTATTAGATACAGGAGCAGATGATACAGTACTAGAAGAAATAAGTTTGCCAGGAAGATG
GAAACCAAAAATGATAGGGGGAATTGGAGGTTTTATCAAAGTAAGACAGTATGATCAGATACCCATAGAAATC
TGCGGACATAAAGCTATAGGTACAGTATTAGTAGGACCTACACCTGTCAACATAATTGGAAGAAATCTGTTGA
CTCAGATTGGCTGCACTTTAAATTTTCCCATTAGTCCTATTGAAACTGTACCAGTAAAATTAAAGCCAGGAAT
GGATGGCCCAAGAGTTAAACAATGGCCATTGACAGAAGAAAAAATAAAAGCATTAGTAGAAATTTGTACAGAG
ATGGAAAAGGAAGGAAAAATTTCAAAAATTGGGCCTGAAAATCCATACAATACTCCAGTATTTGCCATAAAGA
AAAAAGACAGTACTAAATGGAGAAAATTAGTAGATTTCAGAGAACTTAATAAGAGAACTCAAGACTTCTGGGA
AGTTCAATTAGGAATACCACATCCCGCAGGGTTAAAAAAGAAAAAATCAGTAACAGTACTGGATGTGGGTGAT
GCATATTTTTCAGTTCCCTTAGATAAAGACTTTAGGAAGTATACTGCATTTACCATACCTAGTACAAACAATG
AGACACCAGGGATTAGATATCAGTACAACGTGCTTCCACAGGGATGGAAAGGATCACCAGCAATATTCCAAAG
CAGCATGACAAAGATCTTAGAACCTTTTAGAAAACAAAATCCAGACATGGTTATCTATCAATACATGGATGAT
TTGTATGTAGGATCTGACTTAGAAATAGGGCAACATAGAACAAAAATAGAGGAACTGAGACAACATCTGTTGA
AGTGGGGATTTACCACACCAGACAAAAAACATCAGAAAGAACCTCCATTCCTTTGGATGGGTTATGAACTCCA
TCCTGATAAATGGACAGTACAGCCTATAATGCTGCCAGAAAAAGATAGCTGGACTGTCAATGACATACAGAAG
TTAGTGGGAAAATTGAATTGGGCAAGCCAGATTTATCCAGGGATTAAAGTAAGACAATTATGTAAACTCCTTA
GGGGAACCAAAGCACTAACAGACGTAGTACAACTAACAGAAGAAGCAGAGCTAGAACTGGCAGAAAACAGGGA
GATTCTAAAAGAACCAGTACATGGAGTGTATTATGACCCATCAAAAGACTTAATAGCAGAAATACAGAAGCAA
GGGTATGGCCAATGGACATATCAAATATATCAAGAGCCATTTAAAAATCTGAAAACAGGAAAGTATGCAAAAA
TGAGGGGTGCCCACACTAATGATGTAAAACAGTTAACAGAGGCAGTGCAAAAAATAGCCACAGAGAGCATAGT
AATATGGGGAAAGATTCCTAAATTTAAATTACCCATACAAAAAGAAACATGGGAAGCATGGTGGATAGAGTAT
TGGCAAGCTACCTGGATTCCTGAGTGGGAGTTTGTCAATACCCCTCCCTTAGTGAAATTATGGTACCAGTTAG
AGAAAGAACCCATAGTAGGAGCAGAAACTTTCTATGTAGATGGGGCAGCTAATAGGGAGACTAAATTAGGAAA
AGCAGGATATGTTACTGACAGAGGAAGACAAAAGGTTGTCCCCCTAGCGGACACAACAAATCAGAAGACTGAG
TTACAAGCAATTCATCTAGCTTTGCAGGATTCGGGATTAGAGGTAAACATAGTAACAGACTCACAATATGCAT
TAGGAATCATTCAAGCACAACCAGATAAGAGTGAATCAGAGTTAGTCAGTCAAATAATAGAGCAGTTAATAAA
AAAGGAAAAGGTCTACCTGGCATGGGTACCAGCACACAAAGGAATTGGAGGAAATGAACAAGTAGATAAATTA
GTCAGTGCTGGAATTAGGAAAATACTATTTTTAGATGGAATAGATAAGGCCCAAGAAGACCATGAGAAATATC
ACAGTAATTGGAGAGCAATGGCTAATGAATTTAACCTGCCACCTGTAGTAGCAAAAGAAATAGTAGCCAGCTG
TGATAAATGTCAGCTAAAAGGAGAAGCCATGCATGGACAAGTAGACTGTAGTCCAGGAATATGGCAGCTAGAT
TGCACACACTTAGAAGGAAAAATTATCCTGGTAGCAGTTCATGTAGCCAGTGGATATATAGAAGCAGAAGTTA

TTCCAGCAGAGACAGGGCAAGAAACAGCATACTTTATCTTAAAATTAGCAGGAAGATGGCCAGTAAAAACAAT
ACATACAGACAATGGCGGCAATTTCATCAGCAATACGGTTAAGGCCGCCTGTTGGTGGGCAGGGATCAAGCAG
GAATTTGGCATTCCCTACAATCCCCAAAGTCAAGGAGTAGTAGAATCTATGAATAAAGAATTAAAGAAAATTA
TAGGACAGGTAAGAGATCAGGCCGAACATCTTAAGACAGCAGTACAAATGGCAGTATTCATCCACAATTTTAA
AAGAAAAGGGGGGATTGGGGGGTACAGTGCAGGAGAAAGAATAGTAGACATAATAGCAACAGACATACAAACT
AAAGAATTACAAAACCAAATTACAAAACTTTCAAAATTTCGGGTTTATTACAGGGACAGCAGAGATCCACTGT
GGAAAGGACCAGCAAAGCTCCTCTGGAAAGGTGAAGGGGCAGTAGTAATACAAGATAATAGTGAGATAAAAGT
AGTGCCAAGAAGAAAAGCAAAGGTCCTTAGGGATTATGGAAAACAGATGGCAGGTGATGATTGTGTGGCAAGT
AGACAGGATGAGGATTAGAACATGGAAAAGTTTAGTAAAACACCATATGTATGTTTCAGGGAAAGCTAGGAAA
TGGTTCTATAGACATCACTATGAAAGCACTCATCCAAGAATAAGTTCAGAAGTACACATCCCACTAGGGGATG
CTGAATTAGTAATAACAACATATTGGGGTCTGCATACAGGAGAAAGAGACTGGCATTTGGGTCAGGGAGTCTC
CATAGAATGGAGGAAAAAGAGATATAGCACACAAGTAGACCCTAACCTAGCAGACCAACTAATTCATCTGTAT
TACTTTGATTGTTTTTCAGAATCTGCTATAAGAAATGCCATATTAGGACGTATAGTTAGACCTAGGTGTGATT
ATCAAGCAGGACATAACAAGGTAGGATCCCTACAGTACTTGGCATTAGCAGCATTAATAACACCAAAAAAGAT
AAAACCACCTTTGCCTAGTGTTAGGAAACTGACAGAGGATAGATGGAACAAGCCCCAGGAGACCAAGGGCCAC
AGAGGGAGCCATACAATGAATGGACACTAGAGCTTTTAGAGGAACTTAAAAGTGAAGCTGTTAAACATTTTCC
TAGGCCATGGCTCCATAGCTTAGGACAATATATCTATGAAACTTATGGGGATACTTGGGCAGGAGTGGAAGCC
ATTATAAGAATTCTGCAACAAATGCTGTTTATTCATTTCAGAATTGGATGTCATCATAGCAGAATAGGTATTC
TTCGACGAAGGGGAACAAGGAATGGAGCCAGTAGATCCTAGACTAGAGCCCTGGAAGCATCCAGGGAGCCAGC
CTAGGACTGCTTGTAGCAAATGCTATTGTAAAAAGTGTTGCTTTCATTGCCAAGTTTGTTTCATAACAAAAGG
CTTAGGCATCTCCTATGGCAGGAAGAAGCGGAGACAGCGACGAAGAACTCATCAAGACAGTCAGACTCATCAA
GTTTCTCTACCAAAGCAGTAAGTAGTATATGTAATGCAATCTGTACAAATATTAGCAATAGTAGCATTAGTAG
TAGGAGCAATAATAGCAATAGTTGTGTGGACCATAGTGTTAATAGAATATAGGAAGATATTAAGACAAAGAAA
AATAGACAGGTTAATTGATAGAATAATAGAAAGAGCAGAAGACAGTGGCAATGAGAGTGAAGGAGATCAGGAG
GAATTGTCAGCTCTTGTGGAGATGGGGCACCATGCTCCTTGGGATGTTAATGATCTGTAGTGCTACAGAACAG
TTATGGGTCACAGTGTATTATGGGGTACCTGTGTGGAAAGAAGCAACCACCACTCTATTTTGTGCGTCAGATG
CTAAAGCATATGACACAGAGGCACATAATGTTTGGGCCACACATGCCTGTGTACCCACAGACCCTAACCCACA
AGAAGTAGTATTGGAAAATGTGACAGAAAATTTTAACATGTGGAAAAATAAGATGGTAGAACAGATGCAGGAG
GATATAATCAGTTTATGGGATGAAAGCCTAAAGCCATGTGTAAAACTAACCCCACTCTGTGTCACTTTAAATT
GCACTCATTTGCAGAATGTTACTTACAATAGTACTTGGGAAGGGATGAAGGAAGAAATAAAAAACTGCTCCTT
CAATATCACCACAGGCATAGGAAATAAGATGAAGAAAGAATATGCTTTTTTCTATAATCTTGATATAAGACCA
ATAGAAAATGATAATACAGATAATACAAGCTATATGTTAATAAATTGTAACACCTCAGTCATTAAACAGGCCT
GTCCAAAGGTATCCTTTGAACCAGTTCCCATACATTATTGTGCCCCGGCTGGTTTTGCGATTCTAAAGTGTAA
TGATAAGAAATTCAATGGAACGGGACCATGTACAAATGTCAGCACAGTACAATGTACACATGGAATTAGGCCA
GTAGTGTCAACTCAACTGCTGCTAAATGGCAGTCTAGCAGAAGAAGAGGTAGTAATTAGATCTGAAAATTTCA
CGAACAATGCTAAAATCATAATAGTACAGCTGAACGAATCTGTAGAAATTAATTGTACAAGACCCAACAACAA
TACAAGAAAAAGGATAACTATGGGACCGGGGAGAGTACTTTATGCAACAGGAGAAATAGTAGGAGATATAAGA
CAAGCACATTGTAACATCAGTGGAGCAAAATGGGAAAACACTTTAAAACAGATAGTTAAAAAATTACAAGAAC
AATTTGAGAATAAAACAATAATCTTTAATCAATCCTCAGGAGGGGACCCAGAAATTGTAATGCATAGTTTTAA
TTGTGGAGGGGAATTTTTCTACTGTAATACAACACAGCTGTTTAATAGTACTTGGAATAGTACTGGGAATAAA
ATTGAAGGGTCACCAAACATTACAGGAAATATCACACTCCCATGCAGAATAAAACAAATTATAAACAGGTGGC
AGGAAGTAGGAAAAGCGATGTATGCCCCTCCCATCAGAGGACTAATTAGATGTTCGTCAAATATTACAGGGCT
GCTAATAACAAGAGATGGTGGTAATGGGTCCGAGACCAAAAATAAGTCCGAGACCTTCAGACCTGGAGGAGGA
GATATGAGGGACAATTGGAGAAGTGAATTATATAAATATAAAGTAGTAAGAATTGAGCCATTAGGAGTAGCAC
CCACCAAGGCAAAGAGAAGAGTGGTGCAGAGAGAAAAAAGAGCAGTGGGAACGCTAGGAGCTATGTTCCTTGG
GTTCTTGGGAGCAGCAGGAAGCACTATGGGCGCAGCGTCATTGACGCTGACGGTACAGGCCAGACTATTATTG
TCTGGTATAGTGCAACAGCAGAACAATTTGCTGAGGGCTATTGAGGCGCAACAGCATCTGTTGCAACTCACAG
TCTGGGGCATCAAGCAGCTTCAGGCAAGAGTCCTGGCTGTGGAAAGATACCTAAAGGATCAACAGCTCCTAGG
GATTTGGGGTTGCTCTGGAAAACTCATCTGCACCACTGCTGTGCCTTGGAATACTAGTTGGAGTAATAAATCT
TATGAGGAGATTTGGAATAACATGACATGGATGCAGTGGGAGAGAGAAATTGACAATTACACACAATTAATAT
ACACCTTAATTGAAGATTCGCAAAACCAACAAGAAAAGAATGAACTAGAATTATTAGAATTGGATAAGTGGGC
AAGTTTGTGGAATTGGTTTGACATAACAAAATGGCTGTGGTATATAAAAATATTCATAATGATAATAGGAGGA
TTAGTAGGTTTAAGAATAATTTTTACTGTGCTTTCTATAGTGAATAGAGTTAGGCAGGGATACTCACCATTAT
CATTCCAGACCCGCCTCCCAGCCCAGAGGGGACCCGACAGGCCCGAAGGAATCGAAGAAGAAGGTGGAGAGAA

AGACAGAGACAGATCCGGACGATTAGTGACTGGATTCTTAGCACTCATCTGGGACGATCTGCGGAGCCTGTGC
CTCTTCAGCTACCACCGCTTGAGAGACTTACTCTTGATTGTAGCGAGGATTGTGGAACTTCTGGGACGCAGGG
GGTGGGAGGCCCTCAAGTATTGGTGGAATCTCCTACAGTATTGGAGTCAGGAACTAAAGAATAGTGCTGTTAG
TTTGCTTAATACCACAGCTATAGCAGTAGCTGAGGGGACAGATAGGATTTTAGAAGTAGTACAAAGAATTTGG
AGAGCTGTTCTCCACATACCTAGAAGAATAAGACAGGGCTTAGAAAGGCTTTTGCTATAAGATGGGTGGCAAG
TGGTCAAAACGTATGAGTGATGGATGGTCTGTTGTAAGGGAAAGAATGAGACGAGCTGCGCCAGCTGAGCCAG
CAGCAGATGGGGTGGGAGCAGCATCTCGAGACTTGGATAGACATGGAGCAATCACAAGCAGTAATACAGCAAC
TAGTAATGCTGCTTGTGCATGGGTAGAAGCACAAGAGGAGGAGGAAGAGGTGGGTTTTCCAGTCAGGCCTCAG
GTACCTGTAAGACCAATGACTTACAAGGCAGCTGTAGATCTTAGCCACTTTTTAAAAGAAAAGGGGGGACTGG
AAGGGCTAGTTTACTCCCAAAAAAGGCAAGATATCCTTGATCTGTGGGTCTACCACACACAGGGCTTCTTCCC
TGACTGGCAGAATTACACACCAGGGCCAGGGATCAGATATCCACTGACCCTTGGATGGTGCTTCAAGCTAGTA
CCAGTTGAGCCAGAGCAGGTAGAAGAGGCCAATGAAGGAGAGAACAACAGTCTATTACACCCTATGAGCCTGC
ATGGGATGGATGACCCGGAGCATGAAGTATTAGTGTGGAGGTTTGACAGCCGCCTAGCATTTCATCACATGGC
CCGAGAGCTGCATCCGGAATATTACAAAGACTGCTGACATTGAGCTTTCTACAAGGGACTTTCCGCTGGGGAC
TTTCCAGGGAGGTGTGGCCGGGGCGGGACGGGGAGTGGCGAACCCTCAGATGCTGCATATAAGCAGCTGCTTT
CTGCTTGTACTGGGTGTCTCTGGTTAGACCAGATCAGAGCCTGGGAGCTCTCTGGCTAACTAGGGAACCCACT
GCTTAAGCCTCAATAAAGCTTGCCT

>V-C6 HIV-1 genome, derived from RNA genomic sequence
AGCAGTGGCGCCCGAACAGGGACTTGAAAGCGAAAGTAGAACCAGAGGAGGTCTCTCGACGCAGGACTCGGCT
TGCTGAAGCGCGCACAGCAAGAGGCGAGGGGCGGCGACTGGTGAGTACGCCAAAACTATAATTTTGACTAGCG
AAGGCTAGAAGGAGAGAGATGGGTGCGAGAGCGTCAGTATTAAGCGGGGGAAAATTAGATAAATGGGAAAAAA
TTCGGTTAAGGCCAGGGGGAAAGAAAACATATAAATTAAAACATATAGTATGGGCAAGCAGGGAGCTAGAACG
ATTCGCAGTTAATCCTGGCCTTTTAGAGACATCAGAAGGCTGTAGACAGATCCTGGGACAGCTACAACCATCC
CTTCAGACAGGATCAGAAGAACTTAAATCATTATATAATGCAGTAGCAACCCTCTATTGTGTGCATCAAAATA
TAGATGTAAGAGACACCAAGGAAGCTTTAGACAAGATAGAGGAAGAGCAAAACAAAAGTAAGAAAAGGCACA
GCAAGTAGCAGCTGACACAGAAAACAGCAGCAAGGTCAGCCAAAATTATCCTATAGTGCAGAACCTTCAGGGG
CAAATGGTACATCAGGCAATATCACCTAGAACCTTAAATGCATGGGTAAAGGTAGTAGAAGAGAAGGCTTTCA
GCCCAGAAGTAATACCCATGTTTTCAGCATTATCAGAAGGAGCCACCCCACAAGATTTAAACACCATGTTAAA
CACAGTGGGGGGACATCAAGCAGCTATGCAAATGTTAAAAGAGACCATCAATGAGGAAGCCGCAGAATGGGAT
AGATTGCATCCAGTGCATGCAGGGCCTATTGCACCAGGCCAGATGAGAGAACCAAGGGGAAGTGACATAGCAG
GAACTACTAGTACCCTTCAGGAACAAATAGGATGGATGACACATAATCCACCTATCCCAGTAGGAGAAATCTA
TAAAAGATGGATAATCATGGGATTAAATAAAATAGTAAGAATGTATAGTCCTACCAGCATTCTGGACATAAGA
CAAGGACCAAAAGAACCCTTTAGAGATTATGTAGACCGGTTCTATAAAACTCTAAGAGCCGAGCAAGCTTCAC
AGGAGGTAAAAAATTGGATGACAGAAACTTTGTTGGTCCAAAATGCGAACCCAGATTGTAAGACTATTTTAAA
AGCATTAGGGCCAGCAGCTACATTAGAAGAAATGATGACAGCATGTCAGGGAGTGGGGGGACCCGGCCATAAA
GCAAGAGTTTTGGCTGAAGCAATGAGCCAAGTAACAAATTCAGCTACCATAATGATGCAGAAAGGCAATTTTA
GGAACCAAAGAAAAACTGTTAAGTGTTTCAATTGTGGCAAAGAAGGGCACATAGCCAGAAATTGCAGGGCCCC
TAGGAAAAAGGGCTGTTGGAAATGTGGAAAGGAAGGACACCAAATGAAAGATTGTACTGAGAGACAGGCTAAT
TTTTTAGGGAAGATCTGGCCTTCCCACAAGGGAAGGCCAGGGAATTTTCTTCAGAGCAGACCAGAGCCAACAG
CCCCACCAGAAGAGAGCTTCAGGTTTGGGGAAGAGACAACGAATCCCCCTCAGAAGCAGGAGCCGATAGACAA
GGAAATGTATCCTTTAACTTCCCTCAGATCACTCTTTGGCAACGACCCCTCGTCACAGTAAAGATAGGGGGGC
AACTAAAGGAAGCTCTATTAGATACAGGAGCAGATGATACAGTACTAGAAGAAATAAGTTTGCCAGGAAGATG
GAAACCAAAAATGATAGGGGGAATTGGAGGTTTTATCAAAGTAAGACAGTATGATCAGATACCCATAGAAATC
TGCGGACATAAAGCTATAGGTACAGTATTAGTAGGACCTACACCTGTCAACATAATTGGAAGAAATCTGTTGA
CTCAGATTGGCTGCACTTTAAATTTTCCCATTAGTCCTATTGAAACTGTACCAGTAAAATTAAAGCCAGGAAT
GGATGGCCCAAGAGTTAAACAATGGCCATTGACAGAAGAAAAAATAAAAGCATTAGTAGAAATTTGTACAGAG
ATGGAAAAGGAAGGAAAAATTTCAAAAATTGGGCCTGAAAATCCATACAATACTCCAGTATTTGCCATAAAGA
AAAAAGACAGTACTAAATGGAGAAAATTAGTAGATTTCAGAGAACTTAATAAGAGAACTCAAGACTTCTGGGA
AGTTCAATTAGGAATACCACATCCCGCAGGGTTAAAAAAGAAAAAATCAGTAACAGTACTGGATGTGGGTGAT
GCATATTTTTCAGTTCCCTTAGATAAAGACTTTAGGAAGTATACTGCATTTACCATACCTAGTACAAACAATG
AGACACCAGGGATTAGATATCAGTACAACGTGCTTCCACAGGGATGGAAAGGATCACCAGCAATATTCCAAAG
CAGCATGACAAAGATCTTAGAACCTTTTAGAAAACAAAATCCAGACATGGTTATCTATCAATACATGGATGAT
TTGTATGTAGGATCTGACTTAGAAATAGGGCAACATAGAACAAAAATAGAGGAACTGAGACAACATCTGTTGA
AGTGGGGATTTACCACACCAGACAAAAAACATCAGAAAGAACCTCCATTCCTTTGGATGGGTTATGAACTCCA
TCCTGATAAATGGACAGTACAGCCTATAATGCTGCCAGAAAAAGATAGCTGGACTGTCAATGACATACAGAAG
TTAGTGGGAAAATTGAATTGGGCAAGCCAGATTTATCCAGGGATTAAAGTAAGACAATTATGTAAACTCCTTA
GGGGAACCAAAGCACTAACAGACGTAGTACAACTAACAGAAGAAGCAGAGCTAGAACTGGCAGAAAACAGGGA
GATTCTAAAAGAACCAGTACATGGAGTGTATTATGACCCATCAAAAGACTTAATAGCAGAAATACAGAAGCAA
GGGTATGGCCAATGGACATATCAAATATATCAAGAGCCATTTAAAAATCTGAAAACAGGAAAGTATGCAAAAA
TGAGGGGTGCCCACACTAATGATGTAAAACAGTTAACAGAGGCAGTGCAAAAAATAGCCACAGAGAGCATAGT
AATATGGGGAAAGATTCCTAAATTTAAATTACCCATACAAAAAGAAACATGGGAAGCATGGTGGATAGAGTAT
TGGCAAGCTACCTGGATTCCTGAGTGGGAGTTTGTCAATACCCCTCCCTTAGTGAAATTATGGTACCAGTTAG
AGAAAGAACCCATAGTAGGAGCAGAAACTTTCTATGTAGATGGGGCAGCTAATAGGGAGACTAAATTAGGAAA
AGCAGGATATGTTACTGACAGAGGAAGACAAAAGGTTGTCCCCCTAGCGGACACAACAAATCAGAAGACTGAG
TTACAAGCAATTCATCTAGCTTTGCAGGATTCGGGATTAGAGGTAAACATAGTAACAGACTCACAATATGCAT
TAGGAATCATTCAAGCACAACCAGATAAGAGTGAATCAGAGTTAGTCAGTCAAATAATAGAGCAGTTAATAAA
AAAGGAAAAGGTCTACCTGGCATGGGTACCAGCACACAAAGGAATTGGAGGAAATGAACAAGTAGATAAATTA
GTCAGTGCTGGAATTAGGAAAATACTATTTTTAGATGGAATAGATAAGGCCCAAGAAGACCATGAGAAATATC
ACAGTAATTGGAGAGCAATGGCTAATGAATTTAACCTGCCACCTGTAGTAGCAAAAGAAATAGTAGCCAGCTG
TGATAAATGTCAGCTAAAAGGAGAAGCCATGCATGGACAAGTAGACTGTAGTCCAGGAATATGGCAGCTAGAT
TGCACACACTTAGAAGGAAAAATTATCCTGGTAGCAGTTCATGTAGCCAGTGGATATATAGAAGCAGAAGTTA

TTCCAGCAGAGACAGGGCAAGAAACAGCATACTTTATCTTAAAATTAGCAGGAAGATGGCCAGTAAAAACAAT
ACATACAGACAATGGCGGCAATTTCATCAGCAATACGGTTAAGGCCGCCTGTTGGTGGGCAGGGATCAAGCAG
GAATTTGGCATTCCCTACAATCCCCAAAGTCAAGGAGTAGTAGAATCTATGAATAAAGAATTAAAGAAAATTA
TAGGACAGGTAAGAGATCAGGCCGAACATCTTAAGACAGCAGTACAAATGGCAGTATTCATCCACAATTTTAA
AAGAAAAGGGGGGATTGGGGGGTACAGTGCAGGAGAAAGAATAGTAGACATAATAGCAACAGACATACAAACT
AAAGAATTACAAAACCAAATTACAAAACTTTCAAAATTTCGGGTTTATTACAGGGACAGCAGAGATCCACTGT
GGAAAGGACCAGCAAAGCTCCTCTGGAAAGGTGAAGGGGCAGTAGTAATACAAGATAATAGTGAGATAAAAGT
AGTGCCAAGAAGAAAAGCAAAGGTCCTTAGGGATTATGGAAAACAGATGGCAGGTGATGATTGTGTGGCAAGT
AGACAGGATGAGGATTAGAACATGGAAAAGTTTAGTAAAACACCATATGTATGTTTCAGGGAAAGCTAGGAAA
TGGTTCTATAGACATCACTATGAAAGCACTCATCCAAGAATAAGTTCAGAAGTACACATCCCACTAGGGGATG
CTGAATTAGTAATAACAACATATTGGGGTCTGCATACAGGAGAAAGAGACTGGCATTTGGGTCAGGGAGTCTC
CATAGAATGGAGGAAAAAGAGATATAGCACACAAGTAGACCCTAACCTAGCAGACCAACTAATTCATCTGTAT
TACTTTGATTGTTTTTCAGAATCTGCTATAAGAAATGCCATATTAGGACGTATAGTTAGACCTAGGTGTGATT
ATCAAGCAGGACATAACAAGGTAGGATCCCTACAGTACTTGGCATTAGCAGCATTAATAACACCAAAAAAGAT
AAAACCACCTTTGCCTAGTGTTAGGAAACTGACAGAGGATAGATGGAACAAGCCCCAGGAGACCAAGGGCCAC
AGAGGGAGCCATACAATGAATGGACACTAGAGCTTTTAGAGGAACTTAAAAGTGAAGCTGTTAAACATTTTCC
TAGGCCATGGCTCCATAGCTTAGGACAATATATCTATGAAACTTATGGGGATACTTGGGCAGGAGTGGAAGCC
ATTATAAGAATTCTGCAACAAATGCTGTTTATTCATTTCAGAATTGGATGTCATCATAGCAGAATAGGTATTC
TTCGACGAAGGGGAACAAGGAATGGAGCCAGTAGATCCTAGACTAGAGCCCTGGAAGCATCCAGGGAGCCAGC
CTAGGACTGCTTGTAGCAAATGCTATTGTAAAAAGTGTTGCTTTCATTGCCAAGTTTGTTTCATAACAAAAGG
CTTAGGCATCTCCTATGGCAGGAAGAAGCGGAGACAGCGACGAAGAACTCATCAAGACAGTCAGACTCATCAA
GTTTCTCTACCAAAGCAGTAAGTAGTATATGTAATGCAATCTGTACAAATATTAGCAATAGTAGCATTAGTAG
TAGGAGCAATAATAGCAATAGTTGTGTGGACCATAGTGTTAATAGAATATAGGAAGATATTAAGACAAAGAAA
AATAGACAGGTTAATTGATAGAATAATAGAAAGAGCAGAAGACAGTGGCAATGAGAGTGAAGGAGATCAGGAG
GAATTGTCAGCTCTTGTGGAGATGGGGCACCATGCTCCTTGGGATGTTAATGATCTGTAGTGCTACAGAACAG
TTATGGGTCACAGTGTATTATGGGGTACCTGTGTGGAAAGAAGCAACCACCACTCTATTTTGTGCGTCAGATG
CTAAAGCATATGACACAGAGGCACATAATGTTTGGGCCACACATGCCTGTGTACCCACAGACCCTAACCCACA
AGAAGTAGTATTGGAAAATGTGACAGAAAATTTTAACATGTGGAAAAATAAGATGGTAGAACAGATGCAGGAG
GATATAATCAGTTTATGGGATGAAAGCCTAAAGCCATGTGTAAAACTAACCCCACTCTGTGTCACTTTAAATT
GCACTCATTTGCAGAATGTTACTTACAATAGTACTTGGGAAGGGATGAAGGAAGAAATAAAAAACTGCTCCTT
CAATATCACCACAGGCATAGGAAATAAGATGAAGAAAGAATATGCTTTTTTCTATAATCTTGATATAAGACCA
ATAGAAAATGATAATACAGATAATACAAGCTATATGTTAATAAATTGTAACACCTCAGTCATTAAACAGGCCT
GTCCAAAGGTATCCTTTGAACCAGTTCCCATACATTATTGTGCCCCGGCTGGTTTTGCGATTCTAAAGTGTAA
TGATAAGAAATTCAATGGAACGGGACCATGTACAAATGTCAGCACAGTACAATGTACACATGGAATTAGGCCA
GTAGTGTCAACTCAACTGCTGCTAAATGGCAGTCTAGCAGAAGAAGAGGTAGTAATTAGATCTGAAAATTTCA
CGAACAATGCTAAAATCATAATAGTACAGCTGAACGAATCTGTAGAAATTAATTGTACAAGACCCAACAACAA
TACAAGAAAAAGGATAACTATGGGACCGGGGAGAGTACTTTATGCAACAGGAGAAATAGTAGGAGATATAAGA
CAAGCACATTGTAACATCAGTGGAGCAAAATGGGAAAACACTTTAAAACAGATAGTTAAAAAATTACAAGAAC
AATTTGAGAATAAAACAATAATCTTTAATCAATCCTCAGGAGGGGACCCAGAAATTGTAATGCATAGTTTTAA
TTGTGGAGGGGAATTTTTCTACTGTAATACAACACAGCTGTTTAATAGTACTTGGAATAGTACTGGGAATAAA
ATTGAAGGGTCACCAAACATTACAGGAAATATCACACTCCCATGCAGAATAAAACAAATTATAAACAGGTGGC
AGGAAGTAGGAAAAGCGATGTATGCCCCTCCCATCAGAGGACTAATTAGATGTTCGTCAAATATTACAGGGCT
GCTAATAACAAGAGATGGTGGTAATGGGTCCGAGACCAAAAATAAGTCCGAGACCTTCAGACCTGGAGGAGGA
GATATGAGGGACAATTGGAGAAGTGAATTATATAAATATAAAGTAGTAAGAATTGAGCCATTAGGAGTAGCAC
CCACCAAGGCAAAGAGAAGAGTGGTGCAGAGAGAAAAAAGAGCAGTGGGAACGCTAGGAGCTATGTTCCTTGG
GTTCTTGGGAGCAGCAGGAAGCACTATGGGCGCAGCGTCATTGACGCTGACGGTACAGGCCAGACTATTATTG
TCTGGTATAGTGCAACAGCAGAACAATTTGCTGAGGGCTATTGAGGCGCAACAGCATCTGTTGCAACTCACAG
TCTGGGGCATCAAGCAGCTTCAGGCAAGAGTCCTGGCTGTGGAAAGATACCTAAAGGATCAACAGCTCCTAGG
GATTTGGGGTTGCTCTGGAAAACTCATCTGCACCACTGCTGTGCCTTGGAATACTAGTTGGAGTAATAAATCT
TATGAGGAGATTTGGAATAACATGACATGGATGCAGTGGGAGAGAGAAATTGACAATTACACACAATTAATAT
ACACCTTAATTGAAGATTCGCAAAACCAACAAGAAAAGAATGAACTAGAATTATTAGAATTGGATAAGTGGGC
AAGTTTGTGGAATTGGTTTGACATAACAAAATGGCTGTGGTATATAAAAATATTCATAATGATAATAGGAGGA
TTAGTAGGTTTAAGAATAATTTTTACTGTGCTTTCTATAGTGAATAGAGTTAGGCAGGGATACTCACCATTAT
CATTCCAGACCCGCCTCCCAGCCCAGAGGGGACCCGACAGGCCCGAAGGAATCGAAGAAGAAGGTGGAGAGAA

AGACAGAGACAGATCCGGACGATTAGTGACTGGATTCTTAGCACTCATCTGGGACGATCTGCGGAGCCTGTGC
CTCTTCAGCTACCACCGCTTGAGAGACTTACTCTTGATTGTAGCGAGGATTGTGGAACTTCTGGGACGCAGGG
GGTGGGAGGCCCTCAAGTATTGGTGGAATCTCCTACAGTATTGGAGTCAGGAACTAAAGAATAGTGCTGTTAG
TTTGCTTAATACCACAGCTATAGCAGTAGCTGAGGGGACAGATAGGATTTTAGAAGTAGTACAAAGAATTTGG
AGAGCTGTTCTCCACATACCTAGAAGAATAAGACAGGGCTTAGAAAGGCTTTTGCTATAAGATGGGTGGCAAG
TGGTCAAAACGTATGAGTGATGGATGGTCTGTTGTAAGGGAAAGAATGAGACGAGCTGCGCCAGCTGAGCCAG
CAGCAGATGGGGTGGGAGCAGCATCTCGAGACTTGGATAGACATGGAGCAATCACAAGCAGTAATACAGCAAC
TAGTAATGCTGCTTGTGCATGGGTAGAAGCACAAGAGGAGGAGGAAGAGGTGGGTTTTCCAGTCAGGCCTCAG
GTACCTGTAAGACCAATGACTTACAAGGCAGCTGTAGATCTTAGCCACTTTTTAAAAGAAAAGGGGGGACTGG
AAGGGCTAGTTTACTCCCAAAAAAGGCAAGATATCCTTGATCTGTGGGTCTACCACACACAGGGCTTCTTCCC
TGACTGGCAGAATTACACACCAGGGCCAGGGATCAGATATCCACTGACCCTTGGATGGTGCTTCAAGCTAGTA
CCAGTTGAGCCAGAGCAGGTAGAAGAGGCCAATGAAGGAGAGAACAACAGTCTATTACACCCTATGAGCCTGC
ATGGGATGGATGACCCGGAGCATGAAGTATTAGTGTGGAGGTTTGACAGCCGCCTAGCATTTCATCACATGGC
CCGAGAGCTGCATCCGGAATATTACAAAGACTGCTGACATTGAGCTTTCTACAAGGGACTTTCCGCTGGGGAC
TTTCCAGGGAGGTGTGGCCGGGGCGGACGGGGAGTGGCGAACCCTCAGATGCTGCATATAAGCAGCTGCTTT
CTGCTTGTACTGGGTGTCTCTGGTTAGACCAGATCAGAGCCTGGGAGCTCTCTGGCTAACTAGGGAACCCACT
GCTTAAGCCTCAATAAAGCTTGCCT

>X-11D HIV-1 genome, derived from RNA genomic sequence
TAGCAGTGGCGCCCGAACAGGGACTTGAAGTTAATAGGGACTCGAAAGCGAAAGTTCCAGAGAAGCTCTCTCG
ACGCAGGGACTCGACTTGCTGAAGTGCACGCAGCAAGAGGCGAGAGCGGCGACTGGTGAGTACGCCAAATTTC
GACTAGCGGAGGCTAGAAGGAGAGAGATGGGTGCGAGAGCGTCGATACTAAGTGGGGGAAAATTAGATGCATG
GGAGAAAATTCGGTTAAGGCCAGGGGGAAAGAAACAATATCGACTAAAACATTTAGTATGGGCAAGCAGGGAG
TTGGAAAGATTTGCACTTAATCCTAGTCTTTTAGAAACAGCAGAAGGATGCCAACAACTGATAGAACAGTTAC
AATCAACTCTCAGGACAGGATCAGAAGAACTTAAATCATTATTTAATACAATAGCAACCCTTTGGTGCGTACA
TCAAAAGATAGACATAAAAGACACCAAGGAAGCCTTAGATAAAATAGAGGAAGAACAAAATAAGAGCAAGCAA
AAGGCACAGCAAAAGACACAGCAGGCAGCAGCTGCCGCAGGAAGCAGCAGCCAAAATTACCCTATAGTGCAAA
ATGCACAAGGGCAAATGATACATCAGGCCATGTCACCTAGAACTTTGAACGCATGGGTGAAGGTAGTAGAGGA
GAAGGCTTTCAGCCCAGAAGTAATACCCATGTTTACAGCATTATCAGAAGGAGCCACCCCACAAGACTTAAAT
ATGATGCTAAACATAGTGGGGGGACACCAGGCAGCAATGCAGATGTTAAAAGATACCATCAATGAGGAAGCTG
CAGAATGGGACAGGACACATCCTGTACATGCAGGGCCTGCTCCACCAGGCCAGATGAGAGAACCAAGGGGAAG
TGACATAGCAGGAACTACTAGTACCCTTCAAGAACAAATAGGATGGATGACAAGCAATCCACCTATCCCAGTG
GGAGAAATCTATAAAAGATGGATAGTCCTGGGATTAAATAAAATAGTAAGAATGTATAGCCCTGTCAGCATTT
TGGATATAAGACAAGGGCCAAAAGAACCCTTTAGAGACTATGTAGATAGGTTCTTTAAAACTTTAAGAGCTGA
ACAAGCTACACAGGAGGTAAAAAACTGGATGACAGAAACCTTGCTGGTCCAAAATGCGAATCCAGATTGTAAG
ACCATTCTAAGAGGATTAGGACCAGGGGCTACATTAGAAGAAATGATGACAGCATGTCAGGGAGTGGGAGGAC
CTGGCCATAAAGCAAGGGTTTTGGCTGAGGCTATGAGTCAAGCGCAACAGTCCAACATAATGATGCAGAAGGG
AAACTTTAGGGGCCAGAGAACAATAAAGTGTTTCAATTGTGGCAAAGAAGGACACCTAGCCAGAAACTGCAAG
GCCCCTAGGAAAAGGGGTTGTTGGAAATGTGGTAAGGAAGGACACCAAATGAAAGACTGTACTGAAAGACAGG
CTAATTTTTTAGGGAAAATTTGGCCTTCCAACAAGGGGAGGCCAGGAAATTTTCCTCAGAGCAGACCGGAACC
AACGGCCCCACCACTAGAGAACTTTGGAGTGGGGGAAGAGATACCCCCCCTTCAGAAGCAGGAACCCTCCTCT
CCGAAACAGGAACCGGGGGACAAGGGACTATATCCTCCTTTAGCCTCCCTCAAATCACTCTTTGGCAACGACC
AGTAGTCACAGTAAGAATAGAGGGAGAGGTAATAGAAGCCCTATTAGACACAGGGGCAGATGATACAGTAATA
GAAAAGATAAATTTACCAGGAAAATGGAAACCAAAAATGATAGGGGGAATTGGAGGCTTTATCAAAGTAAGAC
AGTATGATCAGATAAGTATAGAAATTTGTGGAAAAAGGGCCATAGGTACAGTATTAGTAGGACCTACACCTGT
CAACATAATTGGACGAAATATGTTGACTCAGATTGGTTGTACTTTAAATTTTCCAATAAGTCCTATTGAAACT
GTACCAGTAAAATTAAAGCCAGGAATGGATGGGCCAAAGGTTAAACAATGGCCATTGACAGAAGAAAAAATAA
AAGCATTAACAGAAATTTGTACAGATATGGAAAAGGAAGGAAAAATTTCAAGAATTGGGCCTGAAAATCCATA
CAATACTCCAGTATTTGCCATAAAGAAAAAAGATAGTACTAAATGGAGAAAATTAGTAGATTTCAGAGAACTC
AATAAGAGAACTCAAGACTTCTGGGAGGTCCAATTAGGAATACCTCATCCCGCAGGGTTAAAAAAGAAAAAAT
CAGTAACAGTATTAGATGTGGGGGATGCATATTTTTCAGTTCCCTTAGATAAGGACTTTAGAAAGTATACTGC
CTTCACTGTACCTAGTATAAATAATGAGACACCAGGAATTAGATATCAGTACAACGTGCTCCCACAGGGATGG
AAAGGATCACCAGCAATATTTCAGGCAAGTATGATAAAAATATTAGAGCCCTATAGAATAAATAATCCAGAGG
TGGTGATCTATCAATATATGGATGATTTATATGTAGGATCTGACTTAGAGATAGGGCAGCATAGAGCAAAAAT
AGAGGAGTTGAGAGCACATCTATTGAAGTGGGGATTTACCACACCAGACAAAAAGCATCAGAAAGAACCTCCA
TTTCTTTGGATGGGATATGAACTCCATCCTGACAAATGGACAGTCCAGCCTATACAACTACCGGATAAAGACC
ACTGGACTGTCAATGATATACAGAAATTAGTGGGAAAACTAAATTGGGCAAGTCAGATTTATGCAGGAATTAA
AGTAAGACAACTGTGTAAACTCCTCAGGGGAGCCAAAGCACTAACAGATATAGTATCAATGACTGAGGAAGCA
GAATTGGAATTGGCAGAGAACAGGGAAATTTTAAAAGAACCTGTACATGGGGTGTATTATGACCCAACAAAAG
ACTTAGTAGCAGAAGTACAGAAACAAGGGCAAGGCCAATGGACATATCAAATTTATCAAGAGCCATTTAAAAA
TCTAAAAACAGGAAAATATGCAAAAAGGAAGTCTGCCCACACTAATGATGTAAAACAGTTAGCAGAGGTAGTG
CAAAAAATAGCTGTAGAAAGCATAGTAATTTGGGGGAAGACCCCTAAATTTAGACTACCCATACAAAGAGAAA
CATGGGAAACATGGTGGACAGAGTATTGGCAGGCTACCTGGATACCTGACTGGGAGTTTGTCAATACCCCTCC
TTTAGTAAAATTATGGTACCAGTTAGAAAAAGACCCCATAGTAGGGGCAGAAACTTTCTATGTAGATGGGGCA
GCCAATAGGGAAACTAAGCTAGGAAAAGCAGGGTATGTCACAGACAGAGGAAGACAAAAGGTTGTCTCCCTAA
CTGAGACAACAAATCAAAAGACTGAATTACAGGCAATATATCTAGCCTTGCAAGATTCAGGATCAGAAGTAAA
TATAGTGACAGACTCACAATATGCATTGGGAATTATTCAGGCACAACCAGACAGAAGTGAATCAGAATTAGTT
AATCAAGTAATAGAGAAGCTAATAGAAAAGGACAAGGTCTACCTGTCATGGGTACCAGCACATAAAGGGATTG
GAGGAAATGAACAAGTAGATAAATTGGTCAGTAATGGAATCAGAAGAGTACTATTTTTAGATGGCATAGATAA
AGCCCAAGAAGATCACGAAAAATATCACAGCAATTGGAGAACAATGGCTAGTGATTTTAATCTGCCACCTATA
GTAGCAAAAGAAATAGTGGCCAGCTGTGATAAATGTCAGCTGAAAGGGGAAGCCATGCATGGACAAGTAGACT
GTAGTCCAGGAATATGGCAATTAGATTGTACACATTTAGAAGGAAAAATTATCCTGGTAGCAGTCCATGTAGC

CAGTGGCTATATAGAAGCAGAAGTTATCCCAGCAGAAACAGGATCGGAGACAGCATACTTTATATTAAAATTA
GCAGGAAGATGGCCAGTGAAAGTAATACACACAGACAATGGCCCCAATTTTATCAGTGCTGCAGTAAAGGCAG
CATGTTGGTGGGCAAATGTCACACAAGAATTTGGAATTCCCTACAATCCCCAAAGCCAAGGAGTAGTGGAATC
TATGAATAAAGAACTAAAGAAAATTATAGGACAGGTCAGGGATCAAGCTGAACATCTTAAGACAGCAGTACAG
ATGGCAGTATTCATTCACAATTTTAAAAGAAAAGGGGGGATTGGGGGGTACAGTGCAGGGGAAAGAATAATAG
ACATAATAGCATCAGATATACAAACTAAAGAACTACAAAAACAAATTACAAAAATTCAAAATTTTCGGGTTTA
TTACAGGGACAGCAGAGACCCCATTTGGAAAGGACCAGCAAAACTACTCTGGAAAGGTGAAGGGGCAGTAGTA
ATACAGGACAATAGTGATATAAAAGTAGTACCAAGAAGAAAAGCAAAAATCATTAAGGATTATGGAAAACAGA
TGGCAGGTGATGATTGTGTGGCAGGTAGACAGGATGAGGATTAGAACATGGAACAGTTTAGTAAAACATCATA
TGTATGTCTCTAAGAAAGCTAAGGGTTGGTTTTATAGACATCACTATGAAAGTAGGCATCCAAAAGTGAGTTC
AGAAGTACACATCCCATTAGGGGATGCTATGTTAGTAGTAAGAACATATTGGGGTCTGCTTACAGGAGAAAGA
GATTGGCACTTGGGTCATGGGGTCTCCATAGAATGGAGGCAGAAAAGATATAGCACACAAATAGATCCTGAAC
TAGCAGACCAACTAATTCACTCGCATTATTTTGACTGTTTTACAGACTCTGCTATAAGAAAAGCCACATTAGG
ACAAATAGTTAGACCTAAGTGTGAATATCAAGCAGGACATAATAAGGTAGGATCGCTACAATATTTGGCACTA
AAAGCATTAGTAACACCAGAAAGGACAAAGCCACCTTTACCTAGTGTTAAGAAGTTAACAGAAGACAGATGGA
ACAAGTCCCACAAGACCAGGGGCCACAGAGGGAGCCGCTCAATGAATGGACACTAGAACTGTTAGAAGAGCTT
AAGGAAGAAGCTGTTAGACATTTTCCTAGGCCGTGGCTCCATGGATTAGGACAACATATCTACAACACATATG
GGGATACTTGGGAAGGGGTTGTAGCTATAATAAGAATTTTGCAACAACTACTGTTTATTCATTTCAGAATTGG
GTGTCAACATAGCAGAATAGGCATTATTCCAGGGAGAAGAGGCAGGAATGGAGCTGGTAGATCCTAGCCTAGA
GCCATGGAACCAACCGGGAAGTCAGCCTACAACTGCTTGTAACAATTGTTACTGTAAAATATGCTGCTGGCAT
TGCCAATTATGCTTTTTGAAAAAGGGCTTAGGCATCTCCTATGGCAGGAAGAAGCGGAGACCCCGACGAAGAA
CTCCTCAAAGCCGTCAAGATCATCAAAATCCTGTACCAAAGCAGTGAGTAGTAATAATTAGTATATGTAATGA
AATCTTTAGGAATAGCTGCAATAGTAGGATTAGTAGTAGCATTCATAGCAGCCATAGTTGTGTGGACCATAGT
GTTTATTGAATATAGAAAAATAAGGAAACAGAAGAAAATAGACAAGATACTTGATAGAATAAGAGAAAGAGCA
GAAGACAGTGGCAATGAGAGTGATGGGGACACAGAAGAATTATACACTCTTATGGAGGTGGGGTATGATAATA
TTTTGGATAATGATAATTTGTAATGCTAAAGACTTGTGGGTCACAGTCTATTATGGGGTACCAGTGTGGAGAG
ACGCAGAGACCACCCTATTTTGTGCATCAGATGCCAAAGCATATGATTCAGAAGTACATAATGTCTGGGCTAC
ACATGCCTGTGTACCCACAGACCCTAACCCACAAGAAATACATTTGAAAAATGTAACAGAAAATTTTAACATG
TGGAAAAATACCATGGTGGAGCAGATGCATGAAGATATAATCAGTCTCTGGGACCAAAGCCTAAAGCCATGTG
TAAAATTAACCCCGCTCTGCGTTACTTTAAATTGTAGTGACTATAACTCCACCAGCTATGATAAATCCACCAA
CAACTCTGAAATGCAAGAAATAAAAAACTGCTCTTTCAATATAACCACAGAATTAAGAGATAAGAAACAGAAA
ATGTATGCACTTTTTTATAAACTTGATGTAAAACAACTTGATAATGATAATCAGACGTATAGTTTAATAAATT
GTAACACCTCAACCATTACACAGGCTTGTCCAAAGGTATCCTTTGAGCCAATTCCCATACATTATTGTGCCCC
AGCTGGTTTTGCAATTCTAAAGTGTAAGGATAAGATGTTCAATGGAACAGGGCCATGCAAGAATGTCAGCACA
GTACAATGCACACATGGAATCAAGCCAGTAGTATCAACTCAACTGCTGTTAAATGGCAGTCTAGCAGAAGAAG
AGGTAGTGATTAGATCTGAGAATATCACAAACAATGCCAAGACCATAATAGTACAGTTGAATAAGCCTGTAAG
AATTAATTGTACCAGAACTAGTAACAATACAAGAAAAAGTGTACGTATAGGACCAGGGCAAACATACTATGCA
ACAGGTGAAATAATAGGAGATATAAGAAAAGCACATTGTAATGTCAGTAAAACAGAATGGGATAAGGCTTTAC
AACAGGTAGCCACACAATTAAAGAAGCATTTCAATACCACAATAATCTTTAATAATGCCTCAGGAGGAGATCT
AGAAATCACAACACATAGTTTTAATTGTAGAGGAGAATTTTTTTATTGCAATACAACAAACCTGTATAATAGC
ACTTGGGATAATAGCACTACCAGTACAAATATCACAGGGTCAAATGATACTATAACTCTCCAATGCAGACTAA
AGCAATTTATAAAAATGTGGCAGAGAGTAGGACAAGCAATGTATGCCCCTCCTATCCAAGGAGAAATAAGGTG
TGATTCAAACATTACGGGACTATTATTAACAAGAGATGGAGGGAATAATGGGACAAATGAGACCTTTAGGCCT
GGAGGAGGAGATATGAGGGACAATTGGAGAAGTGAATTATATAAGTATAAAGTAGTAAAAATTGAACCAATAG
GTGTGGCACCAACCAGGGCAAAAAGAAGAATGGTGGAGAGAGAAAAAAGAGCAGTTGGACTGGGAGCTGTTTT
CCTTGGGTTCTTAGGAGCAGCAGGAAGCACTATGGGCGCGGCGTCAATAACGCTGACGGTACAGGCCAGGCAA
TTATTGTCTGGTATAGTGCAACAGCAGAGCAATTTGCTGAAGGCTATAGAGGCTCAACAGCAAATGTTGAGAC
TCACGGTCTGGGGCATTAAGCAGCTCCAGGCAAGAGTCCTGGCTCTGGAAAGATACCTAAAGGATCAACAGCT
CCTAGGAATTTGGGGCTGCTCTGGAAAACTCATCTGCACCACTGGTGTACGCTGGAACTCAAGCTGGAGTAAT
AAAACTTATGAGGACATATGGGATAACATGACCTGGGTGCAATGGGAGAGGGAAATTAGCAATTACACAAACA
CAATATATACTCTACTTGAAGAATCGCAGAACCAGCAGGAAAAAAATGAACAAGACTTATTGGCATTGGAAAA
ATGGACAAATCTGTGGAATTGGTTTAACATATCAAATTGGTTATGGTATATAAAAATATTTATAATGATAGTA
GGAGGTTTAATAGGTTTAAGAATAGTTTTTACTGTGCTTACTATAATAAGGAGAGTTAGGCAGGGATACTCAC
CTTTGTCGTTCCAGACCCTTCCCCTACACCAGAGGGAACCCGACAGGCCCGAAGGAATCGAAGAAGGAGGTGG

CGAGCAAGACAGAGGCAGATCAGTAAGATTAGTGAGCGGATTCTTAGCTCTTGCCTGGGACGACCTACGGAGC
CTGTGCCTTTTCAGCTACCACCAATTGAGAAACTTTGCCTTGATTGCAGCGAGAACTGTGGAACTCCTGGGAC
ACAGCAGTCTCAAGGGACTGAGACTGGGGTGGGAAGCCCTCAAATATCTGTGGAATCTTCTGTCATACTGGGG
TCAGGAACTAAAGAATAGTGCTATTAATTTGTTTGATACCATAGCAATAGCAGTAGCTAACTGGACAGACAGA
GGTATAGAAATAGGACAAAGAGTTGGCAGAGCTATTGGCAACATACCTAGAAGAATCAGACAGGGCCTAGAAA
GAGCTTTGCTATAACATGGGTGGCAAATGGTCAAAAAGCAGCATAGTGGGATGGCCTAAGATTAGGGAAAGGA
TGAGACAAAGCCCTCCAGGAGGCCCTCCAGCAGCGACAGGAACAGGAGCAGTATCCCCAGCAGCAACAGGAAC
AGGAGCAGTATCTCAAGATTTAGCTAGACATGGAGCAATCACAAGCAGTAATACCGCCTCTACTAATTCTGCT
TGTGCCTGGCTGGAAGCACAAGAGGAAGAGGATGAGGTAGGCTTTCCAGTCAAACCACAGGTACCTTTGAGAC
CAATGACTTATAAACTAGCTTTCGATCTCAGCTTCTTTTTAAAAGATAAGGGGGGACTGGAAGGGTTAGTTTG
GTCCCAGAAAAGACAAGATATCCTTGATCTGTGGATGTATCACACACAAGGAATCTTCCCAGATTGGCAGAAC
TACACACCAGGGCCAGGGACTAGATTCCCACTGACCTTTGGGTGGTGCTTCAAACTAGTACCACTAGATCCAG
CAGAGATAGAGGCAGCTAATGAAGGAGAGAACAACAGCTTATTACACCCCATCAGTCAACATGGGATAGAAGA
CGGGGACAGAGAGGTGCTGGTCTGGAGATTTGACAGCAGCCTGGCAAGAGAACACAAAGCCCGAGAGCTGCAT
CCGGAGTTCTATAAAGACTGCTGACACAGAAGTTGCTGACTGGGACTTTCCACTGGGGACTTTCCGGGGAGGT
GTGGTTGGGGAGGAGTTTGGGAGTGGCTAACCCTCAGATGCTGCATATAAGCAGCTGCTTCTCGCCTGTACGT
GGTCTCTCTTGCTAGACCAGATTTGAGCCTGGGAGCTCTCTGGCTAGTTAAGGGACCCACTGCTTAAGCCTCA
ATAAAGCTTGCCT

>X-12D HIV-1 genome, derived from RNA genomic sequence
TAGCAGTGGCGCCCGAACAGGGACTTGAAGTTAATAGGGACTCGAAAGCGAAAGTTCCAGAGAAGCTCTCTCG
ACGCAGGGACTCGGCTTGCTGAAGTGCACGCAGCAAGAGGCGAGAGCGGCGACTGGTGAGTACGCCAAATTTC
GACTAGCGGAGGCTAGAAGGAGAGAGATGGGTGCGAGAGCGTCGATACTAAGTGGGGGAAAATTAGATGCATG
GGAGAAAATTCGGTTAAGGCCAGGGGGAAAGAAAAAATATCGACTAAAACATTTAGTATGGGCAAGCAGGGAG
TTGGAAAGATTTGCACTTAATCCTAGTCTTTTAGAAACAGCAGAAGGATGTCAACAACTGATGGAACAGTTAC
AATCAACTCTCAGGACAGGATCAGAAGAACTTAAATCATTATTTAATACAATAGCAACCCTTTGGTGCGTACA
TCAAAAGATAGACATAAAAGACACCAAGGAAGCCTTAGATAAAATAGAGGAAGAACAAAATAAGAGCAAGCAA
AAGACACAGCAAAAGACACAGCAGGCAGCAGCTGCCGCAGGAAGCAGCAGCCAAAATTACCCTATAGTGCAAA
ATGCACAAGGGCAAATGATACATCAGGCCATGTCACCTAGAACTTTGAACGCATGGGTGAAGGTAGTAGAGGA
GAAGGCTTTCAGCCCAGAAGTAATACCCATGTTTACAGCATTATCAGAAGGAGCCACCCCACAAGACTTAAAT
ATGATGCTAAACATAGTGGGGGGACACCAGGCAGCAATGCAGATGTTAAAAGATACCATCAATGAGGAAGCTG
CAGAATGGGACAGGACACATCCTGTACATGCAGGGCCTGCTCCACCAGGCCAGATAAGAGAACCAAGGGGAAG
TGACATAGCAGGAACTACTAGTACCCTTCAAGAACAAATAGGATGGATGACAAGCAATCCACCTATCCCAGTG
GGAGAAATCTATAAAAGATGGATAGTCCTGGGATTAAATAAAATAGTAAGAATGTATAGCCCTGTCAGCATTT
TGGATATAAGACAAGGGCCAAAAGAACCCTTTAGAGACTATGTAGATAGGTTCTTTAAAACTTTAAGAGCTGA
ACAAGCTACACAGGATGTAAAAAACTGGATGACAGAAACCTTGCTGGTCCAAAATGCGAATCCAGATTGTAAG
ACCATTCTAAGAGCATTAGGACCAGGGGCTACATTAGAAGAAATGATGACAGCATGTCAGGGAGTGGGAGGAC
CTGGCCATAAAGCAAGGGTTTTGGCTGAGGCTATGAGTCAAGCGCAACAGTCCAACATAATGATGCAGAAGGG
AAACTTTAGGGGCCAGAGAACAATAAAGTGTTTCAATTGTGGCAAAGAAGGACACCTAGCCAGAAACTGCAAG
GCCCCTAGGAAAAGGGGTTGTTGGAAATGTGGTAAGGAAGGACACCAAATGAAAGACTGTACTGAAAGACAGG
CTAATTTTTTAGGGAAAATTTGGCCTTCCAACAAGGGGAGGCCAGGAAATTTTCCTCAGAGCAGACCGGAACC
AACGGCCCCACCACTAGAGAACTTTGGAGTGGGGGAAGAGATACCCCCCCCTCAGAAGCAGGAACCCTCCTCT
CCGAAACAGGAACCGGGGGACAAGGGACTATATCCTCCTTTAACCTCCCTCAAATCACTCTTTGGCAACGACC
AGTAGTCACAGTAAGAATAGAGGGAGAGGTAATAGAAGCCCTATTAGACACAGGGGCAGATGATACAGTAATA
GAAAAGATAAATTTACCAGGAAAATGGAAACCAAAAATGATAGGGGGAATTGGAGGCTTTATCAAAGTAAGAC
AGTATGATCAGATAAGTATAGAAATTTGTGGAAAAAGGGCCATAGGTACAGTATTAGTAGGACCTACACCTGT
CAACATAATTGGACGAAATATGTTGACTCAGATTGGTTGTACTTTAAATTTTCCAATAAGTCCTATTGAAACT
GTACCAGTAAAATTAAAGCCAGGAATGGATGGGCCAAAGGTTAAACAATGGCCATTGACAGAAGAAAAAATAA
AAGCATTAACAGAAATTTGTACAGATATGGAAAAGGAAGGAAAAATTTCAAGAATTGGGCCTGAAAATCCATA
CAATACTCCAGTATTTGCCATAAAGAAAAAAGATAGTACTAAATGGAGAAAATTAGTAGATTTCAGAGAACTC
AATAAGAGAACTCAAGACTTCTGGGAGGTCCAATTAGGAATACCTCATCCCGCAGGGTTAAAAAAGAAAAAAT
CAGTAACAGTATTAGATGTGGGGGATGCATATTTTTCAGTTCCCTTAGATAAGGACTTTAGAAAGTATACTGC
CTTCACTATACCTAGTATAAATAATGAGACACCAGGAATTAGATATCAGTACAATGTGCTCCCACAGGGATGG
AAAGGATCACCAGCAATATTTCAGGCAAGTATGACAAAAATATTAGAGCCCTATAGAATAAATAATCCAGAGA
TGGTGATCTATCAATATATGGATGATTTATATGTAGGATCTGACTTAGAGATAGGGCAGCATAGAGCAAAAAT
AGAGGAGTTGAGAGCACATCTATTGAAGTGGGGATTTACCACACCAGACAAAAAGCATCAGAAAGAACCTCCA
TTTCTTTGGATGGGATATGAACTCCATCCTGACAAATGGACAGTCCAGCCTATACAGCTACCGGATAAAGACC
ACTGGACTGTCAATGATATACAGAAATTAGTGGGAAAACTAAATTGGGCAAGTCAGATTTATGCAGGAATTAA
AGTAAAACAACTGTGTAAACTCCTCAGGGGAGCCAAAGCACTAACAGATATAGTACCAATGACTGAGGAAGCA
GAATTGGAATTGGCAGAGAACAGGGAAATTTTAAAAGAACCTGTACATGGAGTGTATTATGACCCAACAAAAG
ACTTAGTAGCAGAAGTACAGAAACAAGGGCAAGGCCAATGGACATATCAAATTTATCAAGAGCCATTTAAAAA
TCTAAAAACAGGAAAATATGCAAAAAGGAAGTCTGCCCACACTAATGATGTAAAACAGTTAGCAGAGGTAGTG
CAAAAAATAGCTGTAGAAAGCATAGTAATTTGGGGGAAGACCCCTAAATTTAGACTACCCATACAAAGAGAAA
CATGGGAAACATGGTGGACAGAGTATTGGCAGGCTACCTGGATACCTGACTGGGAGTTTGTCAATACCCCTCC
TTTAGTAAAATTATGGTACCAGTTAGAGAAAGACCCCATAGTAGGGGCAGAAACTTTCTATGTAGATGGGGCA
GCCAATAGGGAAACTAAGCTAGGAAAAGCAGGGTATGTCACAGACAGAGGAAGACAAAAGGTTGTCTCCCTAA
CTGAGACAACAAATCAAAAGACTGAATTACAGGCAATATATCTAGCCTTGCAAGATTCAGGATCAGAAGTAAA
TATAGTGACAGACTCACAATATGCATTGGGAATTATTCAGGCACAACCAGACAGAAGTGAATCAGAATTAGTT
AATCAAGTAATAGAGAAGCTAATAGAAAAGGACAAGGTCTACCTGTCATGGGTACCAGCACATAAAGGGATTG
GAGGAAATGAACAAGTAGATAAATTGGTCAGTAATGGAATCAGAAGAGTACTATTTTTAGATGGCATAGATAA
AGCCCAAGAAGATCACGAAAGATATCACAGCAATTGGAGAACAATGGCTAGTGATTTTAATCTGCCACCTATA
GTAGCAAAAGAAATAGTGGCCAGCTGTGATAAATGTCAGCTGAAAGGGGAAGCCATACATGGACAAATAGACT
GTAGTCCAGGAATATGGCAATTAGATTGTACACATTTAGAAGGAAAAATTATCCTGGTAGCAGTCCATGTAGC

CAGTGGCTATATAGAAGCAGAAGTTATCCCAGCAGAAACAGGATCGGAGACAGCATACTTTATATTAAAATTA
GCAGGAAGATGGCCAGTGAAAGTAATACACACAGACAATGGCCCCAATTTTATCAGTGCTGCAGTAAAGGCAG
CATGTTGGTGGGCAAATGTCACACAAGAATTTGGAATTCCCTACAATCCCCAAAGCCAAGGAGTAGTGGAATC
TATGAATAAAGAACTAAAGAAAATTATAGGACAGGTCAGGGATCAAGCTGAACATCTTAAGACAGCAGTACAG
ATGGCAGTATTCATTCACAATTTTAAAAGAAAAGGGGGGATTGGGGGGTACAGTGCAGGGGAAAGAATAATAG
ACATAATAGCATCAGATATACAAACTAAAGAACTACAAAAACAAATTACAAAAATTCAAAATTTTCGGGTTTA
TTACAGGGACAGCAGAGACCCCATTTGGAAAGGACCAGCAAAACTACTCTGGAAAGGTGAAGGGGCAGTAGTA
ATACAGGACAATAGTGATATAAAAGTAGTACCAAGAAGAAAAGCAAAAATCATTAAGGATTATGGAAAACAGA
TGGCAGGTGATGATTGTGTGGCAGGTAGACAGGATGAGGATTAGAACATGGAACAGTTTAGTAAAACATCATA
TGTATGTCTCTAGGAAAGCTAAGGGTTGGTTTTATAGACATCACTATGAAAGTAGGCATCCAAAAGTGAGTTC
AGAAGTACACATCCCATTAGGGGATGCTATGTTAGTAGTAAGAACATATTGGGGTCTGCTTACAGGAGAAAGA
GATTGGCACTTGGGTCATGGGGTCTCCATAGAATGGAGGCAGAAAAGATATAGCACACAAATAGATCCTGAAC
TAGCAGACCAACTAATTCACTCGCATTATTTTGACTGTTTTACAGACTCTGCTATAAGAAAAGCCACATTAGG
ACAAATAGTTAGACCTAAGTGTGAATATCAAGCAGGACATAATAAGGTAGGATCGCTACAATATTTGGCACTA
AAAGCATTAGTAACACCAGAAAGGACAAAGCCACCTTTACCTAGTGTTAAGAAGTTAACAGAAGACAGATGGA
ACAAGTCCCACAAGACCAGGGGCCACAGAGGGAGCCGTTCAATGAATGGACACTAGAACTGTTAGAAGAGCTT
AAGGATGAAGCTGTTAGACATTTTCCTAGGCCGTGGCTCCATGGATTAGGACAACATATCTACAACACATATG
GGGATACTTGGGAAGGGGTTGTAGCTATAATAAGAATTTTGCAACAACTACTGTTTATTCATTTCAGAATTGG
GTGTCAACATAGCAGAATAGGCATTATTCCAGGGAGAAGAGGCAGGAATGGAGCTGGTAGATCCTAGCCTAGA
GCCATGGAACCAACCGGGAAGTCAGCCTACAACTGCTTGTAACAATTGTTACTGTAAAATATGCTGCTGGCAT
TGCCAATTATGCTTTCTGAACAAGGGCTTAGGCATCTCCTATGGCAGGAAGAAGCGGAGACCCCGACGAAGAA
CTCCTCAAAGCCGTCAGGATCATCAAAATCCTGTACCAAAGCAGTGAGTAGTAATAATTAGTATATGTGATGA
AATCTTTAGGAATAGCTGCAATAGTAGGATTAGTAGTAGCATTCATAGCAGCCATAGTTGTGTGGACCATAGT
GTTTATTGAATATAGAAAAATAAGGAAACAGAAGAAAATAGACAAGATACTTGATAGAATAAGAGAAAGAGCA
GAAGACAGTGGCAATGAGAGTGATGGGGACACAGAAGAATTATACACTCTTATGGAGGTGGGGTATGATAATA
TTTTGGATAATGATAATTTGTAATGCTAAAGACTTGTGGGTCACAGTCTATTATGGGGTACCAGTGTGGAGAG
ACGCAGAGACCACCCTATTTTGTGCATCAGATGCCAAAGCATATGATTCAGAAGTACATAATGTCTGGGCTAC
ACATGCCTGTGTACCCACAGACCCTAACCCACAAGAAATACATTTGGAAAATGTAACAGAAAATTTTAACATG
TGGAAAAATACCATGGTGGAGCAGATGCATGAAGATATAATCAGTCTCTGGGACCAAAGCCTAAAGCCATGTG
TAAAGTTAACCCCGCTCTGCGTTACTTTAAATTGTAGTGACTATACATATAACTCCACCAGCTATGTTAAATC
CACCAACAACTCTGAAATGCAAGAAATAAAAAACTGCTCTTTCAATATAACCACAGAATTAAGAGATAAGAAA
CAGAAAATGTATGCACTTTTTTATAAACTTGATGTAAAACAACTTGATAATAATAATCAGACGTATAGTTTAA
TAAATTGTAACACCTCAACCATTACACAGGCTTGTCCAAAGGTATCCTTTGAGCCAATTCCCATACATTATTG
TGCCCCAGCTGGTTTTGCAATTCTAAAGTGTAAGGATAAGAGGTTCAATGGAACAGGGCCATGCAAGAATGTC
AGCACAGTACAATGCACACATGGAATCAAGCCAGTAGTATCAACTCAACTGCTGTTAAATGGCAGTCTAGCAG
AAGAAGAGGTAGTGATTAGATCTGAGAATATCACAAACAATGCCAAGACCATAATAGTACAGTTGAATAAGCC
TGTAAGAATTAATTGTACCAGAACTGGTAACAATACAAGAAAAAGTGTACGTATAGGACCAGGGCAAACATAC
TATGCAACAGGTGAAATAATAGGAGATATAAGAAAAGCATATTGTAATGTCAGTAAAACAGAATGGGATGAGG
CTTTACAACAGGTAGTCACACAATTAAAGAAGCATTTCAATACCACAATAATCTTTAATAGTGCCTCAGGAGG
AGATCTAGAAATTACAACACATAGTTTTAATTGTAGAGGAGAATTTTTTTATTGCAATACAACAAACCTGTAT
AATAGCACTTGGAATAATAGCACTTGGAATGGCAATACCAGTACAAATCTCACAGAGTCAAATGATACTATAA
CTCTCCAATGCAGACTAAAGCAATTTATAAAAATGTGGCAGAGAGTAGGACAAGCAATGTATGCCCCTCCTAT
CCAAGGAGAAATAAGGTGTGATTCAAACATTACTGGACTATTATTAACAAGAGATGGAGGGAATAATAGGACA
AATGAGACCTTTAGGCCTGGAGGAGGAGATATGAGGGACAATTGGAGAAGTGAATTATATAAGTATAAAGTAG
TAAAAATTGAACCAATAGGTGTGGCACCAACCAGGGCAAAAAGAAGAATGGTGGAGAGAGAAAAAAGAGCAGT
TGGACTGGGAGCTGTTTTCCTTGGGTTCTTAGGAGCAGCAGGAAGCACTATGGGCGCGGCGTCAATAACGCTG
ACGGTACAGGCCAGGCAATTATTGTCTGGTATAGTGCAACAGCAGAGCAATTTGCTGAAGGCTATAGAGGCTC
AACAGCAAATGTTGAGACTCACGGTCTGGGGCATTAAGCAGCTCCAGGCAAGAGTCCTGGCTCTGGAAAGATA
CCTAAAGGATCAACAGCTCCTAGGAATTTGGGGCTGCTCTGGAAAACTCATCTGCACCACTGGTGTACGCTGG
AACTCAAGCTGGAGTAATAAAACTTATGAGGACATATGGGATAACATGACCTGGGTGCAATGGGAGAGGGAAA
TTAGCAATTACACAAACACAATATATACTCTACTTGAAGAATCGCAGAACCAGCAGGAAAAAAATGAACAAGA
CTTATTGGCATTGGACAAATGGACAAATCTGTGGAATTGGTTTAACATATCAAATTGGTTATGGTATATAAAA
ATATTTATAATGATAGTAGGAGGTTTAATAGGTTTAAGAATAGTTTTTACTGTGCTTACTATAATAAGGAGAG
TTAGGCAGGGATACTCACCTTTGTCGTTCCAGACCCTTCCCCTACACCAGAGGGAACCCGACAGGCCCGAAGG

AATCGAAGAAGGAGGTGGCGAGCAAAACAGAGGCAGATCAGTAAGATTAGTGAGCGGATTCTTAGCTCTTGCC
TGGGACGACCTACGGAGCCTGTGCCTCTTCAGCTACCACCAATTGAGAAACTTTGCCTTGATTGCAGCGAGAA
CTGTGGAACTCCTGGGACACAGCAGTCTCAAGGGACTGAGACTGGGGTGGGAAGCCCTCAAATATCTGTGGAA
TCTTCTGTCATACTGGGGTCAGGAACTAAAGAATAGTGCTATTAATTTGTTTGATACCATAGCAATAGCAGTA
GCTAACTGGACAGACAGAGGTATAGAAATAGGACAAAGAGCTGGCAGAGCTATTGGCAACATACCTAGAAGAA
TCAGACAGGGCCTAGAAAGAGCTTTGCTATAACATGGGTGGCAAATGGTCAAAAAGCAGCATAGTGGGATGGC
CTAAGATTAGGGAAAGGATGAGACAAAGCCCTCCAACAGGAAGCCCTCCAGCAGCAACAGGAACAGGAGCAGT
ATCTCAAGATTTAGCTAGACATGGAGCAATCACAAGCAGTAATACAGCCTCTACTAATCCTGCTTGTGCCTGG
CTGGAAGCACAAGAGGAAGAGGATGAGGTAGGCTTTCCAGTCAGACCACAGGTACCTTTGAGACCAATGACTT
ATAAACTAGCTTTCGATCTCAGCTTCTTTTTAAAAGGAAAGGGGGGACTGGAAGGGTTAGTTTGGTCCCAGAA
AAGACAAGATATCCTTGATCTGTGGATGTATCACACACAAGGAATCTTCCCAGATTGGCAGAACTACACACCA
GGGCCAGGGACTAGATTCCCACTGACCTTTGGGTGGTGCTTCAAACTAGTACCACTAGATCCAGCAGAGGTAG
AGGCAGCTAATGAAGGAGAGAACAACAGCTTATTACACCCCATCAGTCAACATGGGATGGAAGACGGGGACAG
AGAGGTGCTGGTCTGGAGATTTGACAGCAGCCTGGCAAGAGAACACAAAGCCCGAGAGCTGCATCCGGAGTTC
TATAAAGACTGCTGACACAGAAGTTGCTGACTGGGACTTTCCACTGGGGACTTTCCGGGGAGGTGTGGTTGGG
GAGGAGTTTGGGAGTGGCTAACCCTCAGATGCTGCATATAAGCAGCTGCTTCTCGCCTGTACGTGGTCTCTCT
TGCTAGACCAGATTTGAGCCTGGGAGCTCTCTGGCTAGTTAAGGGACCCACTGCTTAAGCCTCAATAAAGCTT
GCCT

>X-13D HIV-1 genome, derived from RNA genomic sequence
TAGCAGTGGCGCCCGAACAGGGACTTGAAGTTAATAGGGACTCGAAAGCGAAAGTTCCAGAGAAGCTCTCTCG
ACGCAGGGACTCGGCTTGCTGAAGTGCACGCAGCAAGAGGCGAGAGCGGCGACTGGTGAGTACGCCAAATTTC
GACTAGCGGAGGCTAGAAGGAGAGAGATGGGTGCGAGAGCGTCGATACTAAGTGGGGGAAAATTAGATGCATG
GGAGAAAATTCGGTTAAGGCCAGGGGGAAAGAAAAAATATCGACTAAAACATTTAGTATGGGCAAGCAGGGAG
TTGGAAAGATTTGCACTTAATCCTAGTCTTTTAGAAACAGCAGAAGGATGTCAACAACTGATGGAACAGTTAC
AATCAACTCTCAGGACAGGATCAGAAGAACTTAAATCATTATTTAATACAATAGCAACCCTTTGGTGCGTACA
TCAAAAGATAGACATAAAAGACACCAAGGAAGCCTTAGATAAAATAGAGGAAGAACAAAATAAGAGCAAGCAA
AAGACACAGCAAAAGACACAGCAGGCAGCAGCTGCCGCAGGAAGCAGCAGCCAAAATTACCCTATAGTGCAAA
ATGCACAAGGGCAAATGATACATCAGGCCATGTCACCTAGAACTTTGAACGCATGGGTGAAGGTAGTAGAGGA
GAAGGCTTTCAGCCCAGAAGTAATACCCATGTTTACAGCATTATCAGAAGGAGCCACCCCACAAGACTTAAAT
ATGATGCTAAACATAGTGGGGGGACACCAGGCAGCAATGCAGATGTTAAAAGATACCATCAATGAGGAAGCTG
CAGAATGGGACAGGACACATCCTGTACATGCAGGGCCTGCTCCACCAGGCCAGATAAGAGAACCAAGGGGAAG
TGACATAGCAGGAACTACTAGTACCCTTCAAGAACAAATAGGATGGATGACAAGCAATCCACCTATCCCAGTG
GGAGAAATCTATAAAAGATGGATAGTCCTGGGATTAAATAAAATAGTAAGAATGTATAGCCCTGTCAGCATTT
TGGATATAAGACAAGGGCCAAAAGAACCCTTTAGAGACTATGTAGATAGGTTCTTTAAAACTTTAAGAGCTGA
ACAAGCTACACAGGATGTAAAAAACTGGATGACAGAAACCTTGCTGGTCCAAAATGCGAATCCAGATTGTAAG
ACCATTCTAAGAGCATTAGGACCAGGGGCTACATTAGAAGAAATGATGACAGCATGTCAGGGAGTGGGAGGAC
CTGGCCATAAAGCAAGGGTTTTGGCTGAGGCTATGAGTCAAGCGCAACAGTCCAACATAATGATGCAGAAGGG
AAACTTTAGGGGCCAGAGAACAATAAAGTGTTTCAATTGTGGCAAAGAAGGACACCTAGCCAGAAACTGCAAG
GCCCCTAGGAAAAGGGGTTGTTGGAAATGTGGTAAGGAAGGACACCAAATGAAAGACTGTACTGAAAGACAGG
CTAATTTTTTAGGGAAAATTTGGCCTTCCAACAAGGGGAGGCCAGGAAATTTTCCTCAGAGCAGACCGGAACC
AACGGCCCCACCACTAGAGAACTTTGGAGTGGGGGAAGAGATACCCCCCCCTCAGAAGCAGGAACCCTCCTCT
CCGAAACAGGAACCGGGGGACAAGGGACTATATCCTCCTTTAACCTCCCTCAAATCACTCTTTGGCAACGACC
AGTAGTCACAGTAAGAATAGAGGGAGAGGTAATAGAAGCCCTATTAGACACAGGGGCAGATGATACAGTAATA
GAAAAGATAAATTTACCAGGAAAATGGAAACCAAAAATGATAGGGGGAATTGGAGGCTTTATCAAAGTAAGAC
AGTATGATCAGATAAGTATAGAAATTTGTGGAAAAAGGGCCATAGGTACAGTATTAGTAGGACCTACACCTGT
CAACATAATTGGACGAAATATGTTGACTCAGATTGGTTGTACTTTAAATTTTCCAATAAGTCCTATTGAAACT
GTACCAGTAAAATTAAAGCCAGGAATGGATGGCCCAAAGGTTAAACAATGGCCATTGACAGAAGAAAAAATAA
AAGCATTAACAGAAATTTGTACAGATATGGAAAAGGAAGGAAAAATTTCAAGAATTGGGCCTGAAAATCCATA
CAATACTCCAGTATTTGCCATAAAGAAAAAAGATAGTACTAAATGGAGAAAATTAGTAGATTTCAGAGAACTC
AATAAGAGAACTCAAGACTTCTGGGAGGTCCAATTAGGAATACCTCATCCCGCAGGGTTAAAAAAGAAAAAAT
CAGTAACAGTATTAGATGTGGGGGATGCATATTTTTCAGTTCCCTTAGATAAGGACTTTAGAAAGTATACTGC
CTTCACTATACCTAGTATAAATAATGAGACACCAGGAATTAGATATCAGTACAATGTGCTCCCACAGGGATGG
AAAGGATCACCAGCAATATTTCAGGCAAGTATGACAAAAATATTAGAGCCCTATAGAATAAATAATCCAGAGA
TGGTGATCTATCAATATATGGATGATTTATATGTAGGATCTGACTTAGAGATAGGGCAGCATAGAGCAAAAAT
AGAGGAGTTGAGAGCACATCTATTGAAGTGGGGATTTACCACACCAGACAAAAAGCATCAGAAAGAACCTCCA
TTTCTTTGGATGGGATATGAACTCCATCCTGACAAATGGACAGTCCAGCCTATACAGCTACCGGATAAAGACC
ACTGGACTGTCAATGATATACAGAAATTAGTGGGAAAACTAAATTGGGCAAGTCAGATTTATGCAGGAATTAA
AGTAAAACAACTGTGTAAACTCCTCAGGGGAGCCAAAGCACTAACAGATATAGTACCAATGACTGAGGAAGCA
GAATTGGAATTGGCAGAGAACAGGGAAATTTTAAAAGAACCTGTACATGGAGTGTATTATGACCCAACAAAAG
ACTTAGTAGCAGAAGTACAGAAACAAGGGCAAGGCCAATGGACATATCAAATTTATCAAGAGCCATTTAAAAA
TCTAAAAACAGGAAAATATGCAAAAAGGAAGTCTGCCCACACTAATGATGTAAAACAGTTAGCAGAGGTAGTG
CAAAAAATAGCTGTAGAAAGCATAGTAATTTGGGGGAAGACCCCTAAATTTAGACTACCCATACAAAGAGAAA
CATGGGAAACATGGTGGACAGAGTATTGGCAGGCTACCTGGATACCTGACTGGGAGTTTGTCAATACCCCTCC
TTTAGTAAAATTATGGTACCAGTTAGAGAAAGACCCCATAGTAGGGGCAGAAACTTTCTATGTAGATGGGGCA
GCCAATAGGGAAACTAAGCTAGGAAAAGCAGGGTATGTCACAGACAGAGGAAGACAAAAGGTTGTCTCCCTAA
CTGAGACAACAAATCAAAAGACTGAATTACAGGCAATATATCTAGCCTTGCAAGATTCAGGATCAGAAGTAAA
TATAGTGACAGACTCACAATATGCATTGGGAATTATTCAGGCACAACCAGACAGAAGTGAATCAGAATTAGTT
AATCAAGTAATAGAGAAGCTAATAGAAAAGGACAAGGTCTACCTGTCATGGGTACCAGCACATAAAGGGATTG
GAGGAAATGAACAAGTAGATAAATTGGTCAGTAATGGAATCAGAAGAGTACTATTTTTAGATGGCATAGATAA
AGCCCAAGAAGATCACGAAAGATATCACAGCAATTGGAGAACAATGGCTAGTGATTTTAATCTGCCACCTATA
GTAGCAAAAGAAATAGTGGCCAGCTGTGATAAATGTCAGCTGAAAGGGGAAGCCATACATGGACAAATAGACT
GTAGTCCAGGAATATGGCAATTAGATTGTACACATTTAGAAGGAAAAATTATCCTGGTAGCAGTCCATGTAGC

CAGTGGCTATATAGAAGCAGAAGTTATCCCAGCAGAAACAGGATCGGAGACAGCATACTTTATATTAAAATTA
GCAGGAAGATGGCCAGTGAAAGTAATACACACAGACAATGGCCCCAATTTTATCAGTGCTGCAGTAAAGGCAG
CATGTTGGTGGGCAAATGTCACACAAGAATTTGGAATTCCCTACAATCCCCAAAGCCAAGGAGTAGTGGAATC
TATGAATAAAGAACTAAAGAAAATTATAGGACAGGTCAGGGATCAAGCTGAACATCTTAAGACAGCAGTACAG
ATGGCAGTATTCATTCACAATTTTAAAAGAAAAGGGGGGATTGGGGGGTACAGTGCAGGGGAAAGAATAATAG
ACATAATAGCATCAGATATACAAACTAAAGAACTACAAAAACAAATTACAAAAATTCAAAATTTTCGGGTTTA
TTACAGGGACAGCAGAGACCCCATTTGGAAAGGACCAGCAAAACTACTCTGGAAAGGTGAAGGGGCAGTAGTA
ATACAGGACAATAGTGATATAAAAGTAGTACCAAGAAGAAAAGCAAAAATCATTAAGGATTATGGAAAACAGA
TGGCAGGTGATGATTGTGTGGCAGGTAGACAGGATGAGGATTAGAACATGGAACAGTTTAGTAAAACATCATA
TGTATGTCTCTAGGAAAGCTAAGGGTTGGTTTTATAGACATCACTATGAAAGTAGGCATCCAAAAGTGAGTTC
AGAAGTACACATCCCATTAGGGGATGCTATGTTAGTAGTAAGAACATATTGGGGTCTGCTTACAGGAGAAAGA
GATTGGCACTTGGGTCATGGGGTCTCCATAGAATGGAGGCAGAAAAGATATAGCACACAAATAGATCCTGAAC
TAGCAGACCAACTAATTCACTCGCATTATTTTGACTGTTTTACAGACTCTGCTATAAGAAAAGCCACATTAGG
ACAAATAGTTAGACCTAAGTGTGAATATCAAGCAGGACATGATAAGGTAGGATCGCTACAATATTTGGCACTA
AAAGCATTAGTAACACCAGAAAGGACAAAGCCACCTTTACCTAGTGTTAAGAAGTTAACAGAAGACAGATGGA
ACAAGTCCCACAAGACCAGGGGCCACAGAGGGAGCCGTTCAATGAATGGACACTAGAACTGTTAGAAGAGCTT
AAGGATGAAGCTGTTAGACATTTTCCTAGGCCGTGGCTCCATGGATTAGGACAACATATCTACAACACATATG
GGGATACTTGGGAAGGGGTTGTAGCTATAATAAGAATTTTGCAACAACTACTGTTTATTCATTTCAGAATTGG
GTGTCAACATAGCAGAATAGGCATTATTCCAGGGAGAAGAGGCAGGAATGGAGCTGGTAGATCCTAGCCTAGA
GCCATGGAACCAACCGGGAAGTCAGCCTACAACTGCTTGTAACAATTGTTACTGTAAAATATGCTGCTGGCAT
TGCCAATTATGCTTTCTGAACAAGGGCTTAGGCATCTCCTATGGCAGGAAGAAGCGGAGACCCCGACGAAGAA
CTCCTCAAAGCCGTCAGGATCATCAAAATCCTGTACCAAAGCAGTGAGTAGTAATAATTAGTATATGTGATGA
AATCTTTAGGAATAGCTGCAATAGTAGGATTAGTAGTAGCATTCATAGCAGCCATAGTTGTGTGGACCATAGT
GTTTATTGAATATAGAAAAATAAGGAAACAGAAGAAAATAGACAAGATACTTGATAGAATAAGAGAAAGAGCA
GAAGACAGTGGCAATGAGAGTGATGGGGACACAGAAGAATTATACACTCTTATGGAGGTGGGGTATGATAATA
TTTTGGATAATGATAATTTGTAATGCTAAAGACTTGTGGGTCACAGTCTATTATGGGGTACCAGTGTGGAGAG
ACGCAGAGACCACCCTATTTTGTGCATCAGATGCCAAAGCATATGATTCAGAAGTACATAATGTCTGGGCTAC
ACATGCCTGTGTACCCACAGACCCTAACCCACAAGAAATACATTTGGAAAATGTAACAGAAAATTTTAACATG
TGGAAAAATACCATGGTGGAGCAGATGCATGAAGATATAATCAGTCTCTGGGACCAAAGCCTAAAGCCATGTG
TAAAGTTAACCCCGCTCTGCGTTACTTTAAATTGTAGTGACTATACATATAACTCCACCAGCTATGTTAAATC
CACCAACAACTCTGAAATGCAAGAAATAAAAAACTGCTCTTTCAATATAACCACAGAATTAAGAGATAAGAAA
CAGAAAATGTATGCACTTTTTTATAAACTTGATGTAAAACAACTTGATAATAATAATCAGACGTATAGTTTAA
TAAATTGTAACACCTCAACCATTACACAGGCTTGTCCAAAGGTATCCTTTGAGCCAATTCCCATACATTATTG
TGCCCCAGCTGGTTTTGCAATTCTAAAGTGTAAGGATAAGAGGTTCAATGGAACAGGGCCATGCAAGAATGTC
AGCACAGTACAATGCACACATGGAATCAAGCCAGTAGTATCAACTCAACTGCTGTTAAATGGCAGTCTAGCAG
AAGAAGAGGTAGTGATTAGATCTGAGAATATCACAAACAATGCCAAGACCATAATAGTACAGTTGAATAAGCC
TGTAAGAATTAATTGTACCAGAACTGGTAACAATACAAGAAAAAGTGTACGTATAGGACCAGGGCAAACATAC
TATGCAACAGGTGAAATAATAGGAGATATAAGAAAAGCATATTGTAATGTCAGTAAAACAGAATGGGATGAGG
CTTTACAACAGGTAGTCACACAATTAAAGAAGCATTTCAATACCACAATAATCTTTAATAGTGCCTCAGGAGG
AGATCTAGAAATTACAACACATAGTTTTAATTGTAGAGGAGAATTTTTTTATTGCAATACAACAAACCTGTAT
AATAGCACTTGGAATAATAGCACTTGGAATGGCAATACCAGTACAAATCTCACAGAGTCAAATGATACTATAA
CTCTCCAATGCAGACTAAAGCAATTTATAAAAATGTGGCAGAGAGTAGGACAAGCAATGTATGCCCCTCCTAT
CCAAGGAGAAATAAGGTGTGATTCAAACATTACTGGACTATTATTAACAAGAGATGGAGGGAATAATAGGACA
AATGAGACCTTTAGGCCTGGAGGAGGAGATATGAGGGACAATTGGAGAAGTGAATTATATAAGTATAAAGTAG
TAAAAATTGAACCAATAGGTGTGGCACCAACCAGGGCAAAAAGAAGAATGGTGGAGAGAGAAAAAAGAGCAGT
TGGACTGGGAGCTGTTTTCCTTGGGTTCTTAGGAGCAGCAGGAAGCACTATGGGCGCGGCGTCAATAACGCTG
ACGGTACAGGCCAGGCAATTATTGTCTGGTATAGTGCAACAGCAGAGCAATTTGCTGAAGGCTATAGAGGCTC
AACAGCAAATGTTGAGACTCACGGTCTGGGGCATTAAGCAGCTCCAGGCAAGAGTCCTGGCTCTGGAAAGATA
CCTAAAGGATCAACAGCTCCTAGGAATTTGGGGCTGCTCTGGAAAACTCATCTGCACCACTGGTGTACGCTGG
AACTCAAGCTGGAGTAATAAAACTTATGAGGACATATGGGATAACATGACCTGGGTGCAATGGGAGAGGGAAA
TTAGCAATTACACAAACACAATATATACTCTACTTGAAGAATCGCAGAACCAGCAGGAAAAAAATGAACAAGA
CTTATTGGCATTGGACAAATGGACAAATCTGTGGAATTGGTTTAACATATCAAATTGGTTATGGTATATAAAA
ATATTTATAATGATAGTAGGAGGTTTAATAGGTTTAAGAATAGTTTTTACTGTGCTTACTATAATAAGGAGAG
TTAGGCAGGGATACTCACCTTTGTCGTTCCAGACCCTTCCCCTACACCAGAGGGAACCCGACAGGCCCGAAGG

AATCGAAGAAGGAGGTGGCGAGCAAGACAGAGGCAGATCAGTAAGATTAGTGAGCGGATTCTTAGCTCTTGCC
TGGGACGACCTACGGAGCCTGTGCCTCTTCAGCTACCACCAATTGAGAAACTTTGCCTTGATTGCAGCGAGAA
CTGTGGAACTCCTGGGACACAGCAGTCTCAAGGGACTGAGACTGGGGTGGGAAGCCCTCAAATATCTGTGGAA
TCTTCTGTCATACTGGGGTCAGGAACTAAAGAATAGTGCTATTAATTTGTTTGATACCATAGCAATAGCAGTA
GCTAACTGGACAGACAGAGGTATAGAAATAGGACAAAGAGCTGGCAGAGCTATTGGCAACATACCTAGAAGAA
TCAGACAGGGCCTAGAAAGAGCTTTGCTATAACATGGGTGGCAAATGGTCAAAAAGCAGCATAGTGGGATGGC
CTAAGATTAGGGAAAGGATGAGACAAAGCCCTCCAACAGGAAGCCCTCCAGCAGCGACAGGAACAGGAGCAGT
ATCCCCAGCAGCAACAGGAACAGGAGCAGTATCTCAAGATTTAGCTAGACATGGAGCAATCACAAGCAGTAAT
ACAGCCTCTACTAATCCTGCTTGTGCCTGGCTGGAAGCACAAGAGGAAGAGGATGAGGTAGGCTTTCCAGTCA
GACCACAGGTACCTTTGAGACCAATGACTTATAAACTAGCTTTCGATCTCAGCTTCTTTTTAAAAGGAAAGGG
GGGACTGGAAGGGTTAGTTTGGTCCCAGAAAAGACAAGATATCCTTGATCTGTGGATGTATCACACACAAGGA
ATCTTCCCAGATTGGCAGAACTACACACCAGGGCCAGGGACTAGATTCCCACTGACCTTTGGGTGGTGCTTCA
AACTAGTACCACTAGATCCAGCAGAGGTAGAGGCAGCTAATGAAGGAGAGAACAACAGCTTATTACACCCCAT
CAGTCAACATGGGATGGAAGACGGGGACAGAGAGGTGCTGGTCTGGAGATTTGACAGCAGCCTGGCAAGAGAA
CACAAAGCCCGAGAGCTGCATCCGGAGTTCTATAAAGACTGCTGACACAGAAGTTGCTGACTGGGACTTTCCA
CTGGGGACTTTCCGGGGAGGTGTGGTTGGGGAGGAGTTTGGGAGTGGCTAACCCTCAGATGCTGCATATAAGC
AGCTGCTTCTCGCCTGTACGTGGTCTCTCTTGCTAGACCAGATTTGAGCCTGGGAGCTCTCTGGCTAGTTAAG
GGACCCACTGCTTAAGCCTCAATAAAGCTTGCCT

>X-14B HIV-1 genome, derived from RNA genomic sequence
TAGCAGTGGCGCCCGAACAGGGACTTGAAGTTAATAGGGACTCGAAAGCGAAAGTTCCAGAGAAGCTCTCTCG
ACGCAGGGACTCGGCTTGCTGAAGTGCACGCAGCAAGAGGCGAGAGCGGCGACTGGTGAGTACGCCAAATTTC
GACTAGCGGAGGCTAGAAGGAGAGAGATGGGTGCGAGAGCGTCGATACTAAGTGGGGGAAAATTAGATGCATG
GGAGAAAATTCGGTTAAGGCCAGGGGGAAAGAAAAAATATCGACTAAAACATTTAGTATGGGCAAGCAGGGAG
TTGGAAAGATTTGCACTTAATCCTAGTCTTTTAGAAACAGCAGAAGGATGTCAACAACTGATAGAACAGTTAC
AATCAACTCTCAGGACAGGATCAGAAGAACTTAAATCATTATTTAATACAATAGCAACCCTTTGGTGCGTACA
TCAAAAGATAGACATAAAAGACACCAAGGAAGCCTTAGATAAAATAGAGGAAGAACAAAATAGGAGCAAGCAA
AAGACACAGCAAAAAACACAGCAGGCAGCAGCTGCCGCAGGAAGCAGCAGCCAAAATTACCCTATAGTGCAAA
ATGCACAAGGGCAAATGATACATCAGGCCATGTCACCTAGAACTTTGAACGCATGGGTGAAGGTAGTAGAGGA
GAAGGCTTTCAGCCCAGAAGTAATACCCATGTTTACAGCATTATCAGAAGGAGCCACCCCACAAGACTTAAAT
ATGATGCTAAACATAGTGGGGGGACACCAGGCAGCAATGCAGATGTTAAAAGATACCATCAATGAGGAAGCTG
CAGAATGGGACAGGACACATCCTGTACATGCAGGGCCTGCTCCACCAGGCCAGATAAGAGAACCAAGGGGAAG
TGACATAGCAGGAACTACTAGTACCCTTCAAGAACAAATAGGATGGATGACAAGCAATCCACCTATCCCAGTG
GGAGAAATCTATAAAAGATGGATAGTCCTGGGATTAAATAAAATAGTAAGAATGTATAGCCCTGTCAGCATTT
TGGATATAAGACAAGGGCCAAAAGAACCCTTTAGAGACTATGTAGATAGGTTCTTTAAAACTTTAAGAGCTGA
ACAAGCTACACAGGAGGTAAAAAACTGGATGACAGAAACCTTGCTGGTCCAAAATGCGAATCCAGATTGTAAG
ACCATTCTAAGAGGATTAGGACCAGGGGCTACATTAGAAGAAATGATGACAGCATGTCAGGGAGTGGGAGGAC
CTGGCCATAAAGCAAGGGTTTTGGCTGAGGCTATGAGTCAAGCGCAACAGTCCAACATAATGATGCAGAAGGG
AAACTTTAGGGGCCAGAGAACAATAAAGTGTTTCAATTGTGGCAAAGAAGGACACCTAGCCAGAAACTGCAAG
GCCCCTAGGAAAAGGGGTTGTTGGAAATGTGGTAAGGAAGGACACCAAATGAAAGACTGTACTGAAAGACAGG
CTAATTTTTTAGGGAAAATTTGGCCTTCCAACAAGGGGAGGCCAGGAAATTTTCCTCAGAGCAGACCGGAACC
AACGGCCCCACCACTAGAGAACTTTGGAGTGGGGGAAGAGATACCCCCCCTTCAGAAGCAGGAACCCTCCTCT
CCGAAACAGGAACCGAGGGACAAGGGACTATATCCTCCTTTAACCTCCCTCAAATCACTCTTTGGCAACGACC
AGTAGTCACAGTAAGAATAGAGGGAGAGGTAATAGAAGCCCTATTAGACACAGGGGCAGATGATACAGTAATA
GAAAAGATAAATTTACCAGGAAAATGGAAACCAAAAATGATAGGGGGAATTGGAGGCTTTATCAAAGTAAGAC
AGTATGATCAGATAAGTATAGAAATTTGTGGAAAAAGGGCCATAGGTACAGTGTTAGTAGGACCTACACCTGT
CAACATAATTGGACGAAATATGTTGACTCAGATTGGTTGTACTTTAAATTTTCCAATAAGTCCTATTGAAACT
GTACCAGTAAAATTAAAGCCAGGAATGGATGGGCCAAAGGTTAAACAATGGCCATTGACAGAAGAAAAAATAA
AAGCATTAACAGAAATTTGTACAGATATGGAAAAGGAAGGAAAAATTTCAAGAATTGGGCCTGAAAATCCATA
CAATACTCCAGTATTTGCCATAAAGAAAAAAGATAGTACTAAATGGAGAAAATTAGTAGATTTCAGAGAACTC
AATAAGAGAACTCAAGACTTCTGGGAGGTCCAATTAGGAATACCTCATCCCGCAGGGTTAAAAAAGAAAAAAT
CAGTAACAGTATTAGATGTGGGGGATGCATATTTTTCAGTTCCCTTAGATAAGGACTTTAGAAAGTATACTGC
CTTCACTATACCTAGTATAAATAATGAGACACCAGGAATTAGATATCAGTACAATGTGCTCCCACAGGGATGG
AAAGGATCACCAGCAATATTTCAGGCAAGTATGATAAAAATATTAGAGCCCTATAGAATAAATAATCCAGAAA
TGGTGATCTATCAATATATGGATGATTTATATGTAGGATCTGACTTAGAGATAGGGCAGCATAGAGCAAAAAT
AGAGGAGTTGAGAGCACATCTATTGAAGTGGGGACTTACCACACCAGACAAAAAGCATCAGAAAGAACCTCCA
TTTCTTTGGATGGGATATGAACTCCATCCTGACAAATGGACAGTCCAGCCTATACAGCTACCGGATAAAGACC
ACTGGACTGTCAATGATATACAGAAATTAGTGGGAAAACTAAATTGGGCAAGTCAGATTTATGCAGGAATTAA
AGTAAAACAACTGTGTAAACTCCTCAGGGGAGCCAAAGCACTAACAGATATAGTACCAATGACTGAGGAAGCA
GAATTGGAATTGGCAGAGAACAGGGAAATTTTAAAAGAAACTGTACATGGAGTGTATTATGACCCAACAAAAG
ACTTAGTAGCAGAAGTACAGAAACAAGGGCAAGGCCAATGGACATATCAAATTTATCAAGAGCCATTTAAAAA
TCTAAAAACGGGAAAATATGCAAAAAGGAAGTCTGCCCACACTAATGATGTAAAACAGTTAGCAGAGGTAGTG
CAAAAAATAGCTGTAGAAAGCATAGTAATTTGGGGGAAGACCCCTAAATTTAGACTACCCATACAAAGAGAAA
CATGGGAAACATGGTGGACAGAGTATTGGCAGGCTACCTGGATACCTGACTGGGAGTTTGTCAATACCCCTCC
TTTAGTAAAATTATGGTACCAGTTAGAGAAAGACCCCATAGTAGGGGCAGAAACTTTCTATGTAGATGGGGCA
GCCAATAGGGAAACTAAGCTAGGAAAAGCAGGGTATGTCACAGACAGAGGAAGACAAAAGGTTGTCTCCCTAA
CTGAGACAACAAATCAAAAGACTGAATTACAGGCAATATATCTAGCCTTGCAAGATTCAGGATCAGAAGTAAA
TATAGTGACAGACTCACAATATGCATTGGGAATTATTCAGGCACAACCAGACAGAAGTGAATCAGAATTAGTT
AATCAAGTAATAGAGAAGCTAATAGAAAAGGACAAGGTTTACCTGTCATGGGTACCAGCACATAAAGGGATTG
GAGGAAATGAACAAGTAGATAAATTGGTCAGTAATGGAATCAGAAGAGTACTATTTTTAGATGGCATAGATAA
AGCCCAAGAAGATCACGAAAAATATCACAGCAATTGGAGAACAATGGCTAGTGATTTTAATCTGCCACCTATA
GTAGCAAAAGAAATAGTGGCCAGCTGTGATAAATGTCAGCTGAAAGGGGAAGCCATACATGGACAAGTAGACT
GTAGTCCAGGAATATGGCAATTAGATTGTACACATTTAGAAGGAAAAATTATCCTGGTAGCAGTCCATGTAGC

CAGTGGCTATATAGAAGCAGAAGTTATCCCAGCAGAAACAGGATCGGAGACAGCATATTTTATATTAAAATTA
GCAGGAAGATGGCCAGTGAAAGTAATACACACAGACAATGGCCCCAATTTTATCAGTGCTGCAGTAAAGGCAG
CATGTTGGTGGGCAAATGTCACACAAGAATTTGGAATTCCCTACAATCCCCAAAGCCAAGGAGTAGTGGAATC
TATGAATAAAGAACTAAAGAAAATTATAGGACAGGTCAGGGATCAAGCTGAACATCTTAAGACAGCAGTACAG
ATGGCAGTATTCATTCACAATTTTAAAAGAAAAGGGGGGATTGGGGGGTACAGTGCAGGGGAAAGAATAATAG
ACATAATAGCATCAGATATACAAACTAAAGAACTACAAAAACAAATTACAAAAATTCAAAATTTTCGGGTTTA
TTACAGGGACAGCAGAGACCCCATTTGGAAAGGACCAGCAAAACTACTCTGGAAAGGTGAAGGGGCAGTAGTA
ATACAGGACAATAGTGATATAAAAGTAGTACCAAGAAGAAAAGCAAAAATCATTAAGGATTATGGAAAACAGA
TGGCAGGTGATGATTGTGTGGCAGGTAGACAGGATGAGGATTAGAACATGGAACAGTTTAGTAAAACATCATA
TGTATGTCTCTAAGAAAGCTAAGGGTTGGTTTTATAGACATCACTATGAAAGTAGGCATCCAAAAGTGAGTTC
AGAAGTACACATCCCATTAGGGGATGCTATGTTAGTAGTAAGAACATATTGGGGTCTGCTTACAGGAGAAAGA
GATTGGCACTTGGGTCATGGGGTCTCCATAGAATGGAGGCAGAAAAGATATAGCACACAAATAGATCCTGAAC
TAGCAGACCAACTAATTCACTCGCATTATTTTGACTGTTTTACAGACTCTGCTATAAGAAAAGCCACATTAGG
ACAAATAGTTAGACCTAAGTGTGAATATCAAGCAGGACATAATAAGGTAGGATCGCTACAATATTTGGCACTA
AAAGCATTAGTAACACCAGAAAGGACACAGCCACCTTTACCTAGTGTTAAGAAGTTAACAGAAGACAGATGGA
ACAAGTCCCACAAGACCAGGGGCCACAGAGGGAGCCGCTCAATGAATGGACACTAGAACTGTTAGAAGAGCTT
AAGAATGAAGCTGTTAGACATTTTCCTAGGCCGTGGCTCCATGGATTAGGACAACATATCTACAACACATATG
GGGATACTTGGGAAGGGGTTGTAGCTATAATAAGAATTTTGCAACAACTACTGTTTATTCATTTCAGAATTGG
GTGTCAACATAGCAGAATAGGCATTATTCCAGGGAGAAGAGGCAGGAATGGAGCTGGTAGATCCTAGCCTAGA
GCCATGGAACCATCCGGGAAGTCAGCCTACAACTGCTTGTAACAATTGTTACTGTAAAATATGCTGCTGGCAT
TGCCAATTATGCTTTTTGAACAAGGGCTTAGGCATCTCCTATGGCAGGAAGAAGCGGAGACCCCGACGAAGAA
CTCCTCAAAGCCGTCAAGATCATCAAAATCCTGTACCAAAGCAGTAAGTAGTAATAATTAGTATATGTAATGA
AATCTTTAGGAATAGCTGCAATAGTAGGATTAGTAGTAGCATTCATAGCAGCCATAGTTGTGTGGACCATAGT
GTTTATTGAATATAGAAAAATAAGGAAACAGAAGAAAATAGACAAGATACTTGATAGAATAAGAGAAAGAGCA
GAAGACAGTGGCAATGAGAGTGATGGGGACACAGAAGAATTATACACTCTTATGGAGGTGGGGTATGATAATA
TTTTGGATAATGATAATTTGTAATGCTAAAGACTTGTGGGTCACAGTCTATTATGGGGTACCAGTGTGGAGAG
ACGCAGAGACCACCTTATTTTGTGCATCAGATGCCAAAGCATATGATTCAGAAGTACATAATGTCTGGGCTAC
ACATGCCTGTGTACCCACAGACCCTAACCCACAAGAAATACATTTGGAAAATGTAACAGAAAATTTTAACATG
TGGAAAAATACCATGGTGGAGCAGATGCATGAAGATATAATCAGTCTCTGGGACCAAAGCCTAAAGCCATGTG
TACAGTTAACCCCGCTCTGCGTTACTTTAAATTGTAGTGACTATAACTCCACCAGCTATGTTAACTACACCAA
CAAGTCTGACATGCAAGAAATAAAAAACTGCTCTTTCAATATAACCACAGAATTAAGAGATAAGAAACAGAAA
ATGTATGCACTTTTTTATAAACTTGATGTAAAACAACTTGATAATAATAATCAGACGTATAGTTTAATAAATT
GTAACACCTCAACCATTACACAGGCTTGTCCAAAGGTATCCTTTGAGCCAATTCCCATACATTATTGTGCCCC
AGCTGGTTTTGCAATTCTAAAGTGTAAGGATAAGAGTTTCAATGGAACAGGGCCATGCAAGAATGTCAGCACA
GTACAATGCACACATGGAATCAAGCCAGTAGTATCAACTCAACTGCTGTTAAATGGCAGTCTAGCAGAAGAAG
AGGTAGTGATTAGATCTGAGAATATCACAAACAATGCCAAGACCATAATAGTACAGTTGAATAAGCCTGTGAG
AATTAATTGTACCAGAACTAGTAACAATACAAGAAAAAGTGTACGTATAGGACCAGGGCAAACATACTATGCA
ACAGGTGAAATAATAGGAGATATAAGAAAAGCACATTGTAATGTCAGTAAAACAGAATGGGATAAGGCTTTAC
AACAGGTAGCCACACAATTAAAGAAGCATTTCAATACCACAATAATCTTTAATAATGCCTCAGGAGGAGATCT
AGAAATTACAACACATAGTTTTAATTGTAGAGGAGAATTTTTTTATTGCAATACAACAAACCTGTATAATAGC
ACTTGGAATGACACTACCAGTACAAATCTCACAGAGTCAAATGATACTATAACTCTCCAATGCAGACTAAAGC
AATTTATAAAAATGTGGCAGAGAGTAGGACAAGCAATGTATGCCCCTCCTATCCAAGGAGAAATAAGGTGTGA
TTCAAACATTACTGGACTATTATTAACAAGAGATGGAGGGAATAATAGGACAAATGAGACCTTTAGGCCTGGA
GGAGGAGATATGAGGGACAATTGGAGAAGTGAATTATATAAGTATAAAGTAGTAAAAATTGAACCAATAGGTG
TGGCACCAACCAGGGCAAAAAGAAGAATGGTGGAGAGAGAAAAAAGAGCAGTTGGACTGGGAGCTGTTTTCCT
TGGGTTCTTAGGAGCAGCAGGAAGCACTATGGGCGCGGCGTCAATAACGCTGACGGTACAGGCCAGGCAATTA
TTGTCTGGTATAGTGCAACAGCAGAGCAATTTGCTGAAGGCTATAGAGGCTCAACAGCAAATGTTGAGACTCA
CGGTCTGGGGCATTAAGCAGCTCCAGGCAAGAGTCCTGGCTCTGGAAAGATACCTAAAGGATCAACAGCTCCT
AGGAATTTGGGGCTGCTCTGGAAAACTCATCTGCACCACTGGTGTACGCTGGAACTCAAGCTGGAGTAATAAA
ACTTATGAGGACATATGGGATAACATGACCTGGGTGCAATGGGAGAGGGAAATTAGCAATTACACAAACACAA
TATATACTCTACTTGAAGAATCGCAGAACCAGCAGGAAAAAAATGAACAAGACTTATTGGCATTGAACAAATG
GACAAATCTGTGGAATTGGTTTAACATATCAAATTGGTTATGGTATATAAAAATATTTATAATGATAGTAGGA
GGTTTAATAGGTTTAAGAATAGTTTTTACTGTGCTTACTATAATAAGGAGAGTTAGGCAGGGATACTCACCTT
TGTCGTTCCAGACCCTTCCCCTACACCAGAGGGAACCCGACAGGCCCGAAGGAATCGAAGAAGGAGGTGGCGA

GCAAGACAGAGGCAGATCAGTAAGATTAGTGAGCGGATTCTTAGCTCTTGCCTGGGACGACCTACGGAGCCTG
TGCCTTTTCAGCTACCACCAATTGAGAAACTTTGCCTTGATTGCAGCGAGAACTGTGGAACTCCTGGGACACA
GCAGTCTCAAGGGACTGAGACTGGGGTGGGAAGCCCTCAAATATCTGTGGAATCTTCTGTCATACTGGGGTCA
GGAACTAAAGAATAGTGCTATTAATTTGTTTGATACCATAGCAATAGCAGTAGCTAACTGGACAGACAGAGGT
ATAGAAATAGGACAAAGAGTTGGCAGAGCTATTGGCAACATACCTAGAAGAATCAGACAGGGCCTAGAAAGAG
CTTTGCTATAACATGGGTGGCAAATGGTCAAAAAGCAGCATAGTGGGATGGCCTAAGATTAGGGAAAGGATGA
GACAAAGCCCTCCAGGAGGCCCTCCAGCAGCGACAGGATCCCCAGCAGCAACAGGAACAGGAGCAGTATCTCA
AGATTTAGCTAGACATGGAGCAATCACAAGCAGTAATACAGCCTCTACTAATTCTGCTTGTGCCTGGCTGGAA
GCACAAGAGGAAGAGGATGAGGTAGGCTTTCCAGTCAAACCACAGGTACCTTTGAGACCAATGACTTATAAAC
TAGCTTTCGATCTCAGCTTCTTTTTAAAAGATAAGGGGGGACTGGAAGGGTTAGTTTGGTCCCAGAAAAGACA
AGATATCCTTGATCTGTGGATGTATCACACACAAGGAATCTTCCCAGATTGGCAGAACTACACACCAGGGCCA
GGGACTAGATTCCCACTGACCTTTGGGTGGTGCTTCAAACTAGTACCACTAGAGCCAGCAGAGGTAGAGGCAG
CTAATGAAGGAGAAAACAACAGCTTATTACACCCCATCAGTCAACATGGGATGGAAGACGAGGACAGAGAGGT
GCTGGTCTGGAGATTTGACAGCAGCCTGGCAAGAGAACACAAAGCCCGAGAGCTGCATCCGGAGTTCTATAAA
GACTGCTGACACAGAAGTTGCTGACTGGGACTTTCCACTGGGGACTTTCCGGGGAGGTGTGGTTGGGGAGGAG
TTTGGGAGTGGCTAACCCTCAGATGCTGCATATAAGCAGCTGCTTCTCGCCTGTACGTGGTCTCTCTTGCTAG
ACCAGATTTGAGCCTGGGAGCTCTCTGGCTAGTTAAGGGACCCACTGCTTAAGCCTCAATAAAGCTTGCCT

>X-14C HIV-1 genome, derived from RNA genomic sequence
TAGCAGTGGCGCCCGAACAGGGACTTGAAGTTAATAGGGACTCGAAAGCGAAAGTTCCAGAGAAGCTCTCTCG
ACGCAGGGACTCGGCTTGCTGAAGTGCACGCAGCAAGAGGCGAGAGCGGCGACTGGTGAGTACGCCAAATTTC
GACTAGCGGAGGCTAGAAGGAGAGAGATGGGTGCGAGAGCGTCGATACTAAGTGGGGGAAAATTAGATGCATG
GGAGAAAATTCGGTTAAGGCCAGGGGGAAAGAAAAAATATCGACTAAAACATTTAGTATGGGCAAGCAGGGAG
TTGGAAAGATTTGCACTTAATCCTAGTCTTTTAGAAACAGCAGAAGGATGTCAACAACTGATGGAACAGTTAC
AATCAACTCTCAGGACAGGATCAGAAGAACTTAAATCATTATTTAATACAATAGCAACCCTTTGGTGCGTACA
TCAAAAGATAGACATAAAAGACACCAAGGAAGCCTTAGATAAAATAGAGGAAGAACAAAATAAGAGCAAGCAA
AAGACACAGCAAAAGACACAGCAGGCAGCAGCTGCCGCAGGAAGCAGCAGCCAAAATTACCCTATAGTGCAAA
ATGCACAAGGGCAAATGATACATCAGGCCATGTCACCTAGAACTTTGAACGCATGGGTGAAGGTAGTAGAGGA
GAAGGCTTTCAGCCCAGAAGTAATACCCATGTTTACAGCATTATCAGAAGGAGCCACCCCACAAGACTTAAAT
ATGATGCTAAACATAGTGGGGGGACACCAGGCAGCAATGCAGATGTTAAAAGATACCATCAATGAGGAAGCTG
CAGAATGGGACAGGACACATCCTGTACATGCAGGGCCTGCTCCACCAGGCCAGATAAGAGAACCAAGGGGAAG
TGACATAGCAGGAACTACTAGTACCCTTCAAGAACAAATAGGATGGATGACAAGCAATCCACCTATCCCAGTG
GGAGAAATCTATAAAAGATGGATAGTCCTGGGATTAAATAAAATAGTAAGAATGTATAGCCCTGTCAGCATTT
TGGATATAAGACAAGGGCCAAAAGAACCCTTTAGAGACTATGTAGATAGGTTCTTTAAAACTTTAAGAGCTGA
ACAAGCTACACAGGATGTAAAAAACTGGATGACAGAAACCTTGCTGGTCCAAAATGCGAATCCAGATTGTAAG
ACCATTCTAAGAGCATTAGGACCAGGGGCTACATTAGAAGAAATGATGACAGCATGTCAGGGAGTGGGAGGAC
CTGGCCATAAAGCAAGGGTTTTGGCTGAGGCTATGAGTCAAGCGCAACAGTCCAACATAATGATGCAGAAGGG
AAACTTTAGGGGCCAGAGAACAATAAAGTGTTTCAATTGTGGCAAAGAAGGACACCTAGCCAGAAACTGCAAG
GCCCCTAGGAAAAGGGGTTGTTGGAAATGTGGTAAGGAAGGACACCAAATGAAAGACTGTACTGAAAGACAGG
CTAATTTTTTAGGGAAAATTTGGCCTTCCAACAAGGGGAGGCCAGGAAATTTTCCTCAGAGCAGACCGGAACC
AACGGCCCCACCACTAGAGAACTTTGGAGTGGGGGAAGAGATACCCCCCCCTCAGAAGCAGGAACCCTCCTCT
CCGAAACAGGAACCGGGGGACAAGGGACTATATCCTCCTTTAACCTCCCTCAAATCACTCTTTGGCAACGACC
AGTAGTCACAGTAAGAATAGAGGGAGAGGTAATAGAAGCCCTATTAGACACAGGGGCAGATGATACAGTAATA
GAAAAGATAAATTTACCAGGAAAATGGAAACCAAAAATGATAGGGGGAATTGGAGGCTTTATCAAAGTAAGAC
AGTATGATCAGATAAGTATAGAAATTTGTGGAAAAAGGGCCATAGGTACAGTATTAGTAGGACCTACACCTGT
CAACATAATTGGACGAAATATGTTGACTCAGATTGGTTGTACTTTAAATTTTCCAATAAGTCCTATTGAAACT
GTACCAGTAAAATTAAAGCCAGGAATGGATGGGCCAAAGGTTAAACAATGGCCATTGACAGAAGAAAAAATAA
AAGCATTAACAGAAATTTGTACAGATATGGAAAAGGAAGGAAAAATTTCAAGAATTGGGCCTGAAAATCCATA
CAATACTCCAGTATTTGCCATAAAGAAAAAAGATAGTACTAAATGGAGAAAATTAGTAGATTTCAGAGAACTC
AATAAGAGAACTCAAGACTTCTGGGAGGTCCAATTAGGAATACCTCATCCCGCAGGGTTAAAAAAGAAAAAAT
CAGTAACAGTATTAGATGTGGGGGATGCATATTTTTCAGTTCCCTTAGATAAGGACTTTAGAAAGTATACTGC
CTTCACTATACCTAGTATAAATAATGAGACACCAGGAATTAGATATCAGTACAATGTGCTCCCACAGGGATGG
AAAGGATCACCAGCAATATTTCAGGCAAGTATGACAAAAATATTAGAGCCCTATAGAATAAATAATCCAGAGA
TGGTGATCTATCAATATATGGATGATTTATATGTAGGATCTGACTTAGAGATAGGGCAGCATAGAGCAAAAAT
AGAGGAGTTGAGAGCACATCTATTGAAGTGGGGATTTACCACACCAGACAAAAAGCATCAGAAAGAACCTCCA
TTTCTTTGGATGGGATATGAACTCCATCCTGACAAATGGACAGTCCAGCCTATACAGCTACCGGATAAAGACC
ACTGGACTGTCAATGATATACAGAAATTAGTGGGAAAACTAAATTGGGCAAGTCAGATTTATGCAGGAATTAA
AGTAAAACAACTGTGTAAACTCCTCAGGGGAGCCAAAGCACTAACAGATATAGTACCAATGACTGAGGAAGCA
GAATTGGAATTGGCAGAGAACAGGGAAATTTTAAAAGAACCTGTACATGGAGTGTATTATGACCCAACAAAAG
ACTTAGTAGCAGAAGTACAGAAACAAGGGCAAGGCCAATGGACATATCAAATTTATCAAGAGCCATTTAAAAA
TCTAAAAACAGGAAAATATGCAAAAAGGAAGTCTGCCCACACTAATGATGTAAAACAGTTAGCAGAGGTAGTG
CAAAAAATAGCTGTAGAAAGCATAGTAATTTGGGGGAAGACCCCTAAATTTAGACTACCCATACAAAGAGAAA
CATGGGAAACATGGTGGACAGAGTATTGGCAGGCTACCTGGATACCTGACTGGGAGTTTGTCAATACCCCTCC
TTTAGTAAAATTATGGTACCAGTTAGAGAAAGACCCCATAGTAGGGGCAGAAACTTTCTATGTAGATGGGGCA
GCCAATAGGGAAACTAAGCTAGGAAAAGCAGGGTATGTCACAGACAGAGGAAGACAAAAGGTTGTCTCCCTAA
CTGAGACAACAAATCAAAAGACTGAATTACAGGCAATATATCTAGCCTTGCAAGATTCAGGATCAGAAGTAAA
TATAGTGACAGACTCACAATATGCATTGGGAATTATTCAGGCACAACCAGACAGAAGTGAATCAGAATTAGTT
AATCAAGTAATAGAGAAGCTAATAGAAAAGGACAAGGTCTACCTGTCATGGGTACCAGCACATAAAGGGATTG
GAGGAAATGAACAAGTAGATAAATTGGTCAGTAATGGAATCAGAAGAGTACTATTTTTAGATGGCATAGATAA
AGCCCAAGAAGATCACGAAAGATATCACAGCAATTGGAGAACAATGGCTAGTGATTTTAATCTGCCACCTATA
GTAGCAAAAGAAATAGTGGCCAGCTGTGATAAATGTCAGCTGAAAGGGGAAGCCATACATGGACAAATAGACT
GTAGTCCAGGAATATGGCAATTAGATTGTACACATTTAGAAGGAAAAATTATCCTGGTAGCAGTCCATGTAGC

CAGTGGCTATATAGAAGCAGAAGTTATCCCAGCAGAAACAGGATCGGAGACAGCATACTTTATATTAAAATTA
GCAGGAAGATGGCCAGTGAAAGTAATACACACAGACAATGGCCCCAATTTTATCAGTGCTGCAGTAAAGGCAG
CATGTTGGTGGGCAAATGTCACACAAGAATTTGGAATTCCCTACAATCCCCAAAGCCAAGGAGTAGTGGAATC
TATGAATAAAGAACTAAAGAAAATTATAGGACAGGTCAGGGATCAAGCTGAACATCTTAAGACAGCAGTACAG
ATGGCAGTATTCATTCACAATTTTAAAAGAAAAGGGGGGATTGGGGGGTACAGTGCAGGGGAAAGAATAATAG
ACATAATAGCATCAGATATACAAACTAAAGAACTACAAAAACAAATTACAAAAATTCAAAATTTTCGGGTTTA
TTACAGGGACAGCAGAGACCCCATTTGGAAAGGACCAGCAAAACTACTCTGGAAAGGTGAAGGGGCAGTAGTA
ATACAGGACAATAGTGATATAAAAGTAGTACCAAGAAGAAAAGCAAAAATCATTAAGGATTATGGAAAACAGA
TGGCAGGTGATGATTGTGTGGCAGGTAGACAGGATGAGGATTAGAACATGGAACAGTTTAGTAAAACATCATA
TGTATGTCTCTAGGAAAGCTAAGGGTTGGTTTTATAGACATCACTATGAAAGTAGGCATCCAAAAGTGAGTTC
AGAAGTACACATCCCATTAGGGGATGCTATGTTAGTAGTAAGAACATATTGGGGTCTGCTTACAGGAGAAAGA
GATTGGCACTTGGGTCATGGGGTCTCCATAGAATGGAGGCAGAAAAGATATAGCACACAAATAGATCCTGAAC
TAGCAGACCAACTAATTCACTCGCATTATTTTGACTGTTTTACAGACTCTGCTATAAGAAAAGCCACATTAGG
ACAAATAGTTAGACCTAAGTGTGAATATCAAGCAGGACATAATAAGGTAGGATCGCTACAATATTTGGCACTA
AAAGCATTAGTAACACCAGAAAGGACAAAGCCACCTTTACCTAGTGTTAAGAAGTTAACAGAAGACAGATGGA
ACAAGTCCCACAAGACCAGGGGCCACAGAGGGAGCCGTTCAATGAATGGACACTAGAACTGTTAGAAGAGCTT
AAGGATGAAGCTGTTAGACATTTTCCTAGGCCGTGGCTCCATGGATTAGGACAACATATCTACAACACATATG
GGGATACTTGGGAAGGGGTTGTAGCTATAATAAGAATTTTGCAACAACTACTGTTTATTCATTTCAGAATTGG
GTGTCAACATAGCAGAATAGGCATTATTCCAGGGAGAAGAGGCAGGAATGGAGCTGGTAGATCCTAGCCTAGA
GCCATGGAACCAACCGGGAAGTCAGCCTACAACTGCTTGTAACAATTGTTACTGTAAAATATGCTGCTGGCAT
TGCCAATTATGCTTTCTGAACAAGGGCTTAGGCATCTCCTATGGCAGGAAGAAGCGGAGACCCCGACGAAGAA
CTCCTCAAAGCCGTCAGGATCATCAAAATCCTGTACCAAAGCAGTGAGTAGTAATAATTAGTATATGTGATGA
AATCTTTAGGAATAGCTGCAATAGTAGGATTAGTAGTAGCATTCATAGCAGCCATAGTTGTGTGGACCATAGT
GTTTATTGAATATAGAAAAATAAGGAAACAGAAGAAAATAGACAAGATACTTGATAGAATAAGAGAAAGAGCA
GAAGACAGTGGCAATGAGAGTGATGGGGACACAGAAGAATTATACACTCTTATGGAGGTGGGGTATGATAATA
TTTTGGATAATGATAATTTGTAATGCTAAAGACTTGTGGGTCACAGTCTATTATGGGGTACCAGTGTGGAGAG
ACGCAGAGACCACCCTATTTTGTGCATCAGATGCCAAAGCATATGATTCAGAAGTACATAATGTCTGGGCTAC
ACATGCCTGTGTACCCACAGACCCTAACCCACAAGAAATACATTTGGAAAATGTAACAGAAAATTTTAACATG
TGGAAAAATACCATGGTGGAGCAGATGCATGAAGATATAATCAGTCTCTGGGACCAAAGCCTAAAGCCATGTG
TAAAGTTAACCCCGCTCTGCGTTACTTTAAATTGTAGTGACTATACATATAACTCCACCAGCTATGTTAAATC
CACCAACAACTCTGAAATGCAAGAAATAAAAAACTGCTCTTTCAATATAACCACAGAATTAAGAGATAAGAAA
CAGAAAATGTATGCACTTTTTTATAAACTTGATGTAAAACAACTTGATAATAATAATCAGACGTATAGTTTAA
TAAATTGTAACACCTCAACCATTACACAGGCTTGTCCAAAGGTATCCTTTGAGCCAATTCCCATACATTTTTG
TGCCCCAGCTGGTTTTGCAATTCTAAAGTGTAAGGATAAGAGGTTCAATGGAACAGGGCCATGCAAGAATGTC
AGCACAGTACAATGCACACATGGAATCAAGCCAGTAGTATCAACTCAACTGCTGTTAAATGGCAGTCTAGCAG
AAGAAGAGGTAGTGATTAGATCTGAGAATATCACAAACAATGCCAAGACCATAATAGTACAGTTGAATAAGCC
TGTAAGAATTAATTGTACCAGAACTGGTAACAATACAAGAAAAAGTGTACGTATAGGACCAGGGCAAACATAC
TATGCAACAGGTGAAATAATAGGAGATATAAGAAAAGCATATTGTAATGTCAGTAAAACAGAATGGGATGAGG
CTTTACAACAGGTAGTCACACAATTAAAGAAGCATTTCAATACCACAATAATCTTTAATAGTGCCTCAGGAGG
AGATCTAGAAATTACAACACATAGTTTTAATTGTAGAGGAGAATTTTTTTATTGCAATACAACAAACCTGTAT
AATAGCACTTGGAATAATAGCACTTGGAATGGCAATACCAGTACAAATCTCACAGAGTCAAATGATACTATAA
CTCTCCAATGCAGACTAAAGCAATTTATAAAAATGTGGCAGAGAGTAGGACAAGCAATGTATGCCCCTCCTAT
CCAAGGAGAAATAAGGTGTGATTCAAACATTACTGGACTATTATTAACAAGAGATGGAGGGAATAATAGGACA
AATGAGACCTTTAGGCCTGGAGGAGGAGATATGAGGGACAATTGGAGAAGTGAATTATATAAGTATAAAGTAG
TAAAAATTGAACCAATAGGTGTGGCACCAACCAGGGCAAAAAGAAGAATGGTGGAGAGAGAAAAAAGAGCAGT
TGGACTGGGAGCTGTTTTCCTTGGGTTCTTAGGAGCAGCAGGAAGCACTATGGGCGCGGCGTCAATAACGCTG
ACGGTACAGGCCAGGCAATTATTGTCTGGTATAGTGCAACAGCAGAGCAATTTGCTGAAGGCTATAGAGGCTC
AACAGCAAATGTTGAGACTCACGGTCTGGGGCATTAAGCAGCTCCAGGCAAGAGTCCTGGCTCTGGAAAGATA
CCTAAAGGATCAACAGCTCCTAGGAATTTGGGGCTGCTCTGGAAAACTCATCTGCACCACTGGTGTACGCTGG
AACTCAAGCTGGAGTAATAAAACTTATGAGGACATATGGGATAACATGACCTGGGTGCAATGGGAGAGGGAAA
TTAGCAATTACACAAACACAATATATACTCTACTTGAAGAATCGCAGAACCAGCAGGAAAAAAATGAACAAGA
CTTATTGGCATTGGACAAATGGACAAATCTGTGGAATTGGTTTAACATATCAAATTGGTTATGGTATATAAAA
ATATTTATAATGATAGTAGGAGGTTTAATAGGTTTAAGAATAGTTTTTACTGTGCTTACTATAATAAGGAGAG
TTAGGCAGGGATACTCACCTTTGTCGTTCCAGACCCTTCCCCTACACCAGAGGGAACCCGACAGGCCCGAAGG

AATCGAAGAAGGAGGTGGCGAGCAAGACAGAGGCAGATCAGTAAGATTAGTGAGCGGATTCTTAGCTCTTGCC
TGGGACGACCTACGGAGCCTGTGCCTCTTCAGCTACCACCAATTGAGAAACTTTGCCTTGATTGCAGCGAGAA
CTGTGGAACTCCTGGGACACAGCAGTCTCAAGGGACTGAGACTGGGGTGGGAAGCCCTCAAATATCTGTGGAA
TCTTCTGTCATACTGGGGTCAGGAACTAAAGAATAGTGCTATTAATTTGTTTGATACCATAGCAATAGCAGTA
GCTAACTGGACAGACAGAGGTATAGAAATAGGACAAAGAGCTGGCAGAGCTATTGGCAACATACCTAGAAGAA
TCAGACAGGGCCTAGAAAGAGCTTTGCTATAACATGGGTGGCAAATGGTCAAAAAGCAGCATAGTGGGATGGC
CTAAGATTAGGGAAAGGATGAGACAAAGCCCTCCAACAGGAAGCCCTCCAGCAGCGACAGGAACAGGAGCAGT
ATCCCCAGCAGCAACAGGAACAGGAGCAGTATCTCAAGATTTAGCTAGACATGGAGCAATCACAAGCAGTAAT
ACAGCCTCTACTAATCCTGCTTGTGCCTGGCTGGAAGCACAAGAGGAAGAGGATGAGGTAGGCTTTCCAGTCA
GACCACAGGTACCTTTGAGACCAATGACTTATAAACTAGCTTTCGATCTCAGCTTCTTTTTAAAAAAAAAGGG
GGGACTGGAAGGGTTAGTTTGGTCCCAGAAAAGACAAGATATCCTTGATCTGTGGATGTATCACACACAAGGA
ATCTTCCCAGATTGGCAGAACTACACACCAGGGCCAGGGACTAGATTCCCACTGACCTTTGGGTGGTGCTTCA
AACTAGTACCACTAGATCCAGCAGAGGTAGAGGCAGCTAATGAAGGAGAGAACAACAGCTTATTACACCCCAT
CAGTCAACATGGGATGGAAGACGGGGACAGAGAGGTGCTGGTCTGGAGATTTGACAGCAGCCTGGCAAGAGAA
CACAAAGCCCGAGAGCTGCATCCGGAGTTCTATAAAGACTGCTGACACAGAAGTTGCTGACTGGGACTTTCCA
CTGGGGACTTTCCGGGGAGGTGTGGTTGGGGAGGAGTTTGGGAGTGGCTAACCCTCAGATGCTGCATATAAGC
AGCTGCTTCTCGCCTGTACGTGGTCTCTCTTGCTAGACCAGATTTGAGCCTGGGAGCTCTCTGGCTAGTTAAG
GGACCCACTGCTTAAGCCTCAATAAAGCTTGCCT

>X-16A HIV-1 genome, derived from RNA genomic sequence
TAGCAGTGGCGCCCGAACAGGGACTTGAAGTTAATAGGGACTCGAAAGCGAAAGTTCCAGAGAAGCTCTCTCG
ACGCAGGGACTCGGCTTGCTGAAGTGCACGCAGCAAGAGGCGAGAGCGGCGACTGGTGAGTACGCCAAATTTT
GACTAGCGGAGGCTAGAAGGAGAGAGATGGGTGCGAGAGCGTCGATACTAAGTGGGGGAAAATTAGATGCATG
GGAAAAAATTCGGTTAAGGCCAGGGGGAAAGAAAAAATATCGACTAAAACATTTAGTATGGGCAAGCAGGGAG
TTGGAAAGATTTGCACTTAATCCTAGTCTTTTAGAAACAGCAGAAGGATGTCAACAACTGATAGAACAGTTAC
AATCAACTCTCAGGACAGGATCAGAAGAACTTAAATCATTATTTAATACAATAGCAACCCTTTGGTGCGTACA
TCAAAAGATAGACATAAAAGACACCAAGGAAGCCTTAGATAAAATAGAGGAAGAACAAAATAAGAGCAAGCAA
AAGGCACAGCAAAAGACACAGCAGGCAGCAGCTGCCGCAGGAAGCAGCAGCCAAAATTACCCTATAGTGCAAA
ATGCACAAGGGCAAATGATACATCAGGCCATGTCACCTAGAACTTTGAACGCATGGGTGAAGGTAGTAGAGGA
GAAGGCTTTCAGCCCAGAAGTAATACCTATGTTTACAGCATTATCAGAAGGAGCCACCCCACAAGACTTAAAT
ATGATGCTAAACATAGTGGGGGGACACCAGGCAGCAATGCAGATGTTAAAAGATACCATCAATGAGGAAGCTG
CAGAATGGGACAGGACACATCCTGTACATGCAGGGCCTGCTCCACCAGGCCAGATAAGAGAACCAAGGGGAAG
TGACATAGCAGGAACTACTAGTACCCTTCAAGAACAAATAGGATGGATGACAAGCAATCCACCTATCCCAGTG
GGAGAAATCTATAAAAGATGGATAGTCCTGGGATTAAATAAAATAGTAAGAATGTATAGCCCTGTCAGCATTT
TGGATATAAGACAAGGGCCAAAAGAACCCTTTAGAGACTATGTAGATAGGTTCTTTAAAACTTTAAGAGCTGA
ACAAGCTACACCGGAGGTAAAAAACTGGATGACAGAAACCTTGCTGGTCCAAAATGCGAATCCAGATTGTAAG
ACCATTTTAAGAGGATTAGGACCAGGGGCTACATTAGAAGAAATGATGACAGCATGTCAGGGAGTGGGAGGAC
CTGGCCATAAAGCAAGGGTTTTGGCTGAGGCTATGAGTCAAGCGCAACAGTCCAACATAATGATGCAGAAGGG
AAACTTTAGGGGCCAGAGAACAATAAAGTGTTTCAATTGTGGCAAAGAAGGACACCTAGCCAGAAACTGCAAG
GCCCCTAGGAAAAGGGGTTGTTGGAAATGTGGTAAGGAAGGACACCAAATGAAAGACTGTACTGAAAGACAGG
CTAATTTTTTAGGGAAAATTTGGCCTTCCAACAAGGGGAGGCCAGGAAATTTTCCTCAGAGCAGACCGGAACC
AACGGCCCCACCACTAGAGAACTTTGGAGTGGGGGAAGAGATACCCCCCCCTCAGAAGCAGGAACCCTCCTCT
CCGAAACAGGAACCGGGGGACAAGGGACTATATCCTCCTTTAACCTCCCTCAAATCACTCTTTGGCAACGACC
AGTAGTCACAGTAAGAATAGAGGGAGAGGTAATAGAAGCCCTATTAGACACAGGGGCAGATGATACAGTAATA
GAAAAGATAAATTTACCAGGAAAATGGAAACCAAAAATGATAGGGGGAATTGGAGGCTTTATCAAAGTAAGAC
AGTATGATCAGATAAATATAGAAATTTGTGGAAAAAGGGCCATAGGTACAGTATTAGTAGGACCTACACCTGT
CAACATAATTGGACGAAATATGTTGACTCAGATTGGTTGTACTTTAAATTTTCCAATAAGTCCTATTGAAACT
GTACCAGTAAAATTAAAGCCAGGAATGGATGGGCCAAAGGTTAAACAATGGCCATTGACAGAAGAAAAAATAA
AAGCATTAACAGAAATTTGTACAGATATGGAAAAGGAAGGAAAAATTTCAAGAATTGGGCCTGAAAATCCATA
CAATACTCCAGTATTTGCCATAAAGAAAAAAGATAGTACTAAATGGAGAAAATTAGTAGATTTCAGAGAACTC
AATAAGAGAACTCAAGACTTCTGGGAGGTCCAATTAGGAATACCTCATCCCGCAGGGTTAAAAAAGAAAAAAT
CAGTAACAGTATTAGATGTGGGGGATGCATATTTTTCAGTTCCCTTAGATAAGGACTTTAGAAAGTATACTGC
CTTCACTGTACCTAGTATAAATAATGAGACACCAGGAATTAGATATCAGTACAATGTGCTCCCACAGGGATGG
AAAGGATCACCAGCAATATTTCAGGCAAGTATGATAAAAATATTAGAGCCCTATAGAATAAATAATCCAGAGA
TAGTGATCTATCAATATATGGATGATTTATATGTAGGATCTGACTTAGAGATAGGGCAGCATAGAGCAAAAAT
AGAGGAGTTGAGAGCACATCTATTGAAGTGGGGATTTACCACACCAGACAAAAAGCATCAGAAAGAACCTCCA
TTTCTTTGGATGGGATATGAACTCCATCCTGACAAATGGACAGTCCAGCCCATACAGCTACCGGATAAAGACC
ACTGGACTGTCAATGATATACAGAAATTAGTGGGAAAACTAAATTGGGCAAGTCAGATTTATGCAGGAATTAA
AATAAAACAACTGTGTAAACTCCTCAGGGGAGCCAAAGCACTAACAGATATAGTAACAATGACTGAGGAAGCA
GAATTGGAATTGGCAGAGAACAGGGAAATTTTAAAAGAACCTGTACATGGAGTGTATTATGACCCAACAAAAG
ACTTAGTAGCAGAAGTACAGAAACAAGGGCAAGGCCAATGGACATATCAAATTTATCAAGAGCCATTTAAAAA
TCTAAAAACAGGAAAATATGCAAAAAGGAAGTCTGCCCACACTAATGATGTAAAACAGTTAGCAGAGGTAGTG
CAAAAAATAGCTGTAGAAAGCATAGTAATTTGGGGGAAGACCCCTAAATTTAGACTACCCATACAAAGAGAAA
CATGGGAAACATGGTGGACAGAGTATTGGCAGGCTACCTGGATACCTGACTGGGAGTTTGTCAATACCCCTCC
TTTAGTAAAATTATGGTACCAGTTAGAAAAAGACCCCATAGTAGGGGCAGAAACTTTCTATGTAGATGGGGCA
GCCAATAGGGAAACTAAGCTAGGAAAAGCAGGGTATGTCACAGACAGAGGAAGACAAAAGGTTGTCTCCCTAA
CTGAGACAACAAATCAAAAGACTGAATTACAGGCAATATATCTAGCCTTGCAAGATTCAGGATCAGAAGTAAA
TATAGTGACAGACTCACAATATGCATTGGGAATTATTCAGGCACAACCAGACAGAAGTGAATCAGAATTAGTT
AATCAAGTAATAGAGAAGCTAATAGAAAAGGACAAGGTCTACCTGTCATGGGTACCAGCACATAAAAGGATTG
GAGGAAATGAACAAGTAGATAAATTGGTCAGTAATGGAATCAGAAGAGTACTATTTTTAGATGGCATAGATAA
AGCCCAAGAAGATCACGAAAAATATCACAGCAATTGGAGAACAATGGCTAGTGATTTTAATCTGCCACCTATA
GTAGCGAAAGAAATAGTGGCCAGCTGTGATAAATGTCAGCTGAAAGGGGAAGCCATACATGGACAAGTAGACT
GTAGTCCAGGAATATGGCAATTAGATTGTACACATTTAGAAGGAAAAATTATCCTGGTAGCAGTCCATGTAGC

CAGTGGCTATATAGAAGCAGAAGTTATCCCAGCAGAAACAGGATCAGAGACAGCATATTTTATATTAAAATTA
GCAGGAAGATGGCCAGTGAAAGTAATACACACAGACAATGGCCCCAATTTTACCAGTGCTGCAGTAAAGGCAG
CATGTTGGTGGGCAAATGTCACACAAGAATTTGGAATTCCCTACAATCCCCAAAGCCAAGGAGTAGTGGAATC
TATGAATAAAGAACTAAAGAAAATTATAGGACAGGTTAGGGATCAAGCTGAACATCTTAAGACAGCAGTACAG
ATGGCAGTATTCATTCACAATTTTAAAAGAAAAGGGGGGATTGGGGGGTACAGTGCAGGGGAAAGAATAATAG
ACATAATAGCATCAGATATACAAACTAAAGAACTACAAAAACAAATTACAAAAATTCAAAATTTTCGGGTTTA
TTACAGGGACAGCAGAGACCCCATTTGGAAAGGACCAGCAAAACTACTCTGGAAAGGTGAAGGGGCAGTAGTA
ATACAGGACAATAGTGATATAAAAGTAGTACCAAGAAGAAAAGCAAAAATCATTAAGGATTATGGAAAACAGA
TGGCAGGTGATGATTGTGTGGCAGGTAGACAGGATGAGGATTAGAACATGGAACAGTTTAGTAAAACATCATA
TGTATGTCTCTAAGAAAGCTAAGGGTTGGTTTTATAGACATCACTATGAAAGTAGGCATCCAAAAGTGAGTTC
AGAAGTACACATCCCATTAGGGGATGCTGTGTTAGTAGTAAGAACATATTGGGGTCTGCTTACAGGAGAAAGA
GATTGGCACTTGGGTCATGGGGTCTCCATAGAATGGAGGCAGAAAAGATATAGCACACAAATAGATCCTGAAC
TAGCAGACCAACTAATTCACTCGCATTATTTTGACTGTTTTACAGACTCTGCTATAAGAAAAGCCACATTAGG
ACAAATAGTTAGACCTAAGTGTGAATATCAAGCAGGACATAATAAGGTAGGATCGCTACAATATTTGGCACTA
AAAGCATTAGTAACACCAGAAAGGACACAGCCACCTTTACCTAGTGTTAAGAAGTTAACAGAAGACAGATGGA
ACAAGTCCCACAAGACCAGGGGCCACAGAGGGAGCCGCTCAATGAATGGACACTAGAACTGTTAGAAGAGCTT
AAGGAAGAAGCTGTTAGACATTTTCCTAGGCCGTGGCTCCATGGATTAGGACAACATATCTACAACACATATG
GGGATACTTGGGAAGGGGTTGTAGCTATAATAAGAATTTTGCAACAACTACTGTTTATTCATTTCAGAATTGG
GTGTCAACATAGCAGAATAGGCATTATTCCAGGGAGAAGAGGCAGGAATGGAGCTGGTAGATCCTAGCCTAGA
GCCATGGAACCATCCGGGAAGTCAGCCTACAACTGCTTGTAACAATTGTTACTGTAAAATATGCTGCTGGCAT
TGCCAATTATGCTTTTTGAACAAGGGCTTAGGCATCTCCTATGGCAGGAAGAAGCGGAGACCCCGACGAAGAA
CTCCTCAAAGCCGTCAAGATCATCAAAATCCTGTACCAAAGCAGTGAGTAGTAATAATTAGTATATGTAATGA
AATCTTTAGGAATAGCTGCAATAGTAGGATTAGTAGTAGCATTCATAGCAGCCATAGTTGTGTGGACCATAGT
GTTTATTGAATATAGAAAAATAAGGAAACAGAAGAAAATAGACAAGATACTTGATAGAATAAGAGAAAGAGCA
GAAGACAGTGGCAATGAGAGTGATGGGGACACAGAAGAATTATACACTCTTATGGAGGTGGGGTATGATAATA
TTTTGGATAATGATAATTTGTAATGCTAAAGACTTGTGGGTCACAGTCTATTATGGGGTACCAGTGTGGAGAG
ACGCAGAGACCACCCTATTTTGTGCATCAGATGCCAAAGCATATGATTCAGAAGTACATAATGTCTGGGCTAC
ACATGCCTGTGTACCCACAGACCCTAACCCACAAGAAATACATTTGGAAAATGTAACAGAAAATTTTAACATG
TGGAAAAATACCATGGTGGAGCAGATGCATGAAGATATAATCAGTCTCTGGGACCAAAGCCTAAAGCCATGTG
TACAGTTAACCCCGCTCTGCGTTACTTTAAATTGTAGTGACTATACAAATAATTCCACCAAACCCACCAACAT
CTCTGAAATGCAAGAAATAAAAAACTGCTCTTTCAATATAACCACAGAATTAAGAGATAAGAAACAGAAAATG
TATGCACTTTTTTATAAACTTGATGTAAAACAACTTGATAATAATAATCAGACGTATAGTTTAATAAATTGTA
ACACCTCAACCATTACACAGGCTTGTCCAAAGGTATCCTTTGAGCCAATTCCCATACATTATTGTGCCCCAGC
TGGTTTTGCAATTCTAAAGTGTAAGGATAAGATGTTCAATGGAACAGGGCCATGCAAGAATGTCAGCACAGTA
CAATGCACACATGGAATCAAGCCAGTAGTATCAACTCAACTGCTGTTAAATGGCAGTCTAGCAGAAGGAGAGG
TAGTGATTAGATCTGAGAATATCACAAACAATGCCAAGACCATAATAGTACAGTTGAATAAGCCTGTAAGAAT
TAATTGTACCAGAACTAGTAACAATACAAGAAAAAGTGTACGTATAGGACCAGGGCAAACATACTATGCAACA
GGTGAAATAATAGGAGATATAAGAAAAGCACATTGTAATGTCAGTAAAACAGAATGGGATAAGGCTTTACAAC
AGGTAGCCACACAATTACAGAAGCATTTCAATACCACAATAGTCTTTAATAATGCCTCAGGAGGAGATCTAGA
AATTACAACACATAGTTTTAATTGTAGAGGAGAATTTTTTTATTGCAATACAACAAACCTGTATAATAGCACT
TGGAATAGCACTTGGACAAATCTCACAGAGTCAAATGATACTATAACTCTCCAATGCAGACTAAAGCAATTTA
TAAAAATGTGGCAGAGAGTAGGACAAGCAATGTATGCCCCTCCTATCCAAGGAGAAATAAGGTGTGATTCAAA
CATTACTGGACTATTATTAACAAGAGATGGAGGGAATAATGGGACAAATGAGACCTTTAGGCCTGGAGGAGGA
GATATGAGGGACAATTGGAGAAGTGAATTATATAAGTATAAAGTAGTAAAAATTGAACCAATAGGTGTGGCAC
CAACCAGGGCAAAAAGAAGAATGGTGGAGAGAGAAAAAAGAGCAGTTGGACTGGGAGCTGTTTTCCTTGGGTT
CTTAGGAGCAGCAGGAAGCACTATGGGCGCGGCGTCAATAACGCTGACGGTACAGGCCAGGCAATTATTGTCT
GGTATAGTGCAACAGCAGAGCAATTTGCTGAAGGCTATAGAGGCTCAACAGCAAATGTTGAGACTCACGGTCT
GGGGCATTAAGCAGCTCCAGGCAAGAGTCCTGGCTCTGGAAAGATACCTAAAGGATCAACAGCTCCTAGGAAT
TTGGGGCTGCTCTGGAAAACTCATCTGCACCACTGGTGTACGCTGGAACTCAAGCTGGAGTAATAAAACTTAT
GAGGACATATGGGATAACATGACCTGGGTGCAATGGGAGAGGGAAATTAGCAATTACACAAACACAATATATA
CTCTACTTGAAGAATCGCAGAACCAGCAGGAAAAAAATGAACAAGACTTATTGGCATTGGAAAAATGGACAAA
TCTGTGGAATTGGTTTAACATATCAAATTGGTTATGGTATATAAAAATATTTATAATGATAGTAGGAGGTTTA
ATAGGTTTAAGAATAGTTTTTACTGTGCTTACTATAATAAGGAGAGTTAGGCAGGGATACTCACCTTTGTCGT
TCCAGACCCTTCCCCTACACCAGAGGGAACCCGACAGGCCCGAAGGAATCGAAGAAGGAGGTGGCGAGCAAGA

CAGAGGCAGATCAATAAGATTAGTGAGCGGATTCTTAGCTCTTGCCTGGGACGACCTACGGAGCCTGTGCCTT
TTCAGCTACCACCAATTGAGAAACTTTGCCTTGATTGCAGCGAGAACTGTGGAACTCCTGGGACACAGCAGTC
TCAAGGGACTGAGACTGGGGTGGGAAGCCCTCAAATATCTGTGGAATCTTCTGTCATACTGGGGTCAGGAACT
AAAGAATAGTGCTATTAATTTGTTTGATACCATAGCAATAGCAGTAGCTAACTGGACAGACAGAGGTATAGAA
ATAGGACAAAGAGTTGGCAGAGCTATTGGCAACATACCTAGAAGAATCAGACAGGGCCTAGAAAGAGCTTTGC
TATAACATGGGTGGCAAATGGTCAAAAAGCAGCATAGTGGGATGGCCTAAGATTAGGGAAAGGATGAGACGAA
CCCCTCCAACAGGAAGCCCTCCAACAGCAACAGAAACAGGAGCAGTATCCCCAGCAGCAACAGGAACAGGAGC
AGTATCTCAAGATTTAGCTAGACATGGAGCAATCACAAGCAGTAATACAGCCTCTACTAATTCTGCTTGTGCC
TGGCTGGAAGCACAAGAGGAAGAGGATGAGGTAGGCTTTCCAGTCAAACCACAGGTACCTTTGAGACCAATGA
CTTATAAACTAGCTTTCGATCTCAGCTTCTTTTTAAAAGAAAAGGGGGGACTGGAAGGGTTAGTTTATTCCCA
GAAAAGACAAGATATCCTTGATCTGTGGATGTATCACACACAAGGAATCTTCCCAGATTGGCAGAATTACACA
CCAGGGCCAGGGACTAGATACCCACTGACCTTTGGGTGGTGCTTCAAACTAGTACCACTAGATCCAGCAGAGA
TAGAGGCAGCTAATGAAGGAGAGAACAACAGCTTATTACACCCCATCAGTCAACATGGGATAGAAGACGGGGA
CAGAGAGGTGCTGGTCTGGAGATTTGACAGCAGCCTGGCAAGAGAACACAAAGCCCGAGAGCTGCATCCGGAG
TTCTATAAAGACTGCTGACACAGAAGTTGCTGACTGGGACTTTCCACTGGGGACTTTCCGGGGAGGTGTGGTT
GGGGAGGAGTTTGGGAGTGGCTAACCCTCAGATGCTGCATATAAGCAGCTGCTTCTCGCCTGTACGTGGTCTC
TCTTGCTAGACCAGATTTGAGCCTGGGAGCTCTCTGGCTAGTTAAGGGACCCACTGCTTAAGCCTCAATAAAG
CTTGCCT

>X-16B HIV-1 genome, derived from RNA genomic sequence
TAGCAGTGGCGCCCGAACAGGGACTTGAAGTTAATAGGGACTCGAAAGCGAAAGTTCCAGAGAAGCTCTCTCG
ACGCAGGGACTCGGCTTGCTGAAGTGCACGCAGCAAGAGGCGAGAGCGGCGACTGGTGAGTACGCCAAATTTC
GACTAGCGGAGGCTAGAAGGAGAGAGATGGGTGCGAGAGCGTCGATACTAAGTGGGGGAAAATTAGATGCATG
GGAGAAAATTCGGTTAAGGCCAGGGGGAAAGAAAAAATATCGACTAAAACATTTAGTATGGGCAAGCAGGGAG
TTGGAAAGATTTGCACTTAATCCTAGTCTTTTAGAAACAGCAGAAGGATGTCAACAACTGATGGAACAGTTAC
AATCAACTCTCAGGACAGGATCAGAAGAACTTAAATCATTATTTAATACAATAGCAACCCTTTGGTGCGTACA
TCAAAAGATAGACATAAAAGACACCAAGGAAGCCTTAGATAAAATAGAGGAAGAACAAAATAAGAGCAAGCAA
AAGACACAGCAAAAGACACAGCAGGCAGCAGCTGCCGCAGGAAGCAGCAGCCAAAATTACCCTATAGTGCAAA
ATGCACAAGGGCAAATGATACATCAGGCCATGTCACCTAGAACTTTGAACGCATGGGTGAAGGTAGTAGAGGA
GAAGGCTTTCAGCCCAGAAGTAATACCCATGTTTACAGCATTATCAGAAGGAGCCACCCCACAAGACTTAAAT
ATGATGCTAAACATAGTGGGGGGACACCAGGCAGCAATGCAGATGTTAAAAGATACCATCAATGAGGAAGCTG
CAGAATGGGACAGGACACATCCTGTACATGCAGGGCCTGCTCCACCAGGCCAGATAAGAGAACCAAGGGGAAG
TGACATAGCAGGAACTACTAGTACCCTTCAAGAACAAATAGGATGGATGACAAGCAATCCACCTATCCCAGTG
GGAGAAATCTATAAAAGATGGATAGTCCTGGGATTAAATAAAATAGTAAGAATGTATAGCCCTGTCAGCATTT
TGGATATAAGACAAGGGCCAAAAGAACCCTTTAGAGACTATGTAGATAGGTTCTTTAAAACTTTAAGAGCTGA
ACAAGCTACACAGGATGTAAAAAACTGGATGACAGAAACCTTGCTGGTCCAAAATGCGAATCCAGATTGTAAG
ACCATTCTAAGAGCATTAGGACCAGGGGCTACATTAGAAGAAATGATGACAGCATGTCAGGGAGTGGGAGGAC
CTGGCCATAAAGCAAGGGTTTTGGCTGAGGCTATGAGTCAAGCGCAACAGTCCAACATAATGATGCAGAAGGG
AAACTTTAGGGGCCAGAGAACAATAAAGTGTTTCAATTGTGGCAAAGAAGGACACCTAGCCAGAAACTGCAAG
GCCCCTAGGAAAAGGGGTTGTTGGAAATGTGGTAAGGAAGGACACCAAATGAAAGACTGTACTGAAAGACAGG
CTAATTTTTTAGGGAAAATTTGGCCTTCCAACAAGGGGAGGCCAGGAAATTTTCCTCAGAGCAGACCGGAACC
AACGGCCCCACCACTAGAGAACTTTGGAGTGGGGGAAGAGATACCCCCCCCTCAGAAGCAGGAACCCTCCTCT
CCGAAACAGGAACCGGGGGACAAGGGACTATATCCTCCTTTAACCTCCCTCAAATCACTCTTTGGCAACGACC
AGTAGTCACAGTAAGAATAGAGGGAGAGGTAATAGAAGCCCTATTAGACACAGGGGCAGATGATACAGTAATA
GAAAAGATAAATTTACCAGGAAAATGGAAACCAAAAATGATAGGGGGAATTGGAGGCTTTATCAAAGTAAGAC
AGTATGATCAGATAAGTATAGAAATTTGTGGAAAAAGGGCCATAGGTACAGTATTAGTAGGACCTACACCTGT
CAACATAATTGGACGAAATATGTTGACTCAGATTGGTTGTACTTTAAATTTTCCAATAAGTCCTATTGAAACT
GTACCAGTAAAATTAAAGCCAGGAATGGATGGGCCAAAGGTTAAACAATGGCCATTGACAGAAGAAAAAATAA
AAGCATTAACAGAAATTTGTACAGATATGGAAAAGGAAGGAAAAATTTCAAGAATTGGGCCTGAAAATCCATA
CAATACTCCAGTATTTGCCATAAAGAAAAAAGATAGTACTAAATGGAGAAAATTAGTAGATTTCAGAGAACTC
AATAAGAGAACTCAAGACTTCTGGGAGGTCCAATTAGGAATACCTCATCCCGCAGGGTTAAAAAAGAAAAAAT
CAGTAACAGTATTAGATGTGGGGGATGCATATTTTTCAGTTCCCTTAGATAAGGACTTTAGAAAGTATACTGC
CTTCACTATACCTAGTATAAATAATGAGACACCAGGAATTAGATATCAGTACAATGTGCTCCCACAGGGATGG
AAAGGATCACCAGCAATATTTCAGGCAAGTATGACAAAAATATTAGAGCCCTATAGAATAAATAATCCAGAGA
TGGTGATCTATCAATATATGGATGATTTATATGTAGGATCTGACTTAGAGATAGGGCAGCATAGAGCAAAAAT
AGAGGAGTTGAGAGCACATCTATTGAAGTGGGGATTTACCACACCAGACAAAAAGCATCAGAAAGAACCTCCA
TTTCTTTGGATGGGATATGAACTCCATCCTGACAAATGGACAGTCCAGCCTATACAGCTACCGGATAAAGACC
ACTGGACTGTCAATGATATACAGAAATTAGTGGGAAAACTAAATTGGGCAAGTCAGATTTATGCAGGAATTAA
AGTAAAACAACTGTGTAAACTCCTCAGGGGAGCCAAAGCACTAACAGATATAGTACCAATGACTGAGGAAGCA
GAATTGGAATTGGCAGAGAACAGGGAAATTTTAAAAGAACCTGTACATGGAGTGTATTATGACCCAACAAAAG
ACTTAGTAGCAGAAGTACAGAAACAAGGGCAAGGCCAATGGACATATCAAATTTATCAAGAGCCATTTAAAAA
TCTAAAAACAGGAAAATATGCAAAAAGGAAGTCTGCCCACACTAATGATGTAAAACAGTTAGCAGAGGTAGTG
CAAAAAATAGCTGTAGAAAGCATAGTAATTTGGGGGAAGACCCCTAAATTTAGACTACCCATACAAAGAGAAA
CATGGGAAACATGGTGGACAGAGTATTGGCAGGCTACCTGGATACCTGACTGGGAGTTTGTCAATACCCCTCC
TTTAGTAAAATTATGGTACCAGTTAGAGAAAGACCCCATAGTAGGGGCAGAAACTTTCTATGTAGATGGGGCA
GCCAATAGGGAAACTAAGCTAGGAAAAGCAGGGTATGTCACAGACAGAGGAAGACAAAAGGTTGTCTCCCTAA
CTGAGACAACAAATCAAAAGACTGAATTACAGGCAATATATCTAGCCTTGCAAGATTCAGGATCAGAAGTAAA
TATAGTGACAGACTCACAATATGCATTGGGAATTATTCAGGCACAACCAGACAGAAGTGAATCAGAATTAGTT
AATCAAGTAATAGAGAAGCTAATAGAAAAGGACAAGGTCTACCTGTCATGGGTACCAGCACATAAAGGGATTG
GAGGAAATGAACAAGTAGATAAATTGGTCAGTAATGGAATCAGAAGAGTACTATTTTTAGATGGCATAGATAA
AGCCCAAGAAGATCACGAAAGATATCACAGCAATTGGAGAACAATGGCTAGTGATTTTAATCTGCCACCTATA
GTAGCAAAAGAAATAGTGGCCAGCTGTGATAAATGTCAGCTGAAAGGGGAAGCCATACATGGACAAATAGACT
GTAGTCCAGGAATATGGCAATTAGATTGTACACATTTAGAAGGAAAAATTATCCTGGTAGCAGTCCATGTAGC

CAGTGGCTATATAGAAGCAGAAGTTATCCCAGCAGAAACAGGATCGGAGACAGCATACTTTATATTAAAATTA
GCAGGAAGATGGCCAGTGAAAGTAATACACACAGACAATGGCCCCAATTTTATCAGTGCTGCAGTAAAGGCAG
CATGTTGGTGGGCAAATGTCACACAAGAATTTGGAATTCCCTACAATCCCCAAAGCCAAGGAGTAGTGGAATC
TATGAATAAAGAACTAAAGAAAATTATAGGACAGGTCAGGGATCAAGCTGAACATCTTAAGACAGCAGTACAG
ATGGCAGTATTCATTCACAATTTTAAAAGAAAAGGGGGGATTGGGGGGTACAGTGCAGGGGAAAGAATAATAG
ACATAATAGCATCAGATATACAAACTAAAGAACTACAAAAACAAATTACAAAAATTCAAAATTTTCGGGTTTA
TTACAGGGACAGCAGAGACCCCATTTGGAAAGGACCAGCAAAACTACTCTGGAAAGGTGAAGGGGCAGTAGTA
ATACAGGACAATAGTGATATAAAAGTAGTACCAAGAAGAAAAGCAAAAATCATTAAGGATTATGGAAAACAGA
TGGCAGGTGATGATTGTGTGGCAGGTAGACAGGATGAGGATTAGAACATGGAACAGTTTAGTAAAACATCATA
TGTATGTCTCTAGGAAAGCTAAGGGTTGGTTTTATAGACATCACTATGAAAGTAGGCATCCAAAAGTGAGTTC
AGAAGTACACATCCCATTAGGGGATGCTATGTTAGTAGTAAGAACATATTGGGGTCTGCTTACAGGAGAAAGA
GATTGGCACTTGGGTCATGGGGTCTCCATAGAATGGAGGCAGAAAAGATATAGCACACAAATAGATCCTGAAC
TAGCAGACCAACTAATTCACTCGCATTATTTTGACTGTTTTACAGACTCTGCTATAAGAAAAGCCACATTAGG
ACAAATAGTTAGACCTAAGTGTGAATATCAAGCAGGACATAATAAGGTAGGATCGCTACAATATTTGGCACTA
AAAGCATTAGTAACACCAGAAAGGACAAAGCCACCTTTACCTAGTGTTAAGAAGTTAACAGAAGACAGATGGA
ACAAGTCCCACAAGACCAGGGGCCACAGAGGGAGCCGTTCAATGAATGGACACTAGAACTGTTAGAAGAGCTT
AAGGATGAAGCTGTTAGACATTTTCCTAGGCCGTGGCTCCATGGATTAGGACAACATATCTACAACACATATG
GGGATACTTGGGAAGGGGTTGTAGCTATAATAAGAATTTTGCAACAACTACTGTTTATTCATTTCAGAATTGG
GTGTCAACATAGCAGAATAGGCATTATTCCAGGGAGAAGAGGCAGGAATGGAGCTGGTAGATCCTAGCCTAGA
GCCATGGAACCAACCGGGAAGTCAGCCTACAACTGCTTGTAACAATTGTTACTGTAAAATATGCTGCTGGCAT
TGCCAATTATGCTTTCTGAACAAGGGCTTAGGCATCTCCTATGGCAGGAAGAAGCGGAGACCCCGACGAAGAA
CTCCTCAAAGCCGTCAGGATCATCAAAATCCTGTACCAAAGCAGTGAGTAGTAATAATTAGTATATGTGATGA
AATCTTTAGGAATAGCTGCAATAGTAGGATTAGTAGTAGCATTCATAGCAGCCATAGTTGTGTGGACCATAGT
GTTTATTGAATATAGAAAAATAAGGAAACAGAAGAAAATAGACAAGATACTTGATAGAATAAGAGAAAGAGCA
GAAGACAGTGGCAATGAGAGTGATGGGGACACAGAAGAATTATACACTCTTATGGAGGTGGGGTATGATAATA
TTTTGGATAATGATAATTTGTAATGCTAAAGACTTGTGGGTCACAGTCTATTATGGGGTACCAGTGTGGAGAG
ACGCAGAGACCACCCTATTTTGTGCATCAGATGCCAAAGCATATGATTCAGAAGTACATAATGTCTGGGCTAC
ACATGCCTGTGTACCCACAGACCCTAACCCACAAGAAATACATTTGGAAAATGTAACAGAAAATTTTAACATG
TGGAAAAATACCATGGTGGAGCAGATGCATGAAGATATAATCAGTCTCTGGGACCAAAGCCTAAAGCCATGTG
TAAAGTTAACCCCGCTCTGCGTTACTTTAAATTGTAGTGACTATACATATAACTCCACCAGCTATGTTAAATC
CACCAACAACTCTGAAATGCAAGAAATAAAAAACTGCTCTTTCAATATAACCACAGAATTAAGAGATAAGAAA
CAGAAAATGTATGCACTTTTTTATAAACTTGATGTAAAACAACTTGATAATAATAATCAGACGTATAGTTTAA
TAAATTGTAACACCTCAACCATTACACAGGCTTGTCCAAAGGTATCCTTTGAGCCAATTCCCATACATTATTG
TGCCCCAGCTGGTTTTGCAATTCTAAAGTGTAAGGATAAGAGGTTCAATGGAACAGGGCCATGCAAGAATGTC
AGCACAGTACAATGCACACATGGAATCAAGCCAGTAGTATCAACTCAACTGCTGTTAAATGGCAGTCTAGCAG
AAGAAGAGGTAGTGATTAGATCTGAGAATATCACAAACAATGCCAAGACCATAATAGTACAGTTGAATAAGCC
TGTAAGAATTAATTGTACCAGAACTGGTAACAATACAAGAAAAAGTGTACGTATAGGACCAGGGCAAACATAC
TATGCAACAGGTGAAATAATAGGAGATATAAGAAAAGCATATTGTAATGTCAGTAAAACAGAATGGGATGAGG
CTTTACAACAGGTAGTCACACAATTAAAGAAGCATTTCAATACCACAATAATCTTTAATAGTGCCTCAGGAGG
AGATCTAGAAATTACAACACATAGTTTTAATTGTAGAGGAGAATTTTTTTATTGCAATACAACAAACCTGTAT
AATAGCACTTGGAATAATAGCACTTGGAATGGCAATACCAGTACAAATCTCACAGAGTCAAATGATACTATAA
CTCTCCAATGCAGACTAAAGCAATTTATAAAAATGTGGCAGAGAGTAGGACAAGCAATGTATGCCCCTCCTAT
CCAAGGAGAAATAAGGTGTGATTCAAACATTACTGGACTATTATTAACAAGAGATGGAGGGAATAATAGGACA
AATGAGACCTTTAGGCCTGGAGGAGGAGATATGAGGGACAATTGGAGAAGTGAATTATATAAGTATAAAGTAG
TAAAAATTGAACCAATAGGTGTGGCACCAACCAGGGCAAAAAGAAGAATGGTGGAGAGAGAAAAAAGAGCAGT
TGGACTGGGAGCTGTTTTCCTTGGGTTCTTAGGAGCAGCAGGAAGCACTATGGGCGCGGCGTCAATAACGCTG
ACGGTACAGGCCAGGCAATTATTGTCTGGTATAGTGCAACAGCAGAGCAATTTGCTGAAGGCTATAGAGGCTC
AACAGCAAATGTTGAGACTCACGGTCTGGGGCATTAAGCAGCTCCAGGCAAGAGTCCTGGCTCTGGAAAGATA
CCTAAAGGATCAACAGCTCCTAGGAATTTGGGGCTGCTCTGGAAAACTCATCTGCACCACTGGTGTACGCTGG
AACTCAAGCTGGAGTAATAAAACTTATGAGGACATATGGGATAACATGACCTGGGTGCAATGGGAGAGGGAAA
TTAGCAATTACACAAACACAATATATACTCTACTTGAAGAATCGCAGAACCAGCAGGAAAAAAATGAACAAGA
CTTATTGGCATTGGACAAATGGACAAATCTGTGGAATTGGTTTAACATATCAAATTGGTTATGGTATATAAAA
ATATTTATAATGATAGTAGGAGGTTTAATAGGTTTAAGAATAGTTTTTACTGTGCTTACTATAATAAGGAGAG
TTAGGCAGGGATACTCACCTTTGTCGTTCCAGACCCTTCCCCTACACCAGAGGGAACCCGACAGGCCCGAAGG

AATCGAAGAAGGAGGTGGCGAGCAAGACAGAGGCAGATCAGTAAGATTAGTGAGCGGATTCTTAGCTCTTGCC
TGGGACGACCTACGGAGCCTGTGCCTCTTCAGCTACCACCAATTGAGAAACTTTGCCTTGATTGCAGCGAGAA
CTGTGGAACTCCTGGGACACAGCAGTCTCAAGGGACTGAGACTGGGGTGGGAAGCCCTCAAATATCTGTGGAA
TCTTCTGTCATACTGGGGTCAGGAACTAAAGAATAGTGCTATTAATTTGTTTGATACCATAGCAATAGCAGTA
GCTAACTGGACAGACAGAGGTATAGAAATAGGACAAAGAGCTGGCAGAGCTATTGGCAACATACCTAGAAGAA
TCAGACAGGGCCTAGAAAGAGCTTTGCTATAACATGGGTGGCAAATGGTCAAAAAGCAGCATAGTGGGATGGC
CTAAGATTAGGGAAAGGATGAGACAAAGCCCTCCAACAGGAAGCCCTCCAGCAGCGACAGGAACAGGAGCAGT
ATCCCCAGCAGCAACAGGAACAGGAGCAGTATCTCAAGATTTAGCTAGACATGGAGCAATCACAAGCAGTAAT
ACAGCCTCTACTAATCCTGCTTGTGCCTGGCTGGAAGCACAAGAGGAAGAGGATGAGGTAGGCTTTCCAGTCA
GACCACAGGTACCTTTGAGACCAATGACTTATAAACTAGCTTTCGATCTCAGCTTCTTTTTAAAAAAAAAGGG
GGGACTGGAAGGGTTAGTTTGGTCCCAGAAAAGACAAGATATCCTTGATCTGTGGATGTATCACACACAAGGA
ATCTTCCCAGATTGGCAGAACTACACACCAGGGCCAGGGACTAGATTCCCACTGACCTTTGGGTGGTGCTTCA
AACTAGTACCACTAGATCCAGCAGAGGTAGAGGCAGCTAATGAAGGAGAGAACAACAGCTTATTACACCCCAT
CAGTCAACATGGGATGGAAGACGGGGACAGAGAGGTGCTGGTCTGGAGATTTGACAGCAGCCTGGCAAGAGAA
CACAAAGCCCGAGAGCTGCATCCGGAGTTCTATAAAGACTGCTGACACAGAAGTTGCTGACTGGGACTTTCCA
CTGGGGACTTTCCGGGGAGGTGTGGTTGGGGAGGAGTTTGGGAGTGGCTAACCCTCAGATGCTGCATATAAGC
AGCTGCTTCTCGCCTGTACGTGGTCTCTCTTGCTAGACCAGATTTGAGCCTGGGAGCTCTCTGGCTAGTTAAG
GGACCCACTGCTTAAGCCTCAATAAAGCTTGCCT

>X-21B HIV-1 genome, derived from RNA genomic sequence
TAGCAGTGGCGCCCGAACAGGGACTTGAAGTTAATAGGGACTCGAAAGCGAAAGTTCCAGAGAAGCTCTTTCG
ACGCAGGGACTCGGCTTGCTGAAGTGCACGCAGCAAGAGGCGAGAGCGGCGACTGGTGAGTACGCCAAATTTC
GACTAGCGGAGGCTAGAAGGAGAGAGATGGGTGCGAGAGCGTCGATACTAAGTGGGGGAAAATTAGATGCATG
GGAGAAAATTCGGTTAAGGCCAGGGGGAAAGAAAAAATATCGACTAAAACATTTAGTATGGGCAAGCAGGGAG
TTGGAAAGATTTGCACTTAATCCTAGTCTTTTAGAAACAGCAGAAGGATGTCAACAACTGATGGAACAGTTAC
AATCAACTCTCAGGACAGGATCAGAAGAACTTAAATCATTATTTAATACAATAGCAACCCTTTGGTGCGTACA
TCAAAAGATAGACATAAAAGACACCAAGGAAGCCTTAGATAAAATAGAGGAAGAACAAAATAAGAGCAAGCAA
AAGACACAGCAAAAGACACAGCAGGCAGCAGCTGCCGCAGGAAGCAGCAGCCAAAATTACCCTATAGTGCAAA
ATGCACAAGGGCAAATGATACATCAGGCCATGTCACCTAGAACTTTGAACGCATGGGTGAAGGTAGTAGAGGA
GAAGGCTTTCAGCCCAGAAGTAATACCCATGTTTACAGCATTATCAGAAGGAGCCACCCCACAAGACTTAAAT
ATGATGCTAAACATAGTGGGGGGACACCAGGCAGCAATGCAGATGTTAAAAGATACCATCAATGAGGAAGCTG
CAGAATGGGACAGGACACATCCTGTACATGCAGGGCCTGCTCCACCAGGCCAGATAAGAGAACCAAGGGGAAG
TGACATAGCAGGAACTACTAGTACCCTTCAAGAACAAATAGGATGGATGACAAGCAATCCACCTATCCCAGTG
GGAGAAATCTATAAAAGATGGATAGTCCTGGGATTAAATAAAATAGTAAGAATGTATAGCCCTGTCAGCATTT
TGGATATAAGACAAGGGCCAAAAGAACCCTTTAGAGACTATGTAGATAGGTTCTTTAAAACCTTAAGAGCTGA
ACAAGCTACACAGGATGTAAAAAACTGGATGACAGAAACCTTGCTGGTCCAAAATGCGAATCCAGATTGTAAG
ACCATTCTAAGAGCATTAGGACCAGGGGCTACATTAGAAGAAATGATGACAGCATGTCAGGGAGTGGGAGGAC
CTGGCCATAAAGCAAGGGTTTTGGCTGAGGCTATGAGTCAAGCGCAACAGTCCAACATAATGATGCAGAAGGG
AAACTTTAGGGGCCAGAGAACAATAAAGTGTTTCAATTGTGGCAAAGAAGGACACCTAGCCAGAAACTGCAAG
GCCCCTAGGAAAAGGGGTTGTTGGAAATGTGGTAAGGAAGGACACCAAATGAAAGACTGTACTGAAAGACAGG
CTAATTTTTTAGGGAAAATTTGGCCTTCCAACAAGGGGAGGCCAGGAAATTTTCCTCAGAGCAGACCGGAACC
AACGGCCCCACCACTAGAGAACTTTGGAGTGGGGGAAGAGATACCCCCCCCTCAGAAGCAGGAACCCTCCTCT
CCGAAACAGGAACCGGGGGACAAGGGACTATATCCTCCTTTAACCTCCCTCAAATCACTCTTTGGCAACGACC
AGTAGTCACAGTAAGAATAGAGGGAGAGGTAATAGAAGCCCTATTAGACACAGGGGCAGATGATACAGTAATA
GAAAAGATAAATTTACCAGGAAAATGGAAACCAAAAATGATAGGGGGAATTGGAGGCTTTATCAAAGTAAGAC
AGTATGATCAGATAAGTATAGAAATTTGTGGAAAAAGGGCTATAGGTACAGTATTAGTAGGACCTACACCTGT
CAACATAATTGGACGAAATATGTTGACTCAGATTGGTTGTACTTTAAATTTTCCAATAAGTCCTATTGAAACT
GTACCAGTAAAATTAAAGCCAGGAATGGATGGGCCAAAGGTTAAACAATGGCCATTGACAGAAGAAAAAATAA
AAGCATTAACAGAAATTTGTACAGATATGGAAAAGGAAGGAAAAATTTCAAGAATTGGGCCTGAAAATCCATA
CAATACTCCAGTATTTGCCATAAAGAAAAAAGATAGTACTAAATGGAGAAAATTAGTAGATTTCAGAGAACTC
AATAAGAGAACTCAAGACTTCTGGGAGGTCCAATTAGGAATACCTCATCCCGCAGGGTTAAAAAAGAAAAAAT
CAGTAACAGTATTAGATGTGGGGGATGCATATTTTTCAGTTCCCTTAGATAAGGACTTTAGAAAGTATACTGC
CTTCACTATACCTAGTATAAATAATGAGACACCAGGAATTAGATATCAGTACAATGTGCTCCCACAGGGATGG
AAAGGATCACCAGCAATATTTCAGGCAAGTATGACAAAAATATTAGAGCCCTATAGAATAAATAATCCAGAGA
TGGTGATCTATCAATATATGGATGATTTATATGTAGGATCTGACTTAGAGATAGGGCAGCATAGAGCAAAAAT
AGAGGAGTTGAGAGCACATCTATTGAAGTGGGGATTTACCACACCAGACAAAAAGCATCAGAAAGAACCTCCA
TTTCTTTGGATGGGATATGAACTCCATCCTGACAAATGGACAGTCCAGCCTATACAGCTACCGGATAAAGACC
ACTGGACTGTCAATGATATACAGAAATTAGTGGGAAAACTAAATTGGGCAAGTCAGATTTATGCAGGAATTAA
AGTAAAACAACTGTGTAAACTCCTCAGGGGAGCCAAAGCACTAACAGATATAGTACCAATGACTGAGGAAGCA
GAATTGGAATTGGCAGAGAACAGGGAAATTTTAAAAGAACCTGTACATGGAGTGTATTATGACCCAACAAAAG
ACTTAGTAGCAGAAGTACAGAAACAAGGGCAAGGCCAATGGACATATCAAATTTATCAAGAGCCATTTAAAAA
TCTAAAAACAGGAAAATATGCAAAAAGGAAGTCTGCCCACACTAATGATGTAAAACAGTTAGCAGAGGTAGTG
CAAAAAATAGCTGTAGAAAGCATAGTAATTTGGGGGAAGACCCCTAAATTTAGACTACCCATACAAAGAGAAA
CATGGGAAACATGGTGGACAGAGTATTGGCAGGCTACCTGGATACCTGACTGGGAGTTTGTCAATACCCCTCC
TTTAGTAAAATTATGGTACCAGTTAGAGAAAGACCCCATAGTAGGGGCAGAAACTTTCTATGTAGATGGGGCA
GCCAATAGGGAAACTAAGCTAGGAAAAGCAGGGTATGTCACAGACAGAGGAAGACAAAAGGTTGTCTCCCTAA
CTGAGACAACAAATCAAAAGACTGAATTACAGGCAATATATCTAGCCTTGCAAGATTCAGGATCAGAAGTAAA
TATAGTGACAGACTCACAATATGCATTGGGAATTATTCAGGCACAACCAGACAGAAGTGAATCAGAATTAGTT
AATCAAGTAATAGAGAAGCTAATAGAAAAGGACAAGGTCTACCTGTCATGGGTACCAGCACATAAAGGGATTG
GAGGAAATGAACAAGTAGATAAATTGGTCAGTAATGGAATCAGAAGAGTACTATTTTTAGATGGCATAGATAA
AGCCCAAGAAGATCACGAAAGATATCACAGCAATTGGAGAACAATGGCTAGTGATTTTAATCTGCCACCTATA
GTAGCAAAAGAAATAGTGGCCAGCTGTGATAAATGTCAGCTGAAAGGGGAAGCCATACATGGACAAATAGACT
GTAGTCCAGGAATATGGCAATTAGATTGTACACATTTAGAAGGAAAAATTATCCTGGTAGCAGTCCATGTAGC

CAGTGGCTATATAGAAGCAGAAGTTATCCCAGCAGAAACAGGATCGGAGACAGCATACTTTATATTAAAATTA
GCAGGAAGATGGCCAGTGAAAGTAATACACACAGACAATGGCCCCAATTTTATCAGTGCTGCAGTAAAGGCAG
CATGTTGGTGGGCAAATGTCACACAAGAATTTGGAATTCCCTACAATCCCCAAAGCCAAGGAGTAGTGGAATC
TATGAATAAAGAACTAAAGAAAATTATAGGACAGGTCAGGGATCAAGCTGAACATCTTAAGACAGCAGTACAG
ATGGCAGTATTCATTCACAATTTTAAAAGAAAAGGGGGGATTGGGGGGTACAGTGCAGGGGAAAGAATAATAG
ACATAATAGCATCAGATATACAAACTAAAGAACTACAAAAACAAATTACAAAAATTCAAAATTTTCGGGTTTA
TTACAGGGACAGCAGAGACCCCATTTGGAAAGGACCAGCAAAACTACTCTGGAAAGGTGAAGGGGCAGTAGTA
ATACAGGACAATAGTGATATAAAAGTAGTACCAAGAAGAAAAGCAAAAATCATTAAGGATTATGGAAAACAGA
TGGCAGGTGATGATTGTGTGGCAGGTAGACAGGATGAGGATTAGAACATGGAACAGTTTAGTAAAACATCATA
TGTATGTCTCTAGGAAAGCTAAGGGTTGGTTTTATAGACATCACTATGAAAGTAGGCATCCAAAAGTGAGTTC
AGAAGTACACATCCCATTAGGGGATGCTATGTTAGTAGTAAGAACATATTGGGGTCTGCTTACAGGAGAAAGA
GATTGGCACTTGGGTCATGGGGTCTCCATAGAATGGAGGCAGAAAAGATATAGCACACAAATAGATCCTGAAC
TAGCAGACCAACTAATTCACTCGCATTATTTTGACTGTTTTACAGACTCTGCTATAAGAAAAGCCACATTAGG
ACAAATAGTTAGACCTAAGTGTGAATATCAAGCAGGACATAATAAGGTAGGATCGCTACAATATTTGGCACTA
AAAGCATTAGTAACACCAGAAAGGACAAAGCCACCTTTACCTAGTGTTAAGAAGTTAACAGAAGACAGATGGA
ACAAGTCCCACAAGACCAGGGGCCACAGAGGGAGCCGTTCAATGAATGGACACTAGAACTGTTAGAAGAGCTT
AAGGATGAAGCTGTTAGACATTTTCCTAGGCCGTGGCTCCATGGATTAGGACAACATATCTACAACACATATG
GGGATACTTGGGAAGGGGTTGTAGCTATAATAAGAATTTTGCAACAACTACTGTTTATTCATTTCAGAATTGG
GTGTCAACATAGCAGAATAGGCATTATTCCAGGGAGAAGAGGCAGGAATGGAGCTGGTAGATCCTAGCCTAGA
GCCATGGAACCAACCGGGAAGTCAGCCTACAACTGCTTGTAACAATTGTTACTGTAAAATATGCTGCTGGCAT
TGCCAATTATGCTTTCTGAACAAGGGCTTAGGCATCTCCTATGGCAGGAAGAAGCGGAGACCCCGACGAAGAA
CTCCTCAAAGCCGTCAGGATCATCAAAATCCTGTACCAAAGCAGTGAGTAGTAATAATTAGTATATGTGATGA
AATCTTTAGGAATAGCTGCAATAGTAGGATTAGTAGTAGCATTCATAGCAGCCATAGTTGTGTGGACCATAGT
GTTTATTGAATATAGAAAAATAAGGAAACAGAAGAAAATAGACAAGATACTTGATAGAATAAGAGAAAGAGCA
GAAGACAGTGGCAATGAGAGTGATGGGGACACAGAAGAATTATACACTCTTATGGAGGTGGGGTATGATAATA
TTTTGGATAATGATAATTTGTAATGCTAAAGACTTGTGGGTCACAGTCTATTATGGGGTACCAGTGTGGAGAG
ACGCAGAGACCACCCTATTTTGTGCATCAGATGCCAAAGCATATGATTCAGAAGTACATAATGTCTGGGCTAC
ACATGCCTGTGTACCCACAGACCCTAACCCACAAGAAATACATTTGGAAAATGTAACAGAAAATTTTAACATG
TGGAAAAATACCATGGTGGAGCAGATGCATGAAGATATAATCAGTCTCTGGGACCAAAGCCTAAAGCCATGTG
TAAAGTTAACCCCGCTCTGCGTTACTTTAAATTGTAGTGACTATACATATAACTCCACCAGCTATGTTAAATC
CACCAACAACTCTGAAATGCAAGAAATAAAAAACTGCTCTTTCAATATAACCACAGAATTAAGAGATAAGAAA
CAGAAAATGTATGCACTTTTTTATAAACTTGATGTAAAACAACTTGATAATAATAATCAGACGTATAGTTTAA
TAAATTGTAACACCTCAACCATTACACAGGCTTGTCCAAAGGTATCCTTTGAGCCAATTCCCATACATTATTG
TGCCCCAGCTGGTTTTGCAATTCTAAAGTGTAAGGATAAGAGGTTCAATGGAACAGGGCCATGCAAGAATGTC
AGCACAGTACAATGCACACATGGAATCAAGCCAGTAGTATCAACTCAACTGCTGTTAAATGGCAGTCTAGCAG
AAGAAGAGGTAGTGATTAGATCTGAGAATATCACAAACAATGCCAAGACCATAATAGTACAGTTGAATAAGCC
TGTAAGAATTAATTGTACCAGAACTGGTAACAATACAAGAAAAAGTGTACGTATAGGACCAGGGCAAACATAC
TATGCAACAGGTGAAATAATAGGAGATATAAGAAAAGCATATTGTAATGTCAGTAAAACAGAATGGGATGAGG
CTTTACAACAGGTAGTCACACAATTAAAGAAGCATTTCAATACCACAATAATCTTTAATAGTGCCTCAGGAGG
AGATCTAGAAATTACAACACATAGTTTTAATTGTAGAGGAGAATTTTTTTATTGCAATACAACAAACCTGTAT
AATAGCACTTGGAATAATAGCACTTGGAATGGCAATACCAGTACAAATCTCACAGAGTCAAATGATACTATAA
CTCTCCAATGCAGACTAAAGCAATTTATAAAAATGTGGCAGAGAGTAGGACAAGCAATGTATGCCCCTCCTAT
CCAAGGAGAAATAAGGTGTGATTCAAACATTACTGGACTATTATTAACAAGAGATGGAGGGAATAATAGGACA
AATGAGACCTTTAGGCCTGGAGGAGGAGATATGAGGGACAATTGGAGAAGTGAATTATATAAGTATAAAGTAG
TAAAAATTGAACCAATAGGTGTGGCACCAACCAGGGCAAAAAGAAGAATGGTGGAGAGAGAAAAAAGAGCAGT
TGGACTGGGAGCTGTTTTCCTTGGGTTCTTAGGAGCAGCAGGAAGCACTATGGGCGCGGCGTCAATAACGCTG
ACGGTACAGGCCAGGCAATTATTGTCTGGTATAGTGCAACAGCAGAGCAATTTGCTGAAGGCTATAGAGGCTC
AACAGCAAATGTTGAGACTCACGGTCTGGGGCATTAAGCAGCTCCAGGCAAGAGTCCTGGCTCTGGAAAGATA
CCTAAAGGATCAACAGCTCCTAGGAATTTGGGGCTGCTCTGGAAAACTCATCTGCACCACTGGTGTACGCTGG
AACTCAAGCTGGAGTAATAAAACTTATGAGGACATATGGGATAACATGACCTGGGTGCAATGGGAGAGGGAAA
TTAGCAATTACACAAACACAATATATACTCTACTTGAAGAATCGCAGAACCAGCAGGAAAAAAATGAACAAGA
CTTATTGGCATTGGACAAATGGACAAATCTGTGGAATTGGTTTAACATATCAAATTGGTTATGGTATATAAAA
ATATTTATAATGATAGTAGGAGGTTTAATAGGTTTAAGAATAGTTTTTACTGTGCTTACTATAATAAGGAGAG
TTAGGCAGGGATACTCACCTTTGTCGTTCCAGACCCTTCCCCTACACCAGAGGGAACCCGACAGGCCCGAAGG

AATCGAAGAAGGAGGTGGCGAGCAAGACAGAGGCAGATCAGTAAGATTAGTGAGCGGATTCTTAGCTCTTGCC
TGGGACGACCTACGGAGCCTGTGCCTCTTCAGCTACCACCAATTGAGAAACTTTGCCTTGATTGCAGCGAGAA
CTGTGGAACTCCTGGGACACAGCAGTCTCAAGGGACTGAGACTGGGGTGGGAAGCCCTCAAATATCTGTGGAA
TCTTCTGTCATACTGGGGTCAGGAACTAAAGAATAGTGCTATTAATTTGTTTGATACCATAGCAATAGCAGTA
GCTAACTGGACAGACAGAGGTATAGAAATAGGACAAAGAGCTGGCAGAGCTATTGGCAACATACCTAGAAGAA
TCAGACAGGGCCTAGAAAGAGCTTTGCTATAACATGGGTGGCAAATGGTCAAAAAGCAGCATAGTGGGATGGC
CTAAGATTAGGGAAAGAATGAGACAAAGCCCTCCAACAGGAAGCCCTCCAGCAGCGACAGGAACAGGAGCAGT
ATCCCCAGCAGCAACAGGAACAAGAGCAGTATCTCAAGATTTAGCTAGACATGGAGCAATCACAAGCAGTAAT
ACAGCCTCTACTAATCCTGCTTGTGCCTGGCTGGAAGCACAAGAGGAAGAGGATGAGGTAGGCTTTCCAGTCA
GACCACAGGTACCTTTGAGACCAATGACTTATAAGCTAGCTTTCGATCTCAGCTTCTTTTTAAAACAAAAGGG
GGGACTGGAAGGGTTAGTTTGGTCCCAGAAAAGACAAGATATCCTTGATCTGTGGATGTATCACACACAAGGA
ATCTTCCCAGATTGGCAGAACTACACACCAGGGCCAGGGACTAGATTCCCACTGACCTTTGGGTGGTGCTTCA
AACTAGTACCACTAGATCCAGCAGAGGTAGAGGCAGCTAATGAAGGAGAGAACAACAGCTTATTACACCCCAT
CAGTCAACATGGGATGGAAGACGGGGACAGAGAGGTGCTGGTCTGGAGATTTGACAGCAGCCTGGCAAGAGAA
CACAAAGCCCGAGAGCTGCATCCGGAGTTCTATAAAGACTGCTGACACAGAAGTTGCTGACTGGGACTTTCCA
CTGGGGACTTTCCGGGGAGGTGTGGTTGGGGAGGAGTTTGGGAGTGGCTAACCCTCAGATGCTGCATATAAGC
AGCTGCTTCTCGCCTGTACGTGGTCTCTCTTGCTAGACCAGATTTGAGCCTGGGAGCTCTCTGGCTAGTTAAG
GGACCCACTGCTTAAGCCTCAATAAAGCTTGCCT

>X-22B HIV-1 genome, derived from RNA genomic sequence
TAGCAGTGGCGCCCGAACAGGGACTTGAAGTTAATAGGGACTCGAAAGCGAAAGTTCCAGAGAAGCTCTCTCG
ACGCAGGGACTCGGCTTGCTGAAGTGCACGCAGCAAGAGGCGAGAGCGGCGACTGGTGAGTACGCCAAATTTC
GACTAGCGGAGGCTAGAAGGAGAGAGATGGGTGCGAGAGCGTCGATACTAAGTGGGGGAAAATTAGATGCATG
GGAGAAAATTCGGTTAAGGCCAGGGGGAAAGAAACAATATCGACTAAAACATTTAGTATGGGCAAGCAGGGAG
TTGGAAAGATTTGCACTTAATCCTAGTCTTTTAGAAACAGCAGAAGGATGTCAACAACTGATAGAACAGTTAC
AATCAACTCTCAGGACAGGATCAGAAGAACTTAAATCATTATTTAATACAATAGCAACCCTTTGGTGCGTACA
TCAAAAGATAGACATAAAAGACACCAAGGAAGCCTTAGATAAAATAGAGGAAGAACAAAATAAGAGCAAGCAA
AGGACACAGCAAAAGACACAGCAGGCAGCAGCTGCCGCAGGAAGCAGCAGCCAAAATTACCCTATAGTGCAAA
ATGCACAAGGGCAAATGATACATCAGGCCATGTCACCTAGAACTCTGAACGCATGGGTGAAGGTAGTAGAGGA
GAAGGCTTTCAGCCCAGAAGTAATACCCATGTTTACAGCATTATCAGAAGGAGCCACCCCACAAGACTTAAAT
ATGATGCTAAACATAGTGGGGGGACACCAGGCAGCAATGCAGATGTTAAAAGATACCATCAATGAGGAAGCTG
CAGAATGGGACAGGACACATCCTGTACATGCAGGGCCTGCTCCACCAGGCCAGATGAGAGAACCAAGGGGAAG
TGACATAGCAGGAACTACTAGTACCCTTCAAGAACAAATAGGATGGATGACAAGCAATCCACCTATCCCAGTG
GGAGAAATCTATAAAAGATGGATAGTCCTGGGATTAAATAAAATAGTAAGAATGTATAGCCCTGTCAGCATTT
TGGATATAAGACAAGGGCCAAAAGAACCCTTTAGAGACTATGTAGATAGGTTCTTTAAAACTTTAAGAGCTGA
ACAAGCTACACAGGAGGTAAAAAACTGGATGACAGAAACCTTGCTGGTCCAAAATGCGAATCCAGATTGTAAG
ACCATTCTAAGAGGATTAGGACCAGGGGCTACATTAGAAGAAATGATGACAGCATGTCAGGGAGTGGGAGGAC
CTGGCCATAAAGCAAGGGTTTTGGCTGAGGCCATGAGTCAAGCGCAACAGTCCAACATAATGATGCAGAAGGG
AAACTTTAGGGGCCAGAGAACAATAAAGTGTTTCAATTGTGGCAAAGAAGGACACCTAGCCAGAAACTGCAAG
GCCCCTAGGAAAAGGGGTTGTTGGAAATGTGGTAAGGAAGGACACCAAATGAAAGACTGTACTGAAAGACAGG
CTAATTTTTTAGGGAAAATTTGGCCTTCCAACAAGGGGAGGCCAGGAAATTTTCCTCAGAGCAGACCGGAACC
AACGGCCCCACCACTAGAGAACTTTGGAGTGGGGGAAGAGATACCCCCCCTTCAGAAGCAGGAACCCTCCTCT
CCGAAACAGGAACCGGGGGACAAGGGACTATATCCTCCTTTAACCTCCCTCAAATCACTCTTTGGCAACGACC
AGTAGTCACAGTAAGAATAGAGGGAGAGGTAATAGAAGCCCTATTAGACACAGGGGCAGATGATACAGTAATA
GAAAAGATAAATTTACCAGGAAAATGGAAACCAAAAATGATAGGGGGAATTGGAGGCTTTATCAAAGTAAGAC
AGTATGATCAGATAAGTATAGAAATTTGTGGAAAAAGGGCCATAGGTACAGTATTAGTAGGACCTACACCTGT
CAACATAATTGGACGAAATATGTTGACTCAGATTGGTTGTACTTTAAATTTTCCAATAAGTCCTATTGAAACT
GTACCAGTAAAATTAAAGCCAGGAATGGATGGCCAAAGGTTAAACAATGGCCATTGACAGAAGAAAAAATAA
AAGCATTAACAGAAATTTGTACAGATATGGAAAAAGAAGGAAAAATTTCAAGAATTGGGCCTGAAAATCCATA
CAATACTCCAGTATTTGCCATAAAGAAAAAAGATAGTACTAAATGGAGAAAATTAGTAGATTTCAGAGAACTC
AATAAGAGAACTCAAGACTTCTGGGAGGTCCAATTAGGAATACCTCATCCCGCAGGGTTAAAAAAGAAAAAAT
CAGTAACAGTATTAGATGTGGGGGATGCATATTTTTCAGTTCCCTTAGATAAGGACTTTAGAAAGTATACTGC
CTTCACTATACCTAGTATAAATAATGAGACACCAGGAATTAGATATCAGTACAATGTGCTCCCACAGGGATGG
AAAGGATCACCAGCAATATTTCAGGCAAGTATGATAAAAATATTAGAGCCCTATAGAATAAATAATCCAGAGA
TGGTGATCTATCAATATATGGATGATTTATATGTAGGATCTGACTTAGAGATAGGGCAGCATAGAGCAAAAAT
AGAGGAGTTGAGAGCACATCTATTGAAGTGGGGATTTACCACACCAGACAAAAAGCATCAGAAAGAACCTCCA
TTTCTTTGGATGGGATATGAACTCCATCCTGACAAATGGACAGTCCAGCCTATACAGCTACCGGATAAAGACC
ACTGGACTGTCAATGATATACAGAAATTAGTGGGAAAACTAAATTGGGCAAGTCAGATTTATGCAGGAATTAA
AGTAAGACAACTGTGTAAACTCCTCAGGGGAGCCAAAGCACTAACAGATATAGTATCAATGACTGAGGAAGCA
GAATTGGAATTGGCAGAGAACAGGGAAATTTTAAAAGAACCTGTACATGGAGTGTATTATGACCCAACAAAAG
ACTTAGTAGCAGAAGTACAGAAACAAGGGCAAGGCCAATGGACATATCAAATTTATCAAGAGCCATTTAAAAA
TCTAAAAACAGGAAAATATGCAAAAAGGAAGTCTGCCCACACTAATGATGTAAAACAGTTAGCAGAGGTAGTG
CAAAAAATAGCTGTAGAAAGCATAGTAATTTGGGGGAAGACCCCTAAATTTAGACTACCCATACAAAGAGAAA
CATGGGAAACATGGTGGACAGAGTATTGGCAGGCTACCTGGATACCTGACTGGGAGTTTGTCAATACCCCTCC
TTTAGTAAAATTATGGTACCAGTTAGAGAAAGACCCCATAGTAGGGGCAGAAACTTTCTATGTAGATGGAGCA
GCCAATAGGGAAACTAAGCTAGGAAAAGCAGGGTATGTCACAGACAGAGGAAGACAAAAGGTTGTCTCCCTAA
CTGAGACAACAAATCAAAAGACTGAATTACAGGCAATATATCTAGCCTTGCAAGATTCAGGATCAGAAGTAAA
TATAGTGACAGACTCACAATATGCATTGGGAATTATTCAGGCACAACCAGACAGAAGTGAATCAGAATTAGTT
AATCAAGTAATAGAGAAGCTAATAGAAAAGGACAAGGTCTACCTGTCATGGGTACCAGCACATAAAGGGATTG
GAGGAAATGAACAAGTAGATAAATTGGTCAGTAATGGAATCAGAAGAGTACTATTTTTAGATGGCATAGATAA
AGCCCAAGAAGATCACGAAAAATATCATAGCAATTGGAGAACAATGGCTAGTGATTTTAATCTACCACCTATA
GTAGCAAAAGAAATAGTGGCCAGCTGTGATAAATGTCAGCTGAAAGGGGAAGCCATACATGGACAAGTAGACT
GTAGTCCAGGAATATGGCAATTAGATTGTACACATTTAGAAGGAAAAATTATCCTGGTAGCAGTCCATGTAGC

CAGTGGCTATATAGAAGCAGAAGTTATCCCAGCAGAAACAGGATCGGAGACAGCATACTTTATATTAAAATTA
GCAGGAAGATGGCCAGTGAAAGTAATACACACAGACAATGGCCCCAATTTTACCAGTACTGCAGTAAAGGCAG
CATGTTGGTGGGCAAATGTCACACAAGAATTTGGAATTCCCTACAATCCCCAAAGCCAAGGAGTAGTGGAATC
TATGAATAAAGAACTAAAGAAAATTATAGGACAGGTCAGGGATCAAGCTGAACATCTTAAGACAGCAGTACAG
ATGGCAGTATTCATTCACAATTTTAAAAGAAAAGGGGGGATTGGGGGGTACAGTGCAGGGGAAAGAATAATAG
ACATAATAGCATCAGATATACAAACTAAAGAACTACAAAAACAAATTACAAAAATTCAAAATTTTCGGGTTTA
TTACAGGGACAGCAGAGACCCCATTTGGAAAGGACCAGCAAAACTACTCTGGAAAGGTGAAGGGGCAGTAGTA
ATACAGGACAATAGTGATATAAAAGTAGTACCAAGAAGAAAAGCAAAAATCATTAAGGATTATGGAAAACAGA
TGGCAGGTGATGATTGTGTGGCAGGTAGACAGGATGAGGATTAGAACATGGAACAGTTTAGTAAAACATCATA
TGTATGTCTCTAAGAAAGCTAAGGGTTGGTTTTATAGACATCACTATGAAAGTAGGCATCCAAAAGTGAGTTC
AGAAGTACACATCCCATTAGGGGATGCTATGTTAGTAGTAAGAACATATTGGGGTCTGCTTACAGGAGAAAGA
GATTGGCACTTGGGTCATGGGGTCTCCATAGAATGGAGGCAGAAAAGATATAGCACACAAATAGATCCTGAAC
TAGCAGACCAACTAATTCACTCGCATTATTTTGACTGTTTTACAGACTCTGCTATAAGAAAAGCCACATTAGG
ACAAATAGTTAGACCTAAGTGTGAATATCAAGCAGGACATAATAAGGTAGGATCGCTACAATATTTGGCACTA
AAAGCATTAGTAACACCAGAAAGGACAACGCCACCTTTACCTAGTGTTAAGAAGTTAACAGAAGACAGATGGA
ACAAGTCCCACAAGACCAGGGGCCACAGAGGGAGCCGCTCAATGAATGGACACTAGAACTGTTAGAAGAGCTT
AAGGAAGAAGCTGTTAGACATTTTCCTAGGCCGTGGCTCCATGGATTAGGACAACATATCTACAACACATATG
GGGATACTTGGGAAGGGGTTGTAGCTATAATAAGAATTTTGCAACAACTACTGTTTATTCATTTCAGAATTGG
GTGTCAACATAGCAGAATAGGCATTATTCCAGGGAGAAGAGGCAGGAATGGAGCTGGTAGATCCTAGCCTAGA
GCCATGGAACCATCCGGGAAGTCAGCCTACAACTGCTTGTAACAATTGTTACTGTAAAATATGCTGCTGGCAT
TGCCAATTATGCTTTTTGAACAAGGGCTTAGGCATCTCCTATGGCAGGAAGAAGCGGAGACCCCGACGAAGAA
CTCCTCAAAGCCGTCAAGATCATCAAAATCCTGTACCAAAGCAGTGAGTAGTAATAATTAGTATATGTAATGA
AATCTTTAGGAATAGCTGCAATAGTAGGATTAGTAGTAGCATTCATAGCAGCCATAGTTGTGTGGACCATAGT
GTTTATTGAATATAGAAAAATAAGGAAACAGAAGAAAATAGACAAGATACTTGATAGAATAAGAGAAAGAGCA
GAAGACAGTGGCAATGAGAGTGATGGGGACACAGAAGAATTATACACTCTTATGGAGGTGGGGTATGATAATA
TTTTGGATAATGATAATTTGTAATGCTAAAGACTTGTGGGTCACAGTCTATTATGGGGTACCAGTGTGGAGAG
ACGCAGAGACCACCCTATTTTGTGCATCAGATGCCAAAGCATATGATTCAGAAGTACATAATGTCTGGGCTAC
ACATGCCTGTGTACCCACAGACCCTAACCCACAAGAAATACATTTGGAAAATGTAACAGAAAATTTTAACATG
TGGAAAAATACCATGGTGGAGCAGATGCATGAAGATATAATCAGTCTCTGGGACCAAAGCCTAAAGCCATGTG
TAAAATTAACCCCGCTCTGCGTTACTTTAAATTGTAGTGACTATAACTCCACCAGATATGATAAATCCACCAA
CAACTCTGAAATGCAAGAAATAAAAAACTGCTCTTTCAATATAACCACAGAATTAAGAGATAAGAAACAGAAA
ATGTATGCACTTTTTTATAAACTTGATGTAAAACAACTTGATAATGATAATCAGACGTATAGTTTAATAAATT
GTAACACCTCAACCATTACACAGGCTTGTCCAAAGGTATCCTTTGAGCCAATTCCCATACATTATTGTGCCCC
AGCTGGTTTTGCAATTCTAAAGTGTAAGGATAAGATGTTCAATGGAACAGGGCCATGCAAGAATGTCAGCACA
GTACAATGCACACATGGAATCAAGCCAGTAGTATCAACTCAACTGCTGTTAAATGGCAGTCTAGCAGAAGGAG
AGGTAGTGATTAGATCTGAGAATATCACAAACAATGCCAAGACCATAATAGTACAGTTGAATAAGCCTGTAAG
AATTAATTGTACCAGAACTAGTAACAATACAAGAAAAAGTGTACGTATAGGACCAGGGCAAACATACTATGCA
ACAGGTGAAATAATAGGAGATATAAGAAAAGCACATTGTAATGTCAGTAAAACAGAATGGGATAAGGCTTTAC
AACAGGTAGCCACACAATTAAAGAAGCATTTCAATACCACAATAATCTTTAATAATGCCTCAGGAGGAGATCT
AGAAATTACAACACATAGTTTTAATTGTAGAGGAGAATTTTTTTATTGCAATACAACAAACCTGTATAATAGC
ACTTGGAATGATAGCACTATCATTACAAATCTCACAGGGTCAAATGATACTATAACTCTCCAATGCAGACTAA
AGCAATTTATAAAAATGTGGCAGAGAGTAGGACAAGCAATGTATGCCCCTCCTATCCAAGGAGAAATAAGGTG
TGATTCAAACATTACTGGACTATTATTAACAAGAGATGGAGGGAATAATGGGACAAATGAGACCTTTAGGCCT
GGAGGAGGAGATATGAGGGACAATTGGAGAAGTGAATTATATAAGTATAAAGTAGTAAAAATTGAACCAATAG
GTGTGGCACCAACCAGGGCAAAAAGAAGAATGGTGGAGAGAGAAAAAAGAGCAGTTGGACTGGGAGCTGTTTT
CCTTGGGTTCTTAGGAGCAGCAGGAAGCACTATGGGCGCAGCGTCGATAACGCTGACGGTACAGGCCAGGCAA
TTATTGTCTGGTATAGTGCAACAGCAGAGCAATTTGCTGAAGGCTATAGAGGCTCAACAGCAAATGTTGAGAC
TCACGGTCTGGGGCATTAAGCAGCTCCAGGCAAGAGTCCTGGCTCTGGAAAGATACCTAAAGGATCAACAGCT
CCTAGGAATTTGGGGCTGCTCTGGAAAACTCATCTGCACCACTGGTGTACGCTGGAACTCAAGCTGGAGTAAT
AAAACTTATGAGGACATATGGGATAACATGACCTGGGTGCAATGGGAGAGGGAAATTAGCAATTACACAAACA
CAATATATACTCTACTTGAAGAATCGCAGAACCAGCAGGAAAAAAATGAACAAGACTTATTGGCATTGGAAAA
ATGGACAAATCTGTGGAATTGGTTTAACATATCAAATTGGTTATGGTATATAAAAATATTTATAATGATAGTA
GGAGGTTTAATAGGTCTAAGAATAGTTTTTACTGTGCTTACTATAATAAGGAGAGTTAGGCAGGGATACTCAC
CTTTGTCGTTCCAGACCCTTCCCCTACACCAGAGGGAACCCGACAGGCCCGAAGGAATCGAAGAAGGAGGTGG

CGAGCAAGACAGAGGCAGATCAGTAAGATTAGTGAGCGGATTCTTAGCTCTTGCCTGGGACGACCTACGGAGC
CTGTGCCTTTTCAGCTACCACCAATTGAGAAACTTTGCCTTGATTGCAGCGAGAACTGTGGAACTCCTGGGAC
ACAGCAGTCTCAAGGGACTGAGACTGGGGTGGGAAGCCCTCAAATATCTGTGGAATCTTCTGTCATACTGGGG
TCAGGAACTAAAGAATAGTGCTATTAATTTGTTTGATACCATAGCAATAGCAGTAGCTAACTGGACAGACAGA
GGTATAGAAATAGGACAAAGAGTTGGCAGAGCTATTGGCAACATACCTAGAAGAATCAGACAGGGCCTAGAAA
GAGCTTTGCTATAACATGGGTGGCAAATGGTCAAAAAGCAGCATAGTGGGATGGCCTAAGATTAGGGAAAGGA
TGAGACGAACCCCTCCAACAGGAAGCCCTCCAGCAGCAACAGAAACAGGAGCAGTATCCCCAGCAGCAACAGG
AACAGGAGCAGTATCTCAAGATTTAGCTAGACATGGAGCAATCACAAGCAGTAATACAGCCTCTACTAATCCT
GCTTGTGCCTGGCTGGAAGCACAAGAGGAAGAGGATGAGGTAGGCTTTCCAGTCAAACCACAGGTACCTTTGA
GACCAATGACTTATAAACTAGCTTTCGATCTCAGCTTCTTTTTAAAAGAAAAGGGGGGACTGGAAGGGTTAGT
TTATTCCCAGAAAAGACAAGATATCCTTGATCTGTGGATGTATCACACACAAGGAATCTTCCCAGATTGGCAG
AATTACACACCAGGGCCAGGGACTAGATACCCACTGACCTTTGGGTGGTGCTTCAAACTAGTACCACTAGATC
CAGCAGAGGTAGAGGCAGCTAATGAAGGAGAGAACAACAGCTTATTACACCCCATCAGTCAACATGGGATGGA
AGACGAGGACAGAGAGGTGCTGGTCTGGAGATTTGACAGCAGCCTGGCAAGAGAACACAAAGCCCGAGAGCTG
CATCCGGAGTTCTATAAAGACTGCTGACACAGAAGTTGCTGACTGGGACTTTCCACTGGGGACTTTCCGGGGA
GGTGTGGTTGGGGAGGAGTTTGGGAGTGGCTAACCCTCAGATGCTGCATATAAGCAGCTGCTTCTCGCCTGTA
CGTGGTCTCTCTTGCTAGACCAGATGTGAGCCTGGGAGCTCTCTGGCTAGTTAAGGGACCCACTGCTTAAGCC
TCAATAAAGCTTGCCT
